# Photochemical carboborylation and three-component difunctionalization of α,β-unsaturated ketones with boronic acids *via* tosylhydrazones†

**DOI:** 10.1039/d4sc06537a

**Published:** 2024-11-06

**Authors:** Álvaro Valdés-Maqueda, Manuel Plaza, Carlos Valdés

**Affiliations:** a Departamento de Química Orgánica e Inorgánica, Instituto Universitario de Química Organometálica “Enrique Moles”, Instituto de Investigación Sanitaria del Principado de Asturias (ISPA), Centro de Innovación en Química Avanzada (ORFEO-CINQA), Universidad de Oviedo C/ Julián Clavería 8 33006 Oviedo Spain plazamanuel@uniovi.es acvg@uniovi.es

## Abstract

The reactions of cyclic α,β-unsaturated *N*-tosylhydrazones and alkylboronic acids promoted by 370–390 nm light in the presence of a base give rise to allylic boronic acids that can be trapped as the corresponding pinacolboronates by treatment with pinacol. This reaction features wide scope regarding both coupling partners and functional group tolerance, allowing for the incorporation of a variety of natural product-derived fragments. The allylic boronic acids can be also reacted in a one-pot process with aldehydes, to produce homoallylic alcohols with very high diastereoselectivity. A three-component one-pot procedure has been developed revealing that the methodology is a powerful tool for the generation of structural diversity that is accomplished by incorporation of an ample variety of each of the three elements. Moreover, from a synthetic perspective, in the reaction, the formation of two C–C bonds, at the carbonyl and the β positions of a α,β-unsaturated carbonyl, has been achieved in the three-component reaction.

## Abstract 2

The photochemical carboborylation of α,β-unsaturated tosylhydrazones with boronic acids gives tertiary allylboronates. A one pot sequence involving an aldehyde allylation provides a powerful three-component method for diversity oriented synthesis.

## Graphical abstract

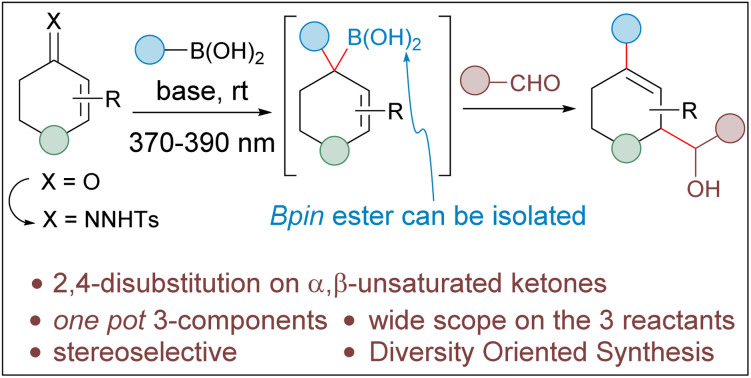

## Introduction

**Scheme 1:**
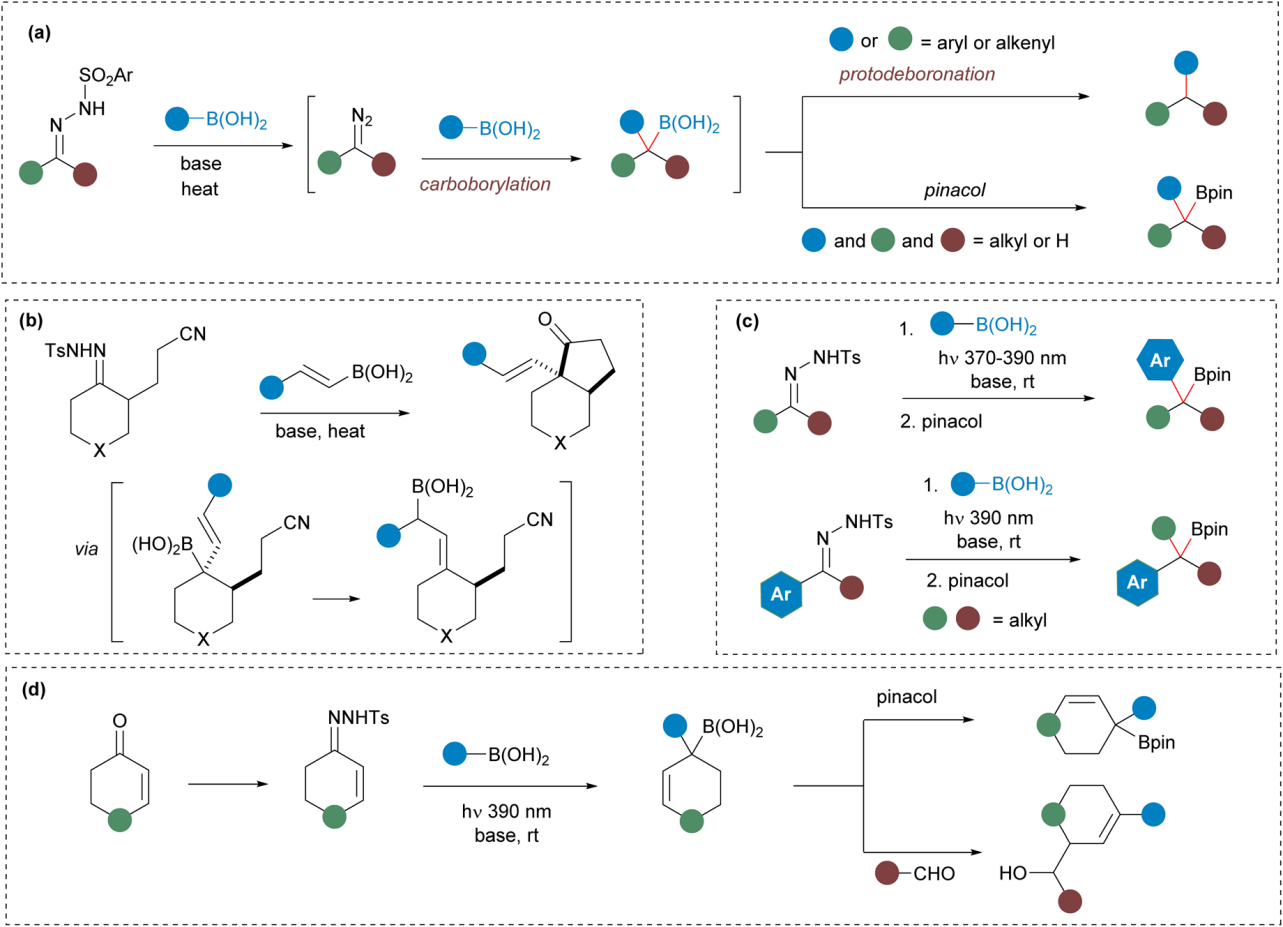
General picture of the reactions of *N*-sulfonylhydrazones with boronic acids: (a) homologation of boronic acids with *N*-sulfonylhydrazones under thermal conditions. (b) Domino cyclizations involving *N*-sulfonylhydrazones and alkenylboronic acids *via in situ* generated allylboronic acid. (c) Synthesis of benzylboronates by homologation of boronic acids under photochemical conditions. (d) This work.

The carbonyl moiety stands out as one of the most useful and versatile functional groups in organic synthesis. Carbonyls can be easily manipulated taking advantage of the electrophilic nature of the carbonyl carbon through a myriad of reactions including nucleophilic additions, condensations and olefination reactions. Nevertheless, chemical methodologies that allow the modification of carbonyls through nonconventional strategies, opening new synthetic opportunities, have been highly demanded over the years. In this context, the conversion of carbonyls into *N*-sulfonylhydrazones is a strategy that has garnered significant attention.^1^ By this method aldehydes and ketones are readily converted into diazoalkanes *in situ* that can further undergo an array of transformations through metal-catalyzed^2^ and also uncatalyzed reactions.^3^ In particular, the carboborylation of *N*-sulfonylhydrazones by reaction with boronic acids, reported by our group several years ago, is a very useful reaction within this area of research.^4^ In this process, the homologation of the boronic acid takes place upon reaction of the diazoalkane generated by decomposition of the *N*-sulfonylhydrazone. In classical thermal reactions with arylboronic acids, the homologated benzyl boronic acids undergo protodeboronation to deliver the products resulting from a reductive cross-coupling (Scheme 1(a)), a synthetic transformation that has found wide applications in organic synthesis.^5^ In contrast, reactions that generate alkylboronic acids can be isolated as boronates amenable for further manipulation (Scheme 1(a)).^6^ Moreover, the intermediate boronic acid can be also trapped intramolecularly to provide Csp^3^-rich carbo-^7^ and heterocycles^8^ in processes that involve the formation of two bonds on the hydrazonic carbon atom (Scheme 1(b)). On the other hand, very recently, we reported the synthesis of the elusive benzylboronates^9^ taking advantage of the ability of *N*-tosylhydrazones to undergo decomposition under photochemical conditions at rt (Scheme 1(c)).^10^ The photochemical reaction turned out to be very versatile as the benzylboronates could be obtained both by reactions of aryl-*N*-sulfonylhydrazones with alkylboronic acids and of alkyl-*N*-sulfonylhydrazones with arylboronic acids providing a broadly applicable methodology.^9^ Nevertheless, a remaining gap in this picture is still the generation and isolation of allylic boronates through a similar methodology from *N*-sulfonylhydrazones and boronic acids. Indeed, taking into consideration the high synthetic usefulness of allylic boronates,^11^ the access to these intermediates through such a simple synthetic strategy might be of high relevance.

The homologation of boronic acid derivatives with diazo compounds to produce allylic boronates, along with various synthetic applications, has been previously explored by Ley *et al.*^12^ More recently, Szabó *et al.* have developed an organocatalytic asymmetric version using alkenylboronic acid derivatives in combination with trifluoromethyldiazomethane and trimethylsilyldiazomethane.^13^ Despite the efficacy of these methodologies, their scope is limited to specific diazo compounds. We envisioned that the synthesis of allylboronates from α,β-unsaturated carbonyls *via* readily accessible *N*-sulfonylhydrazones, a strategy not yet achieved, could significantly expand the synthetic utility of these homologation reactions.^12^

We have previously observed that under the standard thermal conditions, the allylboronic acids generated by reaction of *N*-sulfonylhydrazones and alkenylboronic acids cannot be isolated due to spontaneous protodeboronation, resulting in the formation of α- or γ-protodeboronation products.^14^ Interestingly, the regioselectivity of the protodeboronation turned out to be determined by the nature of the substituents of both reaction partners. On the other hand, we have also showed that the allylboronic acids could be indeed trapped in an intramolecular fashion through bora-aza-ene reactions with nitriles (Scheme 1(b)).^7^ However, the isolation of allylic boronates obtained by homologation with *N*-sulfonylhydrazones, or their participation in subsequent intermolecular processes has not been achieved yet. Taking into account all these precedents, and considering the particular wide interest of allylboronates as intermediates in organic synthesis, we decided to investigate whether the concept we had employed in the photochemical synthesis of benzylboronates^9^ could be applied to the analogous allylic derivatives. Our results regarding their preparation as well as some synthetic applications are described below.

## Results and discussion

**Scheme 2:**
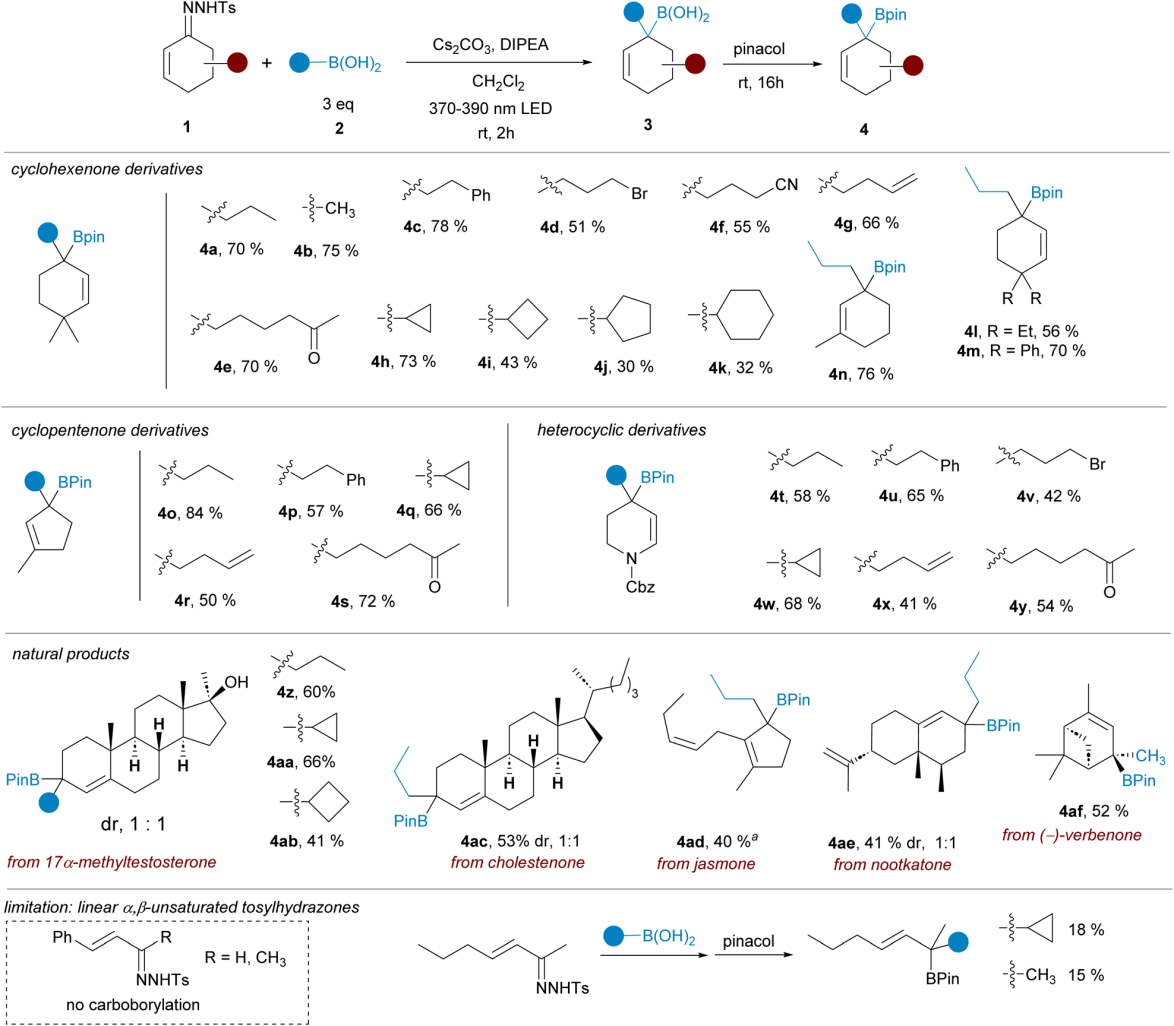
Synthesis of tertiary allylic pinacolboronates by reactions of cyclic α,β-*N*-tosylhydrazones with alkylboronic acids under photochemical activation. Standard reaction conditions: hydrazone 1 (0.2 mMol), boronic acid 3 (3 equiv.), Cs_2_CO_3_ (2 equiv.), DIPEA (2 equiv.), CH_2_Cl_2_ (2 mL), 390 or 370 nm LED lamp, rt, 2 h, then addition of pinacol (5 equiv.) rt, overnight. Isolated yields for the one-pot process are indicated. A 390 nm LED lamp was used unless otherwise indicated. ^*a*^A 370 nm LED lamp was used.

In our initial efforts we selected the reactions between cyclic α,β-unsaturated-*N*-tosylhydrazones 1 and alkylboronic acids 2 (Scheme 2). These transformations would deliver tertiary allylic boronates difficult to obtain by alternative methods.^15^ As prototype substrates 4,4-dimethylcyclohexen-2-one *N*-tosylhydrazone 1a and *n*-propylboronic acid 2a were chosen for the optimization round. After some experimentation it was found that the reaction in CH_2_Cl_2_ as solvent, with a combination of Cs_2_CO_3_ and DIPEA as bases and with irradiation with a 390 nm led lamp for 2 h, followed by treatment with pinacol provided the expected allylic pinacol boronate 4a, *via* the unstable intermediate boronic acid 3, with a satisfactory yield. Noteworthy, allylboronic acid derivatives are known to undergo 1,3-borotropic rearrangements,^16^ however, under these mild reaction conditions, the allylboronate 4 was isolated as unique regioisomer.

Having an appropriate set of conditions to achieve the carboborylation of the α,β-unsaturated *N*-tosylhydrazones, we set out to study the applicability of the process attending to both coupling partners. The scope regarding the boronic acids was explored employing hydrazone 1a. As represented in Scheme 2, the reaction is compatible with primary (4a, 4c) methyl (4b) and secondary (4h–k) alkylboronic acids, and also with boronic acids decorated with sensitive functional groups, such as an alkylbromide 4d, a nitrile 4f and an enolizable ketone 4e, revealing the high functional group tolerance of the transformation. Regarding the structure of the sulfonylhydrazone, derivatives of substituted cyclohexenones and cyclopentenones were assayed, providing the allylic boronates (4l–n) and (4o–s) respectively with similar reaction yields. Moreover, the reaction was applied successfully also to the heterocyclic *N*-tosylhydrazone derived from 2,3-dihydropyridin-4(1*H*)-one to deliver the resulting tetrahydropyridine boronic esters with the same wide scope (4t–y).

The carboborylation reaction was then applied to some natural products featuring an α,β-unsaturated ketone on their structure. In this manner the geminal disubstitution could be achieved for steroid derivatives 17α-methyltestosterone (4z, 4aa, 4ab) and cholestenone (4ac), as well as to the sesquiterpene nootkatone (4ae), although in these cases a 1 : 1 mixture of diastereoisomers was obtained. The reaction was also applied to *cis*-jasmone, an example of a 2,3-disubstituted cyclopentenone (4ad), delivering the expected allylic boronate even for this highly substituted system. Finally, the reaction was also performed with the *N*-tosylhydrazone of (−)-verbenone. Despite the high steric congestion, the carboborylation took place successfully to deliver the boronic ester 4af as a single stereoisomer. Under these conditions, the methodology is limited to cyclic α,β-unsaturated tosylhydrazones. No carboborylation products were detected with β-aryl-substituted α,β-unsaturated hydrazones. On the other hand, with a β-alkyl-α,β-unsaturated tosylhydrazone the allylic pinacolboronates could be isolated albeit in very poor yields. A more detailed discussion is included in the ESI.†

Allylic boronates are particularly versatile reagents which may undergo either α- or γ-substitution reactions. One notably interesting transformation is the allylation of carbonyl compounds.^17^ This reaction typically proceeds through a six-centered concerted transition state and takes place with high diastereoselectivity. Although this allylation reaction is well known, the application to these highly substituted allylboronates might be challenging. We decided to check whether the allylation could be applied directly to the homologated boronic acid 3 avoiding the isolation of the boronic ester. Thus, employing again the model substrates 4,4-dimethylcyclohexen-2-one *N*-tosylhydrazone 1a and *n*-propylboronic acid, once the photochemical reaction had concluded, an aldehyde was added to the reaction mixture in a one-pot fashion. To our delight, we observed that indeed the allylation reaction occurred smoothly to deliver the expected homoallylic alcohols 5 with high yields and diastereoselectivities.‡‡The three component reaction was also attempted mixing the *N*-tosylhydrazone, the boronic acid and the aldehyde at the beginning of the reaction and submitting the mixture to the photochemical conditions. However, the carboborylation reaction did not take place. Instead, the homologated ketone derived from the reaction of the aldehyde with the diazoalkane generated from the tosylhydrazone was the main product obtained as reported by König *et al.* (ref. 10).

The three-component one-pot reaction turned out to be highly general for aromatic aldehydes containing electron-donating (5a, 5b, 5h), electron-withdrawing (5c, 5d) and halogen (5e, 5f) substituents, and includes also a highly hindered *o*,*o*-disubstituted system (5g). Moreover, heteroaromatic aldehydes are also well tolerated (5i–k). It is important to note that the allylation reaction in many cases is a more efficient way to trap the boronic acid than the formation of the pinacol boronic ester, as several examples delivered higher yields than the 70% isolated yield obtained for pinacol boronic ester 4a. The reaction could be applied also to the α,β-unsaturated aldehydes *meta*-acrolein (5l) and cinnamaldehyde (5m).

However, under these reaction conditions the three-component-one-pot reaction seems to be limited to aromatic and α,β-unsaturated aldehydes, while the application to *n*-butanal and 4-methoxyacetophenone as examples of an alkylic aldehyde and an aromatic ketone respectively, led to the obtention of the allylic alcohol derived from the spontaneous oxidation of the allylic boronic acid, as the main product, without the incorporation of the fragment coming from the carbonyl.

**Scheme 3:**
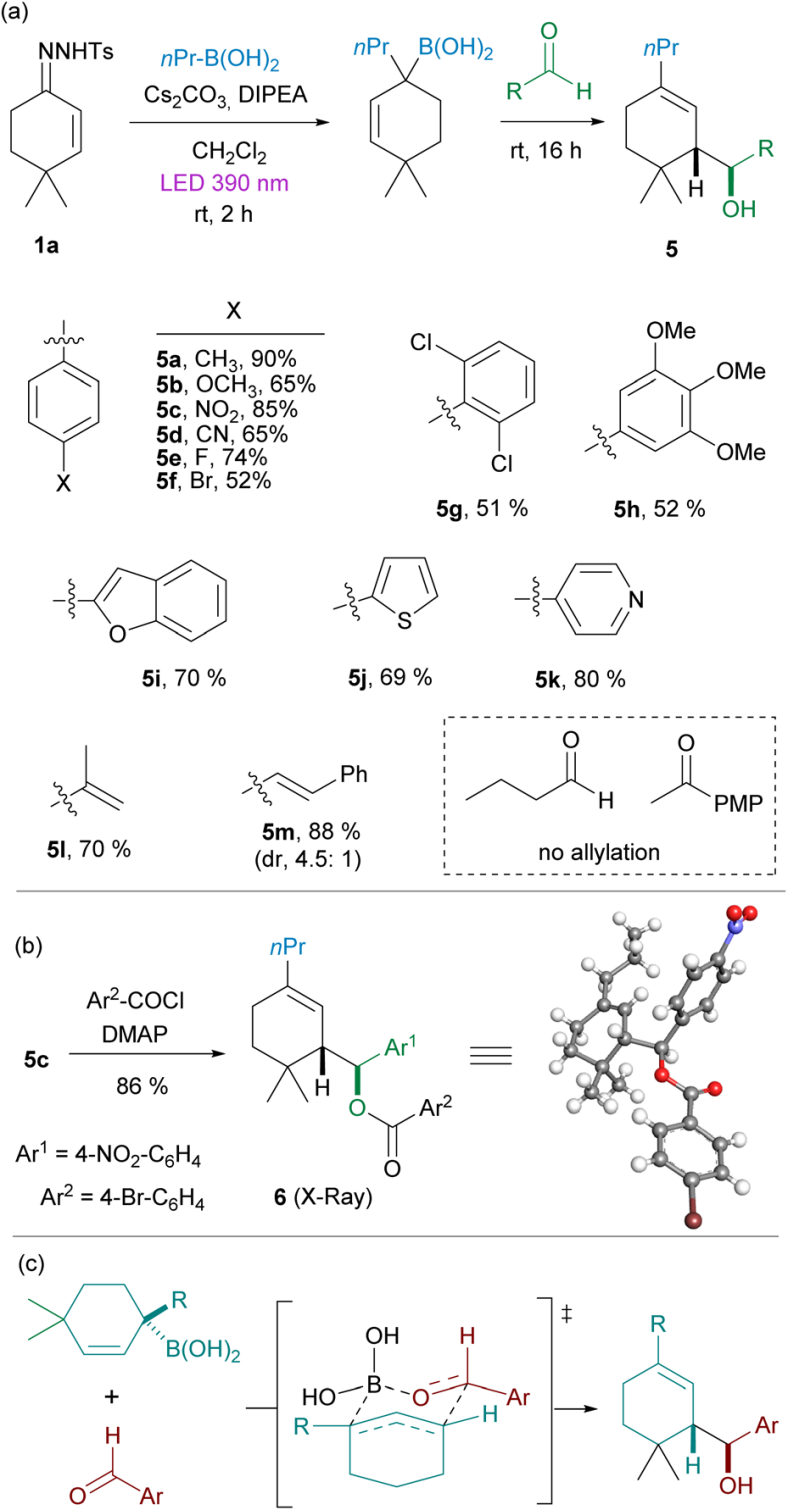
(a) Synthesis of homoallylic alcohols 5 by reactions of *N*-tosylhydrazone 1a with alkylboronic acids under photochemical activation followed by addition of an aldehyde. Standard reaction conditions: hydrazone 1 (0.2 mMol), boronic acid 3 (3 equiv.), Cs_2_CO_3_ (2 equiv.), DIPEA (2 equiv.), CH_2_Cl_2_ (2 mL), 390 nm LED lamp (52 W), rt, 2 h, then addition of the aldehyde (2 equiv.) rt, overnight. Isolated yields for the one-pot process are indicated. (b) Determination of the relative stereochemistry. (c) Mechanistic proposal for the stereochemistry observed based on the classical Zimmerman–Traxler transition state.

The relative stereochemistry of the homoallylic alcohols was determined upon resolution of the X-ray structure of the ester 6, which was obtained by reaction of 5c with 4-bromobenzoyl chloride (Scheme 3(b)) and corresponds to the stereoisomer expected considering that the allylation takes place through a cyclic chair-like Zimmerman–Traxler transition state where the aryl group of the aldehyde occupies an equatorial position (Scheme 3(c)).

The relevance of this transformation should be highlighted. In a very simple three-component reaction, which does not even require the participation of any catalyst, the substitution on the carbonylic and the β positions respectively of an α,β-unsaturated ketone have been carried out *via* the *N*-sulfonylhydrazone. From a synthetic point of view, this is a very rare, and at the same time, a powerful method for the generation of chemical diversity.

**Scheme 4:**
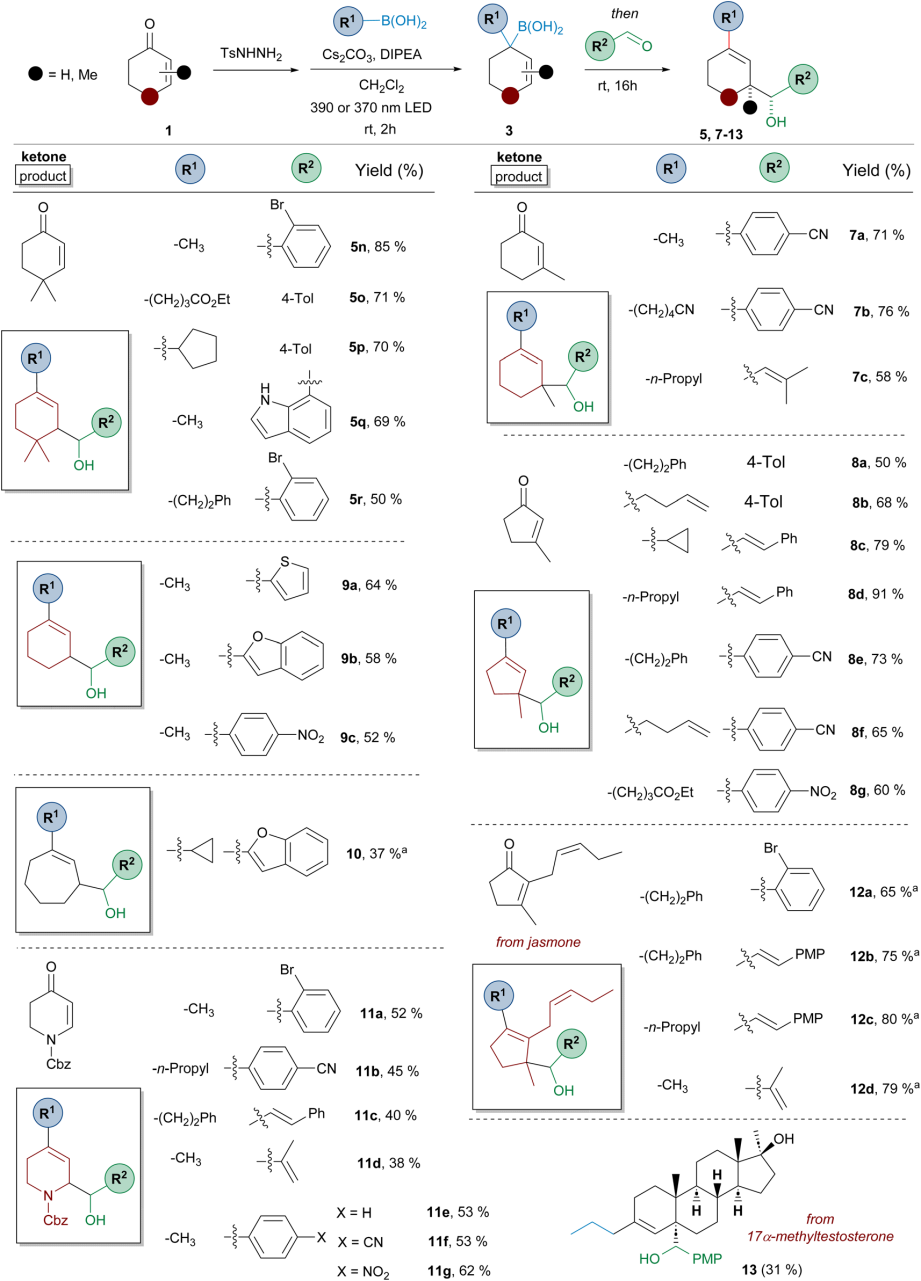
Three-component synthesis of homoallylic alcohols 5–13 by reaction of α,β-unsaturated *N*-tosylhydrazones, boronic acids and aromatic or α,β-unsaturated aldehydes. Standard reaction conditions like in Scheme 3. Isolated yields for the one-pot process are indicated. A 390 nm LED lamp was used unless otherwise indicated. ^*a*^A 370 nm LED lamp was used.

Thus, we set out to investigate the scope of the three-component process regarding the three elements of the reaction: the cycloalkenone, the boronic acid and the aldehyde. The results are presented in Scheme 4.

Regarding the structure of the α,β-unsaturated-*N*-sulfonylhydrazone the scope of the reaction is similar to that described in Scheme 2 for the synthesis of allylic boronates 4. The transformation is compatible with five- (compounds 8 and 12), six- (compounds 5, 7 and 9) and seven- (compound 10) membered ring carbocyclic systems. Moreover, substitution is tolerated at position 3, as represented by the reactions with 3-methylcyclohexenone and 3-methylcyclopentenone (compounds 7 and 8 respectively), in which a new quaternary stereocenter is formed. The reaction proceeded nicely also for a system substituted both at positions 2 and 3 of the double bond, as shown by the examples performed employing the natural jasmone as starting material (compounds 12). In all these cases, an all-carbon quaternary stereocenter is generated in the diastereoselective reaction. The reaction could be also applied to the simplest representatives, the *N*-tosylhydrazones of cyclohexenone and cycloheptenone (compounds 9 and 10 respectively), although the latter delivered the product with lower yield. The three-component sequence could also be applied to *N*-Cbz-2,3-dihydropyridin-4(1*H*)-one to provide substituted dihydropyridine derivatives 11, which are privileged structures for medicinal chemistry.

On the other hand, a structural variety of alkylboronic acids can be incorporated into the three-component transformation, including methyl, *n*-alkyl, cycloalkyl (5p, 8c, 10), as well as functionalized derivatives, as shown by the reaction with boronic acids incorporating a double bond (8b, 8f), ester (5o, 8g) and nitrile (7b) functionalities. Regarding the structure of the aldehyde component, the scope is as wide as that previously shown in Scheme 3, including also the incorporation of other interesting substituents such as *o*-bromophenyl (5r, 11a, 12a) and 7-indolyl (5q).

The three-component reaction was also applied to 17α-methyltestosterone. In this case, the product 13 was obtained as a single diastereoisomer in 31% yield. Although the homologation reaction provides a 1 : 1 mixture of isomers, as indicated in Scheme 2 (4z), only one of the two isomeric allyl boronic acids can undergo the carbonyl allylation for steric reasons, as the approach of the aldehyde through the β-face is hindered by the angular methyl at C10.

Overall, the results presented in Schemes 3 and 4, which include the synthesis of 44 different compounds, reveal the high versatility of this method for the generation of molecular diversity in a very simple way from readily available starting materials. While the set of ketones, aldehydes, and boronic acids in our study may currently be modest, the selection presented clearly demonstrates its potential for expansion into a much larger, structurally diverse library of homoallylic alcohols

**Scheme 5:**
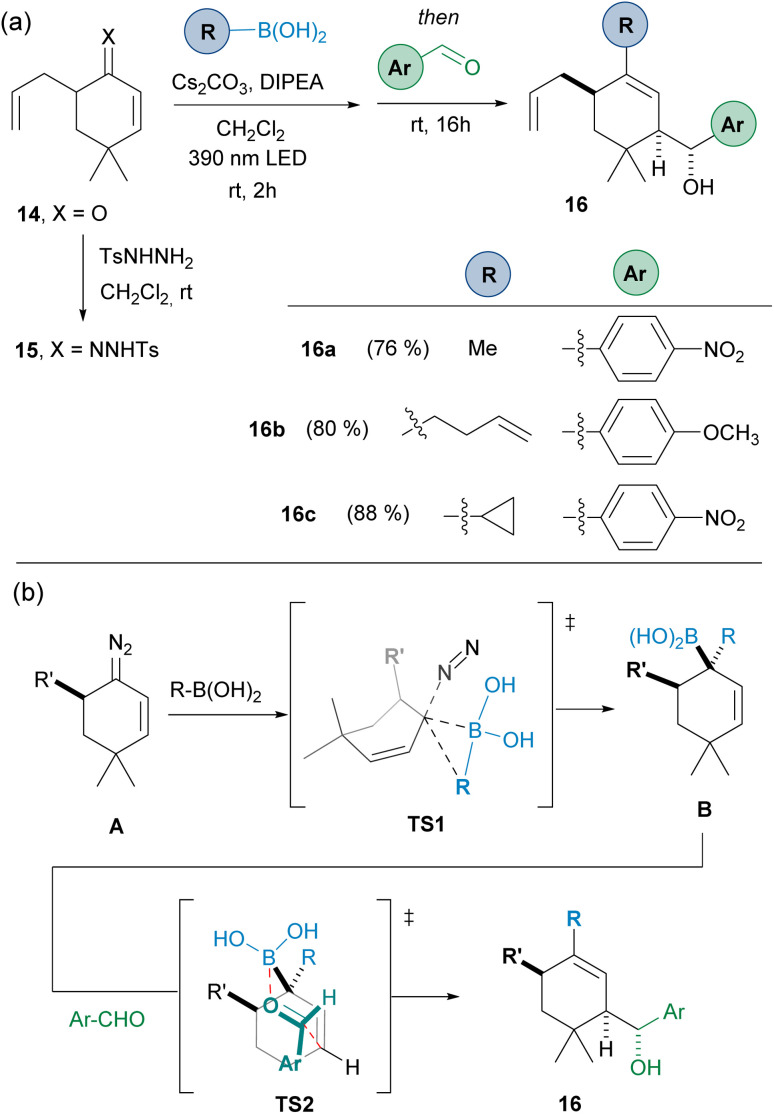
(a) Diastereoselective three-component synthesis of homoallylic alcohols by reaction of the α′-substituted-α,β-*N*-tosylhydrazone 15 with alkyl boronic acids and aromatic aldehydes. (b) Rationale for the diastereoselectivity observed. Standard reaction conditions like in Scheme 3.

To evaluate the possibility of achieving diastereoselective reactions, the 5-substituted cyclohexanone 14 was employed, in the idea that the presence of a substituent at the α-position might control the facial selectivity of the carboborylation, which then would be transferred to the allylation reaction. Thus, starting from ketone 14, condensation with *N*-tosylhydrazide led to *N*-tosylhydrazone 15. Then, the three-component one-pot reaction led to the obtention the homoallylic alcohols 16 as a unique diastereoisomers (Scheme 5(a)).

The stereoselectivity can be explained by taking into consideration that the carboborylation of the diazo compound A takes place through the less hindered face through the transition state TS1 to deliver the allylboronic acid B. Then, the allylation through the TS2 where the Ar group occupies the equatorial position in the chair-like Zimmerman–Traxler transition state defines the stereochemistry observed. This is certainly an important result, as it provides a handle to control the stereoselectivity in the carboborylation-allylation sequence.

**Scheme 6:**
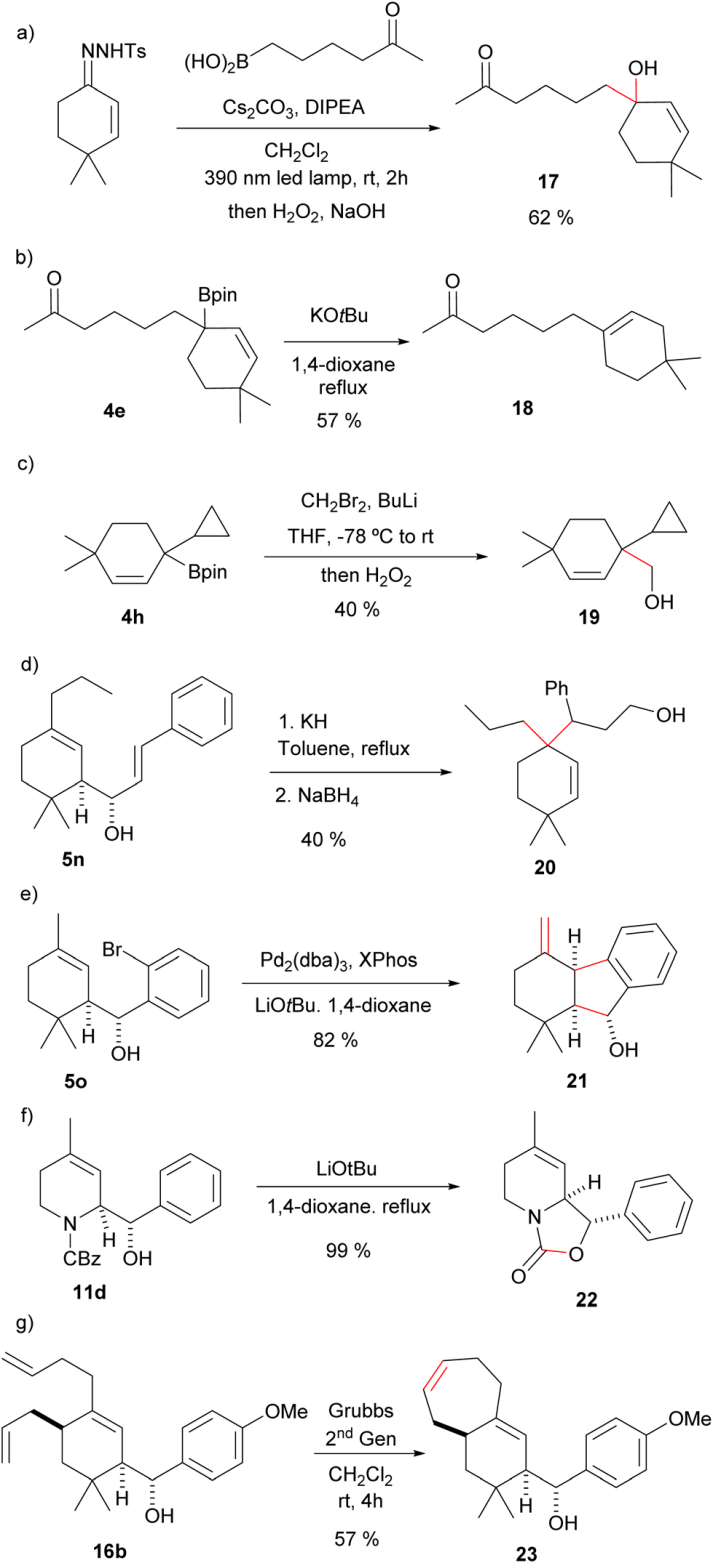
Some synthetic applications of the allylboronic esters and the homoallylic alcohols. (a) Oxidation; (b) protodeboronation; (c) Matteson homologation; (d) Oxy-Cope rearrangement/reduction; (e) intramolecular Heck reaction; (f) oxazolidinone synthesis; (g) ring closing metathesis.

The allylic boronic acids 3 and boronates 4 can participate also in other synthetic transformations. For instance, direct oxidation of the homologated allylboronic acid with H_2_O_2_ gives directly the allylic alcohol 17 (Scheme 6(a)). In the example provided the reaction was performed with the carbonyl containing boronic acid to deliver a hydroxyketone without the need of any protective group. Complementary, the treatment of the allylboronic ester 4e with KO*t*Bu gives the ketoalkene 18, where protodeboronation with migration of the double bond has occurred in a total regioselective manner (Scheme 6(b)). These two transformations would be synthetically very challenging from the α,β-unsaturated carbonyl through an alternative route. Moreover, the latter reaction can be envisioned as a transition-metal-free Csp^3^–Csp^2^ cross coupling process. Additionally, Matteson homologation^18^ of the boronic ester 4h led to the homoallylic alcohol 19 (Scheme 6(c)). In this case two-Csp^3^–Csp^3^ bonds have been formed on the former carbonyl carbon.

The homoallylic alcohols synthesized in the three-component process are also functionalized synthetic intermediates that can be further elaborated. For instance, the compounds derived from the reactions with α,β-unsaturated aldehydes are appropriate substrates for oxy-Cope rearrangements. Indeed, heating 5n in the presence of potassium hydride, followed by reduction with NaBH_4_ the alcohol 20 was obtained as a single stereoisomer (Scheme 6(d)). Interestingly, the overall transformation for the synthetic sequence is again the formation of two Csp^3^–Csp^3^ bonds on the former carbonyl carbon atom of the α,β-unsaturated carbonyl precursor.

On the other hand, intramolecular Heck reaction on the homoallylic alcohol 5o obtained by allylation of *o*-bromobenzaldehyde led to indene derivative 21 (Scheme 6(e)). This is a remarkable transformation indeed, since under a very simple reaction sequence three C–C bonds have been formed on the three consecutive carbon atoms of the α,β-unsaturated carbonyl.

Moreover, treatment of the heterocyclic derivative 11d with LiO*t*Bu in 1,4-dioxane under reflux led to the bicyclo oxazolidinone 22, an interesting core for medicinal chemistry (Scheme 6(f)). As further illustration of the usefulness of the methodology in the generation of structural diversity, ring-closing metathesis on 16b led to the terpenoid-like bicyclic derivative 23 (Scheme 6(g)).

One important factor in generating molecular diversity within drug discovery programs is the ability to produce a wide range of structurally diverse and functionally rich small molecules from a common set of starting materials using a limited set of reactions. The three-component reaction presented here combines several advantageous features: it incorporates a wide array of central cores (*N*-tosylhydrazones), introduces a variety of substituents (boronic acids and aldehydes), features remarkable functional groups tolerance, and allows for further straightforward structural modifications, such as those illustrated in Scheme 6. Thus, this methodology represents a highly appealing tool for Diversity Oriented Synthesis.^19^

## Conclusions

As summary, we have reported in this paper the synthesis of allylic boronates from α,β-unsaturated ketones through the photochemical homologation of alkylboronic acids with the corresponding *N*-tosylhydrazones. The reaction features very wide scope regarding both coupling partners. Additionally, taking advantage of this method for the generation of allylboronic acids, a three-component process has been developed comprising the photochemical homologation followed by an aldehyde allylation.

The overall transformation consists of the double functionalization of the α,β-unsaturated carbonyl on the carbonylic and the β-carbon atom positions with high diastereoselectivity. The versatility of this three-component reaction as a method for the generation of molecular diversity is demonstrated through numerous examples, illustrating its wide applicability with respect to the three partners: the α,β-unsaturated *N*-tosylhydrazone, the boronic acid and the aldehyde.

## Data availability

All data presented in this article are available in the ESI.†

## Author contributions

CV and MP designed the project and supervised the work. AVM and MP carried out the experimental work. All the authors discussed the manuscript. MP and CV wrote the paper.

## Conflicts of interest

There are no conflicts to declare.

## Supplementary Material

SC-015-D4SC06537A-s001

SC-015-D4SC06537A-s002
