# Supplementary material for: Photochemical carboborylation and three-component difunctionalization of α,β-unsaturated ketones with boronic acids via tosylhydrazones
Source: Chem Sci. 2024 Nov 6;15(46):19626–33. doi: 10.1039/d4sc06537a (PMC11575570; doi:10.1039/d4sc06537a)

Supplementary Information (SI) for Chemical Science.
This journal is © The Royal Society of Chemistry 2024

Electronic Supplementary Information for

Photochemical Carboborylation and Three-component Difunctionalization of α,β-unsaturated Ketones with Boronic Acids via Tosylhydrazones

Álvaro Valdés-Maqueda, Manuel Plaza* and Carlos Valdés*

Departamento de Química Orgánica e Inorgánica. Instituto Universitario de Química Organometálica "Enrique Moles" and Centro de Innovación en Química Avanzada (ORFEO-CINQA) Universidad de Oviedo Julián Clavería 8, 33006 Oviedo, Spain.
E-mail: plazamanuel@uniovi.es; acvg@uniovi.es

Table of Contents

1. Experimental Procedures……S2

1.1 General Considerations……S2

1.2 Synthetic procedures for starting materials……S3

1.3 Synthesis and characterization data for *N*-sulfonylhydrazones……S4

1.4 Optimization of the photochemical carboborylation of α,β-unsaturated tosylhydrazones……S5

1.5 Reactions with acyclic α,β-unsaturated tosylhydrazones……S6

1.6 General procedure A: Photochemical synthesis of tertiary allylic pinacolboronates……S8

1.7 General procedure B: Photochemical three-components synthesis of homoallylic alcohols……S8

2. Experimental and characterization data for pinacol boronates 4……S9

3. Experimental and characterization data for alcohols 5, 7, 8, 9, 10, 11, 12, 13, 16 S22

4. Additional synthetic transformations: Experimental procedures and characterization data S40

5. X-Ray Crystallographic data of compound 6……………………………………………S45

6. References………………………………………………………………………………..S47

7. Copies of ^{1}H and ^{13}C NMR spectra………………………………………………..S48

1. Experimental Procedures

1.1 General Considerations

Photochemical reactions in batch were performed in 5 mL glass vials sealed with a septum, under argon atmosphere. The specific reaction time corresponds to the total reaction time. A Kessil® PR160 Rig equipped with different lamps (PR160-370 nm, PR160-390 nm, PR160-427 nm) a cooling fan and a magnetic stirrer was used as the photochemistry setup. A PR time controller was additionally used to select the irradiation time. 5 mL glass vials purchased in VWR® were used to run the photochemical reactions. The vials were sealed with the septum after adding the chemicals and solvent and placed at a distance of 5 cm away from the lamp prior to irradiation at the proper intensity of the Kessil lamp (Figure SI-3). Relevant photophysical properties of the Kessil® PR160L lamps are available at https://kessil.com/products/science_PR160L.php.

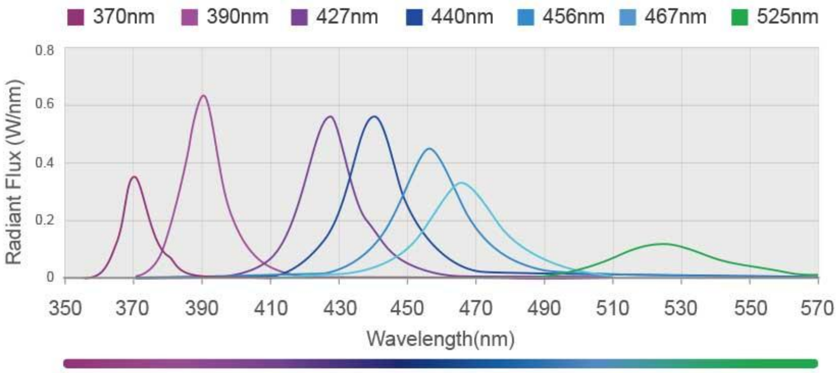

Figure SI-1. Emission spectrums of the different Kessil® lamps.

Power Consumption	370nm (max 43W), 390nm (max 52W), 427nm & 440nm (max 45W), 456nm (max 50W), 467nm (max 44W), 525nm (max 44W)
Input Voltage	100-240 VAC
Operating Temperature	0 - 40°C / 32 - 104°F
Beam Angle	56°
Wavelength Options	370nm, 390nm, 427nm, 440nm, 456nm, 467nm, 525nm
Average Intensity of PR160 series	352mW/cm2 (measured from 1 cm distance)
Dimensions (H x D)	4.49" x 2.48" / 11.4cm x 6.3cm

Figure SI-2. Technical specifications of the Kessil® lamps.

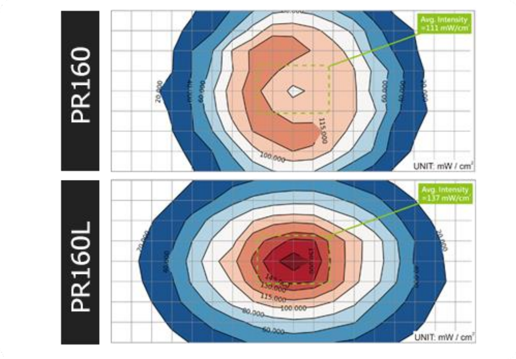

Figure SI-3. Intensity map of the Kessil® lamps.

All the solvents were dried using the corresponding procedures described in D. Perrin, Purification of Laboratory Chemicals, Pergamon Press Ltd. 1980, 2nd Ed.

NMR spectra were recorded in $CDCl_3$ 600, 400, 300 MHz for ^{1}H and 150, 100 or 75 MHz for ^{13}C with the residual solvent signals as standard. The data in the ^{1}H NMR spectra is being reported as s = singlet, bs= broad singlet, d = doublet, dd = double doblet, t = triplet, dt = double triplet, ddt = double double triplet, tt = triple triplet, q = quartet, p = pentuplet, qd = quartet of doublets and so on, m = multiplet or unresolved, chemical shifts in ppm and coupling constant(s) in Hz. The assignment of the ^{13}C NMR spectra have been carried out by means of DEPT-135 experiments. HRMS were measured in ESI mode, with a TOF mass analyser (Bruker model Impact II). The *N*-sulfonylhydrazones employed were prepared from the corresponding carbonyl compounds and *N*-sulfonylhydrazide as described below.

1.2 Synthetic procedures for starting materials

1.2.1 Synthesis of 6-allyl-4,4-dimethylcyclohex-2-en-1-one 14.

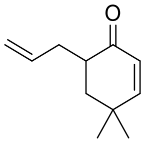

A 100 mL flame-dried Schlenk flask was equipped with a stir bar and a septum, and charged with N_2. Anhydrous THF (40 mL) and diisopropylamine (1.55 mL, 11 mmol, 1.1 equiv) were added. The mixture was cooled to 0 °C, then *n*-BuLi (2.69 M in hexane, 4.46 mL, 12 mmol, 1.2 equiv) was added dropwise via syringe and the resultant solution was stirred at 0 °C for 30 min. Then the solution was cooled to -78 °C and 4,4-dimethylcyclohex-2-en-1-one (1.32 mL, 10 mmol) was added dropwise via syringe. After the solution was stirred for 30 min at the same temperature, DMPU (2.42 mL. 20 mmol, 2 equiv) was added dropwise via syringe and the resultant mixture was stirred for additional 30 min at -78 °C. Then, allyl bromide (2.59 mL, 30 mmol, 3 equiv) was added to the solution and the resulting solution was allowed to warm to -40 °C gradually. After stirring the solution for 13 h, the reaction was quenched with saturated NH_4Cl aq. Then, the solution was extracted with Et_2O. The organic extracts were combined, dried over Na_2SO_4 and concentrated under reduced pressure. The resultant crude product was purified by silica-gel chromatography to give **14** (647.2 mg, 3.94 mmol, 40%) as colorless oil. Spectroscopic data in matches that previously reported in the literature.[1]

^{1}H NMR (401 MHz, $CDCl_3$) δ 6.57 (dd, J = 10.0, 2.2 Hz, 1H), 5.86 – 5.68 (m, 2H), 5.09 – 5.02 (m, 1H), 5.02 – 4.97 (m, 1H), 2.74 – 2.62 (m, 1H), 2.48 (ddt, J = 14.1, 8.6, 4.4 Hz, 1H), 2.13 – 2.00 (m, 1H), 1.84 (ddd, J = 13.4, 4.8, 2.2 Hz, 1H), 1.59 (t, J = 13.7 Hz, 1H), 1.16 (s, 3H), 1.12 (s, 3H).

^{13}C NMR (101 MHz, $CDCl_3$) δ 200.6 (C), 158.9 (CH), 136.3 (CH), 126.8 (CH), 116.7 (CH_2), 42.3 (CH), 41.8 (CH_2), 33.7 (CH_2), 30.7 (CH_3), 25.5 (CH_3).

1.3 Synthesis and characterization data for *N*-sulfonylhydrazones.

The *N*-sulfonylhydrazones **1(a-l) and 15** employed in this work (figure SI-4) were prepared from the corresponding carbonyl compounds following the standard procedure described below unless otherwise indicated. See appropriate references for previously reported *N*-tosylhydrazones: (**1a**)[2]; (**1b**)[3]; (**1c**)[4]; (**1d**)[5]; (**1e**)[6]; (**1g**)[7]; (**1h**)[8]; (**1i, 1k, 1l, 1m**)[9].

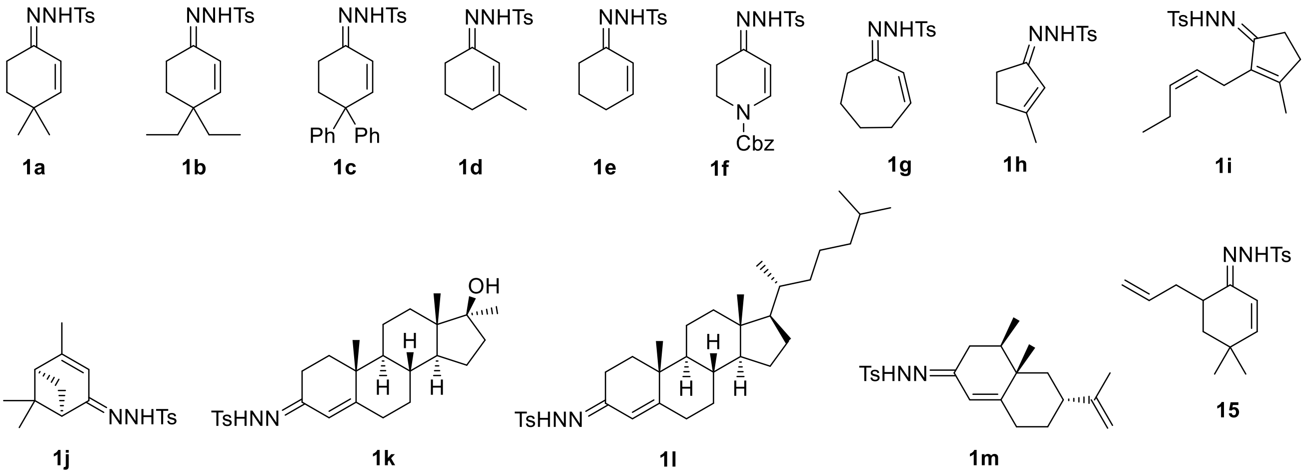

Figure SI-4: *N*-sulfonylhydrazones employed in the paper.

1.3.1 General procedure for the synthesis of *N*-sulfonylhydrazones.

To a stirred solution of the ketone (2 mmol) in 2 mL of MeOH or DCM was added the *N*-sulfonylhydrazide (1.1 equiv). The mixture is stirred overnight at room temperature. The white solid formed is dried under vacuum. The *N*-sulfonylhydrazones can be used without further purification, otherwise can be purified by crystallization of flash chromatography.

1.3.2 Characterization data for new *N*-sulfonylhydrazones.

4-Methyl-N'-((1R,5R,E)-4,6,6-trimethylbicyclo[3.1.1]hept-3-en-2-ylidene)benzenesulfonohydrazide (1j)

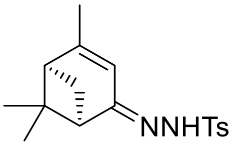

The product is present in the NMR spectra as a 2:1 mixture of rotamers.

^{1}H NMR (300 MHz, $CDCl_3$) δ 7.81 (d, J = 8.0 Hz, 2H), 7.32 – 7.14 (m, 2H), 6.07 (s, 1H), 5.78 (s, *rot. min.*), 3.45 (s, 3H + *rot. min.*), 3.10 (t, J = 5.3 Hz, *rot. min.*), 2.69 (t, J = 5.6 Hz, *rot. min.*), 2.62 – 2.51 (m, 1 H + *rot. min.*), 2.39 (s, 3H + *rot. min.*), 2.25 – 2.13 (m, 1H +

rot. min.), 1.88 (s, 3H), 1.81 (s, *rot. min.*), 1.62 (d, J = 9.0 Hz, 1H), 1.58 (d, J = 9.0 Hz, *rot. min.*), 1.37 (s, *rot. min.*), 1.35 (s, 3H), 0.71 (s, 3H), 0.70 (s, *rot. min.*).

^{13}C NMR (75 MHz, $CDCl_3$) δ 163.4 (C), 163.0 (C), 161.8 (C), 156.5 (C), 143.8 (CH), 143.7 (CH), 135.5 (CH), 135.4 (CH), 129.5 (CH), 129.4 (CH), 127.9 (CH), 118.5 (C), 109.2 (CH), 50.9 (CH_2), 50.7 (CH_2), 49.4 (CH), 49.3 (CH), 49.0 (C), 48.9 (C), 43.2 (CH), 38.0 (CH), 37.0 (CH), 26.0 (CH_3), 23.9 (CH_3), 23.0 (CH_3), 21.9 (CH_3), 21.5 (CH_3), 21.5 (CH_3).

HRMS [ESI(+)]: m/z calcd. for $C_{17}H_{23}N_2O_2S$: 319.1475 [M+H], found: 319.1469.

(*E*)-*N*'-(6-Allyl-4,4-dimethylcyclohex-2-en-1-ylidene)-4-methylbenzenesulfonohydrazide (15)

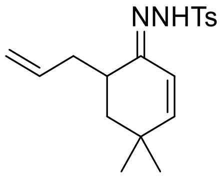

^{1}H NMR (300 MHz, $CDCl_3$) δ 8.08 (s, 1H), 7.84 (d, J = 8.3 Hz, 2H), 7.27 (d, J = 8.3 Hz, 2H), 6.14 (dd, J = 10.2, 1.5 Hz, 1H), 6.01 (dd, J = 10.2, 1.7 Hz, 1H), 5.63 (ddt, J = 17.2, 10.0, 7.1 Hz, 1H), 4.99 – 4.86 (m, 2H), 2.59 (ddd, J = 14.0, 6.2, 4.4 Hz, 1H), 2.51 – 2.41 (m, 1H), 2.41 (s, 3H), 1.99 (dt, J = 14.7, 7.7 Hz, 1H), 1.62 (ddd, J = 13.3, 3.9, 1.7 Hz, 1H), 1.28 – 1.16 (m, 1H), 0.99 (s, 3H), 0.97 (s, 3H).

^{13}C NMR (75 MHz, $CDCl_3$) δ 154.6 (C), 151.4 (CH), 143.9 (C), 136.9 (C), 135. (CH), 129.4 (CH), 128. (CH), 116.2 (CH_2), 113.9 (C), 41.7 (CH_2), 36.2 (CH), 34.9 (CH), 33.6 (CH_2), 30.4 (CH_3), 26.4 (CH_3), 21.7 (CH_3).

HRMS [EI(+)]: m/z calcd. for $C_{18}H_{24}N_2O_2S$: 332.1553 [M+], found: 332.1547.

1.4 Optimization of the photochemical carboborylation of α,β-unsaturated tosylhydrazones

To find proper reaction conditions for the photochemical carboborylation of α,β-unsaturated tosylhydrazones a model reaction between tosylhydrazone **1a** and propylboronic acid was studied following the experimental procedure below. The influence of different parameters is displayed in the Table S1.

A 5 mL glass vial provided with a stirring bar was charged with the corresponding tosylhydrazone **1a** (0.2 mmol), *n*-propylboronic acid (3 equiv) and the solid base (0.4 mmol). The vial was sealed with a septum and evacuated under vacuum and filled with argon. Then, degassed solvent (2 mL) or a solution of the liquid bases if applicable (DIPEA, DBU) (0.4 mmol) in dry and degassed solvet (2 mL) was added to the vial through the septum with the aid of a needle. The vial was placed in the Kessil® PR160 Rig in front of the Kessil® PR160L lamp (390 nm) at a distance of 5 cm. The cooling fan and the light were turned on keeping vigorous stirring at room temperature. After 2 h the light and the cooling fan were turned off, the septum was removed and 5 equiv of pinacol were added. The vial was sealed again and the reaction was stirred for 16 h at rt. Then, the vial was opened and the reaction was quenched with 5 mL of water and 5 mL of CH_2Cl_2. The layers were separated and the aqueous phase was extracted with CH_2Cl_2 (2x5

mL). The combined organic layers were washed with brine (2x5 mL), dried over Na_2SO_4, and concentrated under reduced pressure. The residue was dried under vacuum. The resulting reaction crude was purified by column chromatography.

Table SI-1. Influence of the reaction conditions in the homologation of propylboronic acid with tosylhydrazone **1a**.[a]

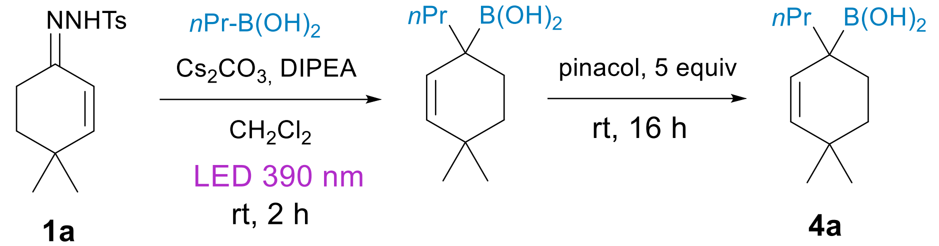

Entry	Deviation from standard conditions	Yield %[b]
**1**	**none**	**70**
2	No DIPEA added	36
3	K_2CO_3 instead of Cs_2CO_3	43
4	DBU instead of Cs_2CO_3	40
5	No light	0
6	No base	0
7	Toluene instead of DCM	26
8	THF instead of DCM	6

[a] Standard conditions: hydrazone **1a** 0.2 mmol, *n*-propyl-B(OH)$_2$ (3 equiv), solvent 2 mL, Kessil PR160L lamp 390 nm (52 W). Isolated yield after column chromatography. DIPEA: Diisopropylethylamine. DBU: 1,8-Diazabicyclo(5.4.0)undec-7-ene

1.5 Reactions with acyclic α,β-unsaturated tosylhydrazones

The application of the photochemical carboborylation to acyclic α,β-unsaturated tosylhydrazones turned out to be unefficient under the reaction conditions. When the reaction was attempted with the tosylhydrazone **24** derived from of benzylidenacetone, it afforded 3-methyl-5-phenylpyrazole **25** as main product, which is formed upon cyclization of the diazoalkane.[10] This reaction is faster than the expected carboborylation. Additionally, no pinacolboronate was detected with the tosylhydrazone of cinnamaldehyde **26** either, leading, in this case, to a complex mixture of products coming from the degradation of the diazocompound, with no traces of the alkylboronic acid being incorporated (scheme SI-1, a).

We examined also the reaction with the β-alkyl-substituted tosylhydrazone of hept-3-en-2-one **27** as an additional model for a linear α,β-unsaturated carbonyl. Under the standard conditions, in the reaction with cyclopropylboronic acid, after the addition of pinacol, a complex reaction mixture was obtained. Nevertheless, the pinacol boronic ester **28** could be isolated albeit in a

very poor 18 % yield after column chromatography. A similar result was obtained when methylboronic acid was employed (scheme SI-1, b).

Considering that the allylation of an aldehyde is a more efficient way to trap the intermediate allylboronic acid than the reaction with pinacol, the three-component sequential reaction was also tested combining the tosylhydrazone **27**, methylboronic acid and 4-methoxybenzaldehyde. This time a mixture of the isomeric homoallylic alcohols **30** and **31** (1: 0.6 ratio) was obtained a 51 % combined yield after chromatographic purification (figure SI-5)(scheme SI-1, c).

This latter result indicates that the carboborylation of the linear α,β-unsaturated tosylhydrazone **27** does indeed take place under the photochemical conditions to deliver initially the expected allylboronic acid **32**. However, in our hands, their relative unstability prevented the isolation of the pinacolboronates in useful yield. Additionally, under the reaction conditions, equilibration via a 1,3-borotropic rearrangement[11] occurs to produce a mixture of regioisomeric allylboronic acids **32** and **33**. The subsequent reaction of each isomer with the aromatic aldehyde furnishes the mixture of homoallylic alcohols **30** and **31** (scheme SI 1, d).

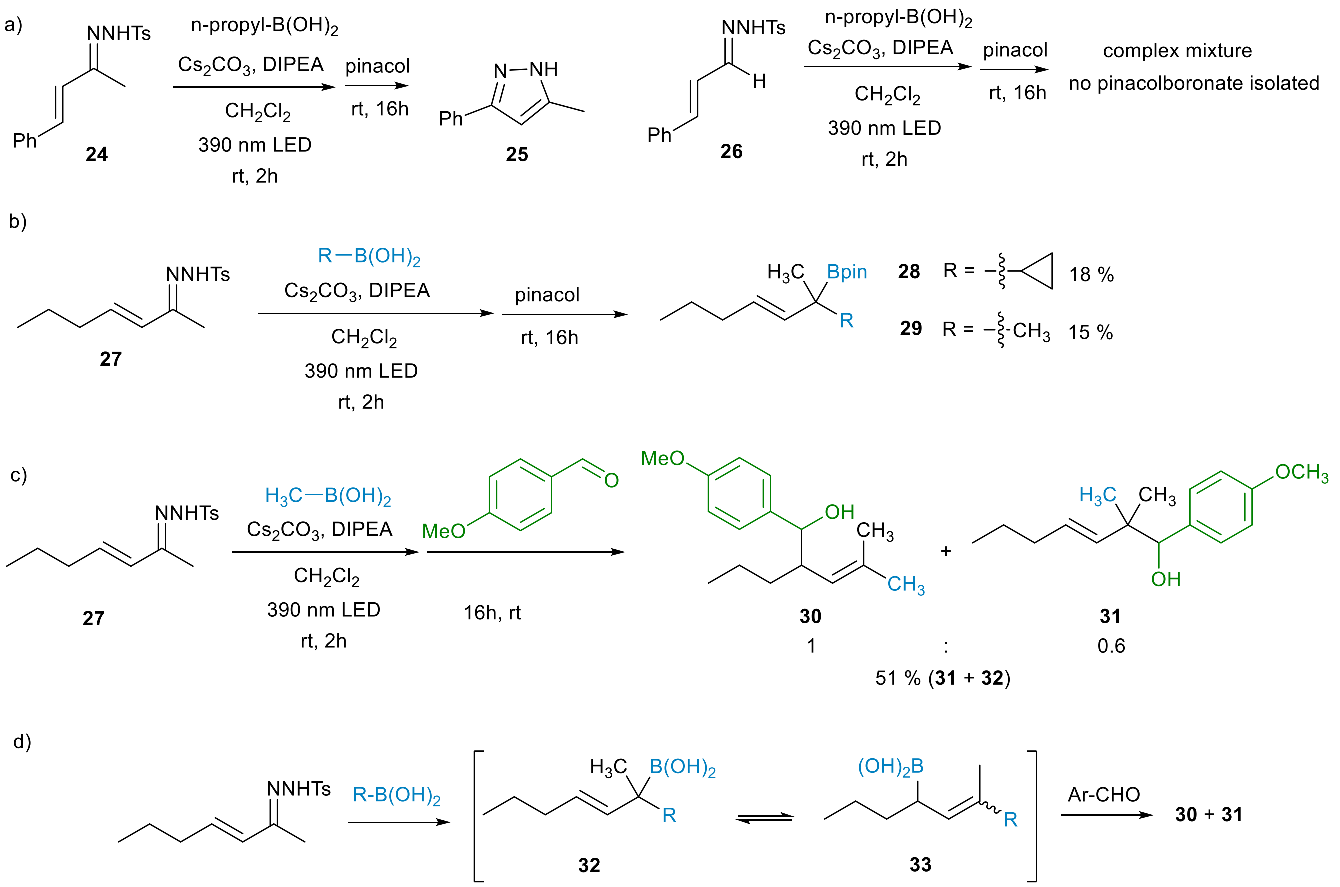

Scheme SI-1. Reactions with acyclic of α,β-unsaturated *N*-tosylhydrazones. a) Unsuccessful reactions with β-aryl-α,β-unsaturated *N*-tosylhydrazones **24** and **26**. b) Carboborylation of a β-alkyl-α,β-unsaturated tosylhydrazone **27**. c) Three component reaction with tosylhydrazone **27**. d) Justification of the formation of regioisomers based on a 1,3-borotropic rearrangement.

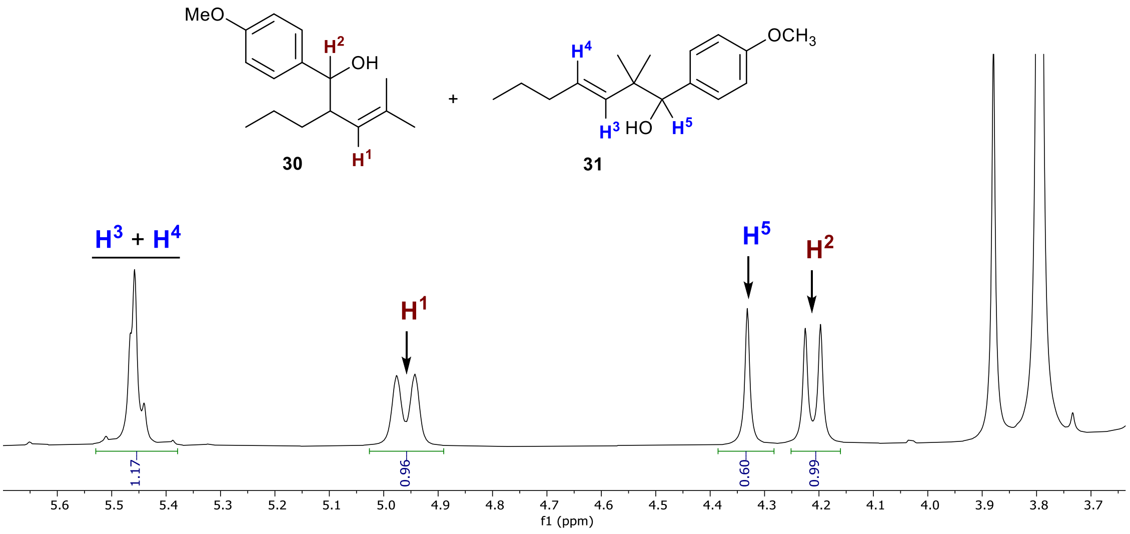

Figure SI-5: Expansion of the 3.6-5.7 ppm region of the ^{1}H NMR ($CDCl_3$, 300 MHz) spectrum of the crude residue of the reaction between the tosylhydrazone **27**, methylboronic acid and 4-methoxybenzaldehyde. The signals corresponding to the alkenyl and C**H**-OH protons can be identified for both regioisomeric homoallylic alcohols **30** and **31** in a 1: 0.6 ratio.

1.6 General procedure A: Photochemical synthesis of tertiary allylic pinacolboronates

A 5 mL glass vial provided with a stirring bar was charged with the corresponding *N*-sulfonylhydrazone (0.2 mmol) the boronic acid (3 equiv) and Cs_2CO_3 (0.4 mmol, 131 mg). The vial was sealed with a septum and evacuated under vacuum and filled with argon. Then a solution of DIPEA (0.4 mmol) in dry and degassed CH_2Cl_2 (2 mL) was added to the vial through the septum with the aid of a needle. The vial was placed in the Kessil® PR160 Rig in front of the Kessil® PR160L lamp (370 or 390 nm) at a distance of 5 cm. The cooling fan and the light were turned on keeping vigorous stirring at room temperature. After the time indicated (2 h) the light and the cooling fan were turned off, the septum was removed and 5 equiv of pinacol were added. The vial was sealed again and the reaction was stirred for 16 h at rt. Then, the vial was opened and the reaction was quenched with 5 mL of water and 5 mL of CH_2Cl_2. The layers were separated and the aqueous phase was extracted with CH_2Cl_2 (2x5 mL). The combined organic layers were washed with brine (2x5 mL), dried over Na_2SO_4, and concentrated under reduced pressure. The residue was dried under vacuum. The resulting reaction crude was purified by column chromatography.

1.7 General procedure B: Photochemical three-components synthesis of homoallylic alcohols

A 5 mL glass vial provided with a stirring bar was charged with the corresponding *N*-sulfonylhydrazone (0.2 mmol) the boronic acid (3 equiv) and Cs_2CO_3 (0.4 mmol, 131 mg). The vial was sealed with a septum and evacuated under vacuum and filled with argon. Then a solution of DIPEA (0.4 mmol) in dry and degassed CH_2Cl_2 (2 mL) was added to the vial through the septum with the aid of a needle. The vial was placed in the Kessil® PR160 Rig in front of the Kessil® PR160L lamp (370 or 390 nm) at a distance of 5 cm. The cooling fan and the light were turned on keeping vigorous stirring at room temperature. After

the time indicated (2 h) the light and the cooling fan were turned off, the septum was removed and 2 equiv of the corresponding aldehyde were added. The vial was sealed again and the reaction was stirred for 16 h at rt. Then, the vial was opened and the reaction was quenched with 5 mL of water and 5 mL of CH_2Cl_2. The layers were separated and the aqueous phase was extracted with CH_2Cl_2 (2x5 mL). The combined organic layers were washed with brine (2x5 mL), dried over Na_2SO_4 and concentrated under reduced pressure. The residue was dried under vacuum. In order to remove the excess of aldehyde, the residue was dissolved in 5 mL of MeOH (if an aromatic aldehyde was used) or DMF (if an aliphatic aldehyde was used). Then, 5 mL of saturated aqueous sodium bisulfite were added and the mixture was shake vigorously for approximately 30 seconds.[12] After that, 10 mL of water and 10 mL of EtOAc were added and the layers were separated. The aqueous phase was extracted with EtOAc (2x5 mL). The combined organic layers were washed with brine (2x5 mL), dried over Na_2SO_4 and concentrated under reduced pressure. The residue was dried under vacuum. The resulting reaction crude was pure and if not, it was purified by column chromatography.

2. Experimental and characterization data for pinacol boronates 4.

2-(4,4-Dimethyl-1-propylcyclohex-2-en-1-yl)-4,4,5,5-tetramethyl-1,3,2-dioxaborolane (4a)

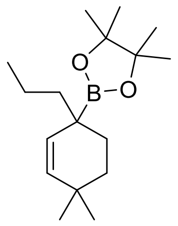

(390 nm lamp) 58.5 mg of *N*-tosylhydrazone **1a** and 53 mg of propylboronic acid afforded 38 mg of **4a** (70 % yield) as a colourless oil.

^{1}H NMR (300 MHz, $CDCl_3$) δ 5.44 (d, *J* = 10.2 Hz, 1H), 5.35 (d, *J* = 10.0 Hz, 1H), 1.85 – 1.77 (m, 1H), 1.47 – 1.24 (m, 7H), 1.21 (d, *J* = 3.0 Hz, 12H), 0.97 – 0.91 (m, 6H), 0.87 (t, *J* = 6.7 Hz, 3H).

^{13}C NMR (75 MHz, $CDCl_3$) δ 137.2 (CH), 131.2 (CH), 83.9 (C), 41.6 (CH_2), 36.3 (CH_2), 31.7 (C), 31.1 (CH_3), 29.5 (CH_3), 28.1 (CH_2), 24.9 (CH_3), 24.6 (CH_3), 19.3 (CH_2), 15.1 (CH_3).

^{11}B NMR (129 MHz, $CDCl_3$) δ 33.88.

HRMS [APCI(+)]: m/z calcd. for $C_{17}H_{32}BO_2$: 279.2490 [M+H], found: 279.2499.

4,4,5,5-Tetramethyl-2-(1,4,4-trimethylcyclohex-2-en-1-yl)-1,3,2-dioxaborolane (4b)

(390 nm lamp) 58.5 mg of *N*-tosylhydrazone **1a** and 36 mg of methylboronic acid afforded 37 mg of **4b** (75 % yield) as a colourless oil.

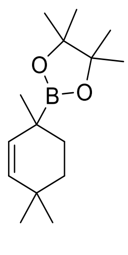

^{1}H NMR (300 MHz, $CDCl_3$) δ 5.44 (d, *J* = 9.9 Hz, 1H), 5.35 (d, *J* = 9.9 Hz, 1H), 1.91 – 1.76 (m, 1H), 1.70 – 1.27 (m, 3H), 1.22 (d, *J* = 1.8 Hz, 12H), 1.01 (s, 3H), 0.97 – 0.93 (m, 6H).

^{13}C NMR (75 MHz, $CDCl_3$) δ 135.5 (CH), 132.2 (CH), 83.2 (C), 36.2 (CH_2), 31.5 (C), 30.9 (CH_3), 30.5 (CH_2), 29.7 (CH_3), 24.8 (CH_3), 24.6 (CH_3).

^{11}B NMR (129 MHz, $CDCl_3$) δ 33.83.

HRMS [APCI(+)]: m/z calcd. for $C_{15}H_{28}BO_2$: 251.2177 [M+H], found: 251.2183.

2-(4,4-Dimethyl-1-phenethylcyclohex-2-en-1-yl)-4,4,5,5-tetramethyl-1,3,2-dioxaborolane (4c)

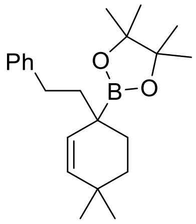

(390 nm lamp) 58.5 mg of *N*-tosylhydrazone **1a** and 90 mg of phenethylboronic acid afforded 53 mg of **4c** (78 % yield) as a colourless oil after column chromatography on SiO_2. Rf = 0.45 (Hex/EtOAc 20:1).

^{1}H NMR (300 MHz, $CDCl_3$) δ 7.33 – 7.23 (m, 2H), 7.23 – 7.13 (m, 3H), 5.54 (d, *J* = 10.0 Hz, 1H), 5.44 (d, *J* = 10.0 Hz, 1H), 2.67 – 2.55 (m, 2H), 1.98 – 1.87 (m, 1H), 1.85 – 1.71 (m, 1H), 1.70 – 1.56 (m, 1H), 1.56 – 1.42 (m, 3H), 1.27 (d, *J* = 2.4 Hz, 12H), 1.02 – 0.92 (m, 6H).

^{13}C NMR (75 MHz, $CDCl_3$) δ 143.5 (C), 136.4 (CH), 130.6 (CH), 128.5 (CH), 128.4 (CH), 125.7 (CH), 83.3 (C), 41.2 (CH_2), 36.2 (CH_2), 32.6 (CH_2), 31.7 (C), 31.0 (CH_3), 29.5 (CH_3), 27.8 (CH_2), 25.0 (CH_3), 24.7 (CH_3).

^{11}B NMR (129 MHz, $CDCl_3$) δ 34.11.

HRMS [ESI(+)]: m/z calcd. for $C_{22}H_{34}BO_2$: 341.2646 [M+H], found: 341.2656.

2-(1-(3-Bromopropyl)-4,4-dimethylcyclohex-2-en-1-yl)-4,4,5,5-tetramethyl-1,3,2-dioxaborolane (4d)

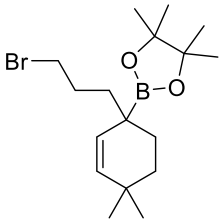

(390 nm lamp) 58.5 mg of *N*-tosylhydrazone **1a** and 100.1 mg of 3-bromopropylboronic acid afforded 51 mg of **4d** (51 % yield) as a colourless oil after column chromatography on SiO_2. Rf = 0.29 (Hex/EtOAc 20:1).

^{1}H NMR (300 MHz, $CDCl_3$) δ 5.40 (s, 2H), 3.37 (t, *J* = 6.9 Hz, 2H), 1.93 – 1.76 (m, 3H), 1.60 – 1.34 (m, 5H), 1.22 (d, *J* = 2.7 Hz, 12H), 0.95 – 0.92 (m, 6H).

^{13}C NMR (75 MHz, $CDCl_3$) δ 136.6 (CH), 130.3 (CH), 83.4 (C), 37.4 (CH_2), 36.1 (CH_2), 34.6 (CH_2), 31.6 (C), 31.0 (CH_3), 29.7 (CH_2), 29.4 (CH_3), 27.8 (CH_2), 24.9 (CH_3), 24.6 (CH_3).

^{11}B NMR (129 MHz, $CDCl_3$) δ 33.82.

HRMS [ESI(+)]: m/z calcd. for $C_{17}H_{31}BBrO_2$: 357.1595 [M+H], found: 357.1598.

6-(4,4-Dimethyl-1-(4,4,5,5-tetramethyl-1,3,2-dioxaborolan-2-yl)cyclohex-2-en-1-yl)hexan-2-one (4e)

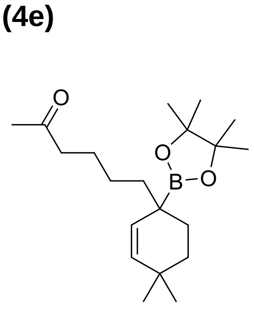

(390 nm lamp) 58.5 mg of *N*-tosylhydrazone **1a** and 86.4 mg of (5-oxohexyl)boronic acid afforded 46.6 mg of **4e** (70 % yield) as a colourless oil after column chromatography on SiO_2. Rf = 0.4 (Hex/EtOAc 5:1).

^{1}H NMR (300 MHz, $CDCl_3$) δ 5.42 (d, *J* = 10.1 Hz, 1H), 5.35 (d, *J* = 10.1 Hz, 1H), 2.40 (t, *J* = 7.5 Hz, 2H), 2.11 (s, 3H), 1.84 – 1.74 (m, 1H), 1.60 – 1.23 (m, 9H), 1.20 (d, *J* = 3.1 Hz, 12H), 0.95 – 0.89 (m, 6H).

^{13}C NMR (75 MHz, $CDCl_3$) δ 209.4 (C), 136.1 (CH), 130.9 (CH), 83.2 (C), 43.8 (CH_2), 38.8 (CH_2), 36.2 (CH_2), 31.6 (C), 31.0 (CH_3), 30.0 (CH_3), 29.5 (CH_3), 27.9 (CH_2), 25.6 (CH_2), 24.9 (CH_3), 24.7 (CH_2), 24.6 (CH_3).

^{11}B NMR (129 MHz, $CDCl_3$) δ 33.45.

HRMS [APCI(+)]: m/z calcd. for $C_{20}H_{36}BO_3$: 335.2752 [M+H], found: 335.2761.

4-(4,4-Dimethyl-1-(4,4,5,5-tetramethyl-1,3,2-dioxaborolan-2-yl)cyclohex-2-en-1-yl)butanenitrile (4f)

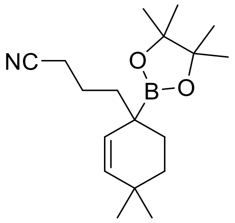

(390 nm lamp) 58.5 mg of *N*-tosylhydrazone **1a** and 67.8 mg of (3-cyanopropyl)boronic acid afforded 32.8 mg of **4f** (55 % yield) as a white solid after column chromatography on SiO_2. Rf = 0.28 (Hex/EtOAc 8:1).

^1H NMR (300 MHz, $CDCl_3$) δ 5.42 (d, *J* = 10.2 Hz, 1H), 5.38 (d, *J* = 10.2 Hz, 1H), 2.31 (t, *J* = 7.0 Hz, 2H), 1.85 – 1.75 (m, 1H), 1.73 – 1.25 (m, 8H), 1.22 (d, *J* = 2.5 Hz, 12H), 0.94 (s, 3H), 0.93 (s, 3H).

^{13}C NMR (75 MHz, $CDCl_3$) δ 137.0 (CH), 129.8 (CH), 119.9 (C), 83.4 (C), 37.9 (CH_2), 35.9 (CH_2), 31.6 (C), 30.9 (CH_3), 29.4 (CH_3), 27.6 (CH_2), 24.9 (CH_3), 24.6 (CH_3), 22.2 (CH_2), 18.0 (CH_2).

^{11}B NMR (129 MHz, $CDCl_3$) δ 33.82.

HRMS [APCI(+)]: m/z calcd. for $C_{18}H_{31}BNO_2$: 304.2442 [M+H], found: 304.2451.

m.p. = 49 – 51 ºC

2-(1-(But-3-en-1-yl)-4,4-dimethylcyclohex-2-en-1-yl)-4,4,5,5-tetramethyl-1,3,2-dioxaborolane (4g)

(390 nm lamp) 58.5 mg of *N*-tosylhydrazone **1a** and 60 mg of 3-butenylboronic acid afforded 38.1 mg of **4g** (66 % yield) as a colourless oil after column chromatography on SiO_2. Rf = 0.39 (Hex/EtOAc 20:1).

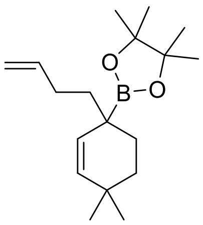

^1H NMR (300 MHz, $CDCl_3$) δ 5.81 (ddt, *J* = 16.9, 10.1, 6.6 Hz, 1H), 5.45 (d, *J* = 9.9 Hz, 1H), 5.38 (d, *J* = 9.9 Hz, 1H), 5.04 – 4.85 (m, 2H), 2.11 – 1.99 (m, 2H), 1.87 – 1.77 (m, 1H), 1.60 – 1.27 (m, 5H), 1.22 (d, *J* = 2.9 Hz, 12H), 0.96 – 0.92 (m, 6H).

^{13}C NMR (75 MHz, $CDCl_3$) δ 139.6 (CH), 136.3 (CH), 130.7 (CH), 114.1 (CH_2), 83.2 (C), 38.3 (CH_2), 36.2 (CH_2), 31.6 (C), 31.0 (CH_3), 30.5 (CH_2), 29.4 (CH_3), 27.8 (CH_2), 24.9 (CH_3), 24.6 (CH_3).

^{11}B NMR (129 MHz, $CDCl_3$) δ 33.85.

HRMS [ESI(+)]: m/z calcd. for $C_{18}H_{32}BO_2$: 291.2490 [M+H], found: 291.2498.

2-(1-Cyclopropyl-4,4-dimethylcyclohex-2-en-1-yl)-4,4,5,5-tetramethyl-1,3,2-dioxaborolane (4h)

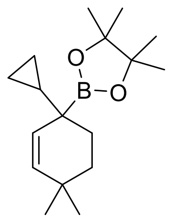

(390 nm lamp) 58.5 mg of *N*-tosylhydrazone **1a** and 51.6 mg of cyclopropylboronic acid afforded 40 mg of **4h** (73 % yield) as a colourless oil after column chromatography on SiO_2. Rf = 0.38 (Hex/EtOAc 20:1).

^{1}H NMR (300 MHz, $CDCl_3$) δ 5.40 (d, *J* = 10.1 Hz, 1H), 5.35 (d, *J* = 10.1 Hz, 1H), 1.89 – 1.77 (m, 1H), 1.57 – 1.27 (m, 3H), 1.21 (d, *J* = 1.6 Hz, 12H), 0.94 (s, 3H), 0.92 (s, 3H), 0.72 (tt, *J* = 9.0, 5.8 Hz, 1H), 0.32 – 0.24 (m, 4H).

^{13}C NMR (75 MHz, $CDCl_3$) δ 136.8 (CH), 128.6 (CH), 83.2 (C), 36.2 (CH_2), 31.7 (C), 30.9 (CH_3), 29.6 (CH_3), 28.6 (CH_2), 24.9 (CH_3), 24.6 (CH_3), 18.5 (CH), 1.1 (CH_2), 0.7 (CH_2).

^{11}B NMR (129 MHz, $CDCl_3$) δ 33.31

HRMS [ESI(+)]: m/z calcd. for $C_{17}H_{30}BO_2$: 277.2333 [M+H], found: 277.2342.

2-(1-Cyclobutyl-4,4-dimethylcyclohex-2-en-1-yl)-4,4,5,5-tetramethyl-1,3,2-dioxaborolane (4i)

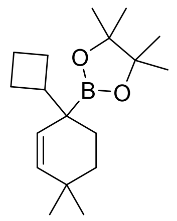

(390 nm lamp) 58.5 mg of *N*-tosylhydrazone **1a** and 60 mg of cyclobutylboronic acid afforded 24.6 mg of **4i** (43 % yield) as a colourless oil after column chromatography on SiO_2. Rf = 0.47 (Hex/EtOAc 20:1).

^{1}H NMR (300 MHz, $CDCl_3$) δ 5.55 (d, *J* = 10.1 Hz, 1H), 5.44 (d, *J* = 10.1 Hz, 1H), 2.47 – 2.30 (m, 1H), 2.00 – 1.76 (m, 5H), 1.76 – 1.63 (m, 2H), 1.52 – 1.39 (m, 1H), 1.39 – 1.27 (m, 2H), 1.22 (d, *J* = 1.9 Hz, 12H), 0.93 (s, 3H), 0.93 (s, 3H).

^{13}C NMR (75 MHz, $CDCl_3$) δ 136.6 (CH), 128.3 (CH), 83.2 (C), 42.5 (CH), 36.6 (CH_2), 31.8 (C), 31.2 (CH_3), 29.3 (CH_3), 25.9 (CH_2), 24.9 (CH_3), 24.7 (CH_3), 24.4 (CH_2), 24.1 (CH_2), 18.1 (CH_2).

^{11}B NMR (129 MHz, $CDCl_3$) δ 33.63.

HRMS [ESI(+)]: m/z calcd. for $C_{18}H_{32}BO_2$: 291.2490 [M+H], found: 291.2498.

2-(1-Cyclopentyl-4,4-dimethylcyclohex-2-en-1-yl)-4,4,5,5-tetramethyl-1,3,2-dioxaborolane (4j)

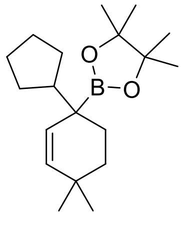

(390 nm lamp) 58.5 mg of *N*-tosylhydrazone **1a** and 68.4 mg of cyclopentylboronic acid afforded 18 mg of **4j** (30 % yield) as a colourless oil after column chromatography on SiO_2. Rf = 0.3 (Hex/EtOAc 20:1).

^{1}H NMR (300 MHz, $CDCl_3$) δ 5.49 (d, *J* = 10.0 Hz, 1H), 5.41 (d, *J* = 10.0 Hz, 1H), 2.01 – 1.84 (m, 1H), 1.82 – 1.72 (m, 1H), 1.70 – 1.26 (m, 11H), 1.22 (d, *J* = 1.6 Hz, 12H), 0.97 – 0.89 (m, 6H).

^{13}C NMR (75 MHz, $CDCl_3$) δ 136.2 (CH), 130.0 (CH), 83.1 (C), 46.5 (CH), 36.9 (CH_2), 31.8 (C), 31.4 (CH_3), 29.2 (CH_3), 28.4 (CH_2), 28.1 (CH_2), 26.8 (CH_2), 26.1 (CH_2), 26.1 (CH_2), 24.9 (CH_3), 24.7 (CH_3).

^{11}B NMR (129 MHz, $CDCl_3$) δ 33.94.

HRMS [APCI(+)]: m/z calcd. for $C_{19}H_{34}BO_2$: 305.2646 [M+H], found: 305.2658.

2-(4,4-Dimethyl-[1,1'-bi(cyclohexan)]-2-en-1-yl)-4,4,5,5-tetramethyl-1,3,2-dioxaborolane (4k)

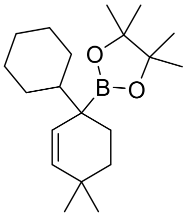

(390 nm lamp) 58.5 mg of *N*-tosylhydrazone **1a** and 76.8 mg of cyclohexylboronic acid afforded 20 mg of **4k** (32 % yield) as a white crystal after column chromatography on SiO_2. Rf = 0.4 (Hex/EtOAc 20:1).

^{1}H NMR (300 MHz, $CDCl_3$) δ 5.39 (s, 2H), 1.78 – 1.56 (m, 7H), 1.50 – 1.37 (m, 4H), 1.22 (d, *J* = 4.9 Hz, 12H), 1.17 – 1.00 (m, 4H), 0.94 – 0.89 (m, 6H).

^{13}C NMR (75 MHz, $CDCl_3$) δ 136.2 (CH), 130.4 (CH), 83.1 (C), 44.6 (CH), 36.7 (CH_2), 31.4 (C), 31.4 (CH_3), 30.0 (CH_2), 29.1 (CH_3), 28.0 (CH_2), 27.3 (CH_2), 27.2 (CH_2), 27.0 (CH_2), 25.0 (CH_3), 24.6 (CH_3), 23.2 (CH_2).

^{11}B NMR (129 MHz, $CDCl_3$) δ 33.89.

HRMS [ESI(+)]: m/z calcd. for $C_{20}H_{36}BO_2$: 319.2803 [M+H], found: 319.2816.

m.p. = 84.4 – 86.4 ^{o}C

2-(4,4-Diethyl-1-propylcyclohex-2-en-1-yl)-4,4,5,5-tetramethyl-1,3,2-dioxaborolane (4l)

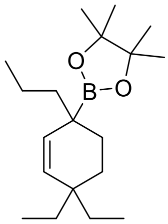

(390 nm lamp) 64.1 mg of *N*-tosylhydrazone **1b** and 53 mg of propylboronic acid afforded 34 mg of **4l** (56 % yield) as a colourless oil after column chromatography on SiO_2. Rf = 0.30 (Hex/EtOAc 40:1).

^{1}H NMR (300 MHz, $CDCl_3$) δ 5.55 (d, *J* = 10.2 Hz, 1H), 5.33 (d, *J* = 10.2 Hz, 1H), 1.83 – 1.74 (m, 1H), 1.51 – 1.23 (m, 11H), 1.22 (d, *J* = 1.4 Hz, 12H), 0.92 – 0.83 (m, 3H)0.83 – 0.72 (m, 6H).

^{13}C NMR (75 MHz, $CDCl_3$) δ 133.6 (CH), 132.9 (CH), 83.1 (C), 41.8 (CH_2), 37.1 (C), 32.2 (CH_2), 31.7 (CH_2), 30.2 (CH_2), 27.7 (CH_2), 24.9 (CH_3), 24.7 (CH_3), 19.3 (CH_2), 15.2 (CH_3), 8.5 (CH_3), 8.4 (CH_3).

^{11}B NMR (129 MHz, $CDCl_3$) δ 33.53.

HRMS [ESI(+)]: m/z calcd. for $C_{19}H_{36}BO_2$: 307.2803 [M+H], found: 307.2811.

4,4,5,5-Tetramethyl-2-(4'-propyl-3',4'-dihydro-2'H-[1,1':1',1''-terphenyl]-4'-yl)-1,3,2-dioxaborolane (4m)

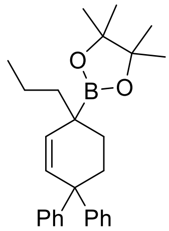

(390 nm lamp) 83.3 mg of *N*-tosylhydrazone **1c** and 53 mg of propylboronic acid afforded 55.7 mg of **4m** (70 % yield) as a white solid after column chromatography on SiO_2. Rf = 0.22 (Hex/EtOAc 20:1).

^{1}H NMR (300 MHz, $CDCl_3$) δ 7.32 – 7.11 (m, 10H), 5.99 (d, *J* = 10.0 Hz, 1H), 5.88 (d, *J* = 10.0 Hz, 1H), 2.40 (dd, *J* = 13.2, 5.5 Hz, 1H), 2.26 (td, *J* = 13.3, 12.6, 2.7 Hz, 1H), 1.86 (dt, *J* = 11.8, 3.6 Hz, 1H), 1.55 – 1.42 (m, 1H), 1.41 – 1.28 (m, 4H), 1.25 (d, *J* = 3.0 Hz, 12H), 0.89 (t, *J* = 6.8 Hz, 3H).

^{13}C NMR (75 MHz, $CDCl_3$) δ 150.5 (C), 148.9 (C), 134.2 (CH), 132.5 (CH), 128.5 (CH), 128.0 (CH), 128.0 (CH), 127.8 (CH), 125.7 (CH), 125.6 (CH), 83.4 (C), 48.8 (C), 41.2 (CH_2), 35.3 (CH_2), 27.5 (CH_2), 25.0 (CH_3), 24.7 (CH_3), 19.4 (CH_2), 15.1 (CH_3).

^{11}B NMR (129 MHz, $CDCl_3$) δ 34.00.

HRMS [ESI(+)]: m/z calcd. for $C_{27}H_{36}BO_2$: 403.2803 [M+H], found: 403.2816.

m.p. = 116.4 – 117.4 ºC

4,4,5,5-Tetramethyl-2-(3-methyl-1-propylcyclohex-2-en-1-yl)-1,3,2-dioxaborolane (4n)

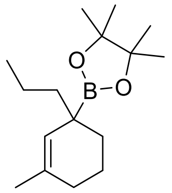

(390 nm lamp) 55.7 mg of *N*-tosylhydrazone **1d** and 53 mg of propylboronic acid afforded 40.1 mg of **4n** (76 % yield) as a colourless oil.

^{1}H NMR (300 MHz, $CDCl_3$) δ 5.27 (s, 1H), 1.89 – 1.78 (m, 3H), 1.76 – 1.67 (m, 1H), 1.64 (s, 3H), 1.60 – 1.47 (m, 1H), 1.47 – 1.26 (m, 4H), 1.21 (d, *J* = 3.2 Hz, 12H), 1.12 (ddd, *J* = 12.4, 11.2, 2.9 Hz, 1H), 0.90 – 0.83 (m, 3H).

^{13}C NMR (75 MHz, $CDCl_3$) δ 132.1 (C), 127.5 (CH), 83.0 (C), 42.2 (CH_2), 30.7 (CH_2), 30.3 (CH_2), 25.0 (CH_3), 24.6 (CH_2), 24.4 (CH_3), 21.5 (CH_2), 19.4 (CH_3), 15.2 (CH_3).

^{11}B NMR (129 MHz, $CDCl_3$) δ 33.84.

HRMS [ESI(+)]: m/z calcd. for $C_{16}H_{30}BO_2$: 265.2333 [M+H], found: 249.2340.

,4,5,5-Tetramethyl-2-(3-methyl-1-propylcyclopent-2-en-1-yl)-1,3,2-dioxaborolane (4o)

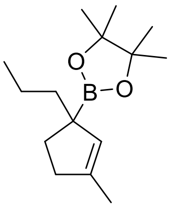

(390 nm lamp) 52.9 mg of *N*-tosylhydrazone **1h** and 53 mg of propylboronic acid afforded 41.6 mg of **4o** (84 % yield) as a yellow oil.

^{1}H NMR (300 MHz, $CDCl_3$) δ 5.21 (s, 1H), 2.25 – 2.16 (m, 2H), 2.15 – 2.05 (m, 1H), 1.69 (s, 3H), 1.55 – 1.49 (m, 1H), 1.48 – 1.41 (m, 1H), 1.32 – 1.23 (m, 3H), 1.21 (d, *J* = 2.4 Hz, 12H), 0.86 (t, *J* = 7.0 Hz, 3H).

^{13}C NMR (75 MHz, $CDCl_3$) δ 138.7 (C), 130.3 (CH), 83.0 (C), 41.2 (CH_2), 36.8 (CH_2), 33.6 (CH_2), 24.8 (CH_3), 24.7 (CH_3), 20.4 (CH_2), 16.9 (CH_3), 15.1 (CH_3).

^{11}B NMR (129 MHz, $CDCl_3$) δ 33.94.

HRMS [APCI(+)]: m/z calcd. for C_9H_{15}: 123.1168 [M-Bpin], found: 123.1167.

4,4,5,5-Tetramethyl-2-(3-methyl-1-phenethylcyclopent-2-en-1-yl)-1,3,2-dioxaborolane (4p)

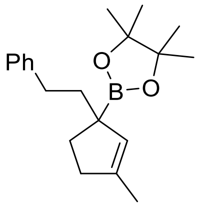

(390 nm lamp) 52.9 mg of *N*-tosylhydrazone **1h** and 90 mg of phenethylboronic acid afforded 35 mg of **4p** (57 % yield) as a colourless oil after column chromatography on SiO_2. Rf = 0.32 (Hex/EtOAc 20:1).

^{1}H NMR (300 MHz, $CDCl_3$) δ 7.32 – 7.22 (m, 2H), 7.21 – 7.11 (m, 3H), 5.28 (q, *J* = 1.6 Hz, 1H), 2.55 (tt, *J* = 9.8, 7.3 Hz, 2H), 2.32 – 2.15 (m, 3H), 1.82 (ddd, *J* = 12.9, 10.7, 6.7 Hz, 1H), 1.74 (s, 3H), 1.71 – 1.58 (m, 2H), 1.25 (d, *J* = 2.5 Hz, 12H).

^{13}C NMR (75 MHz, $CDCl_3$) δ 143.6 (C), 139.4 (C), 129.7 (CH), 128.5 (CH), 128.3 (CH), 125.6 (CH), 83.2 (C), 40.8 (CH_2), 36.8 (CH_2), 33.6 (CH_2), 33.5 (CH_2), 24.9 (CH_3), 24.7 (CH_3), 16.9 (CH_3).

^{11}B NMR (129 MHz, $CDCl_3$) δ 34.10.

HRMS [APCI(+)]: No molecular peak detected.

2-(1-Cyclopropyl-3-methylcyclopent-2-en-1-yl)-4,4,5,5-tetramethyl-1,3,2-dioxaborolane (4q)

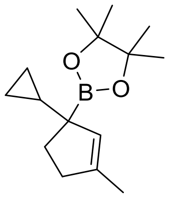

(390 nm lamp) 52.9 mg of *N*-tosylhydrazone **1h** and 51.6 mg of cyclopropylboronic acid afforded 32.4 mg of **4q** (66 % yield) as a colourless oil.

^{1}H NMR (300 MHz, $CDCl_3$) δ 5.05 (s, 1H), 2.29 – 2.07 (m, 3H), 1.69 (s, 3H), 1.68 – 1.61 (m, 1H), 1.22 (d, *J* = 1.8 Hz, 12H), 0.81 (tt, *J* = 8.3, 5.4 Hz, 1H), 0.33 – 0.12 (m, 4H).

^{13}C NMR (75 MHz, $CDCl_3$) δ 140.4 (C), 127.1 (CH), 83.0 (C), 36.8 (CH_2), 33.5 (CH_2), 24.9 (CH_2), 24.6 (CH_3), 17.6 (CH_3), 17.0 (CH_3), 1.5 (CH), 0.5 (CH_3).

^{11}B NMR (129 MHz, $CDCl_3$) δ 33.69.

HRMS [ESI(+)]: m/z calcd. for $C_{15}H_{26}BO_2$: 249.2020 [M+H], found: 249.2026.

2-(1-(But-3-en-1-yl)-3-methylcyclopent-2-en-1-yl)-4,4,5,5-tetramethyl-1,3,2-dioxaborolane (4r)

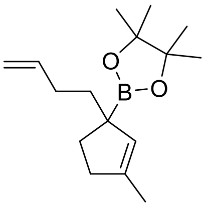

(390 nm lamp) 52.9 mg of *N*-tosylhydrazone **1h** and 60 mg of 3-butenylboronic acid afforded 25.5 mg of **4r** (50% yield) as a colourless oil after column chromatography on SiO_2. Rf = 0.28 (Hex/EtOAc 40:1).

^{1}H NMR (300 MHz, $CDCl_3$) δ 5.82 (ddt, *J* = 16.8, 10.1, 6.6 Hz, 1H), 5.21 (q, *J* = 1.7 Hz, 1H), 5.04 – 4.83 (m, 2H), 2.27 – 2.18 (m, 2H), 2.17 – 2.08 (m, 1H), 2.05 – 1.93 (m, 2H), 1.70 (s, 3H), 1.65 – 1.50 (m, 2H), 1.41 (ddd, *J* = 12.9, 10.1, 6.6 Hz, 1H), 1.22 (d, *J* = 2.5 Hz, 12H).

^{13}C NMR (75 MHz, $CDCl_3$) δ 139.8 (CH), 139.2 (C), 129.9 (CH), 114.0 (CH_2), 83.1 (C), 37.8 (CH_2), 36.8 (CH_2), 33.5 (CH_2), 31.6 (CH_2), 24.9 (CH_3), 24.7 (CH_3), 16.9 (CH_3).

^{11}B NMR (129 MHz, $CDCl_3$) δ 34.00.

HRMS [APCI(+)]: No molecular peak detected.

6-(3-Methyl-1-(4,4,5,5-tetramethyl-1,3,2-dioxaborolan-2-yl)cyclopent-2-en-1-yl)hexan-2-one (4s)

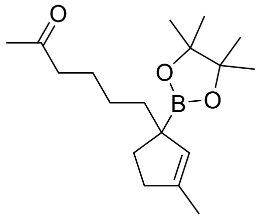

(390 nm lamp) 52.9 mg of *N*-tosylhydrazone **1h** and 86.4 mg of (5-oxohexyl)boronic acid afforded 44 mg of **4s** (72% yield) as a colourless oil after column chromatography on SiO_2. Rf = 0.16 (Hex/EtOAc 8:1).

^{1}H NMR (300 MHz, $CDCl_3$) δ 5.82 (ddt, *J* = 16.8, 10.1, 6.6 Hz, 1H), 5.21 (q, *J* = 1.7 Hz, 1H), 5.04 – 4.83 (m, 2H), 2.27 – 2.18 (m, 2H), 2.17 – 2.08 (m, 1H), 2.05 – 1.93 (m, 2H), 1.70 (s, 3H), 1.65 – 1.50 (m, 2H), 1.41 (ddd, *J* = 12.9, 10.1, 6.6 Hz, 1H), 1.22 (d, *J* = 2.5 Hz, 12H).

^{13}C NMR (75 MHz, $CDCl_3$) δ 139.8 (CH), 139.2 (C), 129.9 (CH), 114.0 (CH_2), 83.1 (C), 37.8 (CH_2), 36.8 (CH_2), 33.5 (CH_2), 31.6 (CH_2), 24.9 (CH_3), 24.7 (CH_3), 16.9 (CH_3).

^{11}B NMR (129 MHz, $CDCl_3$) δ 34.00.

HRMS [APCI(+)]: No molecular peak detected.

Benzyl 4-propyl-4-(4,4,5,5-tetramethyl-1,3,2-dioxaborolan-2-yl)-3,4-dihydropyridine-1(2H)-carboxylate (4t)

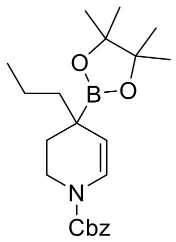

(390 nm lamp) 79.9 mg of *N*-tosylhydrazone **1f** and 53 mg of propylboronic acid afforded 44.7 mg of **4t** (58% yield) as a colourless oil after column chromatography on SiO_2. Rf = 0.5 (Hex/EtOAc 20:1).

The compound is present in the NMR spectra as a 1.5:1 mixture of rotamers.

^{1}H NMR (300 MHz, $CDCl_3$) δ 7.45 – 7.28 (m, 5H), 6.85 (d, *J* = 8.4 Hz, *min. rot.*), 6.77 (d, *J* = 8.4 Hz, 1H), 5.21 (d, *J* = 12.0 Hz, 1H), 5.17 (d, *J* = 12.0 Hz, 1H), 4.90 (d, J = 8.4 Hz, *min. rot.*), 4.80 (dd, J = 8.4, 1.2 Hz, 1H), 4.00 – 3.76 (m, 1H), 3.44 (m, 1H), 2.13 – 1.92 (m, 1H), 1.59 – 1.27 (m, 4H), 1.24 (s, *min. rot.*), 1.23 (s, 12H), 0.91 (t, J = 6.9 Hz, 3H).

^{13}C NMR (75 MHz, $CDCl_3$) δ 153.6 (C, *min. rot.*), 153.2 (C), 136.6 (C), 128.6 (CH), 128.2 (CH), 128.2 (CH), 128.1 (CH), 123.2 (CH, *min. rot.*), 122.7 (CH), 113.5 (CH, *min. rot.*), 113.0 (CH), 83.5 (C), 67.5 (CH_2), 67.4 (CH_2, *min. rot.*), 41.3 (CH_2), 40.9 (CH_2, *min. rot.*), 40.8 (CH_2), 29.6 (CH_2), 24.9 (CH_3), 24.6 (CH_3), 19.4 (CH_2), 15.0 (CH_3).

^{11}B NMR (129 MHz, $CDCl_3$) δ 33.71.

HRMS [ESI(+)]: m/z calcd. for $C_{22}H_{32}BNO_4$+Na: 408.2317 [M+Na], found: 408.2318.

Benzyl 4-phenethyl-4-(4,4,5,5-tetramethyl-1,3,2-dioxaborolan-2-yl)-3,4-dihydropyridine-1(2H)-carboxylate (4u)

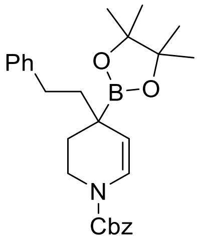

(390 nm lamp) 79.9 mg of *N*-tosylhydrazone **1f** and 90 mg of phenethylboronic acid afforded 58 mg of **4u** (65% yield) as a yellow oil after column chromatography on SiO_2. Rf = 0.20 (Hex/EtOAc 8:1).

The compound is present in the NMR spectra as a 1.5:1 mixture of rotamers.

^{1}H NMR (300 MHz, $CDCl_3$) δ 7.40 – 7.12 (m, 10H), 6.89 (d, *J* = 8.4 Hz, *min.rot.*), 6.80 (d, *J* = 8.4 Hz, 1H), 5.17 (s, 2H), 4.93 (d, *J* = 8.4 Hz, *min.rot.*), 4.82 (d, *J* = 8.4 Hz, 1H), 4.00 – 3.81 (m, 1H), 3.58 – 3.39 (m, 1H), 2.72 – 2.48 (m, 2H), 2.20 – 2.00 (m, 1H), 1.92 – 1.75 (m, 1H), 1.75 – 1.45 (m, 3H), 1.24 (d, *J* = 1.6 Hz, 12H).

^{13}C NMR (75 MHz, $CDCl_3$) δ 153.58 (C, *min.rot.*), 153.19 (C), 142.87 (C), 136.49 (C), 128.62 (CH), 128.45 (CH), 128.40 (CH), 128.22 (CH), 128.16 (CH), 125.83 (CH), 123.72 (CH, *min.rot.*), 123.18 (CH), 112.66 (CH, *min.rot.*), 112.26 (CH), 83.63 (C), 67.53 (CH2), 67.44 (CH2, *min.rot.*) 40.95 (CH2), 40.76 (CH2, *min.rot.*), 32.64 (CH2), 29.46 (CH2), 24.97 (CH3), 24.68 (CH3).

^{11}B NMR (129 MHz, $CDCl_3$) δ 33.47.

HRMS [APCI(+)]: m/z calcd. for $C_{27}H_{35}BNO_4$: 448.2654 [M+H], found: 448.2648.

Benzyl 4-(3-bromopropyl)-4-(4,4,5,5-tetramethyl-1,3,2-dioxaborolan-2-yl)-3,4-dihydropyridine-1(2H)-carboxylate (4v)

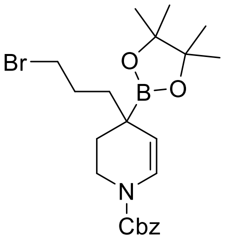

(390 nm lamp) 79.9 mg of *N*-tosylhydrazone **1f** and 100.1 mg 3-bromopropylboronic acid afforded 38.2 mg of **4v** (42% yield) as a colourless oil after column chromatography on SiO_2. Rf = 0.34 (Hex/EtOAc 5:1).

The compound is present in the NMR spectra as a 1.5:1 mixture of rotamers.

^{1}H NMR (300 MHz, $CDCl_3$) δ 7.41 – 7.28 (m, 5H), 6.86 (d, *J* = 8.4 Hz, *min. rot.*), 6.78 (d, *J* = 8.4 Hz, 1H), 5.17 (s, 2H), 4.83 (d, *J* = 8.5 Hz, *min. rot.*), 4.73 (d, *J* = 8.5 Hz, 1H), 3.86 (ddd, *J* = 20.5, 10.4, 4.6 Hz, 1H), 3.53 – 3.40 (m, 1H), 3.37 (t, *J* = 6.8 Hz, 2H), 2.08 – 1.93 (m, 1H), 1.93 – 1.78 (m, 2H), 1.61 (td, *J* = 11.7, 10.6, 5.3 Hz, 1H), 1.55 – 1.37 (m, 2H), 1.22 (d, *J* = 2.2 Hz, 12H).

^{13}C NMR (75 MHz, $CDCl_3$) δ 153.55 (C, *min.rot.*), 153.14 (C), 136.43 (C), 128.62 (CH), 128.24 (CH), 128.17 (CH), 123.90 (CH, *min.rot.*), 123.36 (CH), 112.23 (CH, *min.rot.*), 111.84 (CH), 83.67 (C), 67.55 (CH2), 67.46 (CH2, *min.rot.*), 40.72 (CH2, *min.rot.*), 40.63 (CH2), 37.11 (CH2), 34.19 (CH2), 29.55 (CH2), 29.39 (CH2, *min.rot.*), 24.90 (CH3), 24.64 (CH3).

^{11}B NMR (129 MHz, $CDCl_3$) δ 33.21.

HRMS [ESI(+)]: m/z calcd. for $C_{22}H_{31}BBrNO_4$+Na: 486.1422 [M+Na], found: 486.1438.

Benzyl 4-cyclopropyl-4-(4,4,5,5-tetramethyl-1,3,2-dioxaborolan-2-yl)-3,4-dihydropyridine-1(2H)-carboxylate (4w)

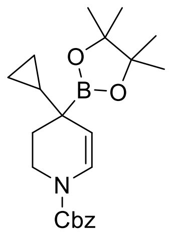

(390 nm lamp) 79.9 mg of *N*-tosylhydrazone **1f** and 51.6 mg of cyclopropylboronic acid afforded 52 mg of **4w** (68% yield) as a colourless oil after column chromatography on SiO_2. Rf = 0.2 (Hex/EtOAc 8:1).

The compound is present in the NMR spectra as a 1.5:1 mixture of rotamers.

^{1}H NMR (300 MHz, $CDCl_3$) δ 7.43 – 7.21 (m, 5H), 6.87 (d, *J* = 8.4 Hz, *min. rot.*), 6.79 (d, *J* = 8.4 Hz, 1H), 5.17 (s, 2H), 4.75 (d, *J* = 8.5 Hz, *min. rot*), 4.64 (d, *J* = 8.4 Hz, 1H), 3.93 – 3.78 (m, 1H), 3.43 (ddd, *J* = 13.0, 9.4, 3.5 Hz, 1H), 2.09 – 1.95 (m, 1H), 1.64 (ddd, *J* = 13.5, 9.3, 4.1 Hz, 1H), 1.22 (d, *J* = 3.0 Hz, 12H), 0.85 – 0.68 (m, 1H), 0.39 – 0.20 (m, 4H).

^{13}C NMR (75 MHz, $CDCl_3$) δ 153.6 (C, *min.rot.*), 153.2 (C), 136.5 (CH), 128.6 (CH), 128.2 (CH), 128.1 (CH), 124.4 (CH, *min.rot.*), 123.8 (CH), 110.0 (CH, *min.rot.*), 109.7 (CH), 83.5 (C), 67.5 (CH_2), 67.4 (CH_2, *min.rot.*), 40.8 (CH_2, *min.rot.*), 40.8 (CH_2) 30.4 (CH_2), 24.9 (CH_3), 24.5 (CH_3), 18.0 (CH_3), 1.0 (CH_2), 0.4 (CH_2).

^{11}B NMR (129 MHz, $CDCl_3$) δ 32.57.

HRMS [ESI(+)]: m/z calcd. for $C_{22}H_{30}BNO_4$+Na: 406.2160 [M+Na], found: 406.2154.

Benzyl 4-(but-3-en-1-yl)-4-(4,4,5,5-tetramethyl-1,3,2-dioxaborolan-2-yl)-3,4-dihydropyridine-1(2H)-carboxylate (4x)

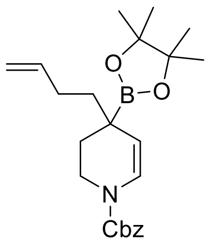

(390 nm lamp) 79.9 mg of *N*-tosylhydrazone **1f** and 60 mg of 3-butenylboronic acid afforded 32.2 mg of **4x** (41% yield) as a colourless oil after column chromatography on SiO_2. Rf = 0.2 (Hex/EtOAc 8:1).

The compound is present in the NMR spectra as a 1.5:1 mixture of rotamers.

^{1}H NMR (300 MHz, $CDCl_3$) δ 7.46 – 7.29 (m, 5H), 6.85 (d, *J* = 8.4 Hz, *min. rot.*), 6.77 (d, *J* = 8.4 Hz, 1H), 5.80 (ddt, *J* = 16.8, 10.1, 6.6 Hz, 1H), 5.17 (s, 2H), 5.00 (dd, *J* = 16.8, 1.7 Hz, 1H), 4.93 (dd, *J* = 10.1, 1.6 Hz, 1H), 4.87 (d, *J* = 8.4 Hz, *min. rot.*), 4.77 (d, *J* = 8.4 Hz, 1H), 3.97 – 3.77 (m, 1H), 3.55 – 3.35 (m, 1H), 2.13 – 1.94 (m, 3H), 1.69 – 1.54 (m, 1H), 1.54 – 1.35 (m, 2H), 1.22 (d, *J* = 2.4 Hz, 12H).

^{13}C NMR (75 MHz, $CDCl_3$) δ 153.57 (C), 153.19 (C), 139.03 (CH), 136.50 (C), 128.61 (CH), 128.21 (CH), 128.16 (CH), 128.12 (CH), 123.56 (CH), 123.02 (CH), 114.50 (CH2), 112.87 (CH), 112.47 (CH), 83.56 (C), 83.52 (C), 67.51 (CH2), 67.41 (CH2), 40.81 (CH2), 40.71 (CH2), 37.96 (CH2), 30.48 (CH2), 29.45 (CH2), 29.37 (CH2), 24.91 (CH3), 24.64 (CH3).

^{11}B NMR (129 MHz, $CDCl_3$) δ 33.28.

HRMS [ESI(+)]: m/z calcd. for $C_{23}H_{32}BNO_4$+Na: 420.2317 [M+Na], found: 420.2333.

Benzyl 4-(5-oxohexyl)-4-(4,4,5,5-tetramethyl-1,3,2-dioxaborolan-2-yl)-3,4-dihydropyridine-1(2H)-carboxylate (4y)

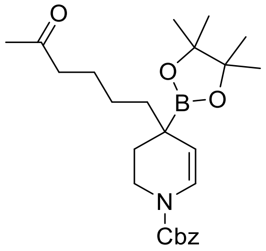

(390 nm lamp) 79.9 mg of *N*-tosylhydrazone **1f** and 86.4 mg of (5-oxohexyl)boronic acid afforded 47.3 mg of **4y** (54% yield) as a white solid after column chromatography on SiO_2. Rf = 0.23 (Hex/EtOAc 3:1).

The compound is present in the NMR spectra as a 1.5:1 mixture of rotamers.

^{1}H NMR (300 MHz, $CDCl_3$) δ 7.46 – 7.29 (m, 5H), 6.83 (d, *J* = 8.4 Hz, *min. rot.*), 6.74 (d, *J* = 8.4 Hz, 1H), 5.15 (s, 2H), 4.83 (d, *J* = 8.4 Hz, *min. rot.*), 4.73 (d, *J* = 8.4 Hz, 1H), 3.85 (td, *J* = 13.7, 6.9 Hz, 1H), 3.53 – 3.32 (m, 1H), 2.40 (t, *J* = 7.4 Hz, 2H), 2.11 (s, 3H), 1.99 (td, *J* = 13.1, 6.5 Hz, 1H), 1.61 – 1.38 (m, 4H), 1.31 – 1.23 (m, 3H), 1.20 (d, *J* = 2.7 Hz, 12H).

^{13}C NMR (75 MHz, $CDCl_3$) δ 209.12 (C), 153.54 (C, *min.rot.*), 153.15 (C), 136.48 (C), 128.59 (CH), 128.19 (CH), 128.14 (CH), 123.44 (CH, *min.rot.*), 122.90 (CH), 112.98 (CH, *min.rot.*), 112.57 (CH), 83.52 (C), 67.48 (CH2), 67.38 (CH2, *min.rot.*), 43.62 (CH2), 40,84 (CH2, *min.rot.*), 40.74 (CH2), 38.42 (CH2), 29.97 (CH3), 29.51 (CH2), 29.43 (CH2, *min.rot.*), 25.58 (CH2), 24.88 (CH2), 24.61 (CH3), 24.49 (CH3).

^{11}B NMR (129 MHz, $CDCl_3$) δ 33.24.

HRMS [ESI(+)]: m/z calcd. for $C_{25}H_{36}BNO_5$+Na: 464.2579 [M+Na], found: 464.2564.

m.p. = 75.6 – 77.6 ºC

(8R,9S,10R,13S,14S,17S)-10,13,17-Trimethyl-3-propyl-3-(4,4,5,5-tetramethyl-1,3,2-dioxaborolan-2-yl)-2,3,6,7,8,9,10,11,12,13,14,15,16,17-tetradecahydro-1H-cyclopenta[a]phenanthren-17-ol (4z)

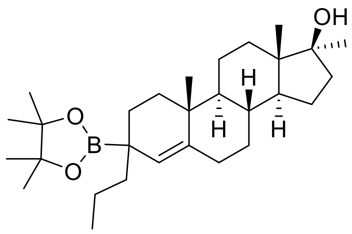

(390 nm lamp) 94.1 mg of *N*-tosylhydrazone **1k** and 53 mg of propylboronic acid afforded 54.8 mg of **4z** as a 1:1 mixture of diasteroisomers (60% yield) as a colourless oil after column chromatography on SiO_2. Rf = 0.27 (Hex/EtOAc 5:1).

The compound is present in the NMR spectra as a 1:1 mixture of diasteroisomers.

^{1}H NMR (300 MHz, $CDCl_3$) δ 5.23 (s, 1H, major isomer), 5.13 (s, 1H, minor isomer), 2.25 – 2.12 (m, 2H), 2.06 – 1.90 (m, 3H), 1.68 – 1.20 (m, 71H), 1.03 – 0.97 (m, 6H), 0.91 – 0.83 (m, 14H).

^{13}C NMR (75 MHz, $CDCl_3$) δ 143.56, 142.77, 126.06, 125.17, 83.04, 82.96, 81.89, 55.47, 53.68, 50.67, 50.60, 45.73, 45.61, 42.62, 40.75, 39.17, 37.55, 37.29, 37.17, 33.62, 33.45, 33.25, 32.99, 32.79, 31.92, 31.85, 29.84, 27.78, 25.92, 25.59, 24.92, 24.72, 24.59, 23.42, 21.56, 21.26, 20.45, 19.97, 19.89, 19.28, 15.22, 15.16, 14.10, 14.04.

^{11}B NMR (129 MHz, $CDCl_3$) δ 34.23.

HRMS [ESI(+)]: m/z calcd. for $C_{29}H_{50}BO_3$: 457.3848 [M+H], found: 457.3851.

(8R,9S,10R,13S,14S,17S)-3-Cyclopropyl-10,13,17-trimethyl-3-(4,4,5,5-tetramethyl-1,3,2-dioxaborolan-2-yl)-2,3,6,7,8,9,10,11,12,13,14,15,16,17-tetradecahydro-1H-cyclopenta[a]phenanthren-17-ol (4aa)

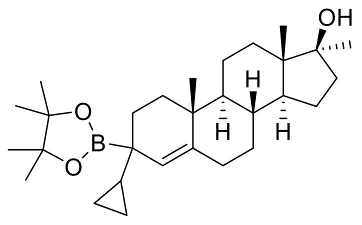

(390 nm lamp) 94.1 mg of *N*-tosylhydrazone **1k** and 51.6 mg of cyclopropylboronic acid afforded 60 mg of **4aa** as a 1:1 mixture of diasteroisomers (66% yield) as a colourless oil after column chromatography on SiO_2. Rf = 0.44 (Hex/EtOAc 3:1).

The compound is present in the NMR spectra as a 1:1 mixture of diasteroisomers.

^{1}H NMR (300 MHz, $CDCl_3$) δ 5.13 (s, 1H, major isomer), 5.04 (s, 1H, minor isomer), 2.25 – 2.11 (m, 2H), 2.09 – 1.88 (m, 3H), 1.70 – 1.19 (m, 65H), 1.01 – 0.95 (m, 6H), 0.86 – 0.69 (m, 10H), 0.34 – 0.24 (m, 6H).

^{13}C NMR (75 MHz, $CDCl_3$) δ 145.60, 143.29, 123.93, 121.00, 83.08, 83.04, 81.88, 55.42, 54.06, 50.58, 45.69, 45.60, 39.15, 37.71, 37.26, 37.21, 37.14, 33.73, 33.59, 33.36, 33.13, 32.77, 31.89, 31.81, 27.95, 27.75, 25.93, 24.93, 24.81, 24.72, 24.62, 24.58, 23.42, 21.47, 21.20, 20.09, 19.97, 19.23, 17.82, 14.09, 14.03, 1.98, 1.12, 0.72, -0.43.

^{11}B NMR (129 MHz, $CDCl_3$) δ 32.97.

HRMS [EI]: m/z calcd. for $C_{29}H_{47}BO_3$: 454.3618 [M], found: 454.3625.

(8R,9S,10R,13S,14S,17S)-3-Cyclobutyl-10,13,17-trimethyl-3-(4,4,5,5-tetramethyl-1,3,2-dioxaborolan-2-yl)-2,3,6,7,8,9,10,11,12,13,14,15,16,17-tetradecahydro-1H-cyclopenta[a]phenanthren-17-ol (4ab)

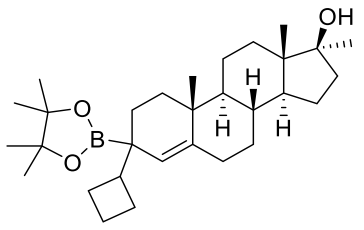

(390 nm lamp) 94.1 mg of *N*-tosylhydrazone **1k** and 60 mg of cyclobutylboronic acid afforded 60 mg of **4ab** as a 1:1 mixture of diasteroisomers (41% yield) as a colourless oil after column chromatography on SiO_2. Rf = 0.44 (Hex/EtOAc 3:1).

The compound is present in the NMR spectra as a 1:1 mixture of diasteroisomers.

^{1}H NMR (300 MHz, $CDCl_3$) δ 5.30 (s, 1H, minor isomer), 5.22 (s, 1H, major isomer), 2.49 – 2.11 (m, 4H), 2.11 – 1.96 (m, 3H), 1.82 – 1.18 (m, 71H), 1.01 – 0.94 (m, 6H), 0.89 – 0.78 (m, 8H), 0.70 – 0.47 (m, 2H).

^{13}C NMR (75 MHz, $CDCl_3$) δ 144.50, 143.36, 122.92, 122.22, 83.00, 81.89, 55.43, 53.09, 50.69, 50.58, 45.76, 45.61, 43.20, 43.03, 39.18, 37.70, 37.26, 37.17, 34.02, 33.93, 33.36, 33.18, 32.81, 31.91, 31.85, 25.93, 25.48, 25.35, 25.17, 25.00, 24.90, 24.70, 24.15, 24.06, 23.43, 21.52, 21.26, 20.83, 19.98, 18.49, 18.06, 14.13, 14.04.

^{11}B NMR (129 MHz, $CDCl_3$) δ 34.13.

HRMS [ESI(+)]: m/z calcd. for $C_{30}H_{49}BO_3$+Na: 491.3667 [M+Na], found: 491.3675

2-((8S,9S,10R,13R,14S,17R)-10,13-dimethyl-17-((R)-6-methylheptan-2-yl)-3-propyl-2,3,6,7,8,9,10,11,12,13,14,15,16,17-tetradecahydro-1H-cyclopenta[a]phenanthren-3-yl)-4,4,5,5-tetramethyl-1,3,2-dioxaborolane (4ac)

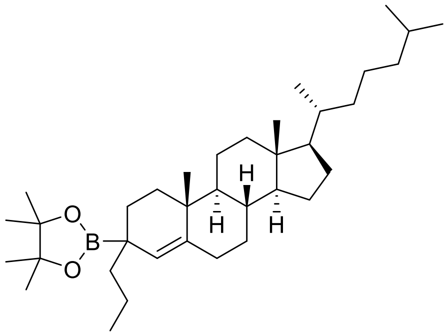

(390 nm lamp) 110.6 mg of *N*-tosylhydrazone **1l** and 53 mg of propylboronic acid afforded 56.5 mg of **4ac** as a 1:1 mixture of diasteroisomers (53% yield) as a colourless oil after column chromatography on SiO_2. Rf = 0.25 (Hex/EtOAc 40:1).

The compound is present in the NMR spectra as a 1:1 mixture of diasteroisomers.

^{1}H NMR (300 MHz, $CDCl_3$) δ 5.22 (s, 1H, minor isomer), 5.12 (s, 1H, major isomer), 2.27 – 2.08 (m, 2H), 2.03 – 1.89 (m, 4H), 1.88 – 0.64 (m, 118H).

^{13}C NMR (75 MHz, $CDCl_3$) δ 143.9, 143.1, 125.8, 124.9, 83.0, 82.9, 77.6, 77.2, 76.7, 56.5, 56.4, 56.3, 55.6, 53.6, 42.8, 42.7, 42.6, 40.8, 40.3, 40.2, 39.7, 37.5, 37.2, 37.0, 36.3, 36.0, 35.9, 34.0, 33.8, 33.2, 33.1, 32.9, 28.4, 28.2, 27.9, 25.6, 24.9, 24.9, 24.7, 24.6, 24.4, 24.0, 23.9, 23.0, 22.7, 22.0, 21.7, 20.5, 20.0, 19.9, 19.3, 18.8, 15.2, 15.2, 12.2, 12.2.

^{11}B NMR (129 MHz, $CDCl_3$) δ 34.67.

HRMS [ESI(+)]: m/z calcd. for $C_{36}H_{64}BO_2$: 539.4994 [M+H], found: 539.5004.

(Z)-4,4,5,5-tetramethyl-2-(3-methyl-2-(pent-2-en-1-yl)-1-propylcyclopent-2-en-1-yl)-1,3,2-dioxaborolane (4ad)

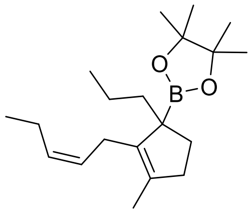

(370 nm lamp) 66.5 mg of *N*-tosylhydrazone **1i** and 53 mg of propylboronic acid afforded 25 mg of **4ad** (40% yield) as a yellow oil after column chromatography on SiO_2. Rf = 0.28 (Hex/EtOAc 100:1).

^{1}H NMR (401 MHz, $CDCl_3$) δ 5.37 – 5.17 (m, 2H), 2.86 – 2.72 (m, 2H), 2.31 – 2.03 (m, 5H), 1.80 – 1.63 (m, 2H), 1.62 (s, 3H), 1.45 (ddd, *J* = 12.4, 9.2, 7.6 Hz, 1H), 1.24 – 1.18 (m, 14H), 0.99 (t, *J* = 7.5 Hz, 3H), 0.88 (t, *J* = 7.0 Hz, 3H).

^{13}C NMR (75 MHz, $CDCl_3$) δ 137.6 (C), 132.5 (C), 130.6 (CH), 128.6 (CH), 82.8 (C), 38.9 (CH_2), 37.6 (CH_2), 32.1 (CH_2), 25.0 (CH_2), 24.9 (CH_3), 24.7 (CH_3), 20.7 (CH_2), 19.5 (CH_2), 15.1 (CH_3), 14.5 (CH_3), 14.3 (CH_3).

^{11}B NMR (129 MHz, $CDCl_3$) δ 33.87.

HRMS [APCI(+)]: m/z calcd. for $C_{20}H_{34}BO_2$: 317.2646 [M-2H+H], found: 317.2637.

2-((4R,4aS,6R)-4,4a-dimethyl-6-(prop-1-en-2-yl)-2-propyl-2,3,4,4a,5,6,7,8-octahydronaphthalen-2-yl)-4,4,5,5-tetramethyl-1,3,2-dioxaborolane (4ae)

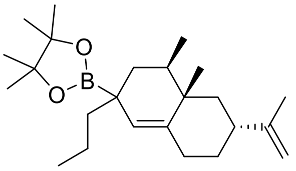

(390 nm lamp) 77.31 mg of *N*-tosylhydrazone **1m** and 53 mg of propylboronic acid afforded 30 mg of **4ae** as a 1:1 mixture of diasteroisomers (41% yield) as a colourless oil after column chromatography on SiO_2. Rf = 0.43 (Hex/EtOAc 20:1).

The compound is present in the NMR spectra as a 1:1 mixture of diasteroisomers.

^{1}H NMR (300 MHz, $CDCl_3$) δ 5.27 (s, 1H, minor isomer), 5.19 (s, 1H, major isomer), 4.72 – 4.61 (m, 4H), 2.36 – 2.17 (m, 4H), 2.17 – 2.05 (m, 2H), 1.90 – 1.69 (m, 10H), 1.62 – 1.39 (m, 8H), 1.40 – 1.15 (m, 34H), 0.94 – 0.80 (m, 20H).

^{13}C NMR (75 MHz, $CDCl_3$) (mixture) δ 151.2, 141.6, 141.0, 126.8, 125.7, 108.3, 108.2, 83.1, 83.0, 45.5, 45.2, 42.3, 41.2, 41.1, 40.9, 40.4, 37.9, 36.0, 35.8, 33.7, 33.4, 33.1, 32.9, 24.9, 24.7, 24.5, 21.1, 21.1, 20.2, 19.3, 19.0, 18.2, 16.0, 15.7, 15.2, 15.2.

^{11}B NMR (129 MHz, $CDCl_3$) δ 33.59.

HRMS [ESI(+)]: m/z calcd. for $C_{24}H_{42}BO_2$: 373.3272 [M+H], found: 373.3278.

4,4,5,5-Tetramethyl-2-((1S,2S,5R)-2,4,6,6-tetramethylbicyclo[3.1.1]hept-3-en-2-yl)-1,3,2-dioxaborolane (4af)

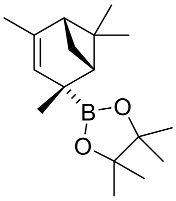

(390 nm lamp) 63.7 mg of *N*-tosylhydrazone **1j** and 36 mg of methylboronic acid afforded 28.8 mg of **4af** (52% yield) as a colourless oil after column chromatography on SiO_2. Rf = 0.45 (Hex/EtOAc 20:1).

[α]=-19.20 (c=0.7, T=18.3).

^{1}H NMR (300 MHz, $CDCl_3$) δ 5.24 (q, J = 1.7 Hz, 1H), 2.41 (ddd, J = 8.8, 6.2, 5.2 Hz, 1H), 2.09 (td, J = 6.0, 1.8 Hz, 1H), 1.89 – 1.81 (m, 1H), 1.68 (d, J = 1.6 Hz, 3H), 1.28 (s, 3H), 1.21 (s, 6H), 1.19 (s, 6H), 1.09 (s, 3H), 0.98 (s, 3H).

^{13}C NMR (75 MHz, $CDCl_3$) δ 140.9 (C), 122.6 (CH), 82.9 (C), 48.6 (CH), 47.2 (CH), 38.9 (C), 33.9 (CH_2), 27.5 (CH_3), 24.8 (CH_3), 24.4 (CH_3), 23.8 (CH_3), 23.5 (CH_3), 19.7 (CH_3).

^{11}B NMR (129 MHz, $CDCl_3$) δ 33.15.

HRMS [ESI (+)]: m/z calcd. for $C_{17}H_{30}BO_2$: 277.2333 [M+H], found: 277.2334.

3. Experimental and characterization data for alcohols 5, 7, 8, 9, 10, 11, 12, 13, 16

(*R)-((*R**)--6,6-Dimethyl-3-propylcyclohex-2-en-1-yl)(p-tolyl)methanol (5a)**

(390 nm lamp) 58.5 mg of *N*-tosylhydrazone **1a**, 53 mg of propylboronic acid and 47.3 μL of 4-methylbenzaldehyde afforded 48.8 mg of **5a** (90% yield) as a colourless oil after column chromatography on SiO_2. Rf = 0.4 (Hex/EtOAc 8:1).

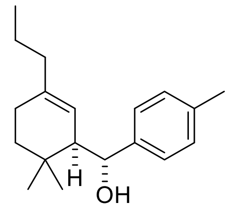

^{1}H NMR (300 MHz, $CDCl_3$) δ 7.24 (d, *J* = 8.1 Hz, 2H), 7.15 (d, *J* = 7.9 Hz, 2H), 4.72 (dt, *J* = 3.1, 1.7 Hz, 1H), 4.47 (dd, *J* = 8.8, 2.3 Hz, 1H), 2.36 (s, 3H), 2.35 – 2.27 (m, 1H), 1.98 – 1.72 (m, 4H), 1.69 (d, *J* = 2.5 Hz, 1H), 1.42 – 1.24 (m, 4H), 1.16 (s, 3H), 1.01 (s, 3H), 0.80 (t, *J* = 7.3 Hz, 3H).

^{13}C NMR (75 MHz, $CDCl_3$) δ 141.5 (C), 138.0 (C), 137.0 (C), 128.9 (CH), 127.3 (CH), 121.3 (CH), 76.5 (CH), 51.4 (CH), 39.7 (CH_2), 37.3 (CH_2), 31.7 (C), 30.8 (CH_3), 25.5 (CH_2), 22.6 (CH_3), 21.1 (CH_3), 20.7 (CH_2), 13.6 (CH_3).

HRMS [ESI(+)]: m/z calcd. for $C_{19}H_{28}O$+Na: 295.2032 [M+Na], found: 295.2030.

(*R)-((*R**)-6,6-Dimethyl-3-propylcyclohex-2-en-1-yl)(4-methoxyphenyl)methanol (5b)**

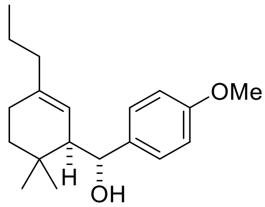

(390 nm lamp) 58.5 mg of *N*-tosylhydrazone **1a**, 53 mg of propylboronic acid and 49.6 µL of *p*-anisaldehyde afforded 37 mg of **5b** (65% yield) as a colourless oil after column chromatography on SiO_2. Rf = 0.4 (Hex/EtOAc 5:1).

^{1}H NMR (300 MHz, $CDCl_3$) δ 7.26 (d, *J* = 8.7 Hz, 2H), 6.86 (d, *J* = 8.7 Hz, 2H), 4.72 (dt, *J* = 3.3, 1.6 Hz, 1H), 4.47 (dd, *J* = 8.8, 2.4 Hz, 1H), 3.81 (s, 3H), 2.35 – 2.22 (m, 1H), 1.94 – 1.72 (m, 4H), 1.71 – 1.65 (m, 1H), 1.40 – 1.22 (m, 4H), 1.15 (s, 3H), 0.99 (s, 3H), 0.79 (t, *J* = 7.3 Hz, 3H).

^{13}C NMR (75 MHz, $CDCl_3$) δ 159.1 (C), 138.3 (C), 137.0 (C), 128.7 (CH), 121.5 (CH), 113.7 (CH), 77.0 (CH), 55.4 (CH), 51.7 (CH_3), 39.9 (CH_2), 37.4 (CH_2), 31.9 (C), 31.0 (CH_3), 25.8 (CH_2), 22.9 (CH_3), 20.9 (CH_2), 13.9 (CH_3).

HRMS [APCI(+)]: m/z calcd. for $C_{19}H_{28}O_2$+Na: 311.1982 [M+Na], found: 311.1979.

(*R)-((*R**)-6,6-Dimethyl-3-propylcyclohex-2-en-1-yl)(4-nitrophenyl)methanol (5c)**

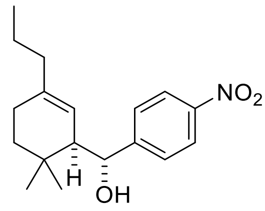

(390 nm lamp) 58.5 mg of *N*-tosylhydrazone **1a**, 53 mg of propylboronic acid and 61.7 mg of 4-nitrobenzaldehyde afforded 50.9 mg of **5c** (85% yield) as a white solid after column chromatography on SiO_2. Rf = 0.35 (Hex/EtOAc 5:1).

^{1}H NMR (300 MHz, $CDCl_3$) δ 7.94 (d, *J* = 8.7 Hz, 2H), 7.27 (d, *J* = 8.7 Hz, 2H), 4.49 – 4.36 (m, 2H), 2.06 (ddt, *J* = 7.9, 3.9, 2.0 Hz, 1H), 1.86 – 1.79 (m, 1H), 1.71 – 1.52 (m, 4H), 1.16 – 0.98 (m, 4H), 0.83 (s, 3H), 0.79 (s, 3H), 0.55 (t, *J* = 7.3 Hz, 3H).

^{13}C NMR (75 MHz, $CDCl_3$) δ 151.9 (C), 147.3 (C), 139.6 (C), 128.3 (CH), 123.5 (CH), 120.5 (CH), 76.6 (CH), 52.0 (CH), 39.8 (CH_2), 36.4 (CH_2), 32.1 (C), 30.3 (CH_3), 25.7 (CH_2), 24.3 (CH_3), 20.9 (CH_2), 13.9 (CH_3).

HRMS [APCI(+)]: m/z calcd. for $C_{11}H_{19}$: 151.1483 [M-$C_7H_6NO_3$], found: 151.1481.

m.p. = 69 – 71 ^{o}C

(*R)-((*R**)--6,6-Dimethyl-3-propylcyclohex-2-en-1-yl)(hydroxy)methyl)benzonitrile (5d)**

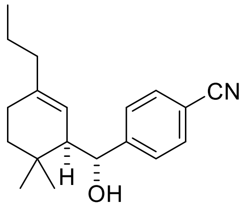

(390 nm lamp) 58.5 mg of *N*-tosylhydrazone **1a**, 53 mg of propylboronic acid and 53.5 mg of 4-formylbenzonitrile afforded 36.5 mg of **5d** (65% yield) as a white solid after column chromatography on SiO_2. Rf = 0.3 (Hex/EtOAc 5:1).

^{1}H NMR (300 MHz, $CDCl_3$) δ 7.61 (d, *J* = 8.1 Hz, 2H), 7.44 (d, *J* = 8.1 Hz, 2H), 4.65 – 4.54 (m, 2H), 2.26 (dd, *J* = 8.3, 3.4 Hz, 1H), 2.03 (s, 1H), 1.94 – 1.72 (m, 4H), 1.37 – 1.21 (m, 4H), 1.06 (s, 3H), 1.00 (s, 3H), 0.78 (t, *J* = 7.3 Hz, 3H).

^{13}C NMR (75 MHz, $CDCl_3$) δ 149.9 (C), 139.4 (C), 132.1 (CH), 128.3 (CH), 120.6 (CH), 119.0 (-CN), 111.2 (C), 76.7 (CH), 51.9 (CH), 39.8 (CH_2), 36.4 (CH_2), 32.1 (C), 30.3 (CH_3), 25.7 (CH_2), 24.2 (CH_3), 20.9 (CH_2), 13.9 (CH_3).

HRMS [ESI(+)]: m/z calcd. for $C_{19}H_{25}NO$+Na: 306.1828 [M+Na], found: 306.1831.

m.p. = 68 – 70 ºC

(*R)-((*R**)-6,6-Dimethyl-3-propylcyclohex-2-en-1-yl)(4-fluorophenyl)methanol (5e)**

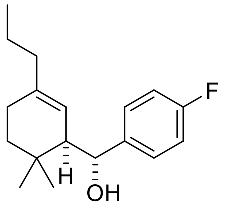

(390 nm lamp) 58.5 mg of *N*-tosylhydrazone **1a**, 53 mg of propylboronic acid and 45 μL of 4-fluorobenzaldehyde afforded 40.6 mg of **5e** (74% yield) as a colourless oil after column chromatography on SiO_2. Rf = 0.5 (Hex/EtOAc 5:1).

^{1}H NMR (300 MHz, $CDCl_3$) δ 7.35 – 7.27 (m, 2H), 7.06 – 6.97 (m, 2H), 4.67 (dd, *J* = 3.4, 1.8 Hz, 1H), 4.52 (dd, *J* = 8.6, 2.5 Hz, 1H), 2.26 (d, *J* = 8.5 Hz, 1H), 1.89 – 1.72 (m, 5H), 1.36 – 1.25 (m, 4H), 1.12 (s, 3H), 1.00 (s, 3H), 0.78 (t, *J* = 7.3 Hz, 3H).

^{13}C NMR (75 MHz, $CDCl_3$) δ 162.26 (C-F, d, $^1J_{C\text{-}F}$ = 245.2 Hz) 140.4 (C), 138.7 (C), 129.13 (CH, d, $^3J_{C\text{-}F}$ = 8.1 Hz), 121.1 (CH), 115.18 (CH, d, $^2J_{C\text{-}F}$ = 21.2 Hz)76.8 (CH), 51.9 (CH), 39.9 (CH_2), 37.0 (CH_2), 32.0 (C), 30.8 (CH_3), 25.8 (CH_2), 23.3 (CH_3), 20.9 (CH_2), 13.9 (CH_3).

^{19}F NMR (282 MHz, $CDCl_3$) δ -115.07.

HRMS [ESI(+)]: m/z calcd. for $C_{18}H_{25}FO$+Na: 299.1782 [M+Na], found: 299.1788.

(*R)-((*R**)-(4-bromophenyl)(-6,6-dimethyl-3-propylcyclohex-2-en-1-yl)methanol (5f)**

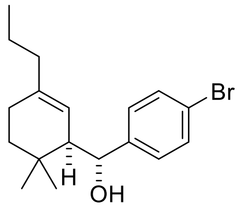

(390 nm lamp) 58.5 mg of *N*-tosylhydrazone **1a**, 53 mg of propylboronic acid and 40 μL of 4-bromobenzaldehyde afforded 34.6 mg of **5f** (52% yield) as a colourless oil after column chromatography on SiO_2. Rf = 0.33 (Hex/EtOAc 8:1).

^{1}H NMR (300 MHz, $CDCl_3$) δ 7.45 (d, *J* = 8.4 Hz, 2H), 7.21 (d, *J* = 8.4 Hz, 2H), 4.67 (dt, *J* = 3.4, 1.7 Hz, 1H), 4.49 (d, *J* = 8.4 Hz, 1H), 2.33 – 2.18 (m, 1H), 1.81 (h, *J* = 6.9, 5.9 Hz, 5H), 1.37 – 1.25 (m, 4H), 1.11 (s, 3H), 0.99 (s, 3H), 0.79 (t, *J* = 7.3 Hz, 3H).

^{13}C NMR (75 MHz, $CDCl_3$) δ 144.0 (C), 138.9 (C), 131.5 (CH), 129.3 (CH), 121.4 (C), 121.0 (CH), 76.9 (CH), 51.7 (CH), 39.9 (CH_2), 37.0 (CH_2), 32.0 (C), 30.8 (CH_3), 25.7 (CH_2), 23.4 (CH_3), 20.9 (CH_2), 13.9 (CH_3).

HRMS [EI(+)]: m/z calcd. for $C_{18}H_{23}Br$: 318.0983 [M-H_2O], found: 318.0978.

(*R)-(2,6-Dichlorophenyl)((*R**)-6,6-dimethyl-3-propylcyclohex-2-en-1-yl)methanol (5g)**

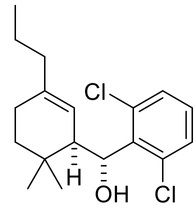

(390 nm lamp) 58.5 mg of *N*-tosylhydrazone **1a**, 53 mg of propylboronic acid and 70 mg of 2,6-dichlorobenzaldehyde afforded 33.4 mg of **5g** (51% yield) as a white solid after column chromatography on SiO_2. Rf = 0.35 (Hex/EtOAc 8:1).

^{1}H NMR (300 MHz, $CDCl_3$) δ 7.34 – 7.21 (m, 2H), 7.11 (t, J = 8.0 Hz, 1H), 5.30 (dd, J = 10.4, 9.2 Hz, 1H), 4.65 – 4.45 (m, 1H), 2.83 (d, J = 10.4 Hz, 1H), 2.75 – 2.60 (m, 1H), 2.03 – 1.89 (m, 1H), 1.89 – 1.76 (m, 1H), 1.71 (t, J = 7.5 Hz, 2H), 1.67 – 1.57 (m, 1H), 1.39 (dt, J = 12.8, 6.1 Hz, 1H), 1.28 – 1.17 (m, 2H), 1.14 (s, 3H), 1.11 (s,3 H), 0.70 (t, J = 7.3 Hz, 3H).

^{13}C NMR (75 MHz, $CDCl_3$) δ 138.4 (C), 135.9 (C), 134.3 (C), 129.8 (CH), 128.7 (CH), 120.2 (CH), 74.1 (CH), 49.8 (CH), 39.7 (CH_2), 35.4 (CH_2), 32.3 (C), 29.5 (CH_3), 25.9 (CH_2), 25.1 (CH_3), 20.9 (CH_2), 13.8 (CH_3)

HRMS [ESI(+)]: m/z calcd. for $C_{18}H_{24}Cl_2O$+Na: 349.1096 [M+Na], found: 349.1092

m.p. = 50 – 52 ^{o}C

(*R)-((*R**)-6,6-Dimethyl-3-propylcyclohex-2-en-1-yl)(3,4,5-trimethoxyphenyl)methanol (5h)**

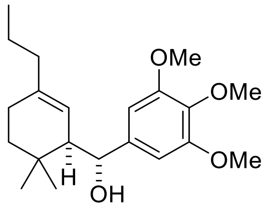

(390 nm lamp) 58.5 mg of *N*-tosylhydrazone **1a**, 53 mg of propylboronic acid and 78.5 mg of 3,4,5-trimethoxybenzaldehyde afforded 35.7 mg of **5h** (52% yield) as a colourless oil after column chromatography on SiO_2. Rf = 0.26 (Hex/EtOAc 3:1).

^{1}H NMR (300 MHz, $CDCl_3$) δ 6.55 (s, 2H), 4.67 (s, 1H), 4.40 (d, J = 8.8 Hz, 1H), 3.84 (d, J = 2.8 Hz, 9H), 2.30 – 2.19 (m, 1H), 1.83 (m, 5H), 1.40 – 1.22 (m, 4H), 1.14 (s, 3H), 1.01 (s, 3H), 0.77 (t, J = 7.3 Hz, 3H).

^{13}C NMR (75 MHz, $CDCl_3$) δ 153.2 (C), 140.5 (C), 138.4 (C), 137.3 (C), 121.4 (CH), 104.3 (CH), 77.9 (C), 61.0 (CH), 56.2 (CH), 51.6 (CH), 39.8 (CH_2), 37.3 (CH_2), 31.9 (C), 31.0 (CH_3), 25.8 (CH_2), 23.1 (CH_3), 20.9 (CH_2), 13.8 (CH_3).

HRMS [ESI(+)]: m/z calcd. for [$C_{21}H_{32}O_4$+Na]: 371.2193 [M+Na], found: 371.2186

(*R)-Benzofuran-2-yl((*R**)-6,6-dimethyl-3-propylcyclohex-2-en-1-yl)methanol (5i)**

(390 nm lamp) 58.5 mg of *N*-tosylhydrazone **1a**, 53 mg of propylboronic acid and 49.5 µL of benzofuran-2-carbaldehyde afforded 55.6 mg of **5i** (70% yield) as a yellow oil after column chromatography on SiO_2. Rf = 0.34 (Hex/EtOAc 8:1).

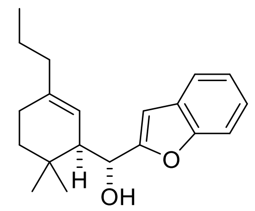

^{1}H NMR (300 MHz, $CDCl_3$) δ 7.60 – 7.52 (m, 1H), 7.52 – 7.43 (m, 1H), 7.32 – 7.17 (m, 2H), 6.61 (d, *J* = 0.9 Hz, 1H), 4.99 (dt, *J* = 3.3, 1.7 Hz, 1H), 4.75 (dd, *J* = 8.2, 4.6 Hz, 1H), 2.56 (d, *J* = 7.0 Hz, 1H), 2.03 (d, *J* = 5.0 Hz, 1H), 1.95 – 1.78 (m, 4H), 1.43 – 1.30 (m, 4H), 1.15 (s, 3H), 0.98 (s, 3H), 0.81 (t, *J* = 7.3 Hz, 3H).

^{13}C NMR (75 MHz, $CDCl_3$) δ 159.3 (C), 154.7 (C), 139.2 (C), 128.3 (C), 124.2 (CH), 122.9 (CH), 121.2 (CH), 120.2 (CH), 111.4 (CH), 104.1 (CH), 70.9 (CH), 50.1 (CH), 39.9 (CH_2), 37.3 (CH_2), 31.7 (C), 30.7 (CH_3), 25.7 (CH_2), 22.6 (CH_3), 21.0 (CH_2), 13.9 (CH_3).

HRMS [ESI(+)]: m/z calcd. for $C_{20}H_{26}O_2$+Na: 321.1825 [M+Na], found: 321.1824.

(*R)-((*R**)-6,6-Dimethyl-3-propylcyclohex-2-en-1-yl)(thiophen-2-yl)methanol (5j)**

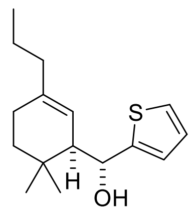

(390 nm lamp) 58.5 mg of *N*-tosylhydrazone **1a**, 53 mg of propylboronic acid and 37.4 µL of thiophene-2-carbaldehyde afforded 36.10 mg of **5j** (69% yield) as a colourless oil after column chromatography on SiO_2. Rf = 0.4 (Hex/Acetone 4:1).

^{1}H NMR (300 MHz, $CDCl_3$) δ 7.29 – 7.20 (m, 1H), 6.99 – 6.88 (m, 2H), 5.11 (dt, *J* = 3.2, 1.5 Hz, 1H), 4.93 (dd, *J* = 7.4, 3.1 Hz, 1H), 2.42 – 2.28 (m, 1H), 1.95 – 1.80 (m, 5H), 1.43 – 1.18 (m, 4H), 1.10 (s, 3H), 0.91 (s, 3H), 0.84 (t, *J* = 7.3 Hz, 3H).

^{13}C NMR (75 MHz, $CDCl_3$) δ 148.0 (C), 139.6 (C), 126.1 (CH), 125.7 (CH), 125.7 (CH), 120.1 (CH), 72.5 (CH), 52.1 (CH), 40.1 (CH_2), 36.5 (CH_2), 31.6 (C), 30.6 (CH_3), 25.9 (CH_2), 23.0 (CH_3), 20.9 (CH_2), 14.0 (CH_3).

HRMS [ESI(+)]: m/z calcd. for $C_{16}H_{24}OS$+Na: 287.1440 [M+Na], found: 287.1445

(*R)-((*R**)-6,6-Dimethyl-3-propylcyclohex-2-en-1-yl)(pyridin-4-yl)methanol (5k)**

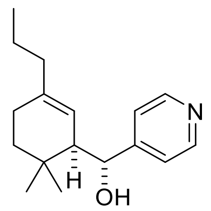

(390 nm lamp) 58.5 mg of *N*-tosylhydrazone **1a**, 53 mg of propylboronic acid and 58.4 µL of isonicotinaldehyde afforded 41 mg of **5k** (80% yield) as a yellow oil after column chromatography on SiO_2. Rf = 0.41 (Hex/EtOAc 4:1).

^{1}H NMR (300 MHz, $CDCl_3$) δ 8.42 (d, *J* = 5.0 Hz, 2H), 7.23 (d, *J* = 5.0 Hz, 2H), 4.75 – 4.60 (m, 1H), 4.52 (d, *J* = 7.8 Hz, 1H), 3.35 (bs, 1H), 2.35 – 2.20 (m, 1H), 1.87 – 1.72 (m, 4H), 1.36 – 1.23 (m, 4H), 1.06 (s, 3H), 0.99 (s, 3H), 0.77 (t, *J* = 7.3 Hz, 3H).

^{13}C NMR (75 MHz, $CDCl_3$) δ 153.9 (C), 149.3 (CH), 139.2 (C), 122.8 (CH), 120.7 (CH), 75.9 (CH), 51.5 (CH), 39.8 (CH_2), 36.4 (CH_2), 32.0 (C), 30.3 (CH_3), 25.7 (CH_2), 24.2 (CH_3), 20.9 (CH_2), 13.9 (CH_3).

HRMS [EI]: m/z calcd. for $C_{17}H_{25}NO$: 259.1936 [M], found: 259.1938

(*R)-1-((*R**)-6,6-Dimethyl-3-propylcyclohex-2-en-1-yl)-2-methylprop-2-en-1-ol (5l)**

(390 nm lamp) 58.5 mg of *N*-tosylhydrazone **1a**, 53 mg of propylboronic acid and 51.4 µL of methacrolein afforded 31 mg of **5l** (70% yield) as a colourless oil after column chromatography on SiO_2. Rf = 0.35 (Hex/EtOAc 10:1).

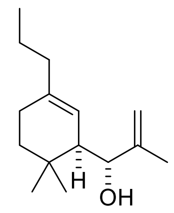

^{1}H NMR (300 MHz, $CDCl_3$) δ 4.95 (s, 1H), 4.93 – 4.85 (m, 2H), 3.92 (d, *J* = 9.2 Hz, 1H), 2.10 – 2.00 (m, 1H), 2.00 – 1.80 (m, 4H), 1.76 (s, 3H), 1.48 – 1.30 (m, 5H), 1.12 (s, 3H), 0.94 (s, 3H), 0.84 (t, *J* = 7.3 Hz, 3H).

^{13}C NMR (75 MHz, $CDCl_3$) δ 147.7 (C), 138.4 (C), 121.3 (CH), 113.6 (CH_2), 79.3 (CH), 47.8 (CH), 40.0 (CH_2), 38.1 (CH_2), 31.8 (C), 31.2 (CH_3), 25.8 (CH_2), 22.1 (CH_3), 21.0 (CH_2), 17.4 (CH_3), 13.9 (CH_3).

HRMS [ESI(+)]: m/z calcd. for $C_{15}H_{25}O$: 221.1900 [M-2H+H], found: 221.1899.

(*S,*E*)-1-((*R**)-6,6-Dimethyl-3-propylcyclohex-2-en-1-yl)-3-phenylprop-2-en-1-ol (5m)**

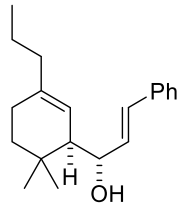

(390 nm lamp) 58.5 mg of *N*-tosylhydrazone **1a**, 53 mg of propylboronic acid and 51.7 µL of trans-cinnamaldehyde afforded 36.10 mg of **5m** (88% yield – 69% major diasteroisomer, 19% minor diasteroisomer) as a colourless oil after column chromatography on SiO_2. Rf = 0.18 (Hex/EtOAc 10:1).

<u>Major diasteroisomer:</u>

^{1}H NMR (300 MHz, $CDCl_3$) δ 7.42 – 7.29 (m, 4H), 7.29 – 7.21 (m, 1H), 6.57 (d, *J* = 15.9 Hz, 1H), 6.31 (dd, *J* = 15.9, 8.0 Hz, 1H), 5.44 (dt, *J* = 3.2, 1.6 Hz, 1H), 4.41 (dd, *J* = 8.0, 5.1 Hz, 1H), 2.29 – 2.19 (m, 1H), 2.05 – 1.80 (m, 4H), 1.53 – 1.25 (m, 4H), 1.06 (s, 3H), 0.96 – 0.85 (m, 6H).

^{13}C NMR (75 MHz, $CDCl_3$) δ 138.6 (C), 137.1 (C), 132.0 (CH), 131.6 (CH), 128.7 (CH), 127.8 (CH), 126.6 (CH), 120.2 (CH), 74.5 (CH), 51.2 (CH), 39.9 (CH_2), 36.5 (CH_2), 31.2 (C), 30.1 (CH_3), 25.7 (CH_2), 23.9 (CH_3), 21.1 (CH_2), 13.9 (CH_3).

<u>Minor diasteroisomer:</u>

^{1}H NMR (300 MHz, $CDCl_3$) δ 7.46 – 7.36 (m, 2H), 7.39 – 7.29 (m, 2H), 7.29 – 7.18 (m, 1H), 6.61 (dd, *J* = 15.9, 1.8 Hz, 1H), 6.31 (dd, *J* = 15.9, 4.7 Hz, 1H), 5.30 (td, *J* = 2.8, 1.5 Hz, 1H), 4.58 (ddt, *J* = 8.7, 4.4, 1.8 Hz, 1H), 2.07 – 1.92 (m, 5H), 1.70 (dt, *J* = 13.5, 6.9 Hz, 1H), 1.53 (d, *J* = 8.7 Hz, 1H), 1.44 (dt, *J* = 13.8, 7.1 Hz, 2H), 1.34 (dd, *J* = 12.8, 6.2 Hz, 1H), 1.06 (s, 3H), 0.99 (s, 3H), 0.88 (t, *J* = 7.3 Hz, 3H).

^{13}C NMR (75 MHz, $CDCl_3$) δ 142.9 (C), 137.2 (C), 133.9 (CH), 128.9 (CH), 128.7 (CH), 127.4 (CH), 126.5 (CH), 117.1 (CH), 71.1 (CH), 50.8 (CH), 40.2 (CH_2), 35.1 (CH_2), 31.6 (C), 29.2 (CH_3), 25.8 (CH_2), 25.6 (CH_3), 21.2 (CH_2), 13.9 (CH_3).

HRMS [APCI(+)]: m/z calcd. for $C_{20}H_{27}O$: 283.2056 [M-2H+H], found: 283.2043.

(*R)-(2-Bromophenyl)((*R**)-3,6,6-trimethylcyclohex-2-en-1-yl)methanol (5n)**

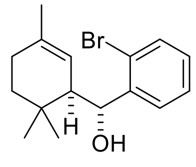

(390 nm lamp) 58.5 mg of *N*-tosylhydrazone **1a**, 36 mg of methylboronic acid and 64.2 µL of 2-bromobenzaldehyde afforded 52.6 mg of **5n** (85% yield) as a yellow oil after column chromatography on SiO_2. Rf = 0.32 (Hex/EtOAc 20:1).

^{1}H NMR (300 MHz, $CDCl_3$) δ 7.64 – 7.44 (m, 2H), 7.34 (t, *J* = 7.5 Hz, 1H), 7.12 (t, *J* = 7.7 Hz, 1H), 5.01 (d, *J* = 8.8 Hz, 1H), 4.51 (d, *J* = 3.4 Hz, 1H), 2.38 – 2.23 (m, 1H), 2.06 – 1.89 (m, 2H), 1.89 – 1.74 (m, 1H), 1.61 – 1.49 (m, 4H), 1.47 – 1.35 (m, 1H), 1.10 (s, 3H), 1.08 (s, 3H).

^{13}C NMR (75 MHz, $CDCl_3$) δ 143.6 (C), 135.0 (C), 132.7 (CH), 129.1 (CH), 128.8 (CH), 127.9 (CH), 123.8 (C), 120.9 (CH), 75.3 (CH), 51.7 (CH), 37.1 (CH_2), 32.1 (C), 30.3 (CH_3), 27.8 (CH_2), 23.8 (CH_3), 23.6 (CH_3).

HRMS [APCI(+)]: m/z calcd. for $C_{16}H_{20}Br$: 291.0743 [M-H_2O], found: 291.0733.

Ethyl 4-((*R)-3-((*R**)-hydroxy(p-tolyl)methyl)-4,4-dimethylcyclohex-1-en-1-yl)butanoate (5o)**

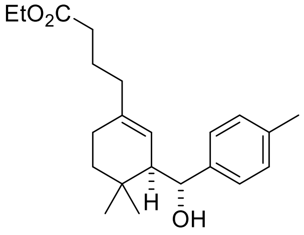

(390 nm lamp) 58.5 mg of *N*-tosylhydrazone **1a**, 96 mg of (4-ethoxy-4-oxobutyl)boronic acid and 47.3 µL of 4-methylbenzaldehyde afforded 47.8 mg of **5o** (71% yield) as a colourless oil after column chromatography on SiO_2. Rf = 0.29 (Hex/EtOAc 10:1).

^{1}H NMR (300 MHz, $CDCl_3$) δ 7.22 (d, *J* = 8.0 Hz, 2H), 7.13 (d, *J* = 8.0 Hz, 2H), 4.72 (dt, *J* = 3.1, 1.6 Hz, 1H), 4.46 (dd, *J* = 8.8, 2.9 Hz, 1H), 4.11 (d, *J* = 7.1 Hz, 1H), 4.07 (d, *J* = 7.1 Hz, 1H), 2.34 (s, 3H), 2.34 – 2.24 (m, 1H), 2.20 – 2.09 (m, 2H), 1.98 – 1.78 (m, 4H), 1.74 (d, *J* = 3.1 Hz, 1H), 1.67 – 1.54 (m, 2H), 1.42 – 1.28 (m, 2H), 1.23 (t, *J* = 7.1 Hz, 3H), 1.14 (s, 3H), 1.00 (s, 3H).

^{13}C NMR (75 MHz, $CDCl_3$) δ 173.9 (C), 141.6 (C), 137.3 (C), 137.2 (C), 129.1 (CH), 127.4 (CH), 122.5 (CH), 77.3 (CH), 60.3 (CH_2), 51.6 (CH), 37.4 (CH_2), 37.1 (CH_2), 33.8 (CH_2), 31.9 (C), 31.0 (CH_3), 25.6 (CH_2), 23.1 (CH_2), 22.8 (CH_3), 21.3 (CH_3), 14.4 (CH_3).

HRMS [EI (+)]: m/z calcd. for $C_{22}H_{30}O_2$: 326.2246 [M-H_2O], found: 326.2250.

(*R)-((*R**)-3-Cyclopentyl-6,6-dimethylcyclohex-2-en-1-yl)(p-tolyl)methanol (5p)**

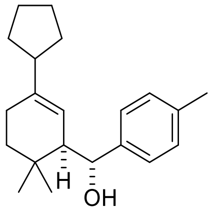

(390 nm lamp) 58.5 mg of *N*-tosylhydrazone **1a**, 68.4 mg of cyclopentylboronic acid and 47.3 µL of 4-methylbenzaldehyde afforded 41.4 mg of **5p** (70% yield) as a colourless oil after column chromatography on SiO_2. Rf = 0.37 (Hex/EtOAc 15:1).

^{1}H NMR (300 MHz, $CDCl_3$) δ 7.23 (d, *J* = 8.1 Hz, 2H), 7.13 (d, *J* = 1.9 Hz, 2H), 4.81 – 4.73 (m, 1H), 4.48 (d, *J* = 8.7 Hz, 1H), 2.35 (s, 3H), 2.33 – 2.26 (m, 1H), 2.26 – 2.13 (m, 1H), 2.01 – 1.75 (m, 2H), 1.70 (d, *J* = 2.5 Hz, 1H), 1.66 – 1.40 (m, 6H), 1.40 – 1.29 (m, 2H), 1.30 – 1.19 (m, 2H), 1.15 (s, 3H), 1.01 (s, 3H).

^{13}C NMR (75 MHz, $CDCl_3$) δ 141.8 (C), 141.1 (C), 137.2 (C), 129.0 (CH), 127.5 (CH), 119.6 (CH), 77.4 (CH), 51.6 (CH), 47.3 (CH), 37.4 (CH_2), 32.0 (C), 31.1 (CH_2), 31.1 (CH_3), 30.8 (CH_2), 25.2 (CH_2), 24.2 (CH_2), 22.8 (CH_3), 21.3 (CH_3).

HRMS [EI (+)]: m/z calcd. for $C_{21}H_{28}$: 280.2191 [M-H_2O], found: 280.2197.

(R^*)-(1H-Indol-7-yl)((R^*)-3,6,6-trimethylcyclohex-2-en-1-yl)methanol (5q)

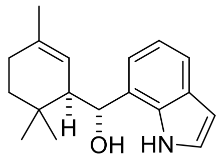

(390 nm lamp) 58.5 mg of *N*-tosylhydrazone **1a**, 36 mg of methylboronic acid and 59.9 mg of 1H-indole-7-carbaldehyde afforded 37 mg of **5q** (69% yield) as a brown oil after column chromatography on SiO_2. Rf = 0.3 (Hex/EtOAc 10:1).

^{1}H NMR (300 MHz, $CDCl_3$) δ 8.96 (bs, 1H), 7.60 (dt, *J* = 7.9, 1.0 Hz, 1H), 7.22 (t, *J* = 2.8 Hz, 1H), 7.06 (t, *J* = 7.5 Hz, 1H), 6.95 (dd, *J* = 7.2, 1.2 Hz, 1H), 6.57 (dd, *J* = 3.2, 2.1 Hz, 1H), 4.78 (d, *J* = 9.4 Hz, 1H), 4.63 (d, *J* = 3.1 Hz, 1H), 2.60 – 2.47 (m, 1H), 2.04 – 1.87 (m, 1H), 1.87 – 1.74 (m, 1H), 1.54 – 1.47 (m, 3H), 1.50 – 1.37 (m, 2H), 1.26 (s, 3H), 1.10 (s, 3H).

^{13}C NMR (75 MHz, $CDCl_3$) δ 134.6 (C), 134.0 (C), 128.9 (C), 126.8 (C), 124.3 (CH), 121.8 (CH), 121.0 (CH), 120.2 (CH), 119.1 (CH), 102.4 (CH), 78.1 (CH), 49.9 (CH), 37.9 (CH_2), 32.1 (C), 31.4 (CH_3), 27.7 (CH_2), 23.6 (CH_3), 22.3 (CH_3).

HRMS [EI (+)]: m/z calcd. for $C_{18}H_{23}NO$: 269.1774 [M], found: 269.1775.

(R^*)-(2-Bromophenyl)-(R^*)-(6,6-dimethyl-3-phenethylcyclohex-2-en-1-yl)methanol (5r)

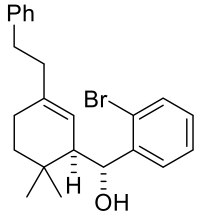

(390 nm lamp) 58.5 mg of *N*-tosylhydrazone **1a**, 90 mg of phenethylboronic acid and 46.3 µL of 2-bromobenzaldehyde afforded 39 mg of **5r** (50% yield) as a brown oil after column chromatography on SiO_2. Rf = 0.35 (Hex/EtOAc 20:1).

^{1}H NMR (300 MHz, $CDCl_3$) δ 7.59 – 7.47 (m, 2H), 7.40 – 7.06 (m, 7H), 5.00 (dd, *J* = 8.6, 2.9 Hz, 1H), 4.58 (dt, *J* = 3.4, 1.7 Hz, 1H), 2.57 (ddt, *J* = 11.9, 8.5, 4.5 Hz, 2H), 2.38 – 2.28 (m, 1H), 2.21 – 2.07 (m, 2H), 2.06 – 1.98 (m, 1H), 1.95 – 1.80 (m, 2H), 1.57 (dt, *J* = 13.2, 5.7 Hz, 1H), 1.43 (ddd, *J* = 13.5, 8.0, 6.0 Hz, 1H), 1.10 (s, 3H), 1.08 (s, 3H).

^{13}C NMR (75 MHz, $CDCl_3$) δ 143.6 (C), 142.6 (C), 138.1 (C), 132.6 (CH), 129.1 (CH), 128.9 (CH), 128.4 (CH), 128.3 (CH), 127.9 (CH), 125.8 (CH), 123.7 (C), 121.2 (CH), 75.4 (CH), 51.7 (CH), 39.6 (CH_2), 37.0 (CH_2), 34.4 (CH_2), 32.2 (C), 30.3 (CH_3), 26.2 (CH_2), 23.8 (CH_3).

HRMS [EI (+)]: m/z calcd. for $C_{23}H_{25}Br$: 380.1140 [M-H_2O], found: 380.1136.

4-((R^*)-((S^*)-1,3-Dimethylcyclohex-2-en-1-yl)(hydroxy)methyl)benzonitrile (7a)

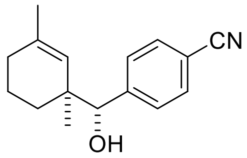

(390 nm lamp) 55.7 mg of *N*-tosylhydrazone **1d**, 36 mg of methylboronic acid and 53.5 mg of 4-formylbenzonitrile afforded 34.2 mg of **7a** (71% yield) as a colourless oil.

^{1}H NMR (300 MHz, $CDCl_3$) δ 7.59 (d, *J* = 8.4 Hz, 2H), 7.43 (d, *J* = 8.4 Hz, 2H), 5.06 (t, *J* = 1.5 Hz, 1H), 4.47 (s, 1H), 2.17 (bs, 1H), 1.74 – 1.68 (m, 2H), 1.69 (s, 3H), 1.68 – 1.46 (m, 3H), 1.04 (ddt, *J* = 13.0, 6.6, 2.9 Hz, 1H), 0.88 (s, 3H).

^{13}C NMR (75 MHz, $CDCl_3$) δ 146.2 (C), 138.8 (C), 131.4 (CH), 128.8 (CH), 126.9 (CH), 119.1 (C), 111.1 (C), 80.4 (CH), 41.2 (C), 30.1 (CH_2), 29.0 (CH_2), 24.3 (CH_3), 24.0 (CH_2), 19.2 (CH_3).

HRMS [ESI (+)]: m/z calcd. for $C_{16}H_{20}NO$: 242.1539 [M+H], found: 242.1540.

4-((*R)-((*S**)-3-(4-Cyanobutyl)-1-methylcyclohex-2-en-1-yl)(hydroxy)methyl)benzonitrile (7b)**

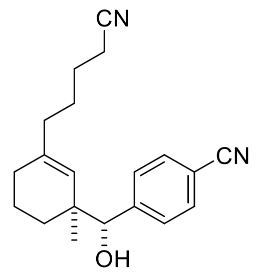

(390 nm lamp) 55.7 mg of *N*-tosylhydrazone **1d**, 76.2 mg of (4-cyanobutyl)boronic acid and 53.5 mg of 4-formylbenzonitrile afforded 46.8 mg of **7b** (76% yield) as a colourless oil.

H NMR (300 MHz, $CDCl_3$) δ 7.61 (d, J = 8.3 Hz, 2H), 7.44 (d, J = 8.2 Hz, 2H), 5.09 (s, 1H), 4.49 (s, 1H), 2.37 (t, J = 6.6 Hz, 2H), 2.03 (t, J = 7.1 Hz, 2H), 1.95 – 1.85 (m, 2H), 1.77 – 1.47 (m, 8H), 1.09 (dq, *J* = 13.1, 2.9 Hz, 1H), 0.90 (s, 3H).

^{13}C NMR (75 MHz, $CDCl_3$) δ 146.2 (C), 141.1 (C), 131.4 (CH), 128.8 (CH), 127.5 (CH), 119.8 (C), 119.1 (C), 111.1 (C), 80.4 (CH), 41.1 (C), 37.3 (CH_2), 29.5 (CH_2), 28.3 (CH_2), 26.8 (CH_2), 25.1 (CH_2), 24.1 (CH_3), 19.3 (CH_2), 17.2 (CH_2).

HRMS [ESI (+)]: m/z calcd. for $C_{20}H_{24}NO$+Na: 331.1781 [M+Na], found: 331.1767.

(*S)-3-Methyl-1-((*S**)-1-methyl-3-propylcyclohex-2-en-1-yl)but-2-en-1-ol (7c)**

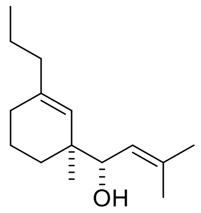

(390 nm lamp) 55.7 mg of *N*-tosylhydrazone **1d**, 53 mg of propylboronic acid and 39 μL of 3-methylbut-2-enal afforded 46.8 mg of **7c** (58% yield) as a colourless oil.

^1H NMR (300 MHz, $CDCl_3$) δ 5.19 (dp, *J* = 9.3, 1.5 Hz, 1H), 5.08 (d, *J* = 2.5 Hz, 1H), 4.04 (d, *J* = 9.3 Hz, 1H), 1.93 (t, *J* = 7.5 Hz, 2H), 1.84 (dd, *J* = 5.7, 3.2 Hz, 2H), 1.74 (d, *J* = 1.4 Hz, 3H), 1.72 – 1.50 (m, 7H), 1.48 – 1.36 (m, 2H), 1.38 – 1.22 (m, 2H), 0.89 (s, 3H), 0.85 (t, *J* = 7.3 Hz, 3H).

^{13}C NMR (75 MHz, $CDCl_3$) δ 140.9 (C), 136.5 (C), 127.3 (CH), 123.9 (CH), 75.2 (CH), 40.8 (C), 40.4 (CH_2), 29.6 (CH_2), 28.5 (CH_2), 26.3 (CH_3), 23.8 (CH_3), 20.9 (CH_2), 19.6 (CH_2), 18.7 (CH_3), 13.8 (CH_3).

HRMS [EI (+)]: m/z calcd. for $C_{15}H_{26}O$: 222.1978 [M], found: 222.1982.

(*R)-((*S**)-1-Methyl-3-phenethylcyclopent-2-en-1-yl)(p-tolyl)methanol (8a)**

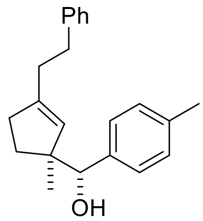

(390 nm lamp) 52.9 mg of *N*-tosylhydrazone **1h**, 90 mg of phenethylboronic acid and 47.3 μL of 4-methylbenzaldehyde afforded 30.5 mg of **8a** (50% yield) as a colourless oil after column chromatography on SiO_2. Rf = 0.3 (Hex/EtOAc 10:1).

^1H NMR (300 MHz, $CDCl_3$) δ 7.37 – 7.05 (m, 9H), 5.07 (s, 1H), 4.45 (s, 1H), 2.85 – 2.72 (m, 2H), 2.47 – 2.37 (m, 2H), 2.35 (s, 3H), 2.31 – 2.13 (m, 3H), 1.48 – 1.33 (m, 1H), 0.97 (s, 3H).

^{13}C NMR (75 MHz, $CDCl_3$) δ 145.6 (C), 142.2 (C), 138.7 (C), 136.9 (C), 131.0 (CH), 128.5 (CH), 128.4 (CH), 127.5 (CH), 126.0 (CH), 79.8 (CH), 54.6 (C), 34.6 (CH_2), 34.1 (CH_2), 33.1 (CH_2), 32.4 (CH_2), 23.2 (CH_3), 21.3 (CH_3).

HRMS [EI (+)]: m/z calcd. for $C_{22}H_{24}$: 288.1873 [M-H_2O], found: 288.1878.

(R^*)-((S^*)-3-(but-3-en-1-yl)-1-methylcyclopent-2-en-1-yl)(p-tolyl)methanol (8b)

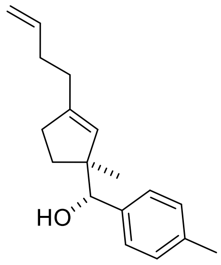

(390 nm lamp) 52.9 mg of *N*-tosylhydrazone **1h**, 60 mg of 3-butenylboronic acid and 47.3 μL of 4-methylbenzaldehyde afforded 34.5 mg of **8b** (68% yield) as a colourless oil after column chromatography on SiO_2. Rf = 0.3 (Hex/EtOAc 10:1).

^1H NMR (300 MHz, $CDCl_3$) δ 7.23 (d, *J* = 8.0 Hz, 2H), 7.12 (d, *J* = 8.0 Hz, 2H), 5.82 (ddt, *J* = 16.7, 10.1, 6.2 Hz, 1H), 5.11 (s, 1H), 5.09 – 4.94 (m, 2H), 4.50 (s, 1H), 2.35 (s, 3H), 2.28 – 2.11 (m, 7H), 1.47 – 1.33 (m, 1H), 0.99 (s, 3H).

^{13}C NMR (75 MHz, $CDCl_3$) δ 145.9 (C), 138.7 (C), 138.6 (CH), 136.9 (C), 130.6 (CH), 128.4 (CH), 127.6 (CH), 114.8 (CH2), 79.8 (CH), 54.7 (C), 34.6 (CH_2), 32.3 (CH_2), 32.0 (CH_2), 30.7 (CH_2), 23.4 (CH_3), 21.3 (CH_3).

HRMS [EI (+)]: m/z calcd. for $C_{18}H_{22}$: 238.1716 [M-H_2O], found: 238.1716

(S^*,*E*)-1-((S^*)-3-Cyclopropyl-1-methylcyclopent-2-en-1-yl)-3-phenylprop-2-en-1-ol (8c)

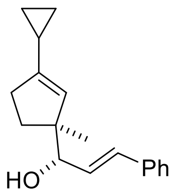

(390 nm lamp) 52.9 mg of *N*-tosylhydrazone **1h**, 51.6 mg of cyclopropylboronic acid and 51.7 μL of trans-cinnamaldehyde afforded 40 mg of **8c** (79% yield) as a colourless oil.

^1H NMR (300 MHz, $CDCl_3$) δ 7.44 – 7.36 (m, 2H), 7.32 (t, *J* = 7.4 Hz, 2H), 7.25 (d, *J* = 6.9 Hz, 1H), 6.62 (d, *J* = 15.9 Hz, 1H), 6.23 (dd, *J* = 15.9, 7.1 Hz, 1H), 5.21 (s, 1H), 4.06 (d, *J* = 7.3 Hz, 1H), 2.25 – 2.09 (m, 2H), 2.14 – 2.00 (m, 1H), 1.87 (s, 1H), 1.58 – 1.43 (m, 2H), 1.07 (s, 3H), 0.73 – 0.59 (m, 2H), 0.62 – 0.46 (m, 2H).

^{13}C NMR (75 MHz, $CDCl_3$) δ 148.5 (C), 137.1 (C), 132.0 (CH), 129.2 (CH), 128.6 (CH), 128.0 (CH), 127.6 (CH), 126.6 (CH), 78.9 (CH), 54.2 (C), 31.9 (CH_2), 31.8 (CH_2), 23.6 (CH_3), 12.3 (CH_3), 5.9 (CH_2).

HRMS [EI (+)]: m/z calcd. for $C_{18}H_{20}$: 236.1560 [M-H_2O], found: 236.1562

(S^*,*E*)-1-((S^*)-1-Methyl-3-propylcyclopent-2-en-1-yl)-3-phenylprop-2-en-1-ol (8d)

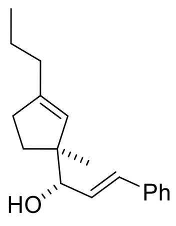

(390 nm lamp) 52.9 mg of *N*-tosylhydrazone **1h**, 53 mg of propylboronic acid and 51.7 μL of trans-cinnamaldehyde afforded 46.2 mg of **8d** (91% yield) as a colourless oil after column chromatography on SiO_2. Rf = 0.32 (Hex/EtOAc 8:1).

^1H NMR (300 MHz, $CDCl_3$) δ 7.48 – 7.37 (m, 2H), 7.38 – 7.29 (m, 2H), 7.29 – 7.21 (m, 1H), 6.61 (d, *J* = 15.9 Hz, 1H), 6.26 (dd, *J* = 15.9, 7.1 Hz, 1H), 5.18 (s, 1H), 4.09 (d, *J* = 7.1 Hz, 1H), 2.32 (ddd, *J* = 8.8, 5.3, 1.7 Hz, 2H), 2.18 – 2.03 (m, 3H), 1.93 (s, 1H), 1.62 – 1.42 (m, 3H), 1.10 (s, 3H), 0.94 (t, *J* = 7.3 Hz, 3H).

^{13}C NMR (75 MHz, $CDCl_3$) δ 147.1 (C), 137.1 (C), 131.9 (CH), 129.5 (CH), 129.3 (CH), 128.6 (CH), 127.5 (CH), 126.5 (CH), 78.8 (CH), 54.2 (C), 34.6 (CH_2), 33.4 (CH_2), 32.3 (CH_2), 23.6 (CH_3), 21.0 (CH_2), 14.0 (CH_3).

HRMS [EI (+)]: m/z calcd. for $C_{18}H_{22}$: 238.1722 [M-H_2O], found: 238.1728

4-((*R)-Hydroxy((*S**)-1-Methyl-3-phenethylcyclopent-2-en-1-yl)methyl)benzonitrile (8e)**

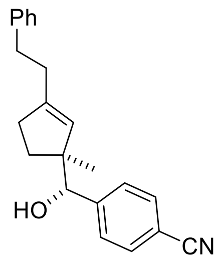

(390 nm lamp) 52.9 mg of *N*-tosylhydrazone **1h**, 90 mg of phenethylboronic acid and 53.5 mg of 4-formylbenzonitrile afforded 46 mg of **8e** (73% yield) as a colourless oil after column chromatography on SiO_2. Rf = 0.35 (Hex/EtOAc 20:1)

^{1}H NMR (300 MHz, $CDCl_3$) δ 7.55 (d, J = 8.3 Hz, 2H), 7.40 – 7.09 (m, 7H), 4.97 (s, 1H), 4.46 (s, 1H), 2.79 (td, J = 7.3, 4.2 Hz, 2H), 2.42 (t, J = 7.6 Hz, 2H), 2.32 – 2.21 (m, 2H), 2.18 – 1.96 (m, 2H), 1.38 (ddd, J = 12.9, 8.3, 6.0 Hz, 1H), 0.94 (s, 3H).

^{13}C NMR (75 MHz, $CDCl_3$) δ 147.0 (C), 146.6 (C), 141.8 (C), 131.4 (CH), 130.2 (CH), 128.5 (CH), 128.4 (CH), 128.3 (CH), 126.1 (CH), 119.1 (C), 110.9 (C), 79.1 (CH), 54.6 (C), 34.5 (CH_2), 33.9 (CH_2), 32.9 (CH_2), 32.3 (CH_2), 22.9 (CH_3).

HRMS [ESI (+)]: m/z calcd. for $C_{22}H_{24}NO$: 318.1852 [M-H_2O], found: 318.1833.

4-((*R)-((*S**)-3-(But-3-en-1-yl)-1-methylcyclopent-2-en-1-yl)(hydroxy)methyl)benzonitrile (8f)**

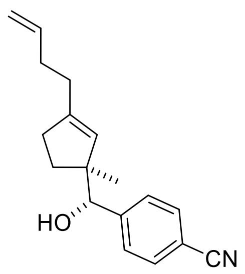

(390 nm lamp) 52.9 mg of *N*-tosylhydrazone **1h**, 60 mg of 3-butenylboronic acid and 53.5 mg of 4-formylbenzonitrile afforded 34.8 mg of **8f** (65% yield) as a colourless oil after column chromatography on SiO_2. Rf = 0.40 (Hex/EtOAc 20:1)

^{1}H NMR (300 MHz, $CDCl_3$) 7.59 (d, J = 8.4 Hz, 2H), 7.45 (d, J = 8.3 Hz, 2H), 5.80 (tdd, J = 15.7, 8.1, 4.9 Hz, 1H), 5.14 – 4.89 (m, 3H), 4.55 (s, 1H), 2.34 – 1.92 (m, 8H), 1.43 – 1.29 (m, 1H), 0.97 (s, 3H).

^{13}C NMR (75 MHz, $CDCl_3$) δ 147.2 (C), 147.0 (C), 138.4 (CH), 131.5 (CH), 129.7 (CH), 128.4 (CH), 119.1 (C), 115.0 (CH), 111.1 (C), 79.2 (CH), 54.7 (C), 34.6 (CH_2), 32.0 (CH_2), 31.9 (CH_2), 30.6 (CH_2), 23.4 (CH_3).

HRMS [EI (+)]: m/z calcd. for $C_{18}H_{21}N$: 249.1517 [M-H_2O], found: 249.1506.

Ethyl 4-((S*)-3-((R*)-Hydroxy(4-nitrophenyl)methyl)-3-methylcyclopent-1-en-1-yl)butanoate (8g)

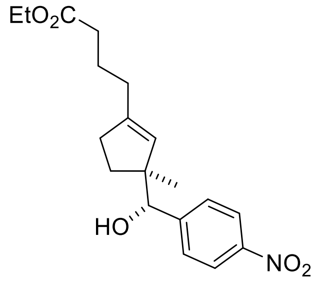

(390 nm lamp) 52.9 mg of *N*-tosylhydrazone **1h**, 96 mg of (4-ethoxy-4-oxobutyl)boronic acid and 61.7 mg of 4-nitrobenzaldehyde afforded 41.6 mg of **8g** (60% yield) as a yellow oil after column chromatography on SiO_2. Rf = 0.3 (Hex/EtOAc 3:1).

^{1}H NMR (300 MHz, $CDCl_3$) δ 8.15 (d, *J* = 8.7 Hz, 2H), 7.50 (d, *J* = 8.7 Hz, 2H), 5.15 – 5.04 (m, 1H), 4.62 (s, 1H), 4.12 (q, *J* = 7.1 Hz, 2H), 2.36 – 2.23 (m, 3H), 2.17 (dd, *J* = 13.1, 2.3 Hz, 1H), 2.14 – 2.04 (m, 3H), 1.83 – 1.69 (m, 2H), 1.45 – 1.33 (m, 1H), 1.25 (t, *J* = 7.1 Hz, 3H), 0.99 (s, 3H).

^{13}C NMR (75 MHz, $CDCl_3$) δ 173.7 (C), 149.2 (C), 147.3 (C), 146.8 (C), 129.9 (CH), 128.5 (CH), 122.9 (CH), 79.2 (CH), 60.5 (CH_2), 54.7 (C), 34.4 (CH_2), 34.0 (CH_2), 32.3 (CH_2), 30.6 (CH_2), 23.3 (CH_3), 23.0 (CH_2), 14.4 (CH_3).

HRMS [APCI(+)]: m/z calcd. for $C_{16}H_{26}NO_5$: 348.1805 [M+H], found: 348.1800.

(*R)-((*S**)-3-Methylcyclohex-2-en-1-yl)(thiophen-2-yl)methanol (9a)**

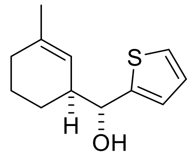

(390 nm lamp) 79.3 mg of *N*-tosylhydrazone **1e**, 36 mg of methylboronic acid and 37.4 μL of thiophene-2-carbaldehyde afforded 26.5 mg of **9a** (64% yield) as a colourless oil after column chromatography on SiO_2. Rf = 0.25 (Hex/EtOAc 20:1)

^{1}H NMR (300 MHz, $CDCl_3$) δ 7.32 – 7.19 (m, 1H), 6.98 (d, J = 3.9 Hz, 2H), 5.17 (s, 1H), 4.78 (dd, J = 6.8, 1.9 Hz, 1H), 2.53 (dtq, J = 9.3, 4.7, 2.5 Hz, 1H), 2.05 (d, J = 2.5 Hz, 1H), 1.96 – 1.85 (m, 42H), 1.85 – 1.70 (m, 2H), 1.65 (s, 3H), 1.60 – 1.33 (m, 2H).

^{13}C NMR (75 MHz, $CDCl_3$) δ 147.0 (C), 138.2 (C), 126.6 (CH), 124.7 (CH), 124.6 (CH), 121.5 (CH), 74.0 (CH), 43.9 (CH), 30.3 (CH_2), 24.3 (CH_2), 24.2 (CH_3), 21.6 (CH_2).

HRMS [EI (+)]: m/z calcd. for $C_{12}H_{14}S$: 190.0816 [M-H_2O], found: 190.0821.

(*R)-Benzofuran-2-yl-((*S**)-3-methylcyclohex-2-en-1-yl)methanol (9b)**

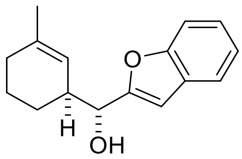

(390 nm lamp) 79.3 mg of *N*-tosylhydrazone **1e**, 36 mg of methylboronic acid and 49.5 μL of benzofuran-2-carbaldehyde afforded 28 mg of **9b** (58% yield) as a colourless oil after column chromatography on SiO_2. Rf = 0.15 (Hex/EtOAc 20:1)

^{1}H NMR (300 MHz, $CDCl_3$) δ 7.55 (dd, J = 6.9, 1.9 Hz, 1H), 7.47 (d, J = 7.8 Hz, 1H), 7.33 – 7.13 (m, 2H), 6.65 (s, 1H), 5.17 (s, 1H), 4.68 (dd, J = 6.9, 4.3 Hz, 1H), 2.76 (bs, 1H), 2.09 (d, J = 4.4 Hz, 1H), 1.97 – 1.71 (m, 4H), 1.66 (s, 3H), 1.60 – 1.40 (m, 2H).

^{13}C NMR (75 MHz, $CDCl_3$) δ 158.5 (C), 154.8 (C), 138.3 (C), 128.3 (C), 124.1 (CH), 122.8 (CH), 121.2 (CH), 121.1 (CH), 111.3 (CH), 103.6 (CH), 72.0 (CH), 40.9 (CH), 30.2 (CH_2), 24.2 (CH_2), 23.8 (CH_3), 21.5 (CH_2).

HRMS [EI (+)]: m/z calcd. for $C_{16}H_{16}O$: 224.1201 [M-H_2O], found: 224.1196.

(*R)-((*S**)-3-Methylcyclohex-2-en-1-yl)(4-nitrophenyl)methanol (9c)**

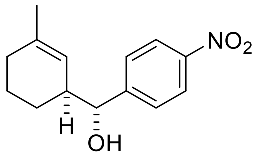

(390 nm lamp) 79.3 mg of *N*-tosylhydrazone **1e**, 36 mg of methylboronic acid and 61.7 mg of 4-nitrobenzaldehyde afforded 25.5 mg of **9c** (52% yield) as a yellow oil after column chromatography on SiO_2. Rf = 0.38 (3:1 Hex/EtOAc).

^{1}H NMR (300 MHz, $CDCl_3$) δ 8.16 (d, *J* = 8.7 Hz, 2H), 7.49 (d, *J* = 8.7 Hz, 2H), 5.14 (s, 1H), 4.73 (d, *J* = 5.3 Hz, 1H), 2.55 – 2.40 (m, 1H), 2.33 – 2.14 (m, 1H), 1.99 – 1.80 (m, 2H), 1.79 – 1.68 (m, 1H), 1.66 (s, 3H), 1.53 – 1.27 (m, 3H).

^{13}C NMR (75 MHz, $CDCl_3$) δ 150.5 (C), 147.1 (C), 139.7 (C), 127.2 (CH), 123.4 (CH), 121.2 (CH), 76.3 (CH), 43.5 (CH), 30.1 (CH_2), 24.2 (CH_3), 22.6 (CH_2), 21.5 (CH_2).

HRMS [EI (+)]: m/z calcd. for $C_{14}H_{15}NO_2$: 229.1103 [M-H_2O], found: 229.1096.

(*R)-Benzofuran-2-yl((*S**)-3-cyclopropylcyclohept-2-en-1-yl)methanol (10)**

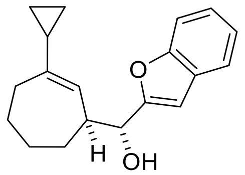

(370 nm lamp) 55.7 mg of *N*-tosylhydrazone **1g**, 51.6 mg of cyclopropylboronic acid and 49.5 µL of benzofuran-2-carbaldehyde afforded 21 mg of **10** (37% yield) as a colourless oil after column chromatography on SiO_2. Rf = 0.26 (8:1 Hex/EtOAc).

^1H NMR (300 MHz, $CDCl_3$) δ 7.55 (d, *J* = 7.2 Hz, 1H), 7.46 (d, *J* = 7.9 Hz, 1H), 7.33 – 7.15 (m, 2H), 6.66 (s, 1H), 5.52 (d, *J* = 4.8 Hz, 1H), 4.84 – 4.70 (m, 1H), 2.87 (dt, *J* = 11.3, 5.8 Hz, 1H), 2.15 – 1.95 (m, 3H), 1.90 (dt, *J* = 13.0, 4.0 Hz, 1H), 1.79 (dd, *J* = 14.2, 7.2 Hz, 1H), 1.76 – 1.63 (m, 2H), 1.63 – 1.54 (m, 1H), 1.34 (ddt, *J* = 10.1, 7.0, 3.9 Hz, 1H), 1.29 – 1.07 (m, 2H), 0.60 – 0.46 (m, 2H), 0.41 – 0.28 (m, 2H).

^{13}C NMR (75 MHz, $CDCl_3$) δ 158.8 (C), 154.8 (C), 145.4 (C), 128.3 (C), 125.3 (CH), 124.1 (CH), 122.8 (CH), 121.1 (CH), 111.4 (CH), 103.8 (CH), 72.4 (CH), 44.2 (CH), 31.3 (CH_2), 29.2 (CH_2), 28.6 (CH_2), 26.4 (CH_2), 18.7 (CH_3), 4.1 (CH_2), 4.1 (CH_2)

HRMS [EI (+)]: m/z calcd. for $C_{19}H_{20}O$: 264.1514 [M-H_2O], found: 264.1510.

Benzyl (*S)-6-((*S**)-(2-bromophenyl)(hydroxy)methyl)-4-methyl-3,6-dihydropyridine-1(2H)-carboxylate (11a)**

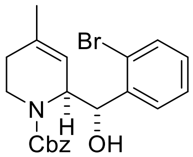

(390 nm lamp) 79.9 mg of *N*-tosylhydrazone **1f**, 36 mg of methylboronic acid and 46.3 µL of 2-bromobenzaldehyde afforded 43.1 mg of **11a** (52% yield) as a colourless oil after column chromatography on SiO_2. Rf = 0.2 (3:1 Hex/EtOAc).

The compound is present in the NMR spectra as a mixture of rotamers.

1H NMR (401 MHz, CDCl3) δ 7.72 – 7.01 (m, 9H), 5.32 – 5.07 (m, 2H + *min. rot.*), 5.05 – 4.89 (m, 1H), 4.77 (s, 1H), 4.63 - 4.57 (m, *min. rot.*), 4.38 – 4.06 (m, 1H), 3.73 (s, *min. rot.*), 3.32 – 3.02 (m, 1H), 2.47 (s, *min. rot.*), 2.28 – 2.07 (m, 1H), 1.91 – 1.59 (m, 4H), 1.27 (s, *min. rot.*).

^{13}C NMR (101 MHz, $CDCl_3$) δ 157.3 (C), 155.7 (C), 140.6 (C), 140.0 (C), 137.1 (C), 136.6 (C), 135.6 (C), 132.7 (CH), 132.5 (CH), 129.2 (CH), 128.9 (CH), 128.6 (CH), 128.5 (CH), 128.2 (CH), 128.1 (CH), 127.7 (CH), 127.5 (CH), 123.4 (C), 122.9 (C), 118.1 (CH), 117.8 (CH), 75.1 (CH), 74.8 (CH), 67.6 (CH_2), 67.3 (CH_2), 58.3 (CH), 56.1 (CH), 38.6 (CH_2), 29.6 (CH_2), 29.2 (CH_2), 23.5 (CH_3).

HRMS [EI (+)]: m/z calcd. for $C_{14}H_{14}BrNO_2$: 307.0208 [M-$PhCH_2OH$], found: 307.0204.

Benzyl (*S)-6-((*S**)-(4-cyanophenyl)(hydroxy)methyl)-4-propyl-3,6-dihydropyridine-1(2H)-carboxylate (11b)**

(390 nm lamp) 79.9 mg of *N*-tosylhydrazone **1f**, 53 mg of propylboronic acid and 53.5 mg of 4-formylbenzonitrile afforded 35 mg of **11b** (45% yield) as a colourless oil after column chromatography on SiO_2. Rf = 0.3 (2:1 Hex/EtOAc).

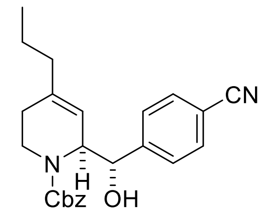

The compound is present in the NMR spectra as a mixture of rotamers.

^{1}H NMR (300 MHz, $CDCl_3$) δ 7.97 – 7.29 (m, 9H), 5.27 – 4.95 (m, 2H + *min. rot.*), 4.88 (s, *min. rot.*), 4.75 (m, 1H + *min. rot.*), 4.64 (s, 1H), 4.39 – 4.05 (m, 1H + *min. rot.*), 4.01 – 3.73 (m, *min. rot.*), 2.90 (m, 1H), 2.66 (s, *min. rot.*), 2.22 – 2.07 (m, 1H), 2.01 – 1.69 (m, 3H + *min. rot.*), 1.50 – 1.22 (m, 2H + *min. rot.*), 0.82 (t, J = 7.4 Hz, 3H).

^{13}C NMR (75 MHz, $CDCl_3$) δ 157.5 (C), 146.9 (C), 140.2 (C), 136.4 (CH), 132.1 (CH), 128.7 (CH), 128.4 (CH), 128.1 (CH), 128.0 (CH), 118.9 (CH), 117.0 (CH), 111.7 (CH), 76.8 (CH), 67.8 (CH_2), 58.5 (CH), 57.9 (CH), 39.5 (CH_2), 38.5 (CH_2), 27.8 (CH_2), 27.4 (CH_2), 20.5 (CH_2), 13.8 (CH_3).

HRMS [ESI (+)]: m/z calcd. for $C_{24}H_{27}N_2O_3$: 391.2016 [M+H], found: 391.2024.

Benzyl (*S)-6-((*S*,E*)-1-Hydroxy-3-phenylallyl)-4-phenethyl-3,6-dihydropyridine-1(2H)-carboxylate (11c)**

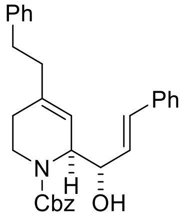

(390 nm lamp) 79.9 mg of *N*-tosylhydrazone **1f**, 90 mg of phenethylboronic acid and 51.7 µL of trans-cinnamaldehyde afforded 36.3 mg of **11c** (40% yield) as a yellow oil after column chromatography on SiO_2. Rf = 0.2 (4:1 Hex/EtOAc).

The compound is present in the NMR spectra as a 2.0:1 mixture of rotamers.

^{1}H NMR (300 MHz, $CDCl_3$) δ 7.49 – 7.07 (m, 15H), 6.66 – 6.50 (m, 1H), 6.25 – 5.99 (m, 1H), 5.39 (s, *min. rot.*), 5.19 (s, 2H), 4.55 (s, *min. rot.*), 4.34 – 4.13 (m, 2H), 3.09 (s, 1H + *min. rot.*), 2.75 (t, J = 7.6 Hz, 2H), 2.44 – 2.08 (m, 2H + *min. rot.*), 1.99 – 1.85 (m, 1H + *min. rot.*).

^{13}C NMR (75 MHz, $CDCl_3$) δ 157.0 (C), 156.9 (C), 141.6 (CH), 139.1 (C), 138.4 (C), 136.6 (C), 136.5 (CH), 132.5 (C), 132.0 (C), 129.1 (C), 129.1 (C), 128.6 (CH), 128.6 (CH), 128.5 (CH), 128.2 (CH), 128.0 (CH), 127.8 (CH), 126.7 (CH), 126.1 (CH), 119.0 (CH), 118.6 (CH), 75.1 (CH), 74.6 (CH), 67.5 (CH_2), 57.6 (CH), 57.1 (CH), 39.3 (CH_2), 39.1 (CH_2), 38.5 (CH_2), 34.0 (CH_2), 28.1 (CH_2).

HRMS [ESI (+)]: m/z calcd. for $C_{30}H_{32}NO_3$: 454.2377 [M+H], found: 454.2381.

Benzyl (*S)-6-((*S**)-1-hydroxy-2-methylallyl)-4-methyl-3,6-dihydropyridine-1(2H)-carboxylate (11d)**

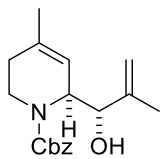

(390 nm lamp) 79.9 mg of *N*-tosylhydrazone **1f**, 36 mg of methylboronic acid and 34.8 µL of methacrolein afforded 23 mg of **11d** (38% yield) as a white solid after column chromatography on SiO_2. Rf = 0.3 (2:1 Hex/EtOAc).

^{1}H NMR (300 MHz, $CDCl_3$) δ 7.48 – 7.27 (m, 5H), 5.22 (s, 1H), 5.20 – 5.08 (m, 2H), 4.96 (s, 1H), 4.93 (s, 1H), 4.63 – 4.42 (m, 1H), 4.33 – 4.11 (m, 1H), 4.02 (d, *J* = 8.5 Hz, 1H), 3.30 (s, *min. rot.*), 3.09 (dt, *J* = 13.5, 6.7 Hz, 1H), 2.31 – 2.08 (m, 1H + *min. rot.*), 1.93 – 1.63 (m, 7H + *min. rot.*), 1.34 – 1.15 (m, *min. rot.*).

^{13}C NMR (75 MHz, $CDCl_3$) δ 157.6 (C), 144.6 (C), 144.1 (C), 136.6 (C), 135.0 (C), 128.6 (CH), 128.2 (CH), 128.0 (CH), 118.5 (CH), 114.3 (CH_2), 78.7 (CH), 77.4 (CH), 67.6 (CH_2), 55.6 (CH), 55.2 (CH), 38.2 (CH_2), 29.7 (CH_2), 29.3 (CH_2), 23.6 (CH_3), 18.0 (CH_3), 17.4 (CH_3).

HRMS [EI (+)]: m/z calcd. for $C_{11}H_{15}NO_2$: 193.1103 [M-$PhCH_2OH$], found: 193.1101.

m.p. = 68.9 – 70 ºC

Benzyl (*S)-6-((*S**)-hydroxy(phenyl)methyl)-4-methyl-3,6-dihydropyridine-1(2H)-carboxylate (11e)**

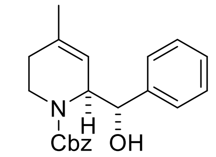

(390 nm lamp) 79.9 mg of *N*-tosylhydrazone **1f**, 36 mg of methylboronic acid and 40.9 µL of benzaldehyde afforded 35.5 mg of **11e** (53% yield) as a colourless oil after column chromatography on SiO_2. Rf = 0.3 (Hex/EtOAc 3:1)

The compound is present in the NMR spectra as a mixture of rotamers.

^{1}H NMR (300 MHz, $CDCl_3$) δ 7.40 – 7.28 (m, 5H), 5.30 – 4.85 (m, 1H + *min. rot.*), 4.73 – 4.49 (m, 1H), 4.34 – 4.03 (m, *min. rot.*), 3.68 (s, *min. rot.*), 3.08 – 2.82 (m, *min. rot.*), 2.57 (s, *min. rot.*), 2.16 (s, *min. rot.*), 1.90 – 1.54 (m, 2H + *min. rot.*).

^{13}C NMR (75 MHz, $CDCl_3$) δ 157.5 (C), 141.5 (C), 140.9 (C), 136.6 (C), 135.1 (C), 128.6 (CH), 128.4 (CH), 128.2 (CH), 128.0 (CH), 127.2 (CH), 118.3 (C), 118.0 (C), 75.9 (CH), 67.6 (CH_2), 58.6 (CH), 58.1 (CH), 38.2 (CH_2), 29.6 (CH_2), 23.5 (CH_3).

HRMS [ESI (+)]: m/z calcd. for $C_{21}H_{24}NO_3$: 338.1751 [M+H], found: 338.1742.

Benzyl (*S)-6-((*S**)-(4-cyanophenyl)(hydroxy)methyl)-4-methyl-3,6-dihydropyridine-1(2H)-carboxylate (11f)**

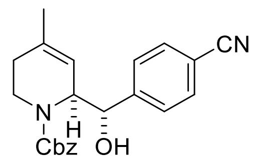

(390 nm lamp) 79.9 mg of *N*-tosylhydrazone **1f**, 36 mg of methylboronic acid and 53.5 mg of 4-formylbenzonitrile afforded 38 mg of **11f** (53% yield) as a colourless oil after column chromatography on SiO_2. Rf = 0.45 (2:1 Hex/EtOAc).

The compound is present in the NMR spectra as a mixture of rotamers.

^{1}H NMR (300 MHz, $CDCl_3$) δ 7.73 – 7.29 (m, 9H), 5.24 – 4.86 (m, 1H+ *min. rot.*), 4.77 – 4.69 (m, 1H + *min. rot.*), 4.62 (s, 1H), 4.33 – 4.06 (m, 1H + *min. rot.*), 3.88 (s, *min. rot.*), 3.07 – 2.84 (m, 1H + *min. rot.*), 2.68 (s, *min. rot.*), 2.16 (s, 3H), 1.89 – 1.55 (m, 2H + *min. rot.*), 1.36 – 1.08 (m, 1H + *min. rot.*), 0.86 (m, 1H + *min. rot.*).

^{13}C NMR (75 MHz, $CDCl_3$) δ 157.4 (C), 146.7 (C), 136.3 (CH), 132.1 (CH), 128.6 (CH), 128.3 (CH), 128.0 (CH), 127.9 (CH), 118.8 (C), 117.2 (CH), 111.6 (CH), 76.6 (CH), 67.7 (CH_2), 58.3 (CH), 38.3 (CH_2), 31.6 (CH_2), 31.0 (CH_2), 29.4 (CH_2), 29.1 (CH_2), 23.5 (CH_2), 22.7 (CH_2), 14.2 (CH_3), 11.5 (CH_3).

HRMS [ESI (+)]: m/z calcd. for $C_{22}H_{23}N_2O_3$: 363.1703 [M+H], found: 363.1695.

Benzyl (S*)-6-((S*)-hydroxy(4-nitrophenyl)methyl)-4-methyl-3,6-dihydropyridine-1(2H)-carboxylate (11g)

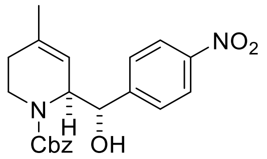

(390 nm lamp) 79.9 mg of *N*-tosylhydrazone **1f**, 36 mg of methylboronic acid and 61.7 mg of 4-nitrobenzaldehyde afforded 47.5 mg of **11g** (62% yield) as a yellow oil after column chromatography on SiO_2. Rf = 0.3 (Hex/EtOAc 2:1)

The compound is present in the NMR spectra as a mixture of rotamers.

^{1}H NMR (300 MHz, $CDCl_3$) δ 8.30 – 7.99 (m, 2H), 7.61 – 7.19 (m, 7H), 5.24 – 4.86 (m, 2H + *min. rot.*), 4.84 – 4.74 (m, 1H + *min. rot.*), 4.64 (s, 1H), 4.29 – 4.07 (m, 1H), 4.00 (s, *min. rot.*), 3.03 – 2.83 (m, 1H + *min. rot.*), 2.25 – 2.05 (m, 1H + *min. rot.*), 1.93 – 1.56 (m, 4H + *min. rot.*).

^{13}C NMR (75 MHz, $CDCl_3$) δ 157.4 (C), 148.8 (C), 147.6 (C), 137.6 (C), 136.6 (C), 136.3 (C), 128.6 (CH), 128.3 (CH), 128.0 (CH), 123.5 (CH), 117.2 (CH), 76.1 (CH), 75.3 (CH), 67.8 (CH_2), 58.4 (CH), 57.9 (CH), 38.4 (CH_2), 29.4 (CH_2), 23.5 (CH_3).

HRMS [ESI (+)]: m/z calcd. for $C_{21}H_{23}N_2O_5$: 383.1601 [M+H], found: 383.1602.

(*R)-(2-Bromophenyl)((*S**)-1-methyl-2-((Z)-pent-2-en-1-yl)-3-phenethylcyclopent-2-en-1-yl)methanol (12a)**

(370 nm lamp) 66.5 mg of *N*-tosylhydrazone **1i**, 90 mg 90 mg of phenethylboronic acid and 46.3 µL of 2-bromobenzaldehyde afforded 56.2 mg of **12a** (65% yield) as a colourless oil after column chromatography on SiO_2. Rf = 0.2 (20:1 Hex/EtOAc).

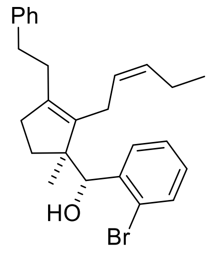

^{1}H NMR (300 MHz, $CDCl_3$) δ 7.61 (dd, *J* = 7.8, 1.8 Hz, 1H), 7.53 (dd, *J* = 8.0, 1.3 Hz, 1H), 7.37 – 7.26 (m, 3H), 7.23 (td, *J* = 7.6, 5.9 Hz, 3H), 7.12 (td, *J* = 7.6, 1.8 Hz, 1H), 5.36 (dtt, *J* = 10.9, 7.2, 1.9 Hz, 1H), 5.19 (s, 1H), 5.11 – 4.96 (m, 1H), 2.88 – 2.63 (m, 4H), 2.57 – 2.36 (m, 3H), 2.36 – 2.26 (m, 2H), 2.26 – 2.15 (m, 2H), 1.82 (d, *J* = 2.3 Hz, 1H), 1.24 (ddd, *J* = 11.8, 7.6, 4.7 Hz, 1H), 1.02 (t, *J* = 7.5 Hz, 3H), 0.95 (s, 3H).

^{13}C NMR (75 MHz, $CDCl_3$) δ 142.1 (C), 140.8 (C), 139.7 (C), 139.0 (C), 132.6 (CH), 132.1 (CH), 129.6 (CH), 128.8 (CH), 128.5 (CH), 128.5 (CH), 127.9 (CH), 127.2 (CH), 126.1 (CH), 124.5 (C), 74.2 (CH), 58.4 (C), 34.2 (CH_2), 32.8 (CH_2), 31.3 (CH_2), 30.0 (CH_2), 23.2 (CH_2), 22.4 (CH_3), 20.9 (CH_2), 14.3 (CH_3).

HRMS [APCI (+)]: m/z calcd. for $C_{26}H_{30}BrO$: 437.1475 [M-2H+H], found: 437.1453.

(S*,E)-3-(4-Methoxyphenyl)-1-((S*)-1-methyl-2-((Z)-pent-2-en-1-yl)-3-phenethylcyclopent-2-en-1-yl)prop-2-en-1-ol (12b)

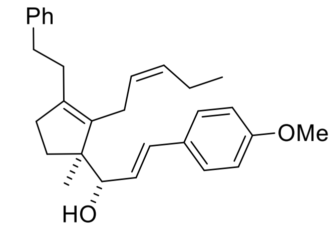

(370 nm lamp) 66.5 mg of *N*-tosylhydrazone **1i**, 90 mg of phenethylboronic acid and 64.9 mg of (E)-3-(4-methoxyphenyl)acrylaldehyde afforded 62.4 mg of **12b** (75% yield) as a colourless oil after column chromatography on SiO_2. Rf = 0.2 (10:1 Hex/EtOAc).

^{1}H NMR (300 MHz, $CDCl_3$) δ 7.39 – 7.15 (m, 7H), 6.92 – 6.81 (m, 2H), 6.56 (d, J = 15.8 Hz, 1H), 6.07 (dd, J = 15.8, 7.2 Hz, 1H), 5.32 (dtt, J = 10.9, 7.3, 1.8 Hz, 1H), 5.06 – 4.91 (m, 1H), 4.05 (d, J = 7.2 Hz, 1H), 3.82 (s, 3H), 2.87 – 2.67 (m, 2H), 2.71 – 2.60 (m, 2H), 2.60 – 2.24 (m, 3H), 2.29 – 2.06 (m, 3H), 1.59 (s, 1H), 1.43 (ddd, J = 12.8, 9.1, 5.2 Hz, 1H), 1.07 – 0.91 (m, 6H).

^{13}C NMR (75 MHz, $CDCl_3$) δ 159.2 (C), 142.0 (C), 140.1 (C), 138.3 (C), 131.8 (CH), 131.7 (CH), 130.0 (C), 128.5 (CH), 127.9 (CH), 127.7 (CH), 126.4 (CH), 126.1 (CH), 114.0 (CH), 76.9 (CH), 56.8 (C), 55.4 (CH_3), 34.2 (CH_2), 33.3 (CH_2), 31.2 (CH_2), 30.3 (CH_2), 23.6 (CH_3), 23.0 (CH_2), 20.8 (CH_2), 14.2 (CH_3).

HRMS [APCI (+)]: m/z calcd. for $C_{29}H_{35}O_2$: 415.2632 [M-2H+H], found: 415.2624.

(S*,E)-3-(4-Methoxyphenyl)-1-((S*)-1-methyl-2-((Z)-pent-2-en-1-yl)-3-propylcyclopent-2-en-1-yl)prop-2-en-1-ol (12c)

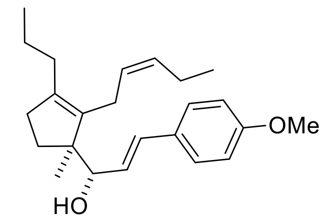

(370 nm lamp) 66.5 mg of *N*-tosylhydrazone **1i**, 53 mg of propylboronic acid and 64.9 mg of (E)-3-(4-methoxyphenyl)acrylaldehyde afforded 56.2 mg of **12c** (80% yield) as a colourless oil after column chromatography on SiO_2. Rf = 0.25 (10:1 Hex/EtOAc).

^{1}H NMR (300 MHz, $CDCl_3$) δ 7.39 – 7.28 (m, 2H), 6.91 – 6.80 (m, 2H), 6.57 (d, J = 15.8 Hz, 1H), 6.09 (dd, J = 15.8, 7.1 Hz, 1H), 5.46 – 5.21 (m, 1H), 4.14 (d, J = 7.1 Hz, 1H), 3.80 (s, 3H), 2.84 (d, J = 7.1 Hz, 2H), 2.32 – 1.97 (m, 7H), 1.80 (s, 1H), 1.53 – 1.34 (m, 3H), 1.09 – 0.95 (m, 6H), 0.91 (t, J = 7.3 Hz, 3H).

^{13}C NMR (75 MHz, $CDCl_3$) δ 159.2 (C), 141.5 (C), 137.2 (C), 131.8 (CH), 131.6 (CH), 130.1 (C), 128.2 (CH), 127.7 (CH), 126.6 (CH), 114.0 (CH), 77.0 (CH), 56.7 (C), 55.4 (CH_3), 33.2 (CH_2), 31.1 (CH_2), 30.4 (CH_2), 23.7 (CH_3), 23.2 (CH_2), 21.4 (CH_2), 20.8 (CH_2), 14.3 (CH_3), 14.2 (CH_3).

HRMS [EI (+)]: m/z calcd. for $C_{24}H_{34}O_2$: 354.2553 [M+H] , found: 354.2554.

(S*)-1-((S*)-1,3-dimethyl-2-((Z)-pent-2-en-1-yl)cyclopent-2-en-1-yl)-2-methylprop-2-en-1-ol (12d)

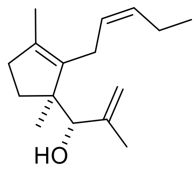

(370 nm lamp) 66.5 mg of *N*-tosylhydrazone **1i**, 36 mg of methylboronic acid and 34.8 µL of methacrolein afforded 36.9 mg of **12d** (79% yield) as a colourless oil after column chromatography on SiO_2. Rf = 0.2 (20:1 Hex/EtOAc).

^{1}H NMR (300 MHz, $CDCl_3$) δ 5.45 – 5.17 (m, 2H), 4.98 – 4.88 (m, 2H), 4.03 (s, 1H), 2.78 (d, *J* = 6.9 Hz, 2H), 2.32 – 2.08 (m, 5H), 1.81 – 1.74 (m, 3H), 1.67 (s, 3H), 1.60 (s, 1H), 1.43 – 1.22 (m, 1H), 1.00 (t, *J* = 7.5 Hz, 3H), 0.93 (s, 3H).

^{13}C NMR (75 MHz, $CDCl_3$) δ 145.4 (C), 137.8 (C), 136.1 (C), 131.7 (CH), 127.9 (CH), 114.6 (CH_2), 78.9 (CH), 56.1 (C), 36.0 (CH_2), 30.4 (CH_2), 23.5 (CH_3), 23.3 (CH_2), 20.8 (CH_2), 20.0 (CH_3), 14.6 (CH_3), 14.2 (CH_3).

HRMS [APCI (+)]: m/z calcd. for $C_{16}H_{26}O$+Na: 257.1876 [M+Na], found: 257.1871.

(5*R*,8*R*,9*S*,10*R*,13*S*,14*S*,17*S*)-5-((*R*)-Hydroxy(p-tolyl)methyl)-10,13,17-trimethyl-3-propyl-2,5,6,7,8,9,10,11,12,13,14,15,16,17-tetradecahydro-1H-cyclopenta[a]phenanthren-17-ol (13)

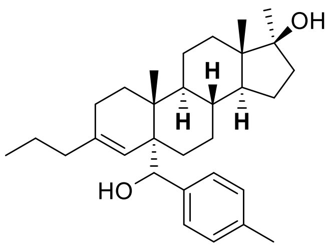

(390 nm lamp) 94.2 mg of *N*-tosylhydrazone **1k**, 53 mg of propylboronic acid and 47.3 µL of 4-methylbenzaldehyde afforded 28 mg of **13** (31% yield) as a colourless oil after column chromatography on SiO_2. Rf = 0.32 (3:1 Hex/EtOAc).

^{1}H NMR (300 MHz, $CDCl_3$) δ 7.11 (d, *J* = 8.0 Hz, 2H), 7.05 (d, *J* = 8.0 Hz, 2H), 4.80 (s, 1H), 4.19 (s, 1H), 2.32 (s, 3H), 2.11 – 2.01 (m, 1H), 1.89 – 1.66 (m, 9H), 1.53 – 1.41 (m, 6H), 1.36 – 1.23 (m, 6H), 1.17 (s, 3H), 1.12 (s, 3H), 1.10 – 0.98 (m, 2H), 0.88 – 0.76 (m, 7H).

^{13}C NMR (75 MHz, $CDCl_3$) δ 140.7 (C), 137.2 (C), 136.4 (C), 129.5 (CH), 128.4 (CH), 128.0 (CH), 81.9 (C), 78.2 (CH), 51.1 (CH), 46.9 (C), 45.3 (C), 43.5 (CH), 39.7 (CH_2), 39.2 (CH_2), 37.6 (C), 36.2 (CH_3), 32.2 (CH_2), 29.1 (CH_2), 27.7 (CH_2), 25.8 (CH_3), 25.7 (CH_2), 25.1 (CH_2), 23.4 (CH_2), 21.5 (CH_2), 21.2 (CH_3), 20.9 (CH_2), 18.9 (CH_3), 14.1 (CH_3), 14.0 (CH_3).

HRMS [ESI(+)]: m/z calcd. for $C_{31}H_{46}O_2$+Na: 473.3390 [M+Na], found: 473.3413.

(*R)-((1*R**,4*R**)-4-Allyl-3,6,6-trimethylcyclohex-2-en-1-yl)(4-nitrophenyl)methanol (16a)**

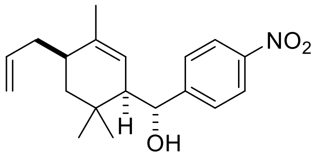

(390 nm lamp) 66.5 mg of *N*-tosylhydrazone **15**, 36 mg of methylboronic acid and 61.7 mg of 4-nitrobenzaldehyde afforded 48 mg of **16a** (76% yield) as a yellow oil after column chromatography on SiO_2. Rf = 0.32 (5:1 Hex/EtOAc).

^{1}H NMR (300 MHz, $CDCl_3$) δ 8.15 (d, *J* = 8.7 Hz, 2H), 7.44 (d, *J* = 8.7 Hz, 2H), 5.68 – 5.46 (m, 1H), 5.07 – 5.01 (m, 1H), 5.01 – 4.93 (m, 1H), 4.76 (dt, *J* = 5.6, 1.7 Hz, 1H), 4.72 (d, *J* = 6.4 Hz, 1H), 2.28 – 2.11 (m, 2H), 2.10 – 1.96 (m, 3H), 1.55 (s, 3H), 1.34 – 1.17 (m, 2H), 1.09 (s, 3H), 0.93 (s, 3H).

^{13}C NMR (75 MHz, $CDCl_3$) δ 151.7 (C), 147.1 (C), 137.1 (C), 136.2 (CH), 128.3 (CH), 123.9 (CH), 123.2 (CH), 116.9 (CH_2), 75.8 (CH), 52.6 (CH), 38.3 (CH_2), 36.9 (CH_2), 36.1 (CH), 32.7 (C), 29.2 (CH_3), 28.6 (CH_3), 21.4 (CH_3).

HRMS [EI(+)]: m/z calcd. for $C_{19}H_{23}NO_2$: 297.1729 [M-H_2O], found: 297.1721.

(*R)-((1*R**,4*R**)-4-Allyl-3-(but-3-en-1-yl)-6,6-dimethylcyclohex-2-en-1-yl)(4-methoxyphenyl)methanol (16b)**

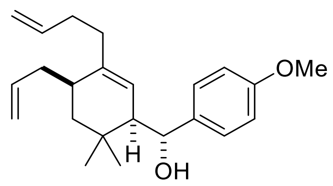

(390 nm lamp) 66.5 mg of *N*-tosylhydrazone **15**, 60 mg of 3-butenylboronic acid and 49.6 µL of *p*-anisaldehyde afforded 54.5 mg of **16b** (80% yield) as a colourless oil after column chromatography on SiO_2. Rf = 0.16 (10:1 Hex/EtOAc).

^{1}H NMR (300 MHz, $CDCl_3$) δ 7.18 (d, *J* = 8.6 Hz, 2H), 6.83 (d, *J* = 8.6 Hz, 2H), 5.72 (ddt, *J* = 16.9, 10.1, 3.5 Hz, 1H), 5.57 (ddt, *J* = 18.4, 15.6, 7.0 Hz, 1H), 5.03 – 4.85 (m, 5H), 4.58 (d, *J* = 6.7 Hz, 1H), 3.79 (s, 3H), 2.29 – 2.02 (m, 5H), 2.02 – 1.79 (m, 4H), 1.12 (s, 3H), 0.92 (s, 3H).

^{13}C NMR (75 MHz, $CDCl_3$) δ 158.9 (C), 138.7 (CH), 138.5 (C), 136.9 (CH), 136.4 (C), 128.7 (CH), 124.5 (CH), 116.3 (CH_2), 114.5 (CH_2), 113.4 (CH), 76.0 (CH), 55.3 (CH), 52.4 (CH), 38.2 (CH_2), 36.8 (CH_2), 34.1 (CH_2), 34.0 (CH_3), 32.2 (CH_2), 32.2 (C), 29.0 (CH_3), 28.8 (CH_3).

HRMS [EI(+)]: m/z calcd. for $C_{23}H_{30}O$: 322.2297 [M-H_2O], found: 322.2298.

4-((*R)-((1*R**,4*R**)-4-allyl-3-cyclopropyl-6,6-dimethylcyclohex-2-en-1-yl)(hydroxy)methyl)benzonitrile (16c)**

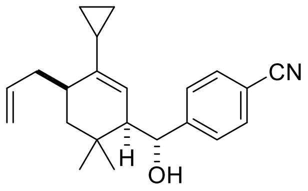

(390 nm lamp) 66.5 mg of *N*-tosylhydrazone **15**, 60 mg of 3-butenylboronic acid and 53.5 mg of 4-formylbenzonitrile afforded 56.6 mg of **16c** (88% yield) as a colourless oil after column chromatography on SiO_2. Rf = 0.22 (5:1 Hex/EtOAc).

^{1}H NMR (300 MHz, $CDCl_3$) δ 7.58 (d, *J* = 8.2 Hz, 2H), 7.34 (d, *J* = 8.2 Hz, 2H), 5.74 – 5.52 (m, 1H), 5.08 – 4.93 (m, 2H), 4.62 (t, *J* = 6.0 Hz, 2H), 2.56 – 2.39 (m, 1H), 2.24 – 2.10 (m, 2H), 2.10 – 1.97 (m, 2H), 1.31 – 1.12 (m, 2H), 1.09 (s, 3H), 0.88 (s, 3H), 0.62 – 0.50 (m, 1H), 0.48 – 0.36 (m, 1H), 0.13 – -0.03 (m, 2H).

^{13}C NMR (75 MHz, $CDCl_3$) δ 149.5 (C), 141.5 (C), 137.0 (CH), 131.8 (CH), 128.3 (CH), 120.5 (CH), 119.1 (C), 116.5 (CH2), 110.9 (C), 76.0 (CH), 52.2 (CH), 38.5 (CH_2), 37.6 (CH_2), 36.3 (CH_3), 32.3 (C), 29.1 (CH_3), 28.4 (CH_3), 14.5 (CH_3), 7.2 (CH_2), 4.0 (CH_2).

HRMS [APCI (+)]: m/z calcd. for $C_{22}H_{26}NO$: 320.2009 [M-2H+H], found: 320.1999.

4. Additional synthetic transformations: Experimental procedures and characterization data.

(*R)-((*R**)-6,6-Dimethyl-3-propylcyclohex-2-en-1-yl)(4-nitrophenyl)methyl 4-bromobenzoate (6)**

Experimental procedure: A 5 mL flask was charged with the homoallylic alcohol 5c (144.1 mg, 0.475 mmol), 4-bromobenzoyl chloride (125.1 mg, 0.570 mmol, 1.2 eq.), DMAP (73.4 mg, 0.617 mmol, 1.3 eq.) and 1.6 mL of CH_2Cl_2. The reaction mixture was stirred at room temperature for 16 hours. Afterwards, glycol (26.6 µL, 0.475 mmol, 1 eq.) was added to quench the excess of the chloride and it was let to stir

for 5 minutes. The solvents were removed under reduced pressure and the compound **6** was purified by column chromatography in SiO_2 to yield 197 mg (86%) as a white crystalline solid. Rf = 0.25 (20:1 Hex/EtOAc).

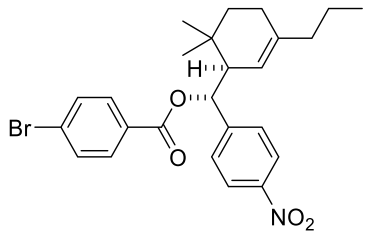

^{1}H NMR (300 MHz, $CDCl_3$) δ 8.22 – 8.14 (m, 2H), 7.95 – 7.86 (m, 2H), 7.59 (d, J = 3.5 Hz, 2H), 7.56 (d, J = 3.6 Hz, 2H), 5.86 (d, J = 9.0 Hz, 1H), 4.62 (d, J = 1.8 Hz, 1H), 2.69 – 2.59 (m, 1H), 1.95 – 1.86 (m, 2H), 1.86 – 1.76 (m, 2H), 1.49 – 1.35 (m, 2H), 1.35 – 1.21 (m, 2H), 1.09 (s, 3H), 0.96 (s, 3H), 0.78 (t, J = 7.3 Hz, 3H).

^{13}C NMR (75 MHz, $CDCl_3$) δ 164.7 (C), 147.7 (C), 147.5 (C), 140.5 (C), 132.0 (CH), 131.3 (CH), 128.9 (CH), 128.6 (C), 123.6 (CH), 119.1 (CH), 78.0 (CH), 49.4 (CH), 39.7 (CH_2), 35.6 (CH_2), 32.1 (C), 29.8 (CH_3), 25.7 (CH_2), 24.5 (CH_3), 20.9 (CH_2), 13.8 (CH_3).

HRMS [APCI (+)]: m/z calcd. for $C_{18}H_{24}NO_2^+$: 286.1802 [M-$C_7H_5BrO_2$], found: 286.1796.

m.p. = 98.5 – 100.5 ºC

6-(1-Hydroxy-4,4-dimethylcyclohex-2-en-1-yl)hexan-2-one (17)

Experimental procedure: Following the General Procedure A, once the carboborylation reaction is finished, the mixture was treated with H_2O_2 in H_2O and stirred overnight. The solution was diluted with 5 mL of water and extracted with CH_2Cl_2 (3x10 mL). The combined organic layers were washed with brine, dried over Na_2SO_4 and concentrated. The hydroxyketone **17** was purified by column chromatography in SiO_2 to yield 31 mg (62 %) as a white solid. Rf = 0.48 (1:1 Hex/EtOAc).

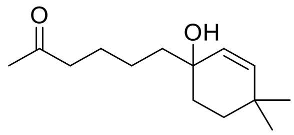

^{1}H NMR (300 MHz, $CDCl_3$) δ 5.49 (d, J = 9.9 Hz, 1H), 5.42 (d, J = 9.9 Hz, 1H), 2.44 (t, J = 7.3 Hz, 2H), 2.13 (s, 3H), 1.69 – 1.36 (m, 10H), 1.00 (s, 3H), 0.93 (s, 3H).

^{13}C NMR (75 MHz, $CDCl_3$) δ 209.3 (C), 140.3 (CH), 130.1 (CH), 69.8 (C), 43.8 (CH_2), 41.9 (CH_2), 33.7 (CH_2), 32.6 (CH_2), 32.1 (C), 30.1 (CH_3), 27.8 (CH_3), 24.4 (CH_2), 23.3 (CH_2).

HRMS [ESI(+)]: m/z calcd. for $C_{14}H_{24}O_2$+Na: 247.1669 [M+Na], found: 247.1670.

m.p. = 69 – 71 ºC

6-(4,4-Dimethylcyclohex-1-en-1-yl)hexan-2-one (18)

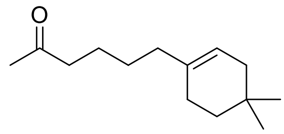

Experimental procedure: The boronic ester **4e** (33.4 mg, 0.1 mmol) was treated with ^{t}BuOK (11.2 mg, 0.1 mmol, 1 eq.) in 1 mL 1,4-dioxane at reflux and stirred overnight under inter atmosphere. The solution was extracted with CH_2Cl_2 (3x10 mL). The combined organic layers were washed with brine, dried over Na_2SO_4 and concentrated. The protodeboronated compound **18** was purified by column chromatography in SiO_2 to yield 12 mg (57%) as a colourless oil. Rf = 0.22 (20:1 Hex/EtOAc).

^{1}H NMR (300 MHz, $CDCl_3$) δ 5.33 – 5.26 (m, 1H), 2.42 (t, *J* = 7.3 Hz, 2H), 2.13 (s, 3H), 1.99 – 1.85 (m, 4H), 1.75 (dt, *J* = 4.0, 2.0 Hz, 2H), 1.57 – 1.47 (m, 2H), 1.43 – 1.30 (m, 4H), 0.88 (s, 6H).

^{13}C NMR (75 MHz, $CDCl_3$) δ 209.5 (C), 136.0 (C), 120.3 (CH), 43.9 (CH_2), 39.4 (CH_2), 37.4 (CH_2), 35.9 (CH_2), 30.0 (CH_3), 28.6 (C), 28.4 (CH_3), 27.4 (CH_2), 26.1 (CH_2), 23.7 (CH_2).

HRMS [ESI(+)]: m/z calcd. for $C_{14}H_{25}O$: 209.1900 [M+H], found: 209.1898.

(1-Cyclopropyl-4,4-dimethylcyclohex-2-en-1-yl)methanol (19)

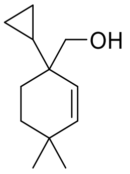

Experimental procedure: To a stirred solution of the boronic ester **4h** (0.250 mmol, 69.06 mg) and dibromomethane (1.25 mmol, 87 µL) in anhydrous THF (2.5 mL, 0.1 M) at –78 °C, was added n-BuLi (2.5 M in hexanes, 1.10 mmol, 440 µL) dropwise. The resulting mixture was stirred for 10 min at –78 °C, warmed to room temperature and stirred for 6 h. The reaction mixture was then cooled to 0 °C and a solution of 2 N NaOH / 30% H_2O_2 (2:1 v/v, 2.5 mL) was added dropwise. This mixture was stirred for overnight at room temperature and diluted with EtOAc (15 mL). The layers were separated and the aqueous layer was extracted with EtOAc (2 × 10 mL). The combined organic layers were washed with water (5 mL) and brine (5 mL), dried (Na_2SO_4), filtered and concentrated in vacuo. The crude residue was purified by flash column chromatography in SiO_2 to yield 18 mg (40% yield) of the alcohol **19** as a colourless oil. Rf = 0.18 (Hex/Acetone 10:1).

^{1}H NMR (300 MHz, $CDCl_3$) δ 5.60 (d, *J* = 10.2 Hz, 1H), 4.94 (d, *J* = 10.2 Hz, 1H), 3.49 (d, *J* = 10.6 Hz, 1H), 3.39 (d, *J* = 10.6 Hz, 1H), 1.79 – 1.65 (m, 1H), 1.65 – 1.52 (m, 2H), 1.47 – 1.41 (m, 2H), 0.96 (s, 3H), 0.95 (s, 3H), 0.67 (tt, *J* = 8.1, 5.8 Hz, 1H), 0.44 – 0.33 (m, 1H), 0.32 – 0.09 (m, 3H).

^{13}C NMR (75 MHz, $CDCl_3$) δ 142.6 (CH_2), 124.7 (CH_2), 71.3 (CH), 39.4 (C), 33.5 (CH), 32.0 (C), 29.9 (CH_3), 29.3 (CH_3), 28.1 (CH_2), 17.5 (CH), 0.7 (CH_2), -1.7 (CH_2).

HRMS [ESI(+)]: m/z calcd. for $C_{12}H_{21}O$: 181.1587 [M+H], found: 181.1591

3-(4,4-Dimethyl-1-propylcyclohex-2-en-1-yl)-3-phenylpropan-1-ol (20)

Experimental procedure: A 10 mL flask was charged with NaH (40.80 mg, 1.70 mmol) and 3 mL of THF. To the stirred solution, 15-Crown-5 (20.3 µL, 0.1 mmol) and the homoallylic alcohol **5n** were added dropwise. The mixture was heated to 80 °C for 16 hours. The flask was cooled down to room temperature and afterwards $NaBH_4$ (38.60 mg, 1 mmol) was added to the reaction. The mixture was stirred for 2 hours at room temperature. The solution was extracted with EtOAc (2x10 mL). The combined organic layers were washed with brine, dried over Na_2SO_4 and concentrated. The product **20** was purified by column chromatography in SiO_2 to yield 23 mg (40%) as colourless oil. Rf = 0.2 (5:1 Hex/EtOAc).

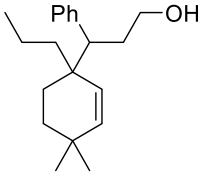

^{1}H NMR (300 MHz, $CDCl_3$) δ 7.28 – 7.16 (m, 5H), 5.46 (d, *J* = 10.2 Hz, 1H), 5.26 (d, *J* = 10.2 Hz, 1H), 3.44 (ddd, *J* = 10.5, 7.2, 5.0 Hz, 1H), 3.33 (dt, *J* = 10.5, 7.3 Hz, 1H), 2.71 (dd, *J* = 11.1, 3.7 Hz, 1H), 2.09 – 1.85 (m, 2H), 1.79 – 1.62 (m, 1H), 1.43

– 1.29 (m, 4H), 1.22 – 1.13 (m, 2H), 1.13 – 0.96 (m, 2H), 0.93 (s, 3H), 0.85 – 0.79 (m, 6H).

^{13}C NMR (75 MHz, $CDCl_3$) δ 142.1 (C), 138.0 (CH), 132.1 (CH), 129.8 (CH), 127.9 (CH), 126.4 (CH), 62.3 (CH_2), 49.4 (CH), 41.2 (C), 41.1 (CH_2), 33.9 (CH_2), 33.5 (CH_2), 31.4 (C), 30.5 (CH_3), 28.6 (CH_3), 26.9 (CH_2), 17.3 (CH_2), 15.0 (CH_3).

HRMS [ESI(+)]: m/z calcd. for $C_{20}H_{30}O$+Na: 309.2189 [M+Na], found: 309.2196.

(4*aS*,9*R***,9*aR***)-1,1-Dimethyl-4-methylene-2,3,4,4a,9,9a-hexahydro-1H-fluoren-9-ol (21)**

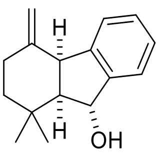

Experimental procedure: A 5 mL vial was charged with the homoallylic alcohol **5o** (30.92 mg, 0.1 mmol), ^{t}BuOLi (32.02 mg, 0.4 mmol), Pd_2dba_3 (3.66 mg, 4 mol%, 0.004 mmol), Xphos (3.81 mg, 8 mol%, 0.008 mmol) and 1 mL of 1,4-dioxane. The reaction was carried out under Ar atmosphere at 110 °C for 24 hours. The flask was let to cool down at room temperature and the crude was filtered with 10 mL of CH_2Cl_2 through a celite pad. The solvents were removed under reduced pressure and the compound **21** was purified by column chromatography in SiO_2 to yield 18.7 mg (82%) as a yellow oil. Rf = 0.33 (5:1 Hex/EtOAc).

^{1}H NMR (600 MHz, $CDCl_3$) δ 7.46 – 7.41 (m, 1H), 7.31 – 7.27 (m, 2H), 7.26 – 7.21 (m, 1H), 4.98 (d, *J* = 8.2 Hz, 1H), 4.74 (t, *J* = 2.1 Hz, 1H), 4.21 (q, *J* = 1.7 Hz, 1H), 3.88 – 3.81 (m, 1H), 2.35 (td, *J* = 12.8, 12.2, 4.6 Hz, 1H), 2.22 (dt, *J* = 14.3, 4.8 Hz, 1H), 2.09 (t, *J* = 8.1 Hz, 1H), 1.67 (s, 1H), 1.60 (td, *J* = 12.6, 11.9, 4.3 Hz, 1H), 1.54 (dtd, *J* = 13.3, 4.7, 1.3 Hz, 1H), 1.19 (s, 3H), 1.12 (s, 3H).

^{13}C NMR (151 MHz, $CDCl_3$) δ 149.1 (C), 145.5 (C), 143.5 (C), 127.5 (CH), 127.2 (CH), 125.4 (CH), 124.2 (CH), 110.1 (CH_2), 75.8 (CH), 63.1 (CH), 46.8 (CH), 38.4 (CH_2), 32.2 (C), 30.9 (CH_2), 29.4 (CH_3), 29.2 (CH_3).

HRMS [EI(+)]: m/z calcd. for $C_{16}H_{20}O$: 228.1509 [M], found: 228.1514.

(1*S*,8*aS***)-7-Methyl-1-phenyl-1,5,6,8a-tetrahydro-3H-oxazolo[3,4-a]pyridin-3-one (22)**

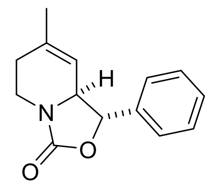

Experimental procedure: A 10 mL Schlenk tube was charged with benzyl 6-(hydroxy(phenyl)methyl)-4-methyl-3,6-dihydropyridine-1(2H)-carboxylate (32 mg, 0.1 mmol) and LiOtBu (32 mg, 0.4 mmol, 4 eq) in 1 mL of 1,4-dioxane. The Schenk was equipped with a reflux condenser and the reaction mixture was stirred under heating from an external oil bath (100ºC, 12 h). The reaction mixture was passed through a short pad of celite, providing compound **22** in quantitative yield (99%) and in a pure form.

^{1}H NMR (600 MHz, $CDCl_3$) δ 7.45 – 7.34 (m, 5H), 5.47 (q, *J* = 1.7 Hz, 1H), 4.99 (d, *J* = 7.2 Hz, 1H), 4.18 – 4.11 (m, 1H), 4.02 (dd, *J* = 13.5, 7.0 Hz, 1H), 3.02 (ddd, *J* = 13.5, 11.6, 4.7 Hz, 1H), 2.41 – 2.32 (m, 1H), 1.92 (ddd, *J* = 17.2, 4.9, 2.4 Hz, 1H), 1.77 (s, 3H).

^{13}C NMR (151 MHz, $CDCl_3$) δ 157.0 (C), 138.2 (C), 136.0 (C), 129.0 (CH), 125.9 (CH), 118.7 (CH), 82.3 (CH), 60.6 (CH), 38.0 (CH_2), 28.9 (CH_2), 23.8 (CH_3).

HRMS [EI(+)]: m/z calcd. for $C_{14}H_{15}NO_2$: 229.1097 [M], found: 229.1095.

(1*R)-((3*R**)-2,2-Dimethyl-2,3,5,6,9,9a-hexahydro-1H-benzo[7]annulen-3-yl)(4-methoxyphenyl)methanol (23)**

Experimental procedure: A 5 mL vial was charged with the homoallylic alcohol **16b** (51.08 mg, 0.15 mmol), Grubbs Catalyst 2nd Generation (12.73 mg, 10 mol%, 0.015 mmol) and 3 mL DCM. The reaction mixture was stirred for 4 hours at room temperature. The crude was filtered with 10 mL of CH_2Cl_2 through a celite pad. The solvents were removed under reduced pressure and the compound **23** was purified by column chromatography in SiO_2 to yield 26.4 mg (57%) as a brown oil. Rf = 0.3 (5:1 Hex/EtOAc).

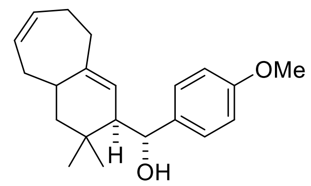

^{1}H NMR (300 MHz, $CDCl_3$) δ 7.21 (d, *J* = 8.7 Hz, 2H), 6.82 (d, *J* = 8.7 Hz, 2H), 5.76 – 5.58 (m, 2H), 5.00 (d, *J* = 5.1 Hz, 1H), 4.63 (d, *J* = 6.2 Hz, 1H), 3.79 (s, 3H), 2.43 – 2.11 (m, 5H), 2.07 – 1.73 (m, 4H), 1.08 (s, 3H), 1.05 – 0.99 (m, 1H), 0.96 (s, 3H).

^{13}C NMR (75 MHz, $CDCl_3$) δ 158.90 (C), 142.12 (C), 136.20 (C), 131.65 (CH), 129.84 (CH), 128.97 (CH), 121.80 (CH), 113.27 (CH), 76.13 (CH), 55.36 (CH), 52.05 (CH), 40.78 (CH_2), 36.15 (CH_3), 35.46 (CH_2), 33.29 (CH_2), 32.21 (C), 28.85 (CH_3), 28.66 (CH_3), 28.44 (CH_2).

HRMS [APCI (+)]: m/z calcd. for $C_{21}H_{27}O_2$: 311.2006 [M-2H+H], found: 311.1996.

(*E*)-2-(2-cyclopropylhept-3-en-2-yl)-4,4,5,5-tetramethyl-1,3,2-dioxaborolane 28

Following general procedure A 56 mg (0.2 mmol) of *N*'-((*E*)-hept-3-en-2-ylidene)-4-methylbenzenesulfonohydrazide afforded 9 mg (18 %) of boronate **28** after column chromatography on SiO_2. (Hex/EtOAc 50:1), Rf = 0.38.

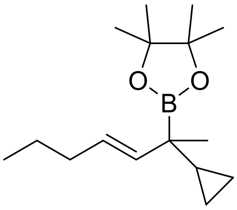

^{1}H NMR (400 MHz, $CDCl_3$) δ 5.53 – 5.42 (m, 1H), 5.35 (dtd, *J* = 15.5, 6.6, 1.5 Hz, 1H), 1.99 (q, *J* = 7.2 Hz, 2H), 1.35 (tdd, *J* = 10.6, 8.2, 3.8 Hz, 2H), 1.26 – 1.17 (m, 12H), 0.98 (d, *J* = 1.7 Hz, 3H), 0.87 (td, *J* = 7.4, 1.7 Hz, 4H), 0.37 – 0.15 (m, 4H).

^{13}C NMR (101 MHz, $CDCl_3$) δ 135.7 (CH), 127.4 (CH), 82.9 (C), 35.1 (CH_2), 24.6 (CH_3), 24.5 (CH_3), 22.9 (CH_2), 20.0 (CH_3), 18.7 (CH), 13.5 (CH_3), 0.79 (CH_2), 0.63 (CH_2).

^{11}B NMR (129 MHz, $CDCl_3$) 33,61

HRMS [EI(+)]: m/z calcd. for $C_{16}H_{29}BO_2$ (M^+): 263.2294 found: 263.2286.

(1*S,2*S**)-1-(4-Methoxyphenyl)-4-methyl-2-propylpent-3-en-1-ol 30 and (**E**)-1-(4-Methoxyphenyl)-2,2-dimethylhept-3-en-1-ol 31**

Following the general procedure B: (390 nm lamp) 56 mg (0.2 mmol) of *N*'-((*E*)-hept-3-en-2-ylidene)-4-methylbenzenesulfonohydrazide 36 mg of methylboronic acid and 64.2 µL of 2-bromobenzaldehyde afforded 14 mg of **30** and 11 mg of **31** (51% combined yield) after column chromatography on SiO_2. (Hex/EtOAc 8:1). Rf (**30**)= 0.19; Rf (**31**)= 0.24.

(1*S,2*S**)-1-(4-Methoxyphenyl)-4-methyl-2-propylpent-3-en-1-ol 30**

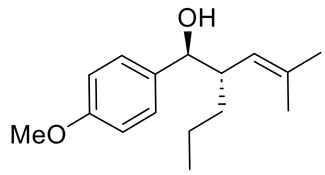

^{1}H NMR (300 MHz, $CDCl_3$) δ 7.37 – 7.18 (m, 2H), 6.98 – 6.78 (m, 2H), 4.97 (d, *J* = 10.1 Hz, 1H), 4.21 (d, *J* = 8.5 Hz, 1H), 3.81 (s, 3H), 2.60 – 2.41 (m, 1H), 1.80 (d, *J* = 1.4 Hz, 3H), 1.67 (s, 3H), 1.34 – 1.21 (2, 3H), 1.11 – 1.01 (m, 2H), 0.75 (t, *J* = 6.6 Hz, 3H).

^{13}C NMR (101 MHz, $CDCl_3$) δ 159.2 (C), 137.0 (C), 135.2 (C), 128.4 (CH), 126.0 (CH), 113.7 CH), 77,6 (CH). 55.4 (CH_3), 47.1 (CH), 33.8 (CH_2), 26.2 (CH_3), 20.5 (CH_2), 18.7 (CH_3), 14.2 (CH_3).

HRMS [EI(+)]: m/z calcd. for $C_{16}H_{22}O$ (M-H_2O): 230.1665 found: 230.1660.

1-(4-Methoxyphenyl)-2,2-dimethylhept-3-en-1-ol 31

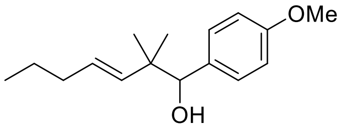

^{1}H NMR (300 MHz, $CDCl_3$) δ 7.20 (d, *J* = 8.7 Hz, 2H), 6.88 – 6.79 (m, 2H), 5.47 (m, 2H), 4.34 (s, 1H), 3.80 (s, 3H), 2.08 – 1.99 (m, 2H), 1.40 (h, *J* = 7.4 Hz, 2H), 0.97 (s, 3H), 0.94 – 0.83 (m, 6H).

^{13}C NMR (101 MHz, $CDCl_3$) δ 159.0 (C), 137.0 (CH), 133.2 (C), 130.1 (CH), 129.0 (CH), 113.0 (CH), 80.6 (CH), 55.4 (CH_3), 41.7 (C), 35.1 (CH_2), 25.3 (CH_2), 22.9 (CH_3), 21.7 (CH_3), 13.9 (CH_3).

HRMS [EI(+)]: m/z calcd. for $C_{16}H_{22}O$ (M-H_2O): 230.1665 found: 230.1664.

5. X-Ray Crystallographic data of compound 6.

A full data set was collected from a suitable single transparent crystal at 298 K in a Rigaku (Oxford diffraction) Xcalibur Nova diffractometer using microfocus Cu K_α radiation and the oscillation method (1.20° frame width and variable exposure time). Data was processed using CrysAlisPro software and an empirical (ABSPACK), and a numerical absorption correction based on a multifaceted crystal model were applied. The structure was solved using SHELXT and space group determined to be $P\bar{1}$. All the non-hydrogen atoms of the expected chemical structure were identified. Some atoms were reassigned. Least-square refinement cycles were carried out using SHELXL program for a final convergence with *wR2* = 0.6908 for all 4416 data and R = 0.2503 for 3694 ($F_o>4\sigma(F_o)$) data. Difference electron density synthesis had no peaks higher than 4.23 $e/Å^3$and deeper than 5.21 $e/Å^3$.

Thermal displacement parameters were then anisotropically refined. Least-square refinement cycles yielded a final *wR2* = 0.2211 for all data and R = 0.0659 $F_o>4\sigma(F_o)$ data and 280 parameters. Difference electron density synthesis had no peaks higher than 0.58 $e/Å^3$and deeper than 0.48 $e/Å^3$. Final refinement cycles after placing hydrogen atoms with isotropic displacement factor 1.2 times of the corresponding riding atom (1.5 times for methyl groups) and weight adjustment stopped with *wR2* = 0.1171, GoF = 1.046 and R = 0.454 for all data and R = 0.0390 $F_o>4\sigma(F_o)$ data and 283 parameters. Difference electron density synthesis had no peaks higher than 0.23 $e/Å^3$and deeper than 0.49 $e/Å^3$.

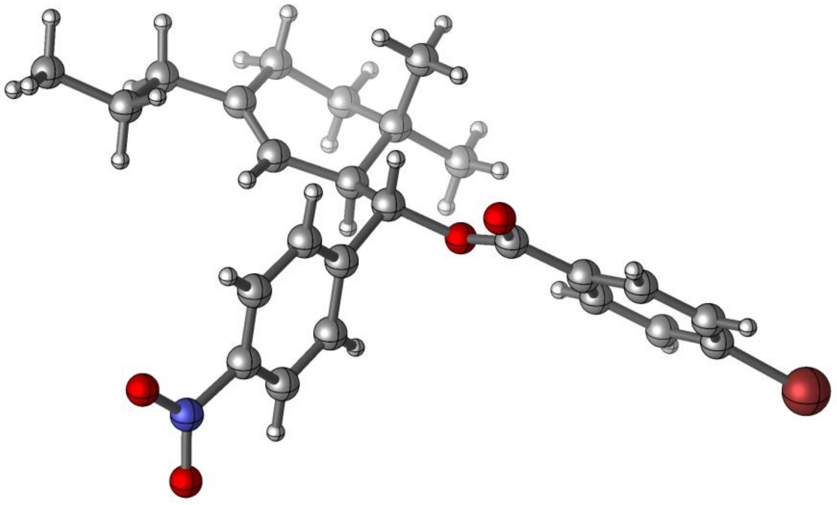

Figure SI-6. Three-dimensional structure of compound **6** determined by X-ray diffraction.

Crystal data, data collection and structure refinement details

**Crystal data**	
Chemical formula	$C_{25}H_{28}BrNO_4$
Formula weight	486.39
Crystal system, space group	Triclinic, $P\bar{1}$
Temperature (K)	298
a, *b*, *c* (Å)	9.6644 (3), 11.0309 (5), 11.8075 (5)
a, b, g (°)	82.030 (4), 71.492 (3), 86.773 (3)
Volume (Å^3)	1182.03 (8)
Z	2.0
Radiation type	Cu *K*a
m (mm^{-1})	2.61
Crystal size (mm)	0.37 × 0.25 × 0.15
**Data collection**	
Diffractometer	Xcalibur, Onyx, Nova
Absorption correction	Gaussian<br>*CrysAlis PRO* 1.171.38.46 (Rigaku Oxford Diffraction, 2015) Numerical absorption correction based on gaussian integration over a multifaceted crystal model Empirical absorption correction using spherical harmonics, implemented in SCALE3 ABSPACK scaling algorithm.
T_{min}, T_{max}	0.358, 1.000
No. of measured, independent and observed [$I > 2s(I)$] reflections	21883, 4416, 3694
R_{int}	0.036
(sin q/l)$_{max}$ (Å^{-1})	0.608
**Refinement**	
$R[F^2 > 2s(F^2)]$, $wR(F^2)$, S	0.039, 0.117, 1.05
No. of reflections	4416
No. of parameters	283
H-atom treatment	H-atom parameters constrained
Dρ_{max}, Dρ_{min} (e Å^{-3})	0.23, -0.49

All data related to the X-ray experiment for determining the structure of this compound have been deposited in the database. (https://www.ccdc.cam.ac.uk/). CCDC deposition number: 2381817. Link to retrieve specific information:

https://www.ccdc.cam.ac.uk/structures/Search?access=referee&ccdc=2381817&Author=Manuel+Plaza+Martinez

6. References

(1) Yamamoto, E.; Gokuden, D.; A. Nagai, Kamachi, T.; Yoshizawa, K.; Hamasaki, A.; Ishida, T.; Tokunaga, M. *Org. Lett.* 2012, **14**, 6178−6181.

(2) Barluenga, J.; Tomás-Gamasa, M.; Aznar, F.; Valdés, C. *Adv. Synth. Catal.* 2010, **325**, 3235-3240.

(3) Plaza, M.; Parisotto, S.; Valdés, C. *Chem. Eur. J.*, 2018, **24**, 14836.

(4) William, G. D.; Milton, E. L.; Noel, D. V.; Robert, H. S.; Duncan, J. H.; Tomer, K. *J. Am. Chem. Soc.* 1968, **90**, 4762–4763.

(5) Bunnell, C. A.; Fuchs, P. L. *J. Org. Chem.* 1977, **42**, 2614-2617.

(6) Boruah, A.; Baruah, B.; Prajapati, D.; Sandhu, J. S. *Synlett*, 1997, **11**, 1251-1252.

(7) M. C. Pérez-Aguilar, C. Valdés, *Angew. Chem. Int. Ed.* 2012, **51**, 5953–5957.

(8) L. H. B. Baptistella, A. M. Aleixo, *Synthetic Communications,* 2002, **32**, 2937-2950.

(9) L. López, M. Cabal, C. Valdés, *Angew. Chem. Int. Ed.* 2022, **61**, *e202113370.*

(10) T. Zhang, Y. Meng, J. Lu, Y. Yang, G-Q. Li, C. Zhu. *Adv Synth Catal.* 2018, **360**, 3063–3068.

(11) a) K. G. Hancock, J. D.Kramer, *J. Am. Chem. Soc.* 1973, **95**, 6463; b) F. W. van der Mei, H. Miyamoto, D. L. Silverio, A. H. Hoveyda, *Angew. Chem. Int. Ed.* 2016, **55**, 4701.

(12) M. M. Boucher, M. H. Furigay, P. K. Quach, C. S. Brindle, *Org. Proc. Res. Dev*. 2017, **21**, 1394−1403

7. Copies of NMR Spectra

6-allyl-4,4-dimethylcyclohex-2-en-1-one (14)

^{1}H NMR (400 MHz, $CDCl_3$)

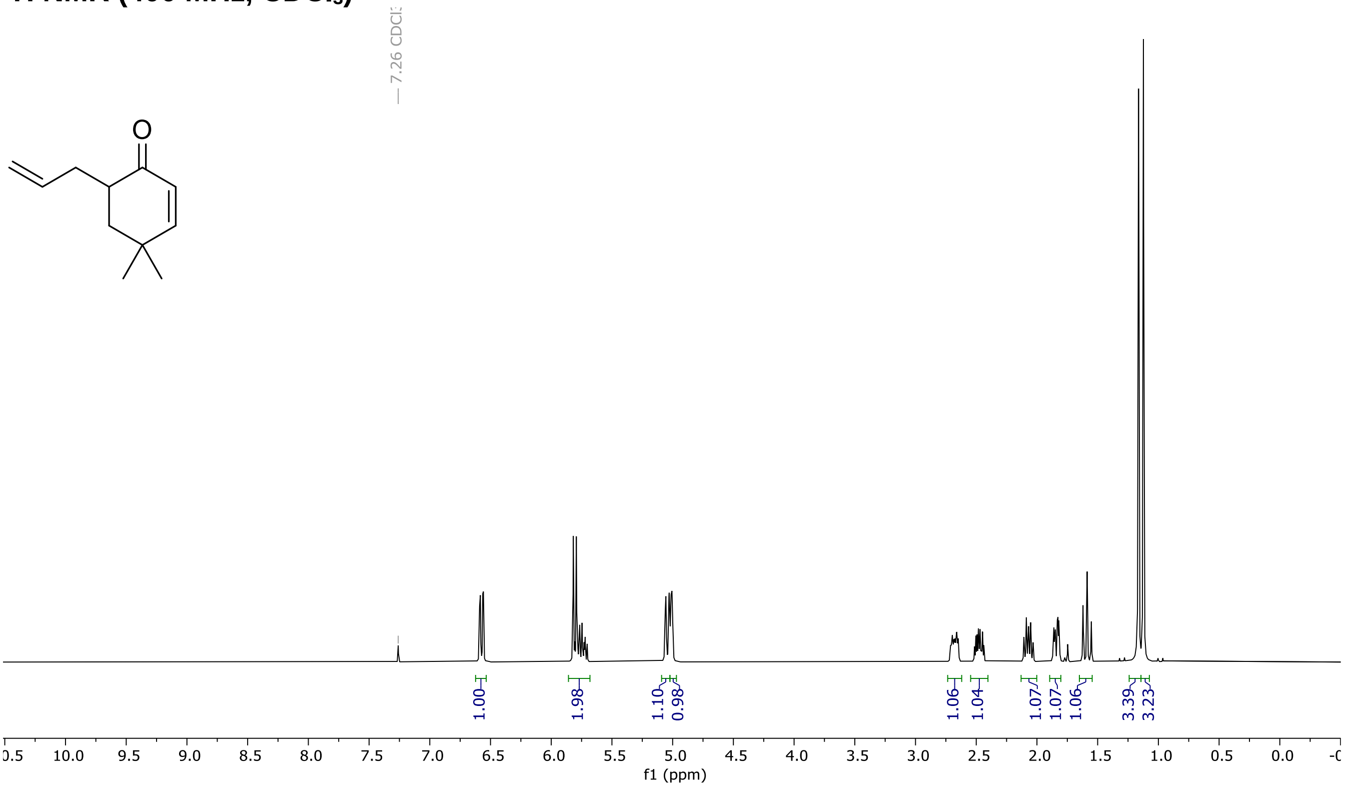

^{13}C NMR (101 MHz, $CDCl_3$)

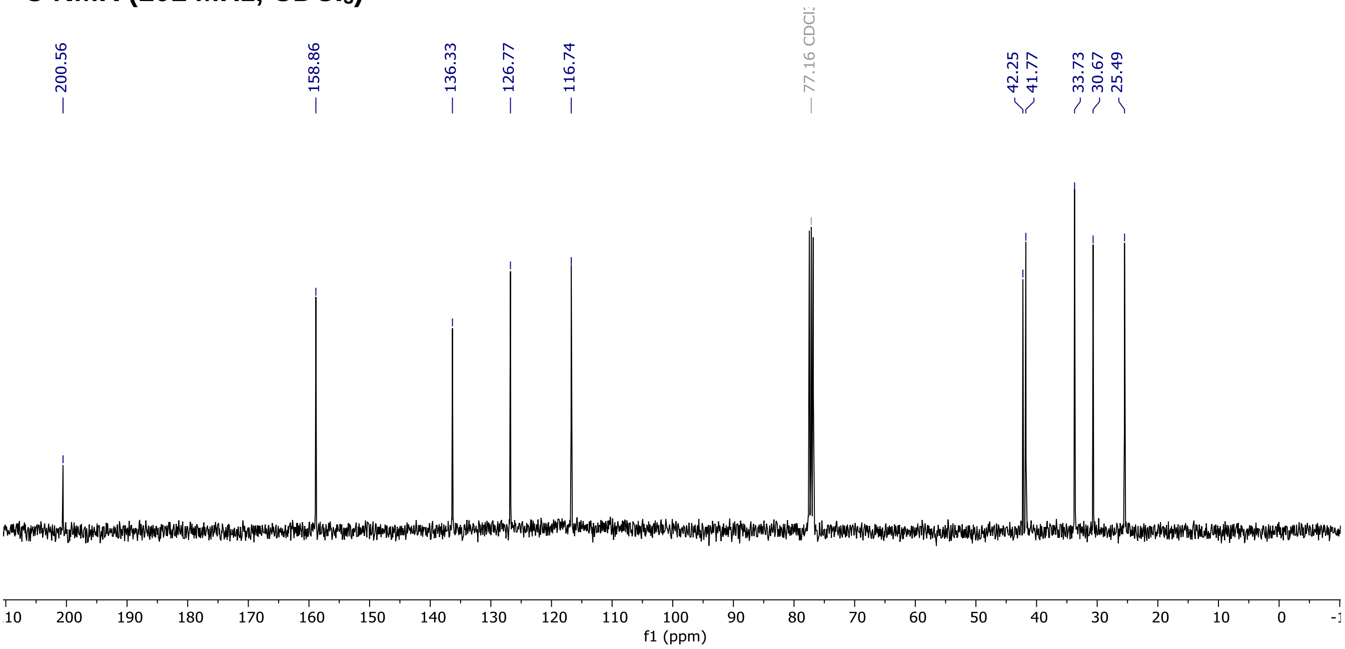

4-methyl-N'-((1R,5R,E)-4,6,6-trimethylbicyclo[3.1.1]hept-3-en-2-ylidene)benzenesulfonohydrazide (1j)

^{1}H NMR (300 MHz, $CDCl_3$)

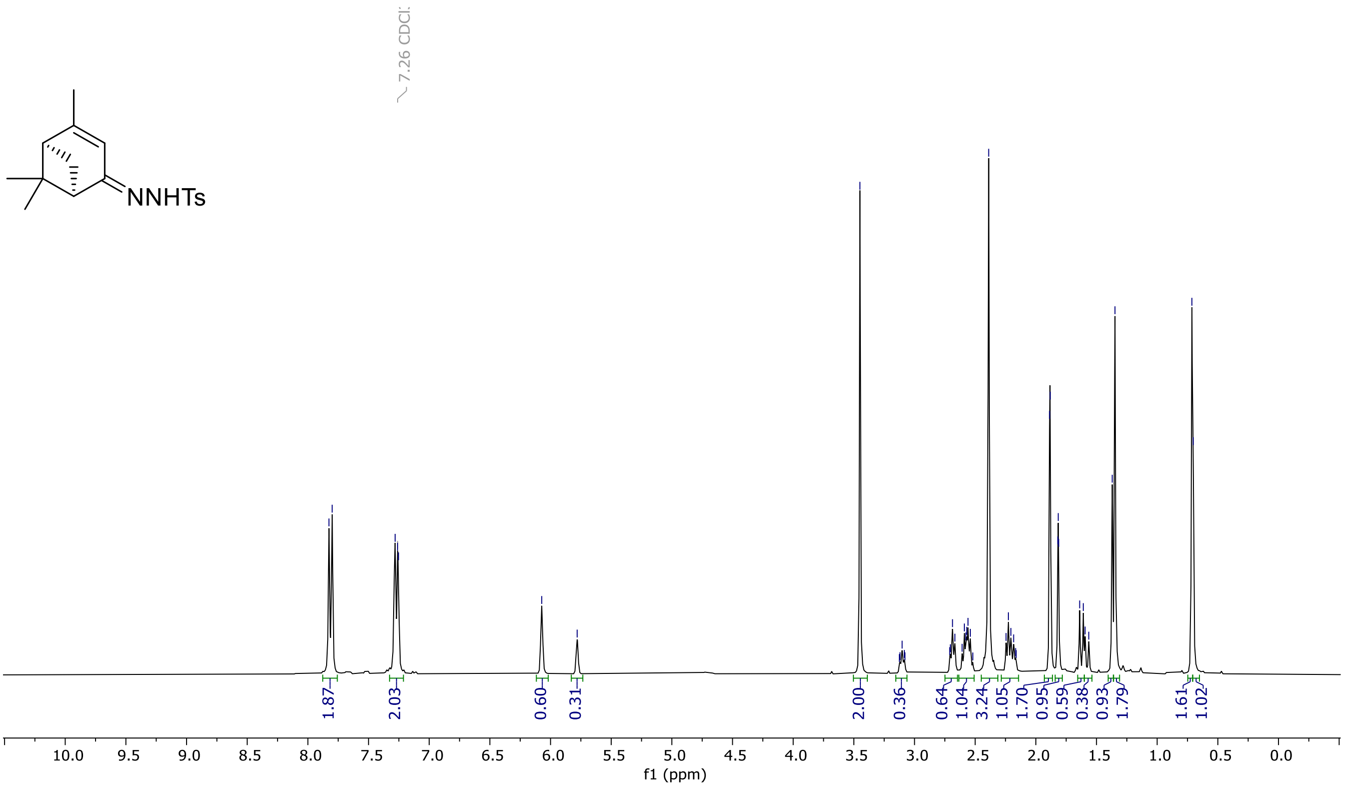

^{13}C NMR (75 MHz, $CDCl_3$)

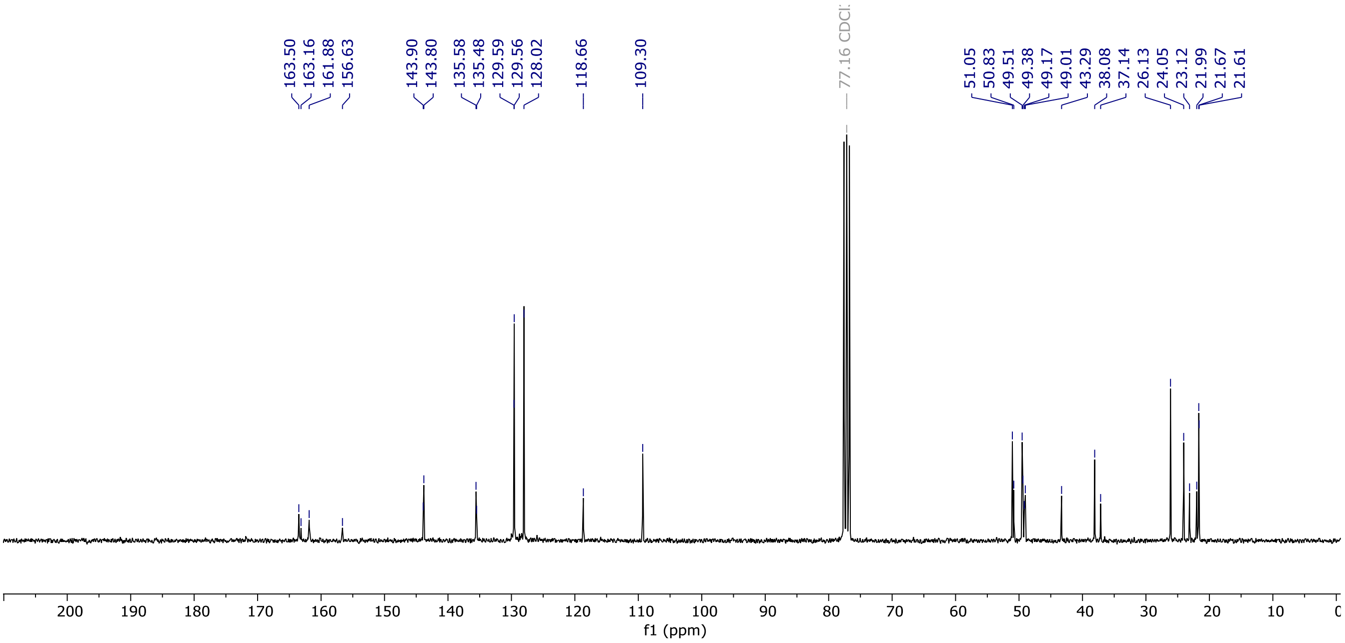

(E)-N'-(6-allyl-4,4-dimethylcyclohex-2-en-1-ylidene)-4-methylbenzenesulfonohydrazide (15)

^{1}H NMR (300 MHz, $CDCl_3$)

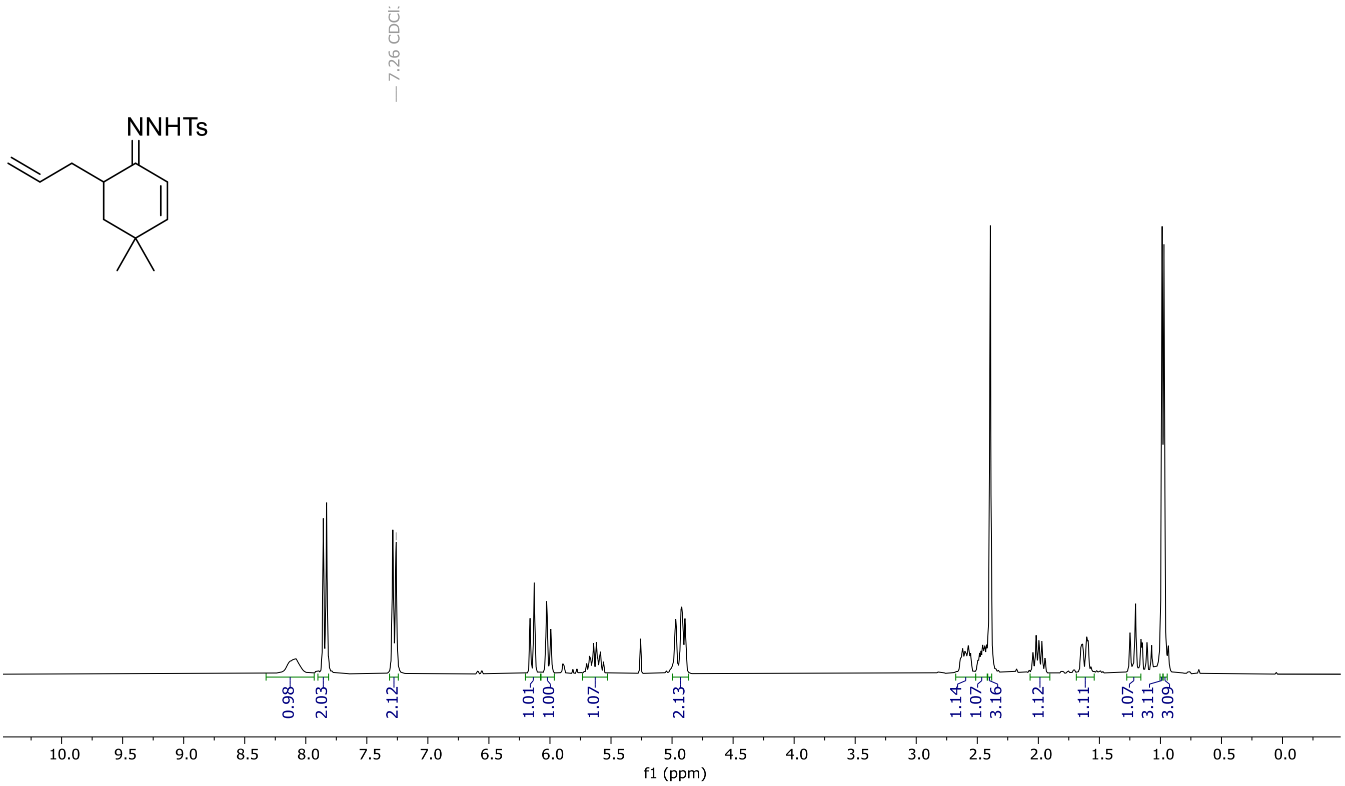

^{13}C NMR (75 MHz, $CDCl_3$)

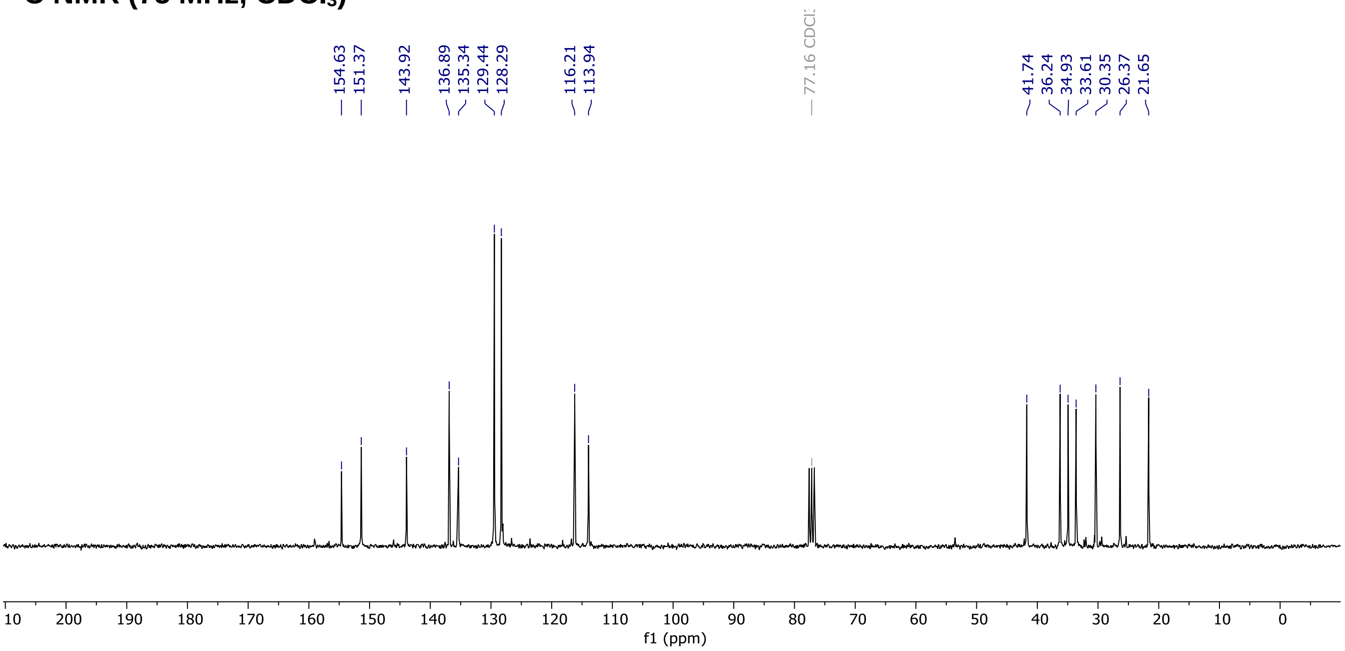

2-(4,4-dimethyl-1-propylcyclohex-2-en-1-yl)-4,4,5,5-tetramethyl-1,3,2-dioxaborolane (4a)

^{1}H NMR (300 MHz, CDCl$_3$)

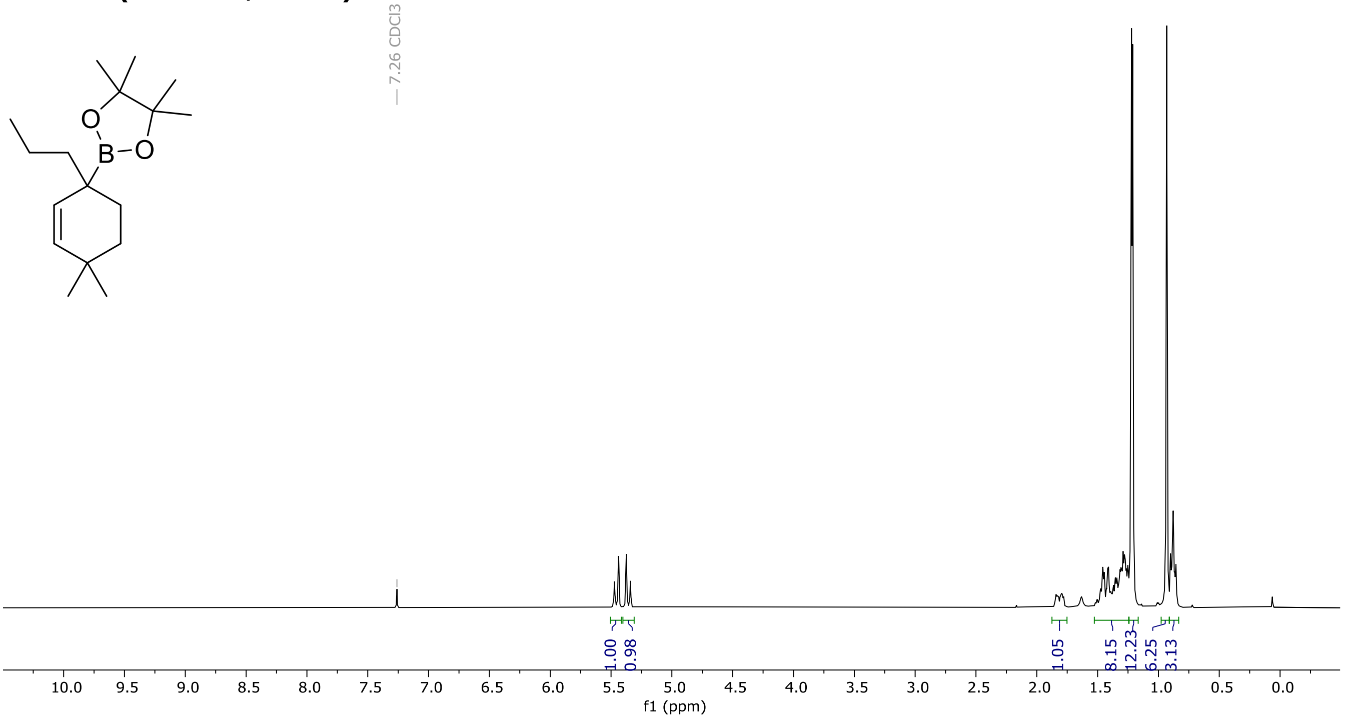

^{13}C NMR (75 MHz, CDCl$_3$)

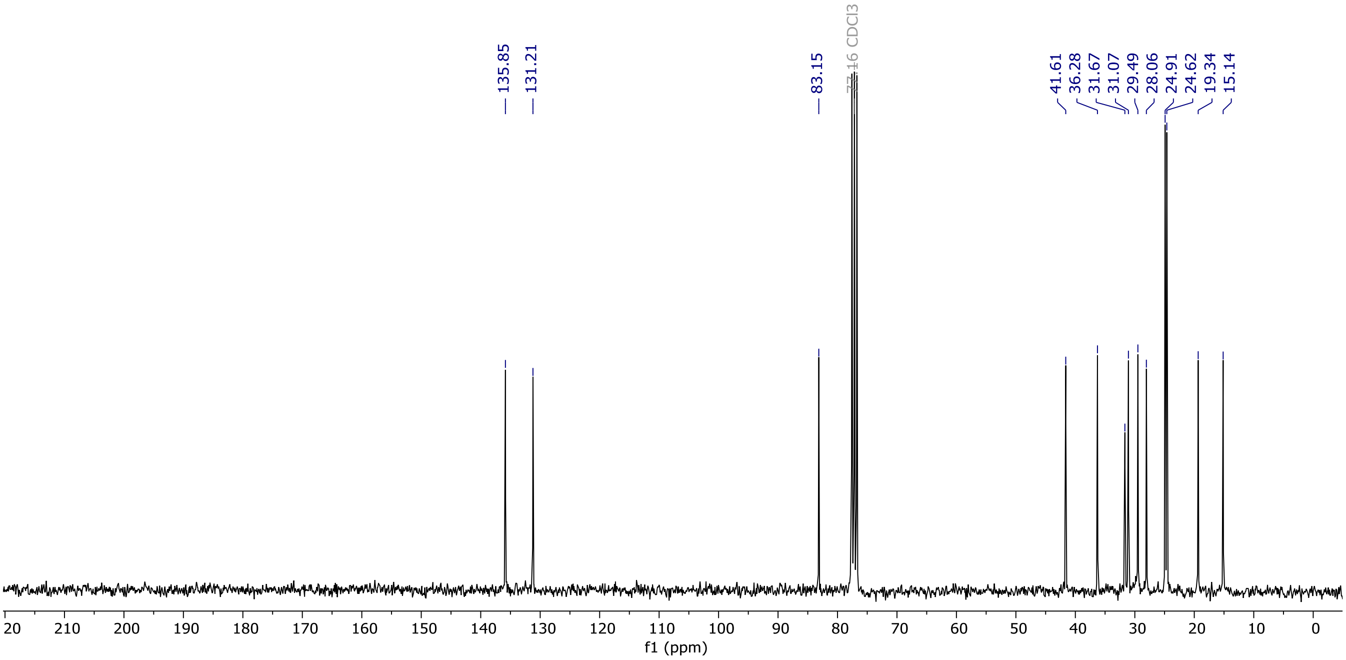

^{11}B NMR (129 MHz, CDCl$_3$)

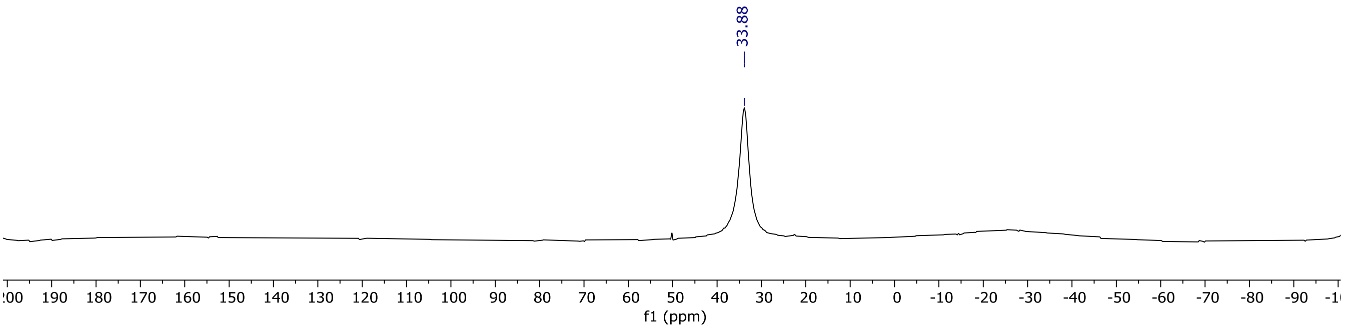

4,4,5,5-tetramethyl-2-(1,4,4-trimethylcyclohex-2-en-1-yl)-1,3,2-dioxaborolane (4b)

^{1}H NMR (300 MHz, $CDCl_3$)

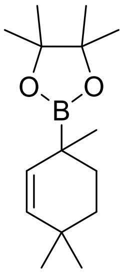

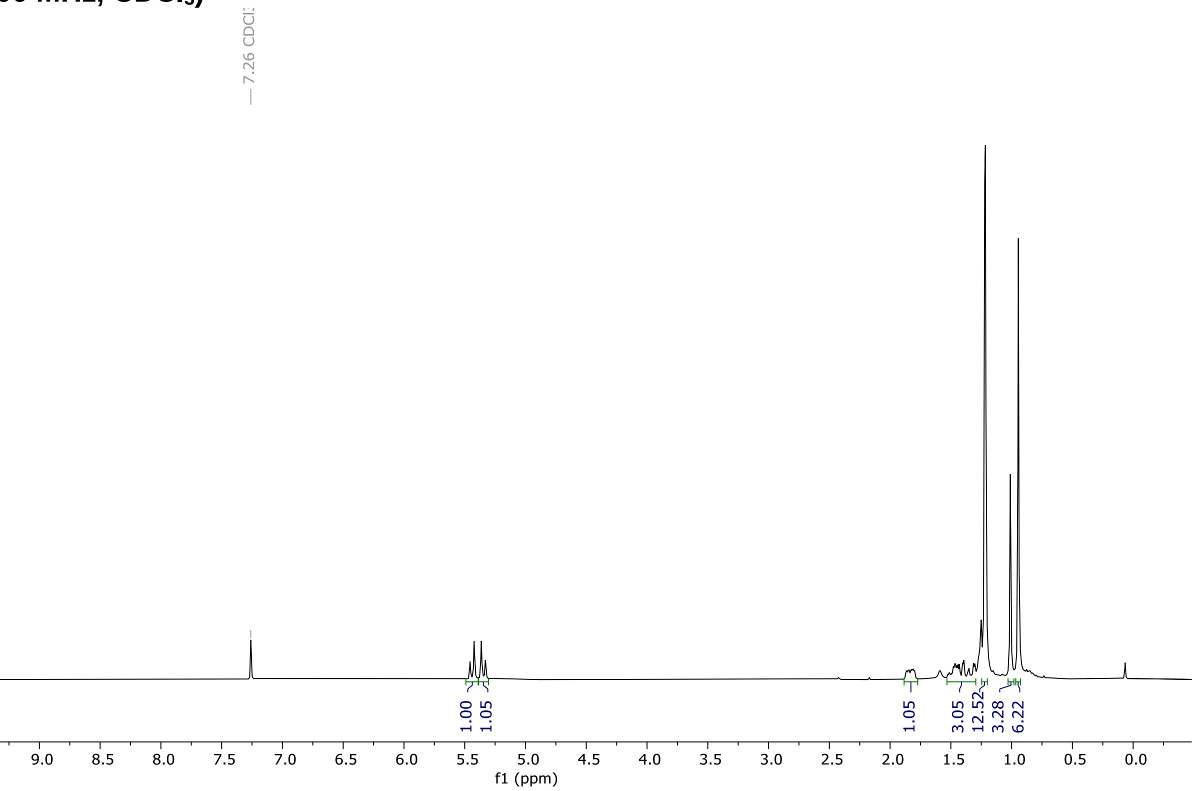

^{13}C NMR (75 MHz, $CDCl_3$)

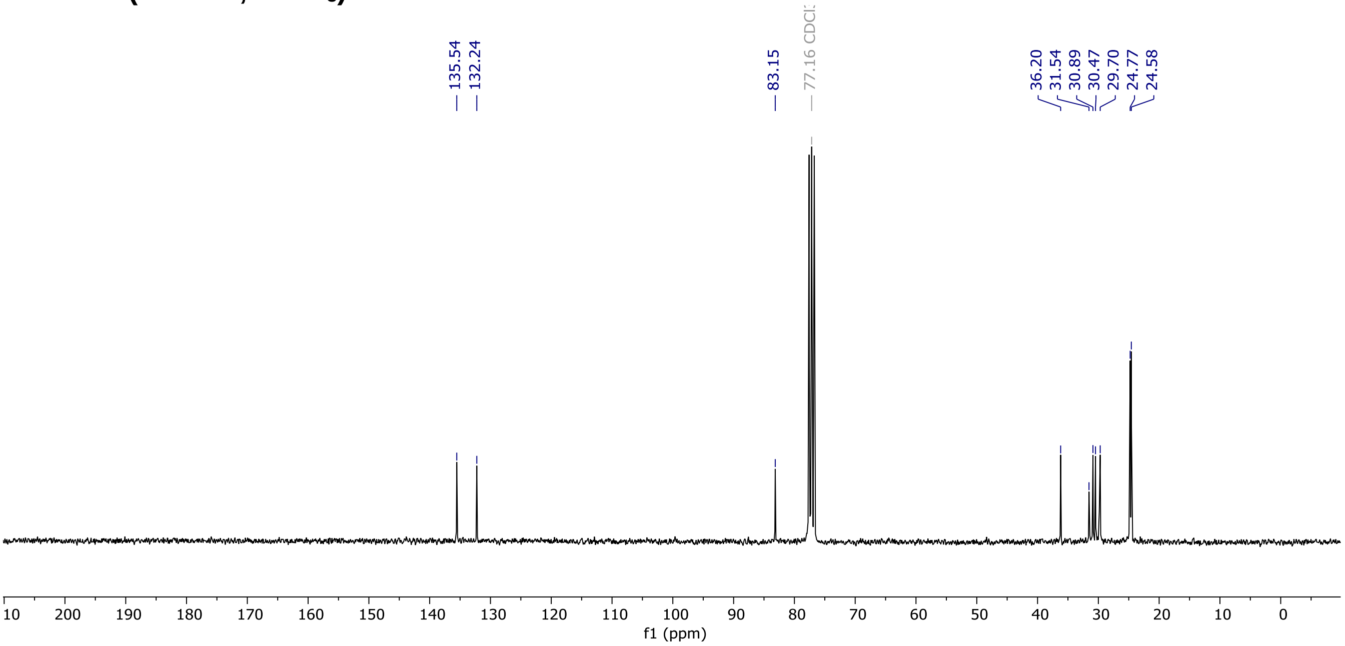

^{11}B NMR (129 MHz, $CDCl_3$)

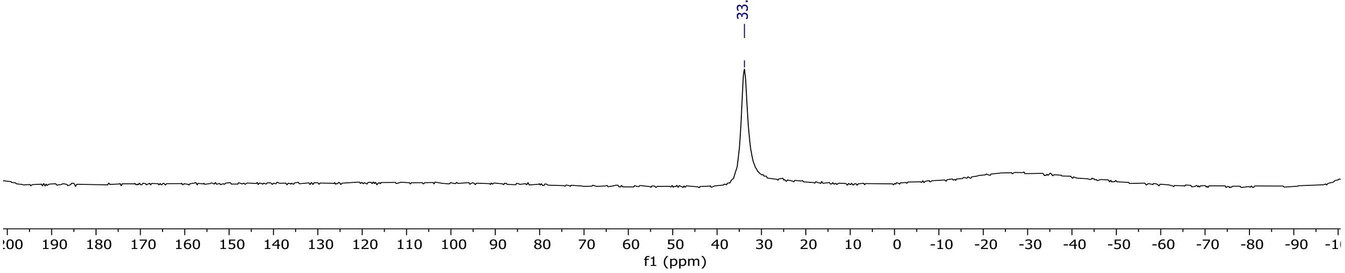

2-(4,4-dimethyl-1-phenethylcyclohex-2-en-1-yl)-4,4,5,5-tetramethyl-1,3,2-dioxaborolane (4c)

^{1}H NMR (300 MHz, $CDCl_3$)

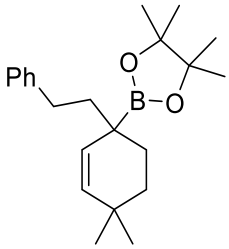

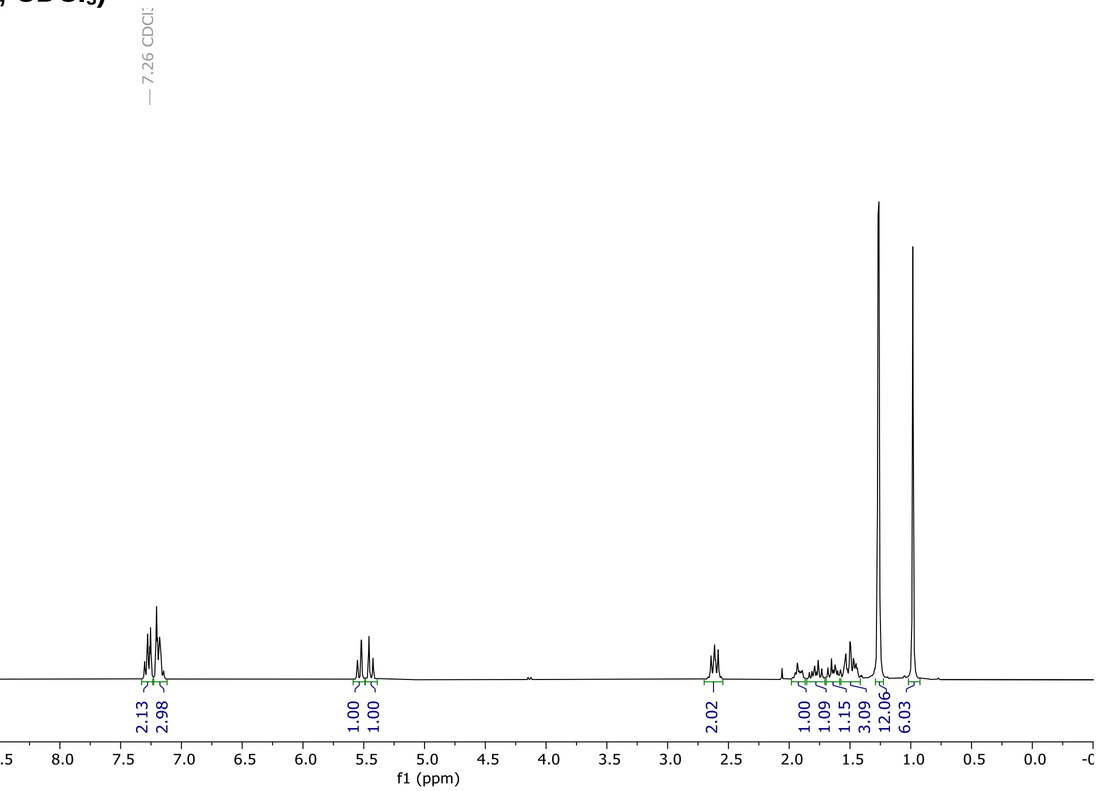

^{13}C NMR (75 MHz, $CDCl_3$)

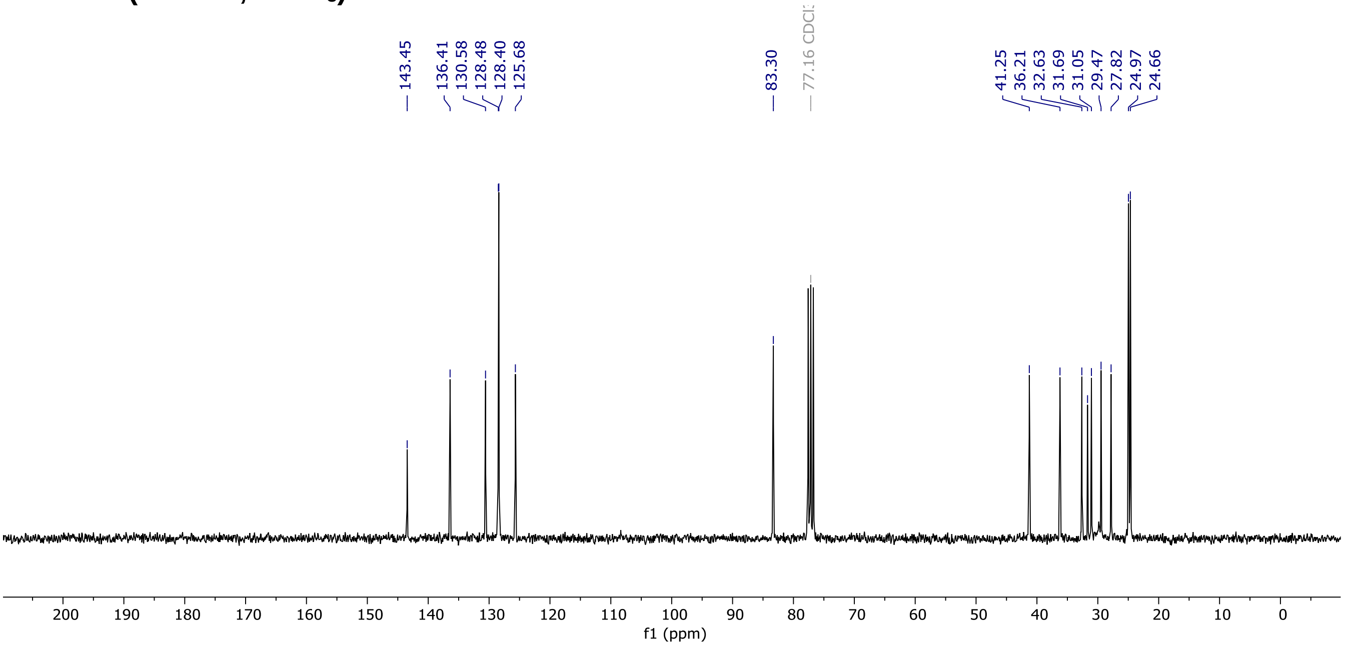

^{11}B NMR (129 MHz, $CDCl_3$)

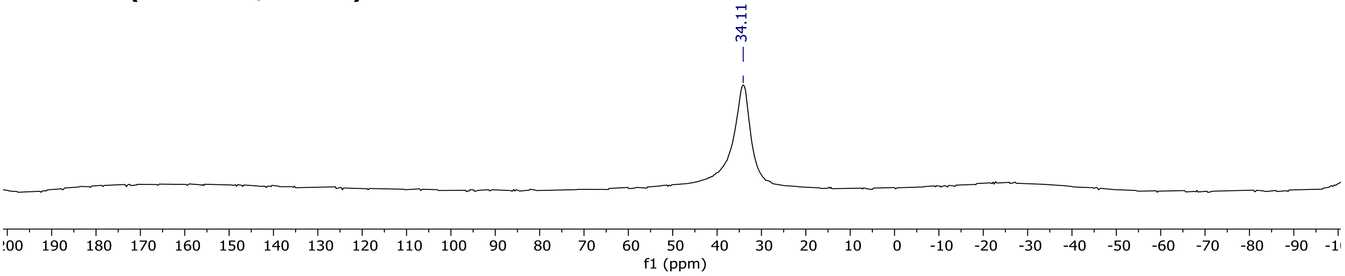

2-(1-(3-bromopropyl)-4,4-dimethylcyclohex-2-en-1-yl)-4,4,5,5-tetramethyl-1,3,2-dioxaborolane (4d)

^{1}H NMR (300 MHz, $CDCl_3$)

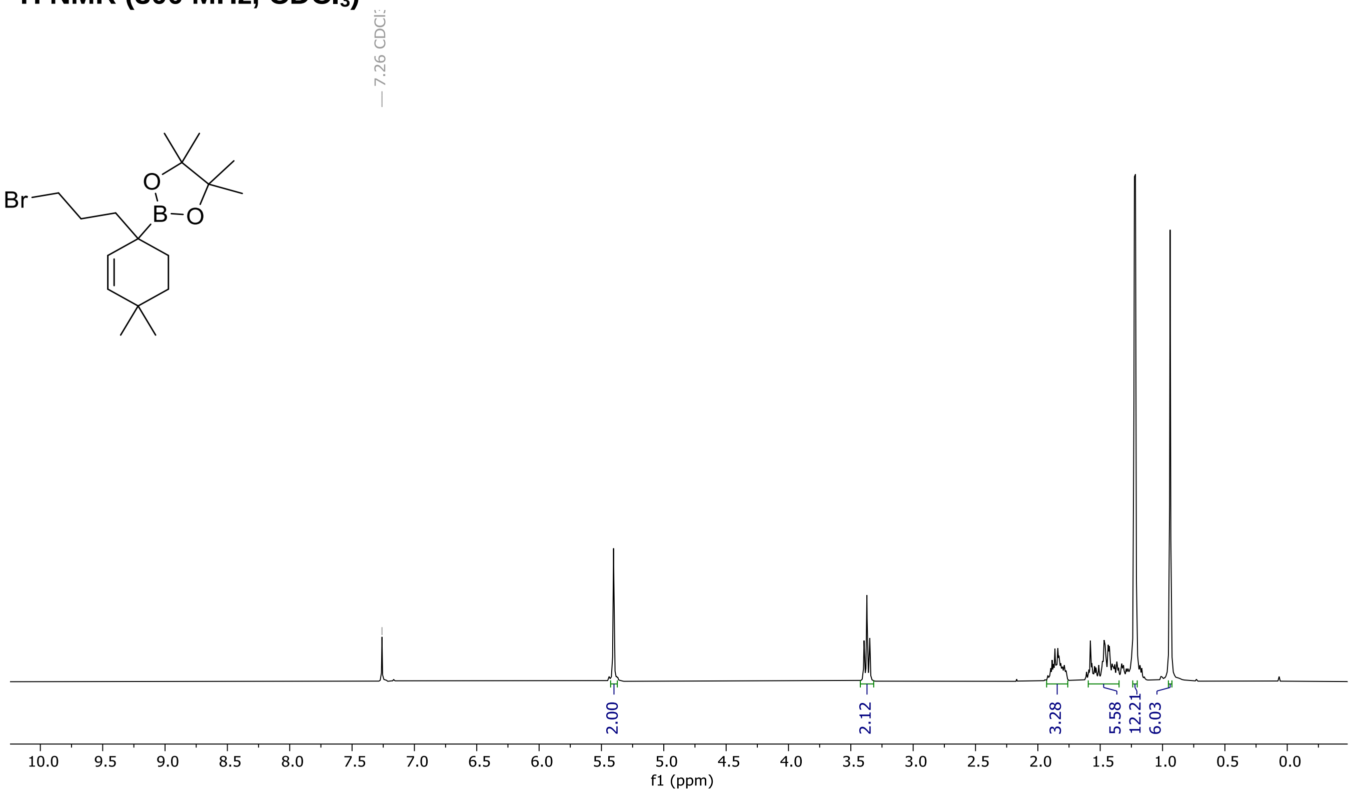

^{13}C NMR (75 MHz, $CDCl_3$)

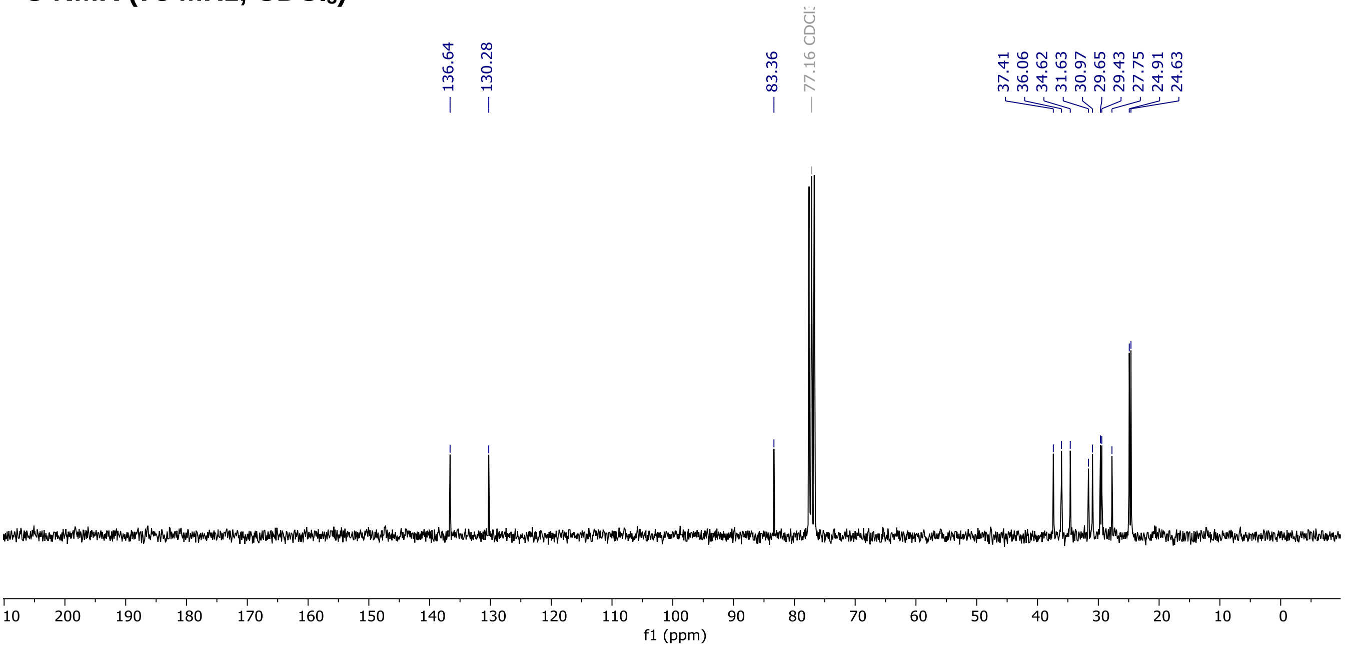

^{11}B NMR (129 MHz, $CDCl_3$)

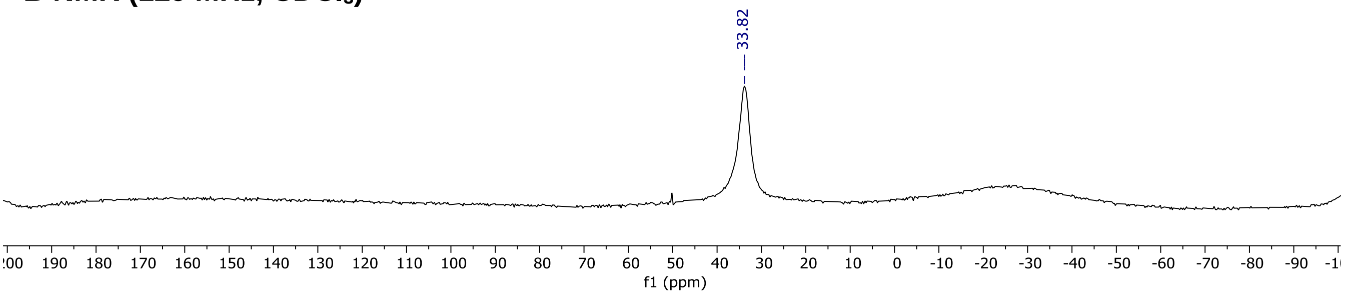

6-(4,4-dimethyl-1-(4,4,5,5-tetramethyl-1,3,2-dioxaborolan-2-yl)cyclohex-2-en-1-yl)hexan-2-one (4e)

^{1}H NMR (300 MHz, $CDCl_3$)

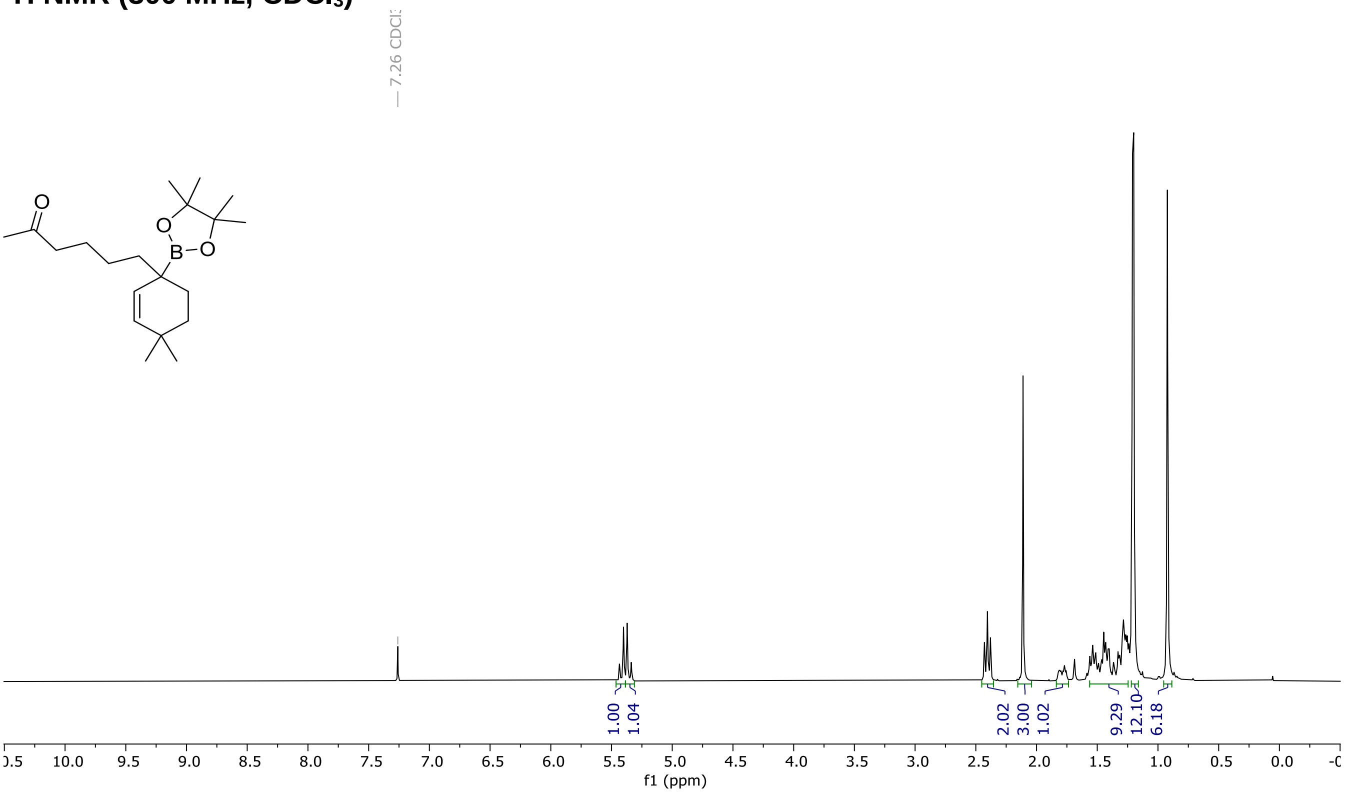

^{13}C NMR (75 MHz, $CDCl_3$)

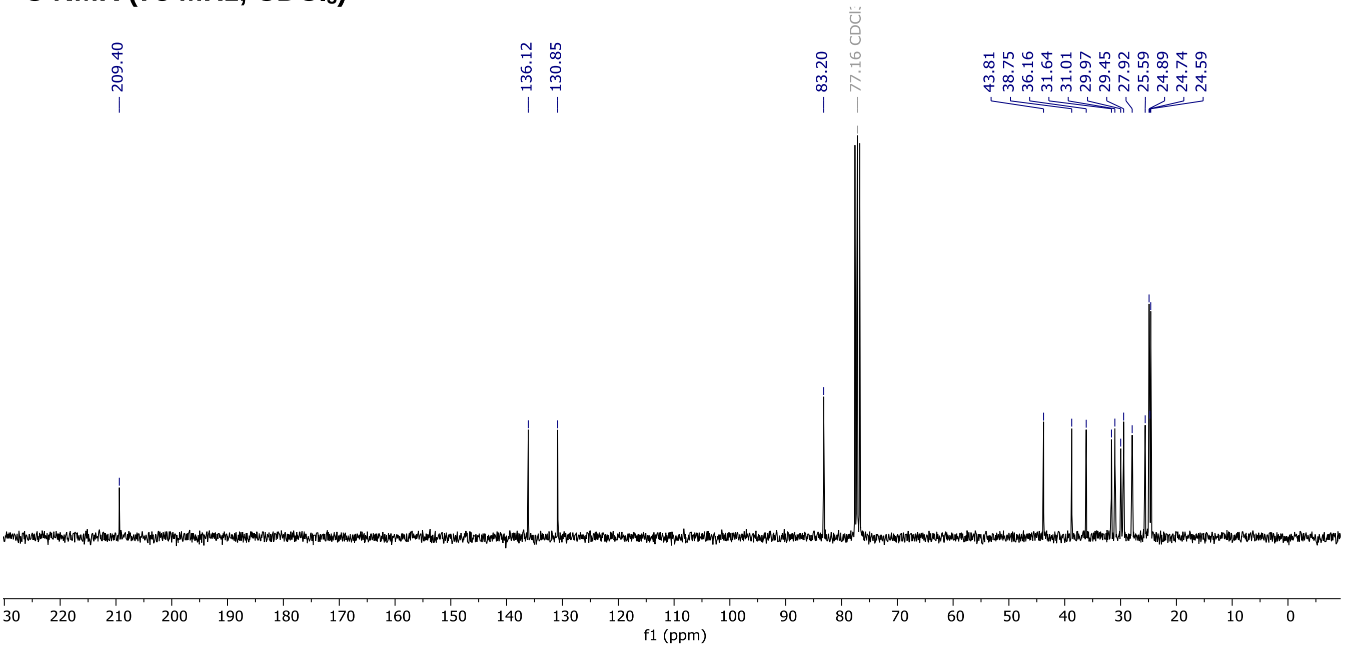

^{11}B NMR (129 MHz, $CDCl_3$)

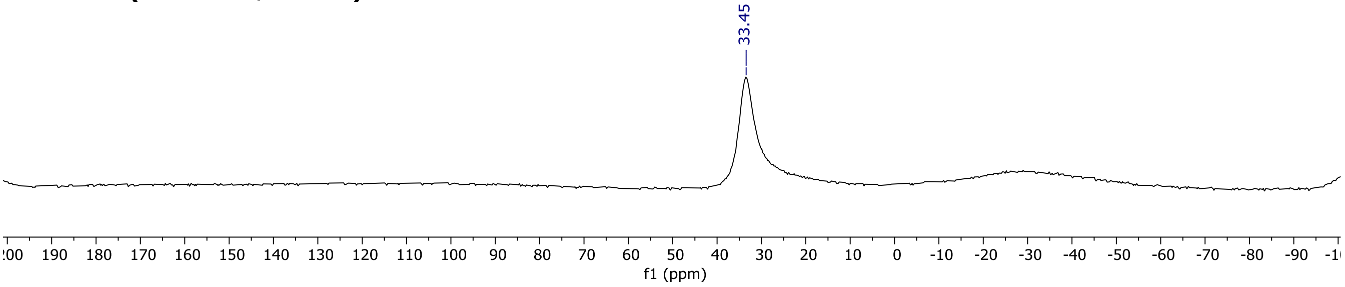

4-(4,4-dimethyl-1-(4,4,5,5-tetramethyl-1,3,2-dioxaborolan-2-yl)cyclohex-2-en-1-yl)butanenitrile (4f)

^{1}H NMR (300 MHz, $CDCl_3$)

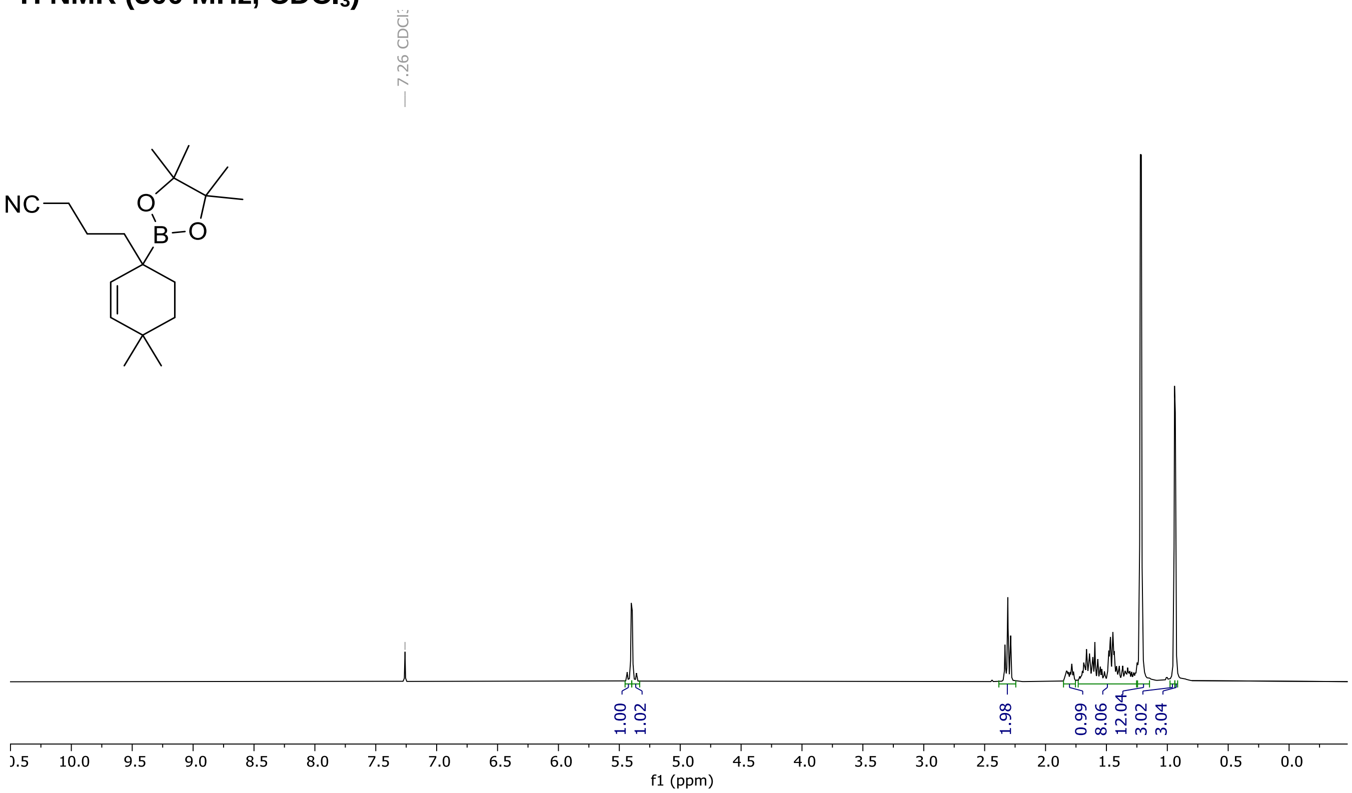

^{13}C NMR (75 MHz, $CDCl_3$)

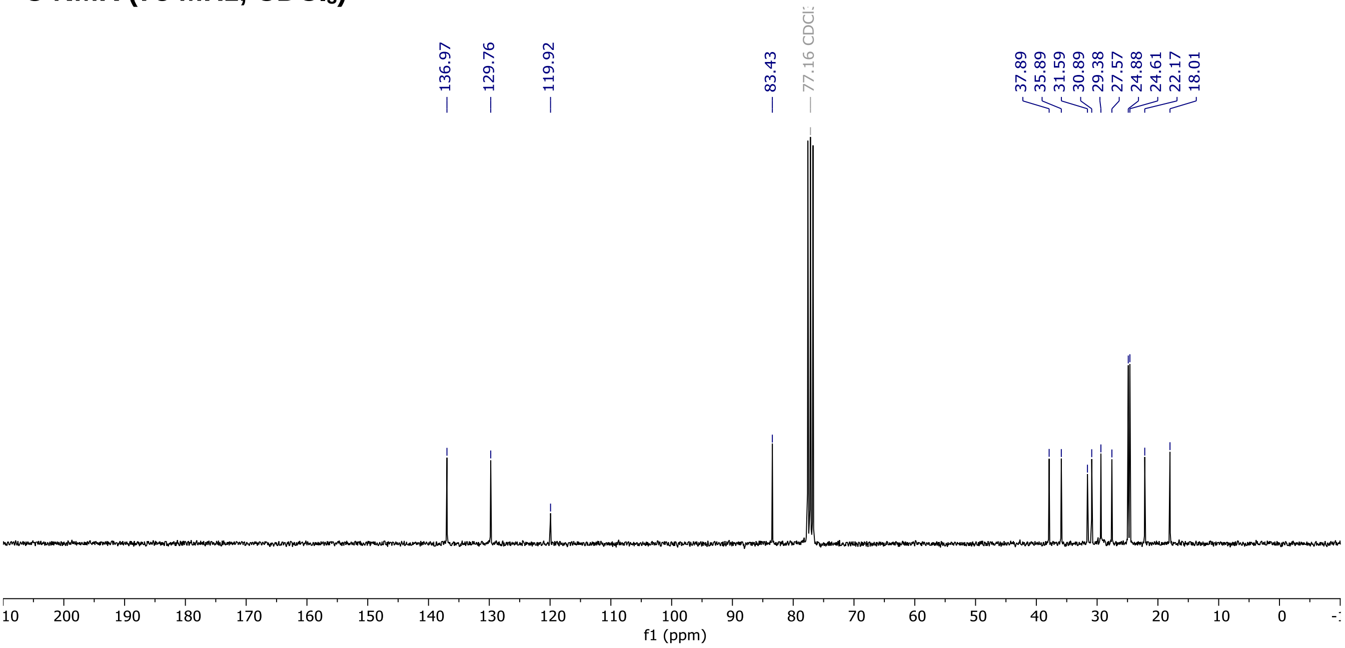

^{11}B NMR (129 MHz, $CDCl_3$)

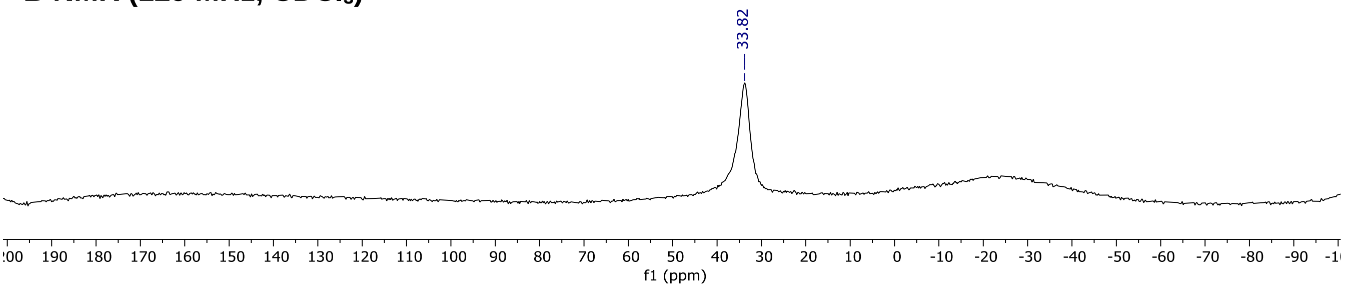

2-(1-(but-3-en-1-yl)-4,4-dimethylcyclohex-2-en-1-yl)-4,4,5,5-tetramethyl-1,3,2-dioxaborolane (4g)

^{1}H NMR (300 MHz, $CDCl_3$)

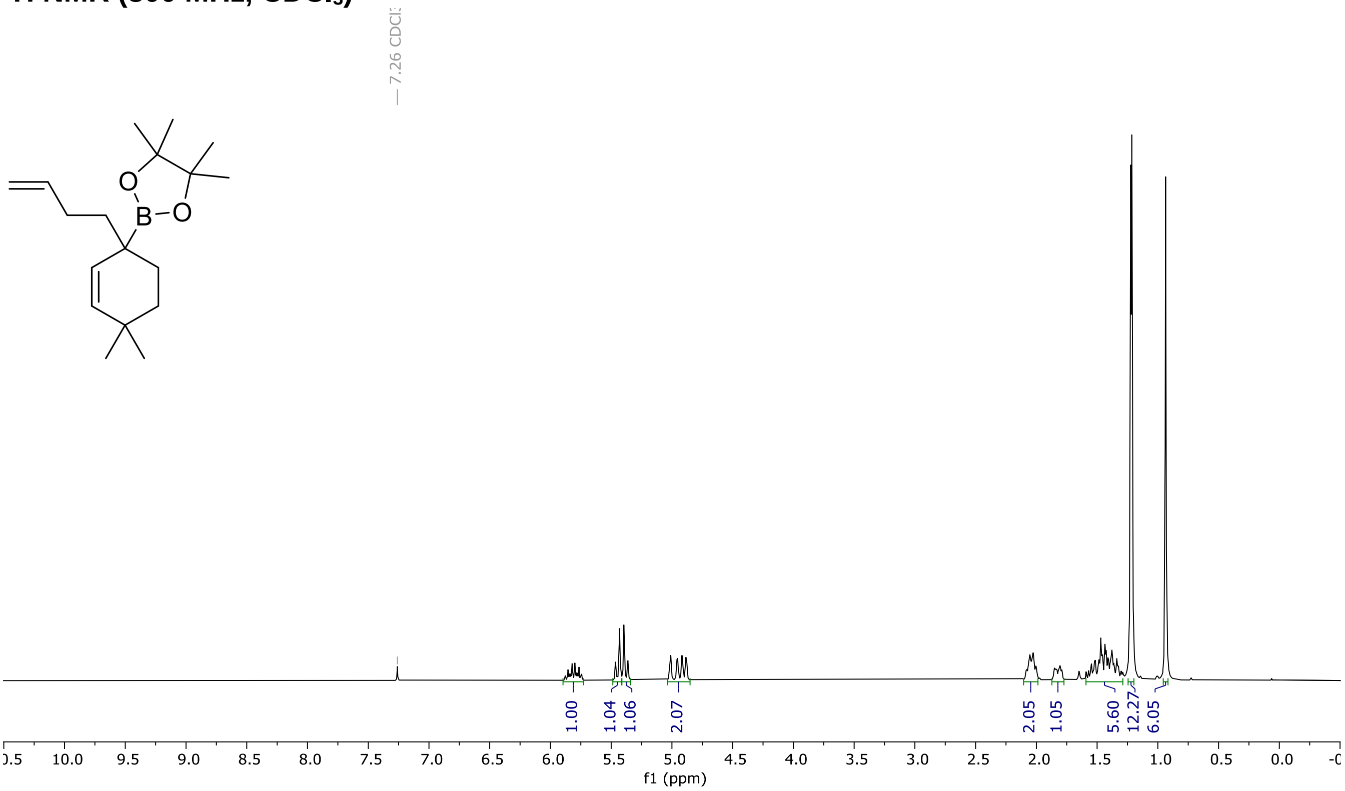

^{13}C NMR (75 MHz, $CDCl_3$)

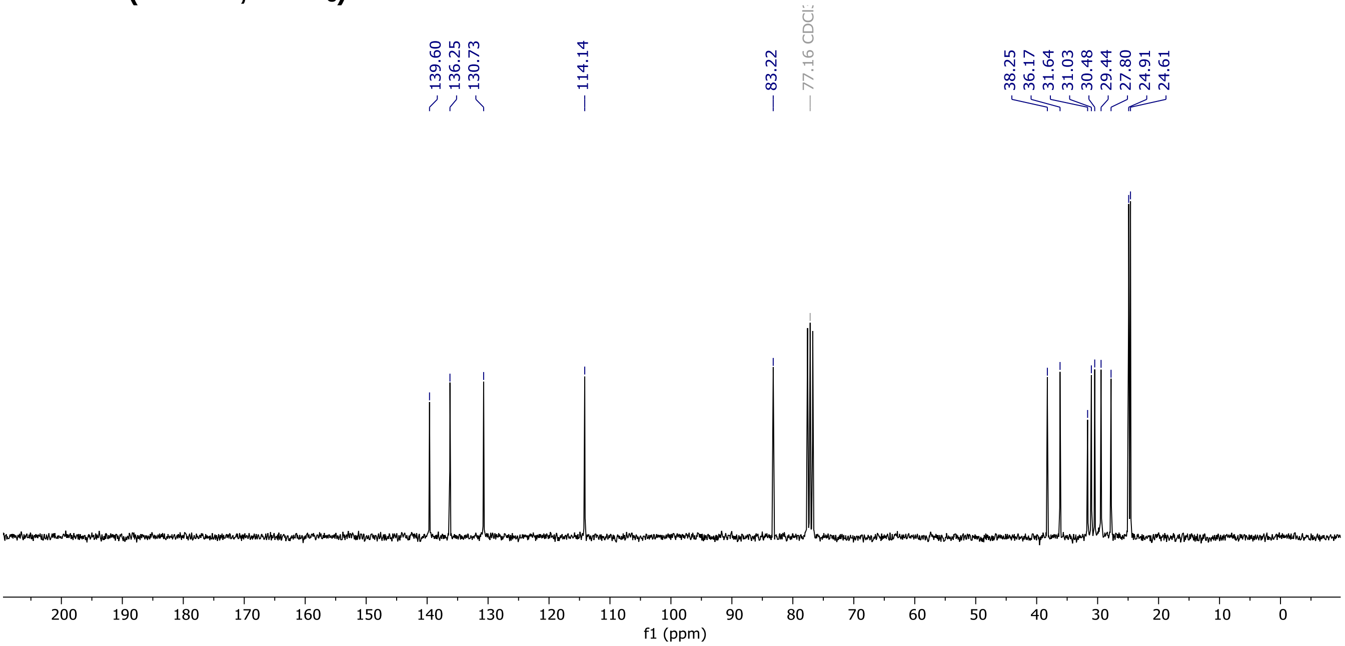

^{11}B NMR (129 MHz, $CDCl_3$)

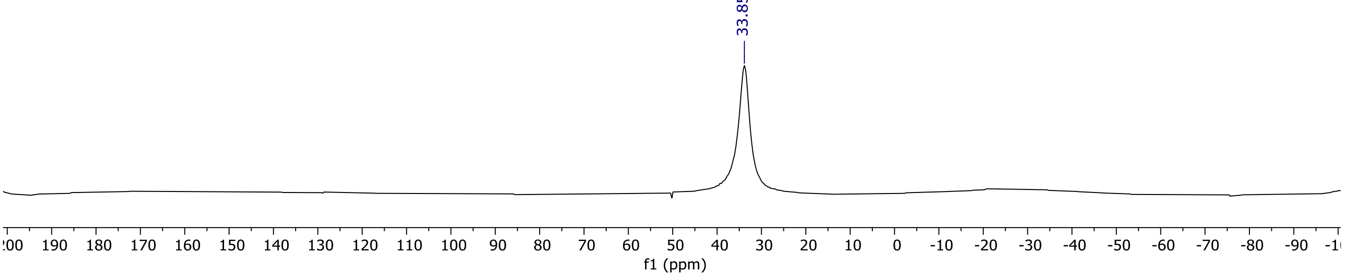

2-(1-cyclopropyl-4,4-dimethylcyclohex-2-en-1-yl)-4,4,5,5-tetramethyl-1,3,2-dioxaborolane (4h)

^{1}H NMR (300 MHz, $CDCl_3$)

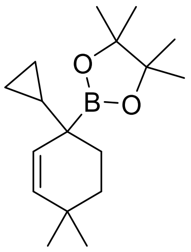

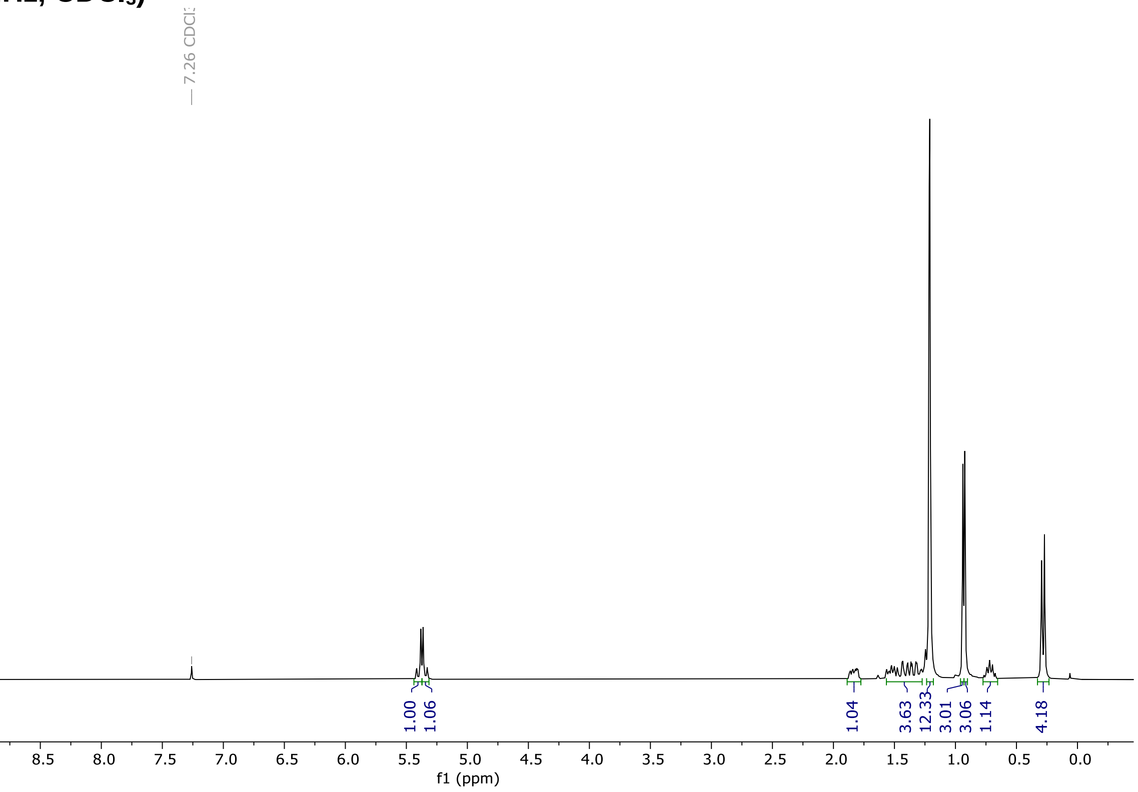

^{13}C NMR (75 MHz, $CDCl_3$)

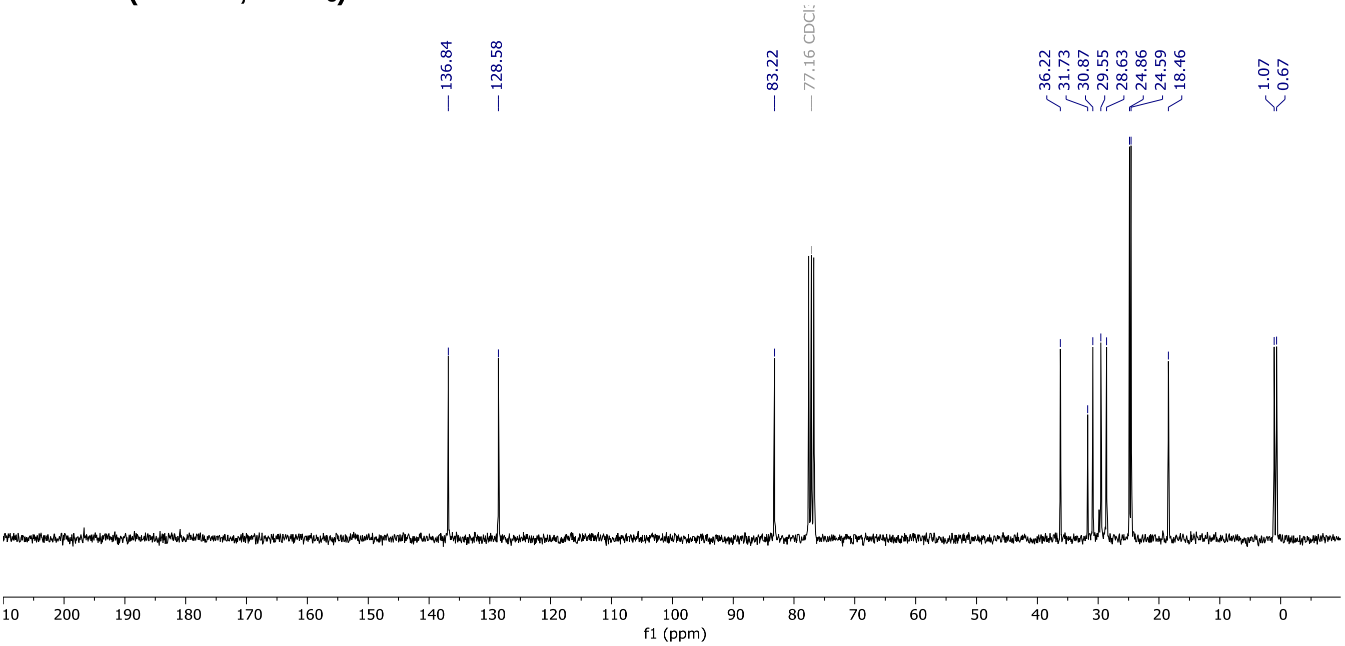

^{11}B NMR (129 MHz, $CDCl_3$)

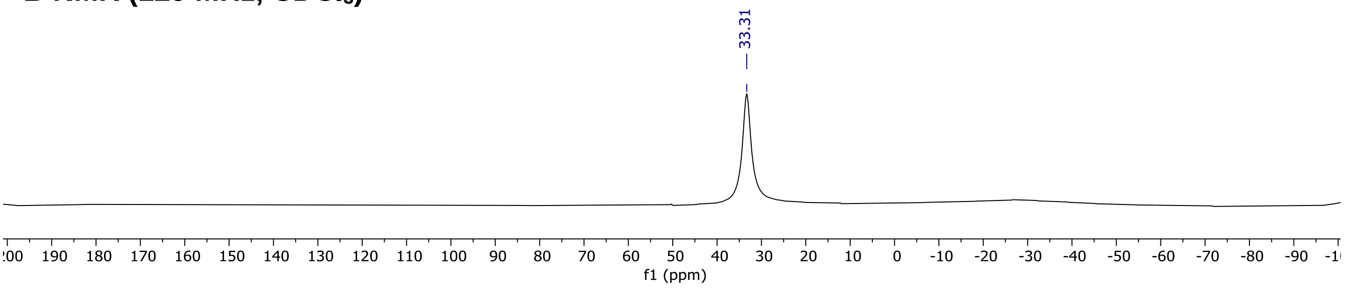

2-(1-cyclobutyl-4,4-dimethylcyclohex-2-en-1-yl)-4,4,5,5-tetramethyl-1,3,2-dioxaborolane (4i)

^{1}H NMR (300 MHz, $CDCl_3$)

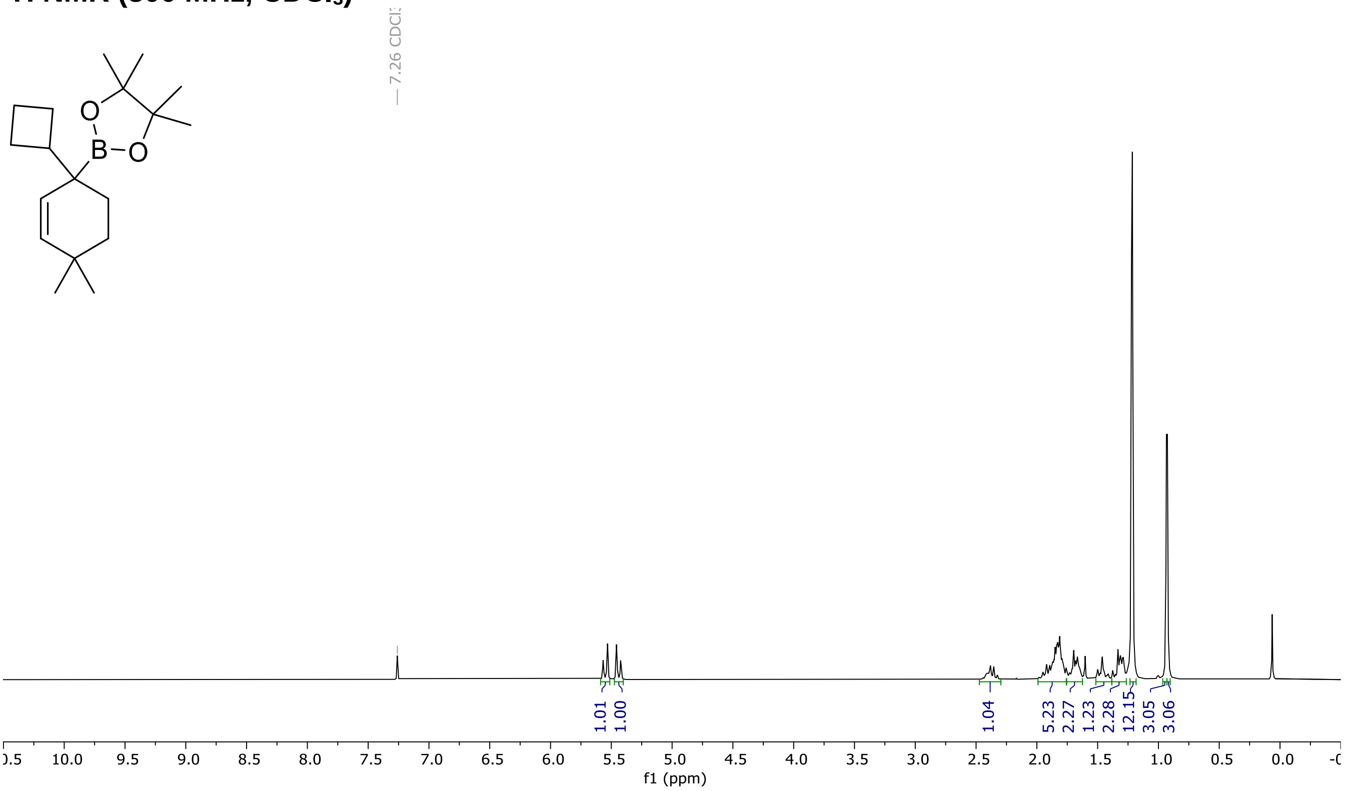

^{13}C NMR (75 MHz, $CDCl_3$)

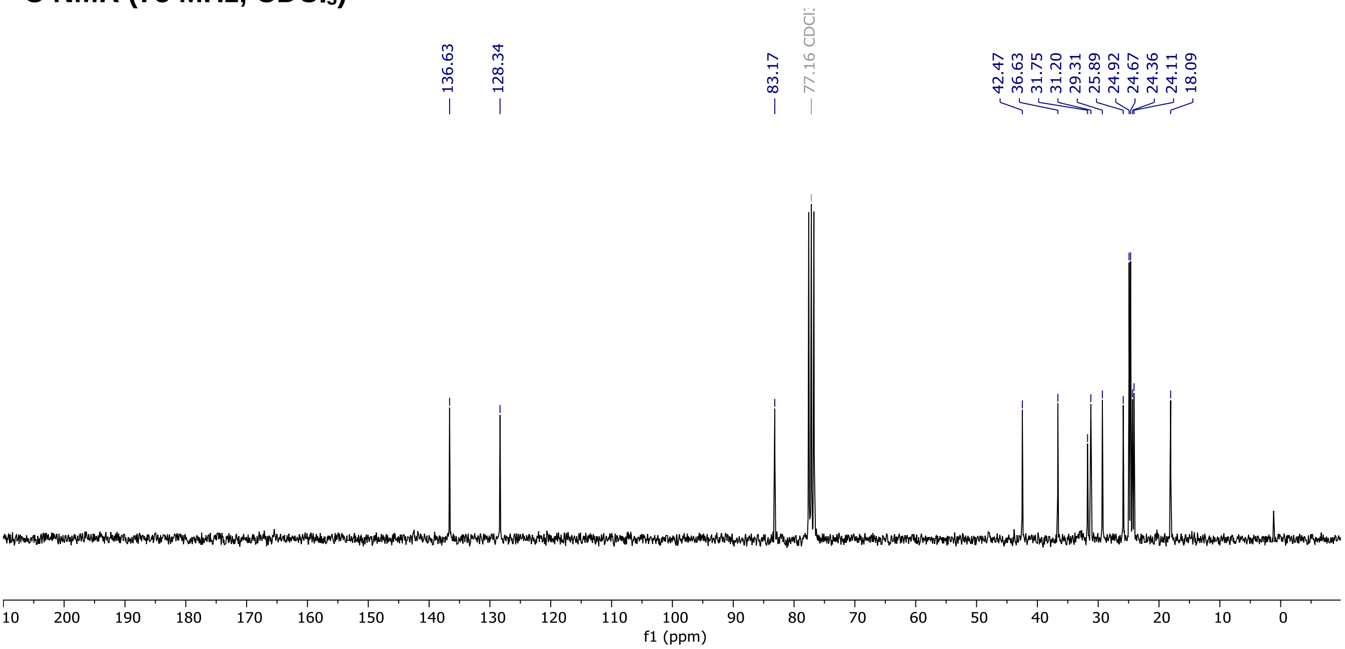

^{11}B NMR (129 MHz, $CDCl_3$)

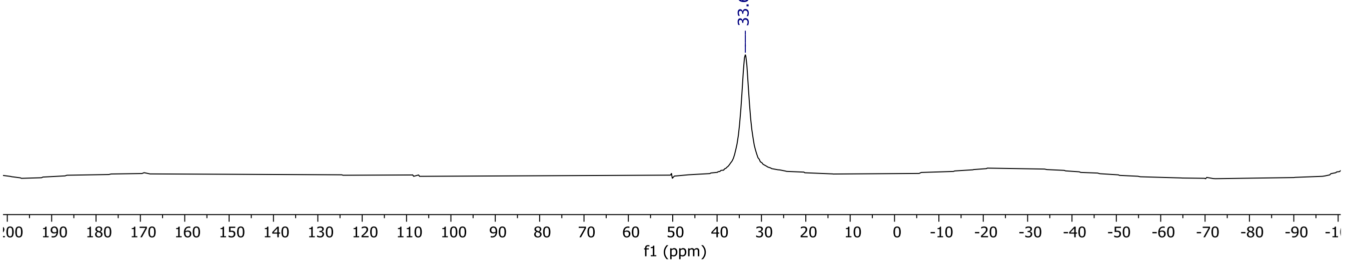

2-(1-cyclopentyl-4,4-dimethylcyclohex-2-en-1-yl)-4,4,5,5-tetramethyl-1,3,2-dioxaborolane (4j)

^{1}H NMR (300 MHz, $CDCl_3$)

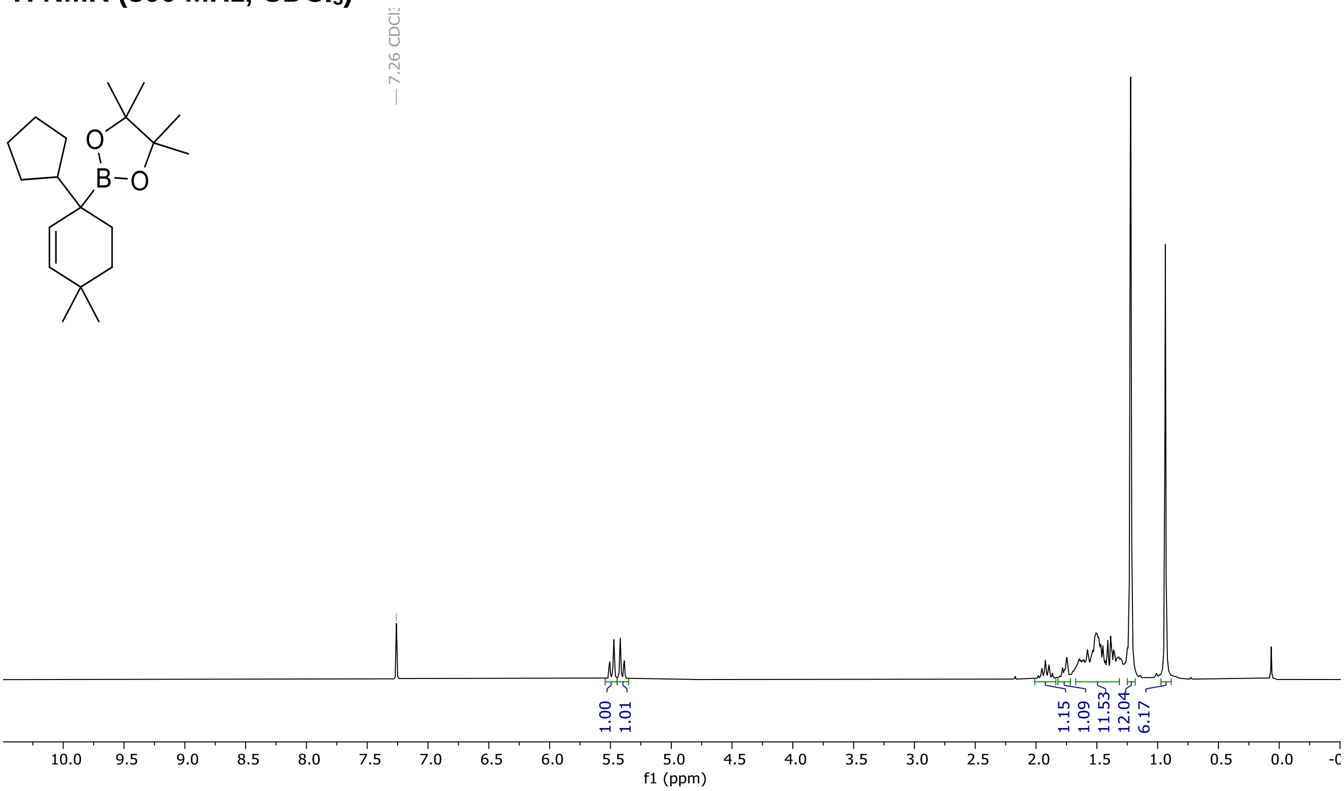

^{13}C NMR (75 MHz, $CDCl_3$)

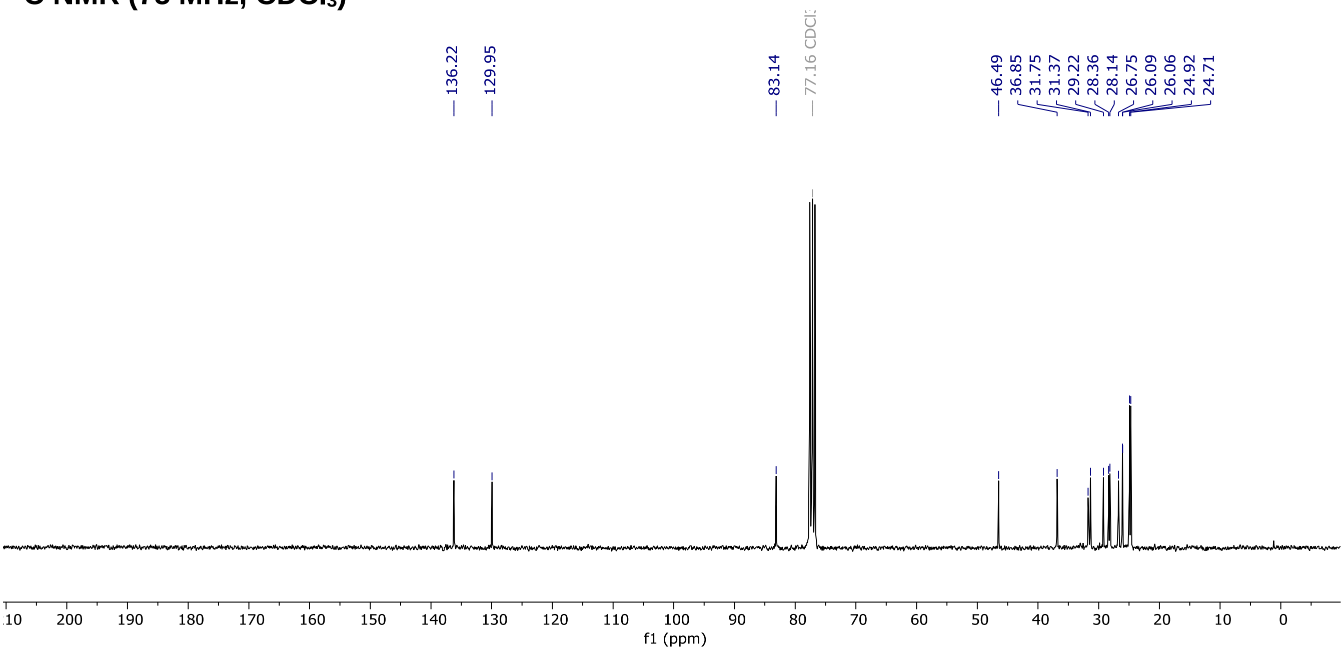

^{11}B NMR (129 MHz, $CDCl_3$)

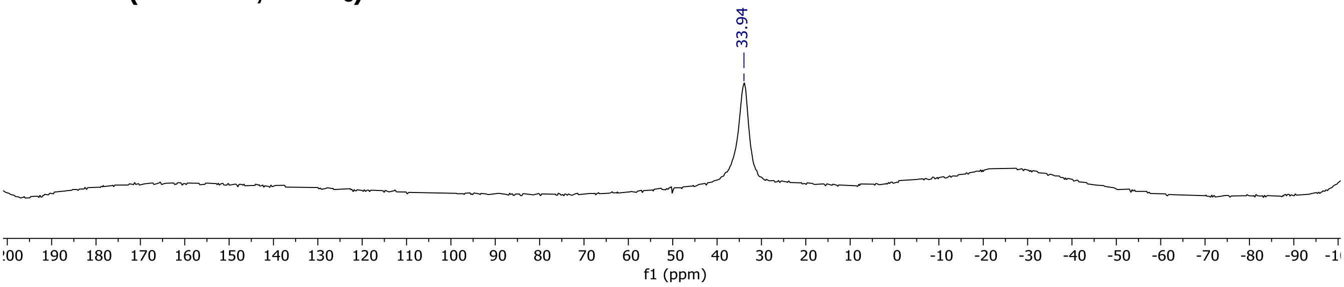

2-(4,4-dimethyl-[1,1'-bi(cyclohexan)]-2-en-1-yl)-4,4,5,5-tetramethyl-1,3,2-dioxaborolane (4k)

^{1}H NMR (300 MHz, $CDCl_3$)

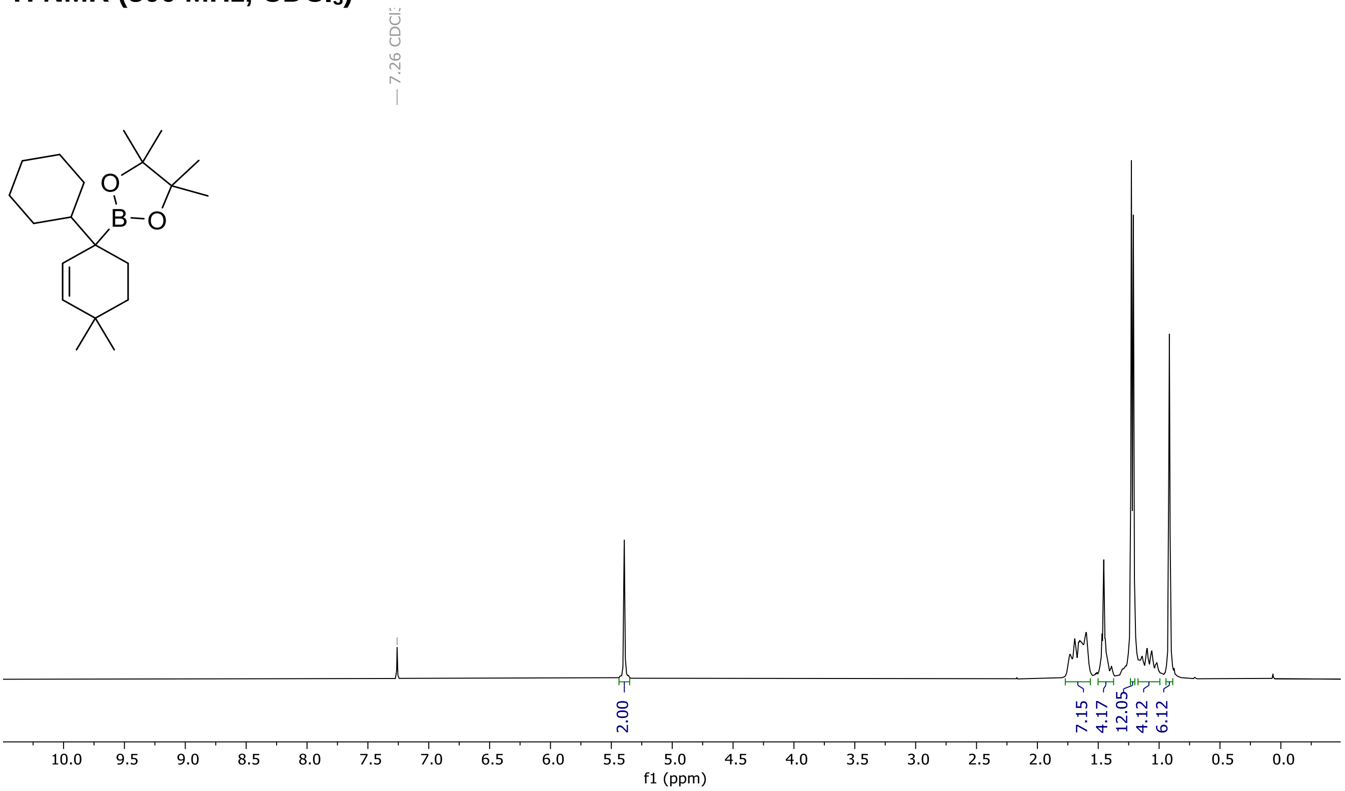

^{13}C NMR (75 MHz, $CDCl_3$)

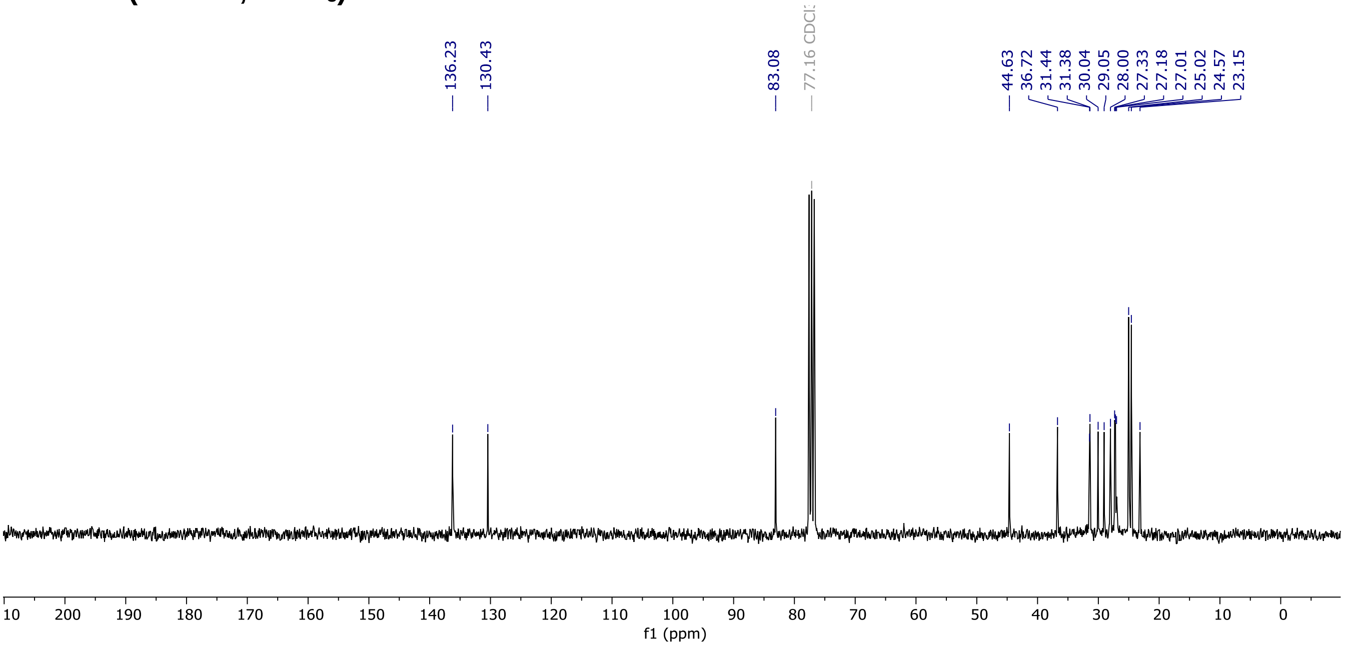

^{11}B NMR (129 MHz, $CDCl_3$)

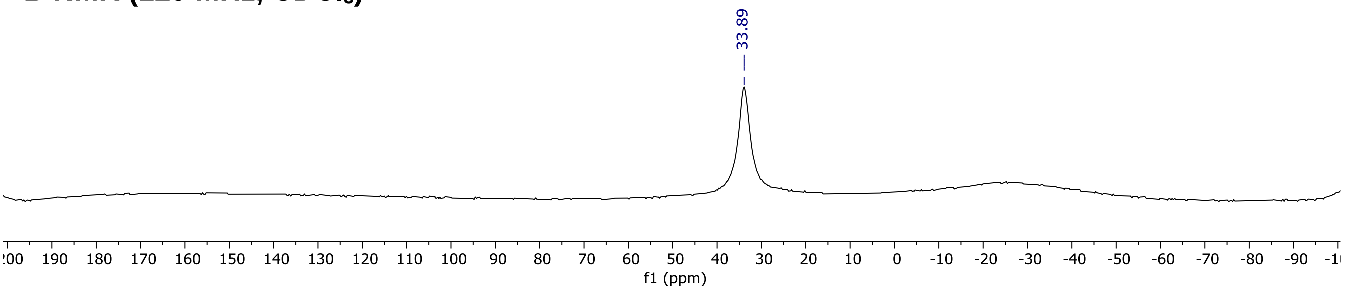

2-(4,4-diethyl-1-propylcyclohex-2-en-1-yl)-4,4,5,5-tetramethyl-1,3,2-dioxaborolane (4l)

^{1}H NMR (300 MHz, $CDCl_3$)

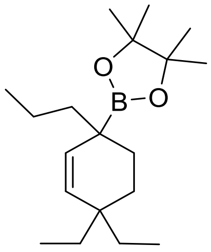

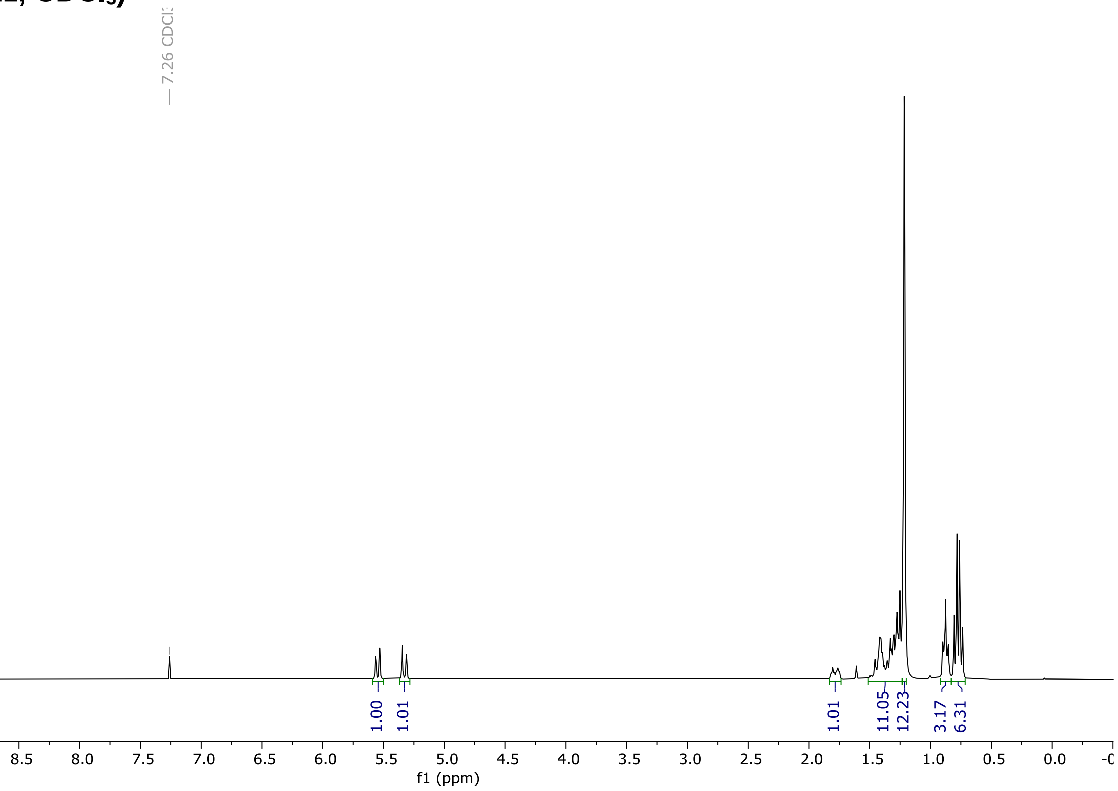

^{13}C NMR (75 MHz, $CDCl_3$)

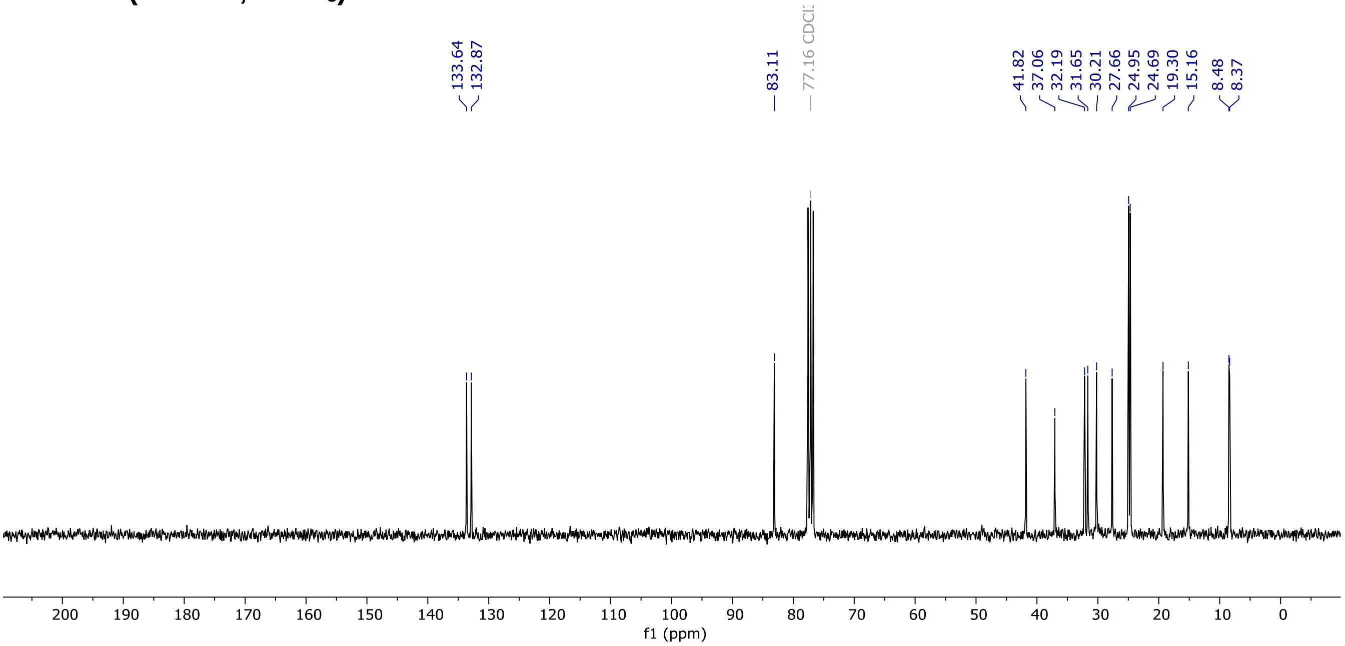

^{11}B NMR (129 MHz, $CDCl_3$)

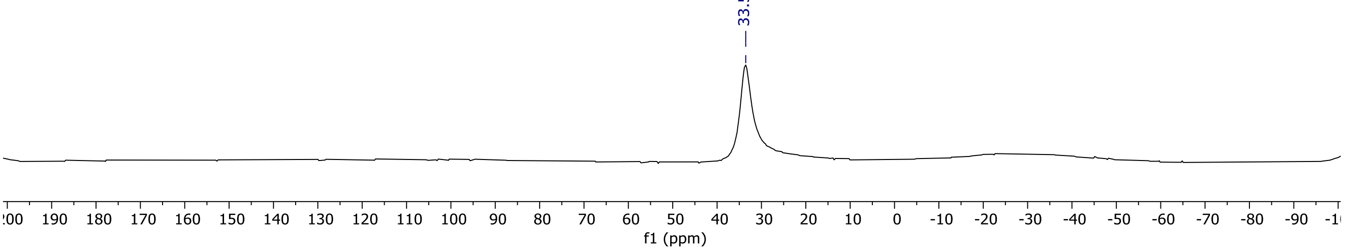

4,4,5,5-tetramethyl-2-(4'-propyl-3',4'-dihydro-2'H-[1,1':1',1''-terphenyl]-4'-yl)-1,3,2-dioxaborolane (4m)

^{1}H NMR (300 MHz, $CDCl_3$)

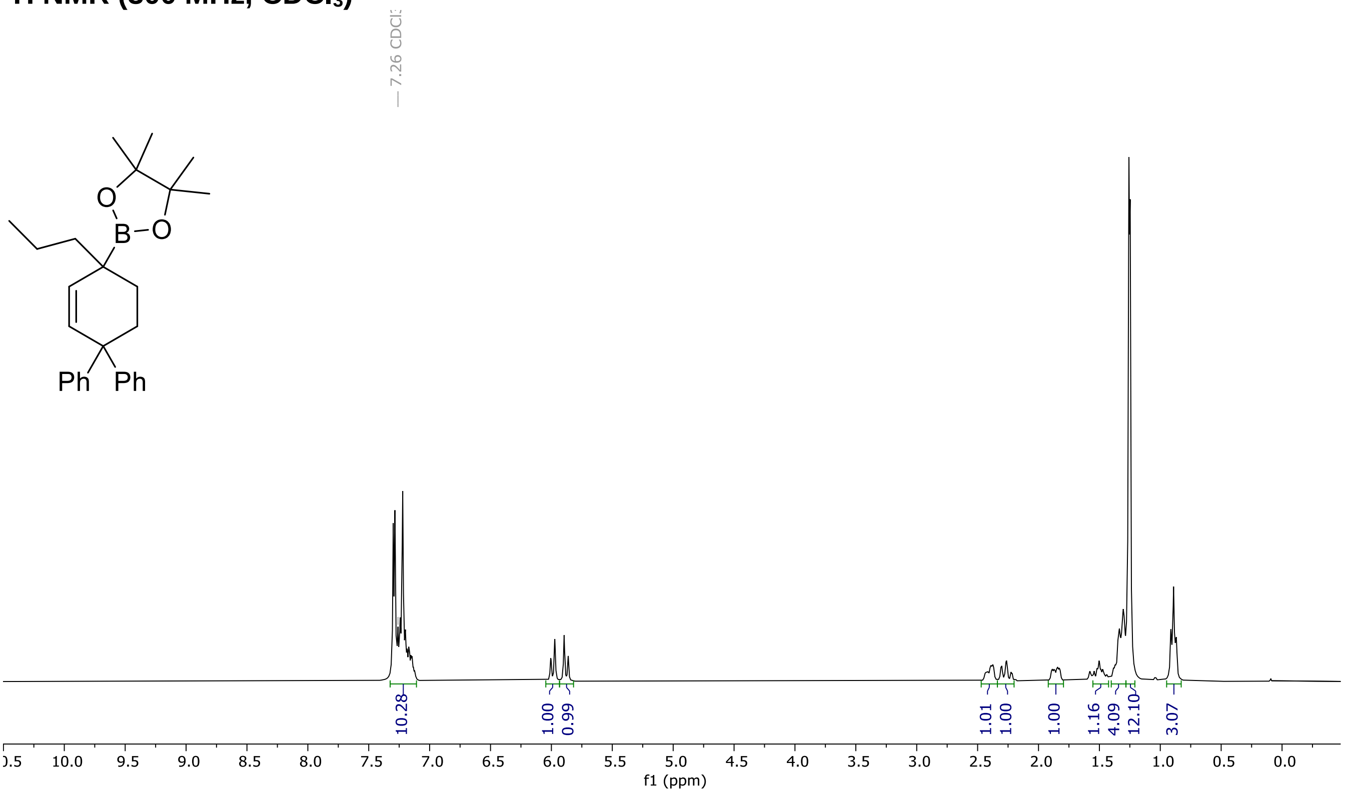

^{13}C NMR (75 MHz, $CDCl_3$)

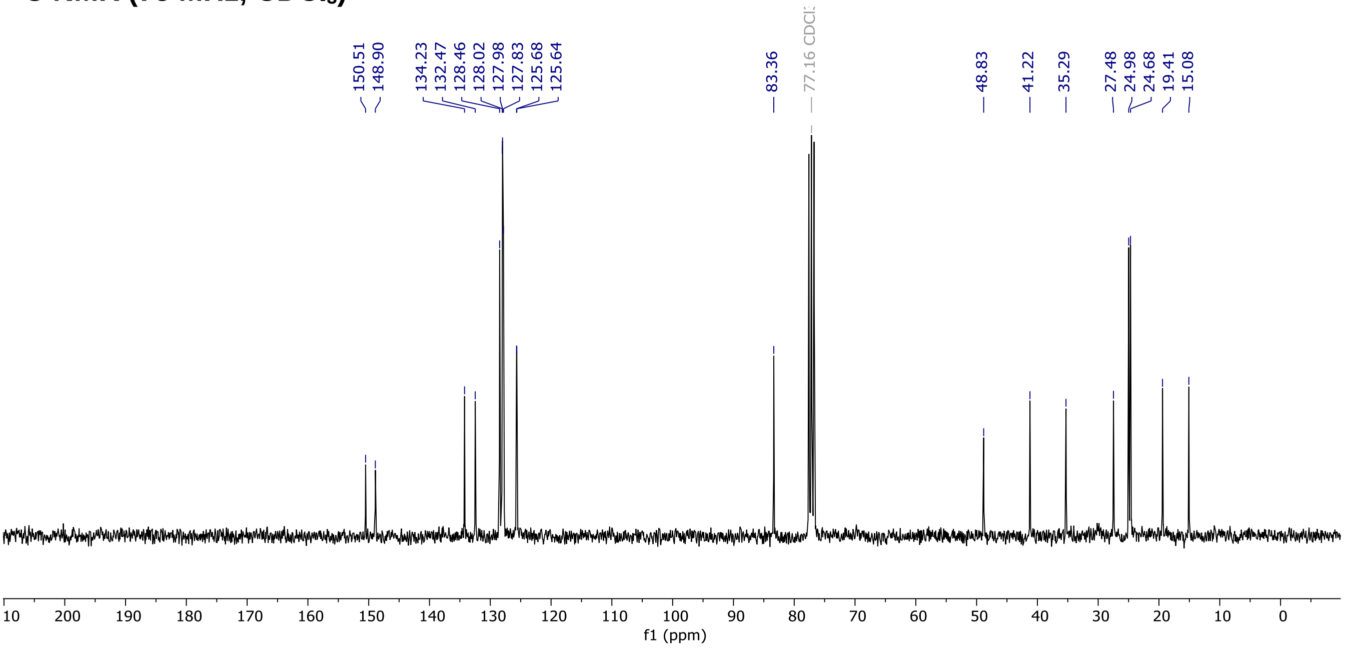

^{11}B NMR (129 MHz, $CDCl_3$)

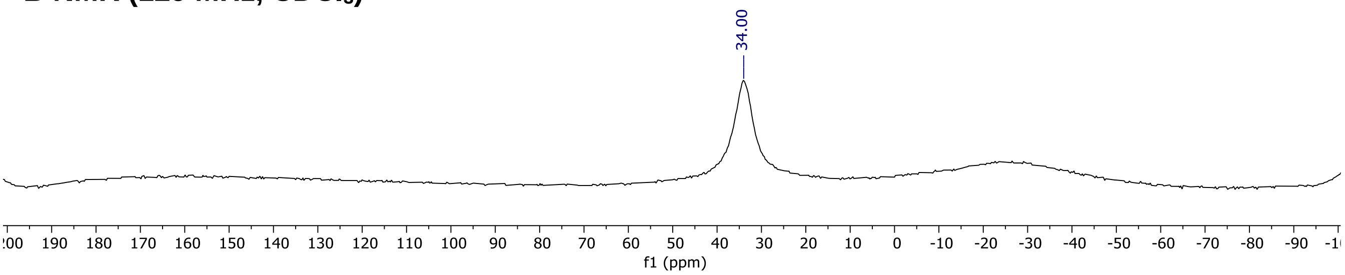

4,4,5,5-tetramethyl-2-(3-methyl-1-propylcyclohex-2-en-1-yl)-1,3,2-dioxaborolane (4n)

^{1}H NMR (300 MHz, $CDCl_3$)

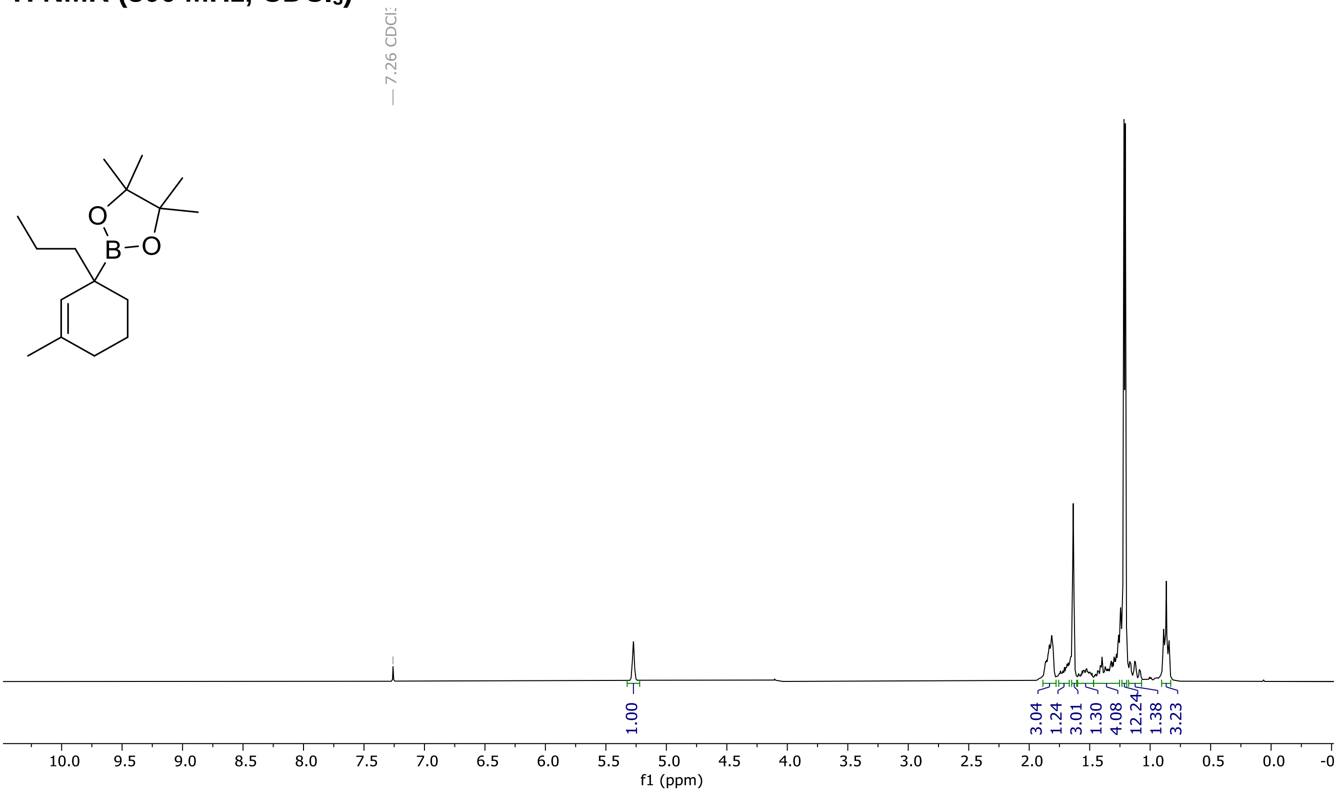

^{13}C NMR (75 MHz, $CDCl_3$)

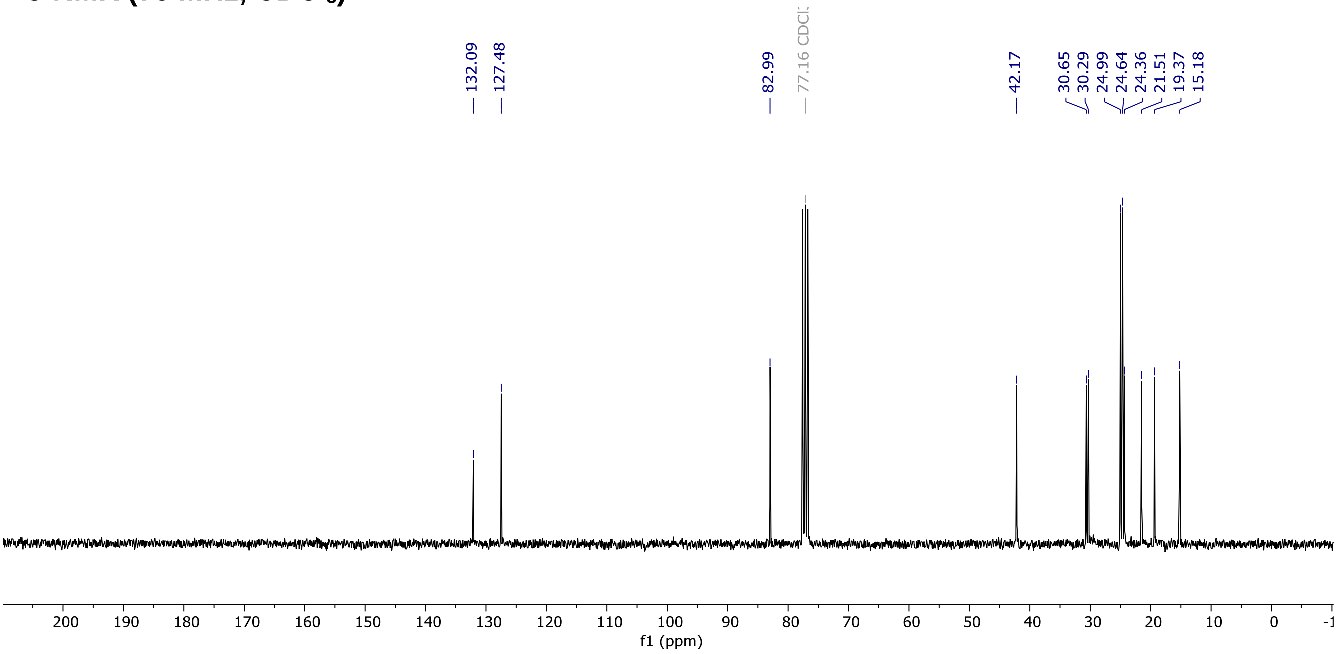

^{11}B NMR (129 MHz, $CDCl_3$)

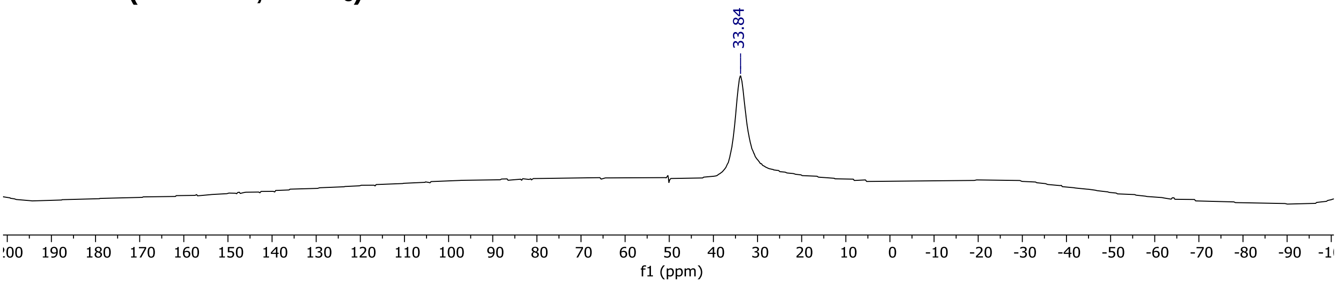

4,4,5,5-tetramethyl-2-(3-methyl-1-propylcyclopent-2-en-1-yl)-1,3,2-dioxaborolane (4o)

^{1}H NMR (300 MHz, $CDCl_3$)

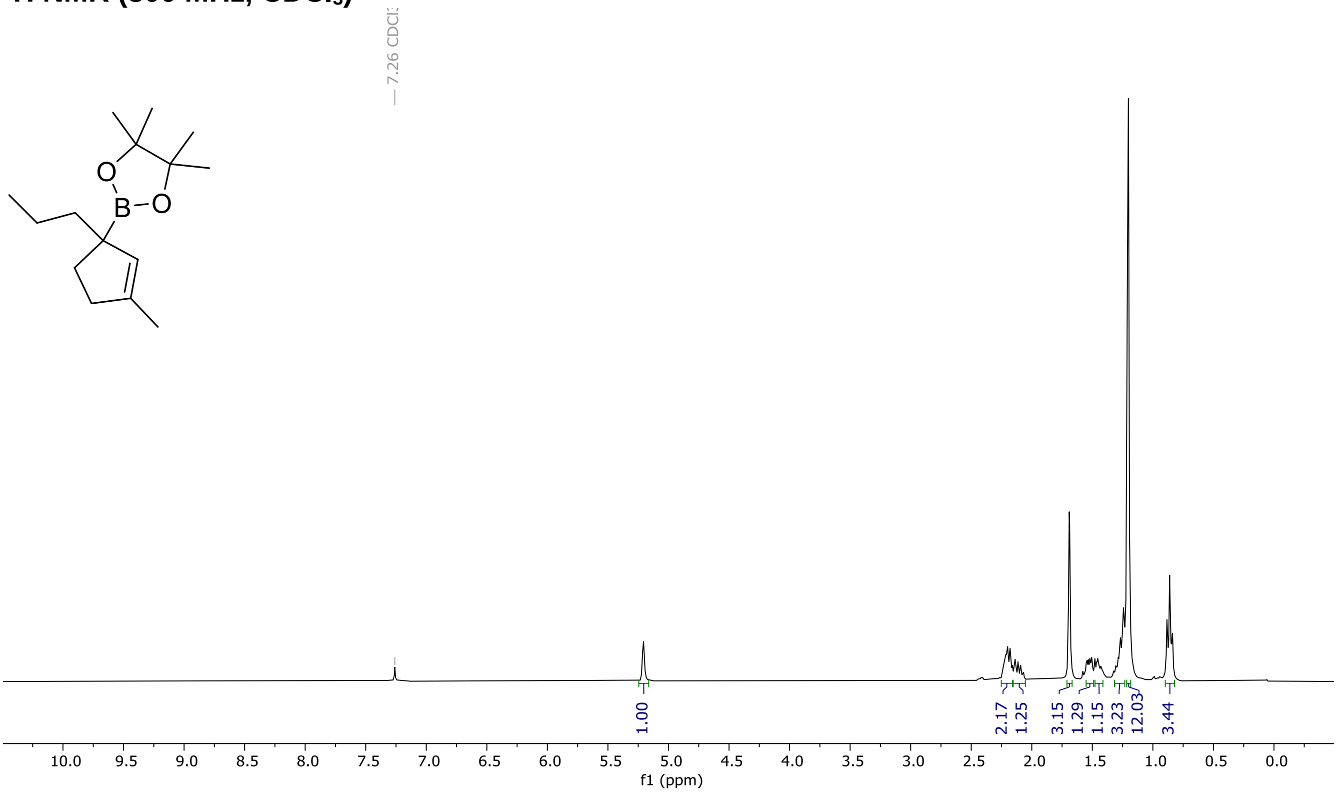

^{13}C NMR (75 MHz, $CDCl_3$)

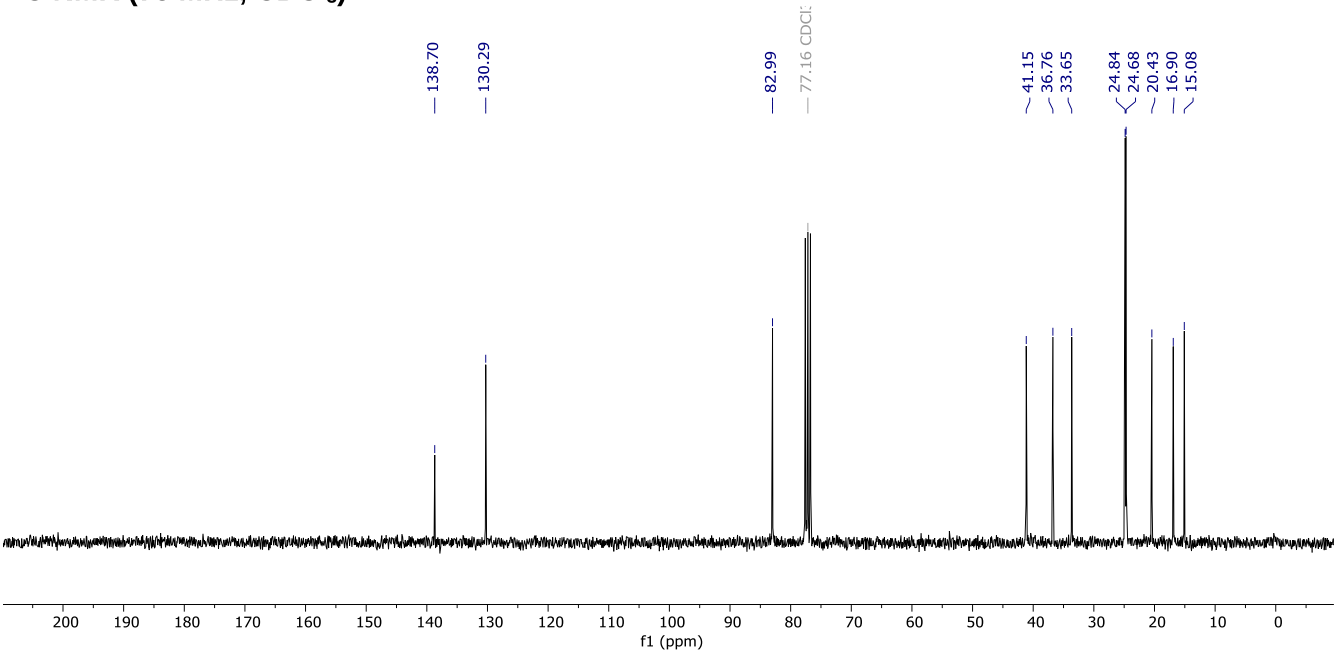

^{11}B NMR (129 MHz, $CDCl_3$)

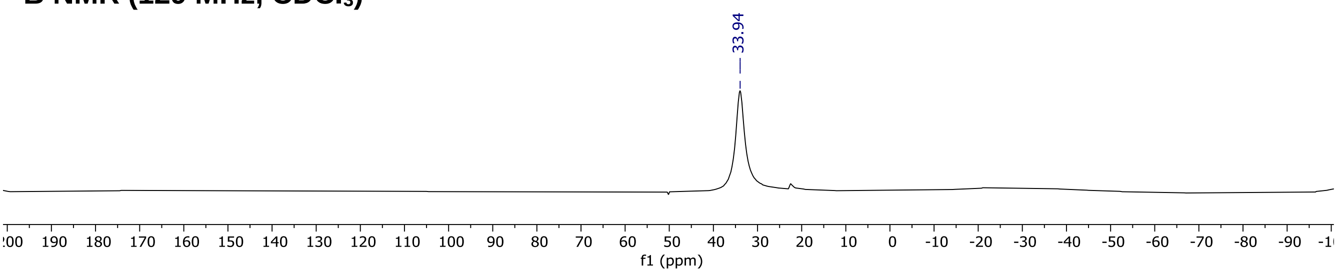

4,4,5,5-tetramethyl-2-(3-methyl-1-phenethylcyclopent-2-en-1-yl)-1,3,2-dioxaborolane (4p)

^{1}H NMR (300 MHz, $CDCl_3$)

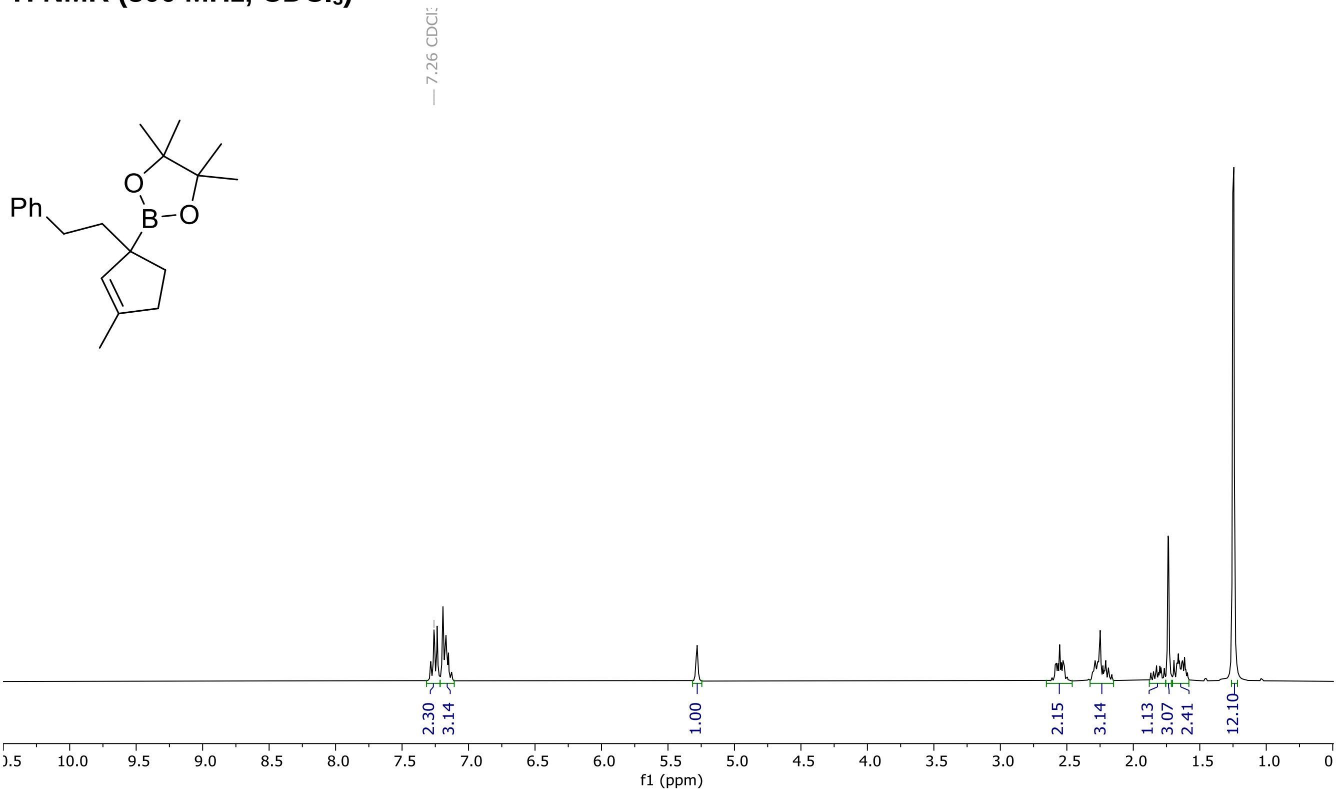

^{13}C NMR (75 MHz, $CDCl_3$)

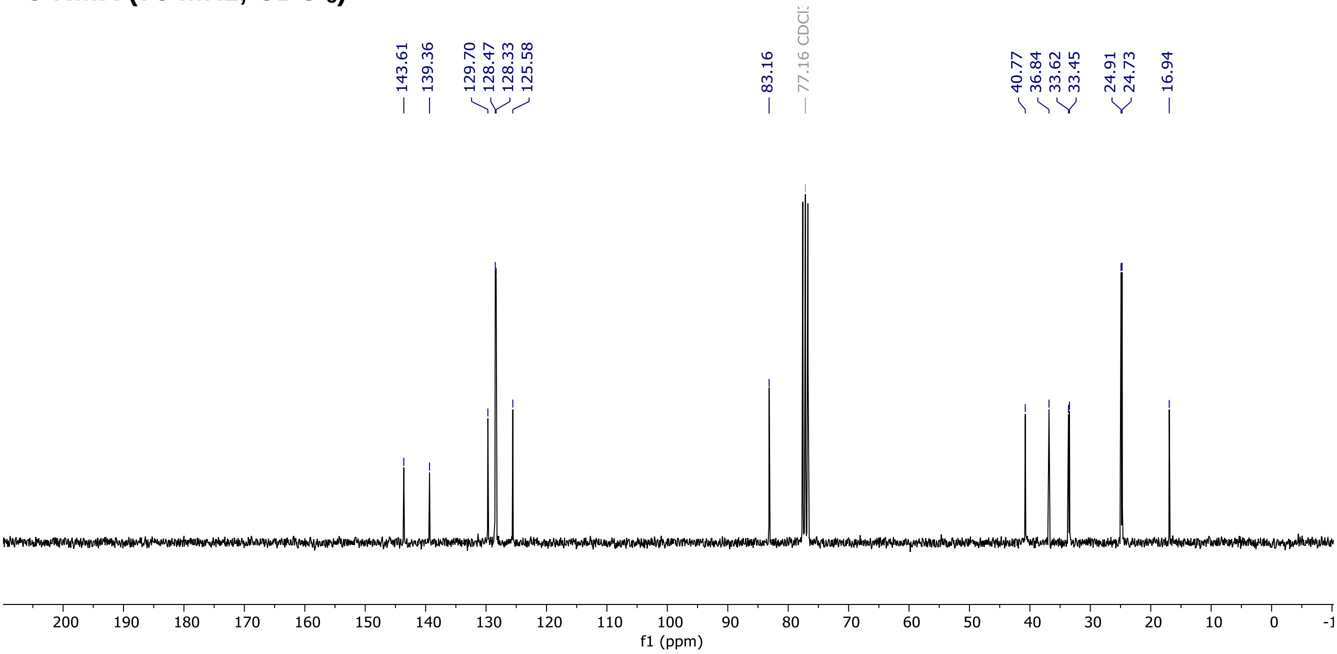

^{11}B NMR (129 MHz, $CDCl_3$)

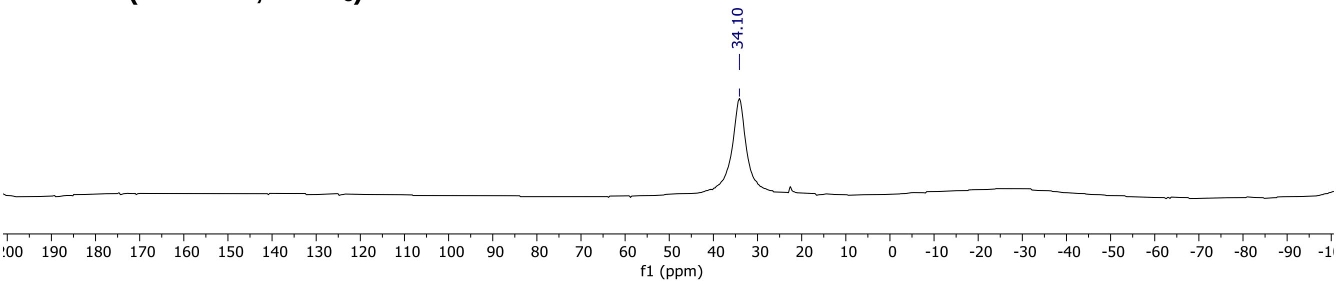

2-(1-cyclopropyl-3-methylcyclopent-2-en-1-yl)-4,4,5,5-tetramethyl-1,3,2-dioxaborolane (4q)

^{1}H NMR (300 MHz, $CDCl_3$)

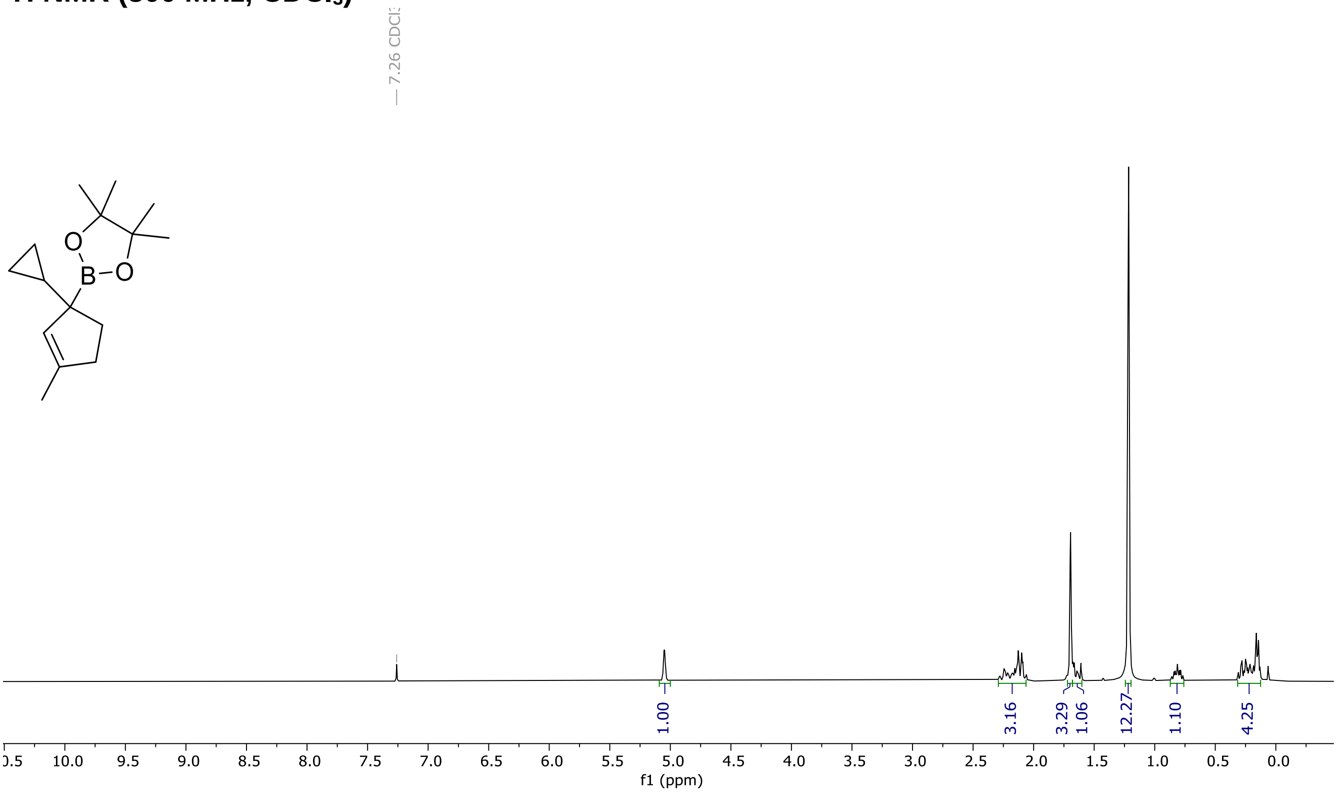

^{13}C NMR (75 MHz, $CDCl_3$)

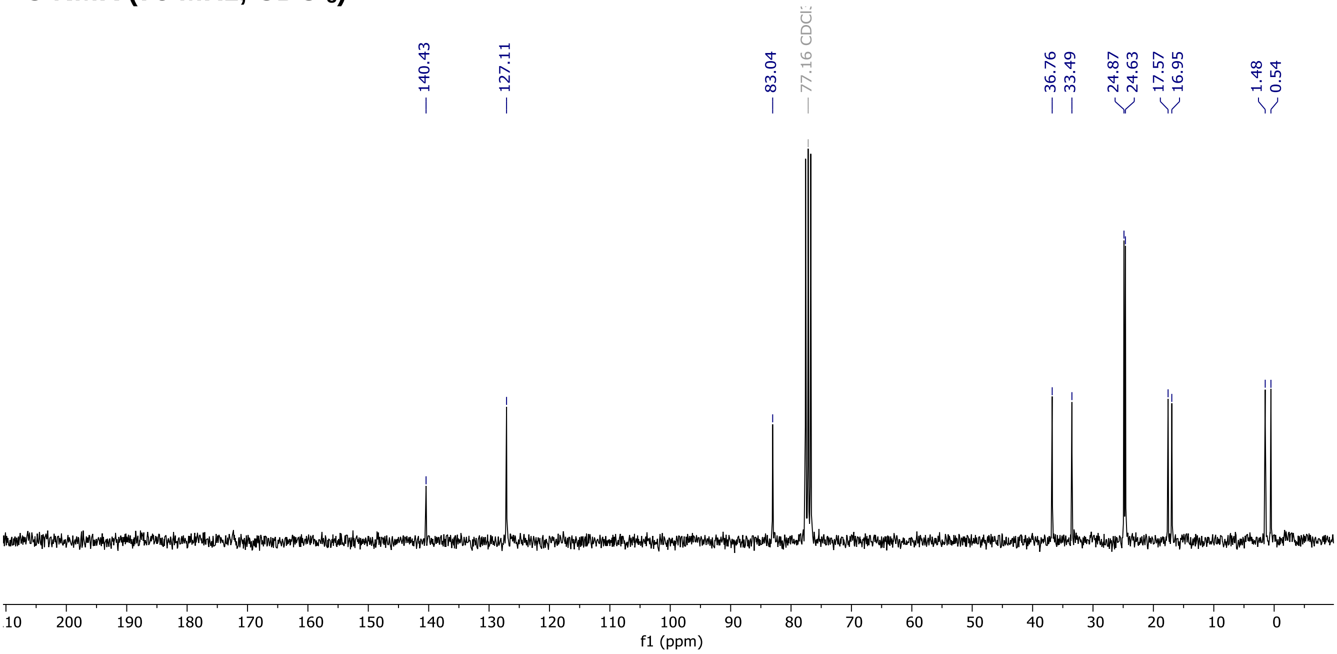

^{11}B NMR (129 MHz, $CDCl_3$)

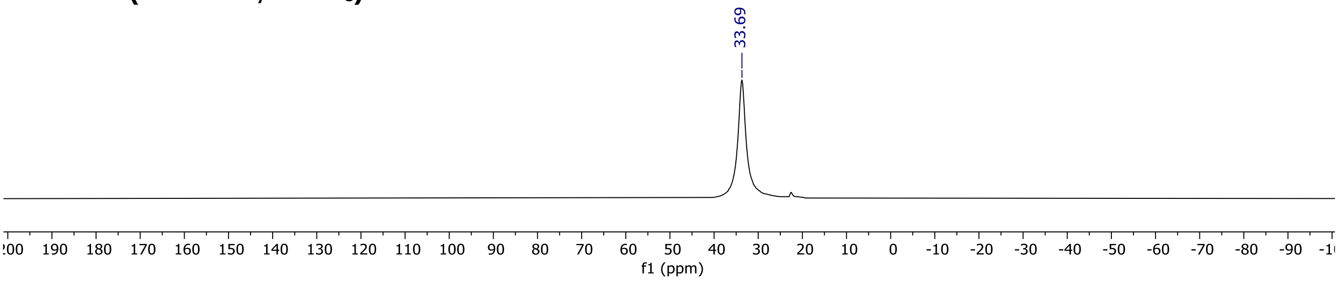

2-(1-(but-3-en-1-yl)-3-methylcyclopent-2-en-1-yl)-4,4,5,5-tetramethyl-1,3,2-dioxaborolane (4r)

^{1}H NMR (300 MHz, CDCl$_3$)

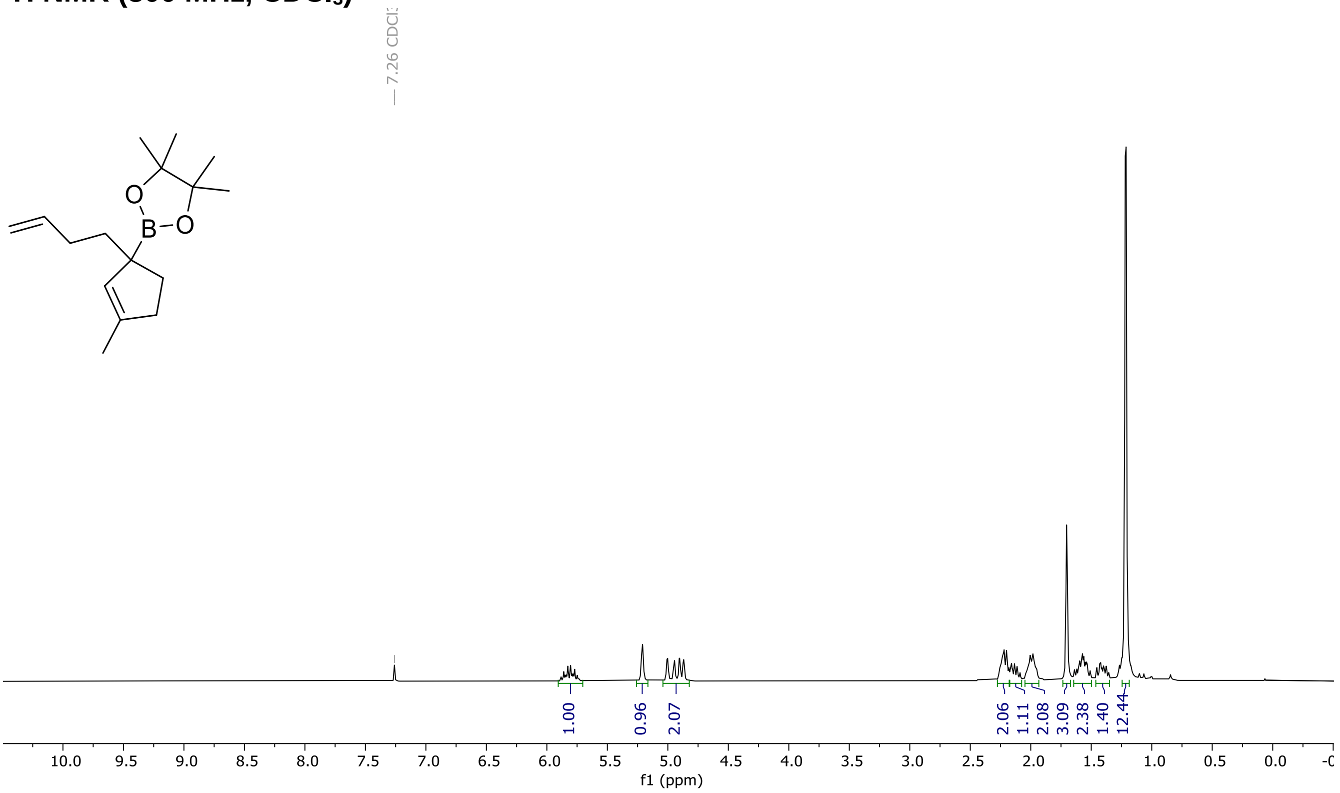

^{13}C NMR (75 MHz, CDCl$_3$)

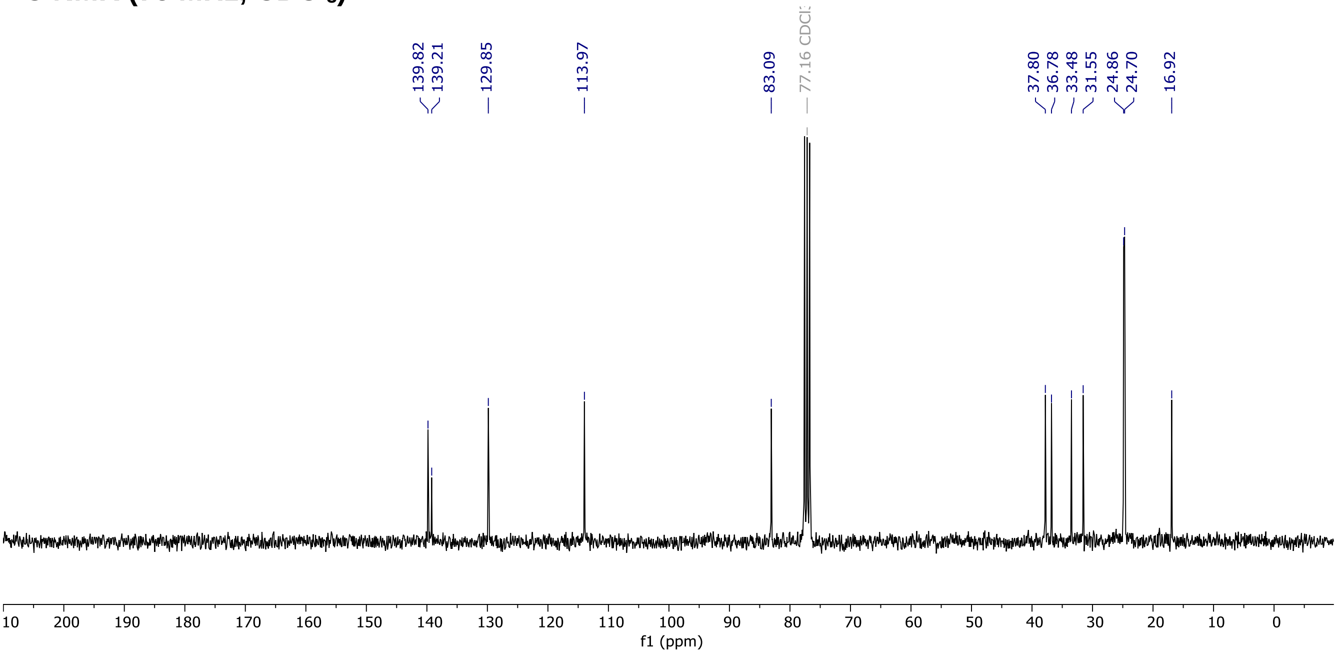

^{11}B NMR (129 MHz, CDCl$_3$)

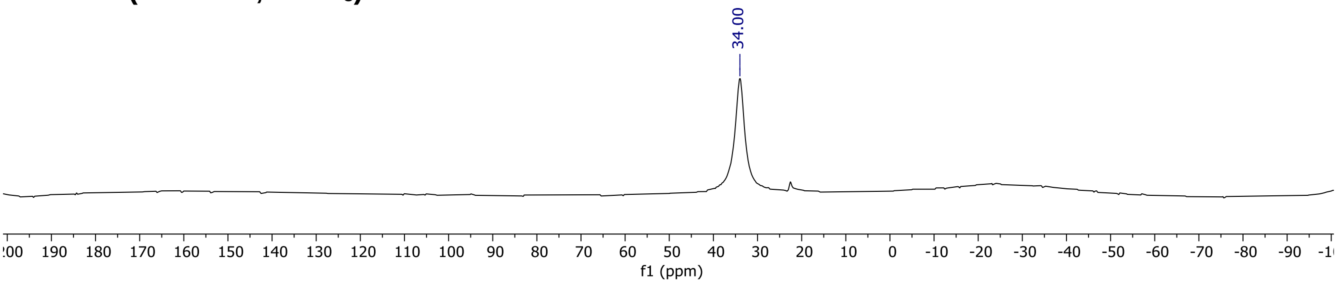

6-(3-methyl-1-(4,4,5,5-tetramethyl-1,3,2-dioxaborolan-2-yl)cyclopent-2-en-1-yl)hexan-2-one (4s)

^{1}H NMR (300 MHz, $CDCl_3$)

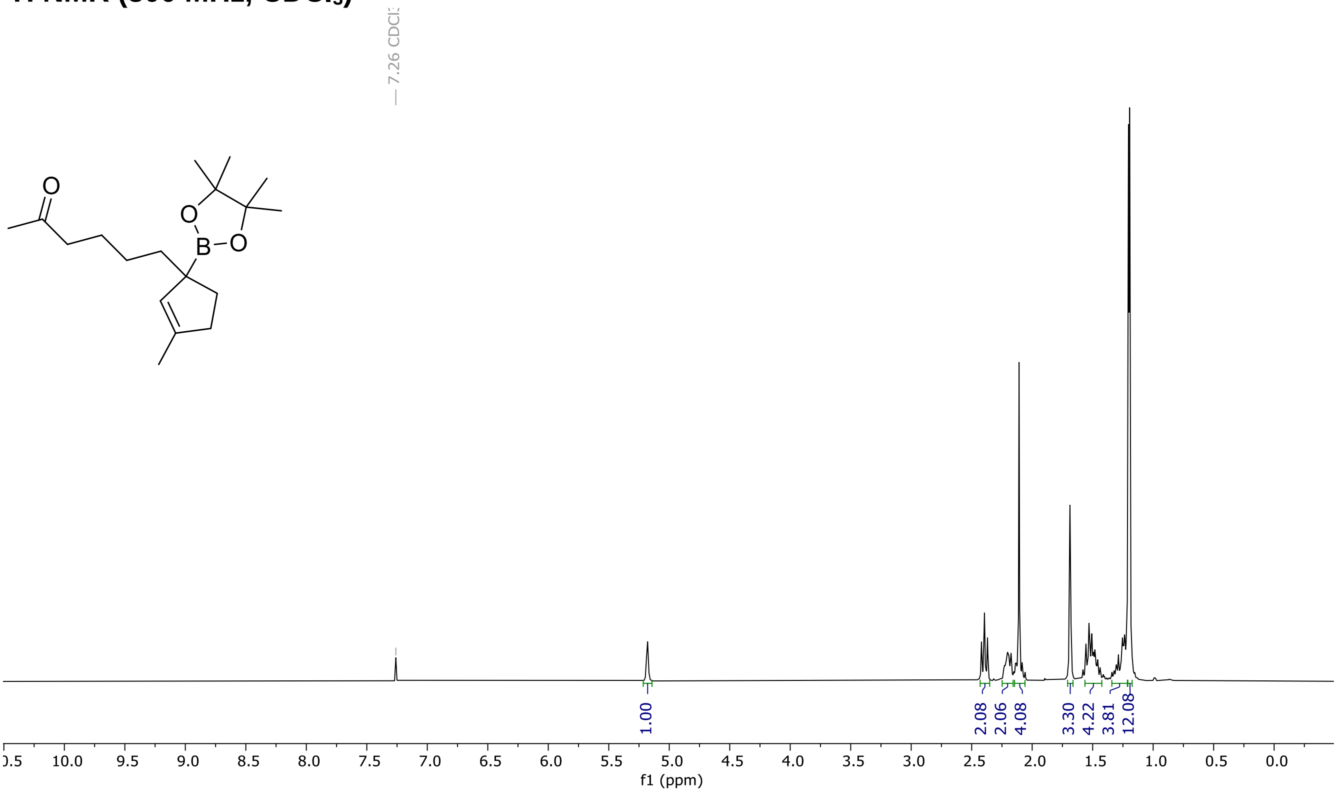

^{13}C NMR (75 MHz, $CDCl_3$)

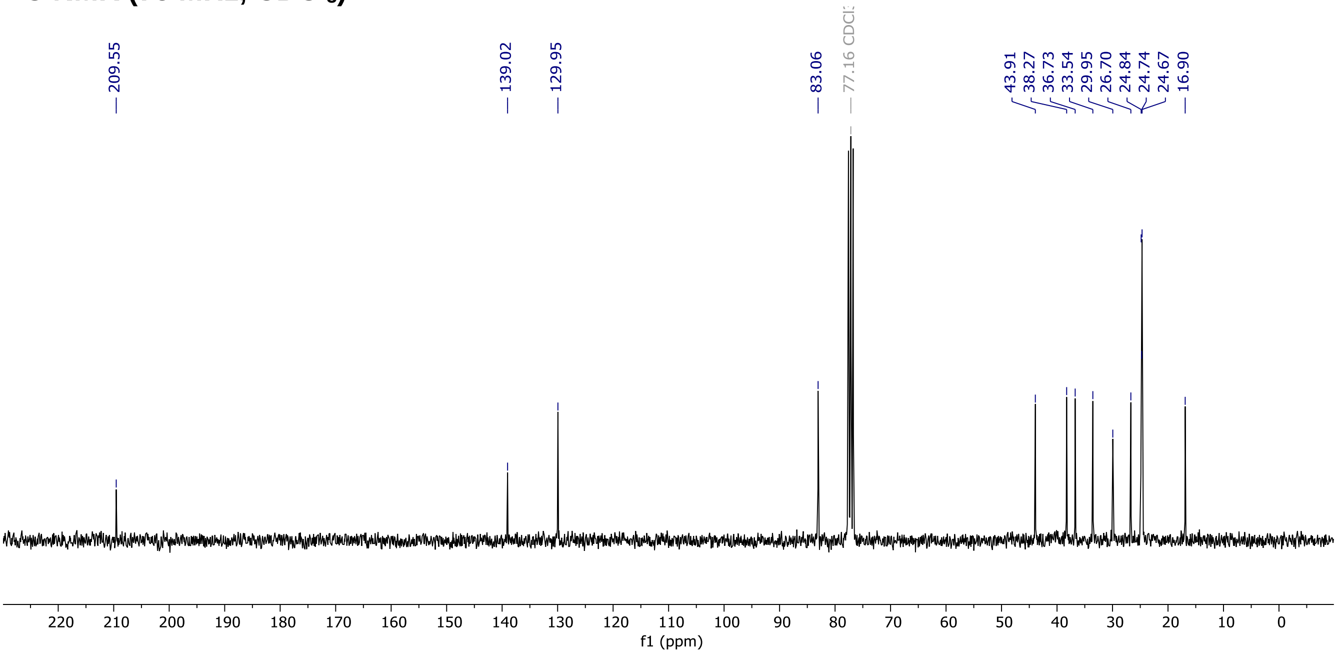

^{11}B NMR (129 MHz, $CDCl_3$)

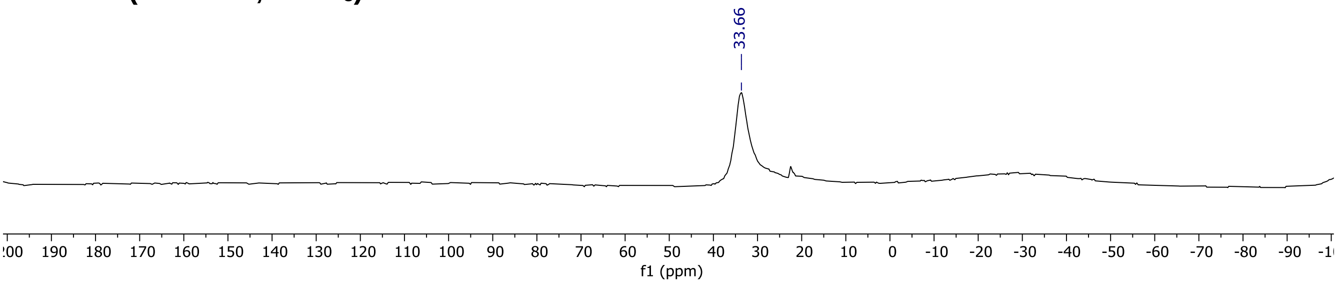

benzyl 4-propyl-4-(4,4,5,5-tetramethyl-1,3,2-dioxaborolan-2-yl)-3,4-dihydropyridine-1(2H)-carboxylate (4t)

^{1}H NMR (300 MHz, $CDCl_3$)

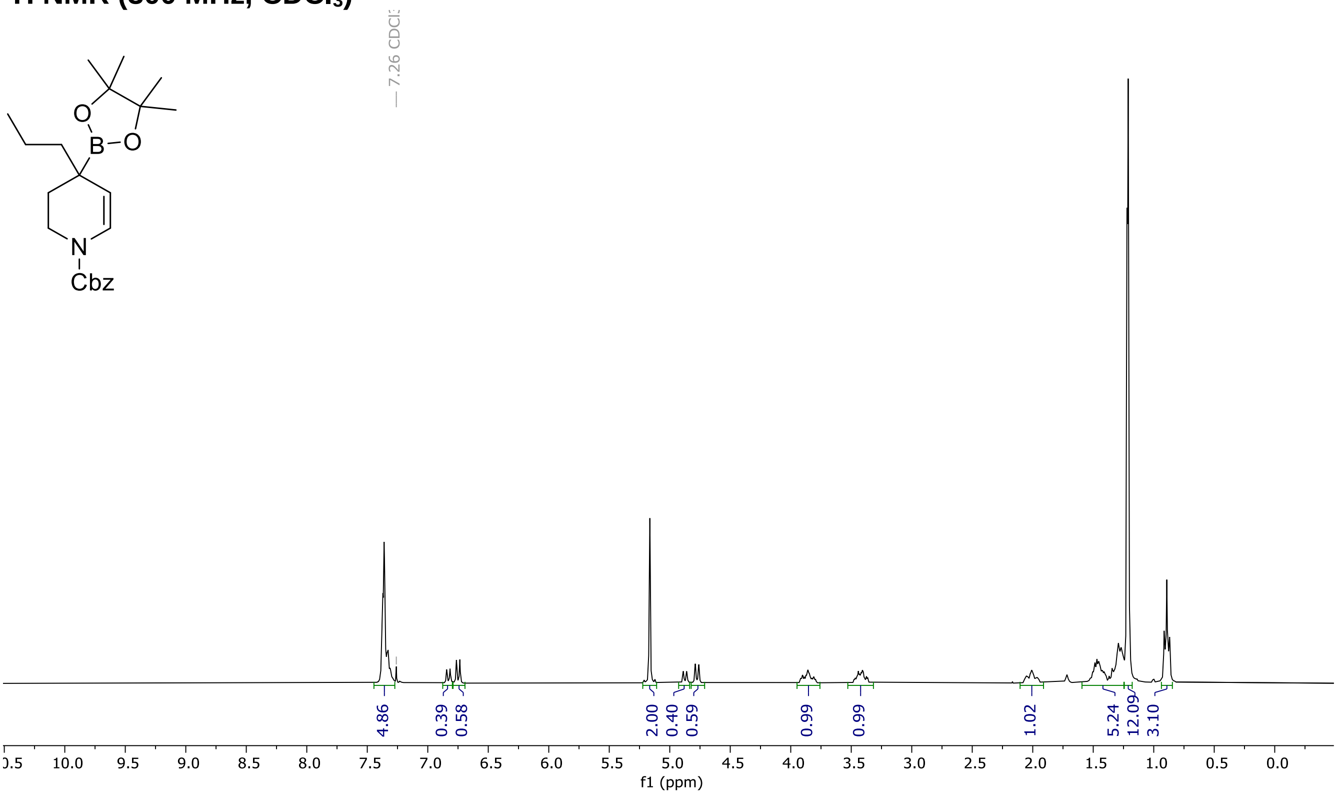

^{13}C NMR (75 MHz, $CDCl_3$)

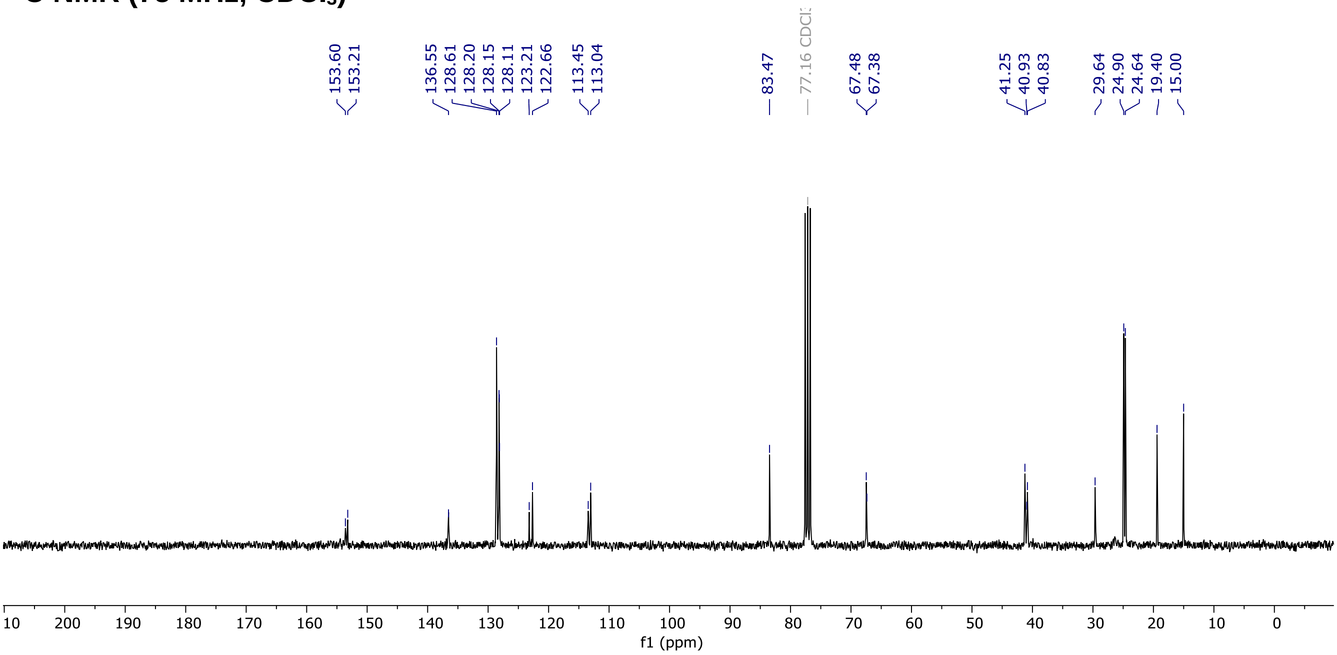

^{11}B NMR (129 MHz, $CDCl_3$)

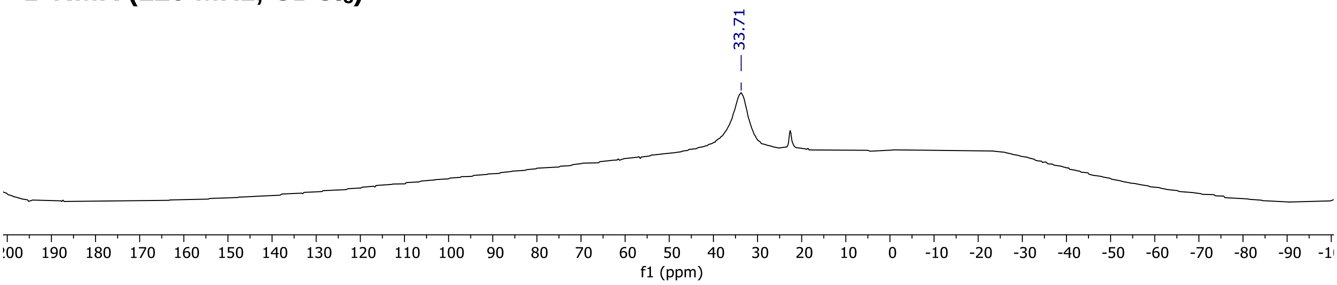

benzyl 4-phenethyl-4-(4,4,5,5-tetramethyl-1,3,2-dioxaborolan-2-yl)-3,4-dihydropyridine-1(2H)-carboxylate (4u)

^{1}H NMR (300 MHz, CDCl$_3$)

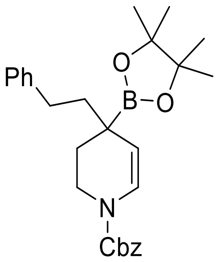

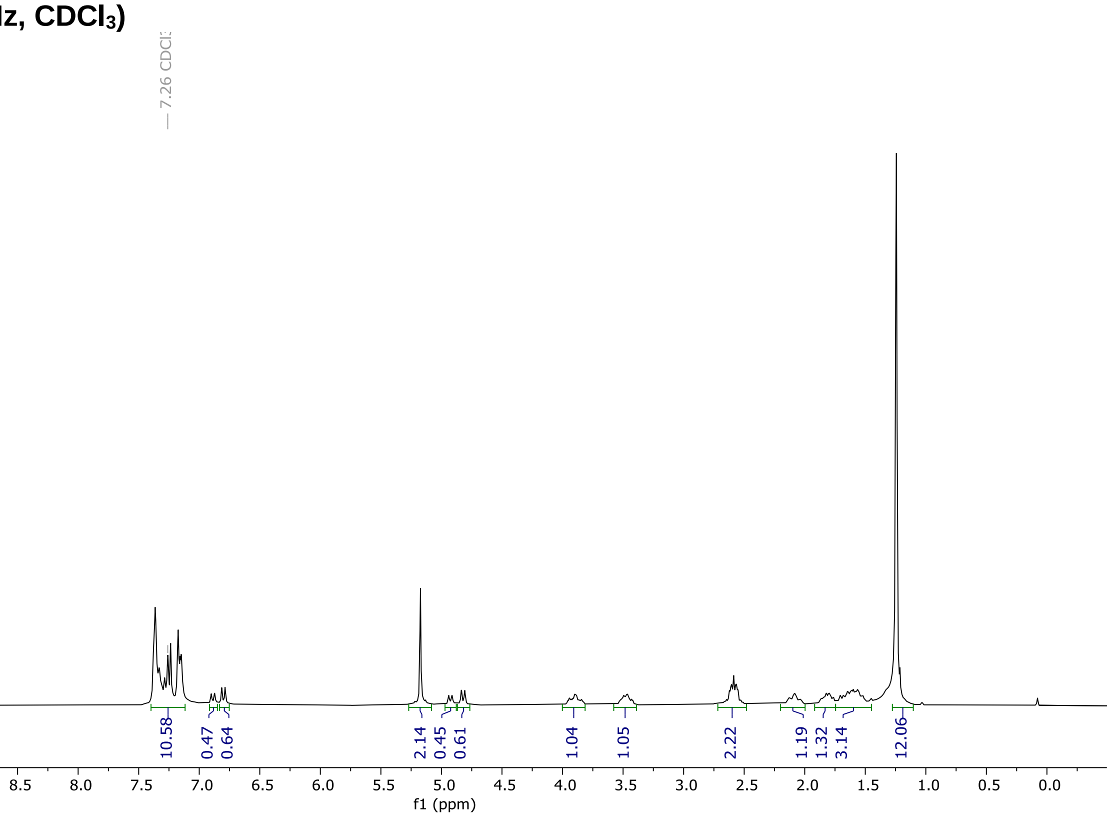

^{13}C NMR (75 MHz, CDCl$_3$)

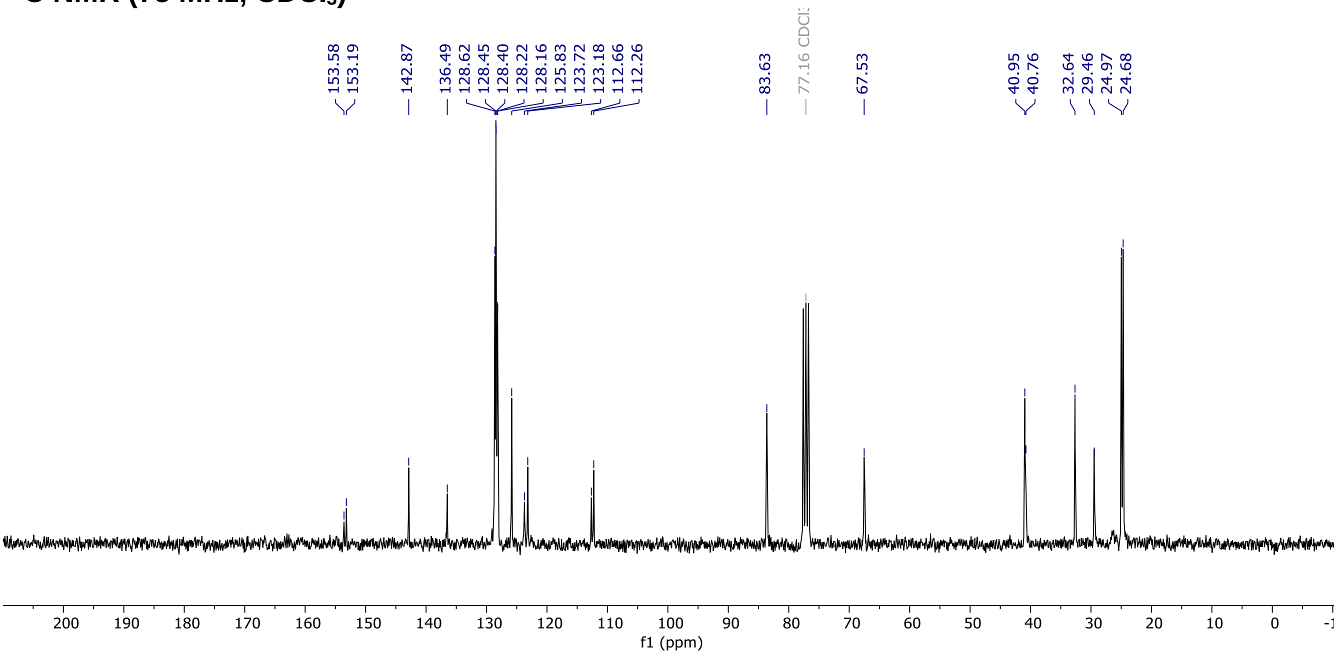

^{11}B NMR (129 MHz, CDCl$_3$)

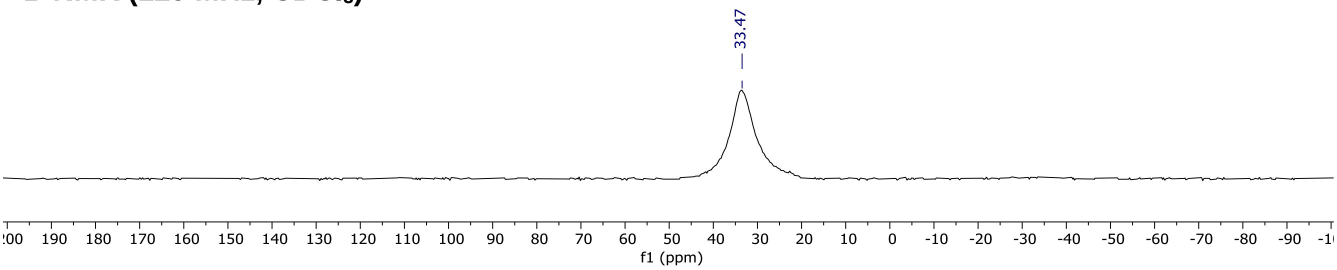

benzyl 4-(3-bromopropyl)-4-(4,4,5,5-tetramethyl-1,3,2-dioxaborolan-2-yl)-3,4-dihydropyridine-1(2H)-carboxylate (4v)

^{1}H NMR (300 MHz, $CDCl_3$)

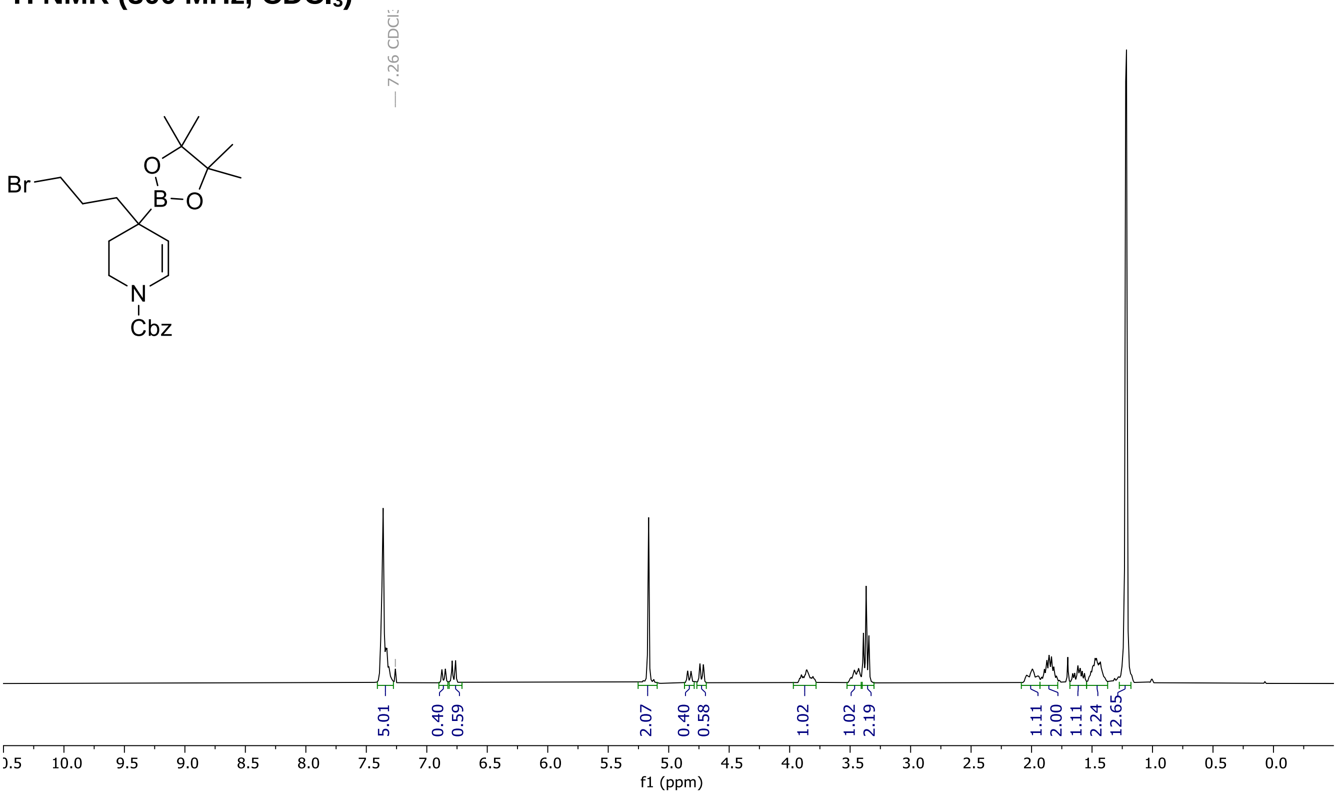

^{13}C NMR (75 MHz, $CDCl_3$)

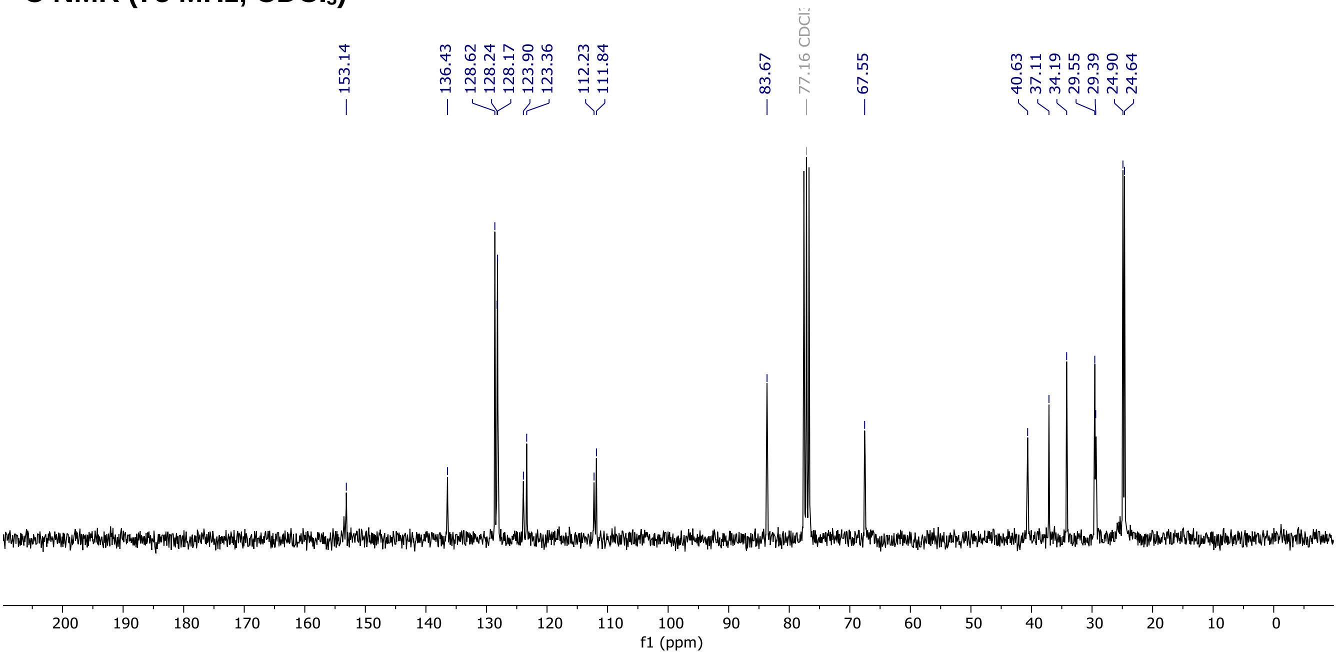

^{11}B NMR (129 MHz, $CDCl_3$)

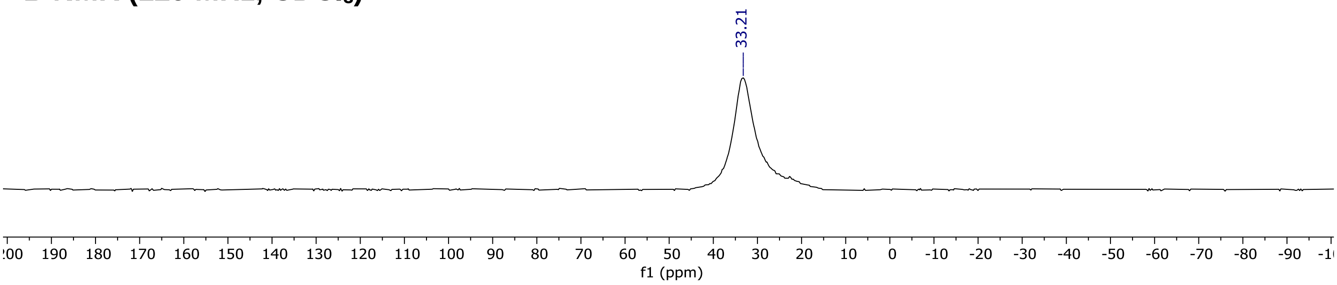

benzyl 4-cyclopropyl-4-(4,4,5,5-tetramethyl-1,3,2-dioxaborolan-2-yl)-3,4-dihydropyridine-1(2H)-carboxylate (4w)

^{1}H NMR (300 MHz, $CDCl_3$)

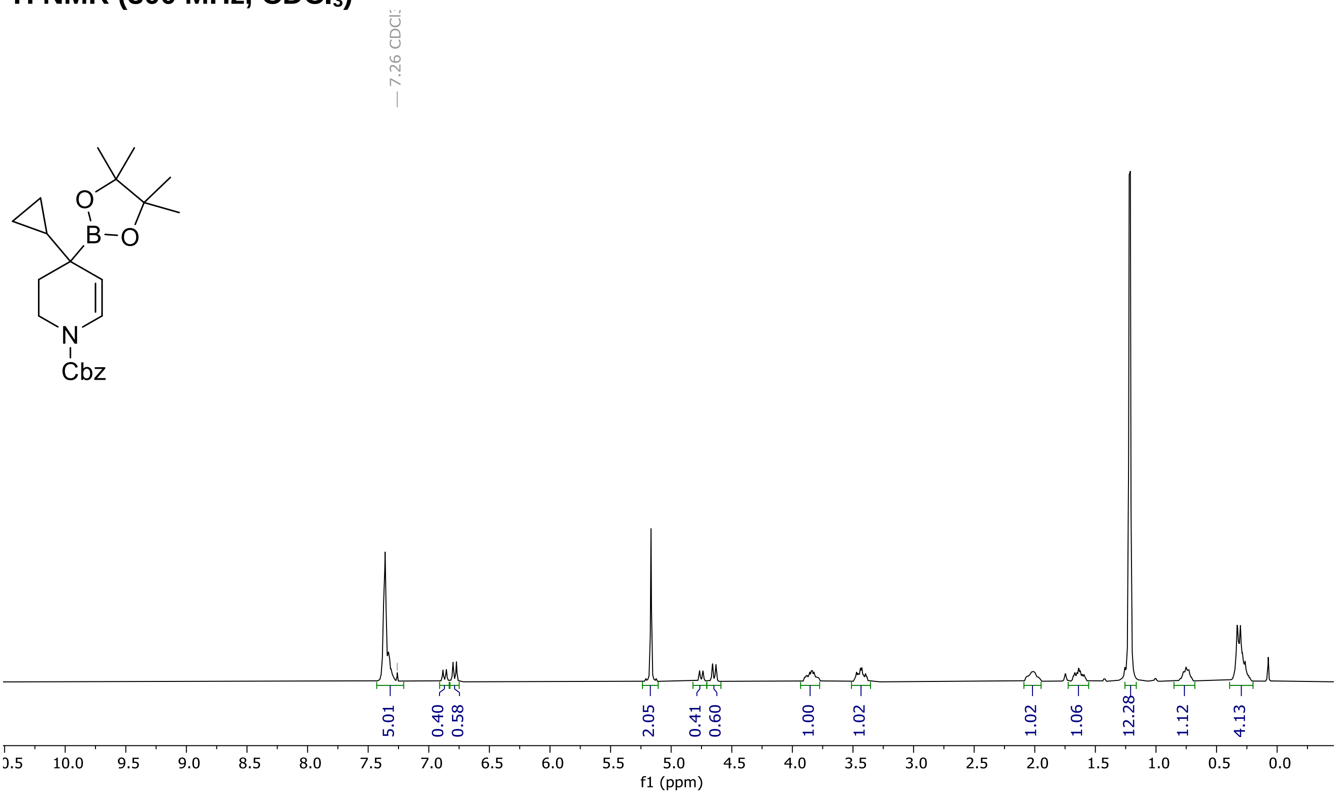

^{13}C NMR (75 MHz, $CDCl_3$)

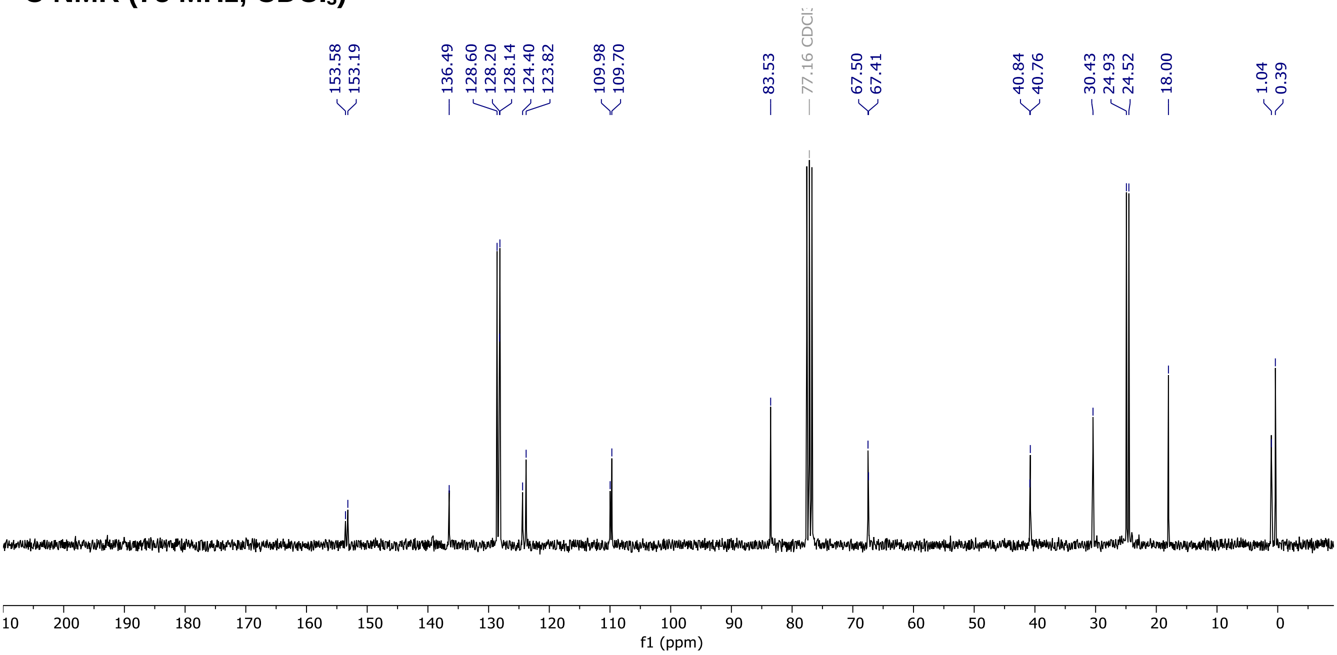

^{11}B NMR (129 MHz, $CDCl_3$)

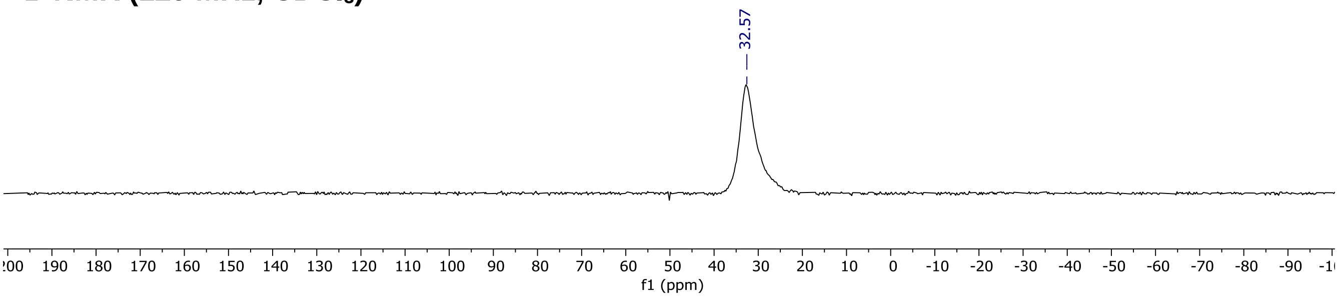

benzyl 4-(but-3-en-1-yl)-4-(4,4,5,5-tetramethyl-1,3,2-dioxaborolan-2-yl)-3,4-dihydropyridine-1(2H)-carboxylate (4x)

^{1}H NMR (300 MHz, $CDCl_3$)

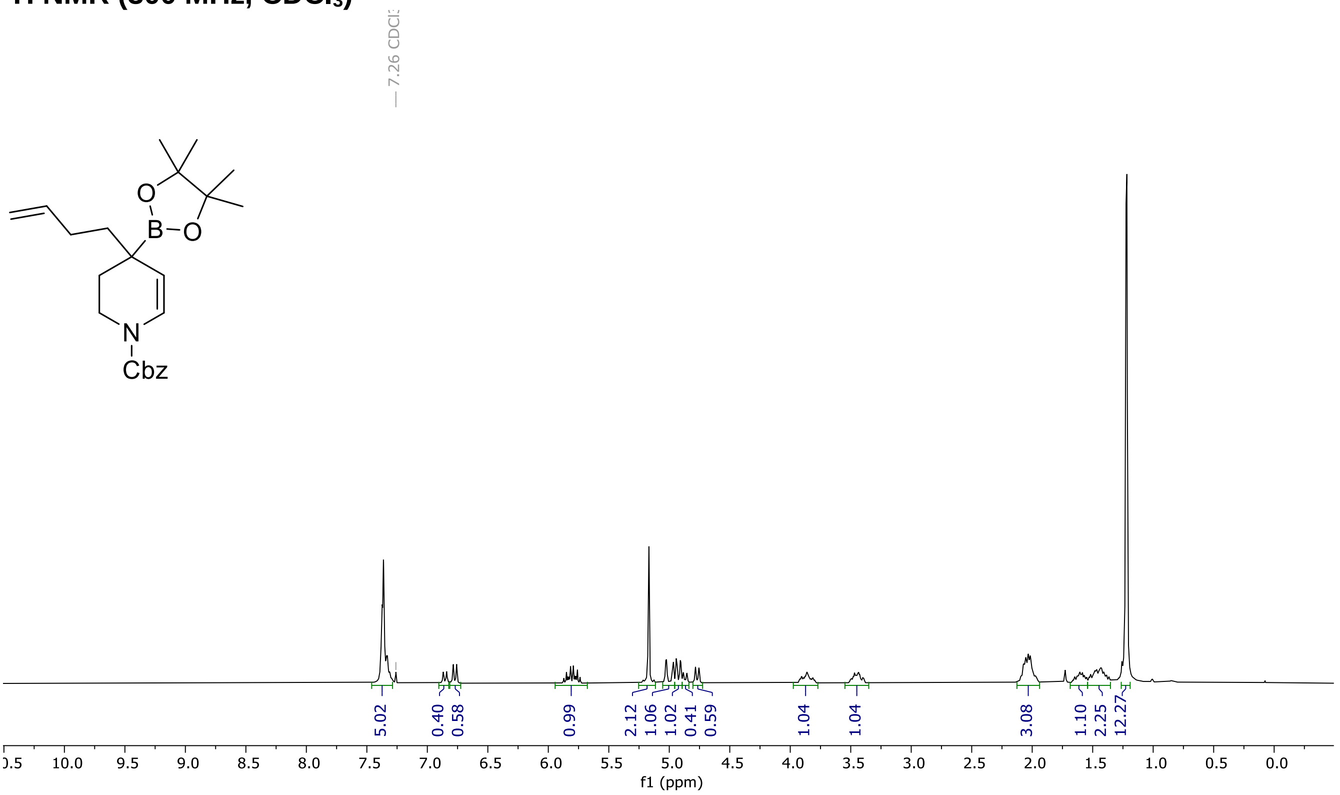

^{13}C NMR (75 MHz, $CDCl_3$)

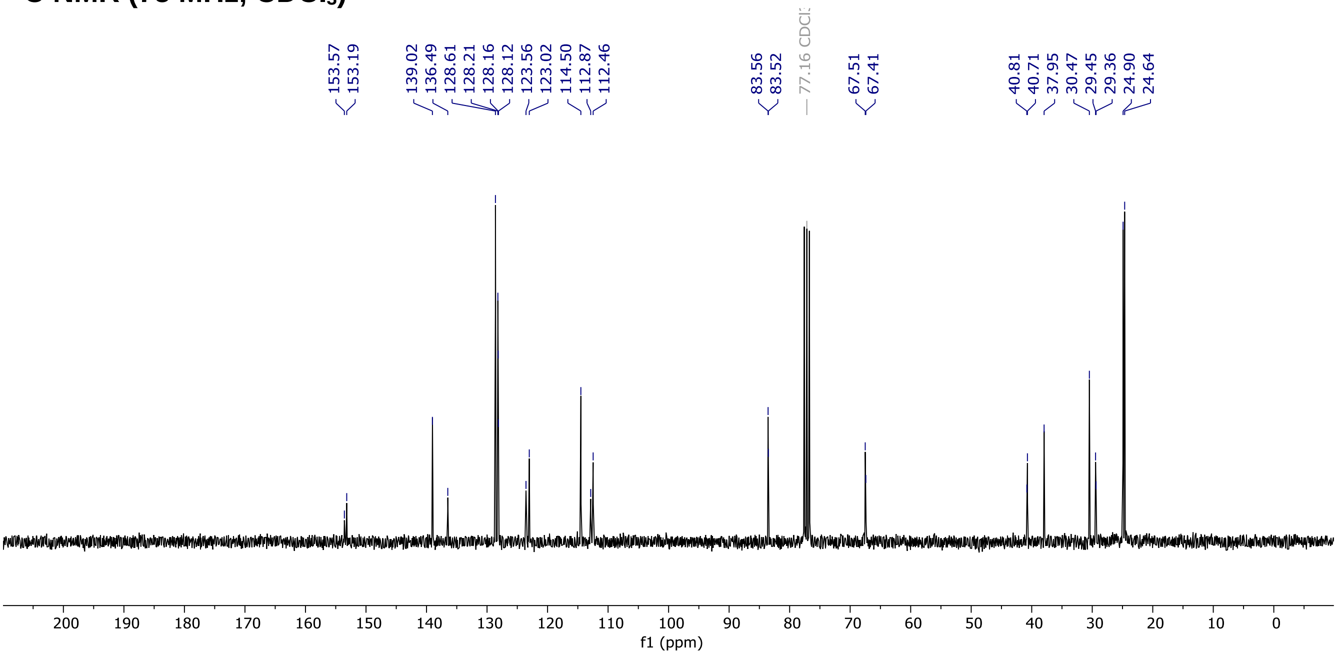

^{11}B NMR (129 MHz, $CDCl_3$)

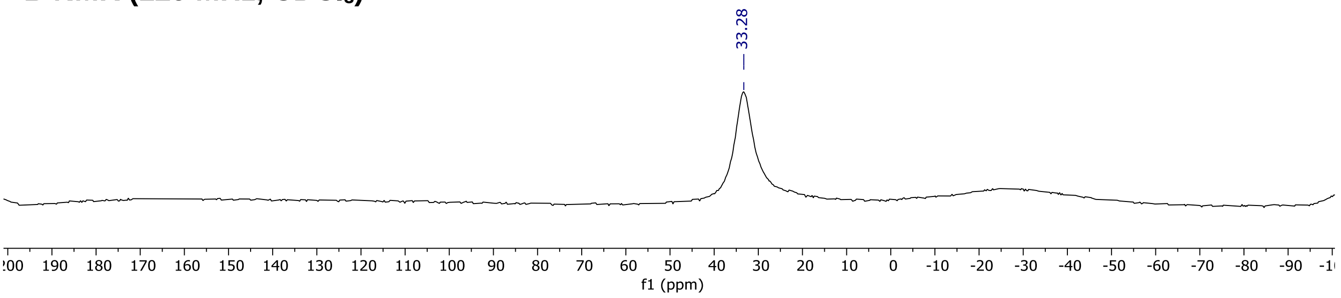

benzyl 4-(5-oxohexyl)-4-(4,4,5,5-tetramethyl-1,3,2-dioxaborolan-2-yl)-3,4-dihydropyridine-1(2H)-carboxylate (4y)

^{1}H NMR (300 MHz, $CDCl_3$)

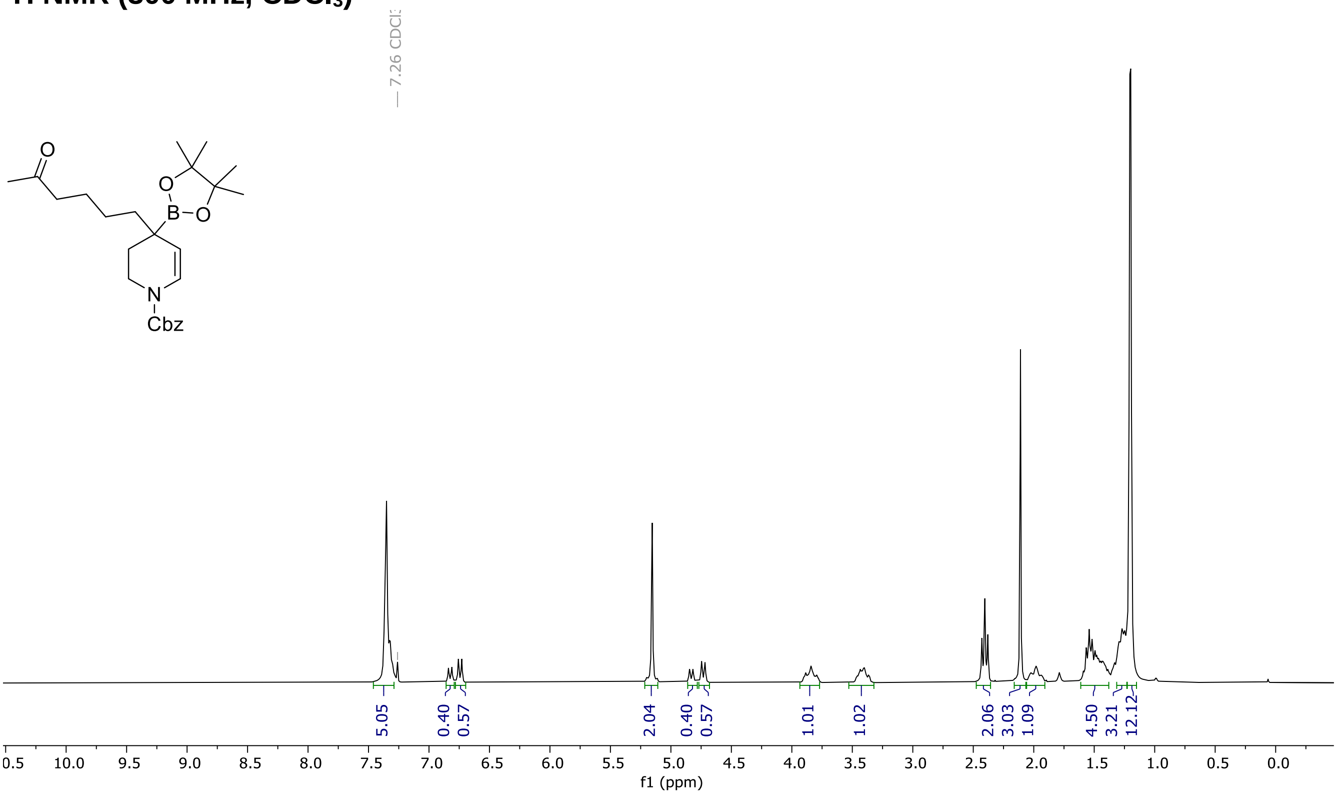

^{13}C NMR (75 MHz, $CDCl_3$)

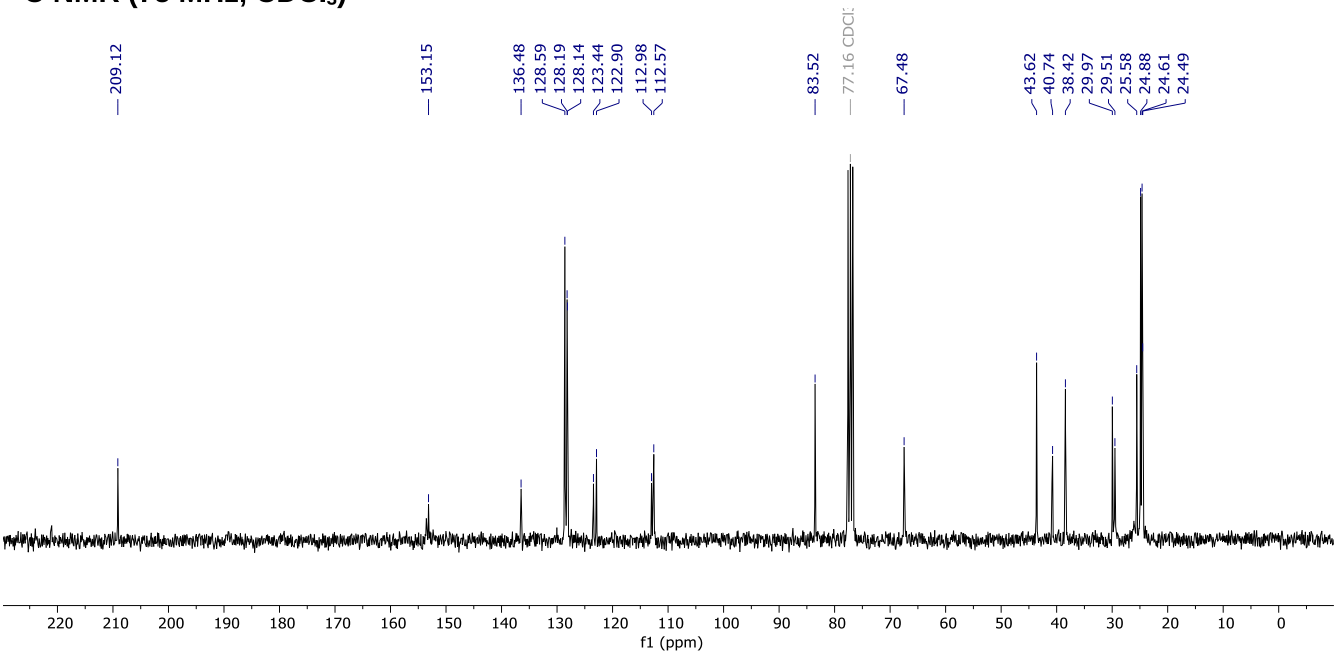

^{11}B NMR (129 MHz, $CDCl_3$)

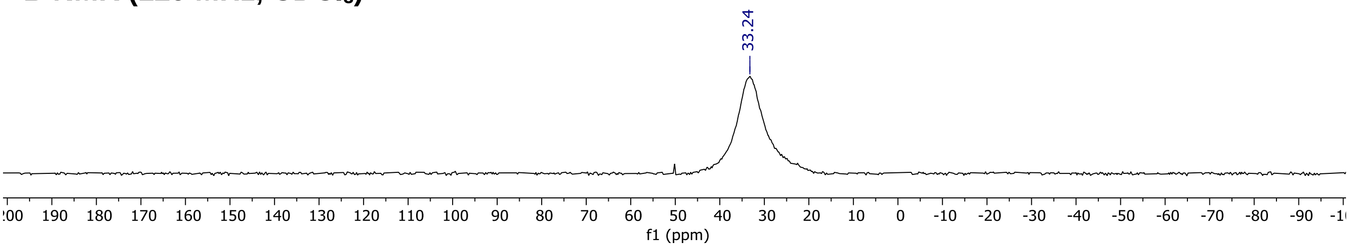

(8R,9S,10R,13S,14S,17S)-10,13,17-trimethyl-3-propyl-3-(4,4,5,5-tetramethyl-1,3,2-dioxaborolan-2-yl)-2,3,6,7,8,9,10,11,12,13,14,15,16,17-tetradecahydro-1H-cyclopenta[a]phenanthren-17-ol (4z)

^{1}H NMR (300 MHz, CDCl$_3$)

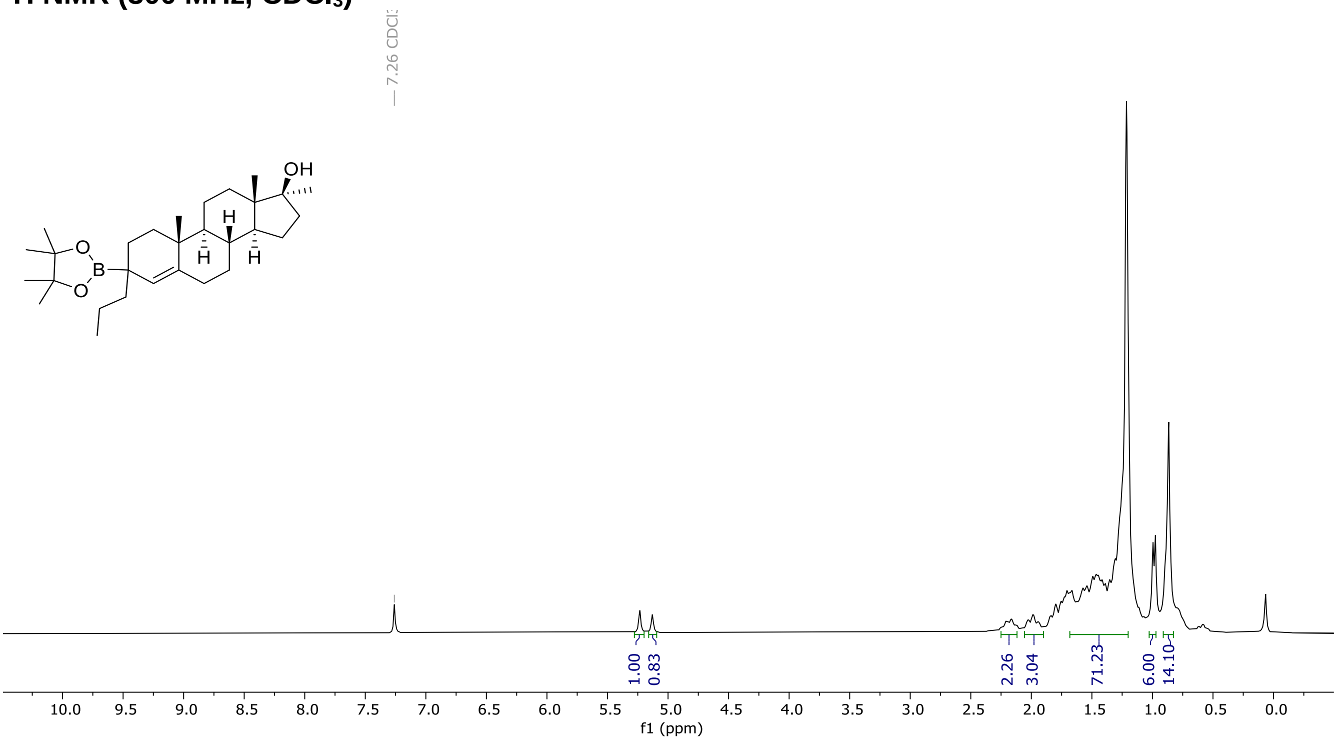

^{13}C NMR (75 MHz, CDCl$_3$)

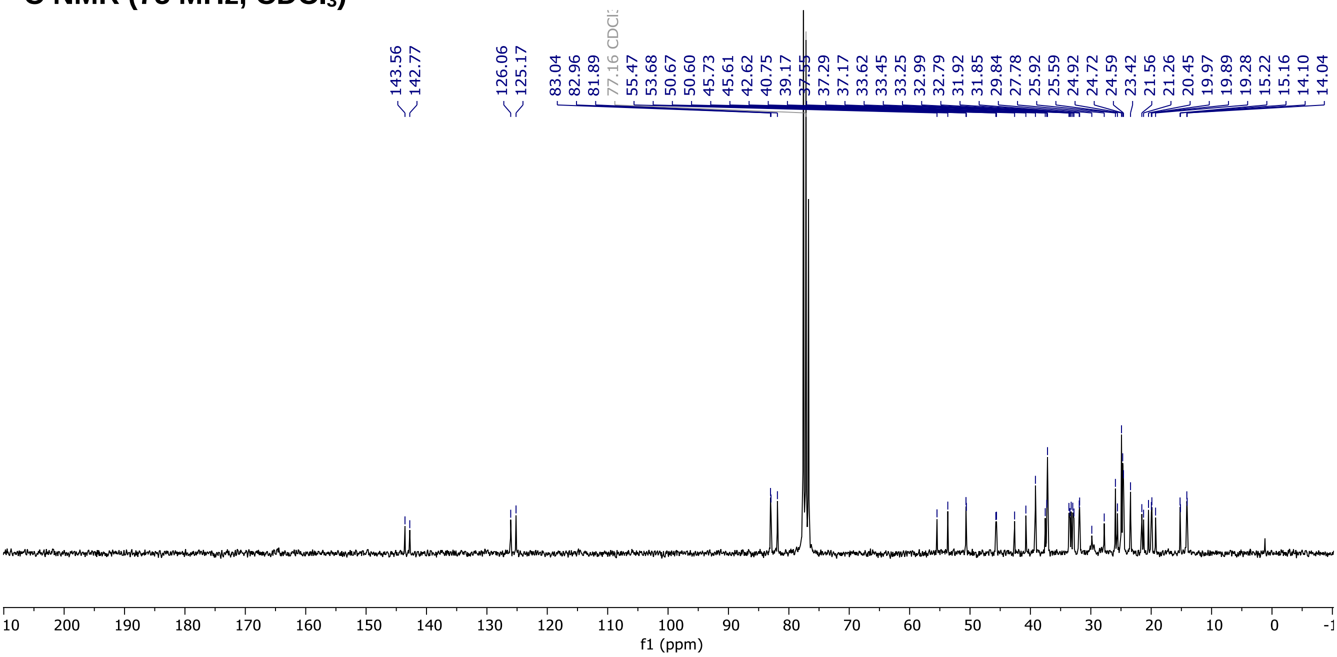

^{11}B NMR (129 MHz, CDCl$_3$)

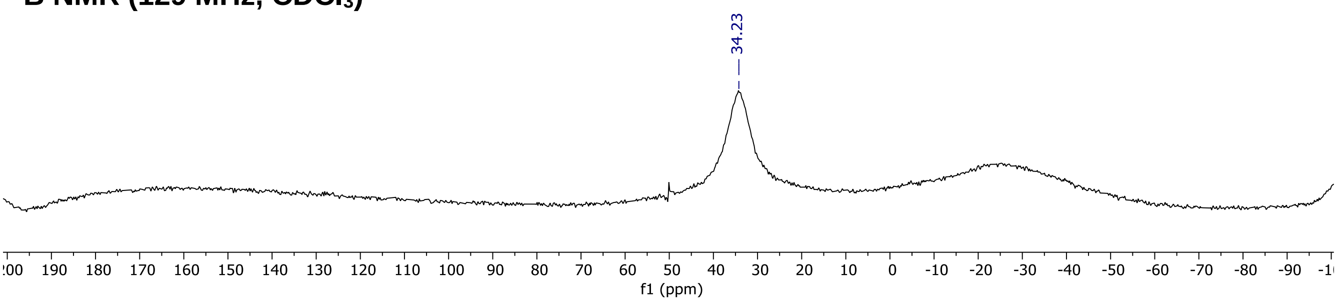

(8R,9S,10R,13S,14S,17S)-3-cyclopropyl-10,13,17-trimethyl-3-(4,4,5,5-tetramethyl-1,3,2-dioxaborolan-2-yl)-2,3,6,7,8,9,10,11,12,13,14,15,16,17-tetradecahydro-1H-cyclopenta[a]phenanthren-17-ol (4aa)

^{1}H NMR (300 MHz, CDCl$_3$)

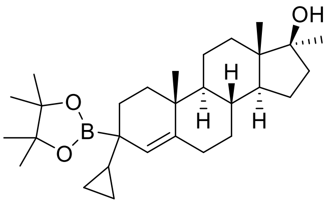

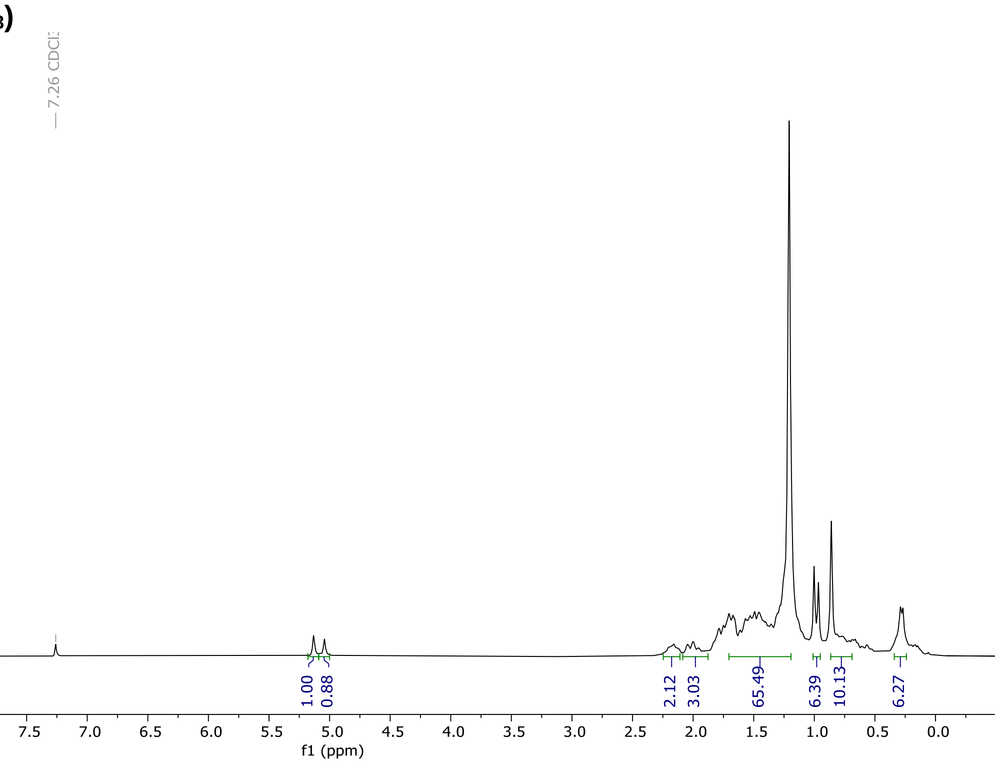

^{13}C NMR (75 MHz, CDCl$_3$)

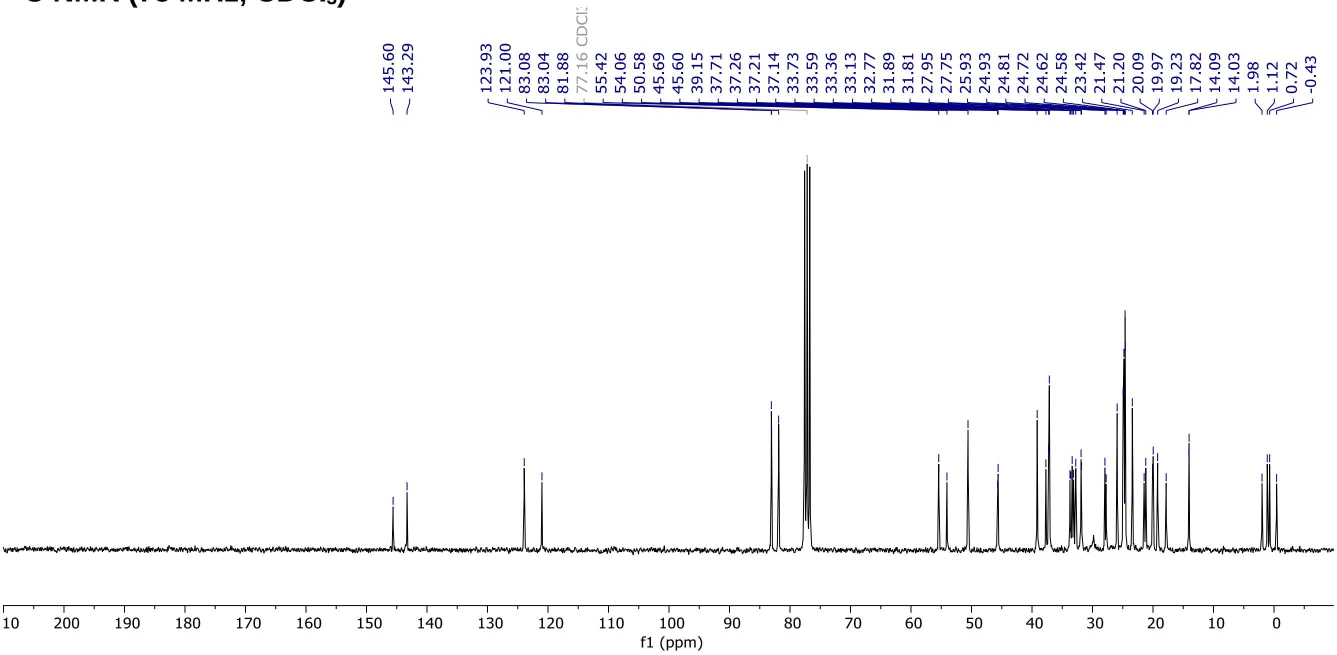

^{11}B NMR (129 MHz, CDCl$_3$)

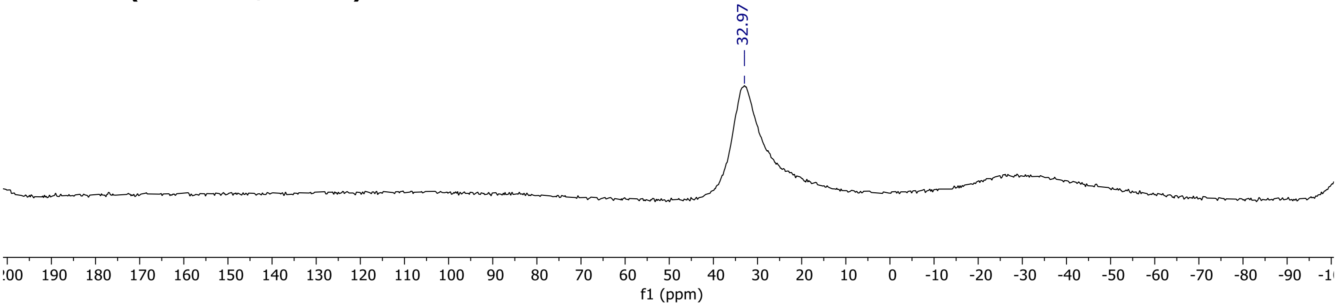

(8R,9S,10R,13S,14S,17S)-3-cyclobutyl-10,13,17-trimethyl-3-(4,4,5,5-tetramethyl-1,3,2-dioxaborolan-2-yl)-2,3,6,7,8,9,10,11,12,13,14,15,16,17-tetradecahydro-1H-cyclopenta[a]phenanthren-17-ol (4ab)

^{1}H NMR (300 MHz, $CDCl_3$)

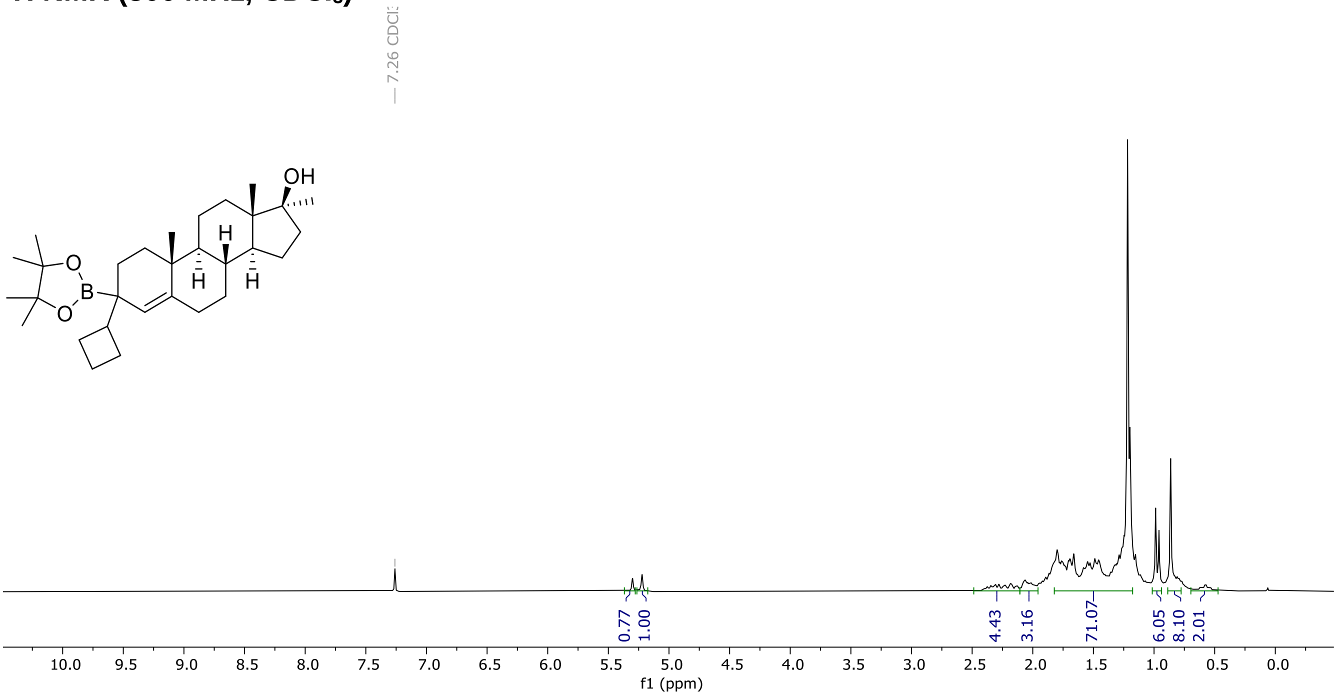

^{13}C NMR (75 MHz, $CDCl_3$)

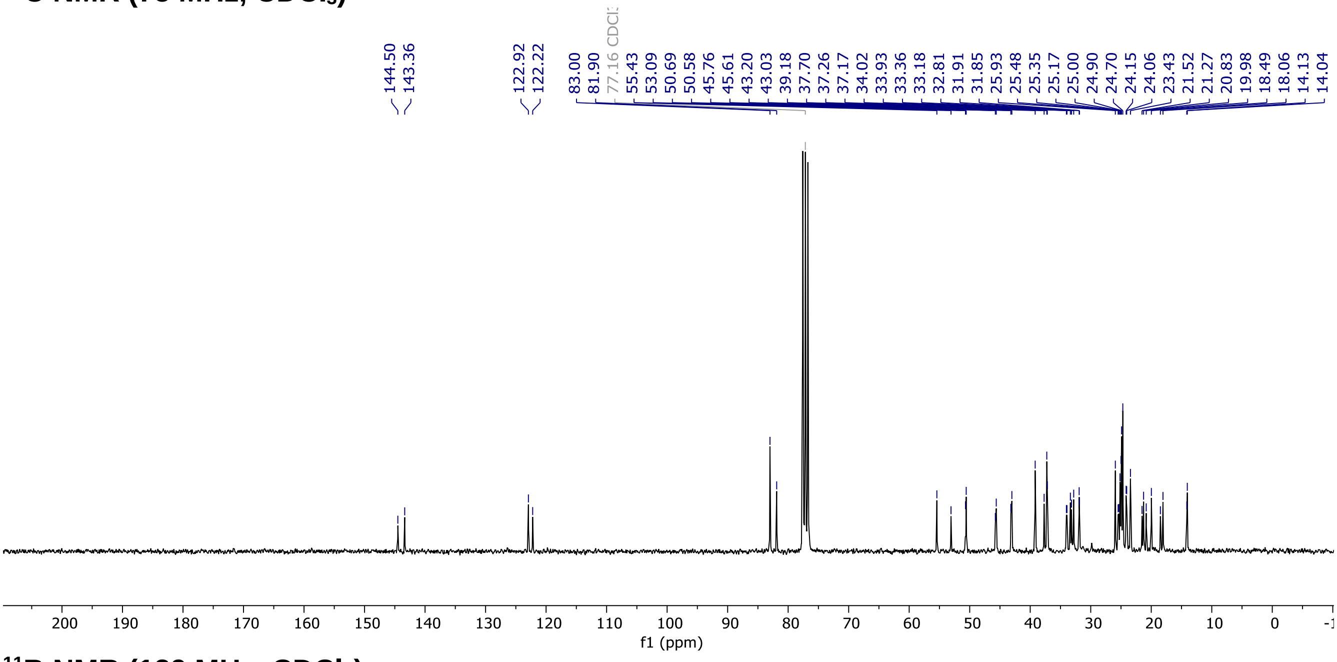

^{11}B NMR (129 MHz, $CDCl_3$)

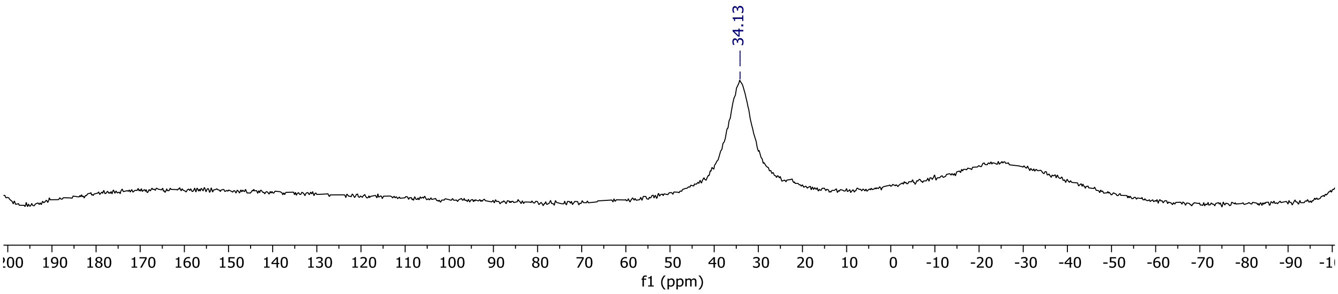

2-((8S,9S,10R,13R,14S,17R)-10,13-dimethyl-17-((R)-6-methylheptan-2-yl)-3-propyl-2,3,6,7,8,9,10,11,12,13,14,15,16,17-tetradecahydro-1H-cyclopenta[a]phenanthren-3-yl)-4,4,5,5-tetramethyl-1,3,2-dioxaborolane (4ac)

^{1}H NMR (300 MHz, CDCl$_3$)

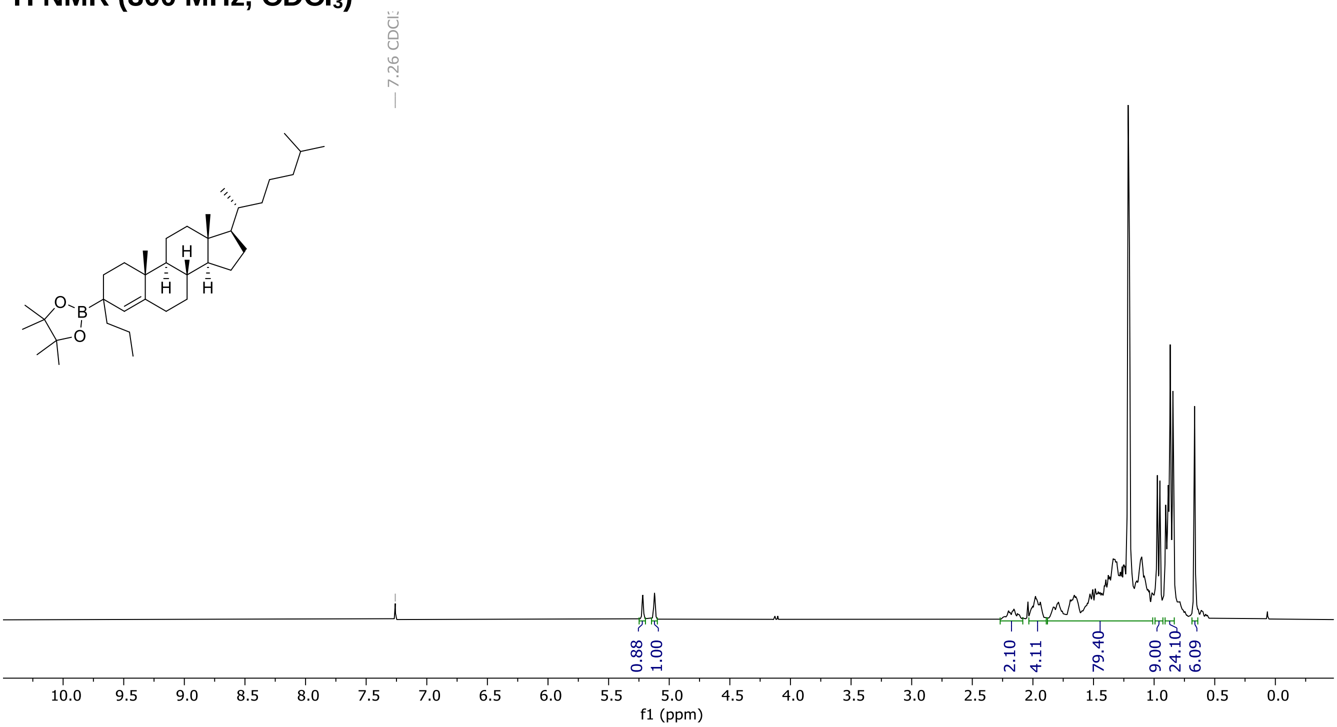

^{13}C NMR (75 MHz, CDCl$_3$)

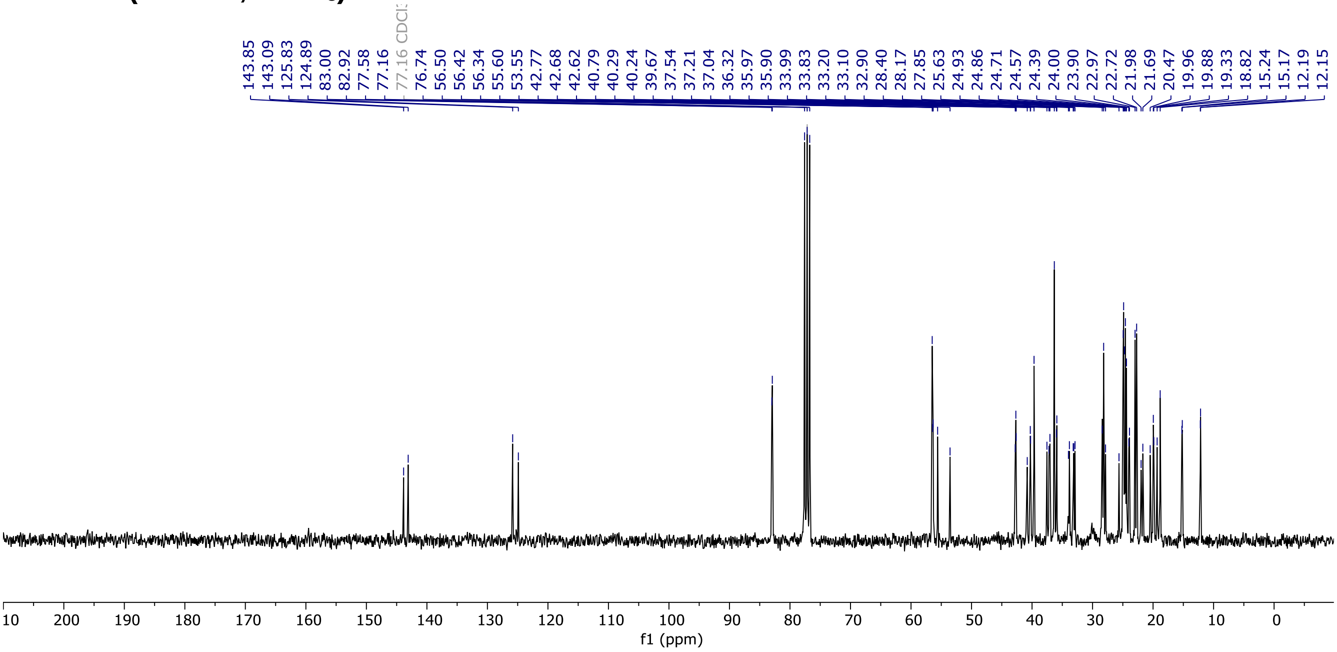

^{11}B NMR (129 MHz, CDCl$_3$)

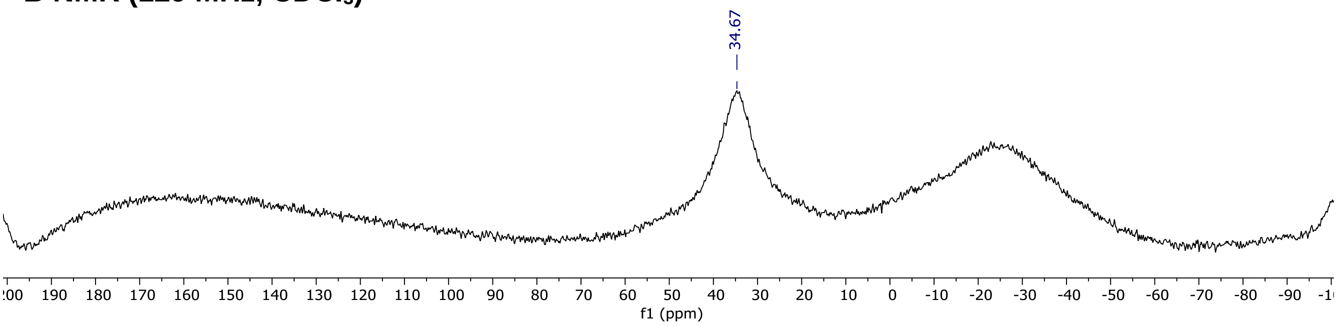

(Z)-4,4,5,5-tetramethyl-2-(3-methyl-2-(pent-2-en-1-yl)-1-propylcyclopent-2-en-1-yl)-1,3,2-dioxaborolane (4ad)

^{1}H NMR (401 MHz, $CDCl_3$)

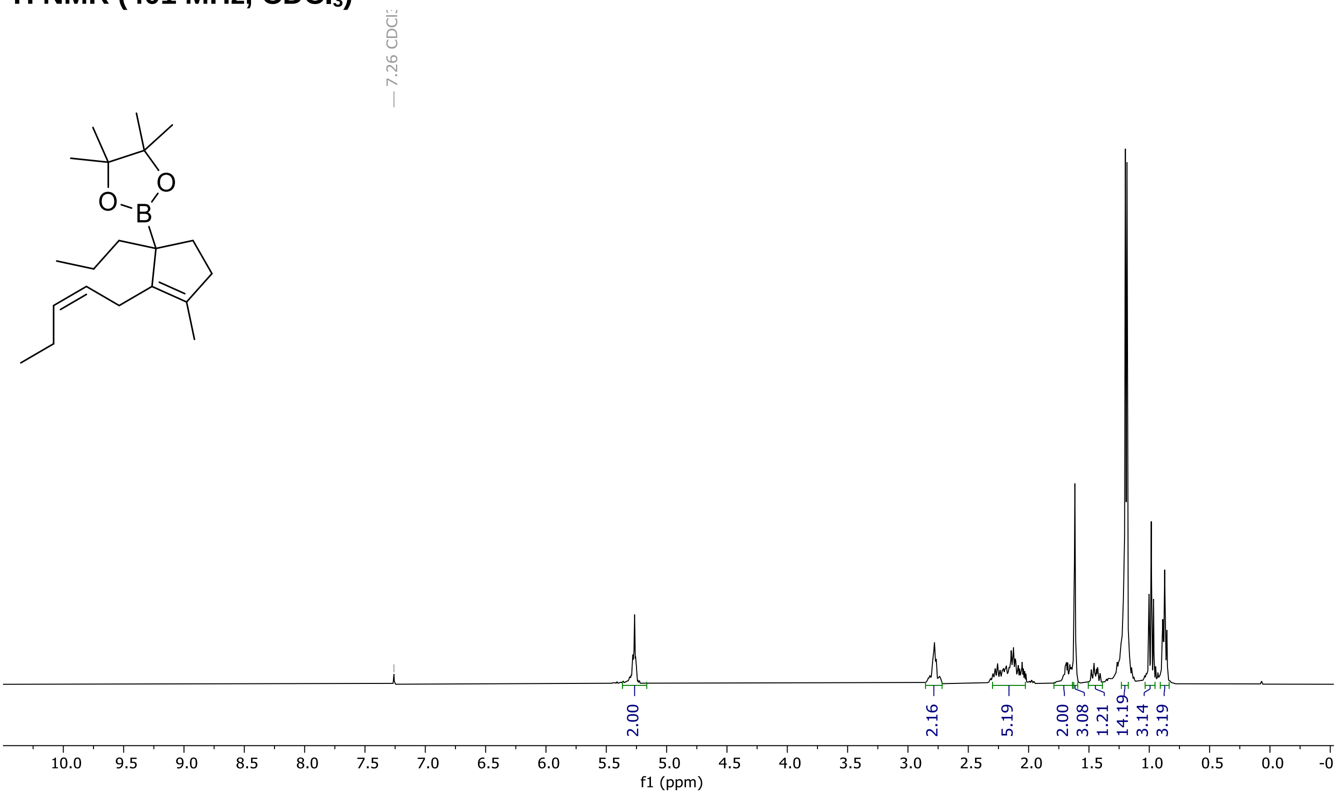

^{13}C NMR (101 MHz, $CDCl_3$)

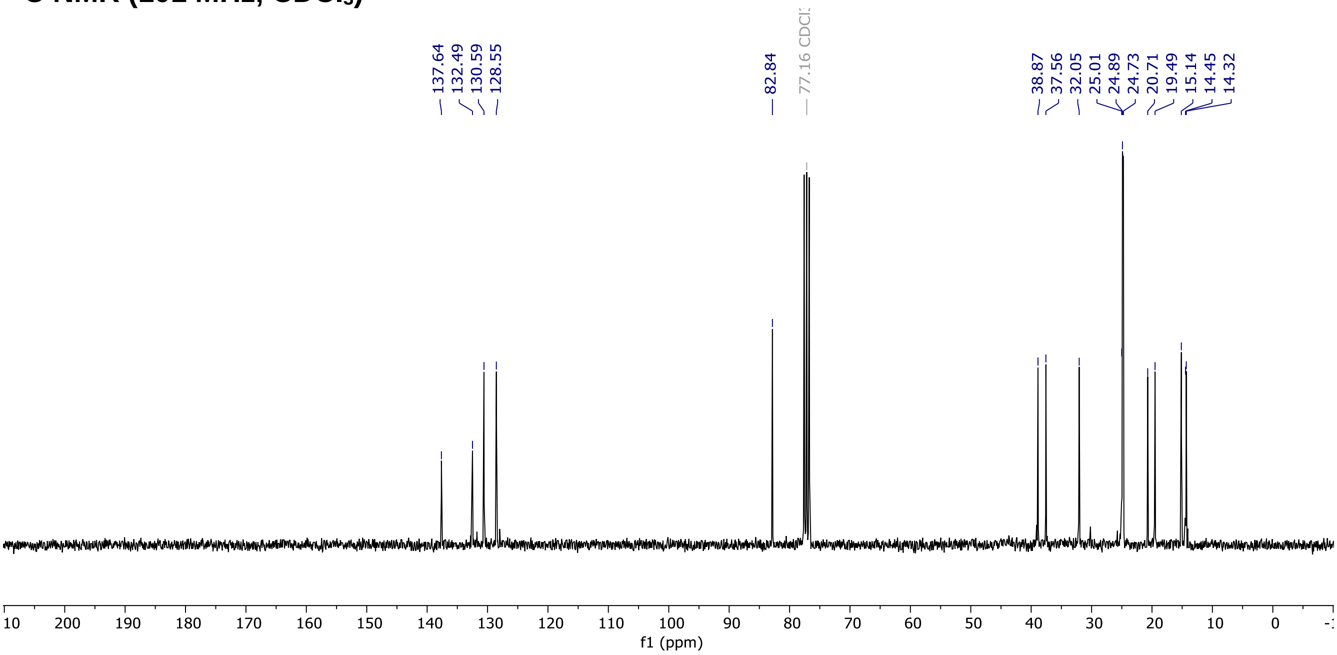

^{11}B NMR (129 MHz, $CDCl_3$)

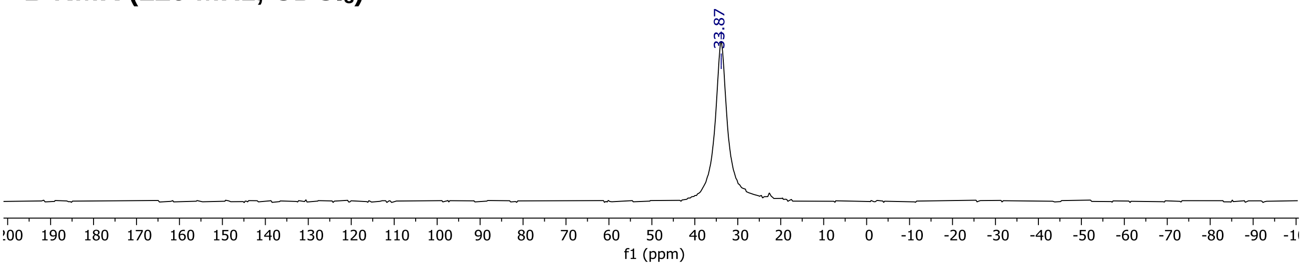

2-((4R,4aS,6R)-4,4a-dimethyl-6-(prop-1-en-2-yl)-2-propyl-2,3,4,4a,5,6,7,8-octahydronaphthalen-2-yl)-4,4,5,5-tetramethyl-1,3,2-dioxaborolane (4ae)

^{1}H NMR (300 MHz, $CDCl_3$)

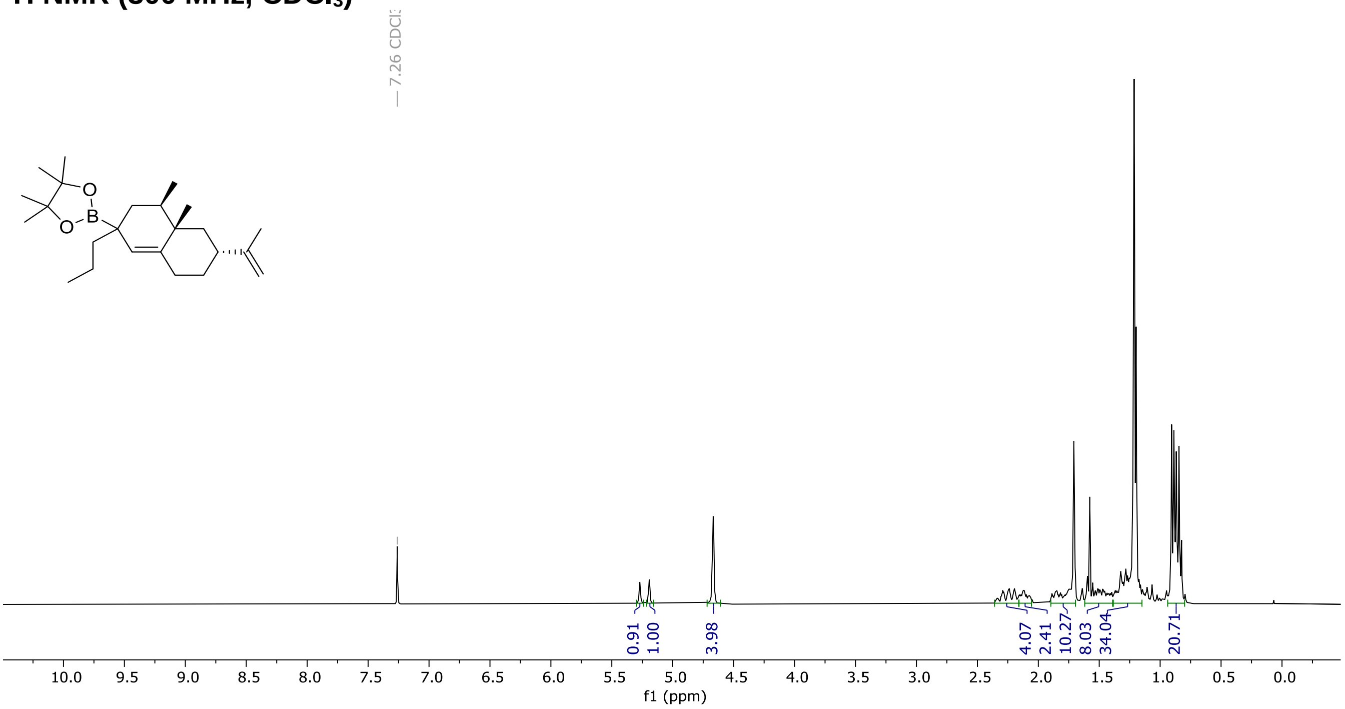

^{13}C NMR (75 MHz, $CDCl_3$)

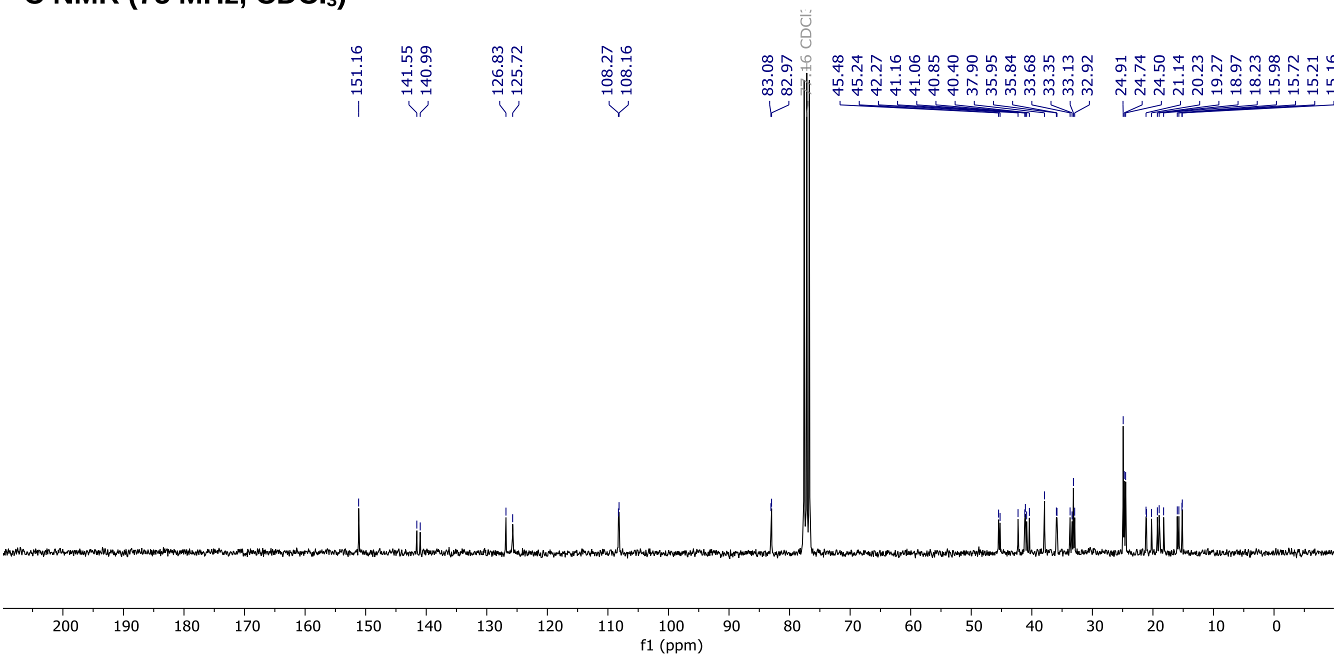

^{11}B NMR (129 MHz, $CDCl_3$)

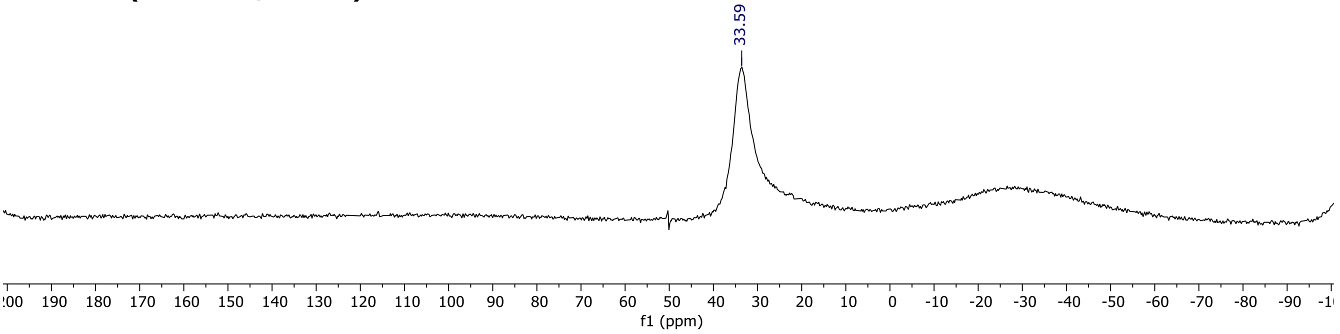

4,4,5,5-tetramethyl-2-((1S,2S,5R)-2,4,6,6-tetramethylbicyclo[3.1.1]hept-3-en-2-yl)-1,3,2-dioxaborolane (4af)

^{1}H NMR (300 MHz, $CDCl_3$)

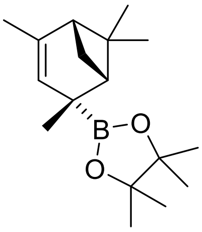

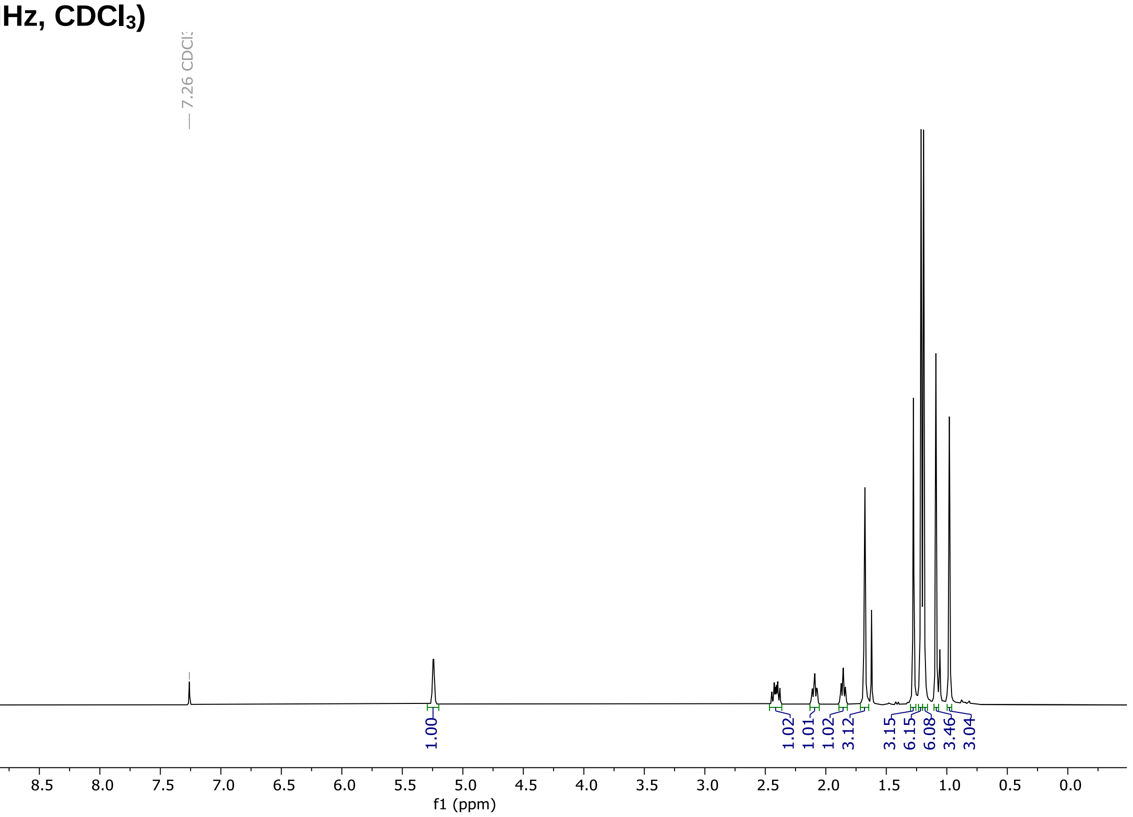

^{13}C NMR (75 MHz, $CDCl_3$)

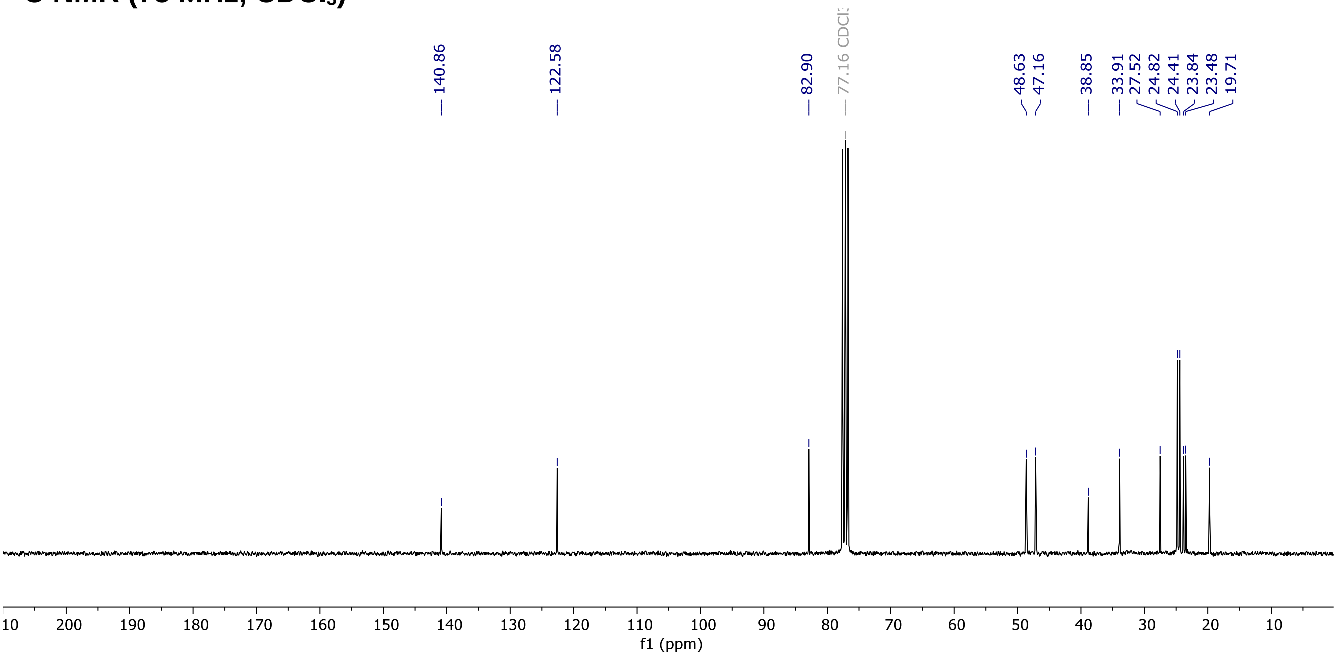

^{11}B NMR (129 MHz, $CDCl_3$)

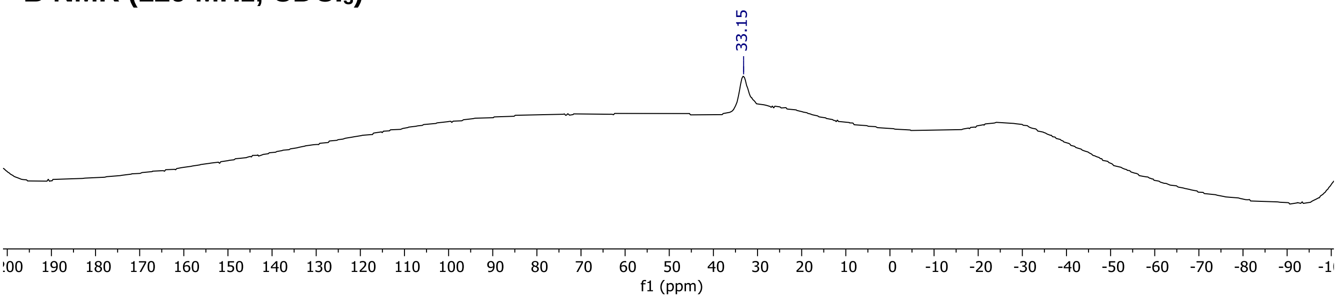

(*R**)-((*R**)-6,6-dimethyl-3-propylcyclohex-2-en-1-yl)(p-tolyl)methanol (5a)

^{1}H NMR (300 MHz, $CDCl_3$)

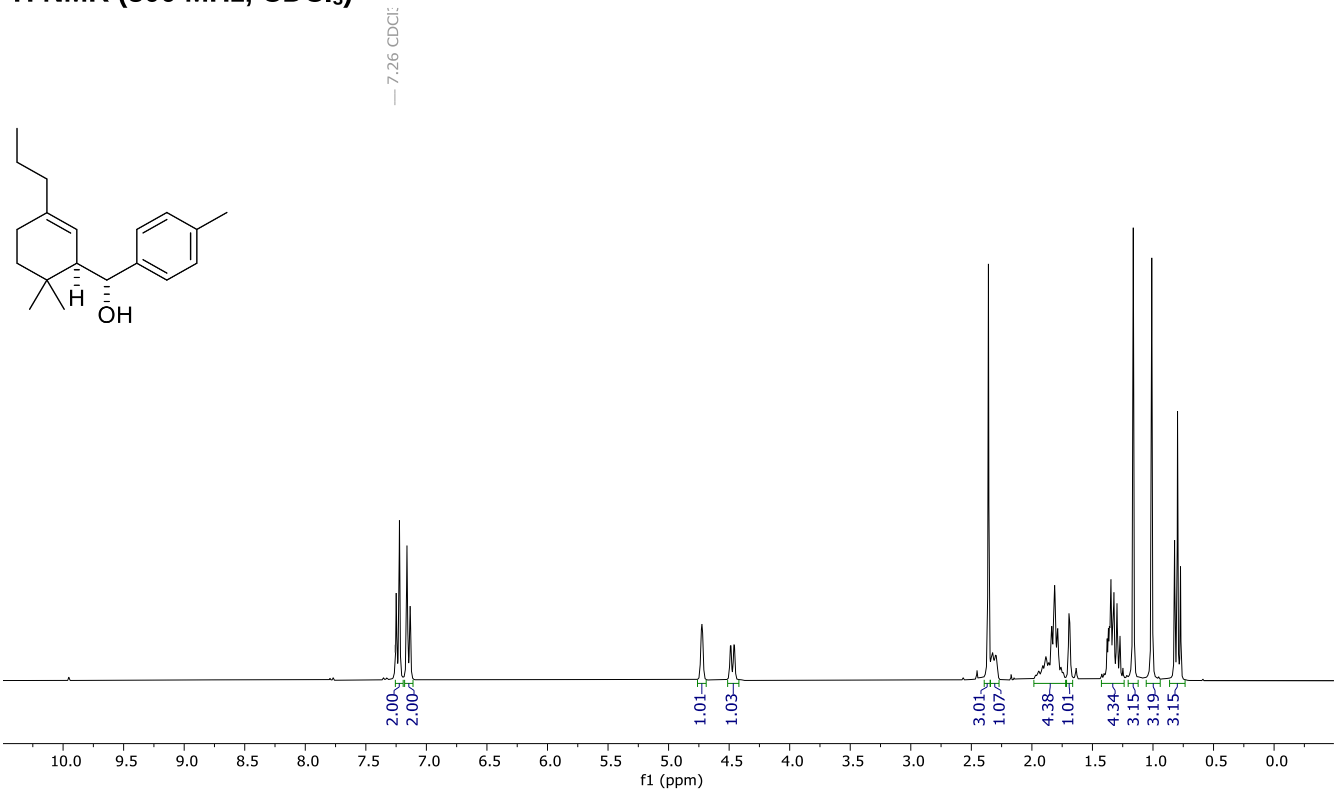

^{13}C NMR (75 MHz, $CDCl_3$)

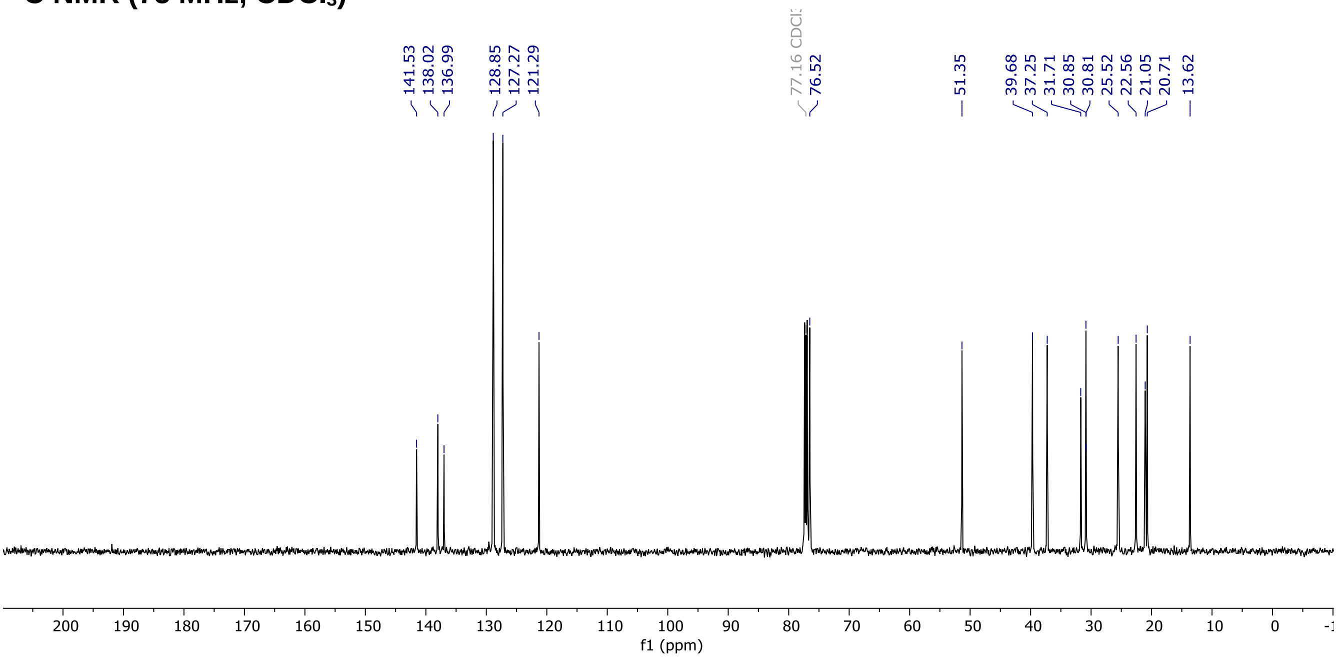

(*R)-((*R**)-6,6-dimethyl-3-propylcyclohex-2-en-1-yl)(4-methoxyphenyl)methanol (5b)**

^{1}H NMR (300 MHz, $CDCl_3$)

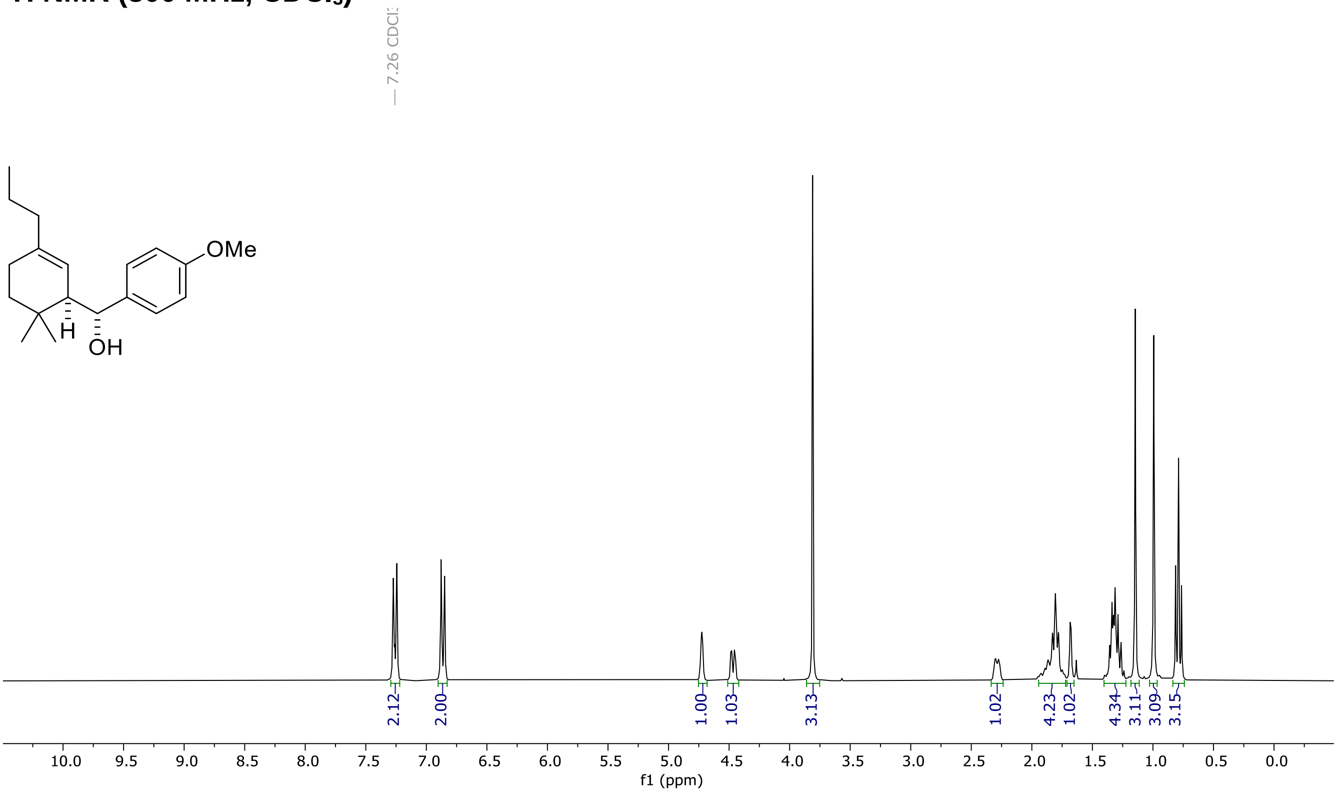

^{13}C NMR (75 MHz, $CDCl_3$)

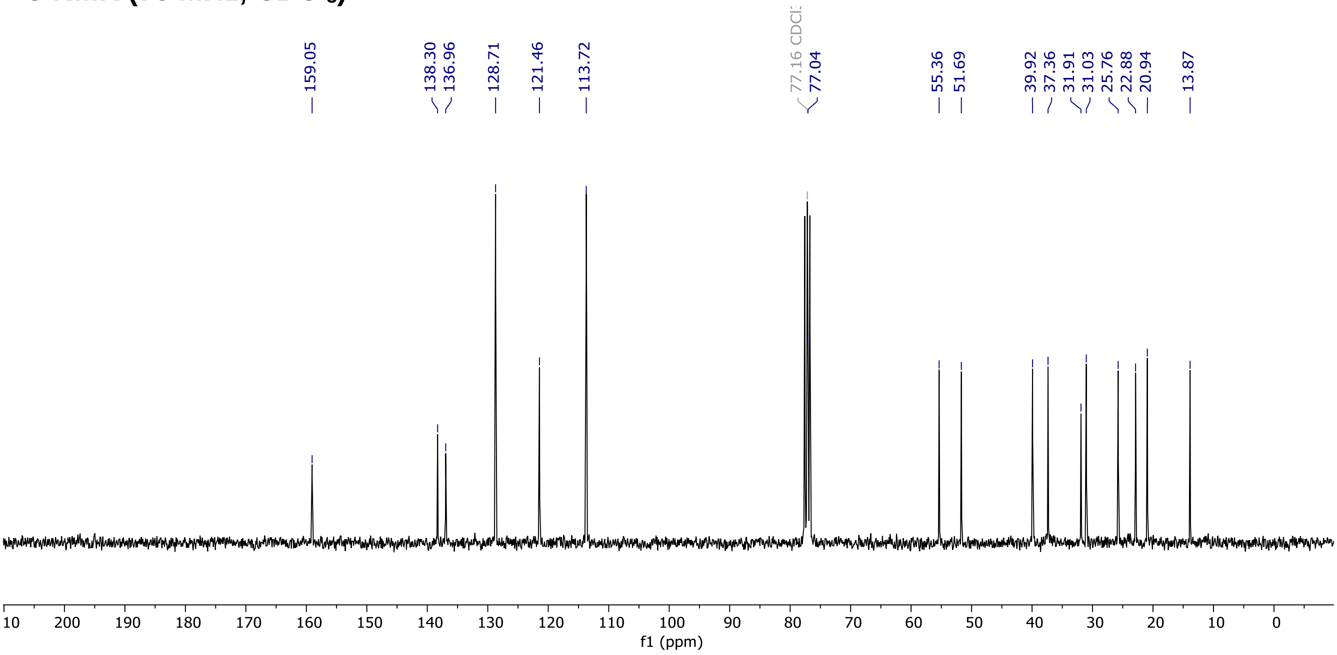

(*R**)-((*R**)-6,6-dimethyl-3-propylcyclohex-2-en-1-yl)(4-nitrophenyl)methanol (5c)

^{1}H NMR (300 MHz, $CDCl_3$)

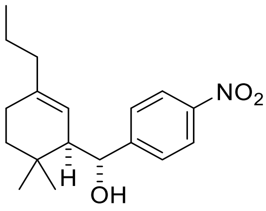

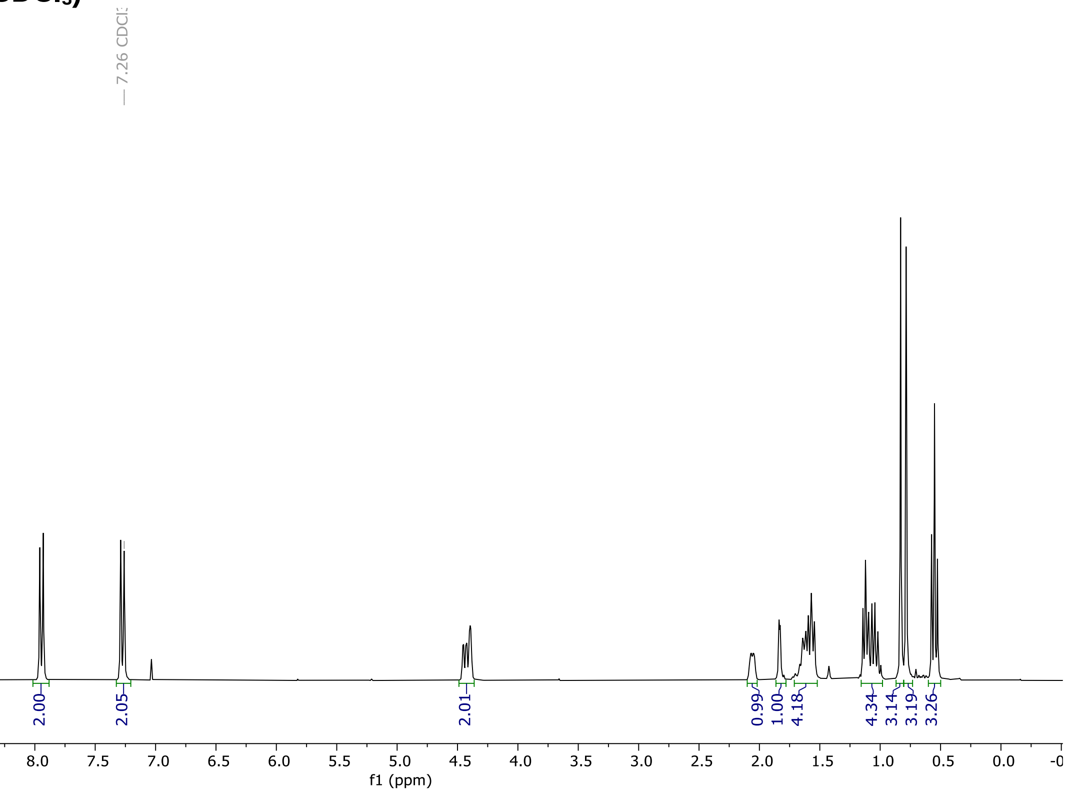

^{13}C NMR (75 MHz, $CDCl_3$)

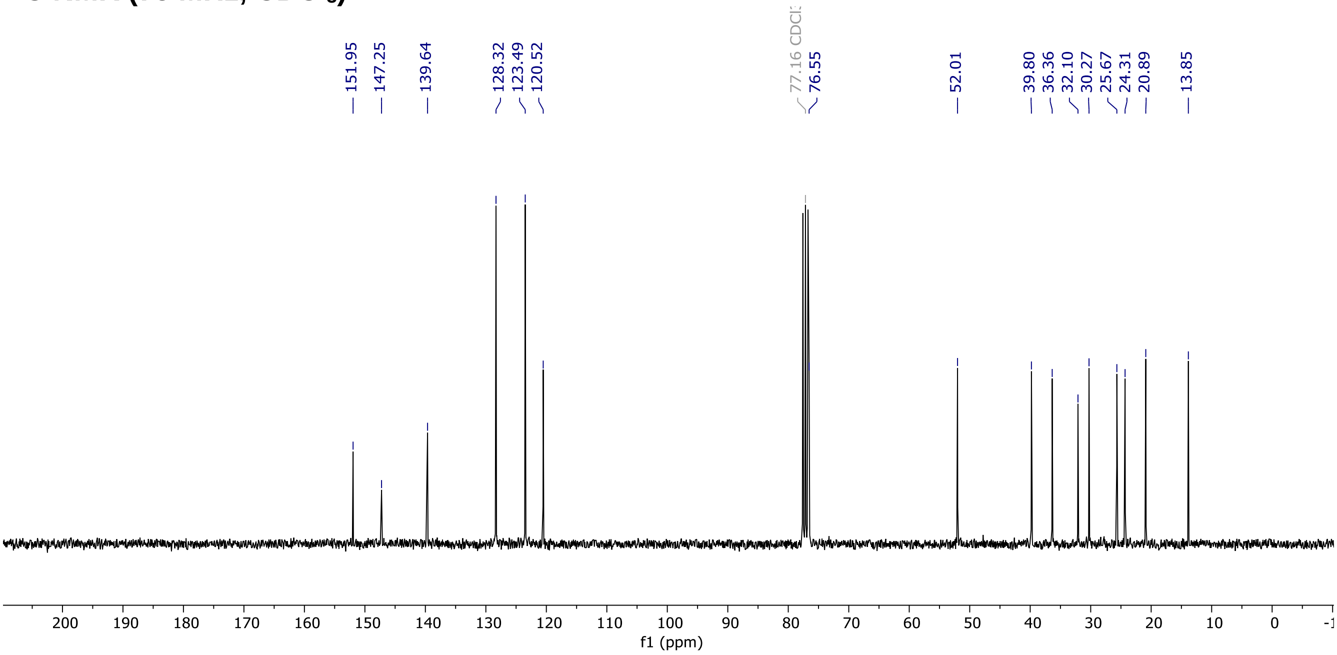

4-((*R**)-((*R**)-6,6-dimethyl-3-propylcyclohex-2-en-1-yl)(hydroxy)methyl)benzonitrile (5d)

^{1}H NMR (300 MHz, $CDCl_3$)

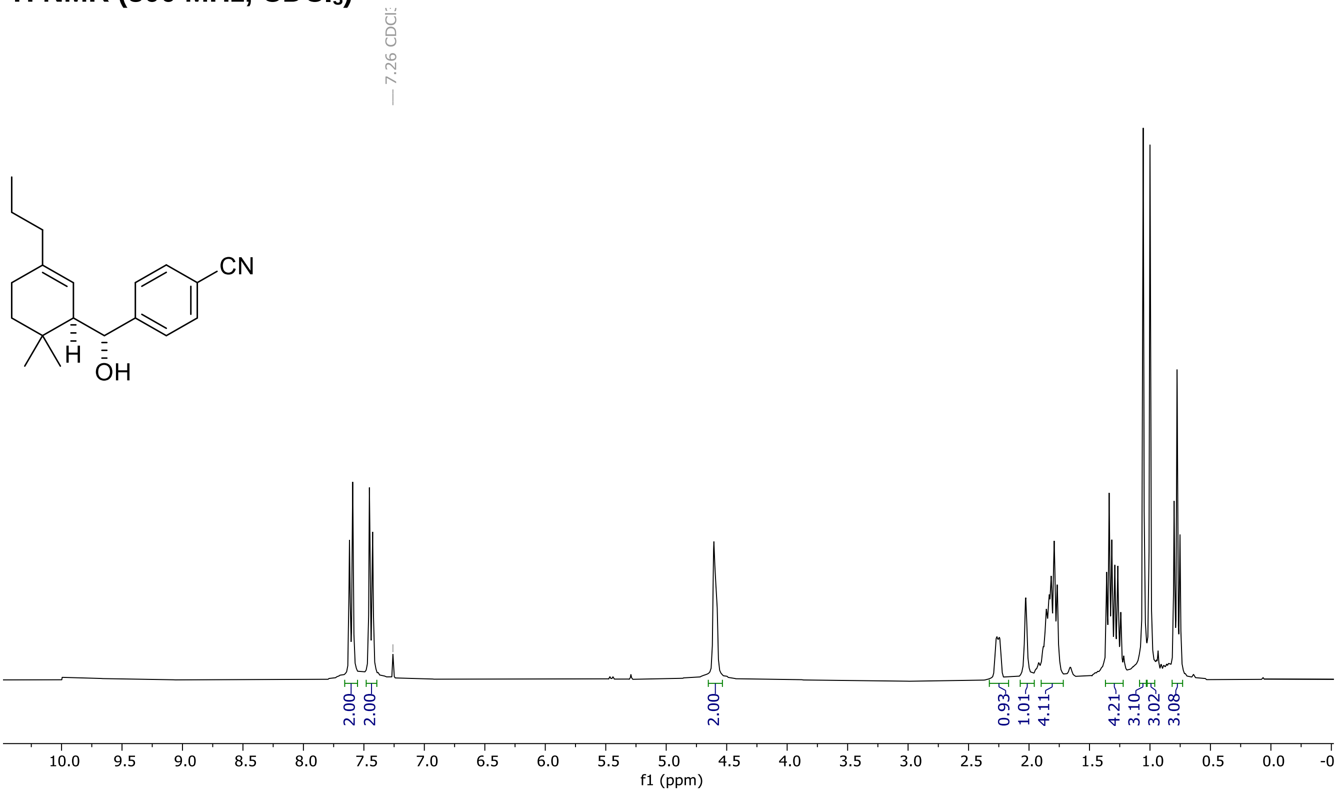

^{13}C NMR (75 MHz, $CDCl_3$)

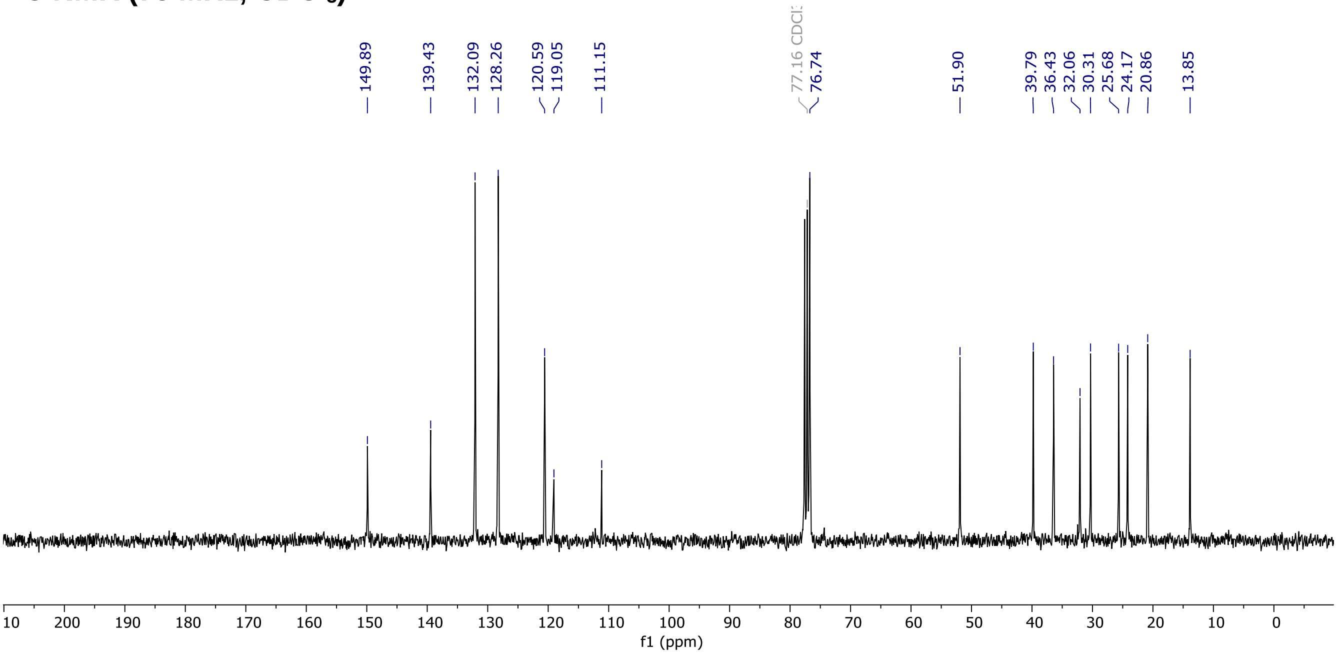

(*R)-((*R**)-6,6-dimethyl-3-propylcyclohex-2-en-1-yl)(4-fluorophenyl)methanol (5e)**

^{1}H NMR (300 MHz, CDCl$_3$)

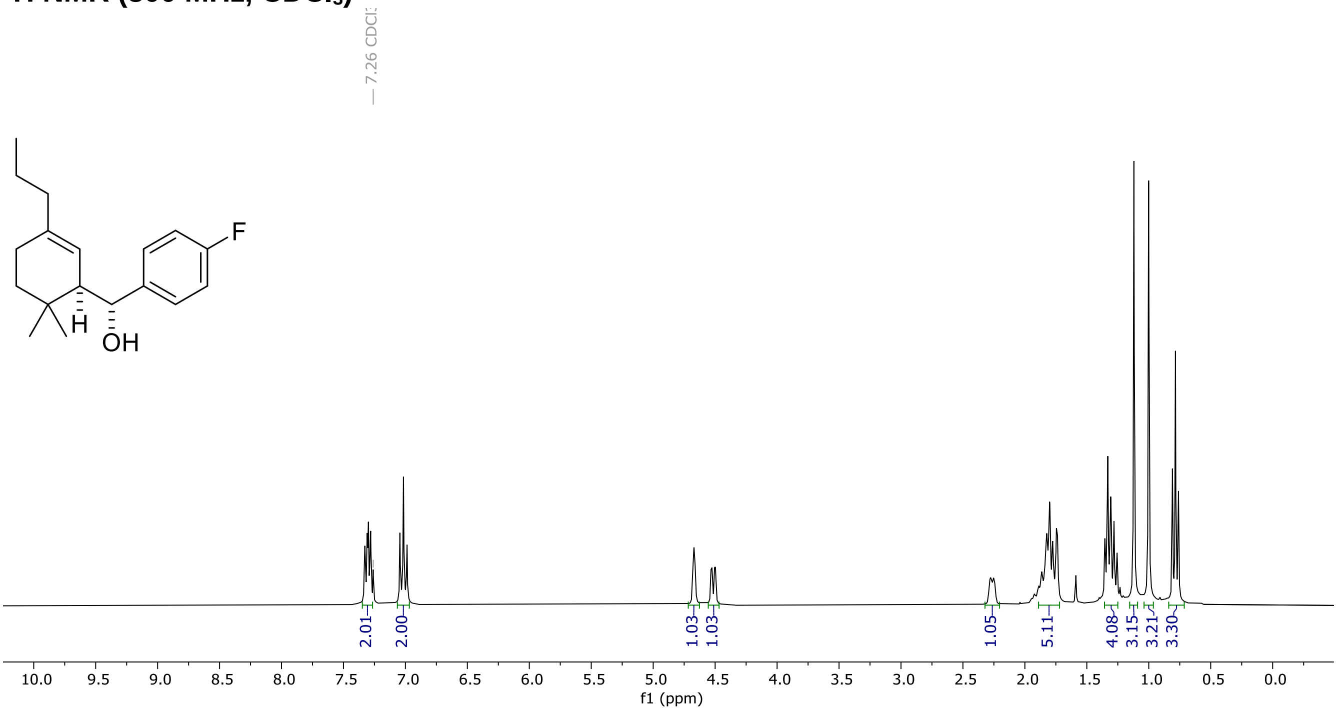

^{13}C NMR (75 MHz, CDCl$_3$)

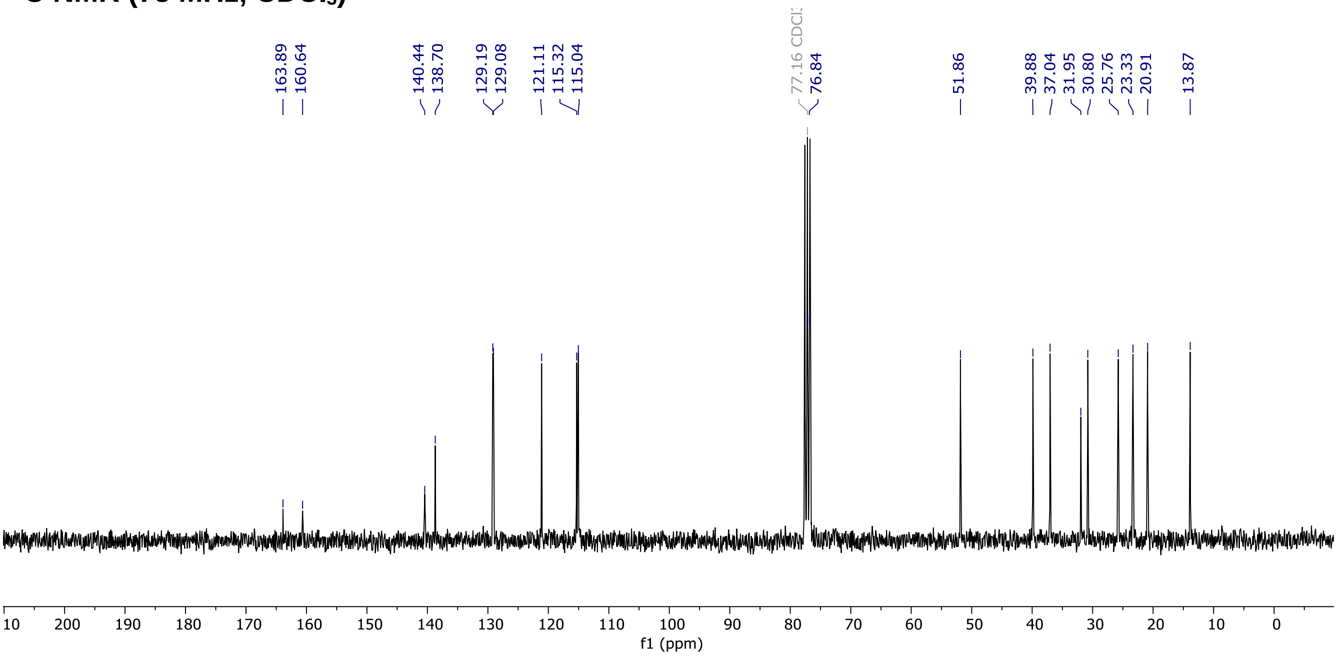

^{19}F NMR (282 MHz, CDCl$_3$)

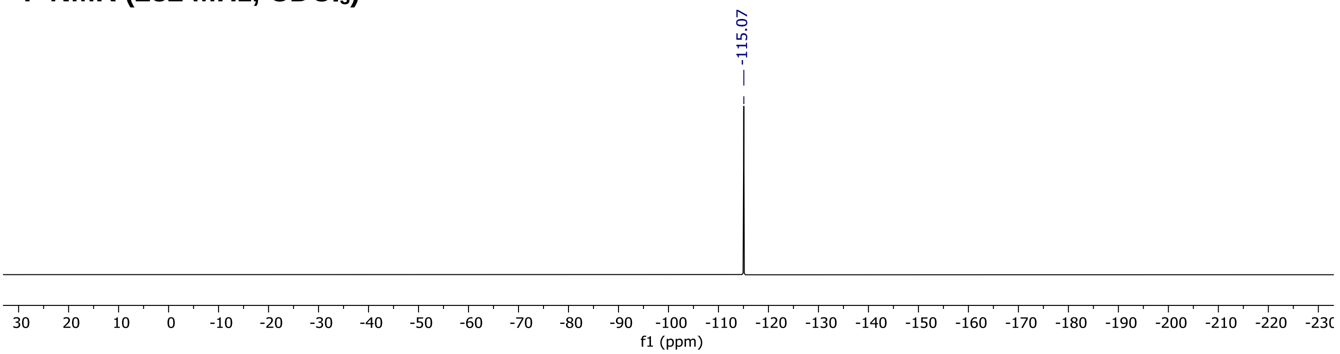

(*R**)-((*R**)-(4-bromophenyl)(-6,6-dimethyl-3-propylcyclohex-2-en-1-yl)methanol (5f)

^{1}H NMR (300 MHz, $CDCl_3$)

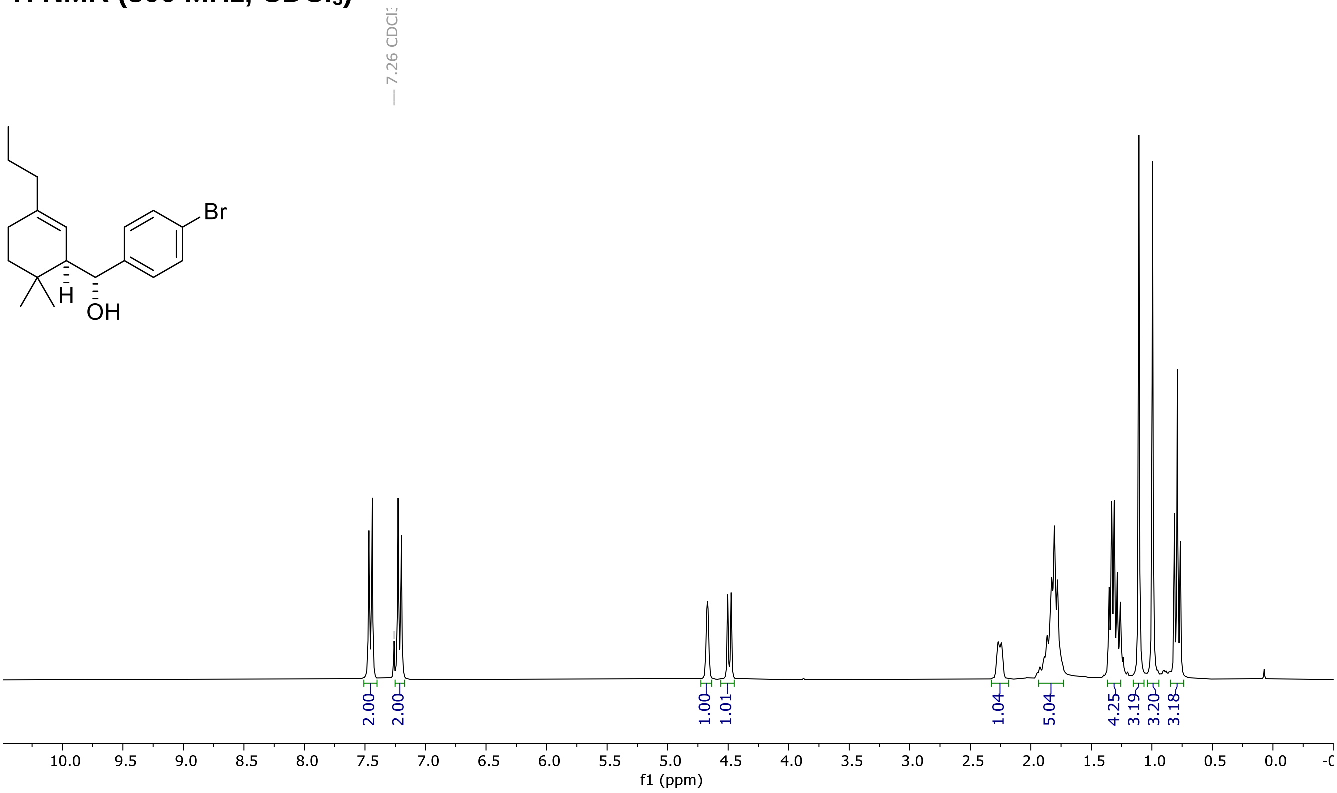

^{13}C NMR (75 MHz, $CDCl_3$)

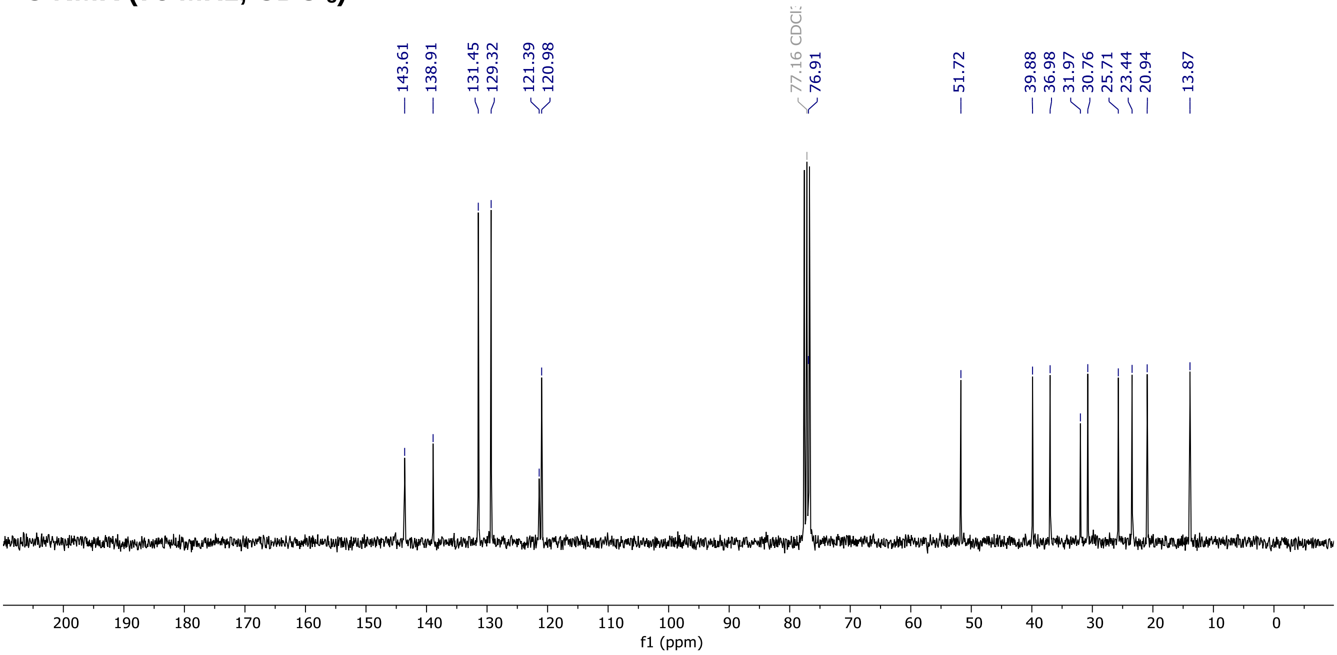

(*R**)-(2,6-dichlorophenyl)((*R**)-6,6-dimethyl-3-propylcyclohex-2-en-1-yl)methanol (5g)

^{1}H NMR (300 MHz, $CDCl_3$)

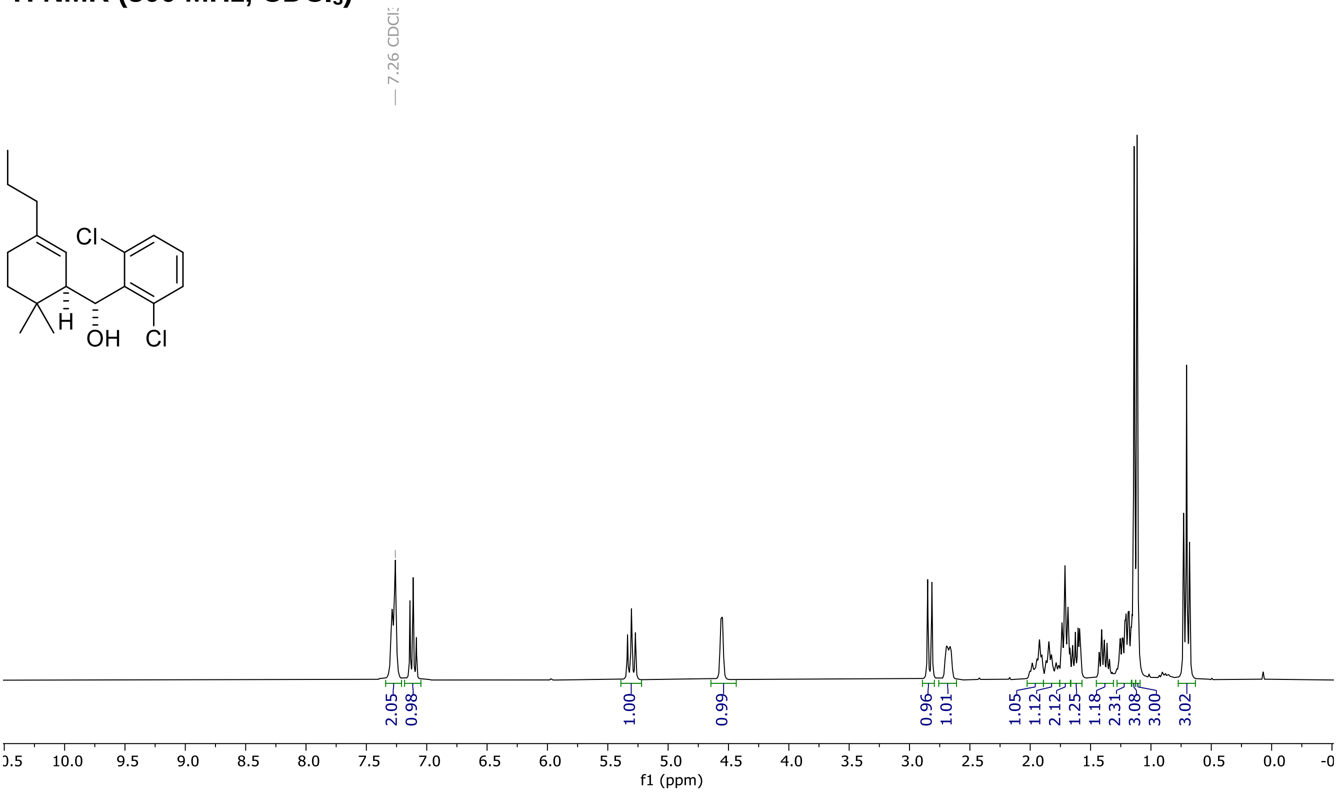

^{13}C NMR (75 MHz, $CDCl_3$)

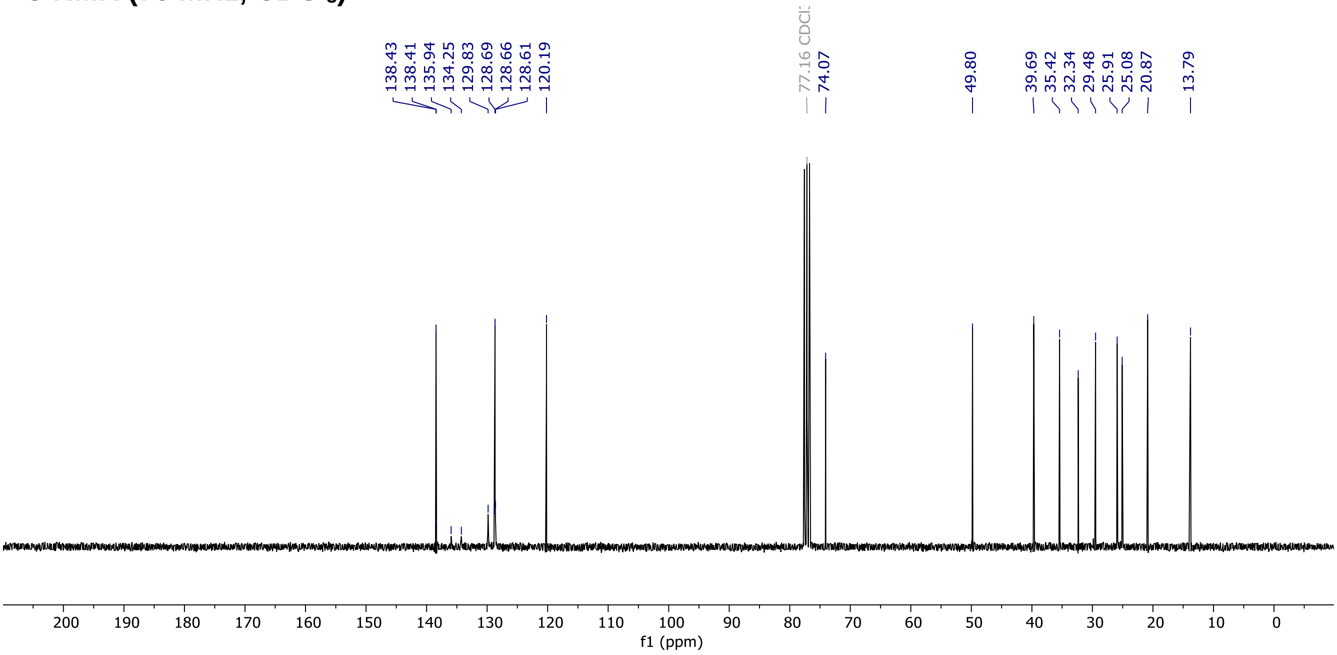

(*R**)-((*R**)-6,6-dimethyl-3-propylcyclohex-2-en-1-yl)(3,4,5-trimethoxyphenyl)methanol (5h)

^{1}H NMR (300 MHz, $CDCl_3$)

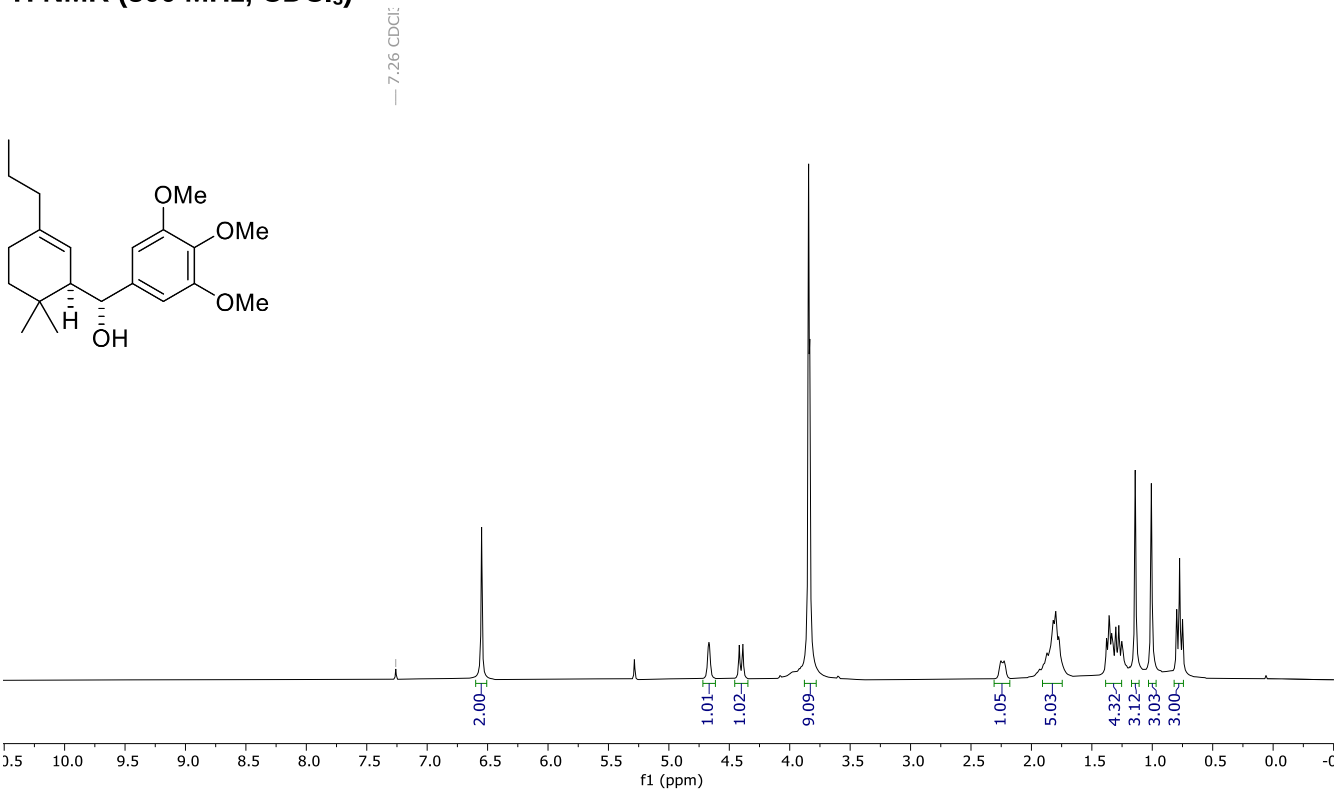

^{13}C NMR (75 MHz, $CDCl_3$)

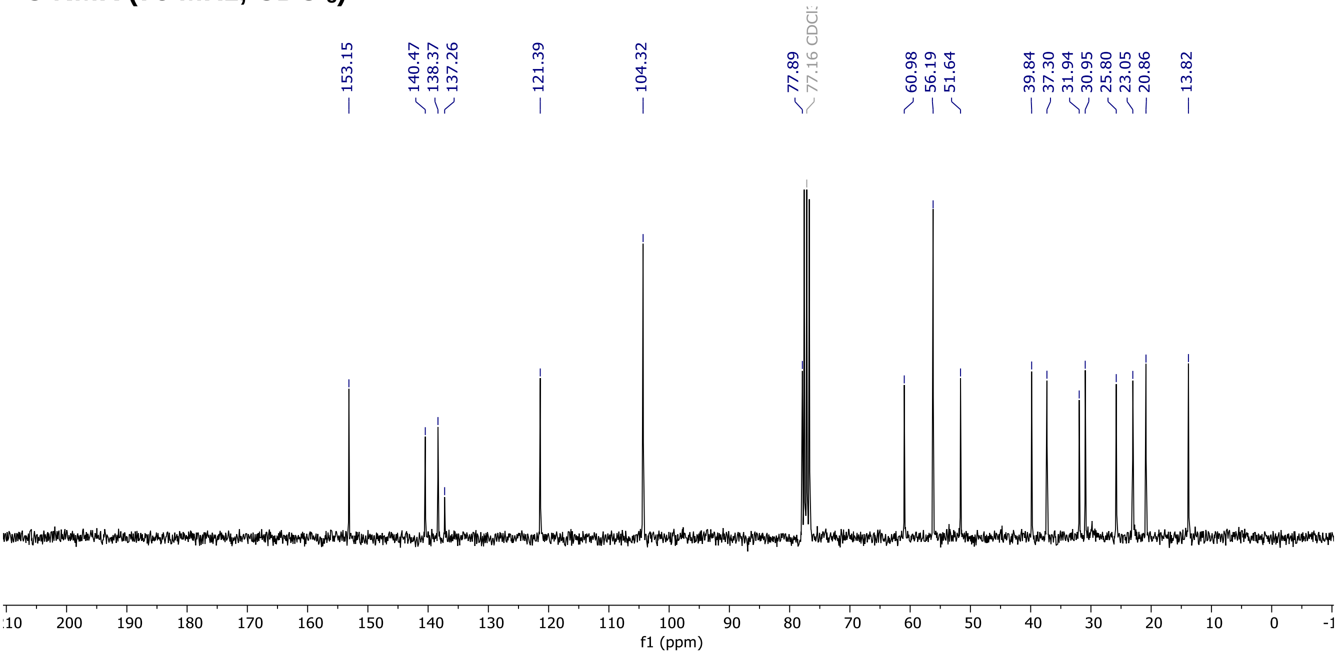

(*R**)-benzofuran-2-yl((*R**)-6,6-dimethyl-3-propylcyclohex-2-en-1-yl)methanol (5i)

^{1}H NMR (300 MHz, $CDCl_3$)

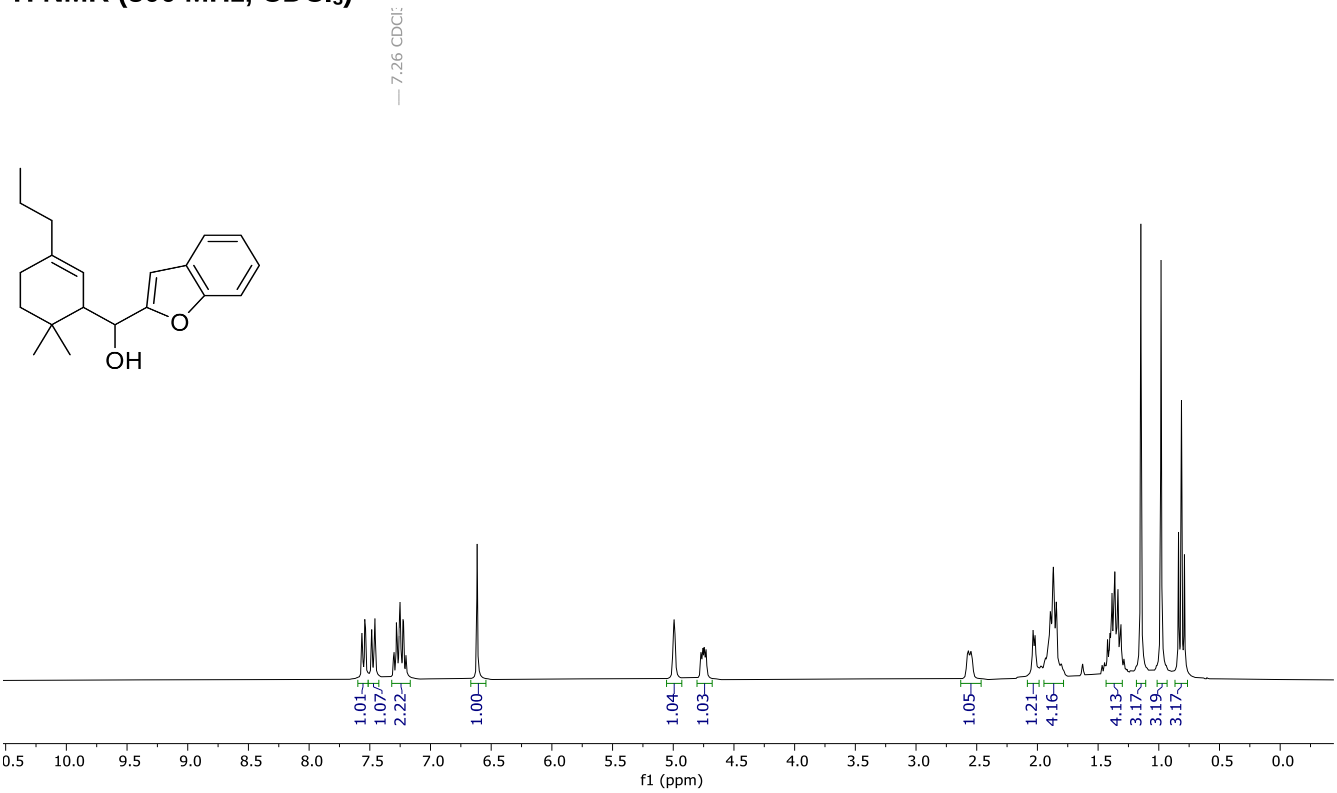

^{13}C NMR (75 MHz, $CDCl_3$)

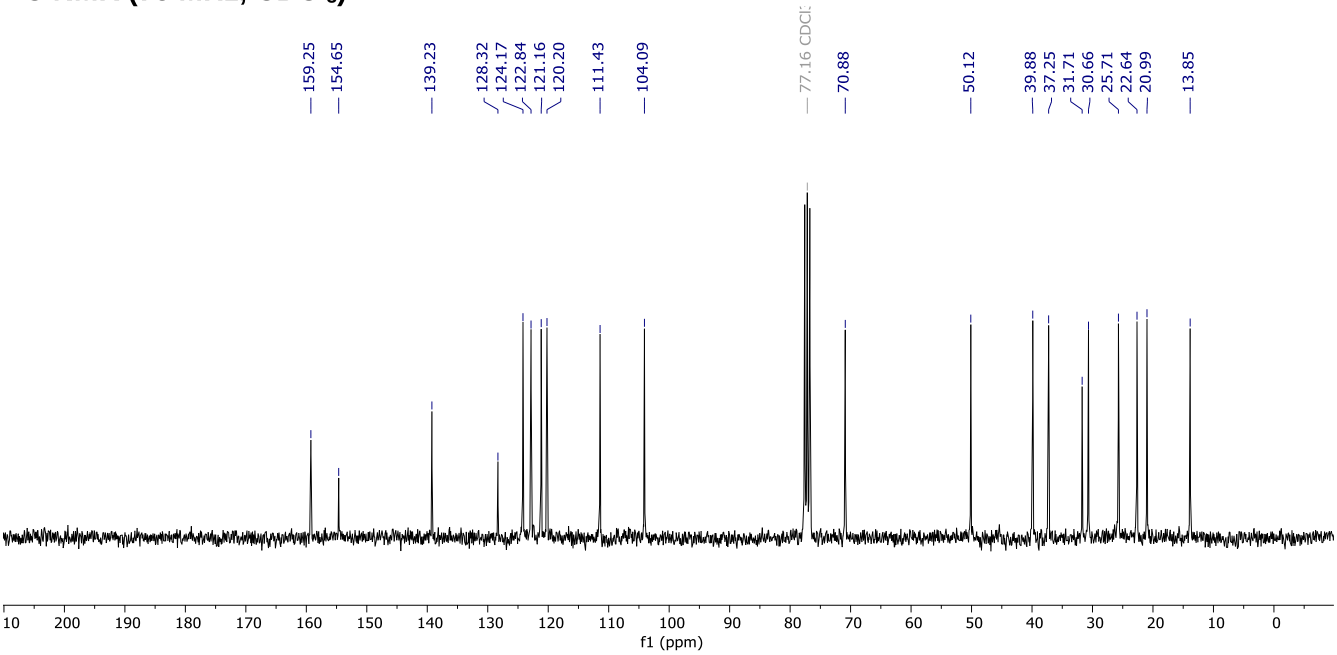

(*R)-((*R**)-6,6-dimethyl-3-propylcyclohex-2-en-1-yl)(thiophen-2-yl)methanol (5j)**

^{1}H NMR (300 MHz, $CDCl_3$)

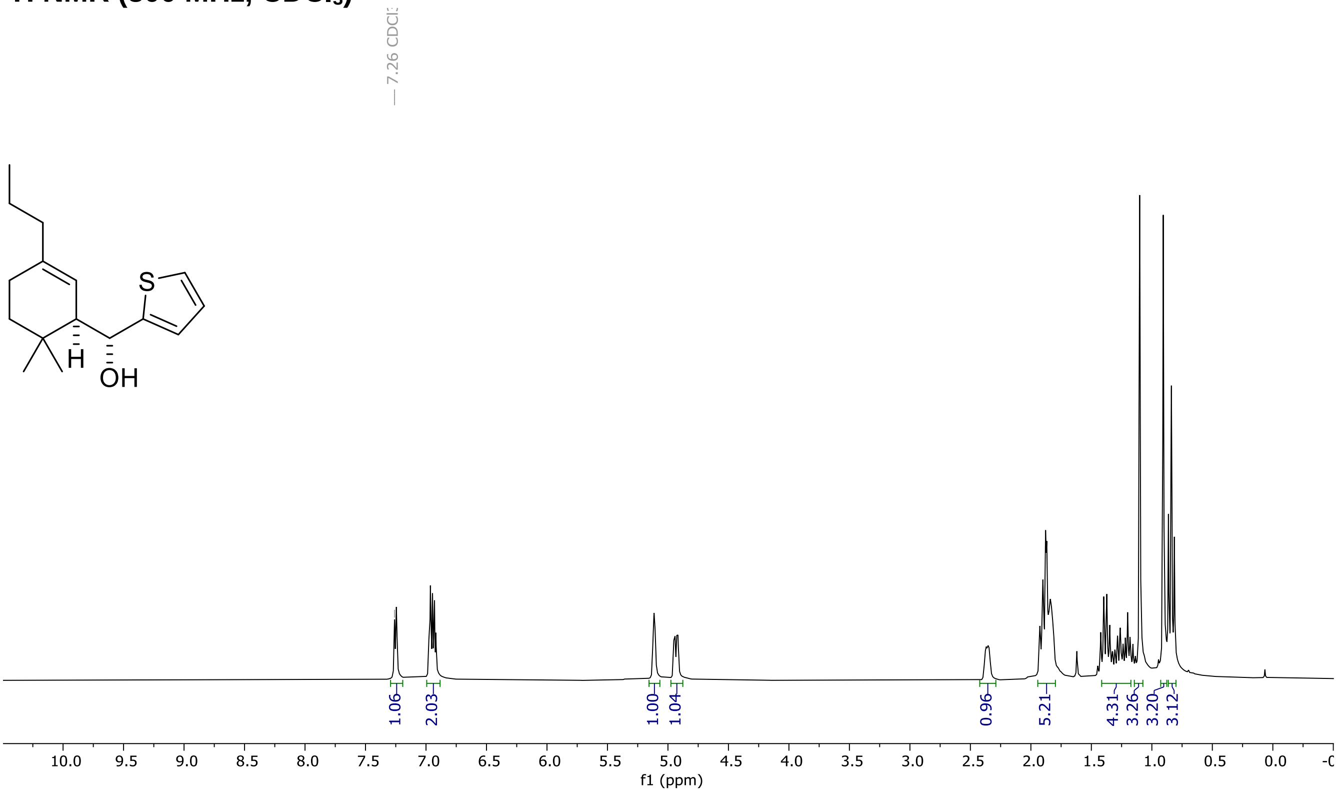

^{13}C NMR (75 MHz, $CDCl_3$)

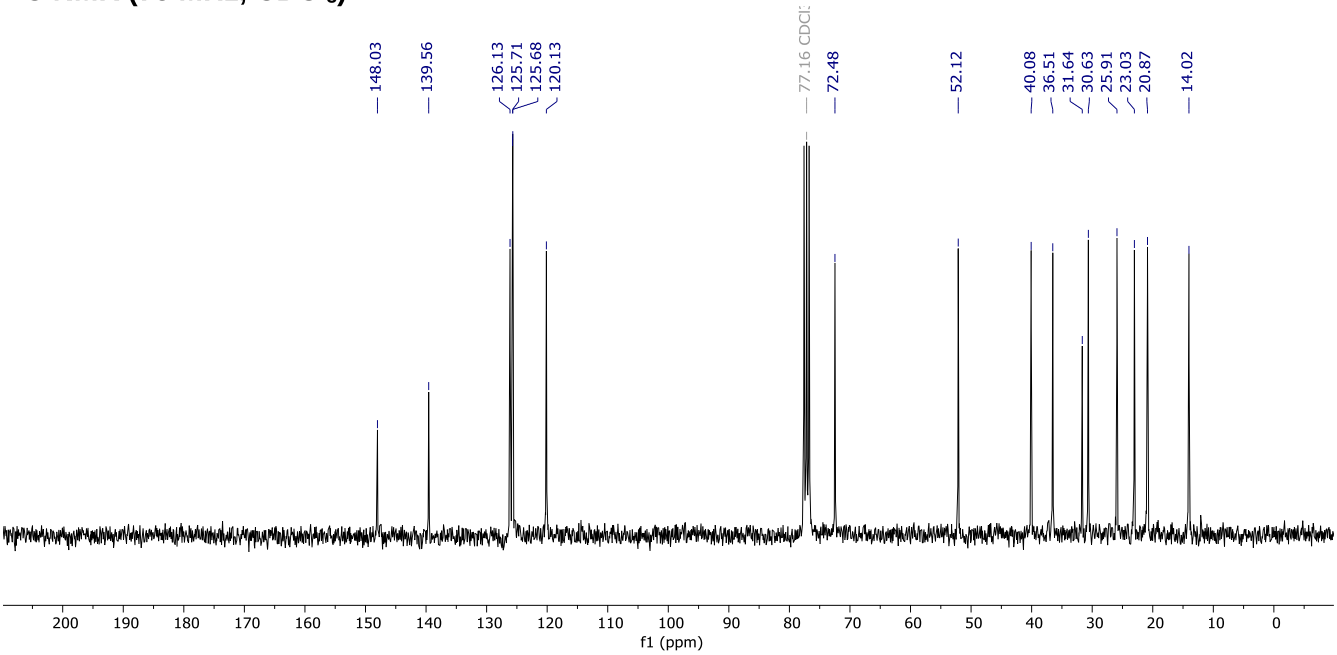

(*R)-((*R**)-6,6-dimethyl-3-propylcyclohex-2-en-1-yl)(pyridin-4-yl)methanol (5k)**

^{1}H NMR (300 MHz, CDCl$_3$)

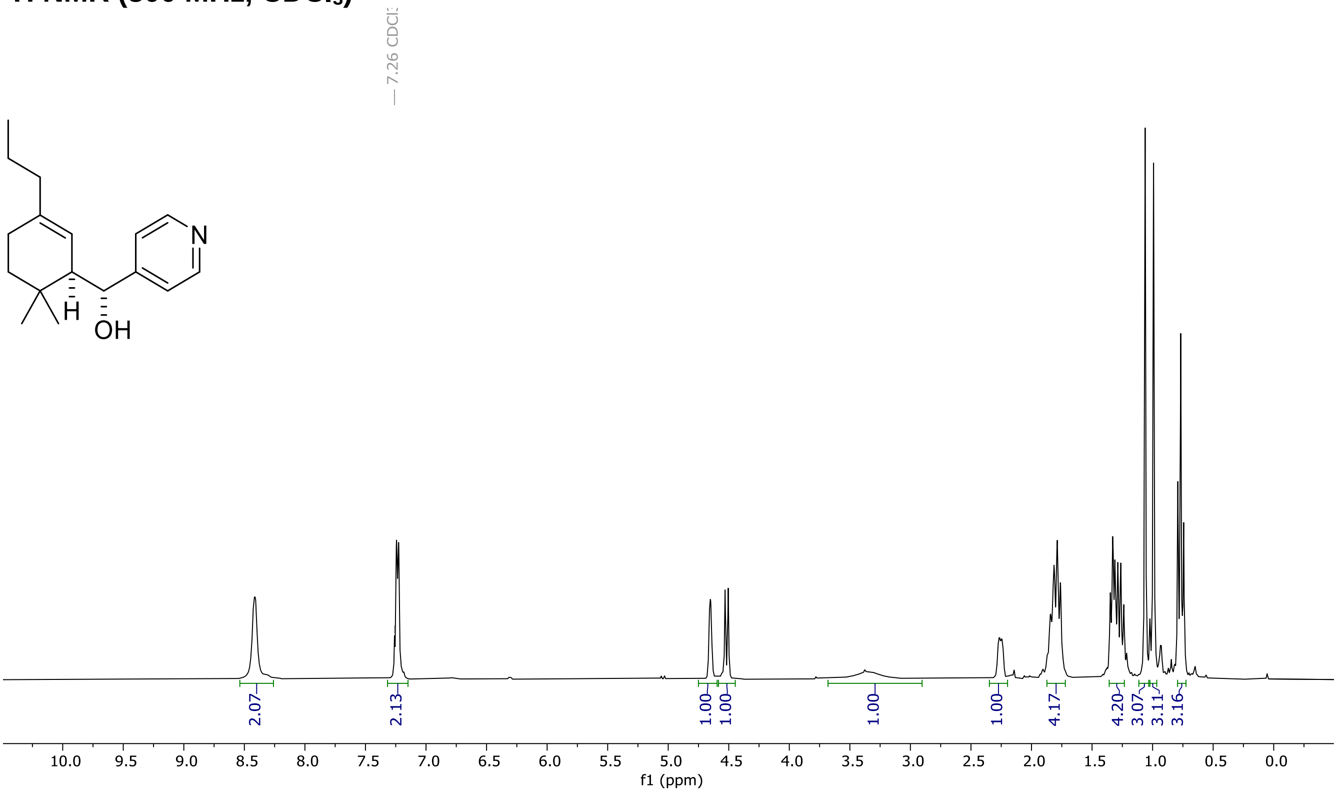

^{13}C NMR (75 MHz, CDCl$_3$)

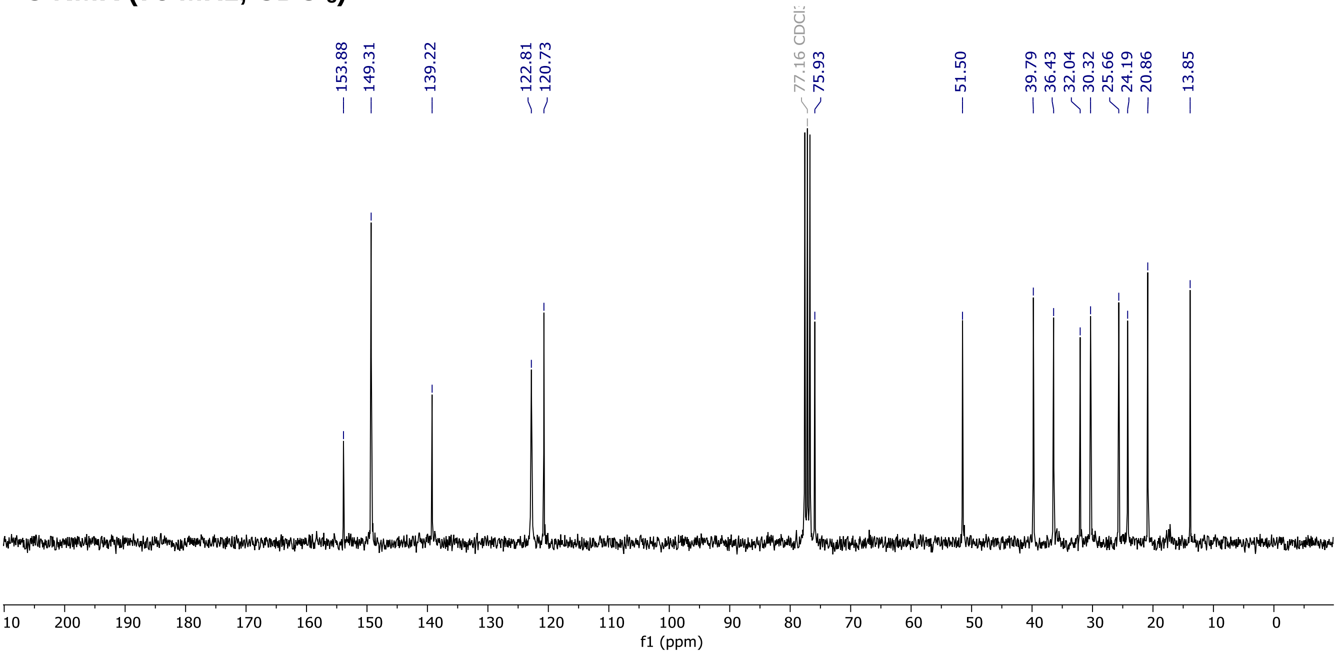

(R^*)-1-((R^*)-6,6-dimethyl-3-propylcyclohex-2-en-1-yl)-2-methylprop-2-en-1-ol (5l)

^{1}H NMR (300 MHz, CDCl$_3$)

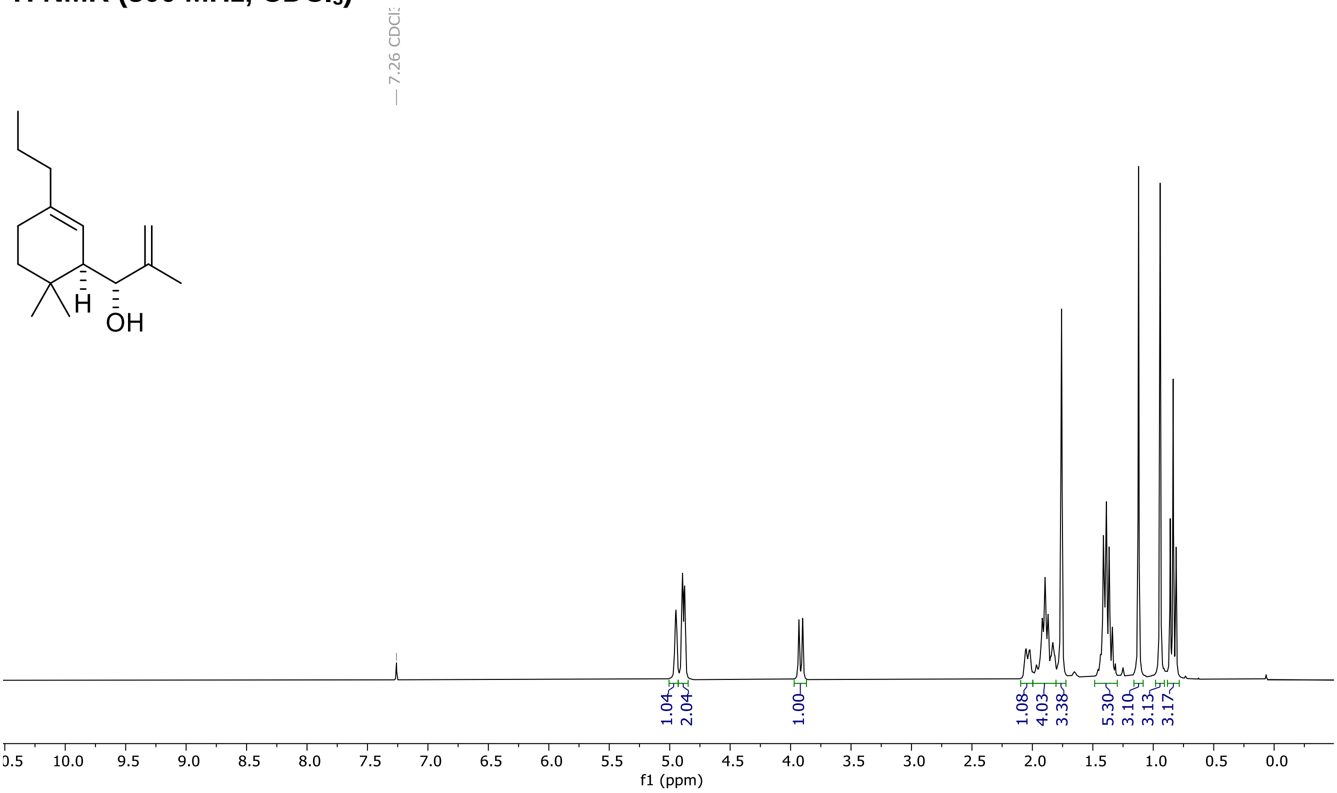

^{13}C NMR (75 MHz, CDCl$_3$)

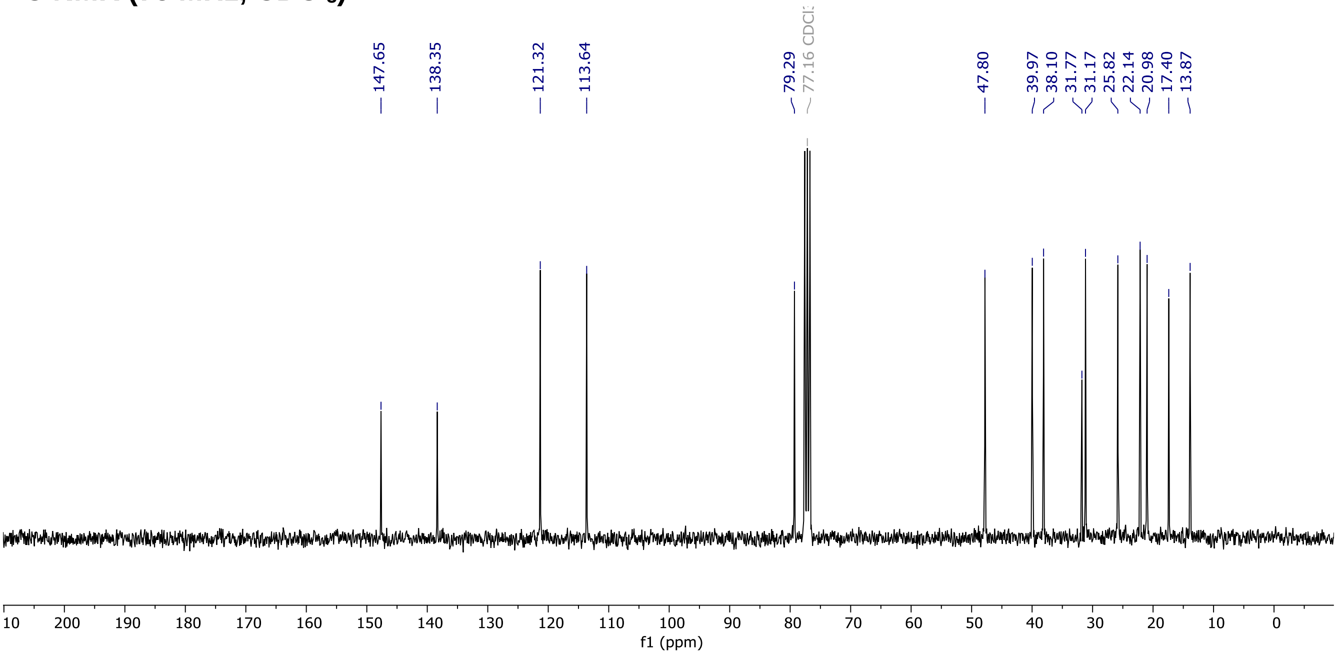

(*S**,*E*)-1-((*R**)-6,6-dimethyl-3-propylcyclohex-2-en-1-yl)-3-phenylprop-2-en-1-ol (5m)

Major isomer

^{1}H NMR (300 MHz, $CDCl_3$)

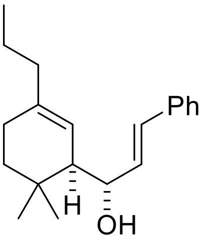

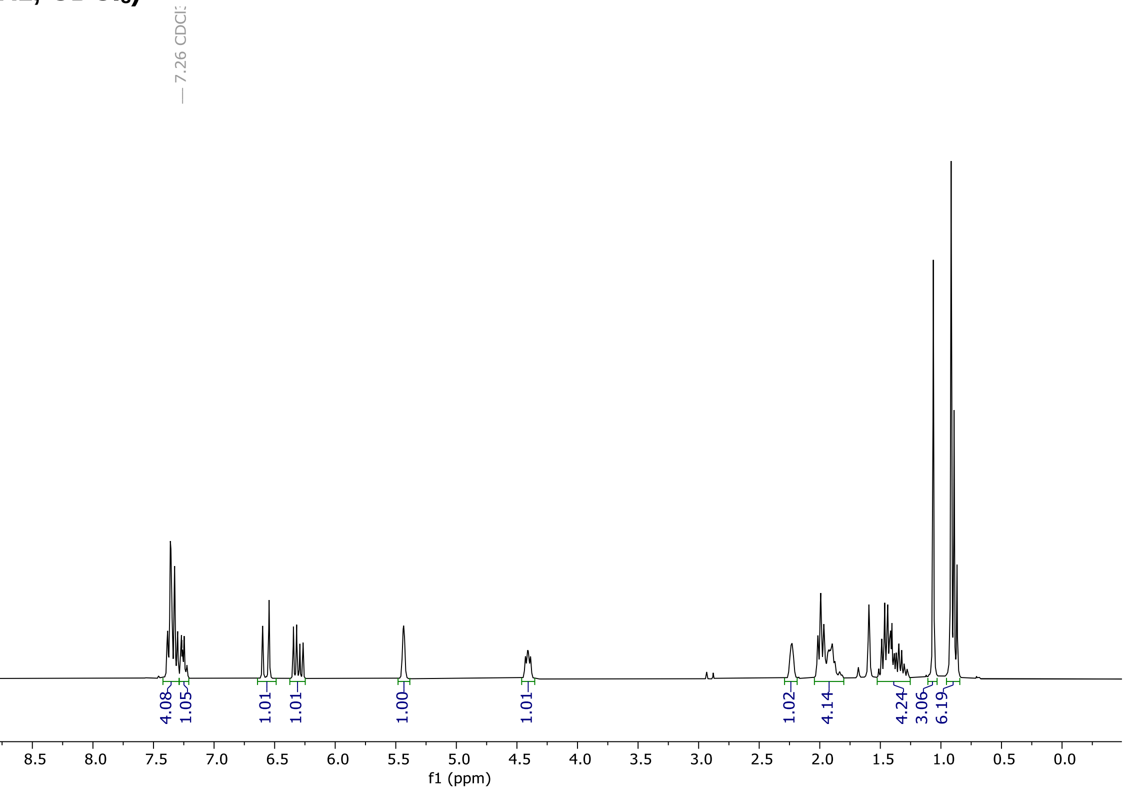

^{13}C NMR (75 MHz, $CDCl_3$)

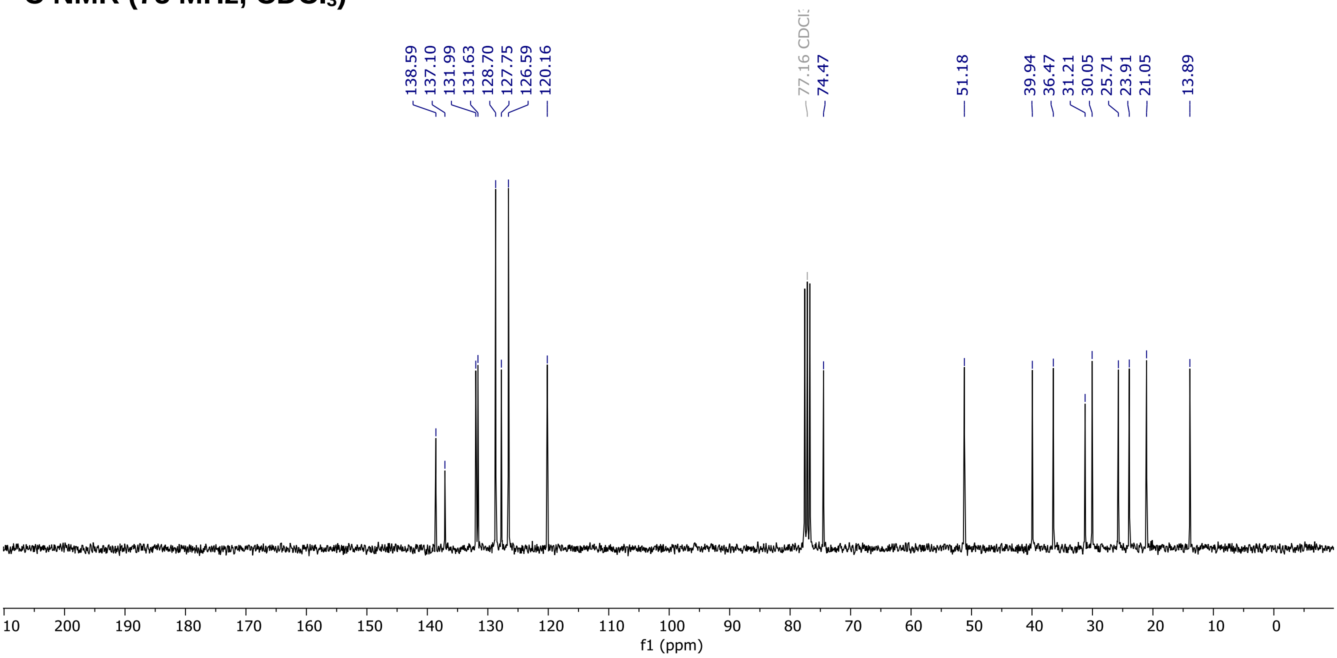

(*S,*E*)-1-((*R**)-6,6-dimethyl-3-propylcyclohex-2-en-1-yl)-3-phenylprop-2-en-1-ol (5m)**

Minor isomer

^{1}H NMR (300 MHz, CDCl$_3$)

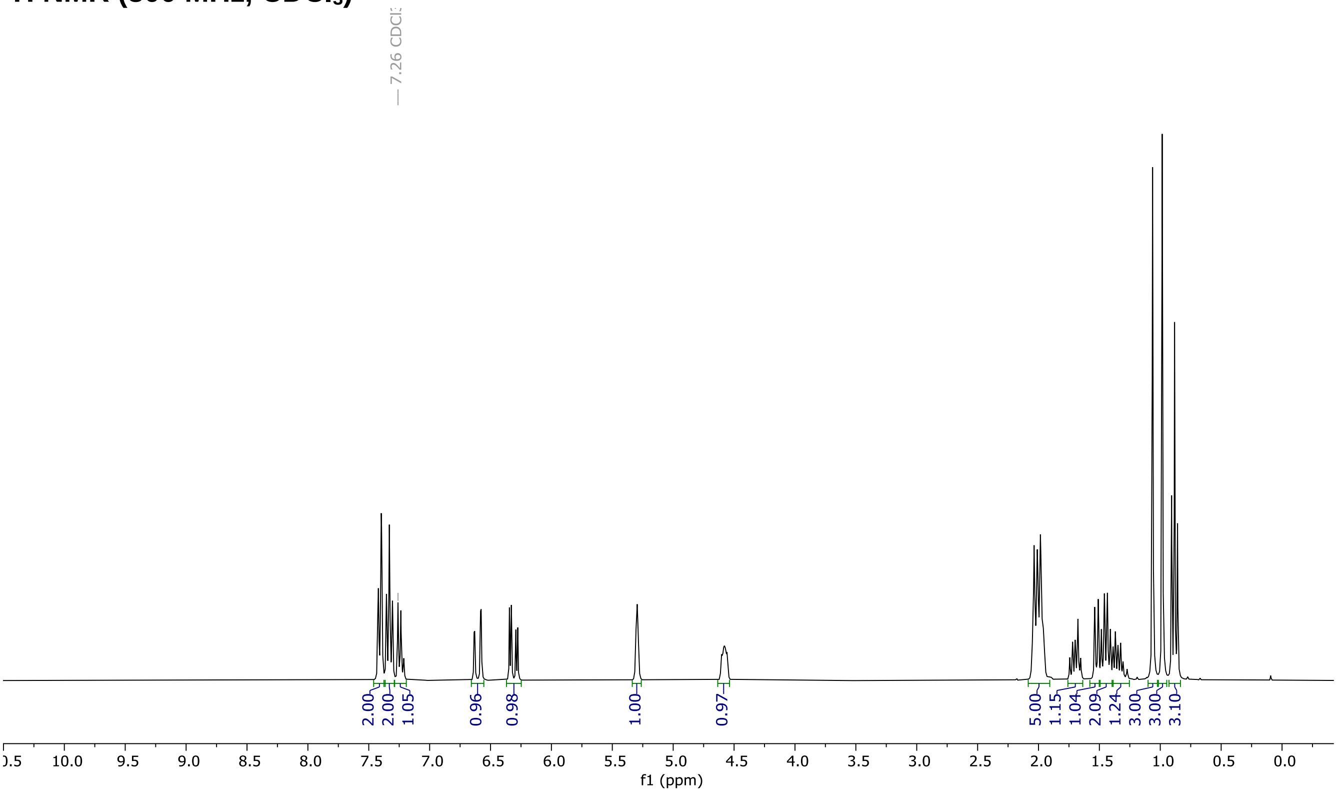

^{13}C NMR (75 MHz, CDCl$_3$)

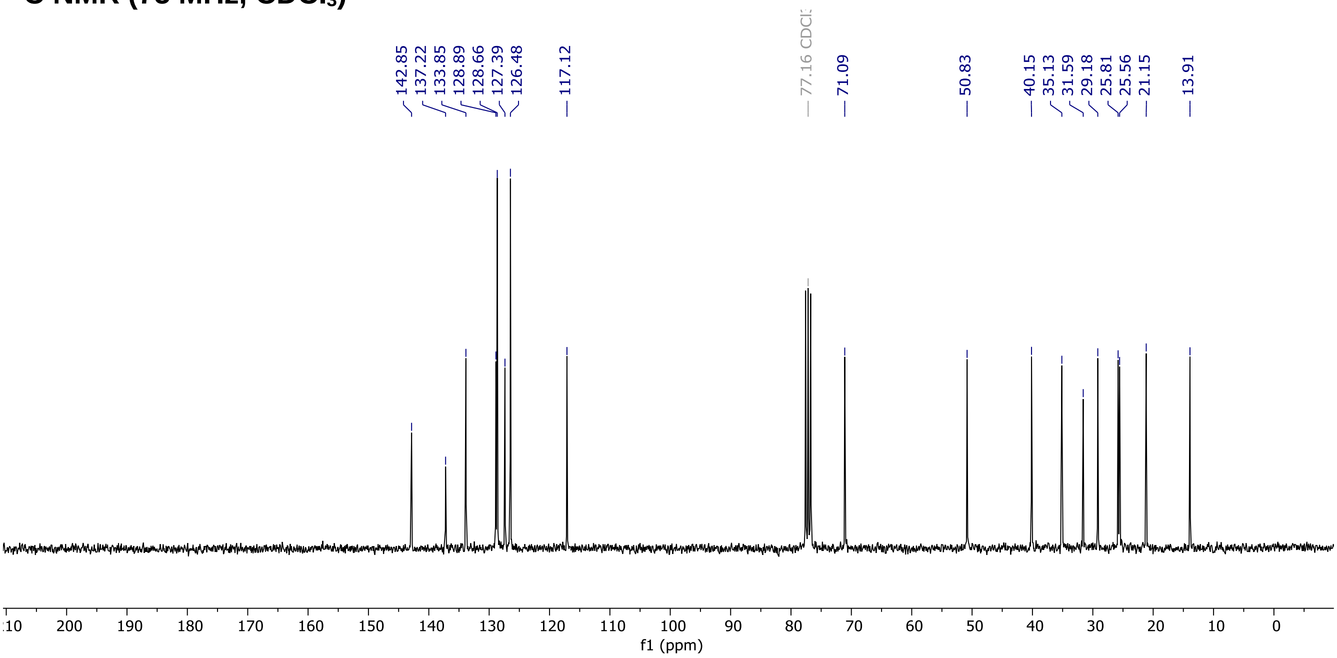

(*R**)-(2-bromophenyl)((*R**)-3,6,6-trimethylcyclohex-2-en-1-yl)methanol (5n)

^{1}H NMR (300 MHz, $CDCl_3$)

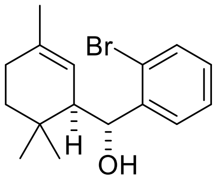

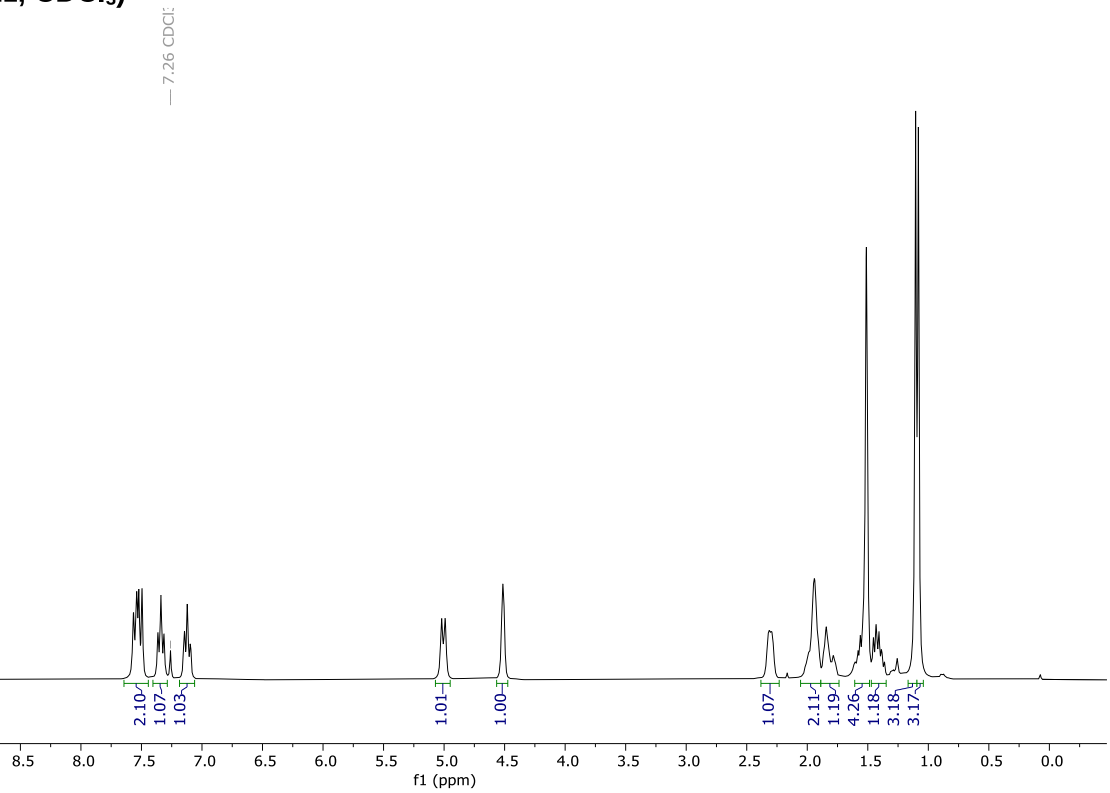

^{13}C NMR (75 MHz, $CDCl_3$)

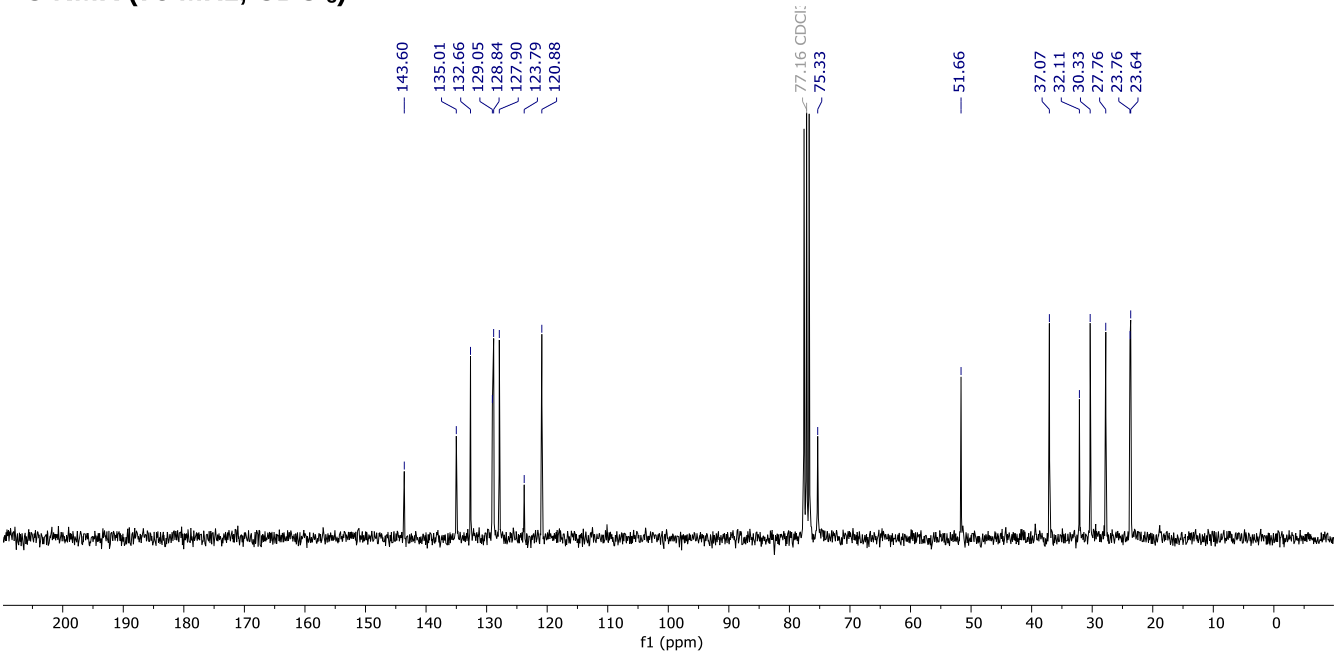

Ethyl 4-((*R**)-3-((*R**)-hydroxy(p-tolyl)methyl)-4,4-dimethylcyclohex-1-en-1-yl)butanoate (5o)

^{1}H NMR (300 MHz, $CDCl_3$)

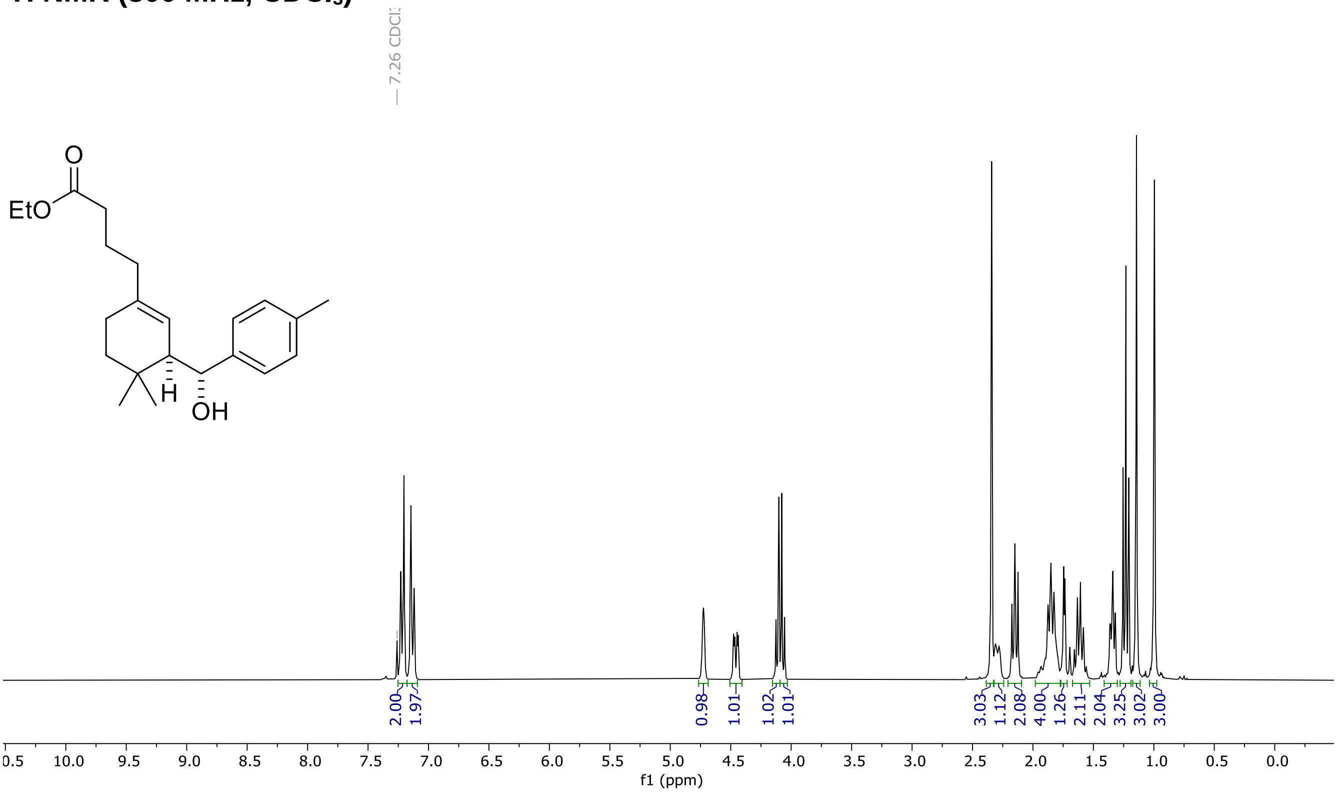

^{13}C NMR (75 MHz, $CDCl_3$)

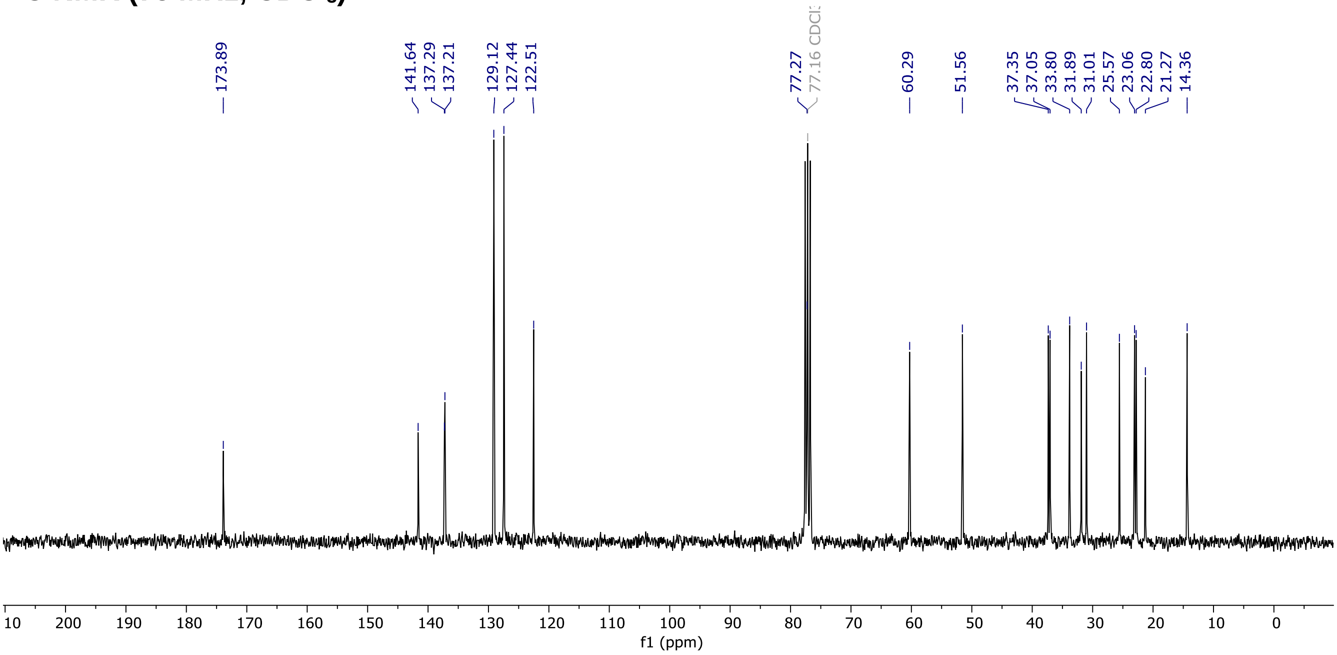

(*R)-((*R**)-3-cyclopentyl-6,6-dimethylcyclohex-2-en-1-yl)(p-tolyl)methanol (5p)**

^{1}H NMR (300 MHz, CDCl$_3$)

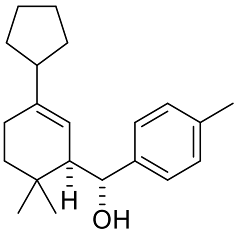

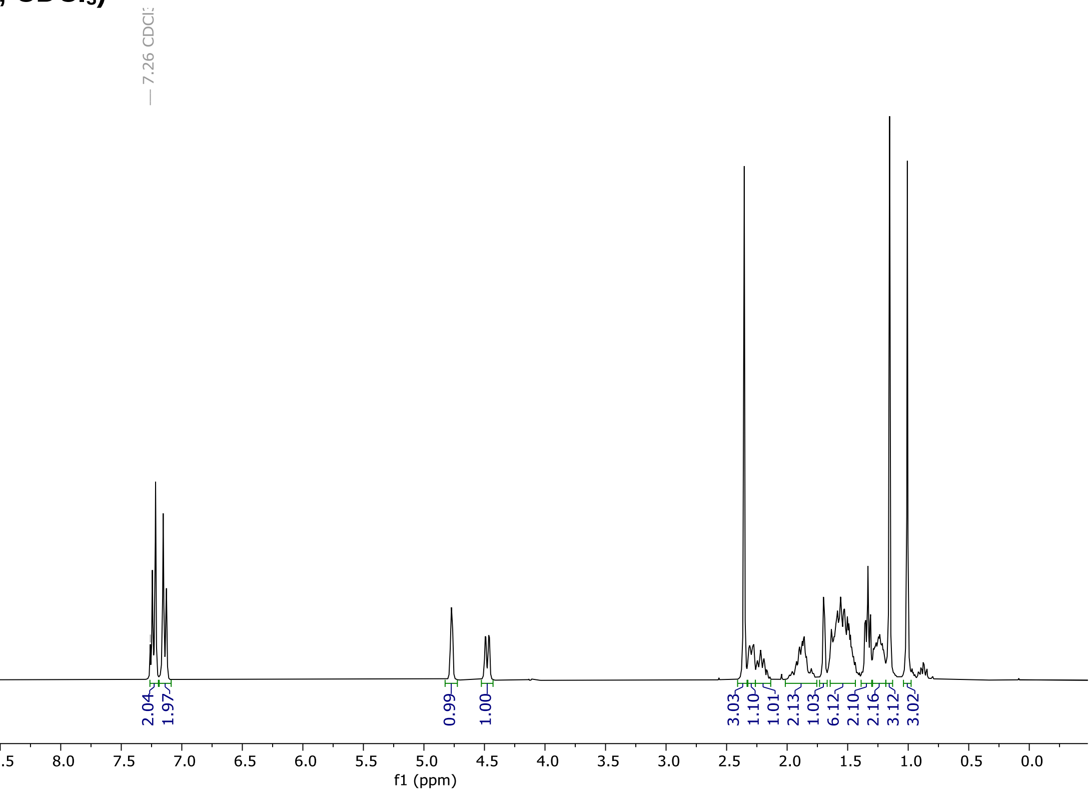

^{13}C NMR (75 MHz, CDCl$_3$)

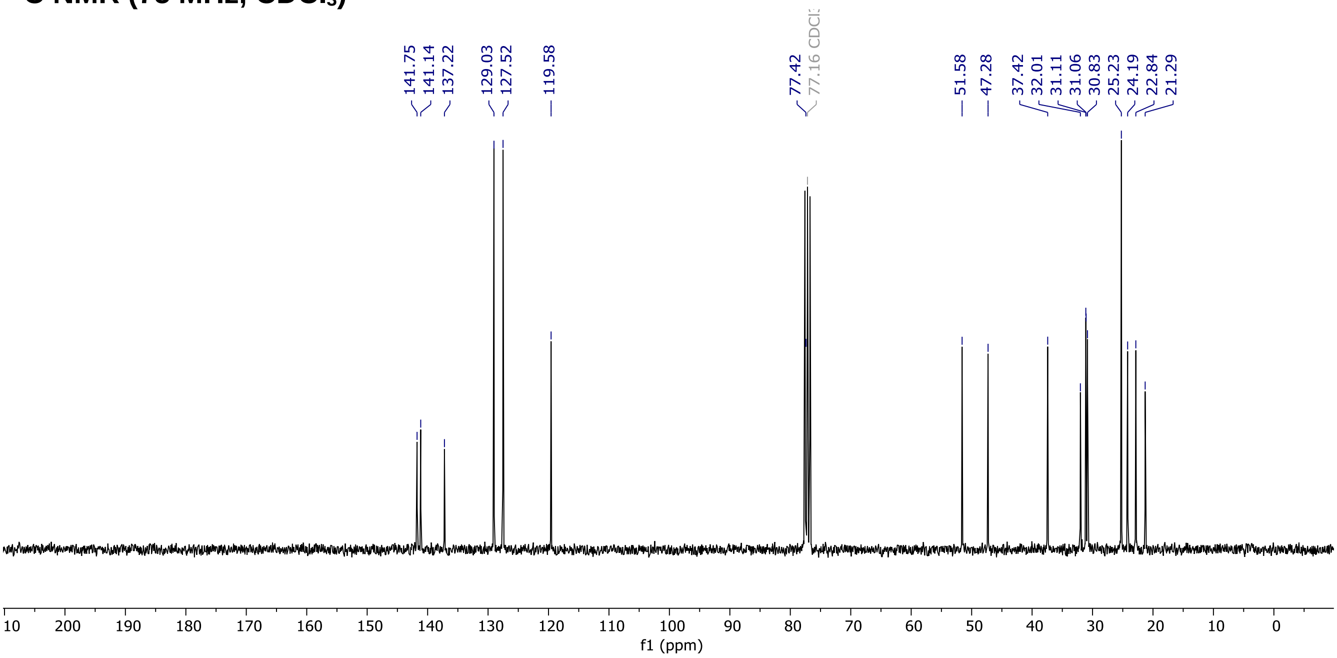

(R*)-(1H-indol-7-yl)((R*)-3,6,6-trimethylcyclohex-2-en-1-yl)methanol (5q)

^{1}H NMR (300 MHz, $CDCl_3$)

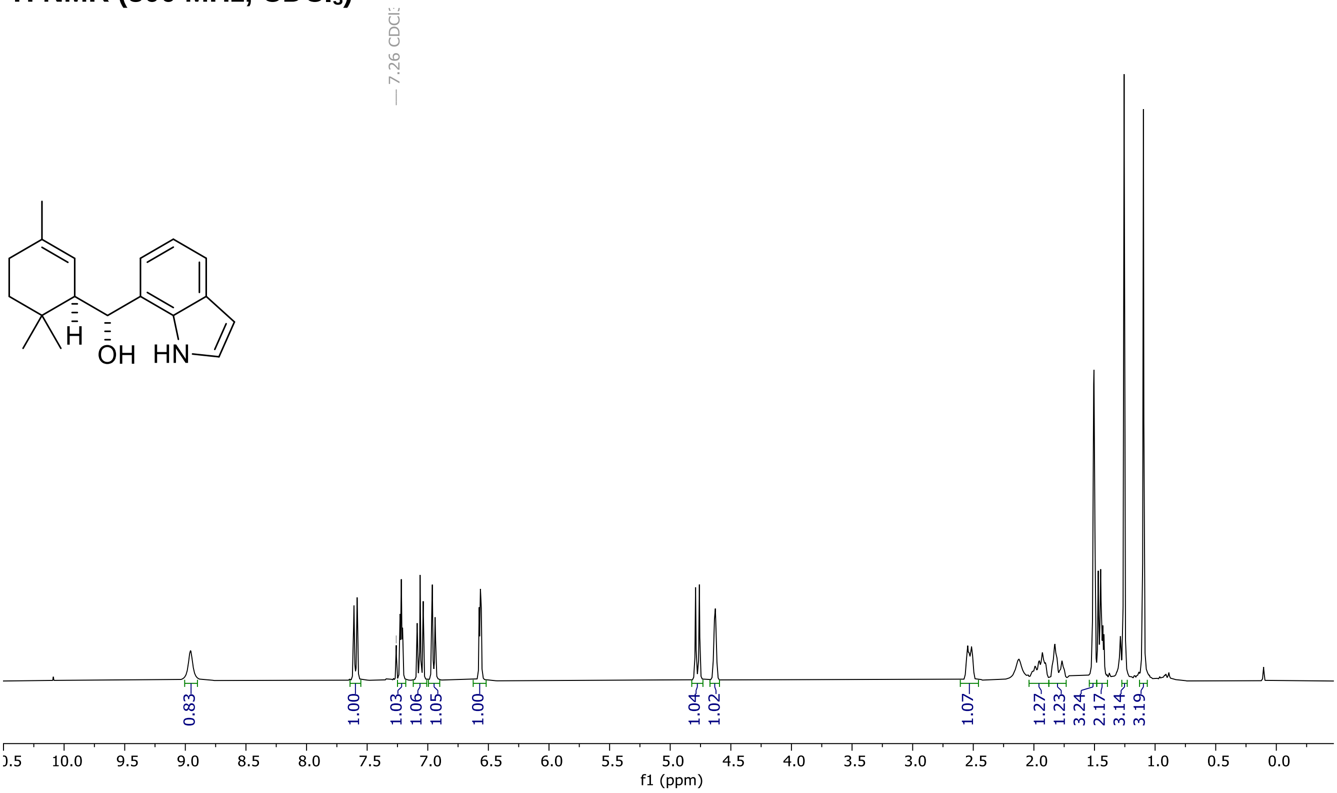

^{13}C NMR (75 MHz, $CDCl_3$)

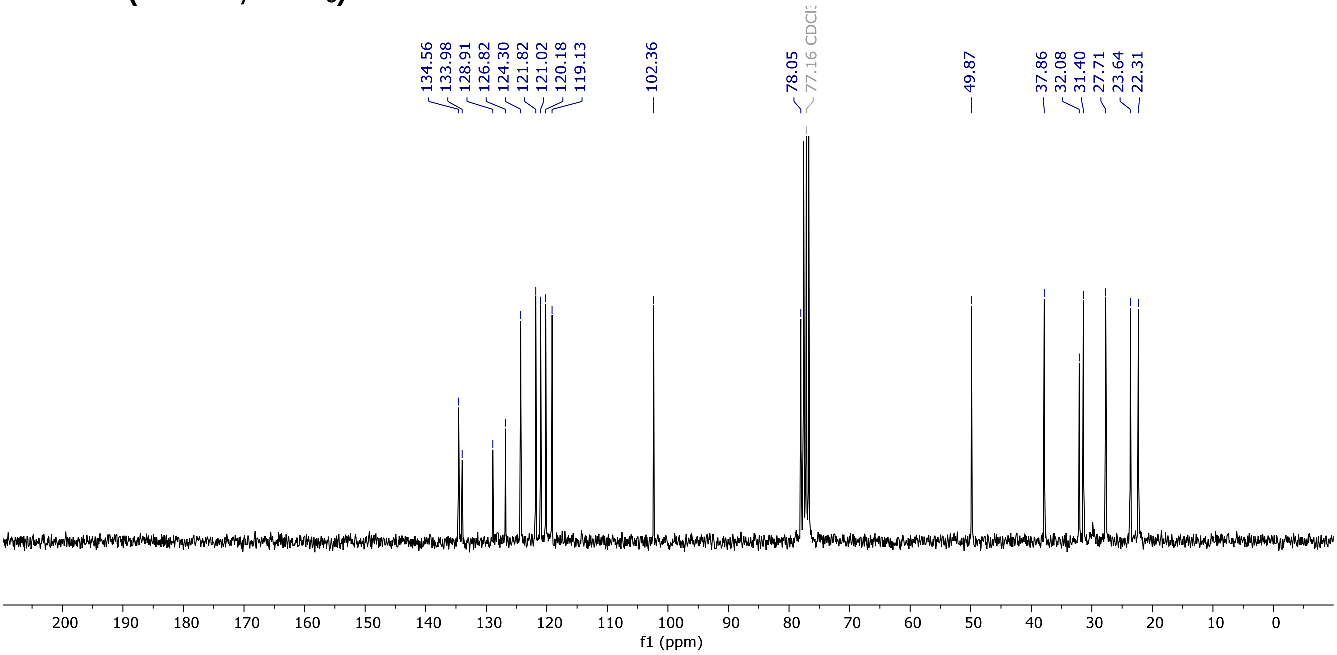

(*R**)-(2-Bromophenyl)-(*R**)-(6,6-dimethyl-3-phenethylcyclohex-2-en-1-yl)methanol (5r)

^{1}H NMR (300 MHz, $CDCl_3$)

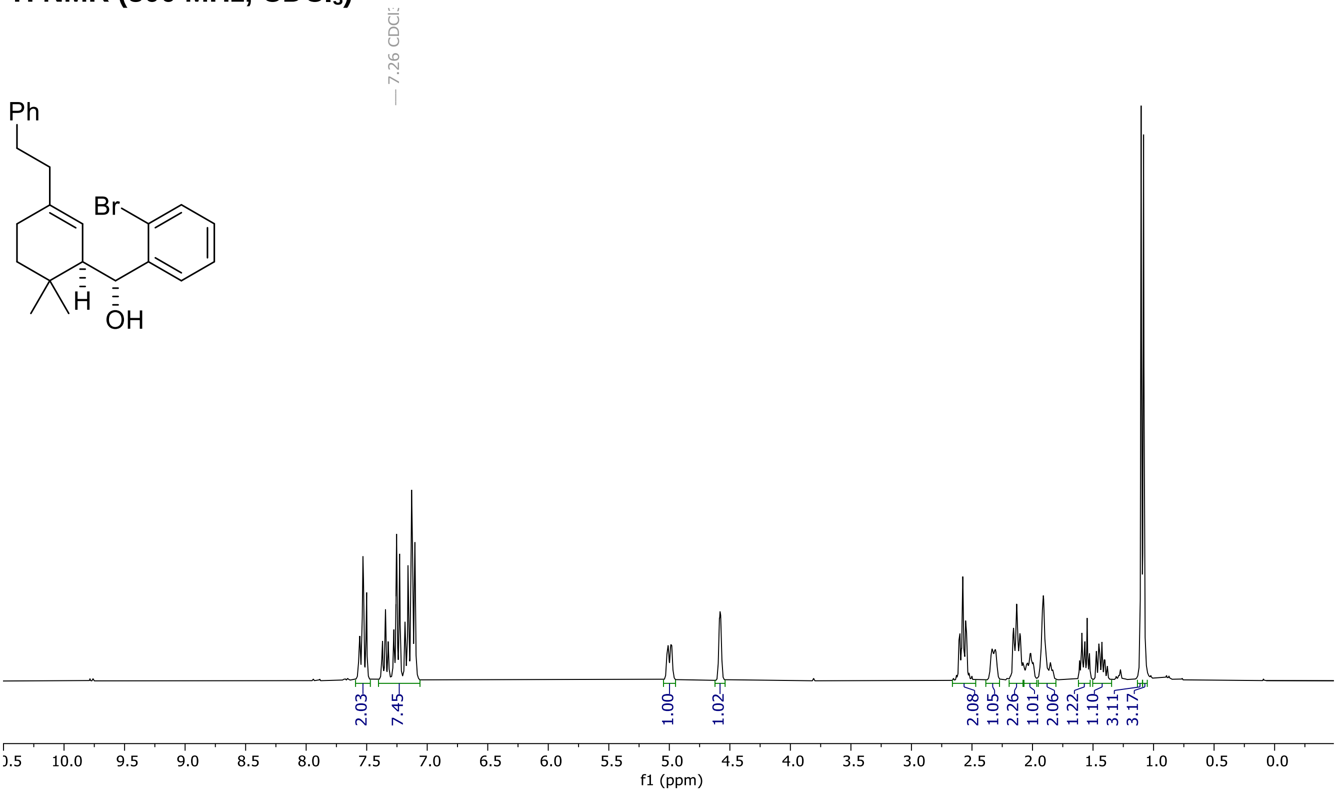

^{13}C NMR (75 MHz, $CDCl_3$)

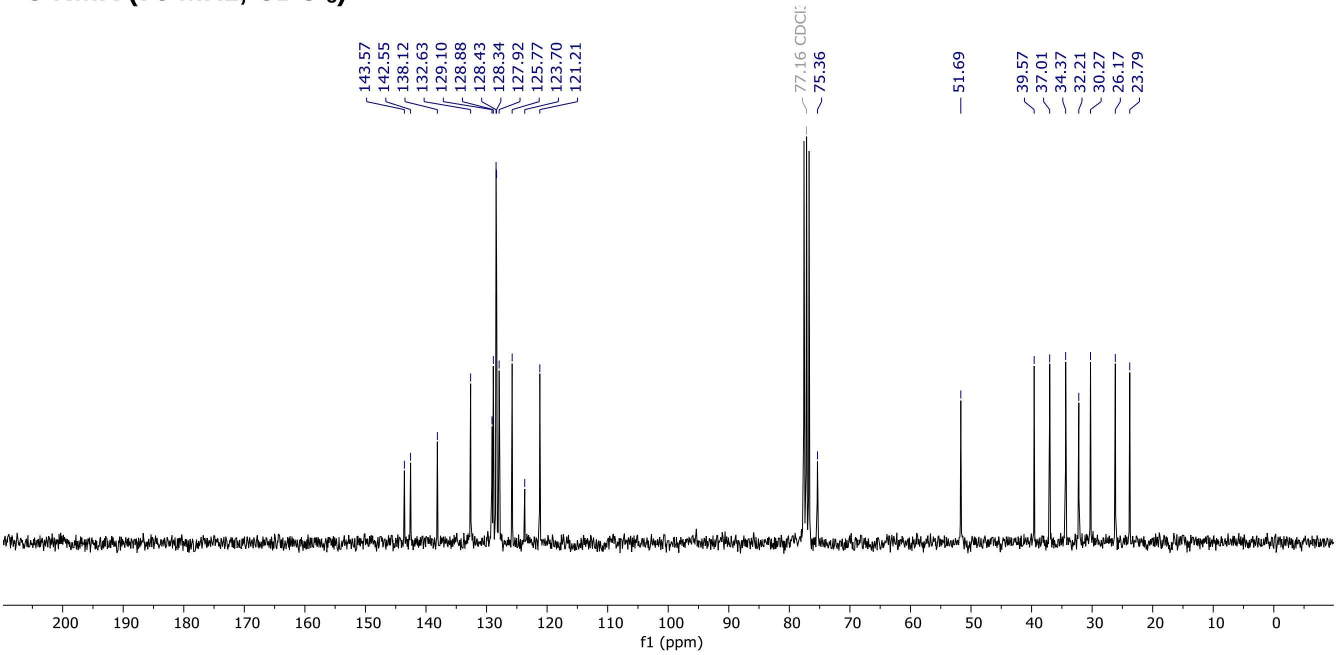

(R)-((R)-6,6-dimethyl-3-propylcyclohex-2-en-1-yl)(4-nitrophenyl)methyl 4-bromobenzoate (6)

^{1}H NMR (300 MHz, CDCl$_3$)

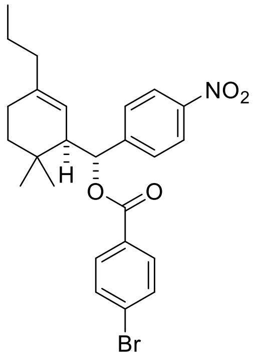

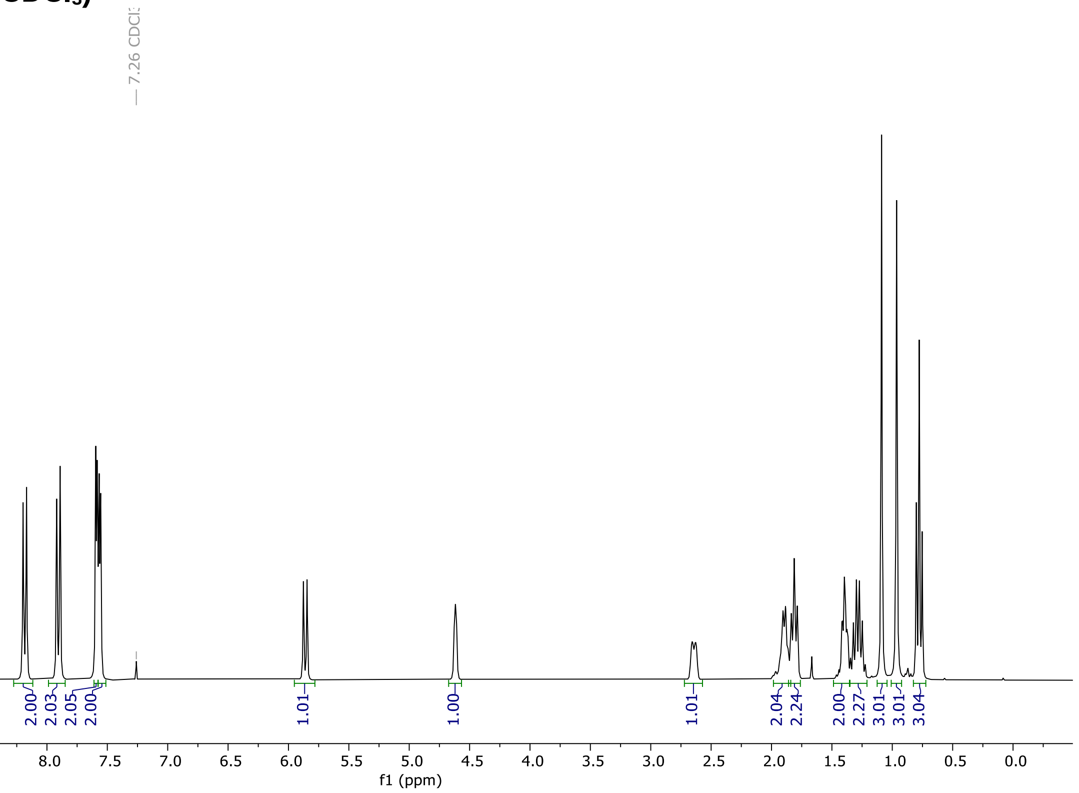

^{13}C NMR (75 MHz, CDCl$_3$)

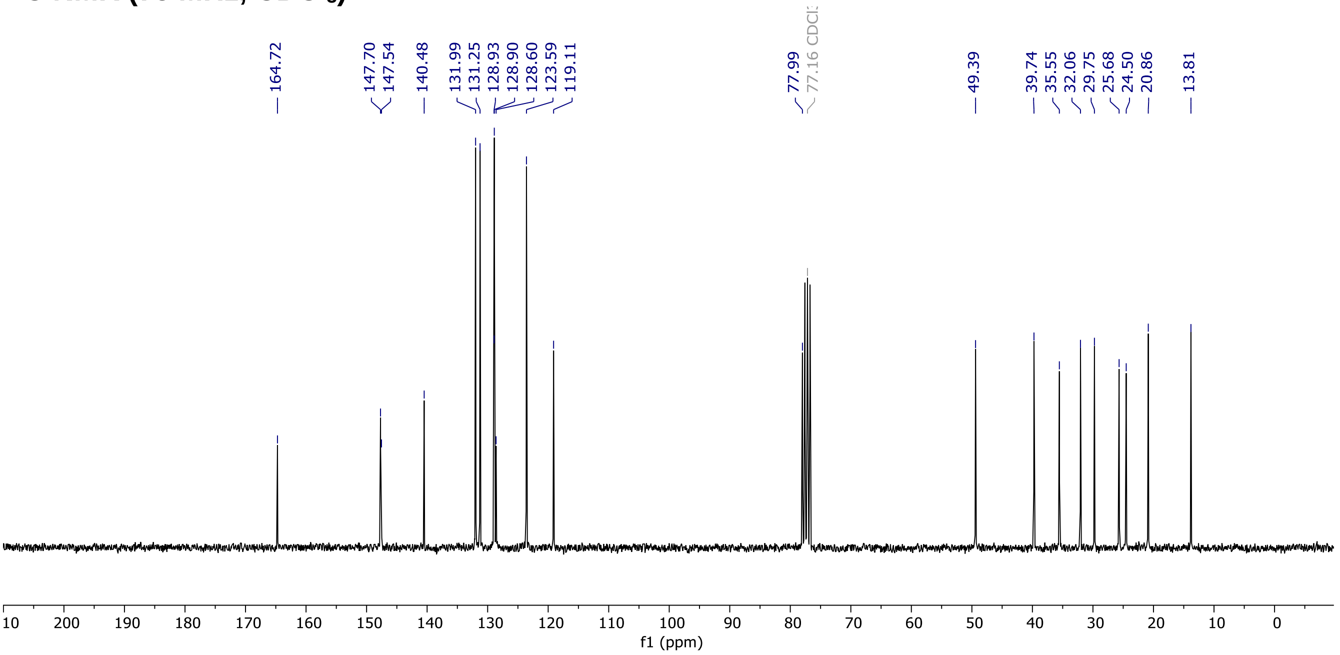

4-((*R)-((*S**)-1,3-dimethylcyclohex-2-en-1-yl)(hydroxy)methyl)benzonitrile (7a)**

^{1}H NMR (300 MHz, $CDCl_3$)

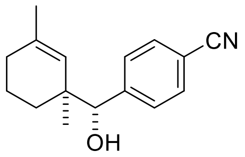

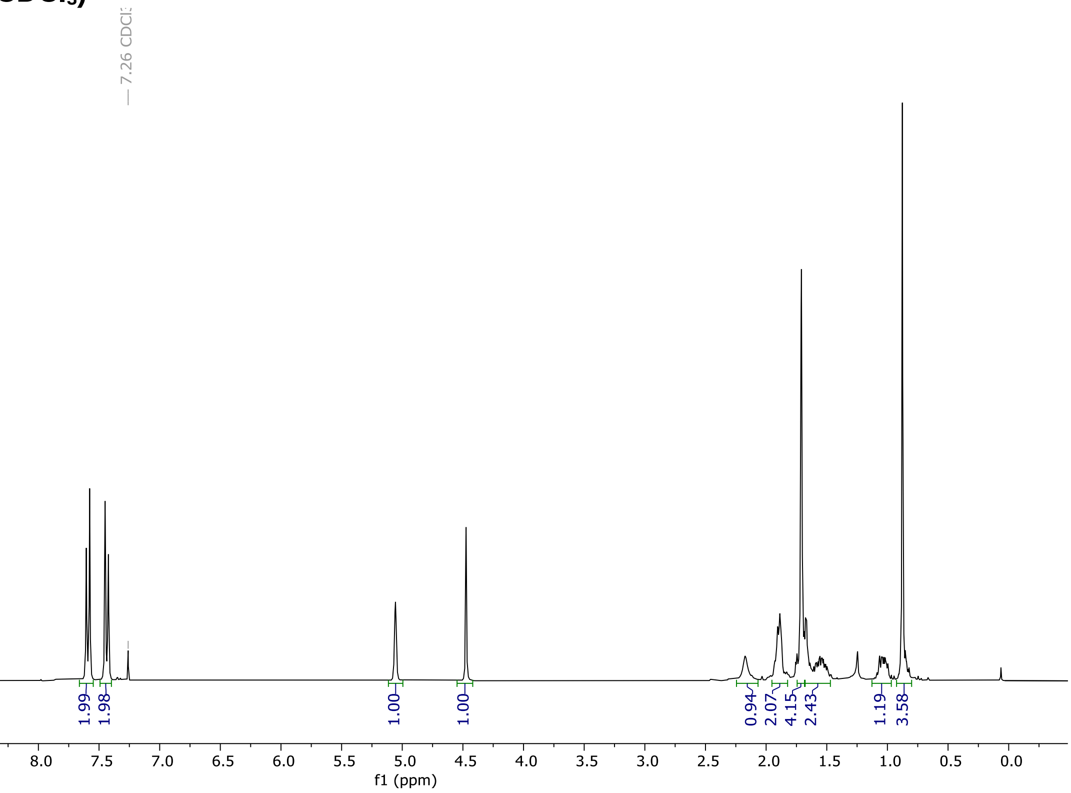

^{13}C NMR (75 MHz, $CDCl_3$)

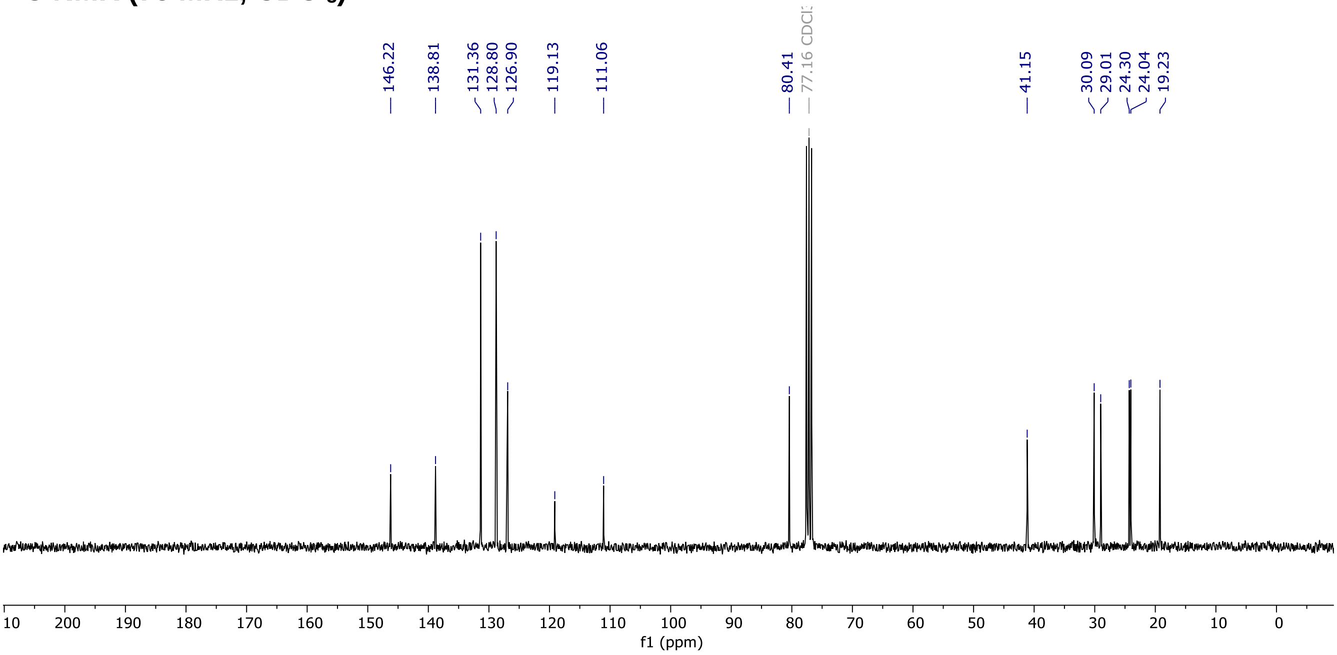

4-((*R)-((*S**)-3-(4-cyanobutyl)-1-methylcyclohex-2-en-1-yl)(hydroxy)methyl)benzonitrile (7b)**

^{1}H NMR (300 MHz, CDCl$_3$)

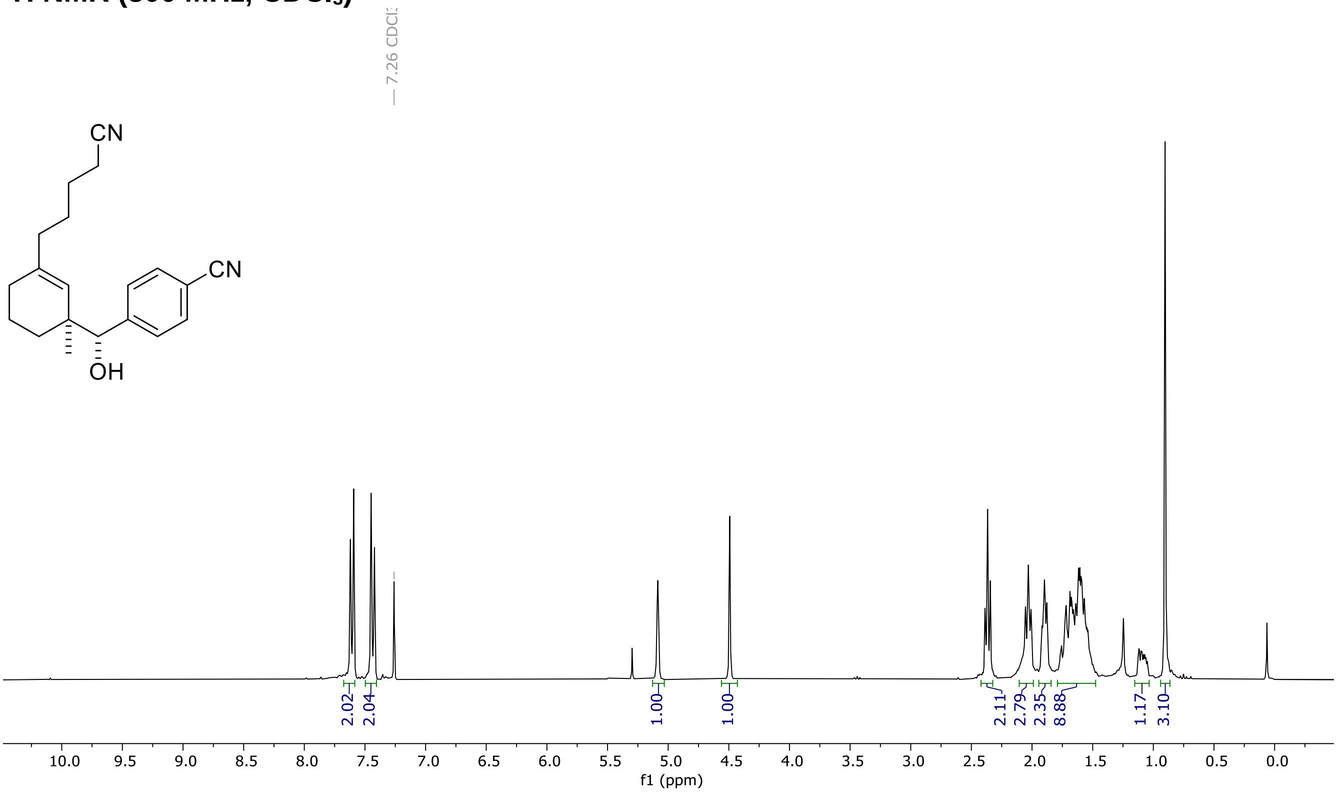

^{13}C NMR (75 MHz, CDCl$_3$)

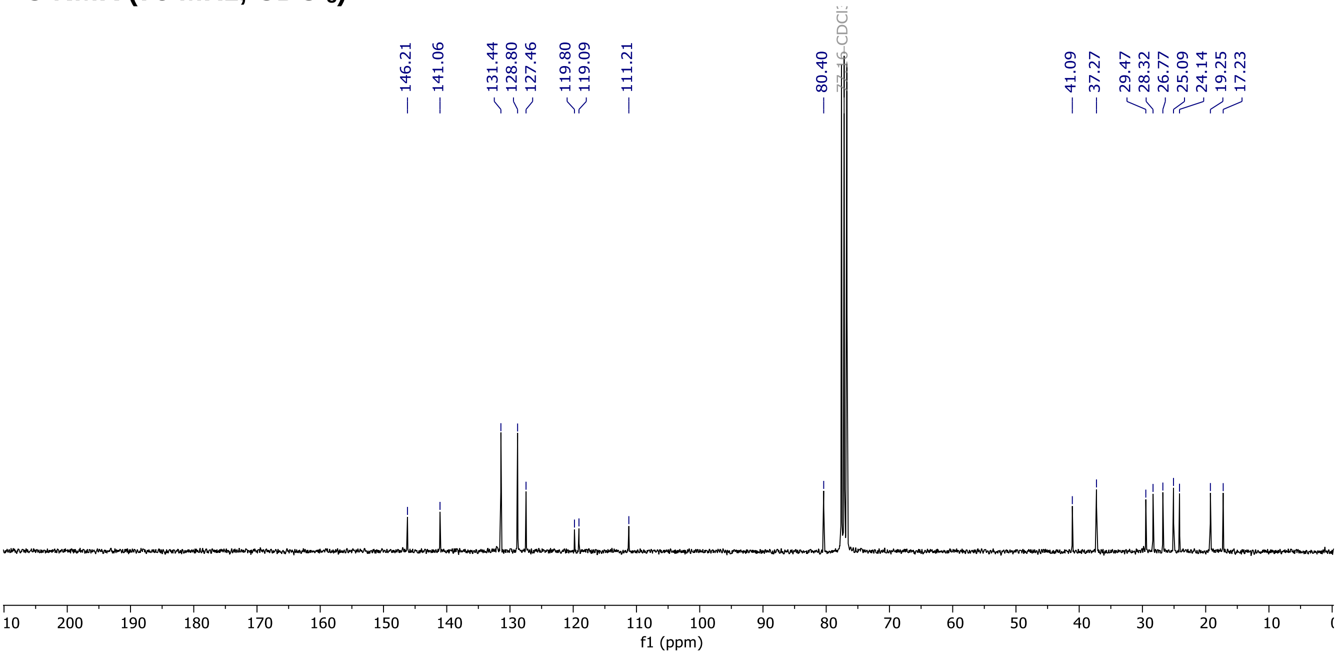

(*S**)-3-methyl-1-((*S**)-1-methyl-3-propylcyclohex-2-en-1-yl)but-2-en-1-ol (7c)

^{1}H NMR (300 MHz, $CDCl_3$)

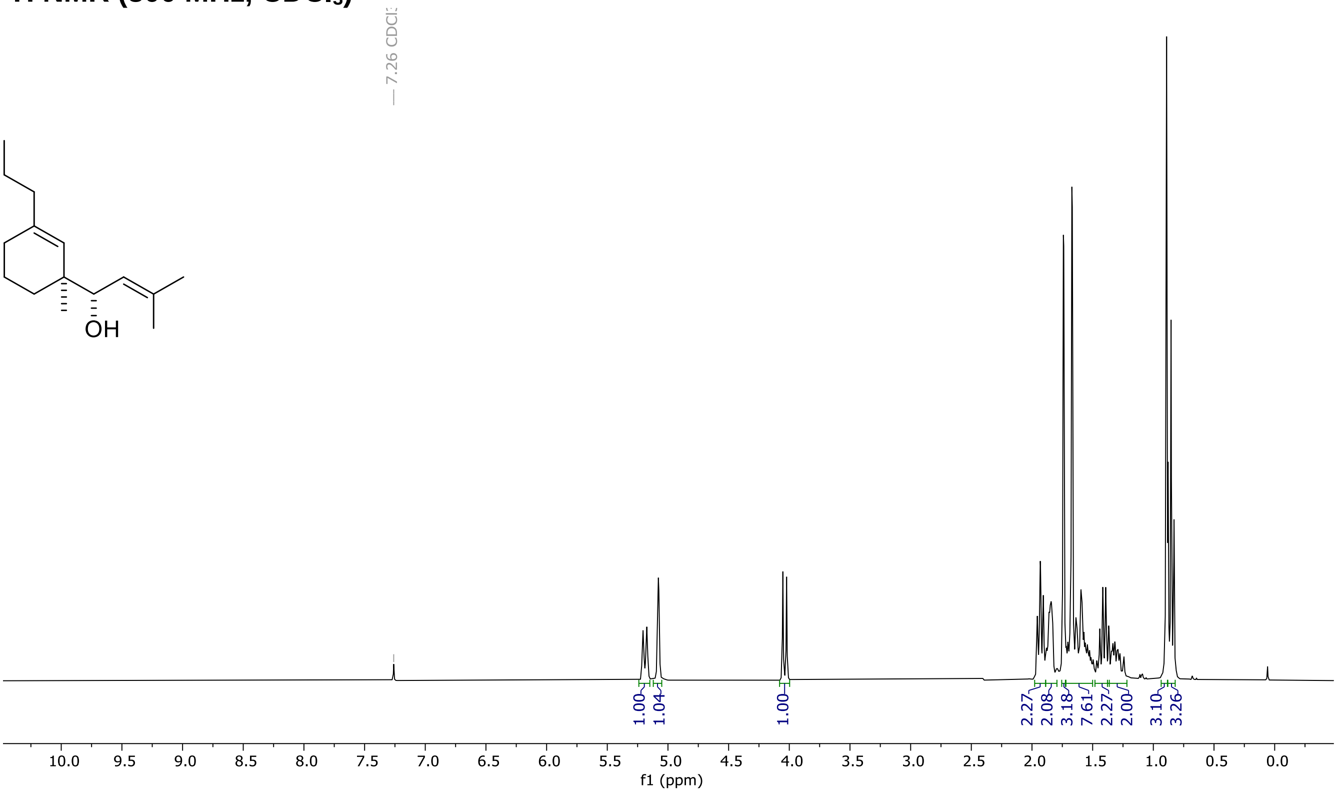

^{13}C NMR (75 MHz, $CDCl_3$)

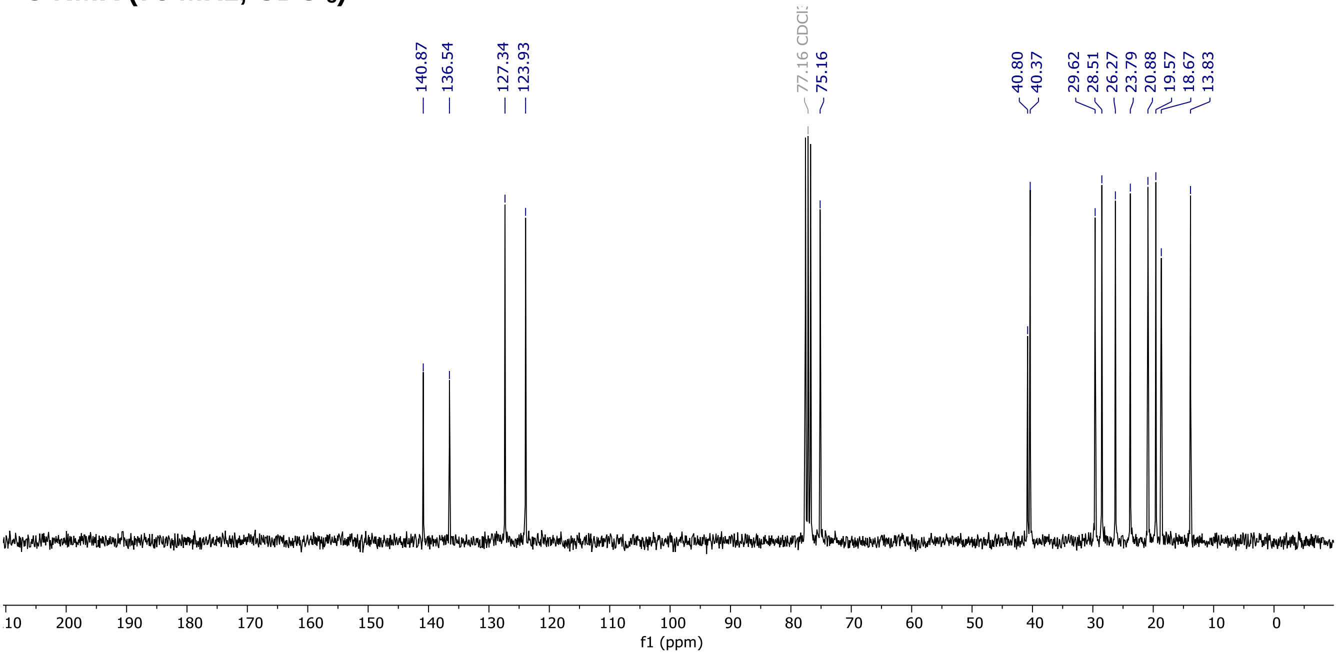

(*R**)-((*S**)-1-methyl-3-phenethylcyclopent-2-en-1-yl)(p-tolyl)methanol (8a)

^{1}H NMR (300 MHz, $CDCl_3$)

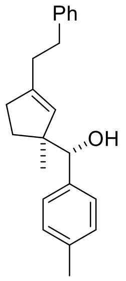

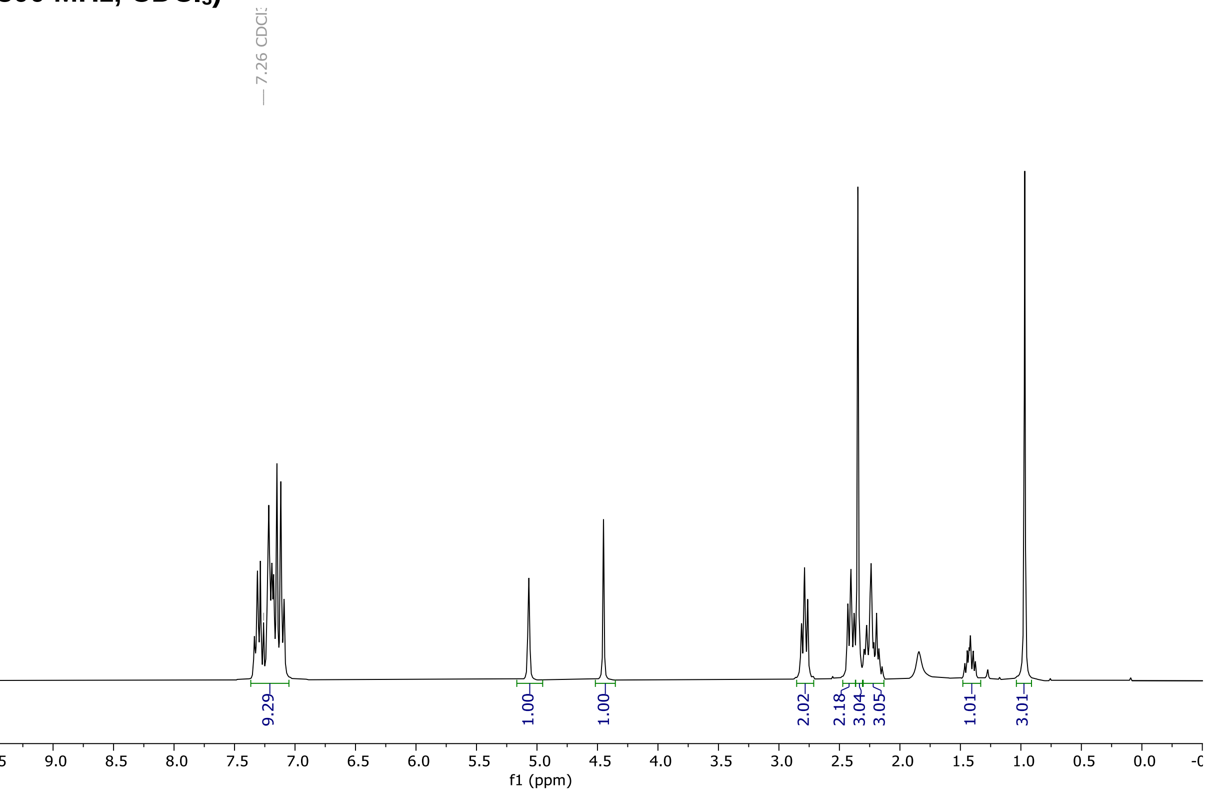

^{13}C NMR (75 MHz, $CDCl_3$)

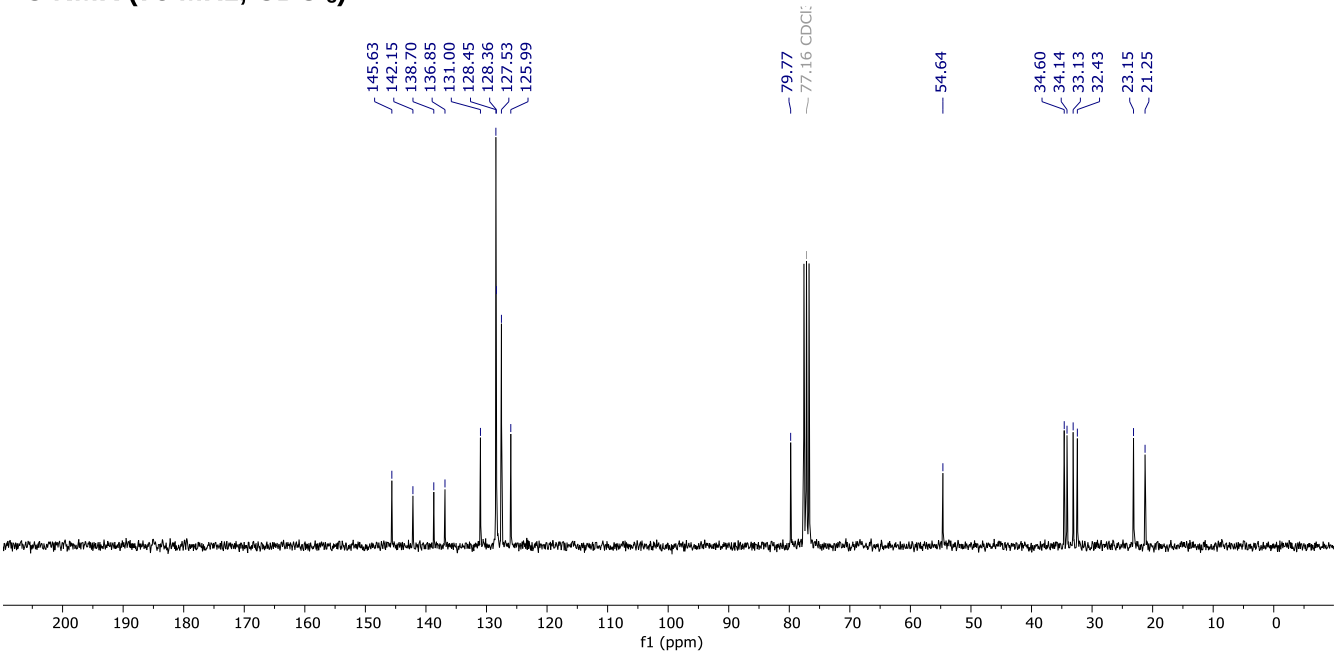

(*R)-((*S**)-3-(but-3-en-1-yl)-1-methylcyclopent-2-en-1-yl)(p-tolyl)methanol (8b)**

^{1}H NMR (300 MHz, $CDCl_3$)

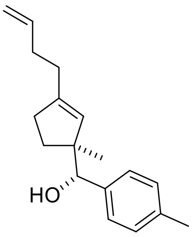

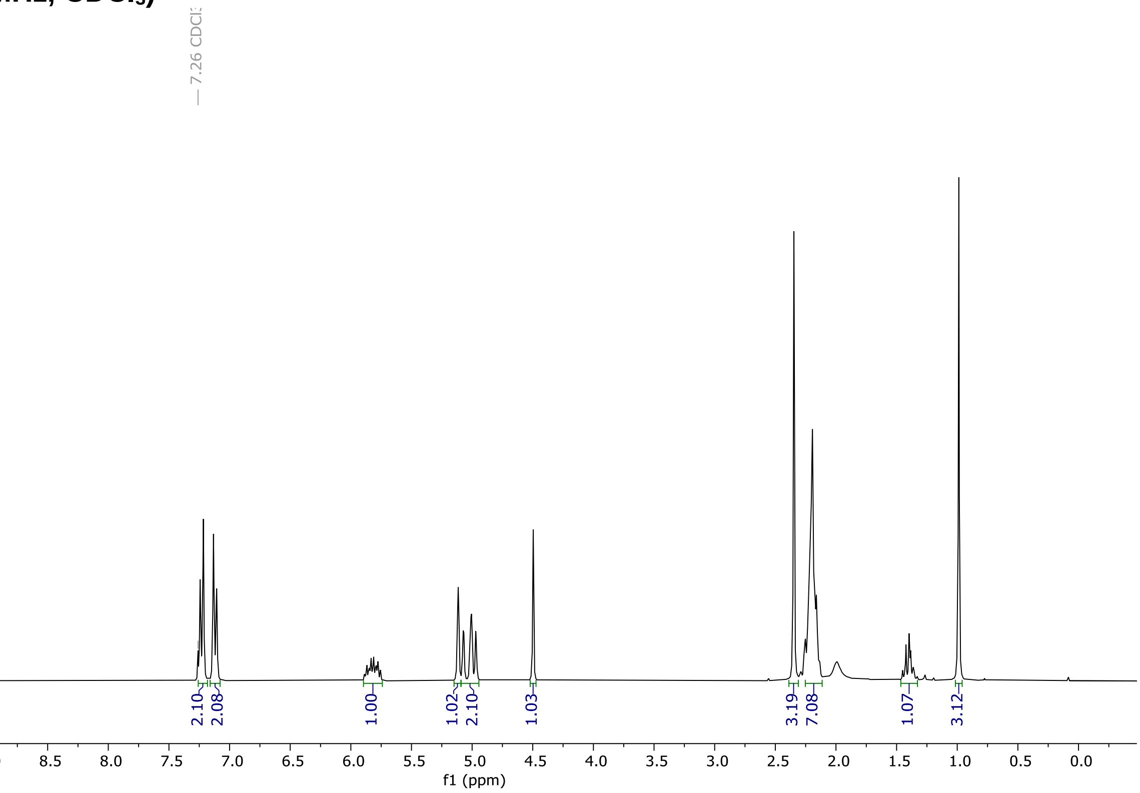

^{13}C NMR (75 MHz, $CDCl_3$)

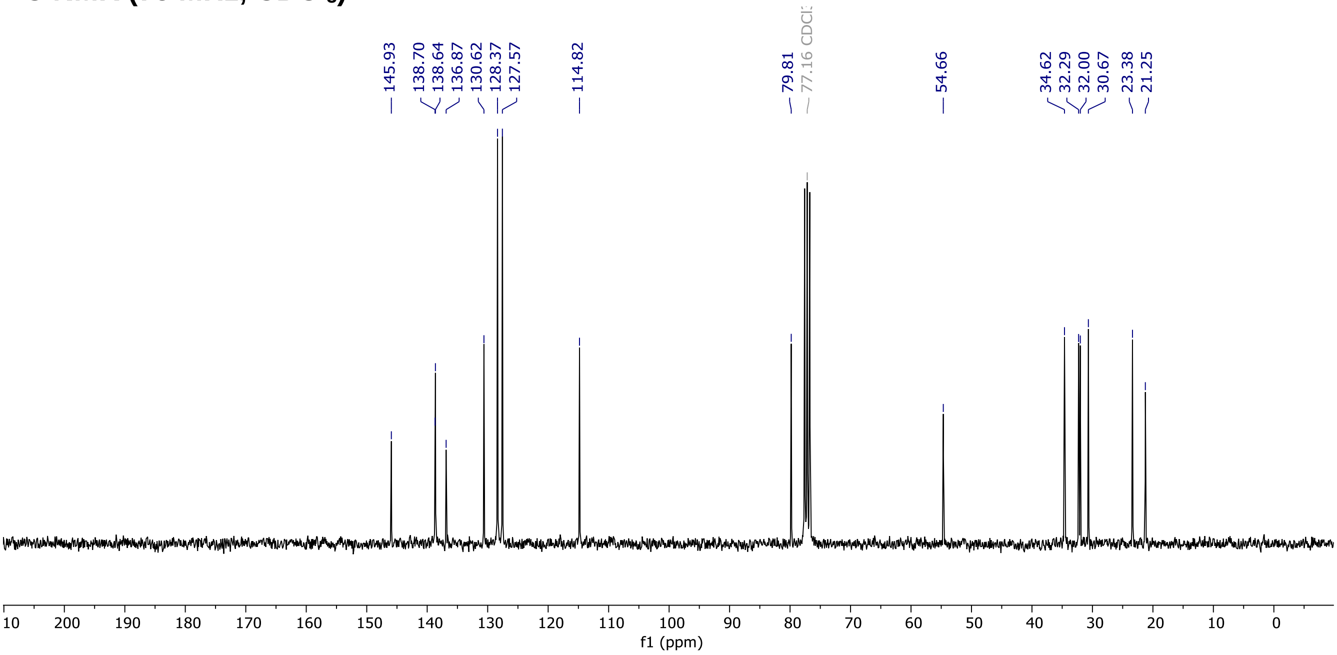

(*S,*E*)-1-((*S**)-3-cyclopropyl-1-methylcyclopent-2-en-1-yl)-3-phenylprop-2-en-1-ol (8c)**

^{1}H NMR (300 MHz, CDCl$_3$)

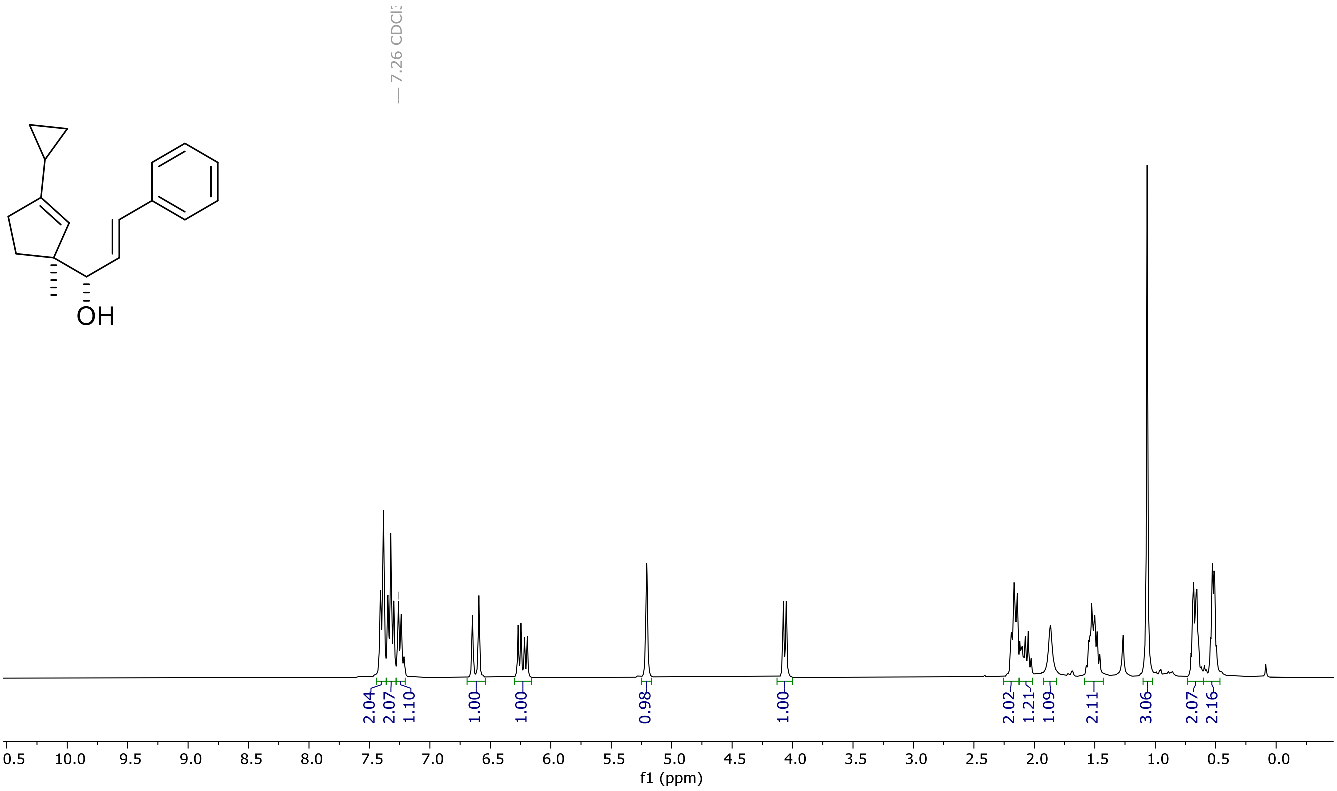

^{13}C NMR (75 MHz, CDCl$_3$)

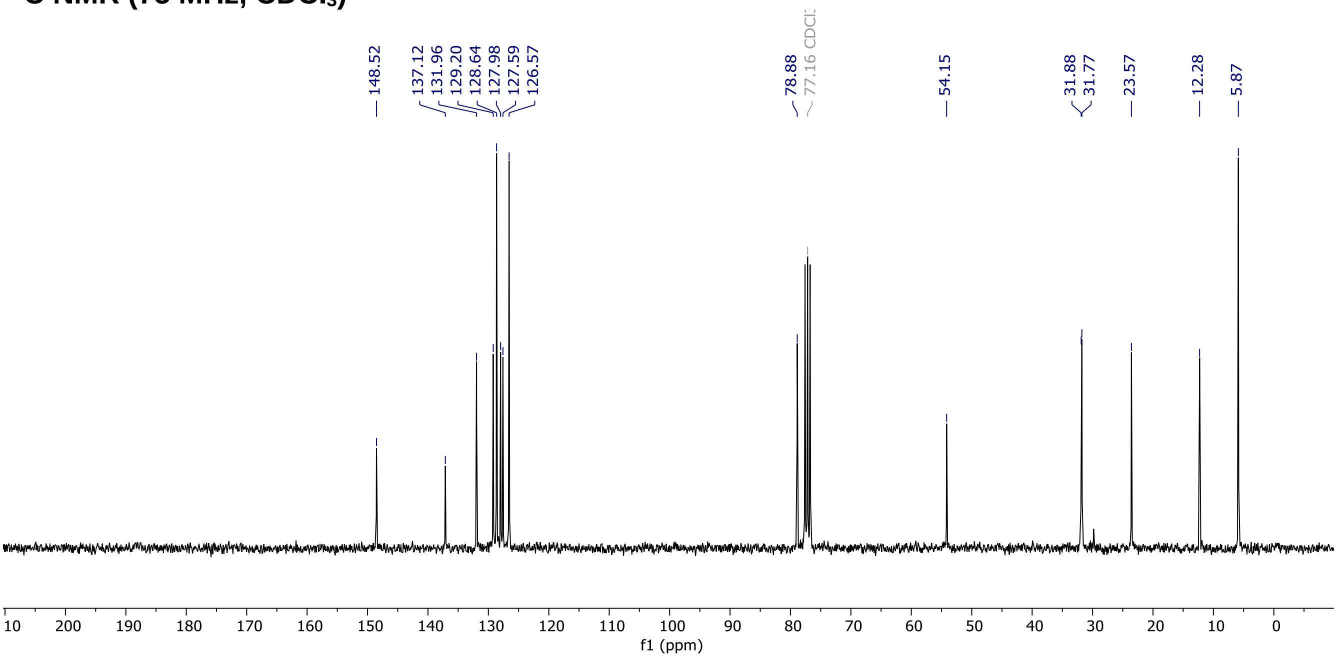

(*S**,*E*)-1-((*S**)-1-methyl-3-propylcyclopent-2-en-1-yl)-3-phenylprop-2-en-1-ol (8d)

^{1}H NMR (300 MHz, $CDCl_3$)

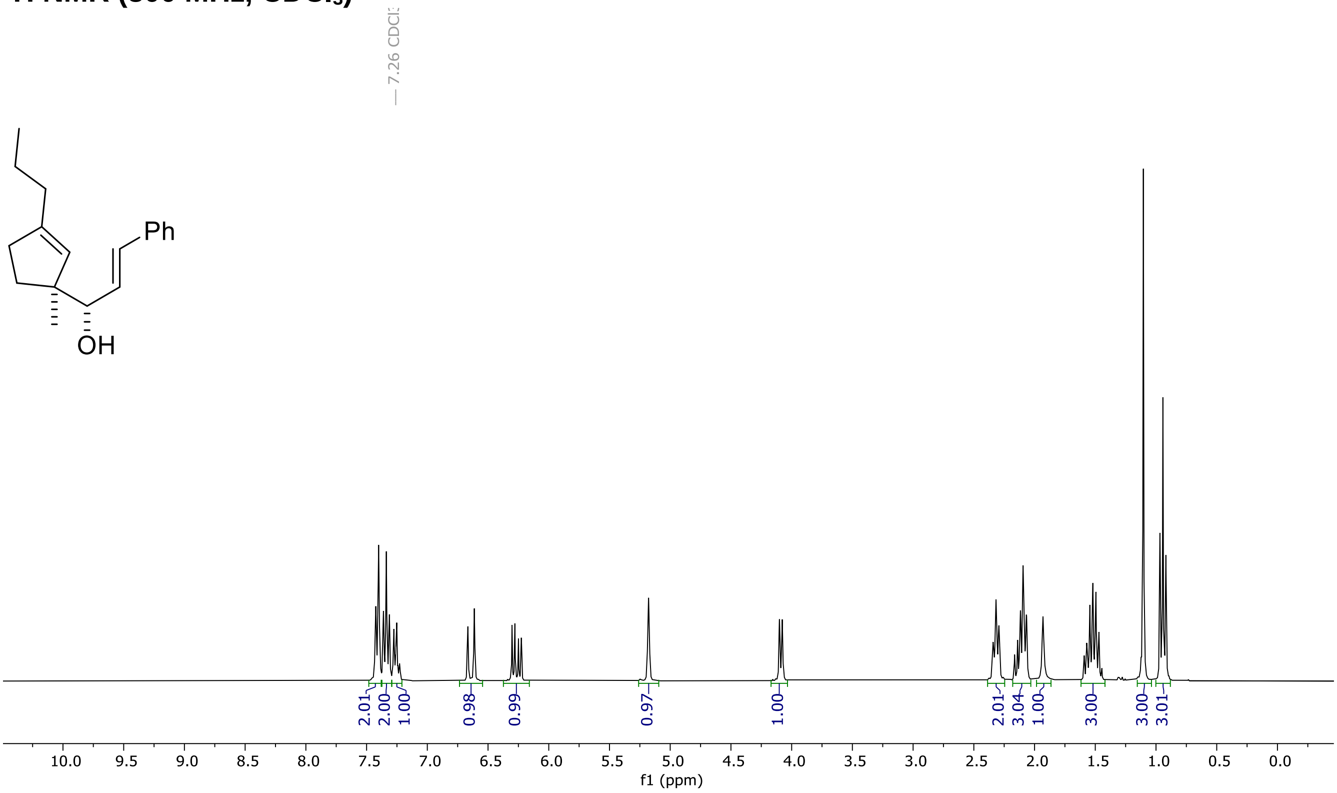

^{13}C NMR (75 MHz, $CDCl_3$)

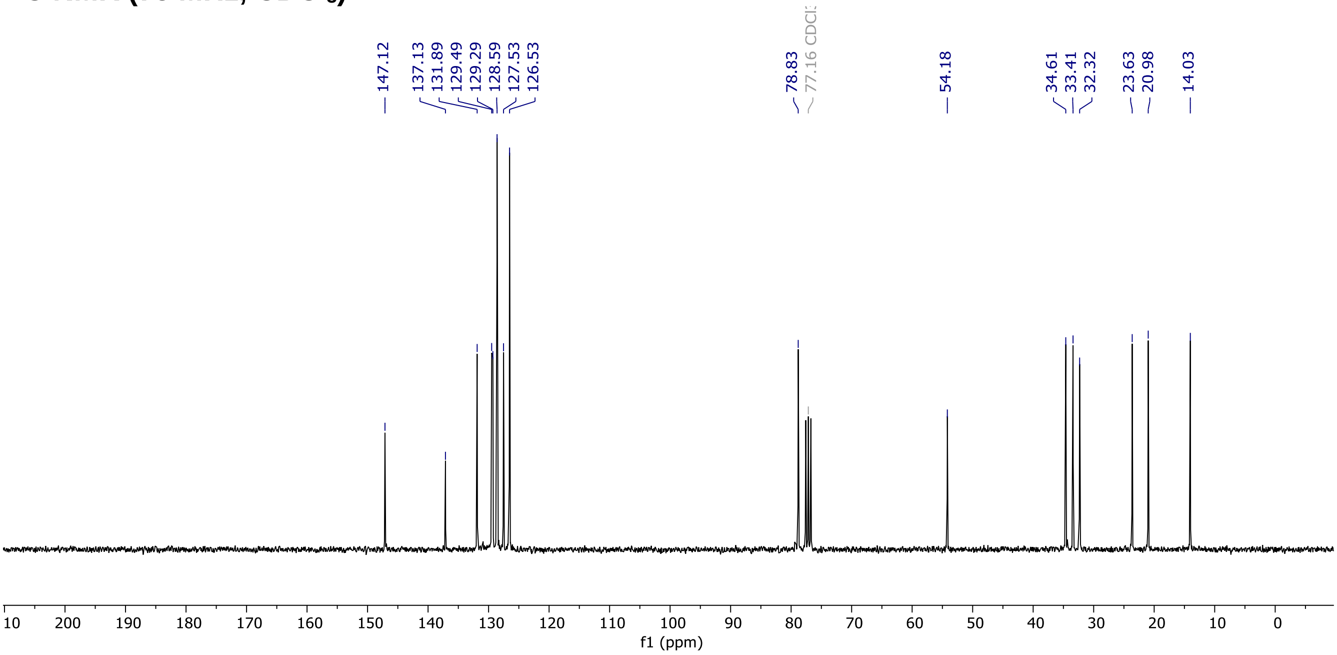

4-((*R)-hydroxy((*S**)-1-methyl-3-phenethylcyclopent-2-en-1-yl)methyl)benzonitrile (8e)**

^{1}H NMR (300 MHz, $CDCl_3$)

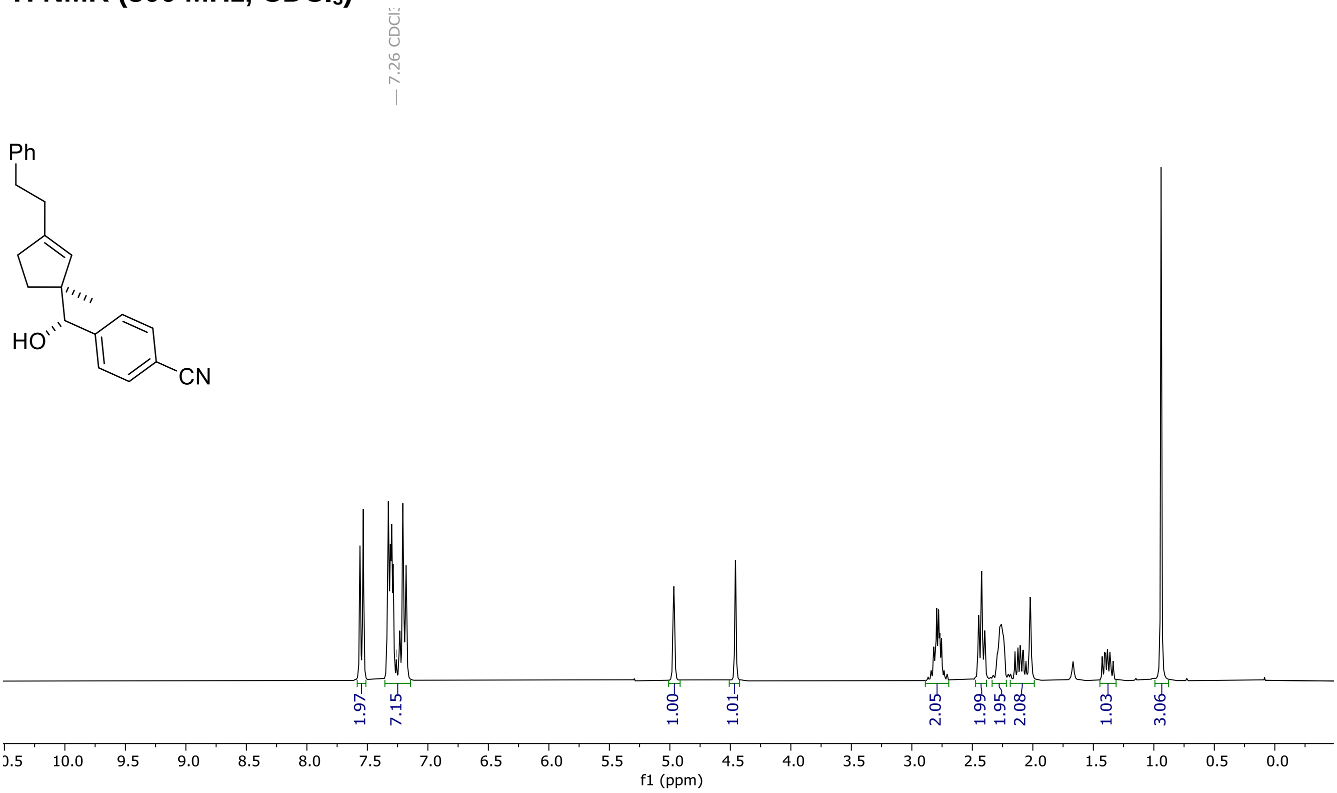

^{13}C NMR (75 MHz, $CDCl_3$)

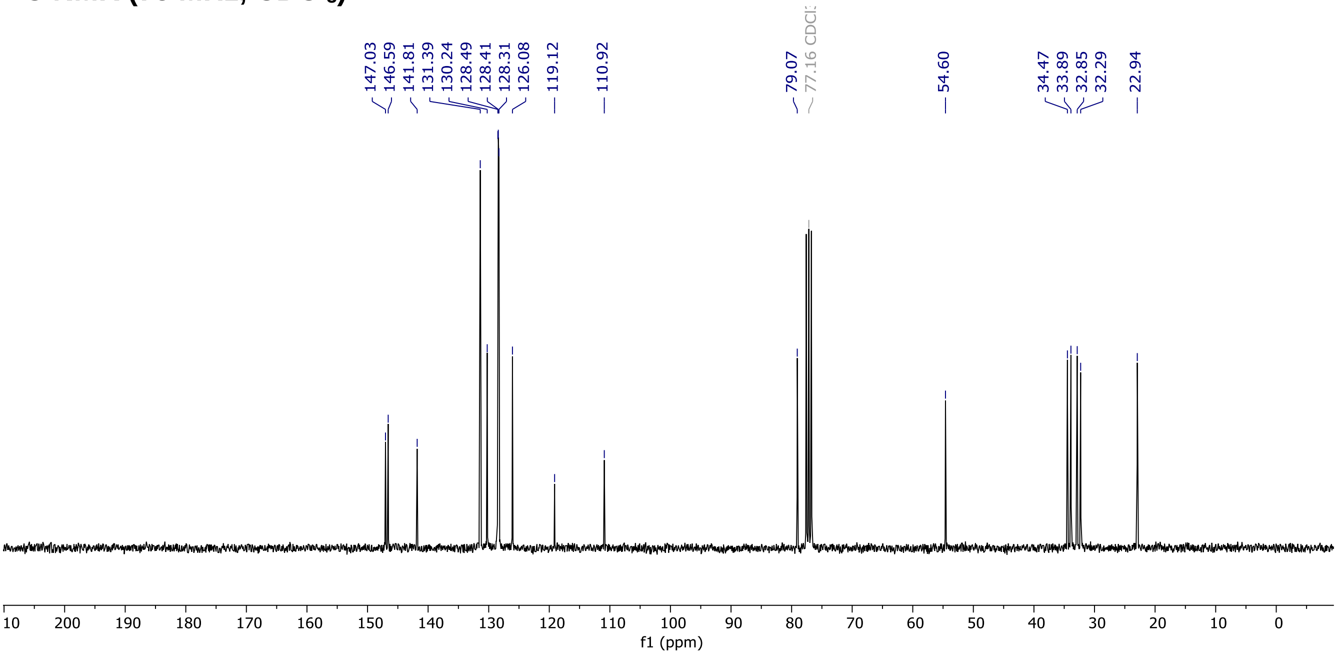

4-((*R)-((*S**)-3-(but-3-en-1-yl)-1-methylcyclopent-2-en-1-yl)(hydroxy)methyl)benzonitrile (8f)**

^{1}H NMR (300 MHz, $CDCl_3$)

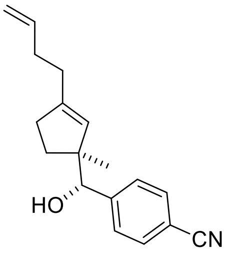

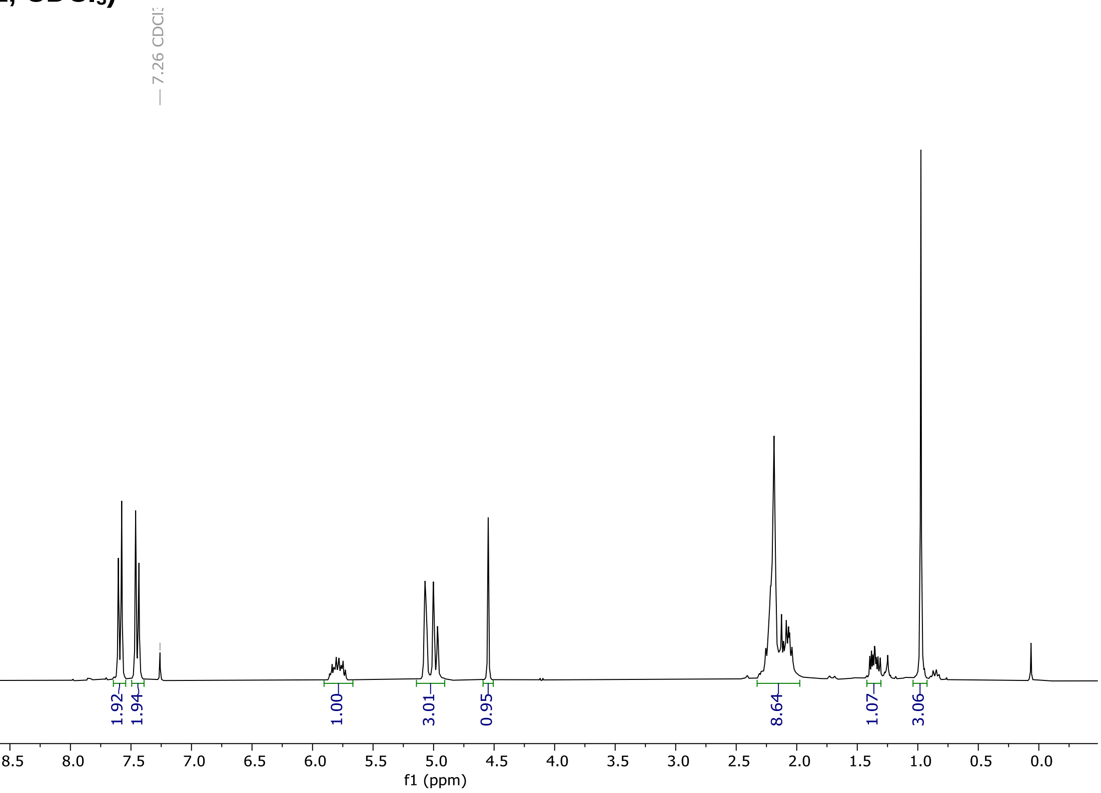

^{13}C NMR (75 MHz, $CDCl_3$)

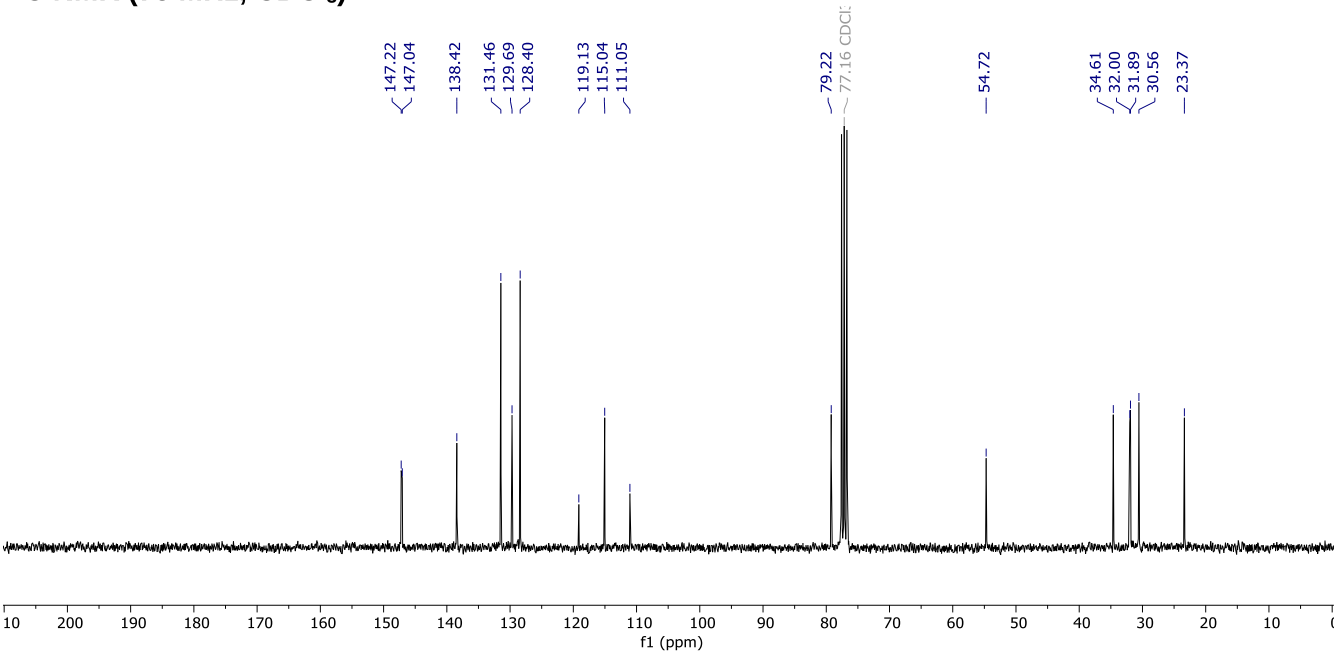

Ethyl 4-((*S)-3-((*R**)-hydroxy(4-nitrophenyl)methyl)-3-methylcyclopent-1-en-1-yl)butanoate (8g)**

^{1}H NMR (300 MHz, CDCl$_3$)

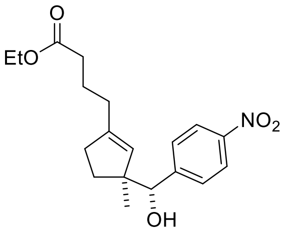

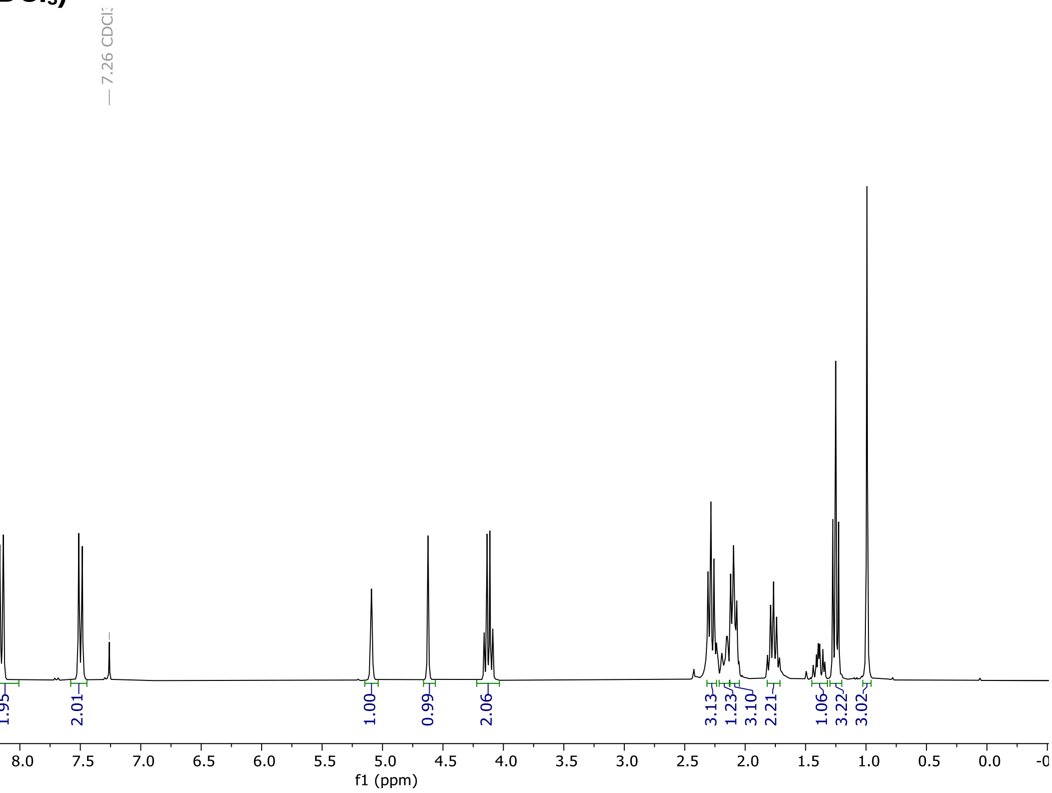

^{13}C NMR (75 MHz, CDCl$_3$)

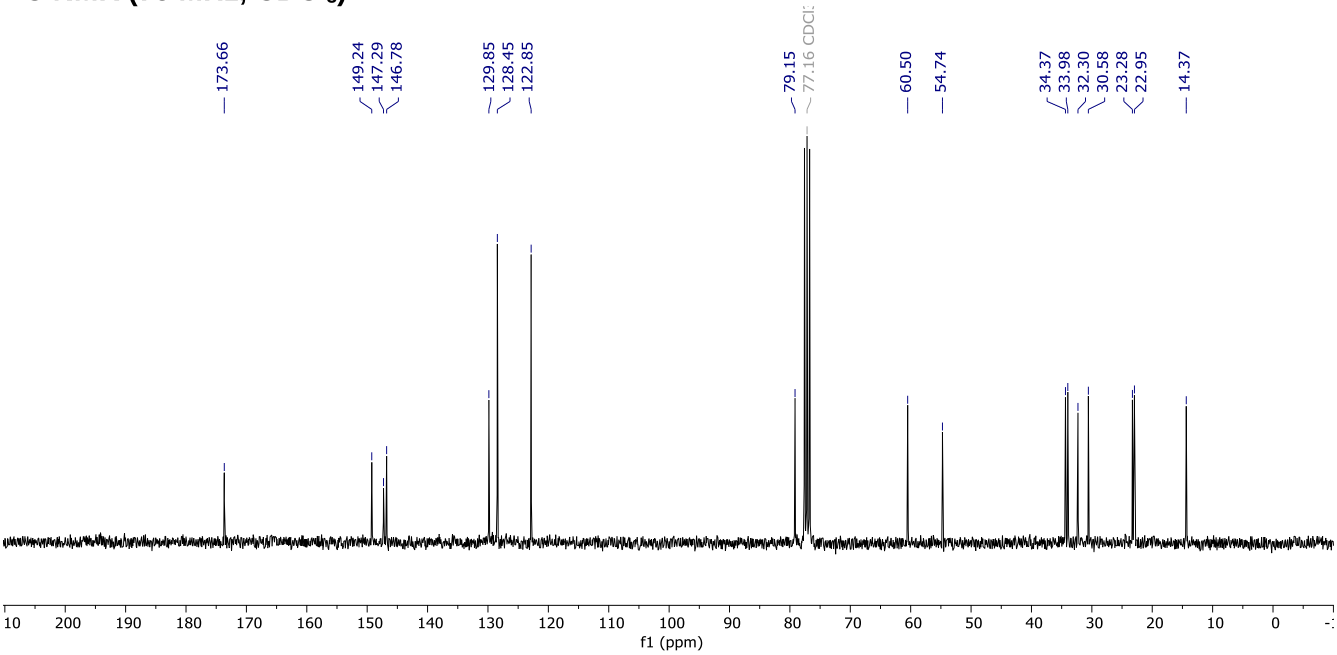

(*R)-((*S**)-3-methylcyclohex-2-en-1-yl)(thiophen-2-yl)methanol (9a)**

^{1}H NMR (300 MHz, $CDCl_3$)

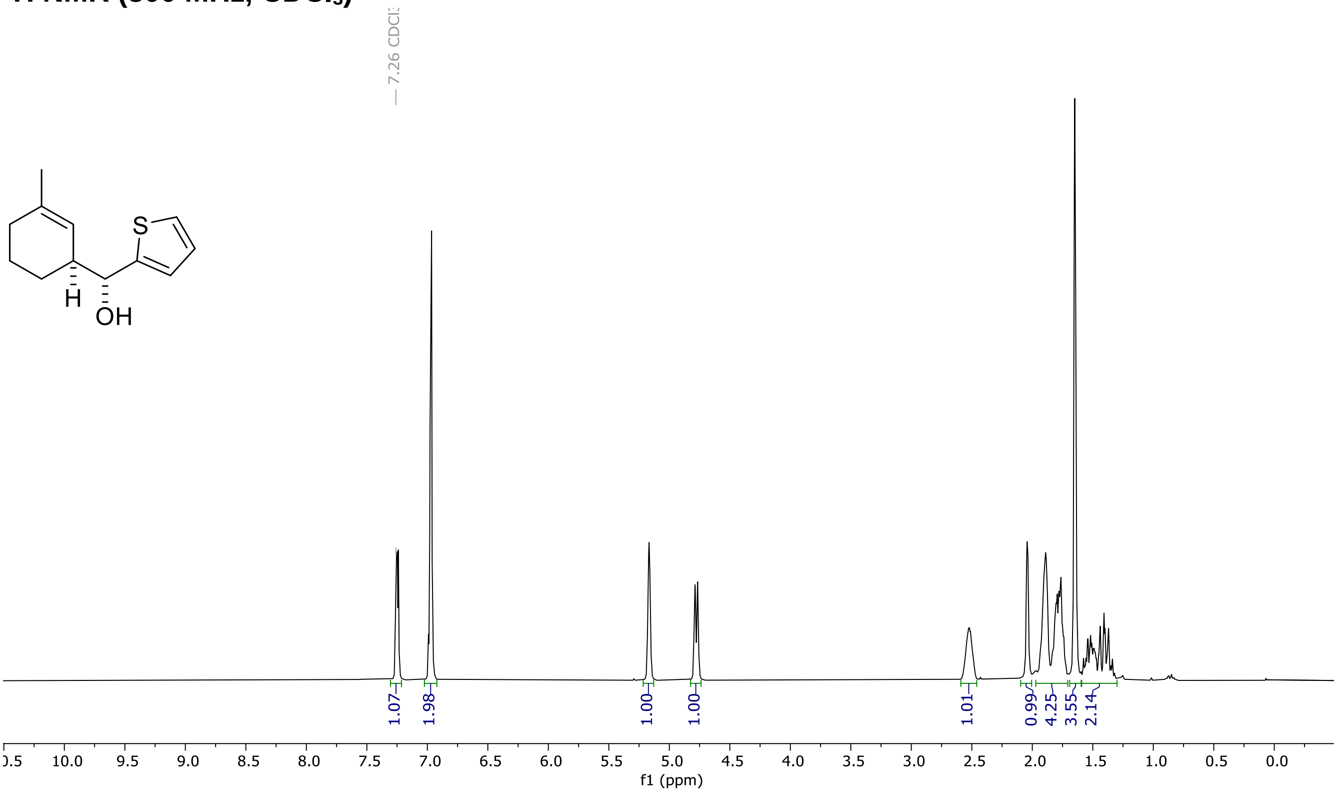

^{13}C NMR (75 MHz, $CDCl_3$)

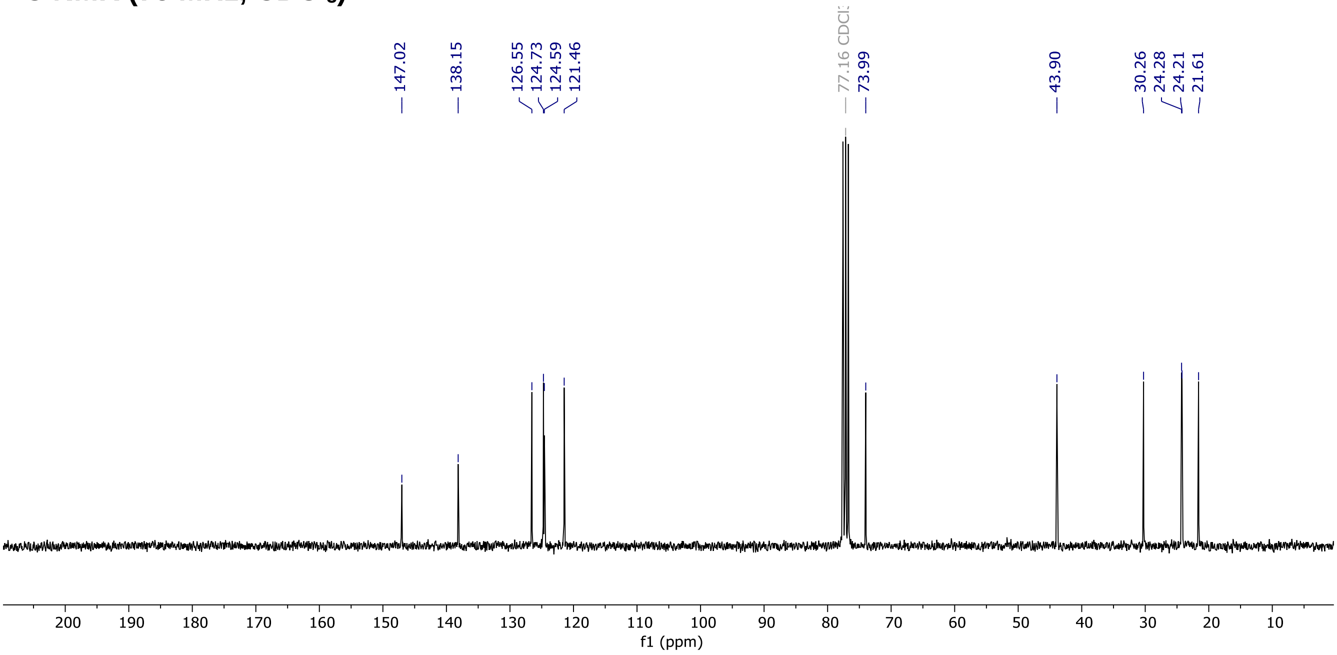

(*R**)-benzofuran-2-yl((*S**)-3-methylcyclohex-2-en-1-yl)methanol (9b)

^{1}H NMR (300 MHz, $CDCl_3$)

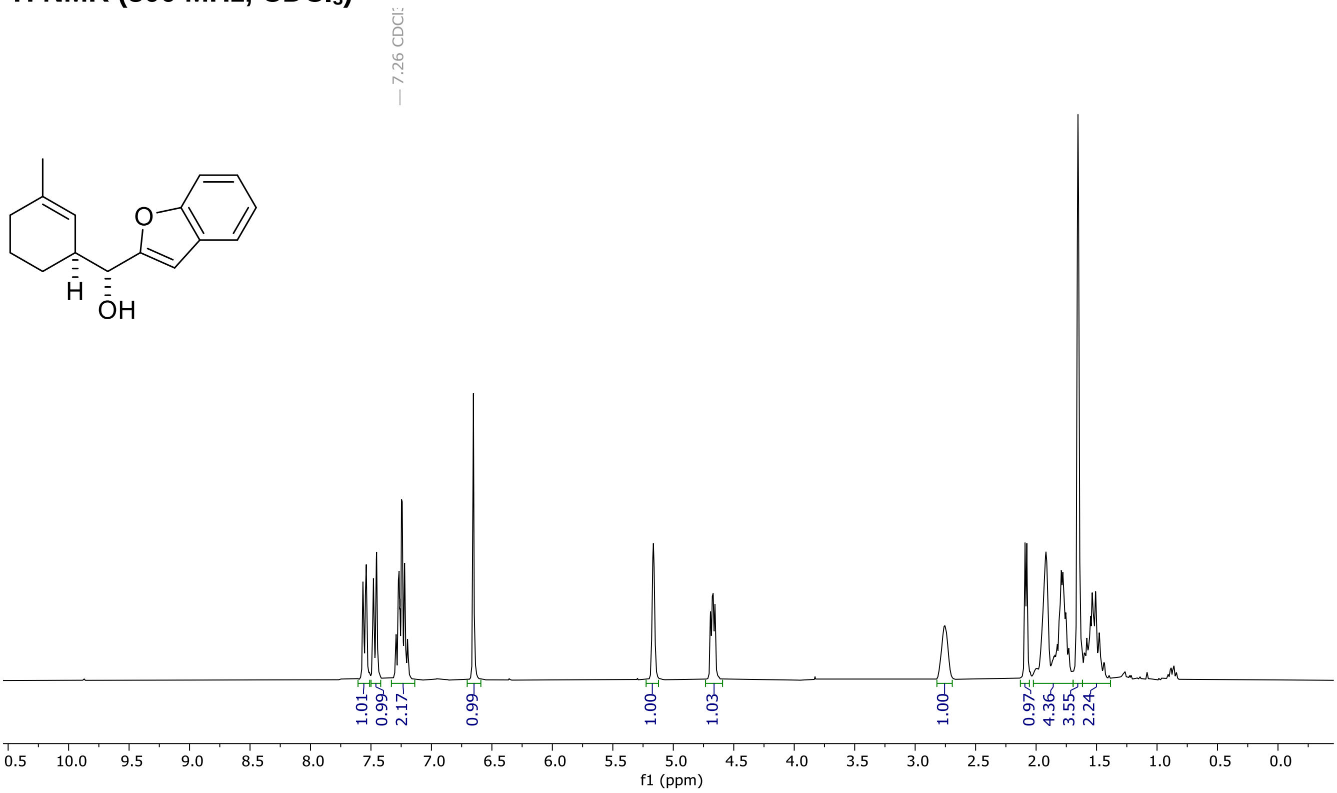

^{13}C NMR (75 MHz, $CDCl_3$)

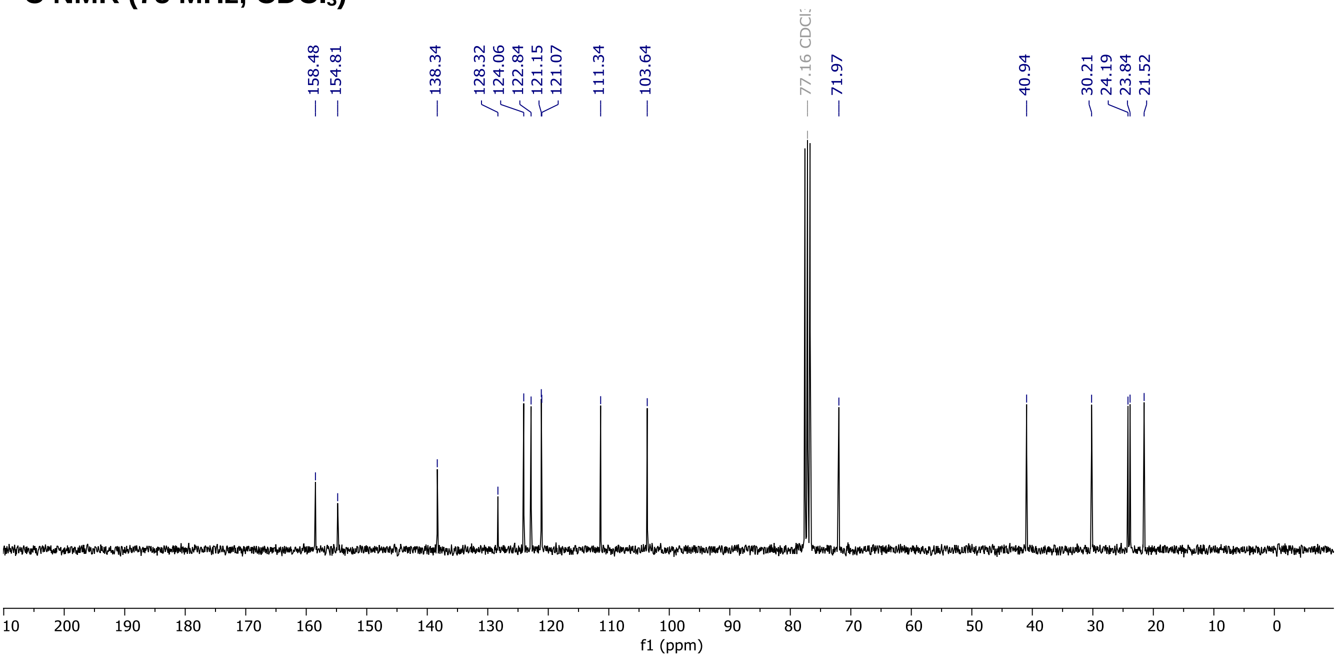

(*R**)-((*S**)-3-methylcyclohex-2-en-1-yl)(4-nitrophenyl)methanol (9c)

^{1}H NMR (300 MHz, $CDCl_3$)

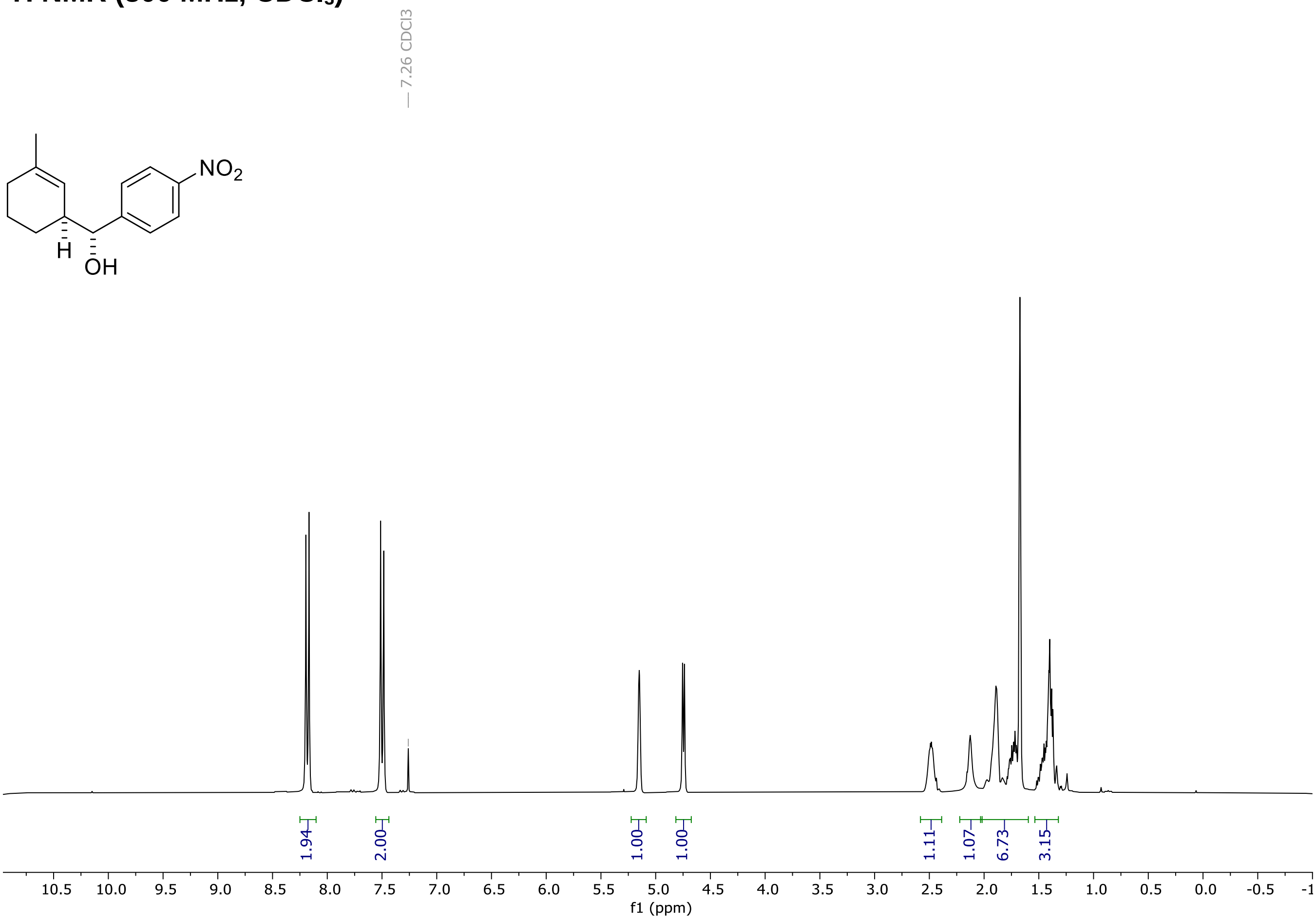

^{13}C NMR (75 MHz, $CDCl_3$)

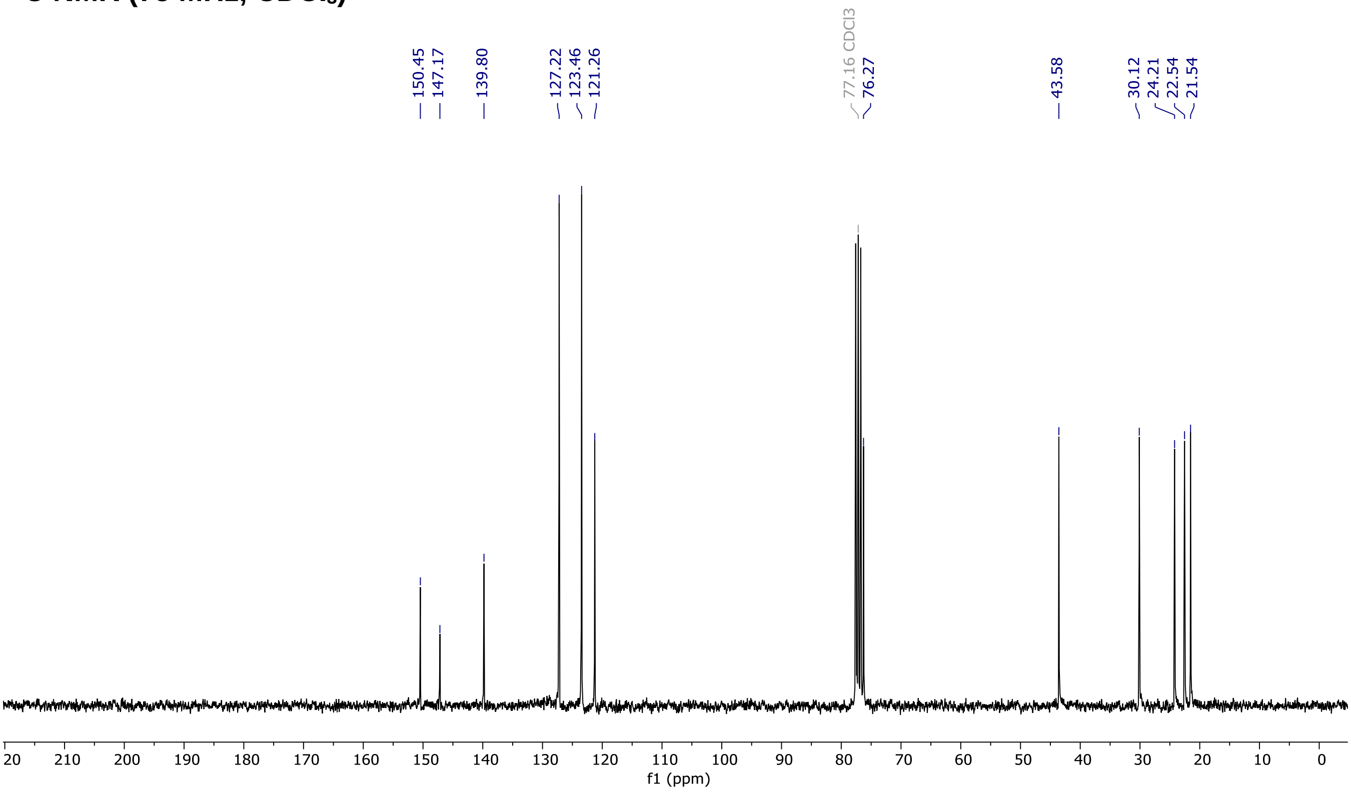

(*R)-benzofuran-2-yl((*S**)-3-cyclopropylcyclohept-2-en-1-yl)methanol (10)**

^{1}H NMR (300 MHz, $CDCl_3$)

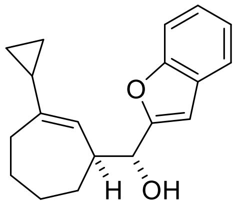

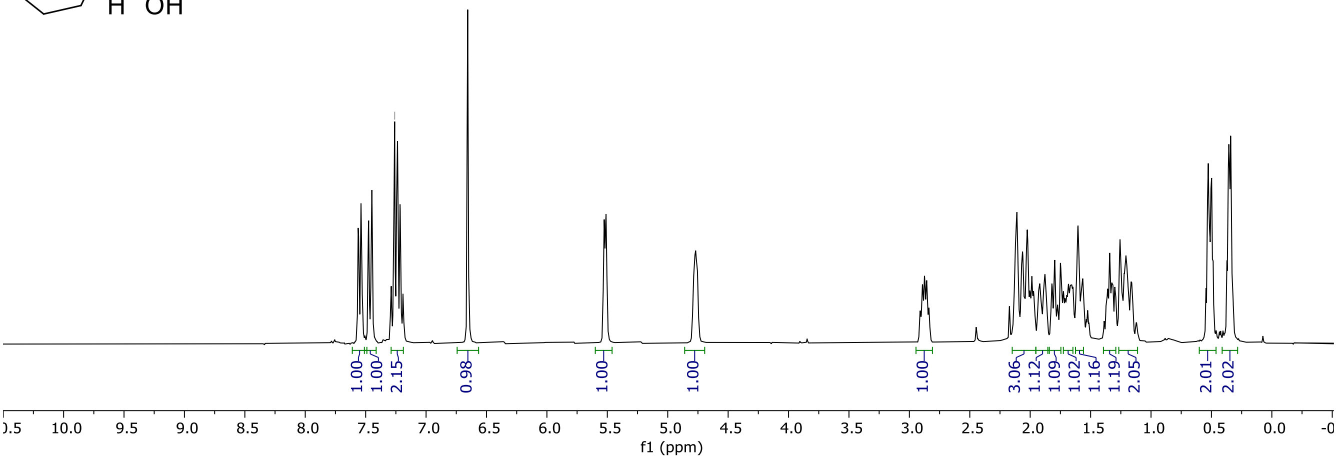

^{13}C NMR (75 MHz, $CDCl_3$)

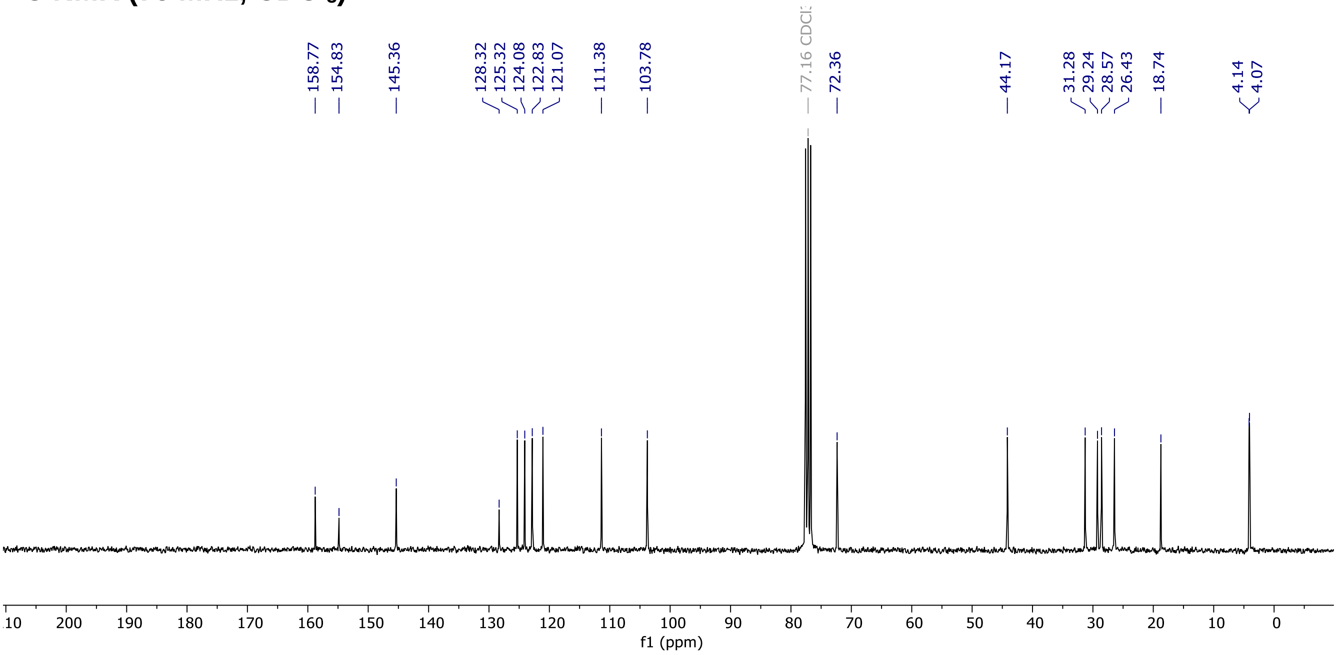

Benzyl (*S)-6-((*S**)-(2-bromophenyl)(hydroxy)methyl)-4-methyl-3,6-dihydropyridine-1(2H)-carboxylate (11a)**

^{1}H NMR (401 MHz, CDCl$_3$)

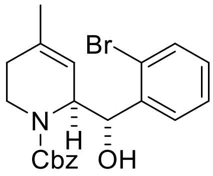

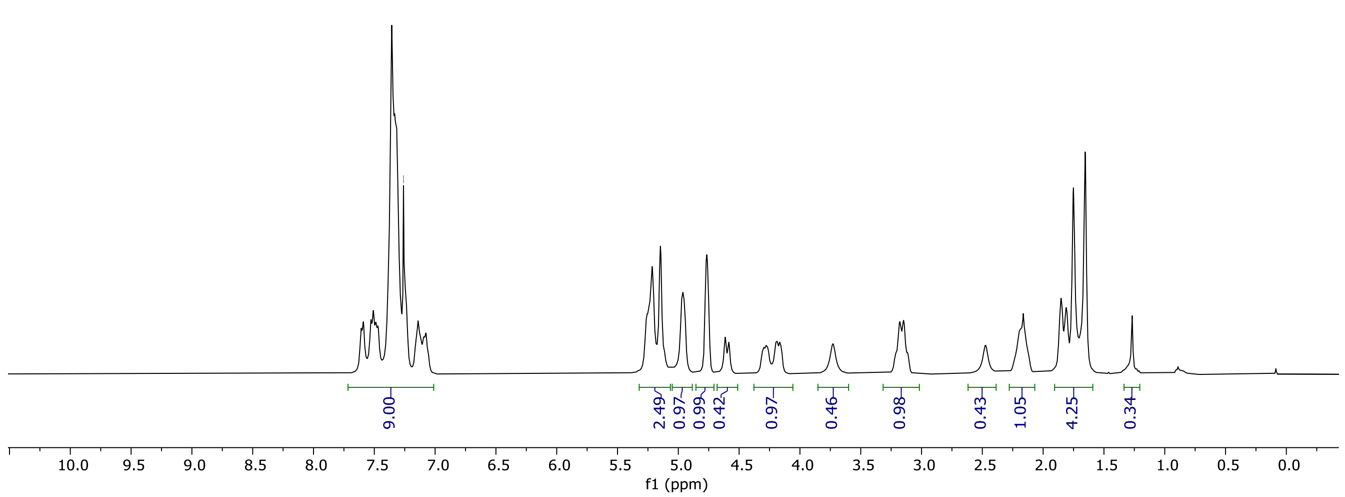

^{13}C NMR (101 MHz, CDCl$_3$)

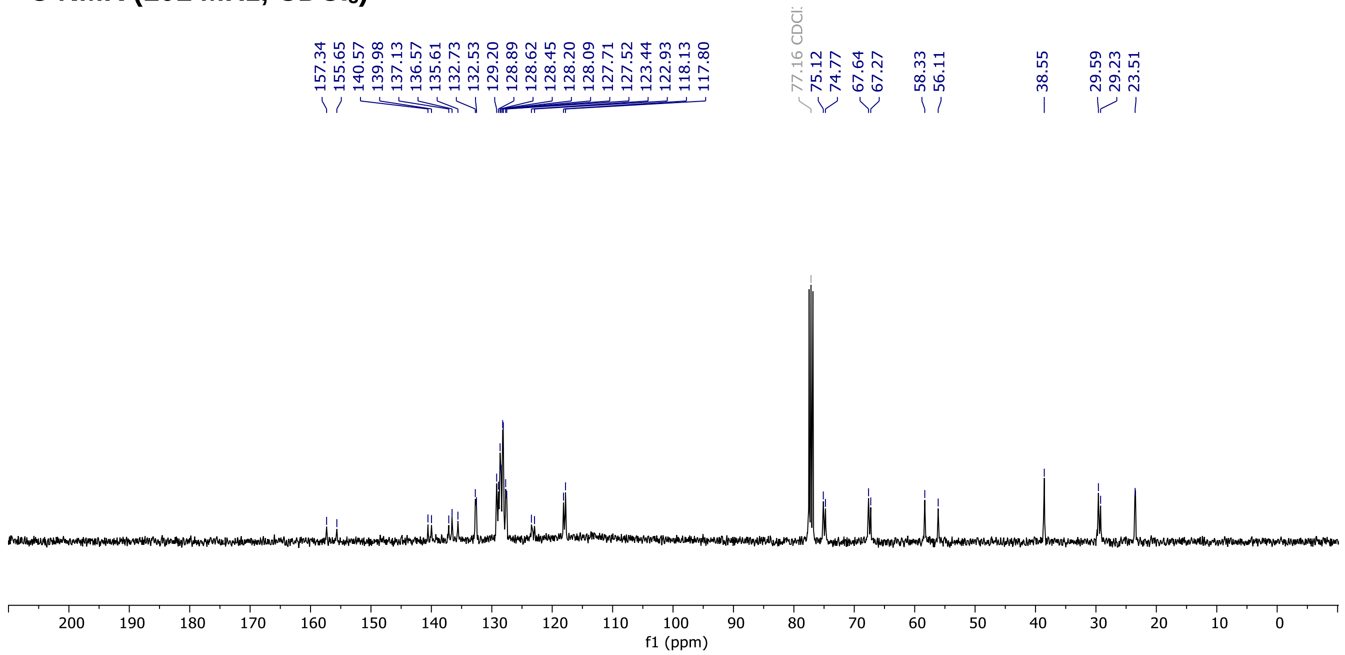

Benzyl (*S)-6-((*S**)-(4-cyanophenyl)(hydroxy)methyl)-4-propyl-3,6-dihydropyridine-1(2H)-carboxylate (11b)**

^{1}H NMR (300 MHz, CDCl$_3$)

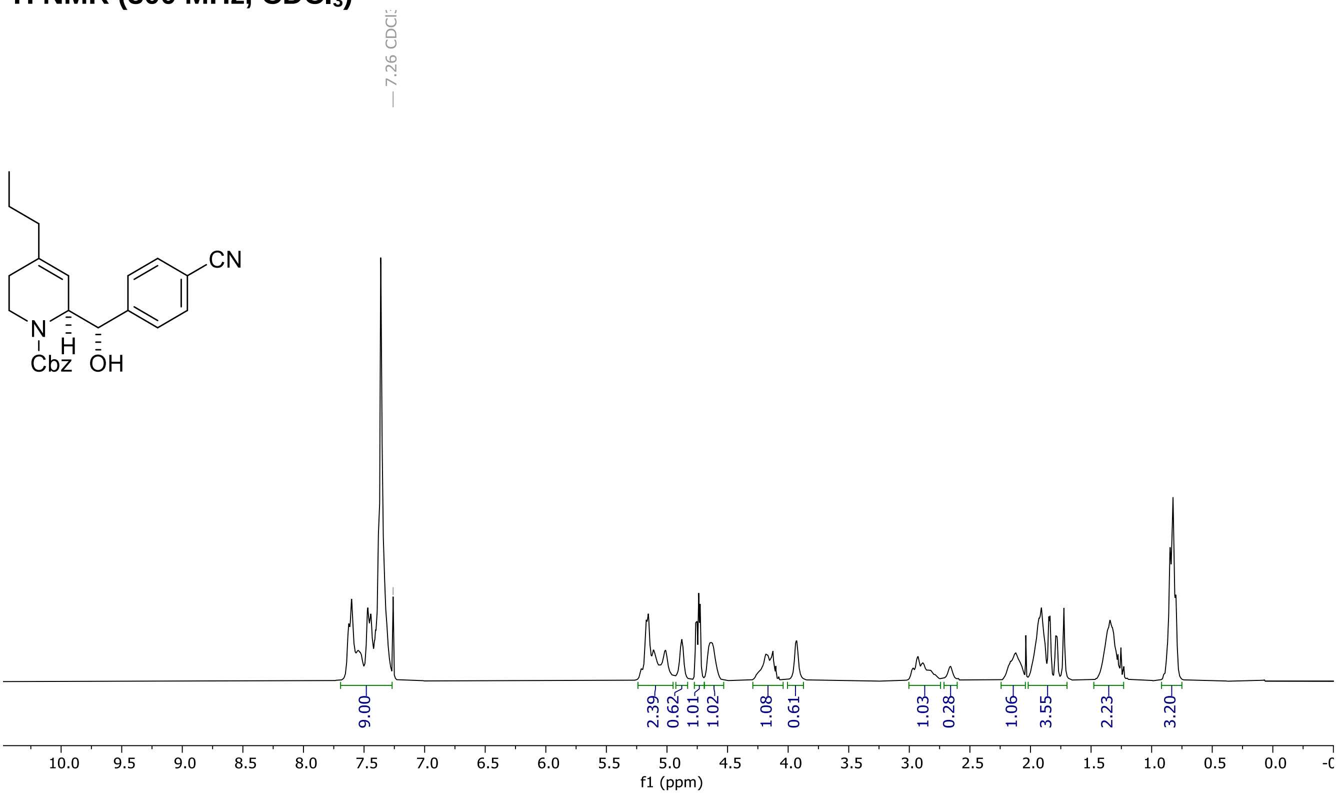

^{13}C NMR (75 MHz, CDCl$_3$)

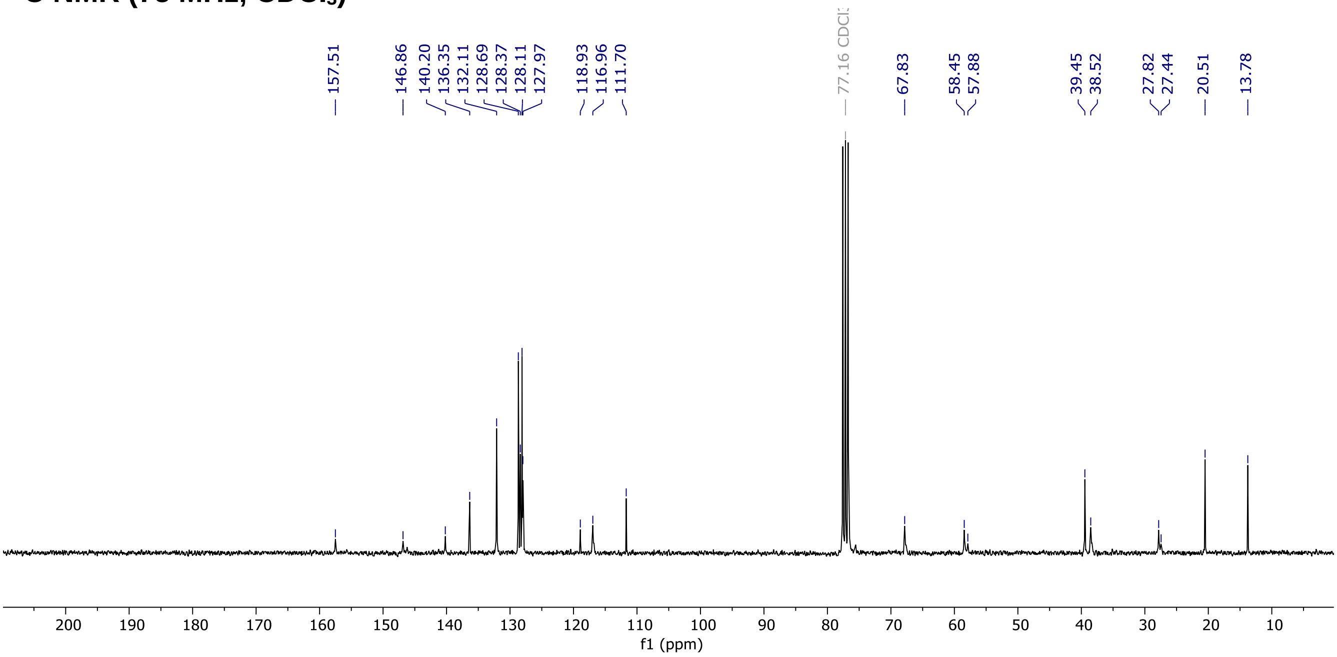

Benzyl (*S)-6-((*S**,*E*)-1-hydroxy-3-phenylallyl)-4-phenethyl-3,6-dihydropyridine-1(2H)-carboxylate (11c)**

^{1}H NMR (300 MHz, $CDCl_3$)

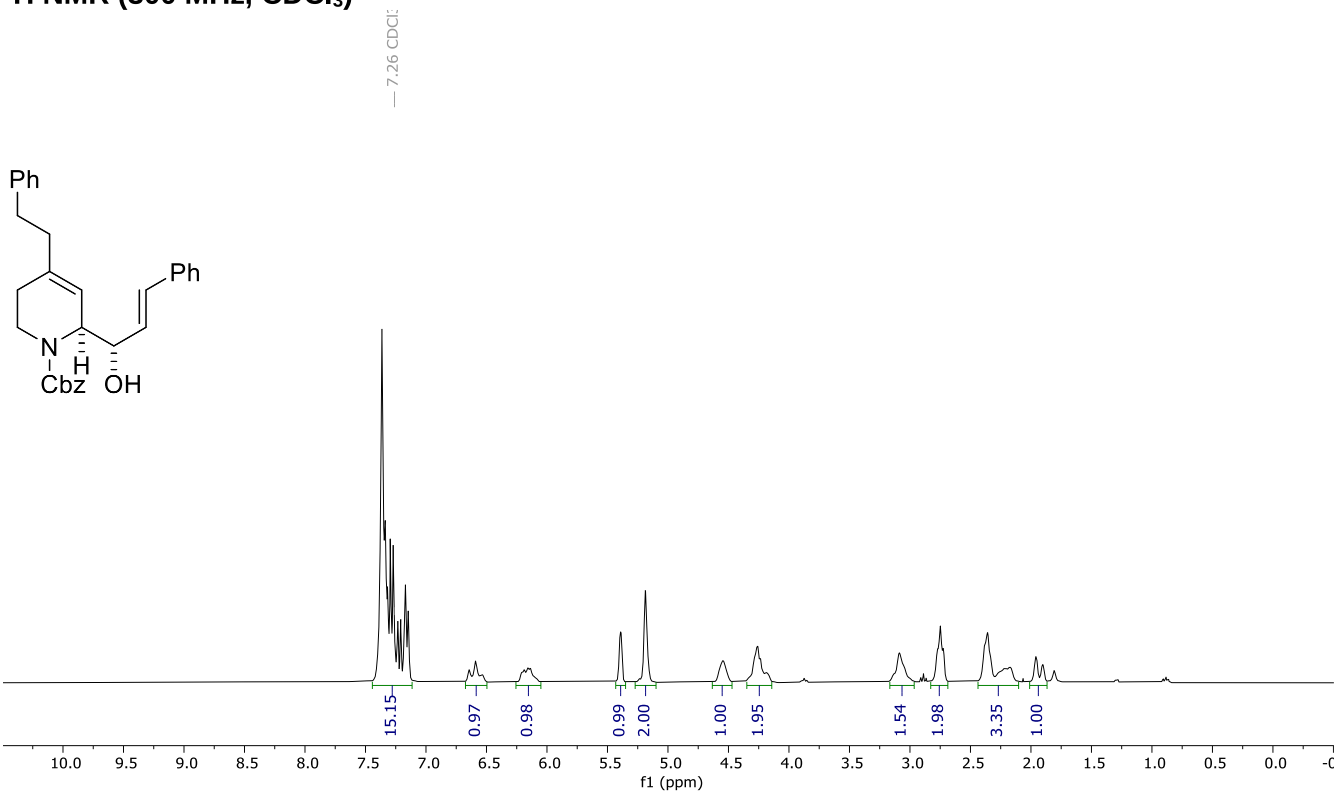

^{13}C NMR (75 MHz, $CDCl_3$)

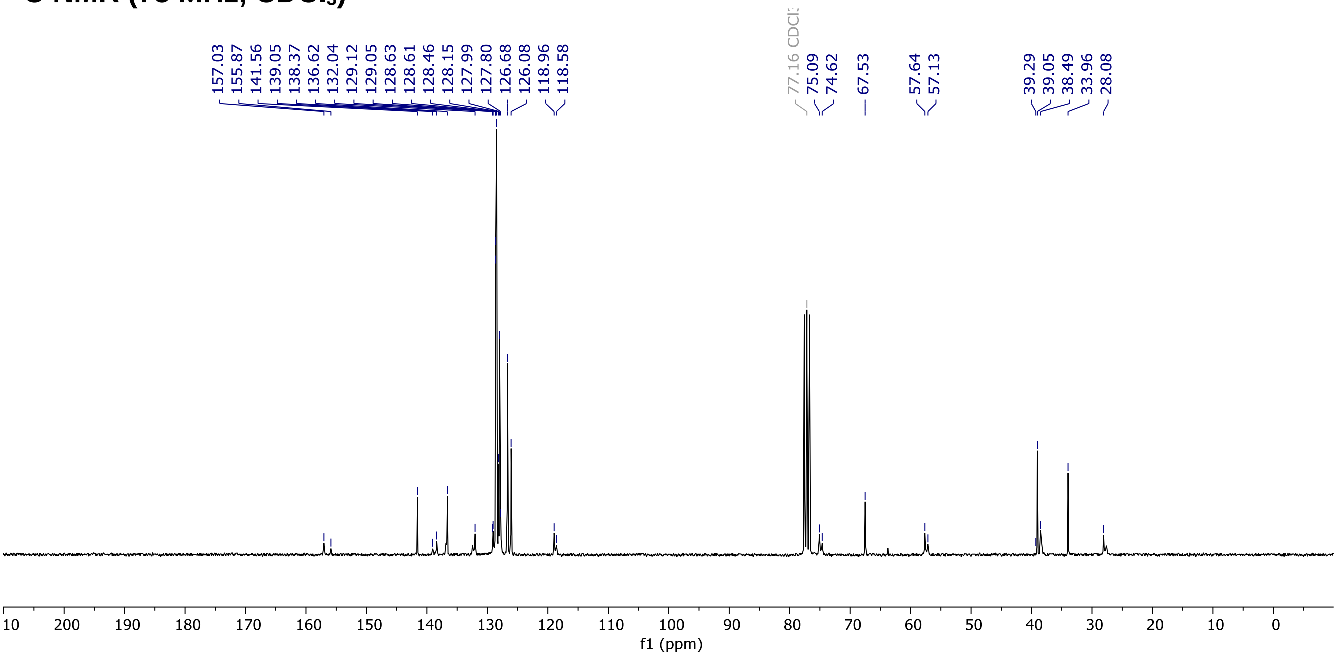

Benzyl (*S)-6-((*S**)-1-hydroxy-2-methylallyl)-4-methyl-3,6-dihydropyridine-1(2H)-carboxylate (11d)**

^{1}H NMR (300 MHz, $CDCl_3$)

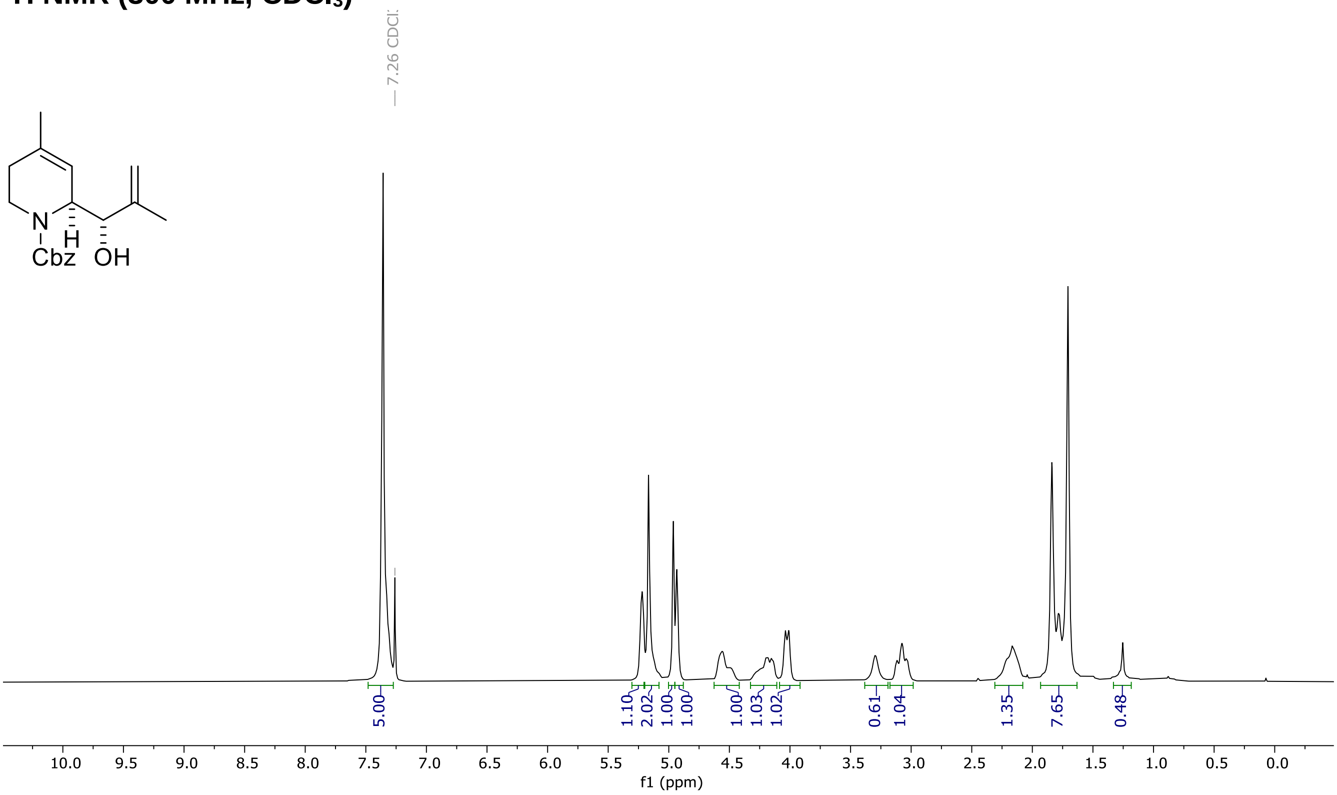

^{13}C NMR (75 MHz, $CDCl_3$)

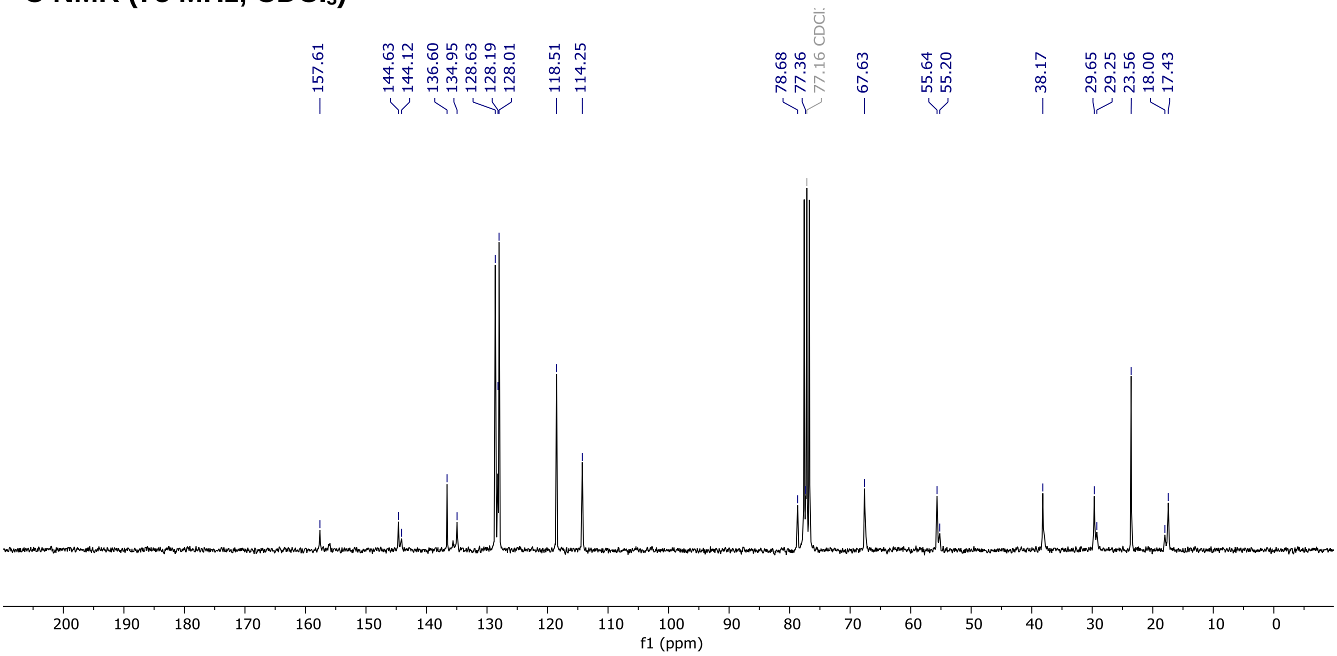

Benzyl (*S)-6-((*S**)-hydroxy(phenyl)methyl)-4-methyl-3,6-dihydropyridine-1(2H)-carboxylate (11e)**

^{1}H NMR (300 MHz, $CDCl_3$)

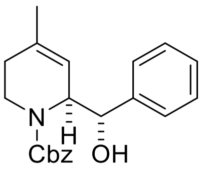

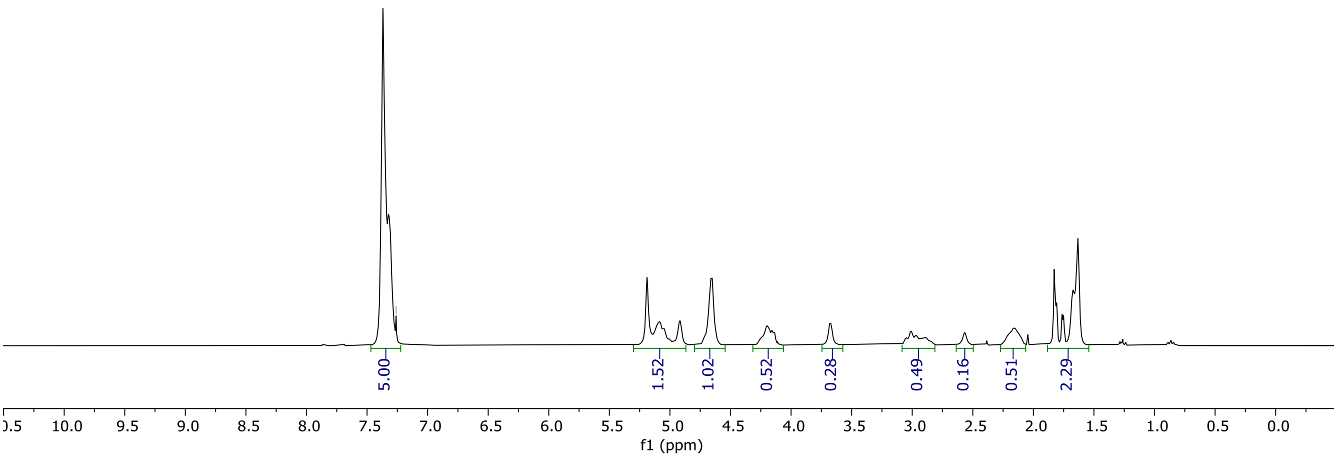

^{13}C NMR (75 MHz, $CDCl_3$)

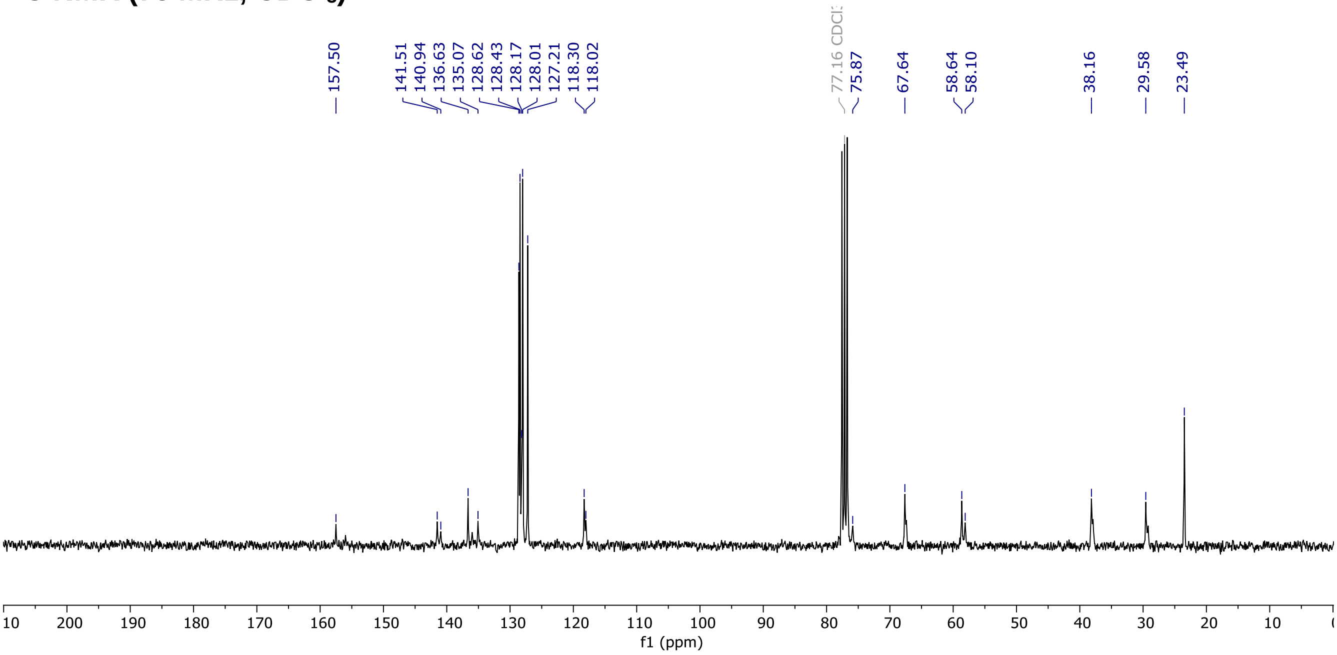

Benzyl (*S)-6-((*S**)-(4-cyanophenyl)(hydroxy)methyl)-4-methyl-3,6-dihydropyridine-1(2H)-carboxylate (11f)**

^{1}H NMR (300 MHz, CDCl$_3$)

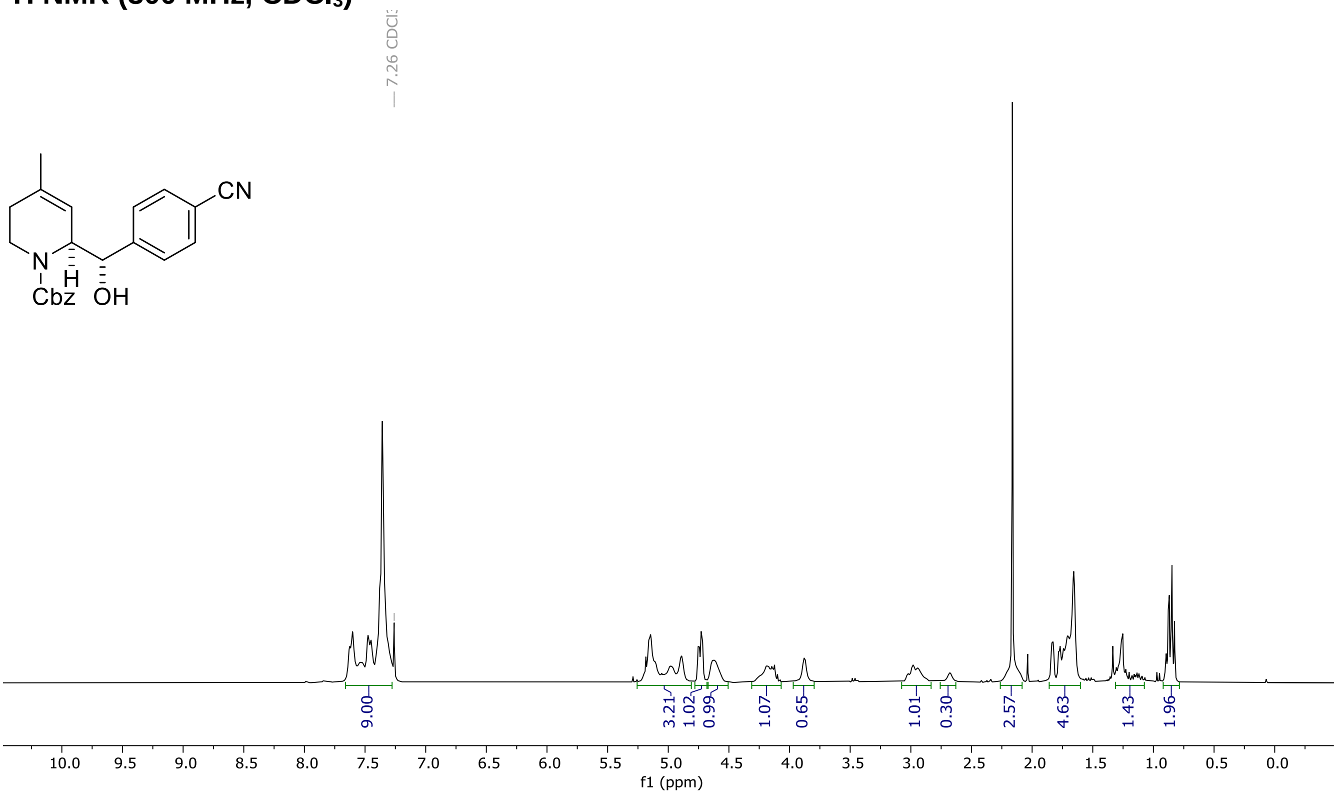

^{13}C NMR (75 MHz, CDCl$_3$)

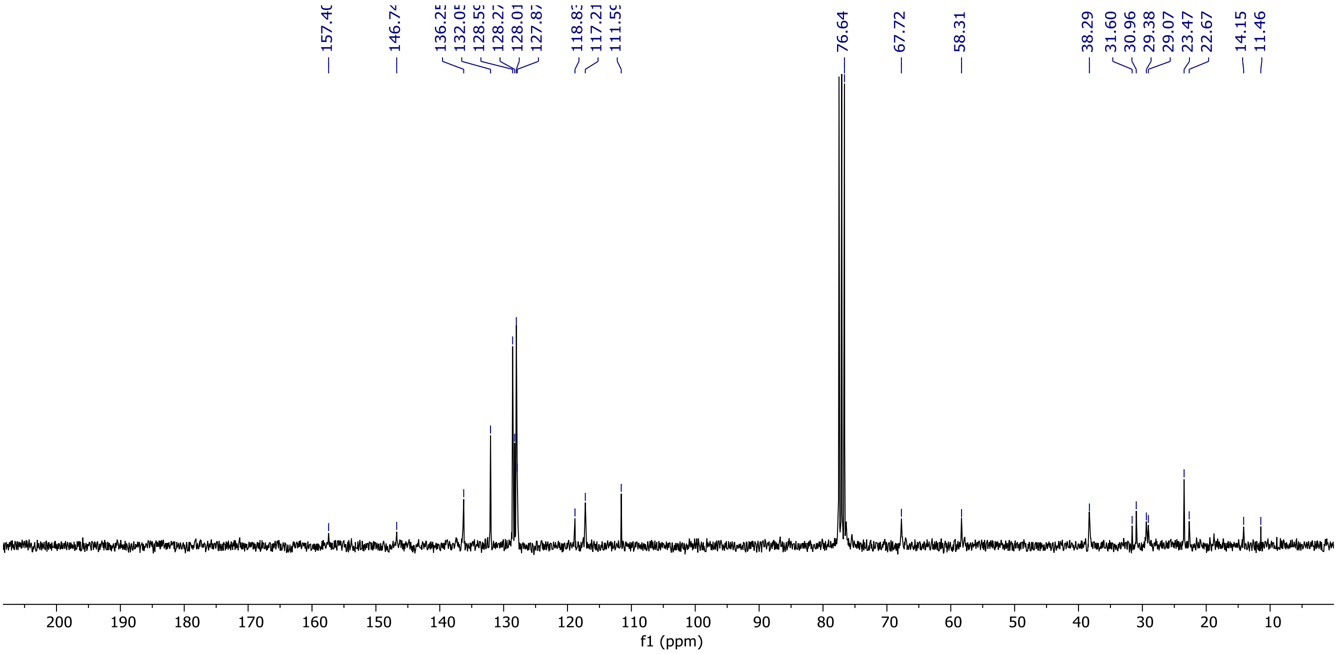

Benzyl (*S)-6-((*S**)-hydroxy(4-nitrophenyl)methyl)-4-methyl-3,6-dihydropyridine-1(2H)-carboxylate (11g)**

^{1}H NMR (300 MHz, $CDCl_3$)

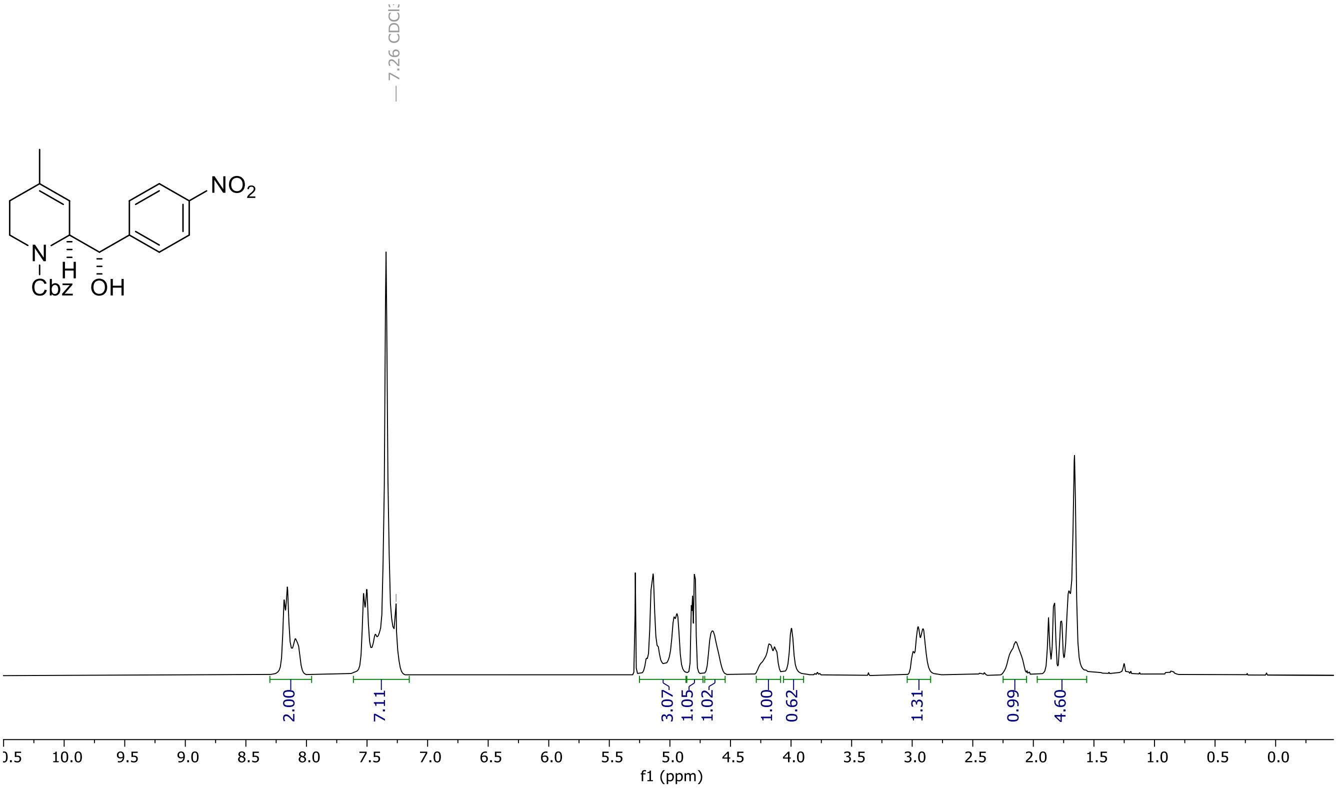

^{13}C NMR (75 MHz, $CDCl_3$)

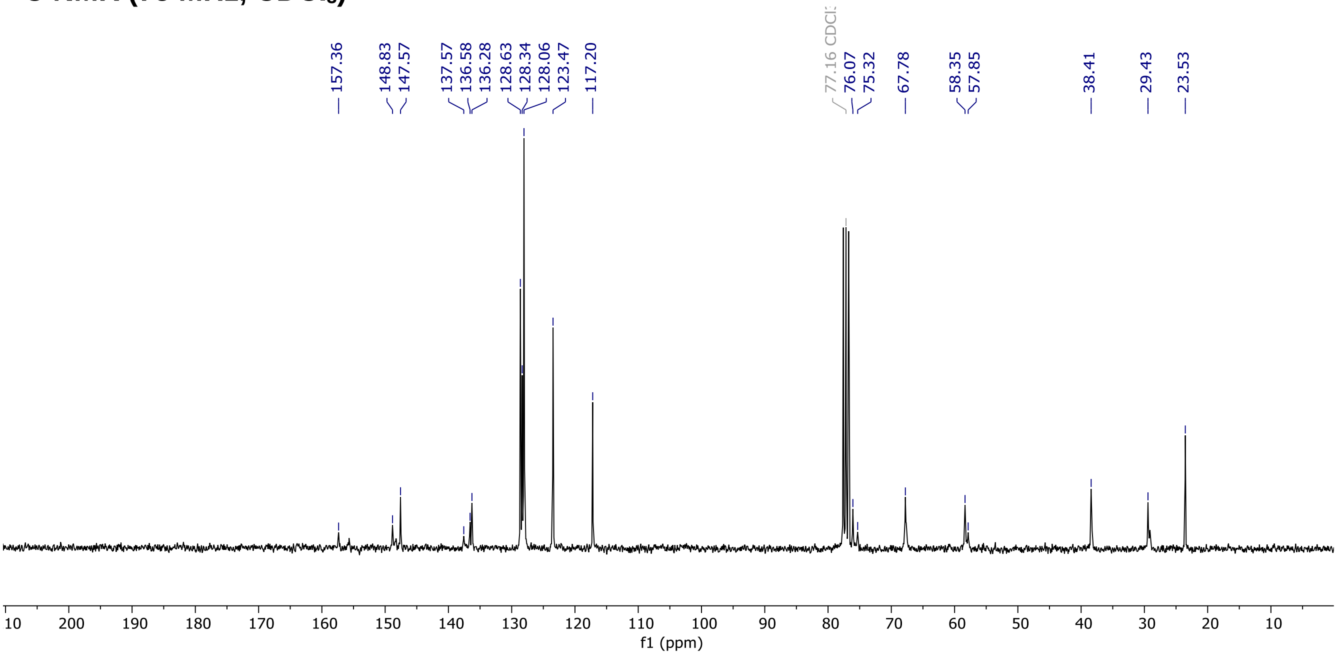

(*R)-(2-bromophenyl)((*S**)-1-methyl-2-((Z)-pent-2-en-1-yl)-3-phenethylcyclopent-2-en-1-yl)methanol (12a)**

^{1}H NMR (300 MHz, $CDCl_3$)

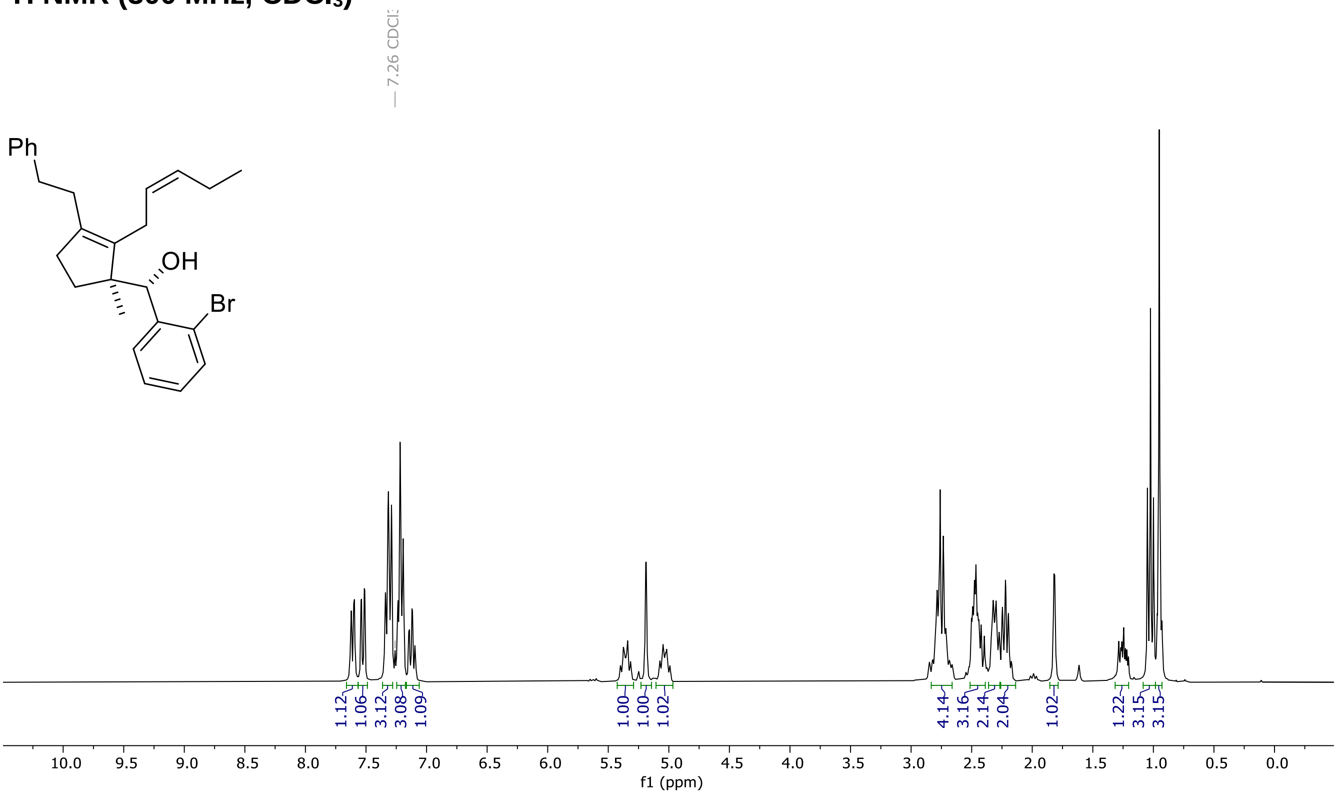

^{13}C NMR (75 MHz, $CDCl_3$)

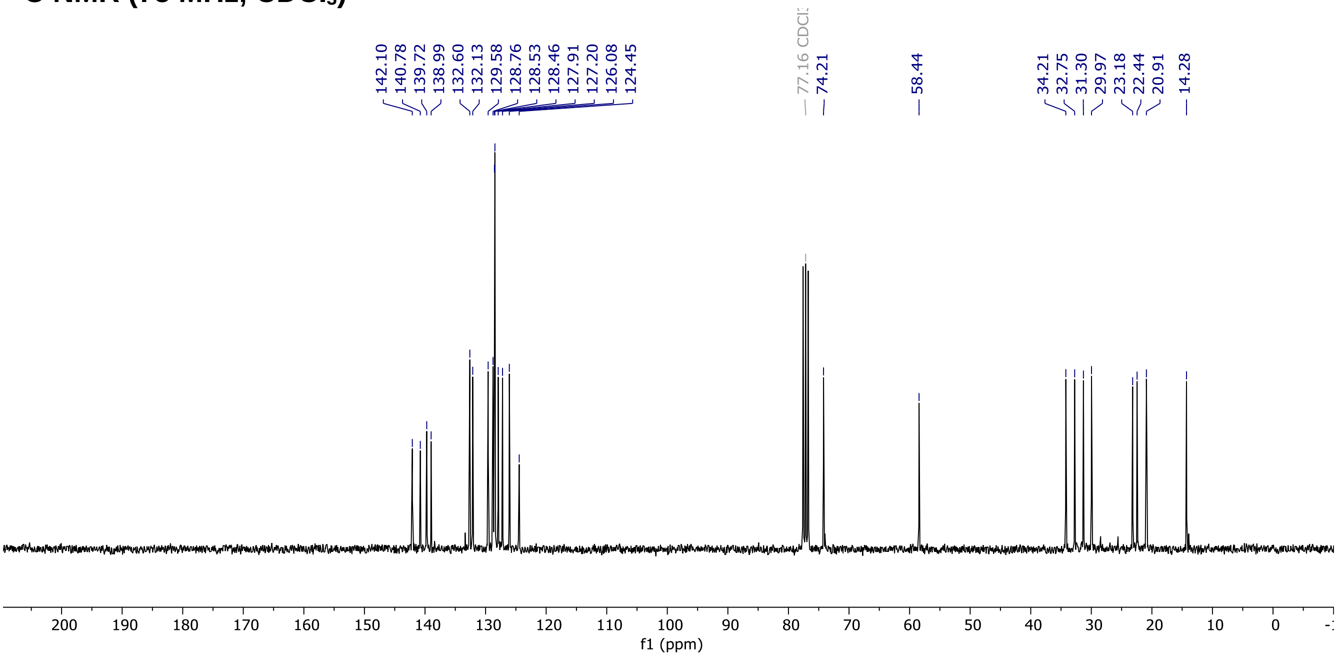

(*S**,*E*)-3-(4-methoxyphenyl)-1-((*S**)-1-methyl-2-((Z)-pent-2-en-1-yl)-3-phenethylcyclopent-2-en-1-yl)prop-2-en-1-ol (12b)

^{1}H NMR (300 MHz, $CDCl_3$)

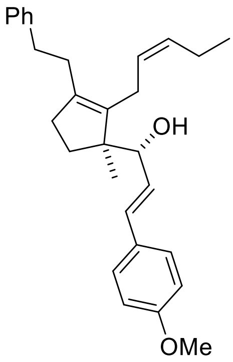

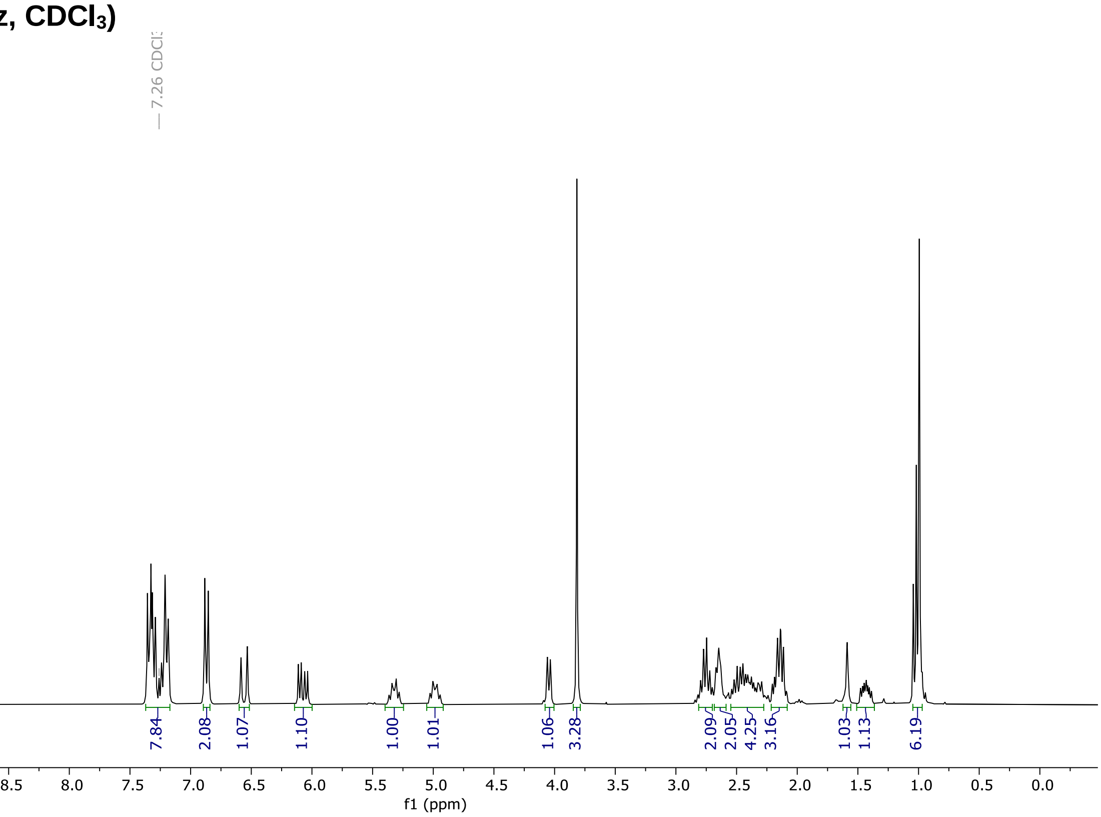

^{13}C NMR (75 MHz, $CDCl_3$)

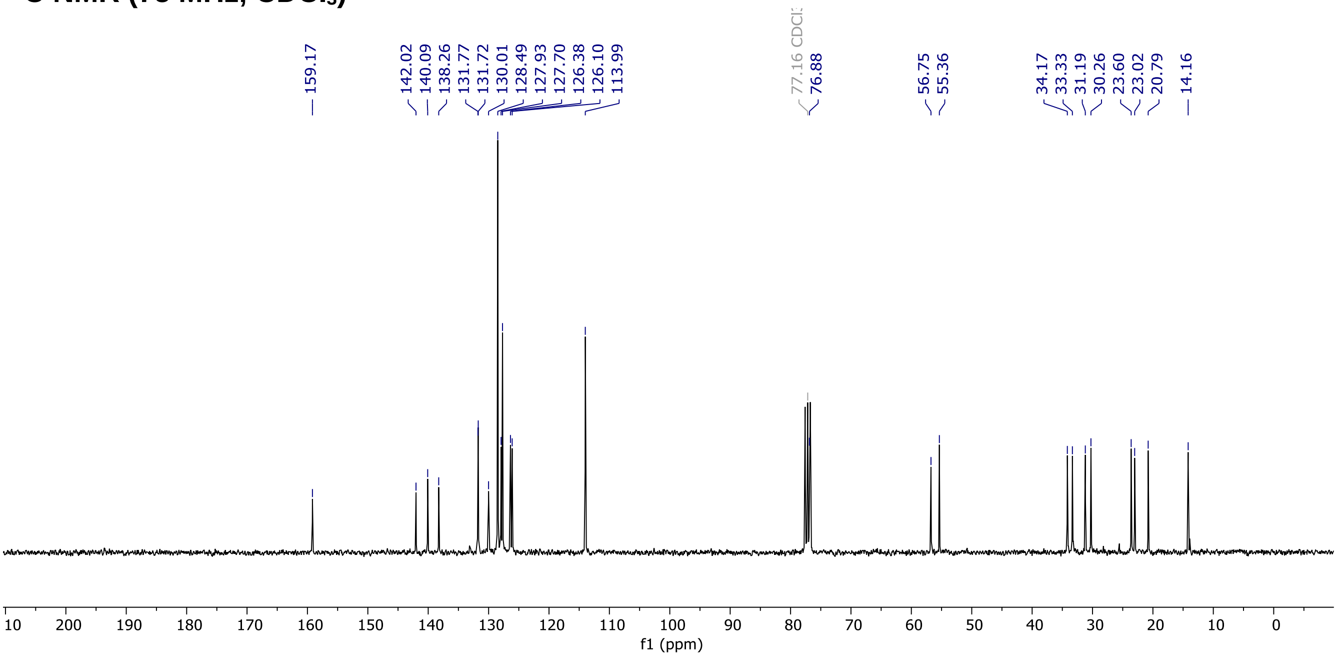

(*S,E)-3-(4-methoxyphenyl)-1-((*S**)-1-methyl-2-((Z)-pent-2-en-1-yl)-3-propylcyclopent-2-en-1-yl)prop-2-en-1-ol (12c)**

^{1}H NMR (300 MHz, $CDCl_3$)

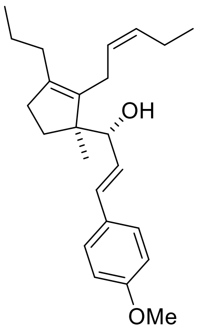

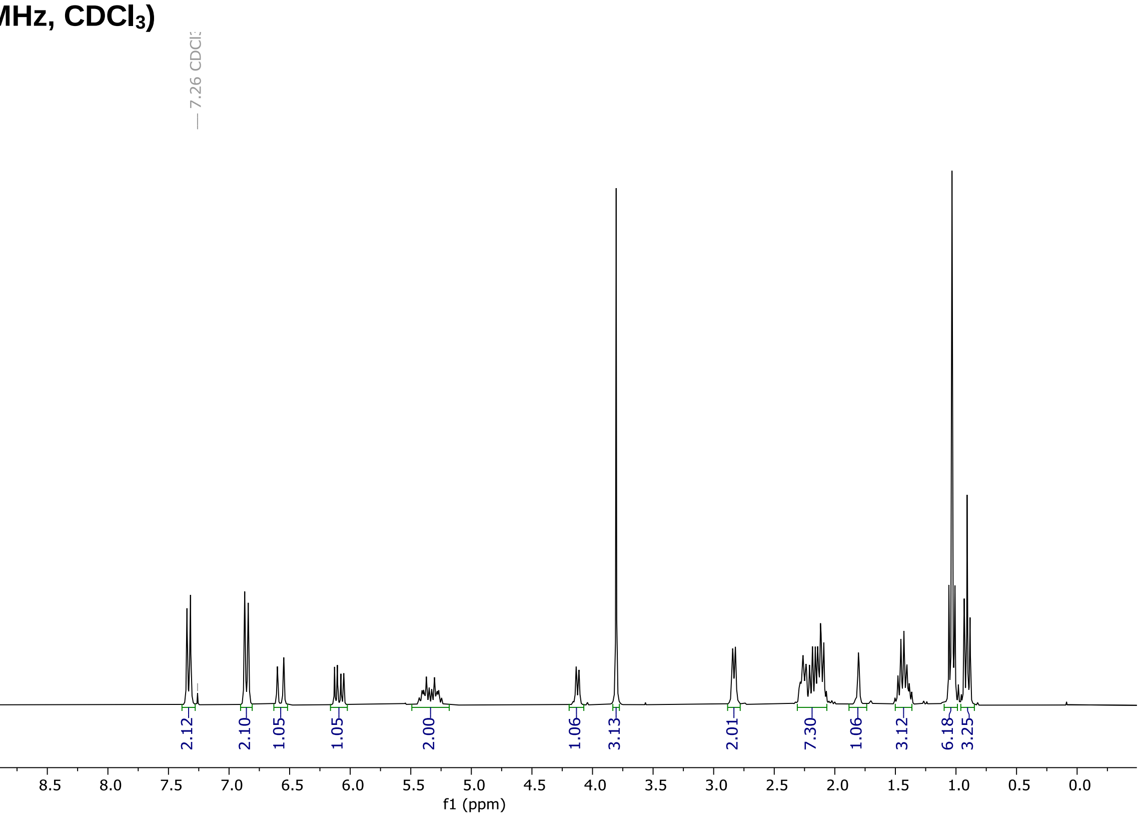

^{13}C NMR (75 MHz, $CDCl_3$)

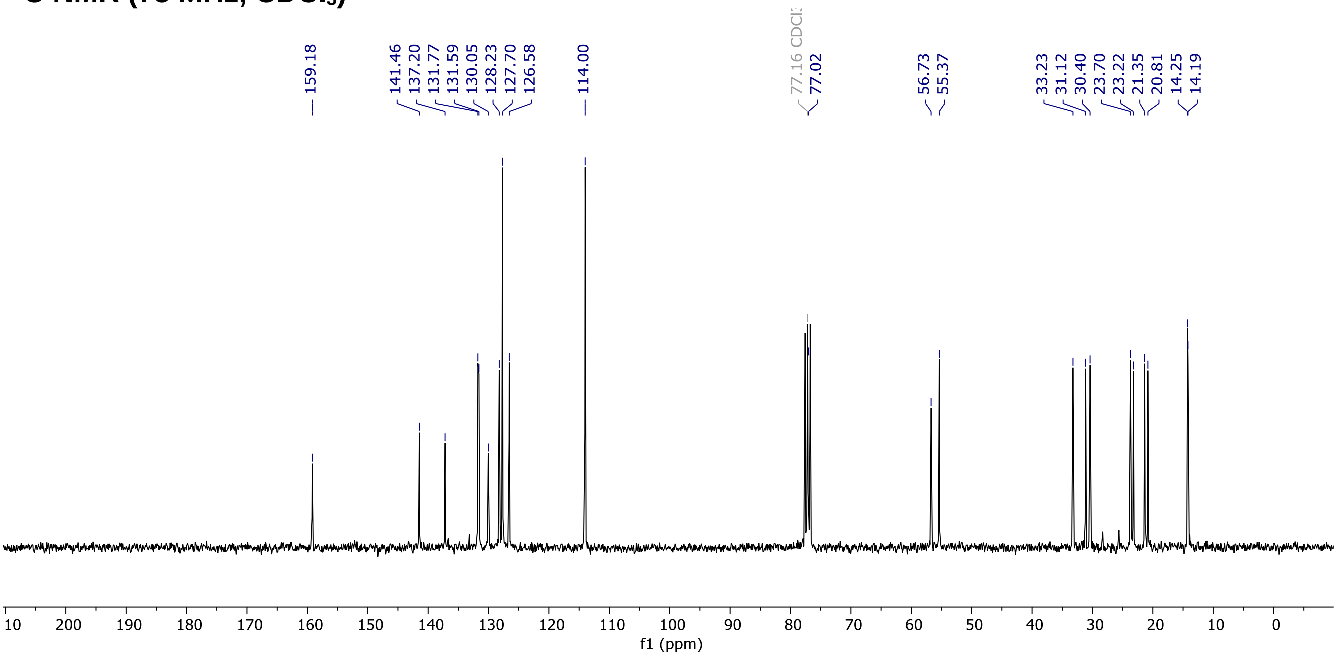

(*S**)-1-((*S**)-1,3-dimethyl-2-((Z)-pent-2-en-1-yl)cyclopent-2-en-1-yl)-2-methylprop-2-en-1-ol (12d)

^{1}H NMR (300 MHz, CDCl$_3$)

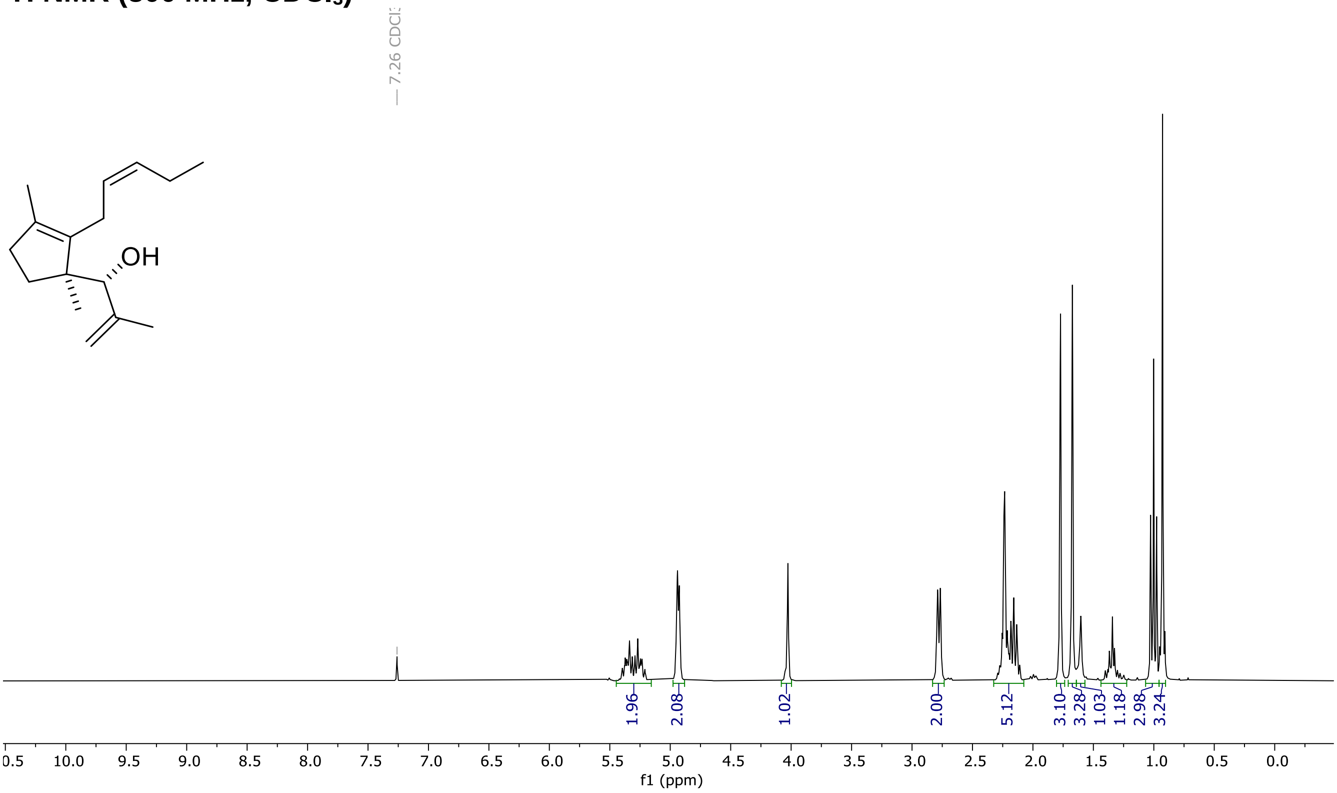

^{13}C NMR (75 MHz, CDCl$_3$)

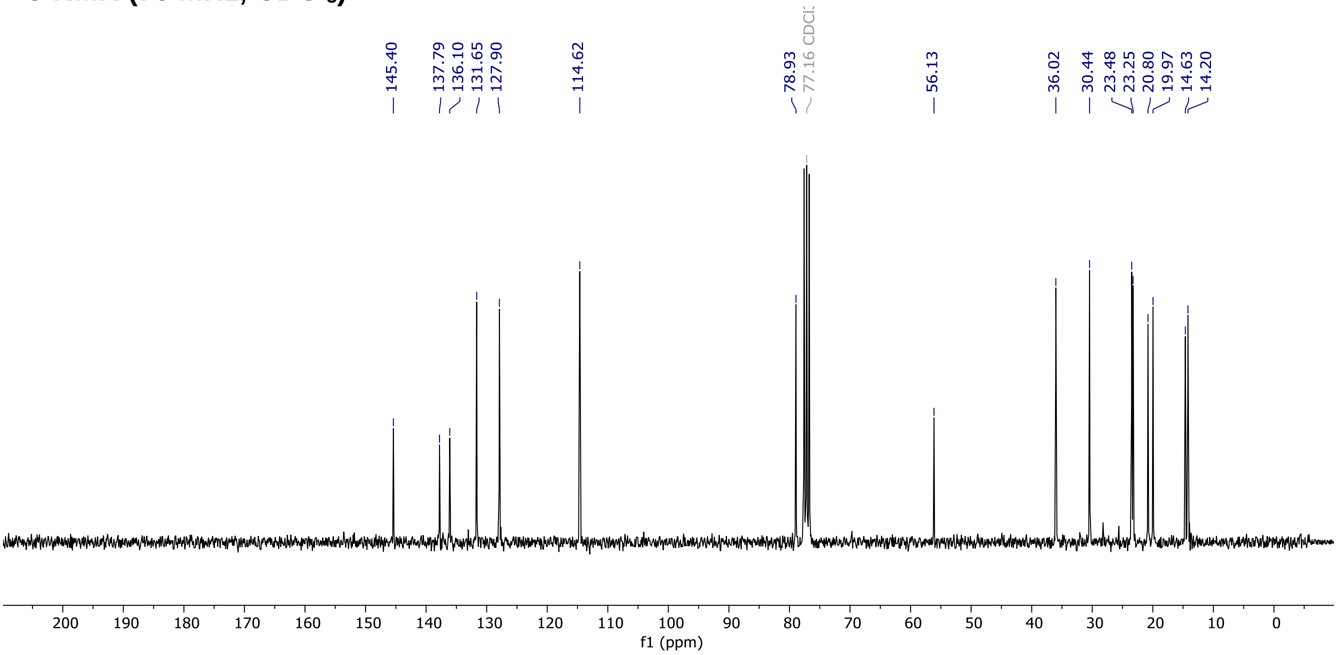

(5R,8R,9S,10R,13S,14S,17S)-5-((R)-hydroxy(p-tolyl)methyl)-10,13,17-trimethyl-3-propyl-2,5,6,7,8,9,10,11,12,13,14,15,16,17-tetradecahydro-1H-cyclopenta[a]phenanthren-17-ol (13)

^{1}H NMR (300 MHz, $CDCl_3$)

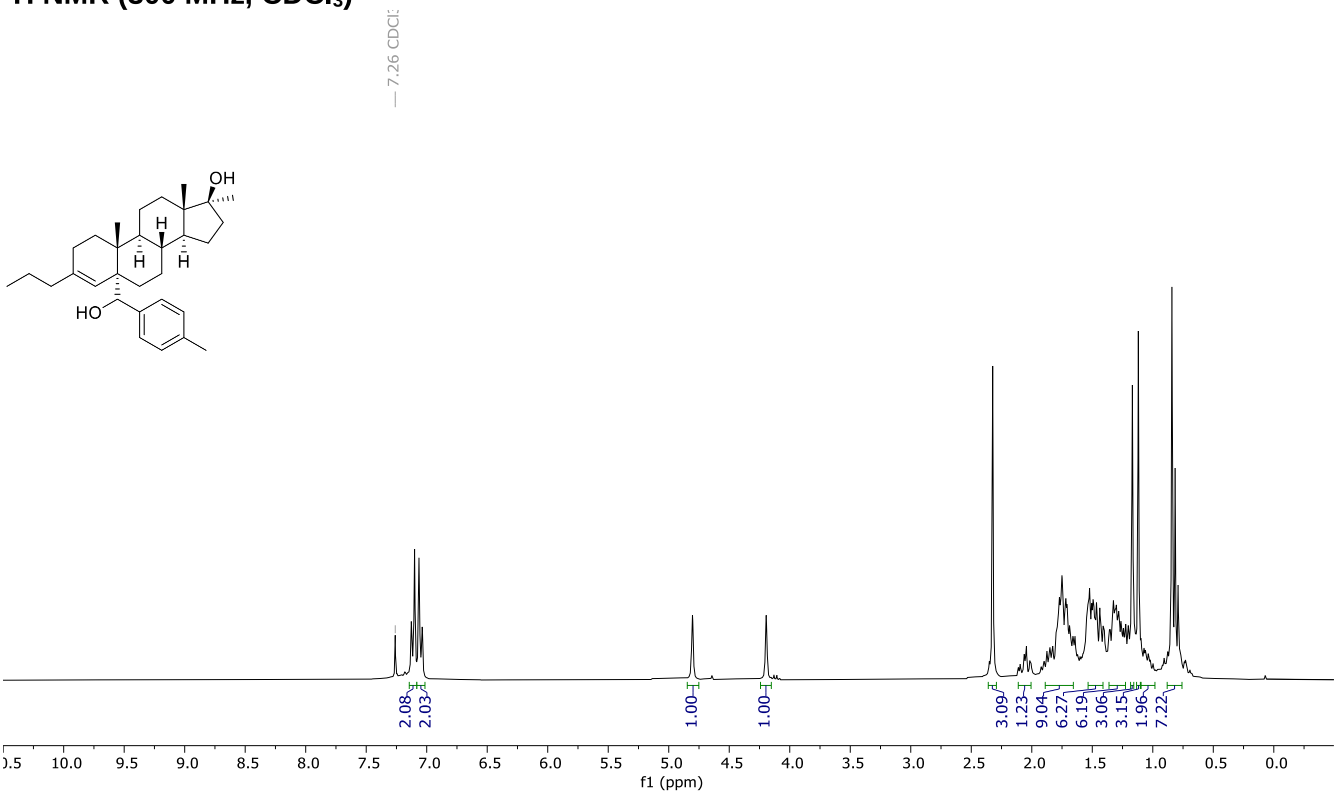

^{13}C NMR (75 MHz, $CDCl_3$)

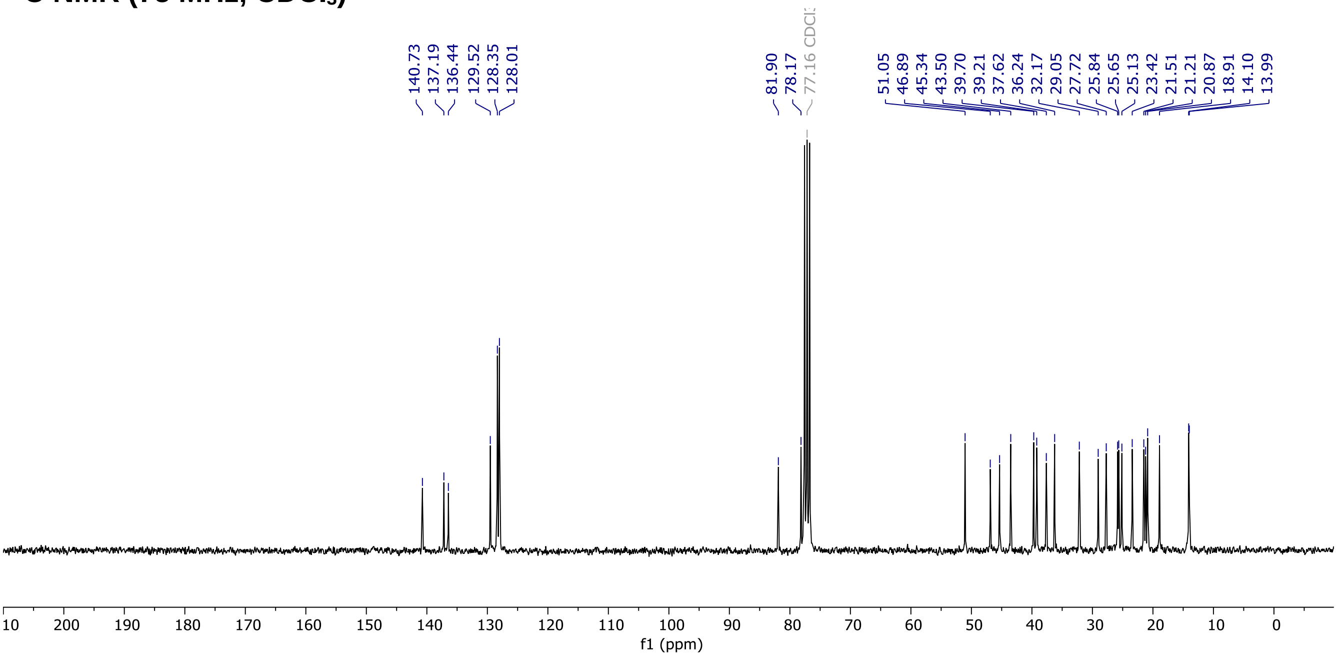

(*R)-((1*R**,4*R**)-4-allyl-3,6,6-trimethylcyclohex-2-en-1-yl)(4-nitrophenyl)methanol (16a)**

^{1}H NMR (300 MHz, $CDCl_3$)

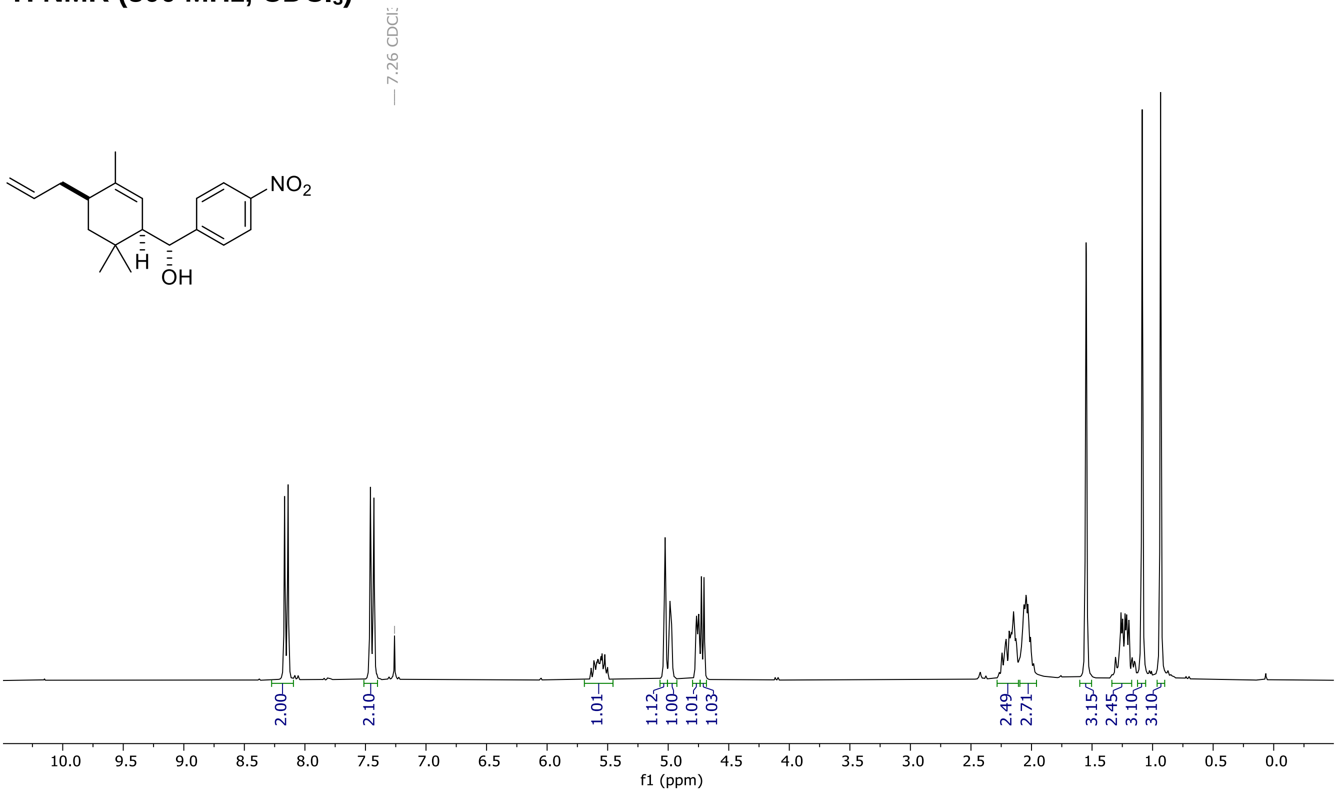

^{13}C NMR (75 MHz, $CDCl_3$)

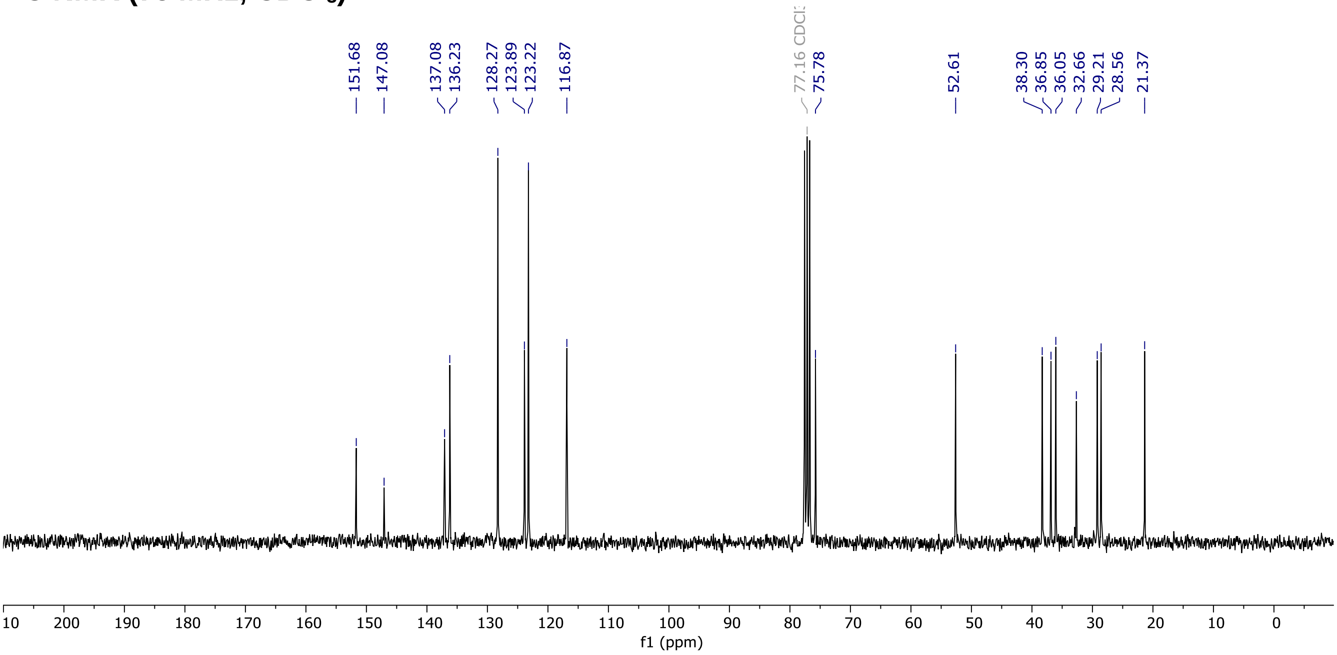

(*R)-((1*R**,4*R**)-4-allyl-3-(but-3-en-1-yl)-6,6-dimethylcyclohex-2-en-1-yl)(4-methoxyphenyl)methanol (16b)**

^{1}H NMR (300 MHz, $CDCl_3$)

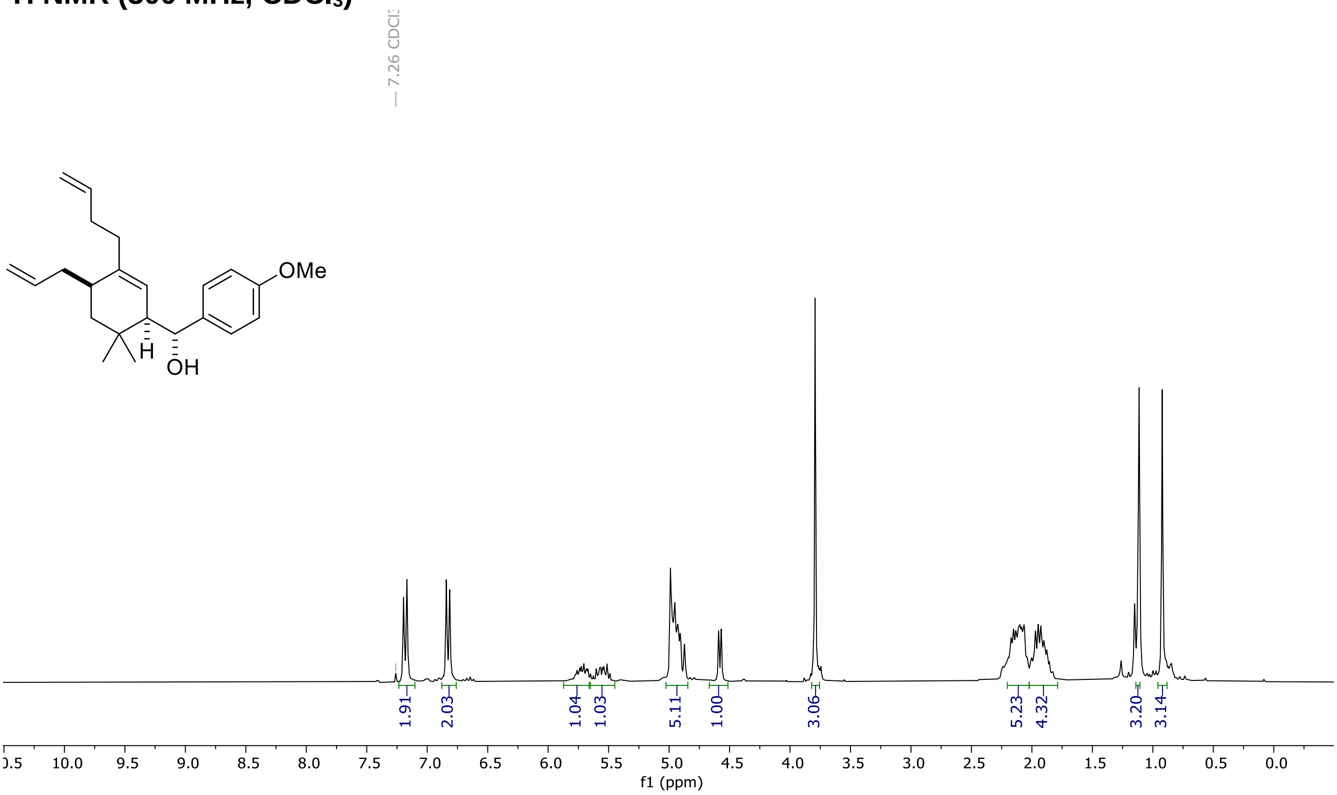

^{13}C NMR (75 MHz, $CDCl_3$)

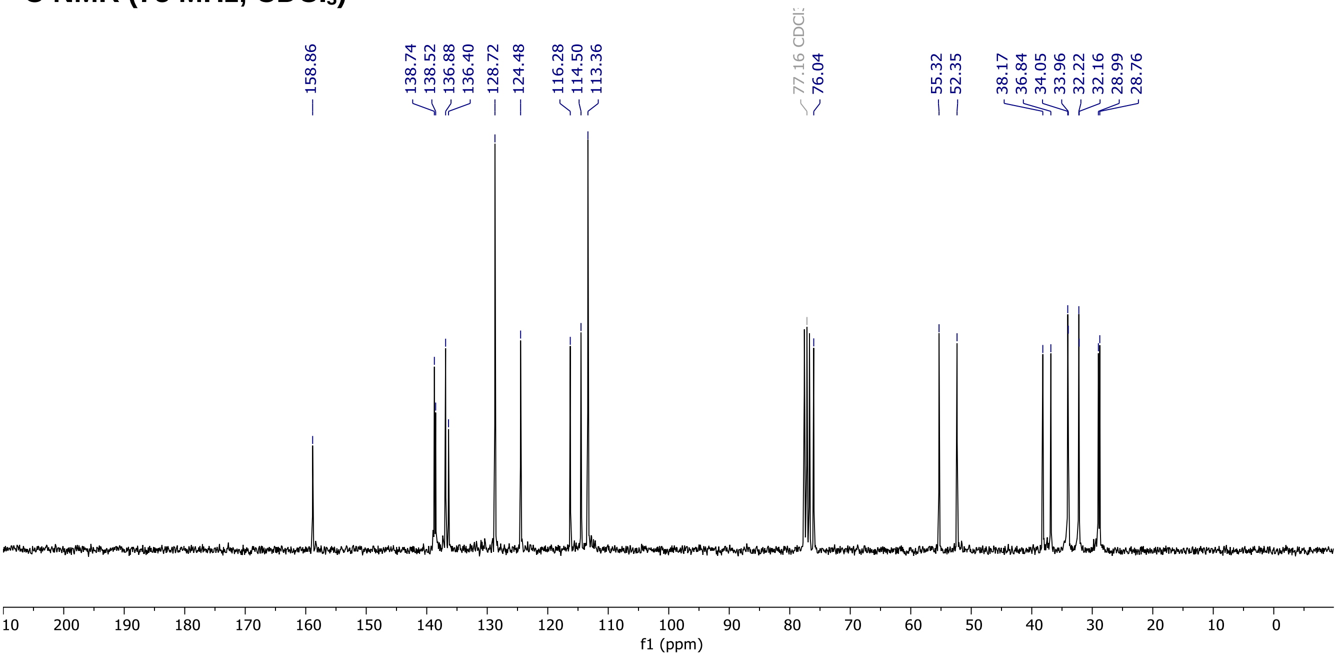

4-((*R)-((1*R**,4*R**)-4-allyl-3-cyclopropyl-6,6-dimethylcyclohex-2-en-1-yl)(hydroxy)methyl)benzonitrile (16c)**

^{1}H NMR (300 MHz, CDCl$_3$)

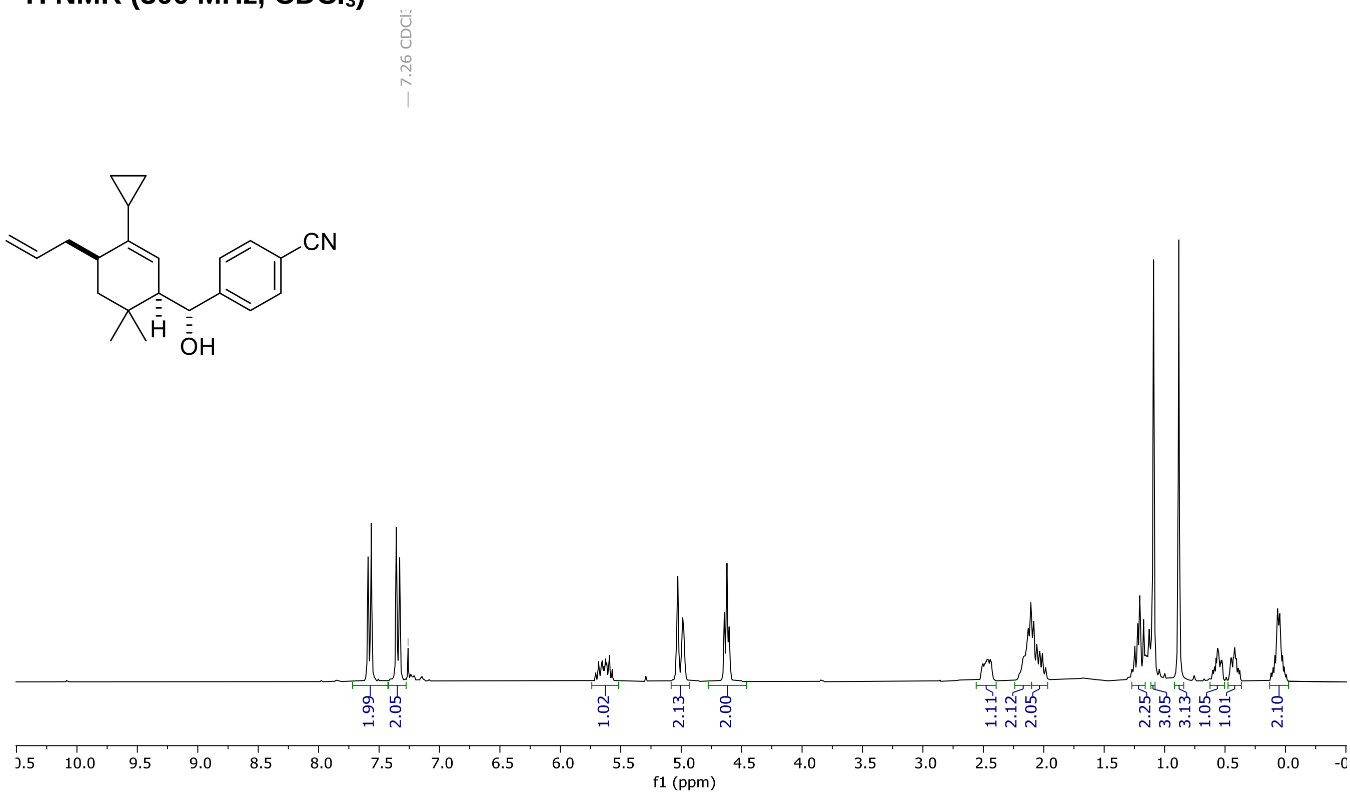

^{13}C NMR (75 MHz, CDCl$_3$)

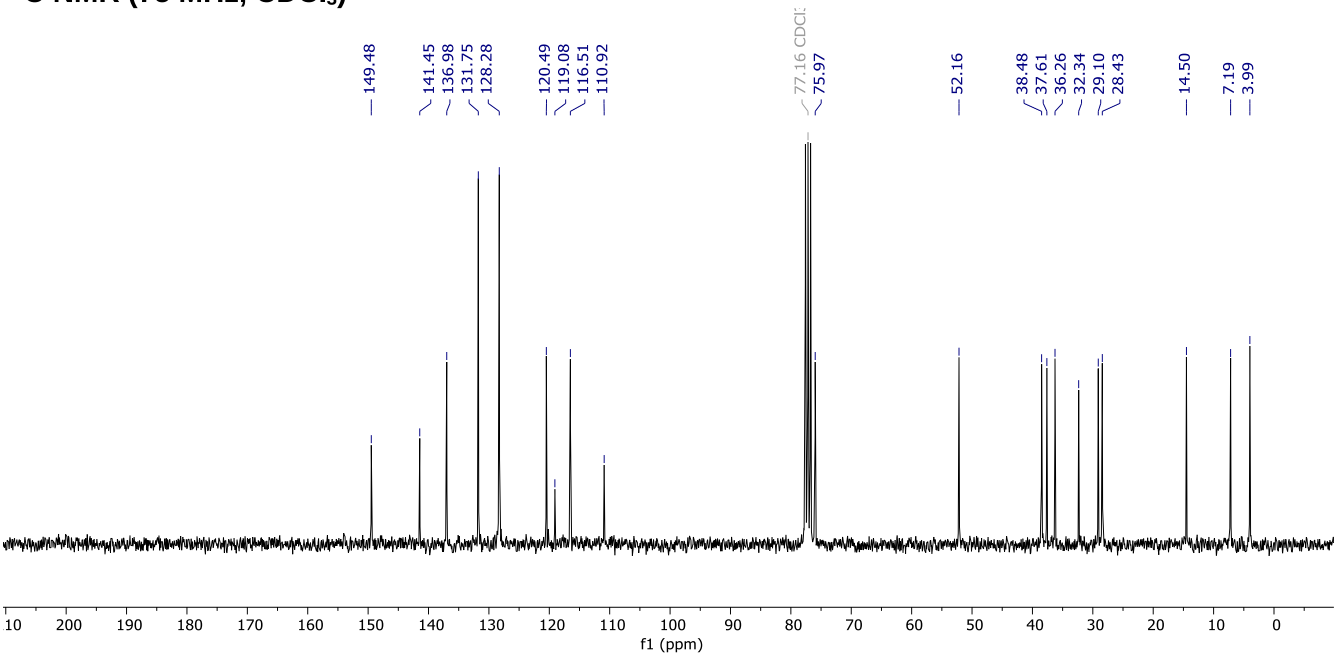

6-(1-hydroxy-4,4-dimethylcyclohex-2-en-1-yl)hexan-2-one (17)

^{1}H NMR (300 MHz, CDCl$_3$)

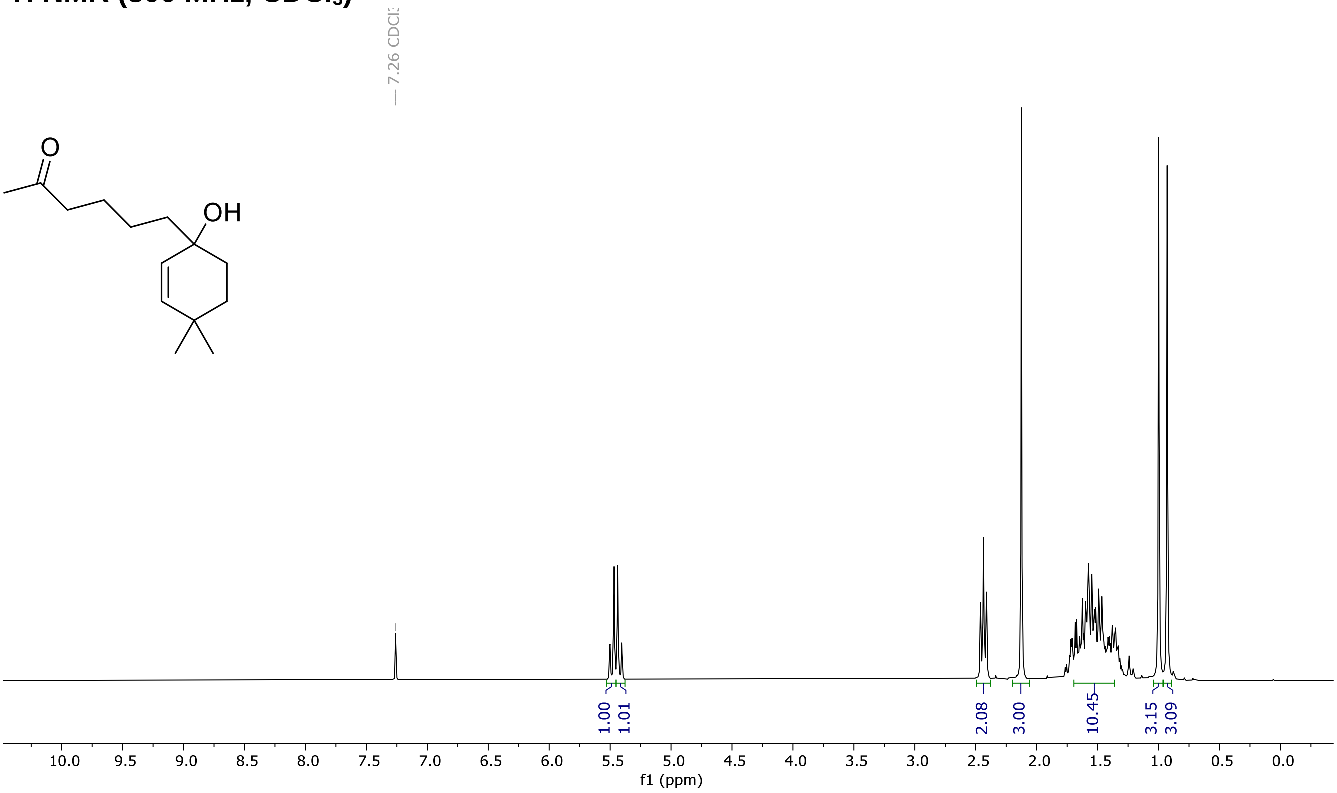

^{13}C NMR (75 MHz, CDCl$_3$)

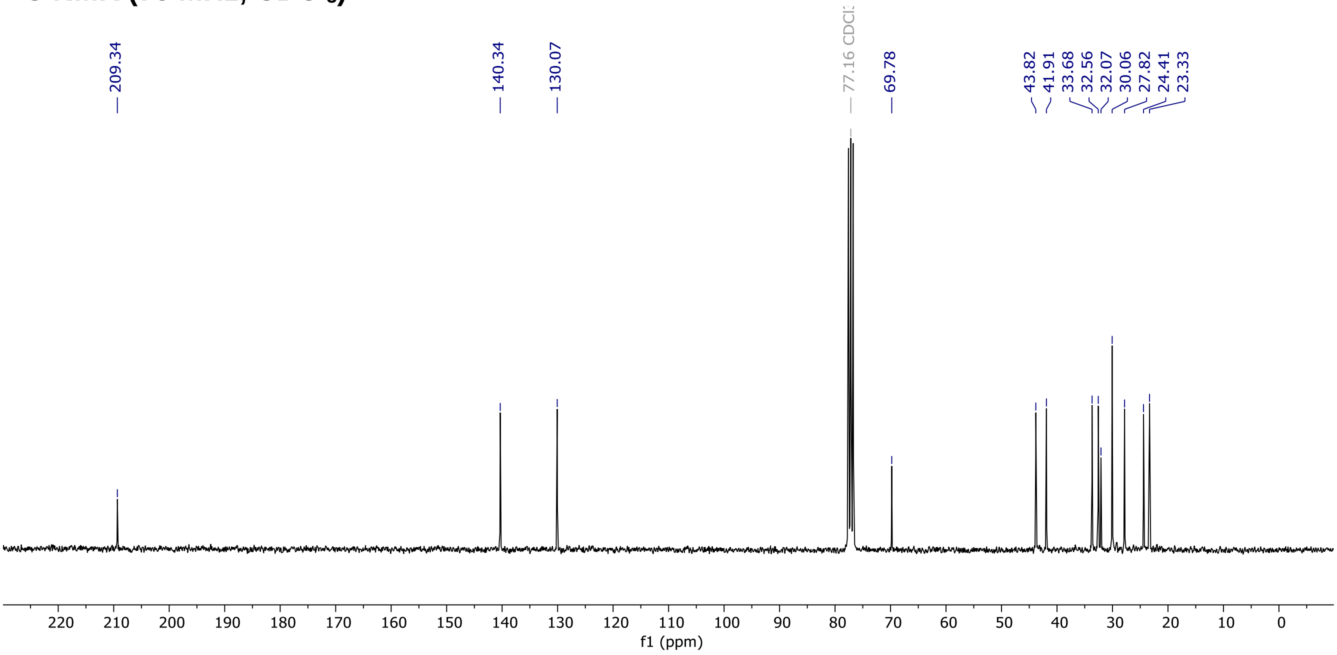

6-(4,4-dimethylcyclohex-1-en-1-yl)hexan-2-one (18)

^{1}H NMR (300 MHz, $CDCl_3$)

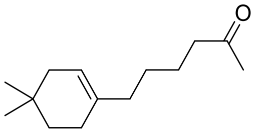

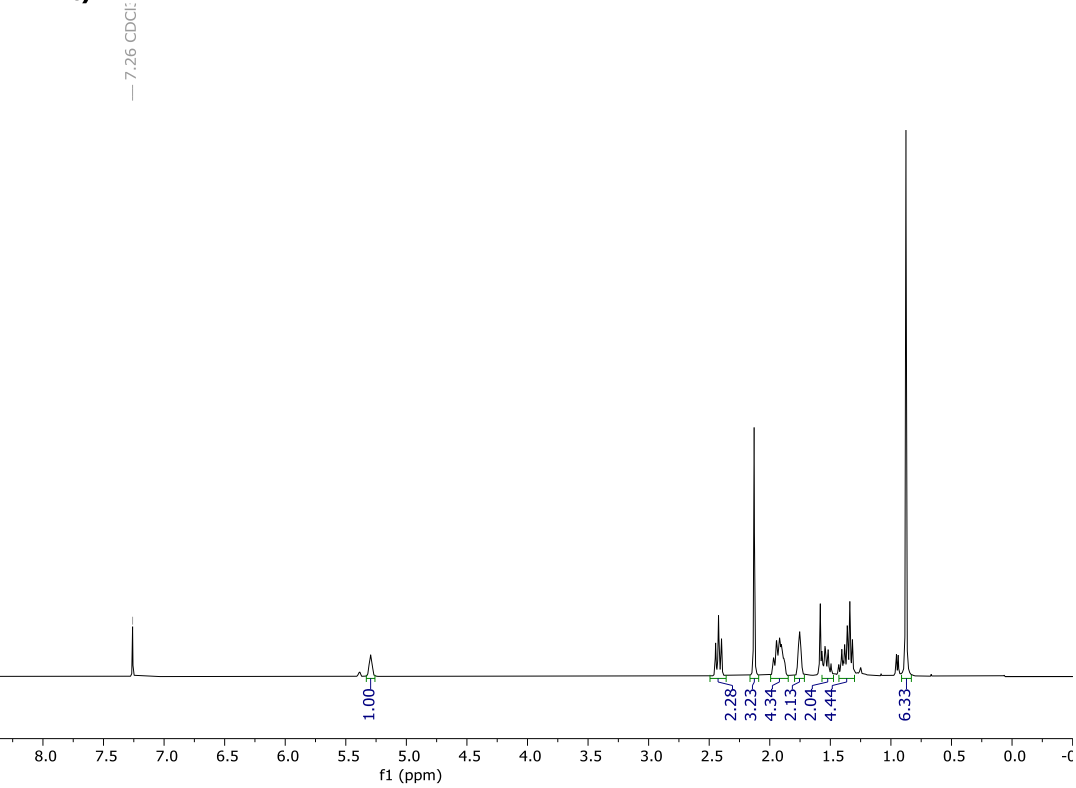

^{13}C NMR (75 MHz, $CDCl_3$)

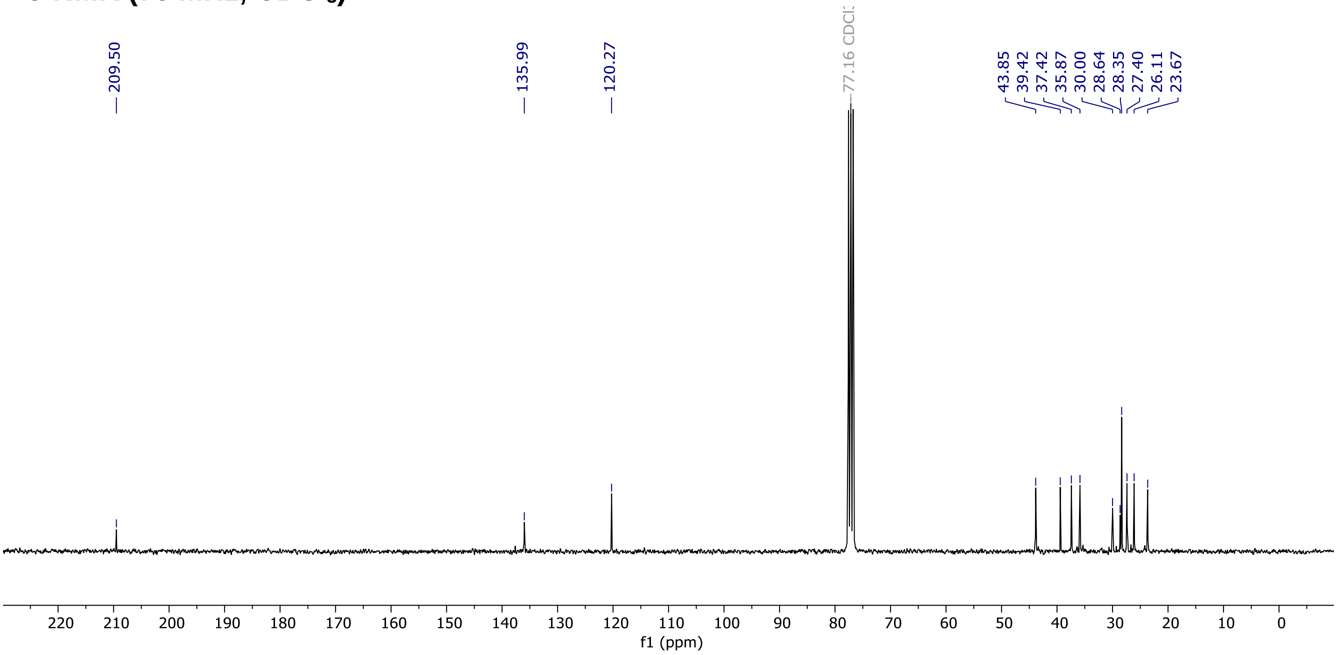

(1-cyclopropyl-4,4-dimethylcyclohex-2-en-1-yl)methanol (19)

^{1}H NMR (300 MHz, $CDCl_3$)

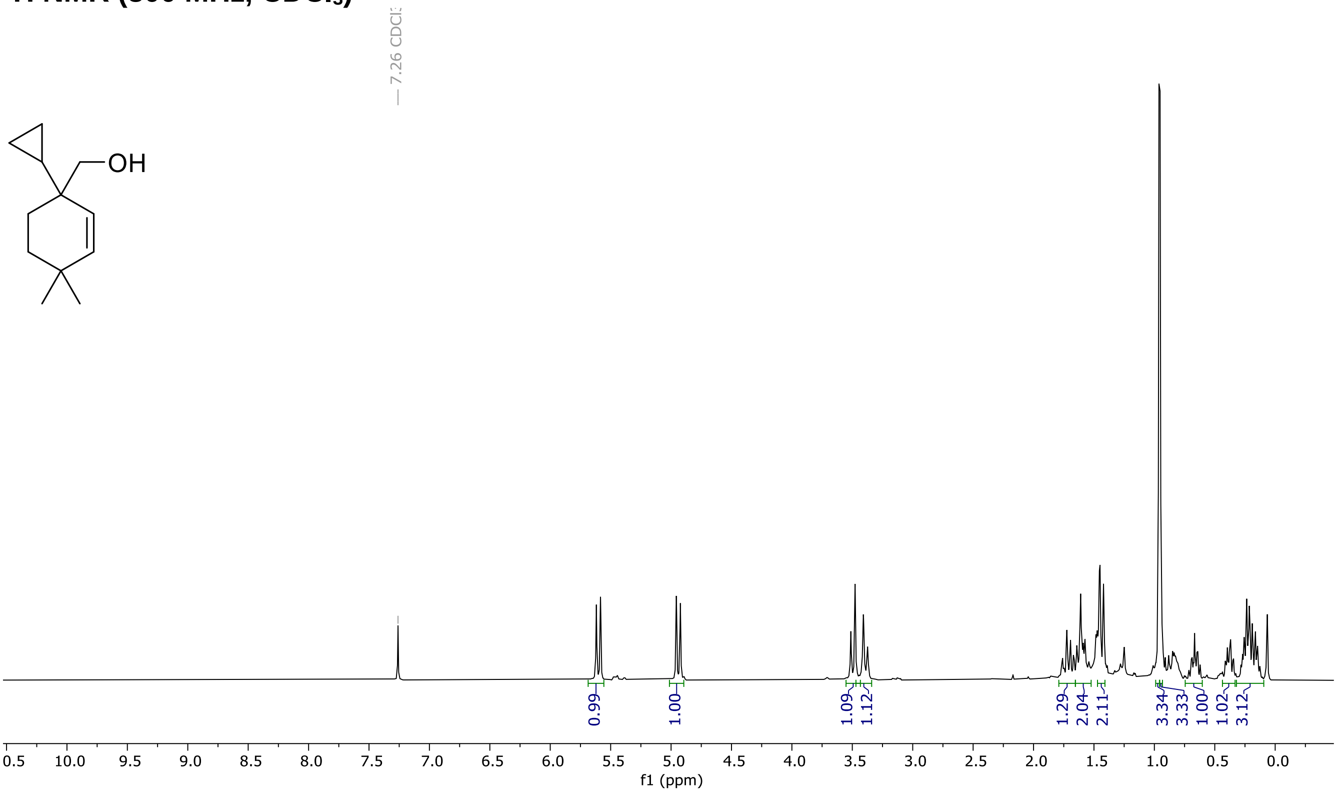

^{13}C NMR (75 MHz, $CDCl_3$)

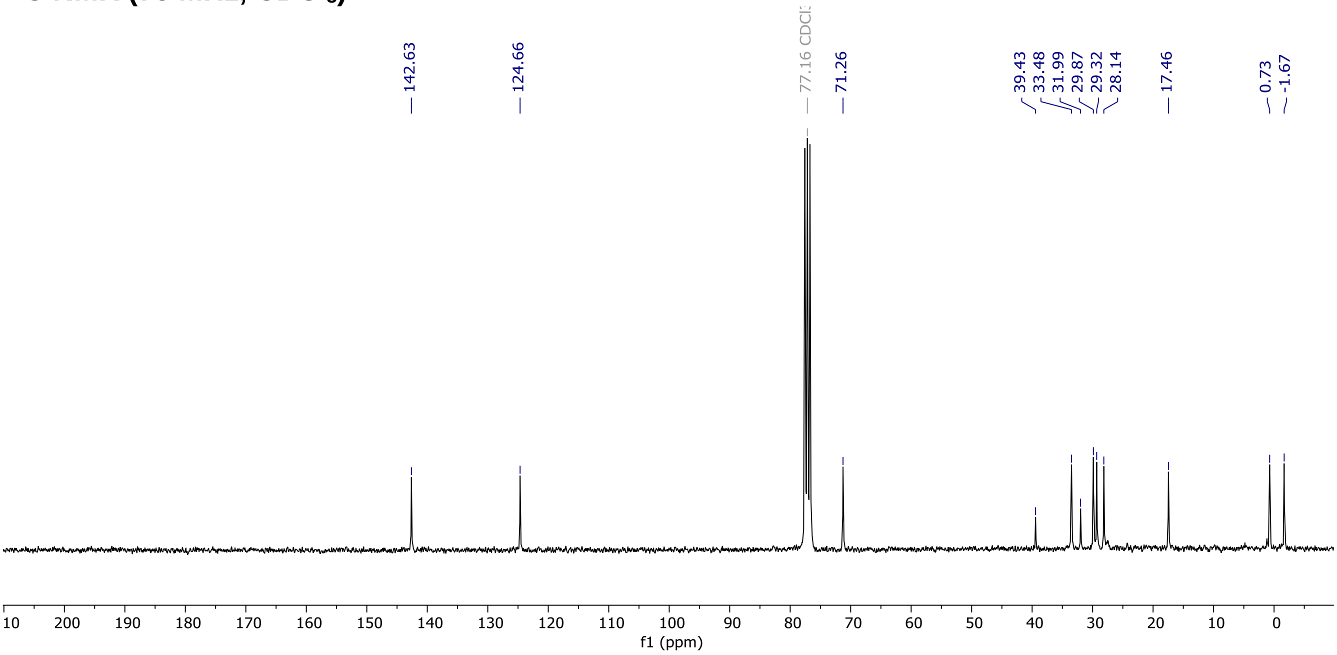

3-(4,4-dimethyl-1-propylcyclohex-2-en-1-yl)-3-phenylpropan-1-ol (20)

^{1}H NMR (300 MHz, $CDCl_3$)

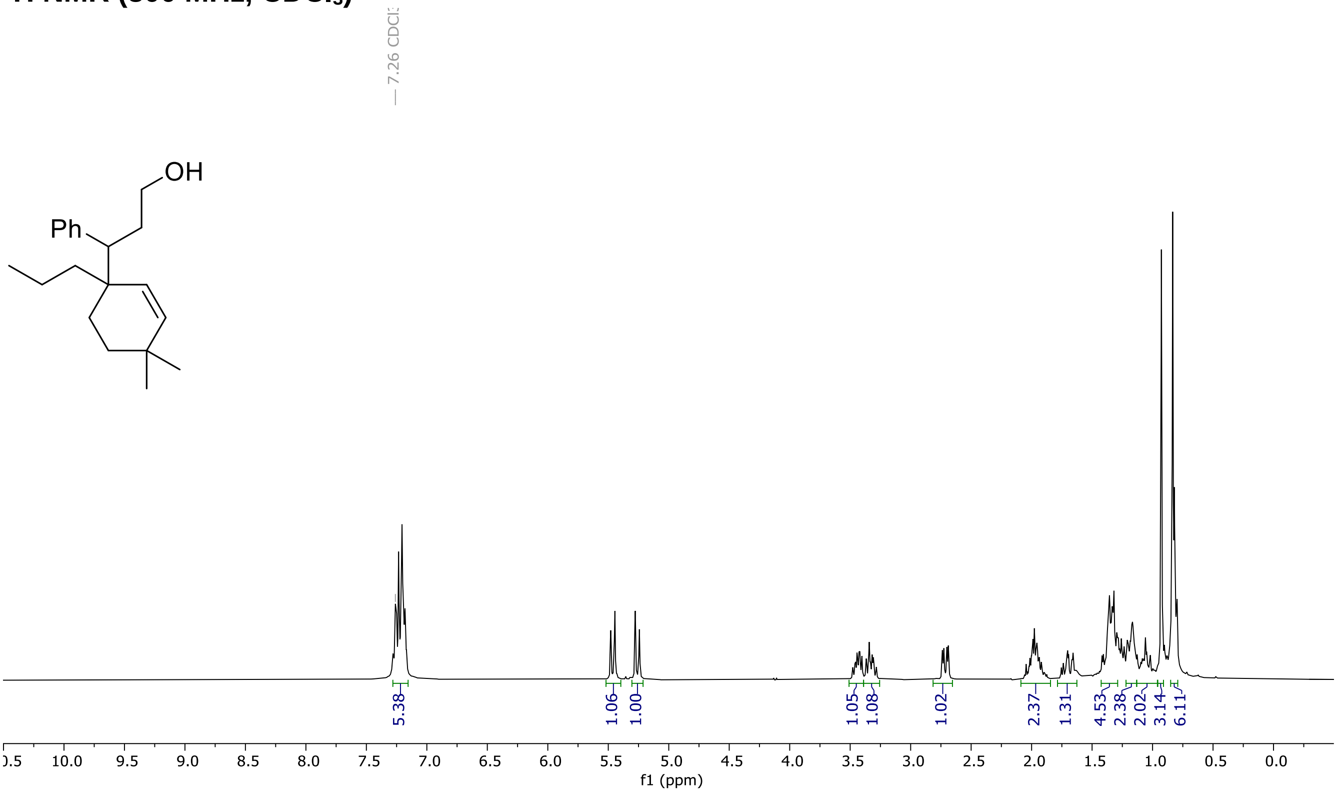

^{13}C NMR (75 MHz, $CDCl_3$)

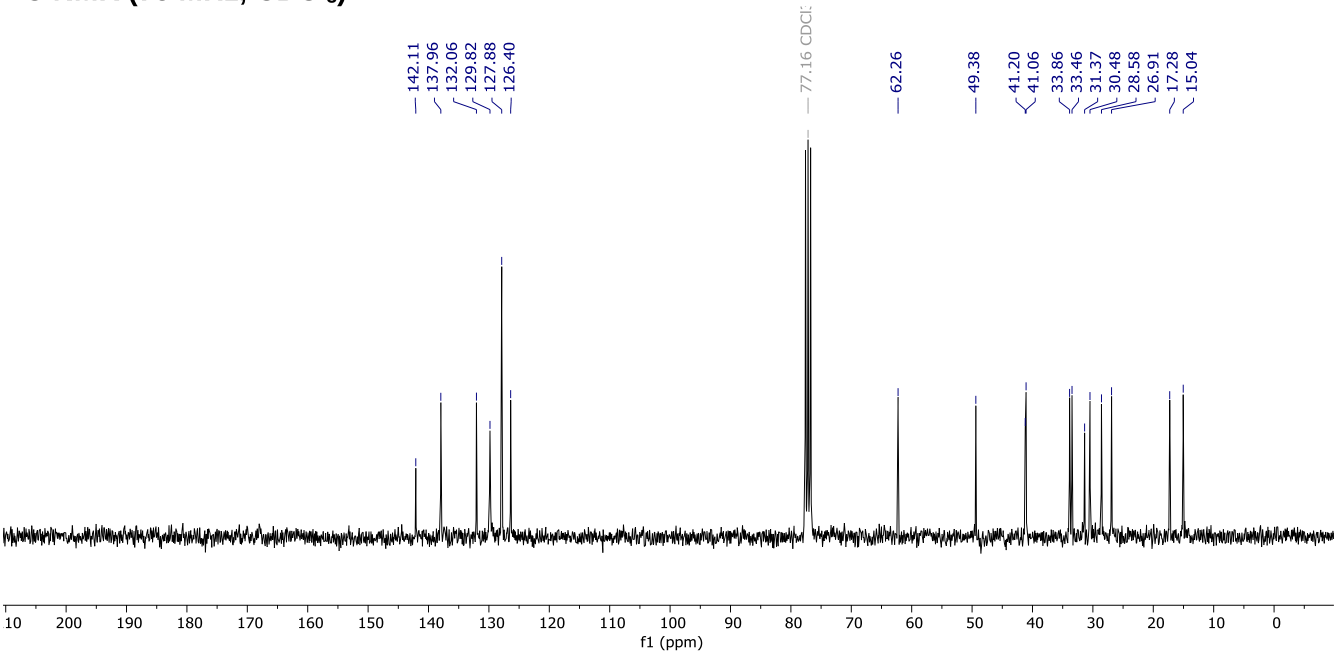

(4a*S**,9*R**,9a*R**)-1,1-dimethyl-4-methylene-2,3,4,4a,9,9a-hexahydro-1H-fluoren-9-ol (21)

^{1}H NMR (300 MHz, $CDCl_3$)

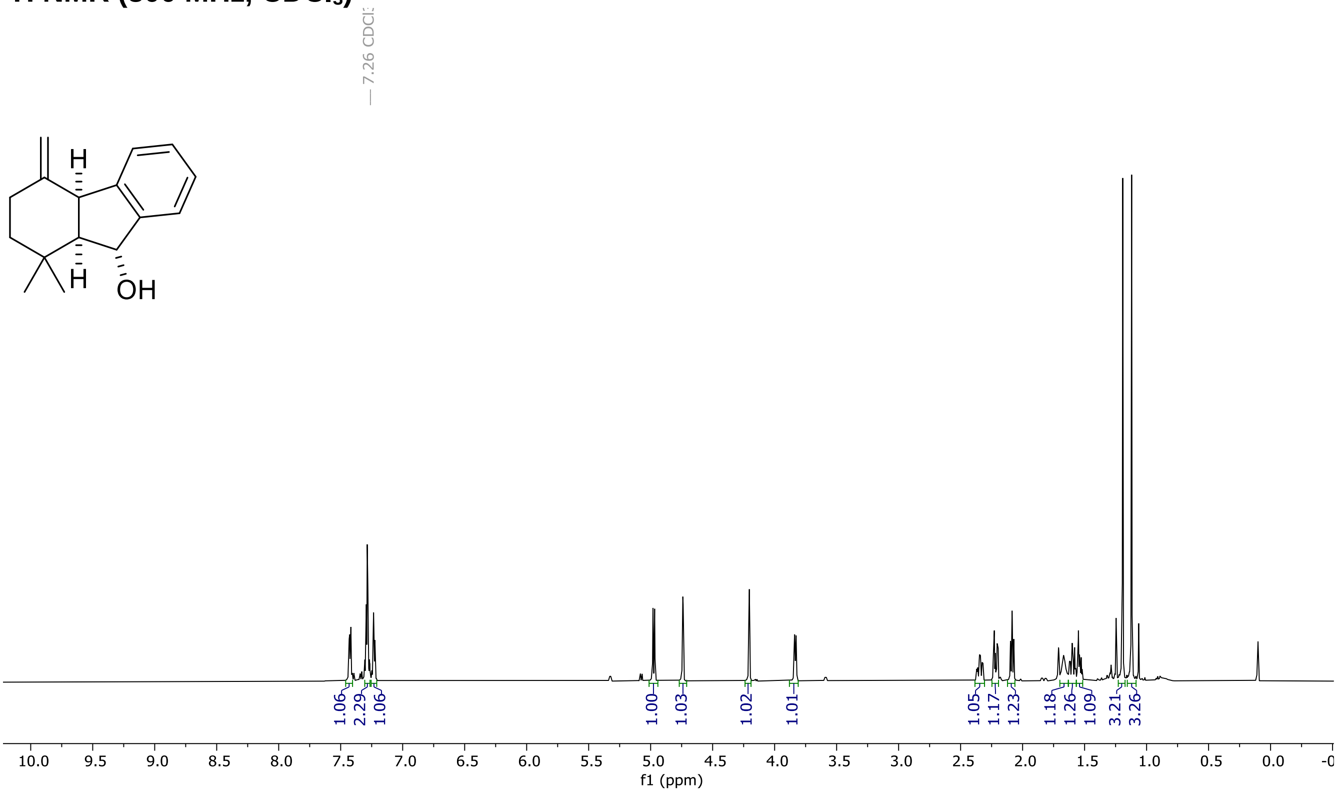

^{13}C NMR (75 MHz, $CDCl_3$)

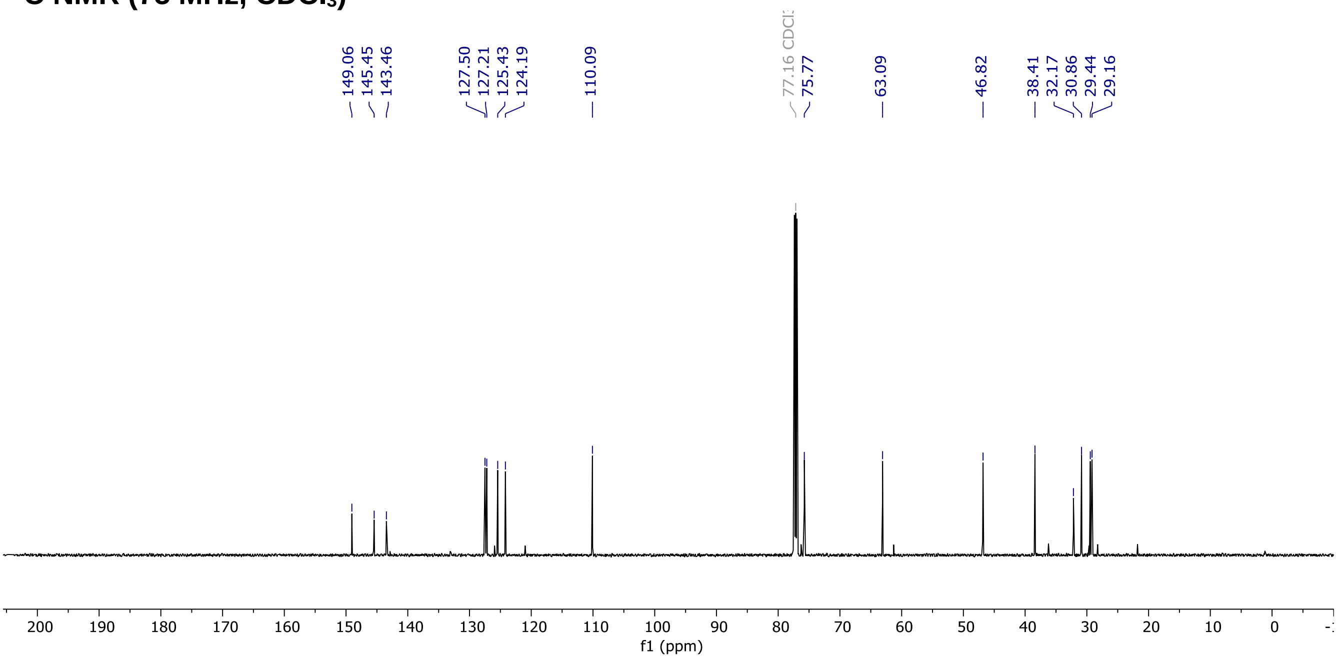

(1*S**,8a*S**)-7-methyl-1-phenyl-1,5,6,8a-tetrahydro-3H-oxazolo[3,4-a]pyridin-3-one (22)

^{1}H NMR (300 MHz, $CDCl_3$)

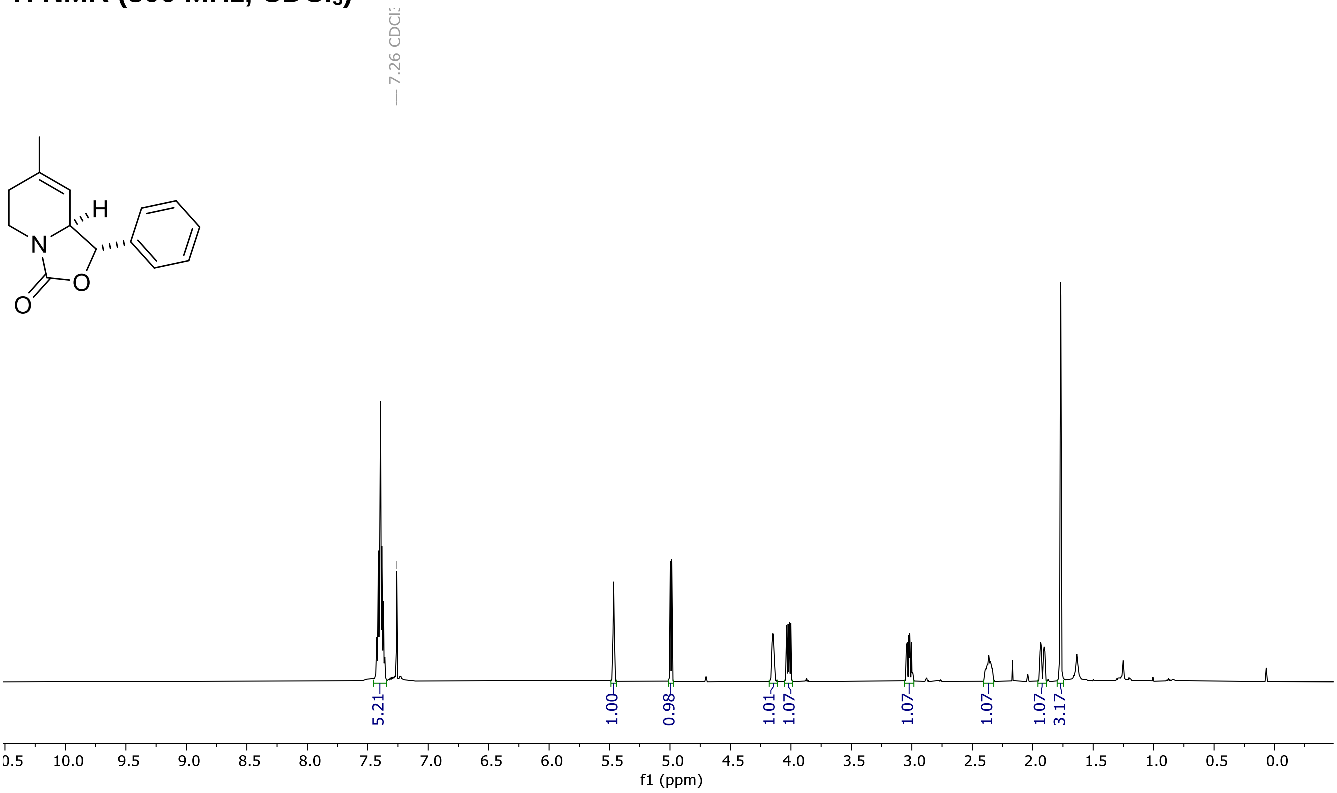

^{13}C NMR (75 MHz, $CDCl_3$)

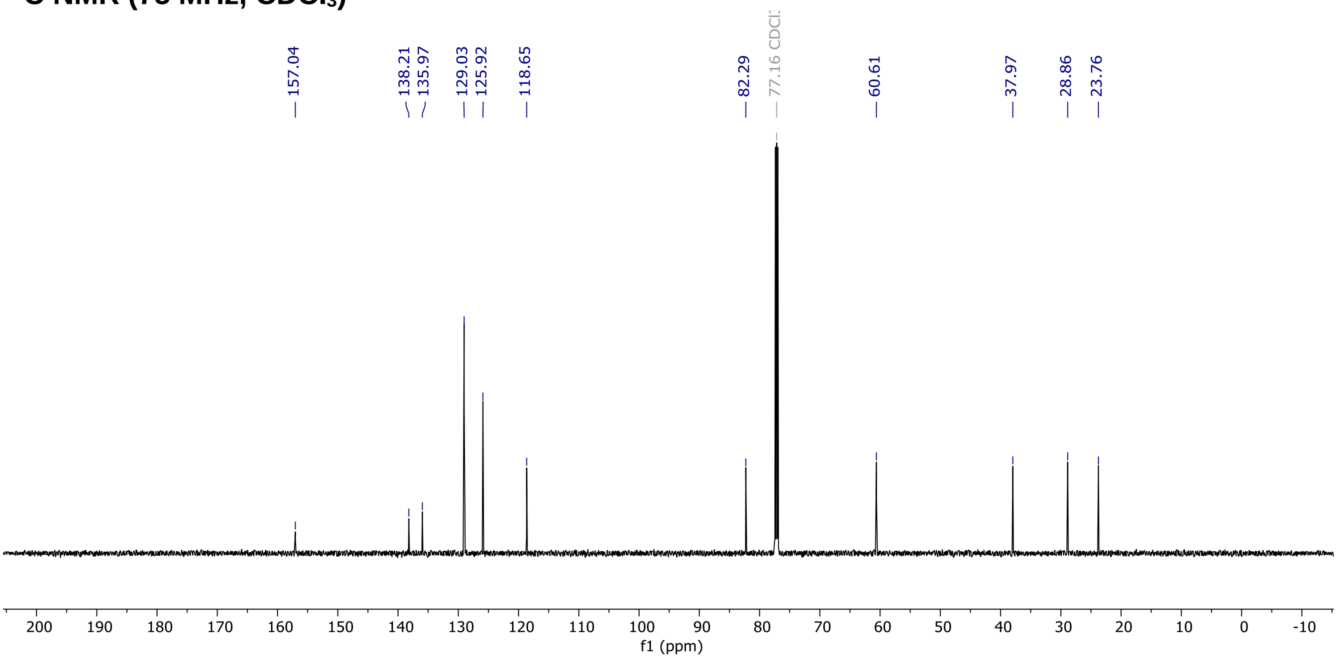

(1*R)-((3*R**)-2,2-dimethyl-2,3,5,6,9,9a-hexahydro-1H-benzo[7]annulen-3-yl)(4-methoxyphenyl)methanol (23)**

^{1}H NMR (300 MHz, $CDCl_3$)

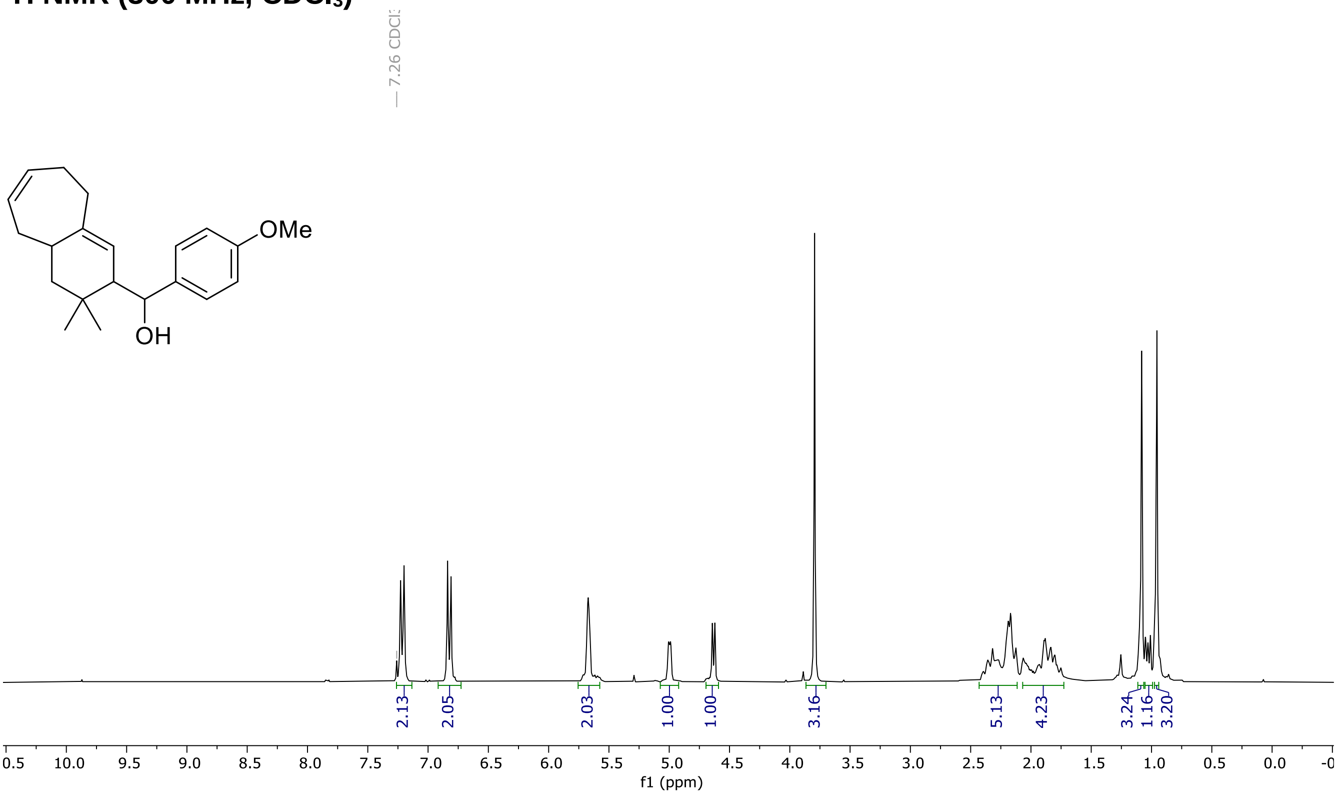

^{13}C NMR (75 MHz, $CDCl_3$)

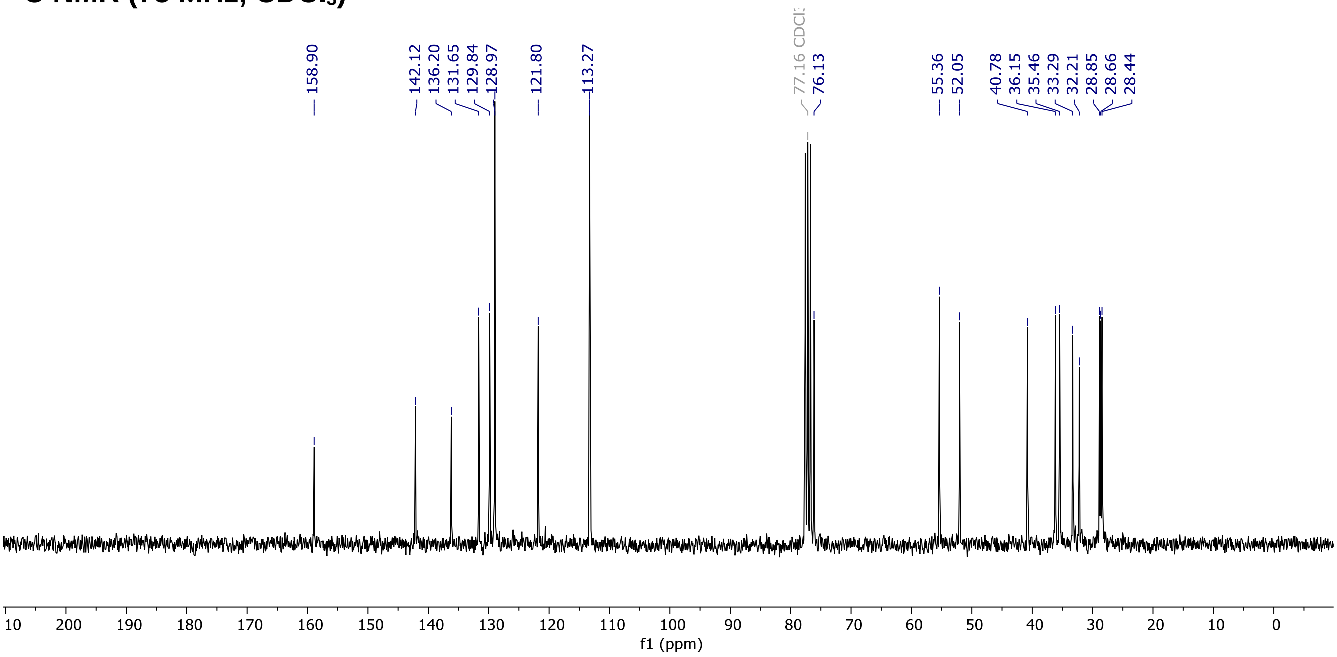

(*E*)-2-(2-cyclopropylhept-3-en-2-yl)-4,4,5,5-tetramethyl-1,3,2-dioxaborolane 28

^{1}H NMR (400 MHz, $CDCl_3$)

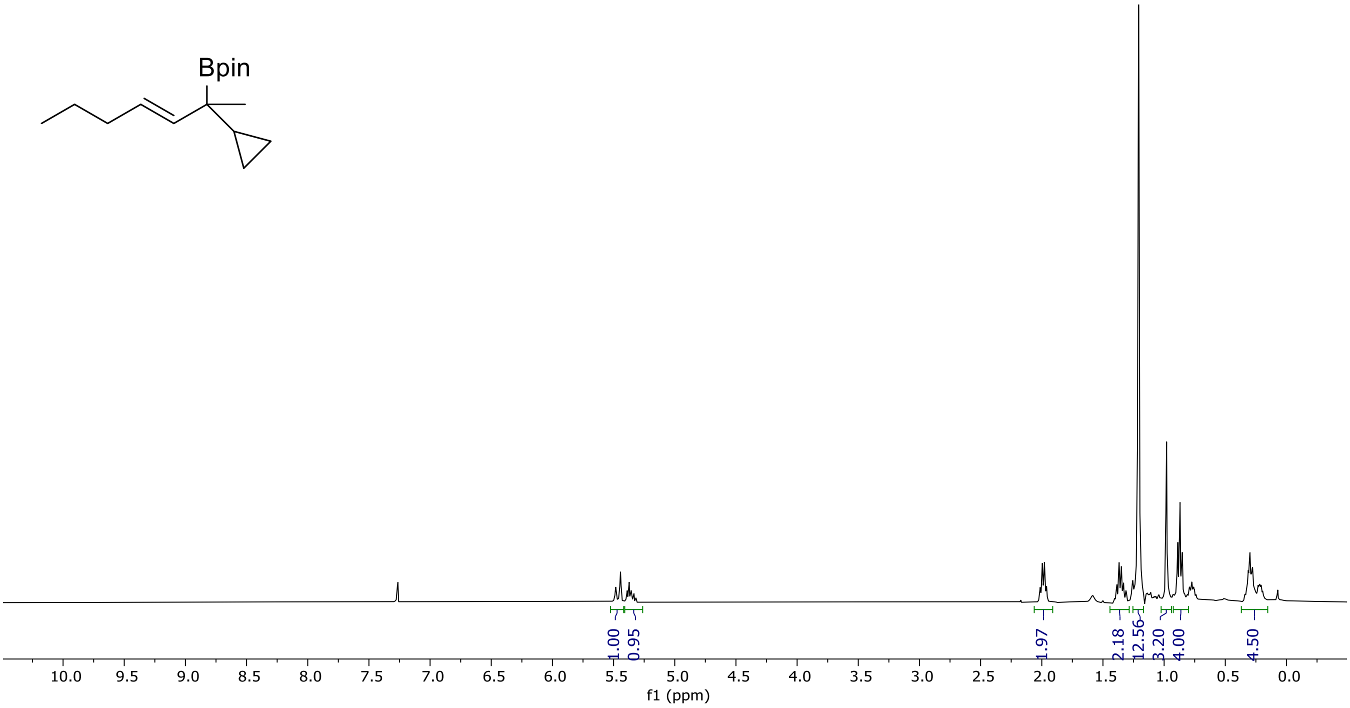

^{13}C NMR (100 MHz, $CDCl_3$)

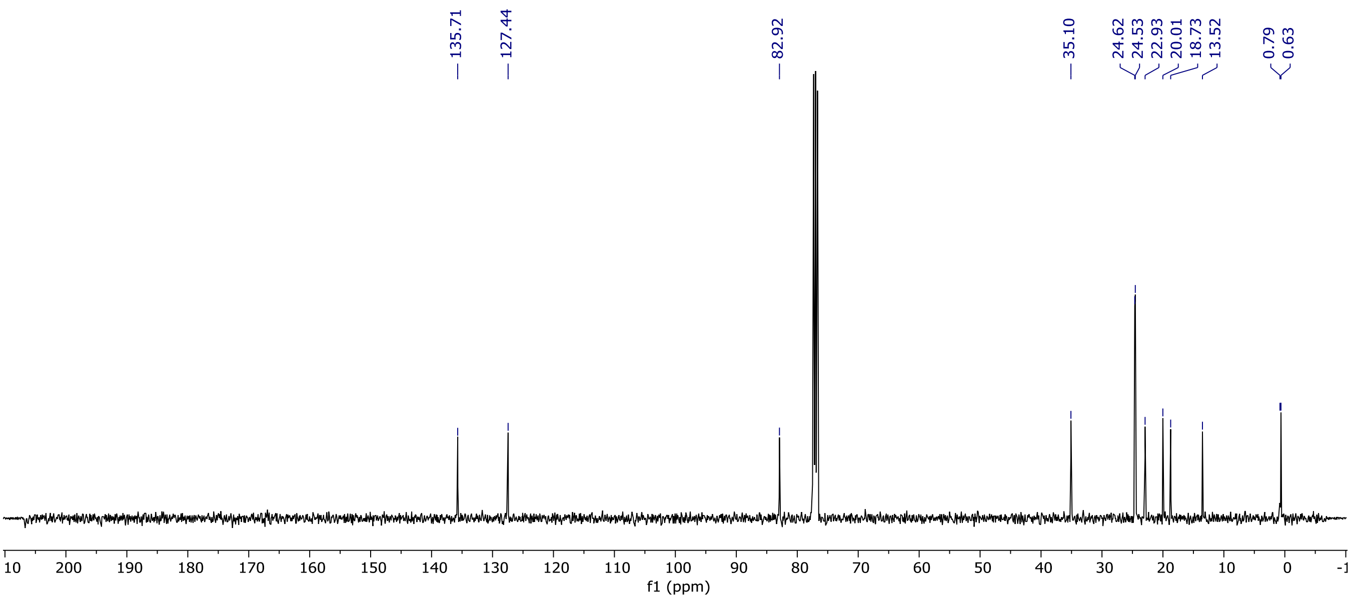

^{11}B NMR (129 MHz, $CDCl_3$)

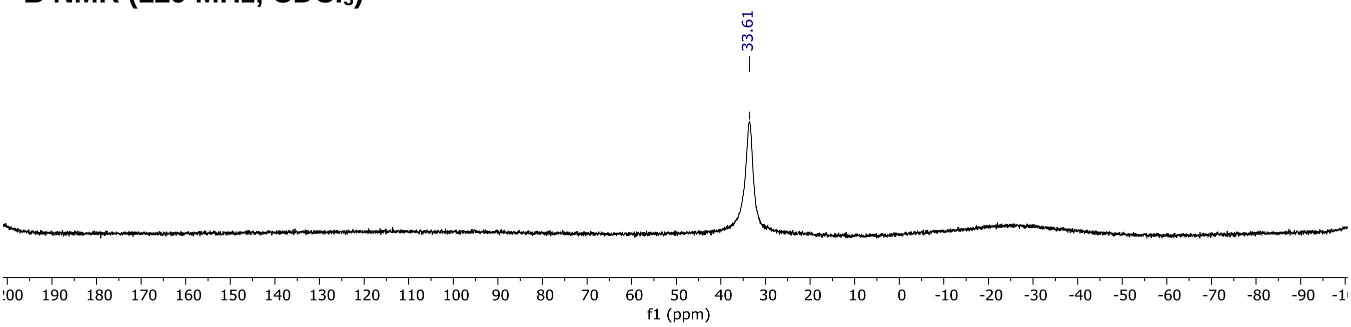

(1*S**,2*S**)-1-(4-Methoxyphenyl)-4-methyl-2-propylpent-3-en-1-ol 30

^{1}H NMR (400 MHz, $CDCl_3$)

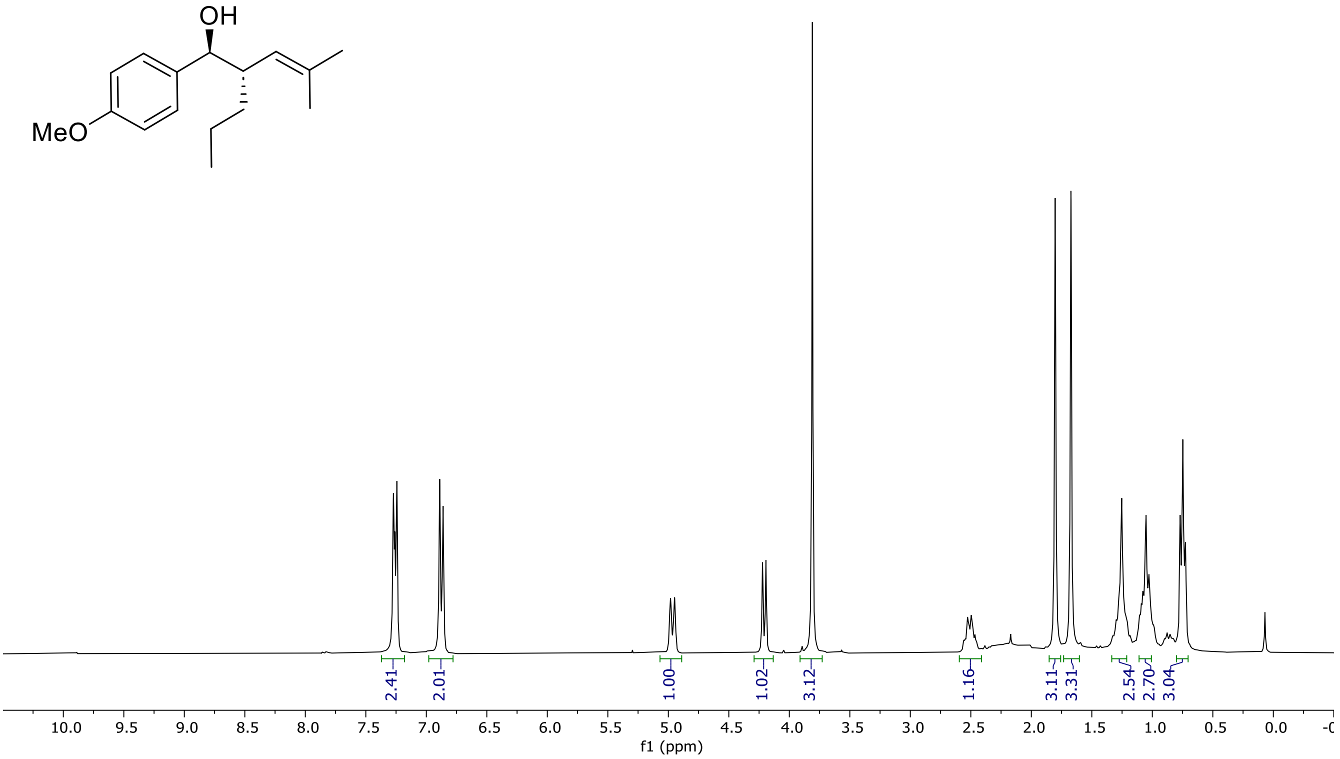

^{13}C NMR (100 MHz, $CDCl_3$)

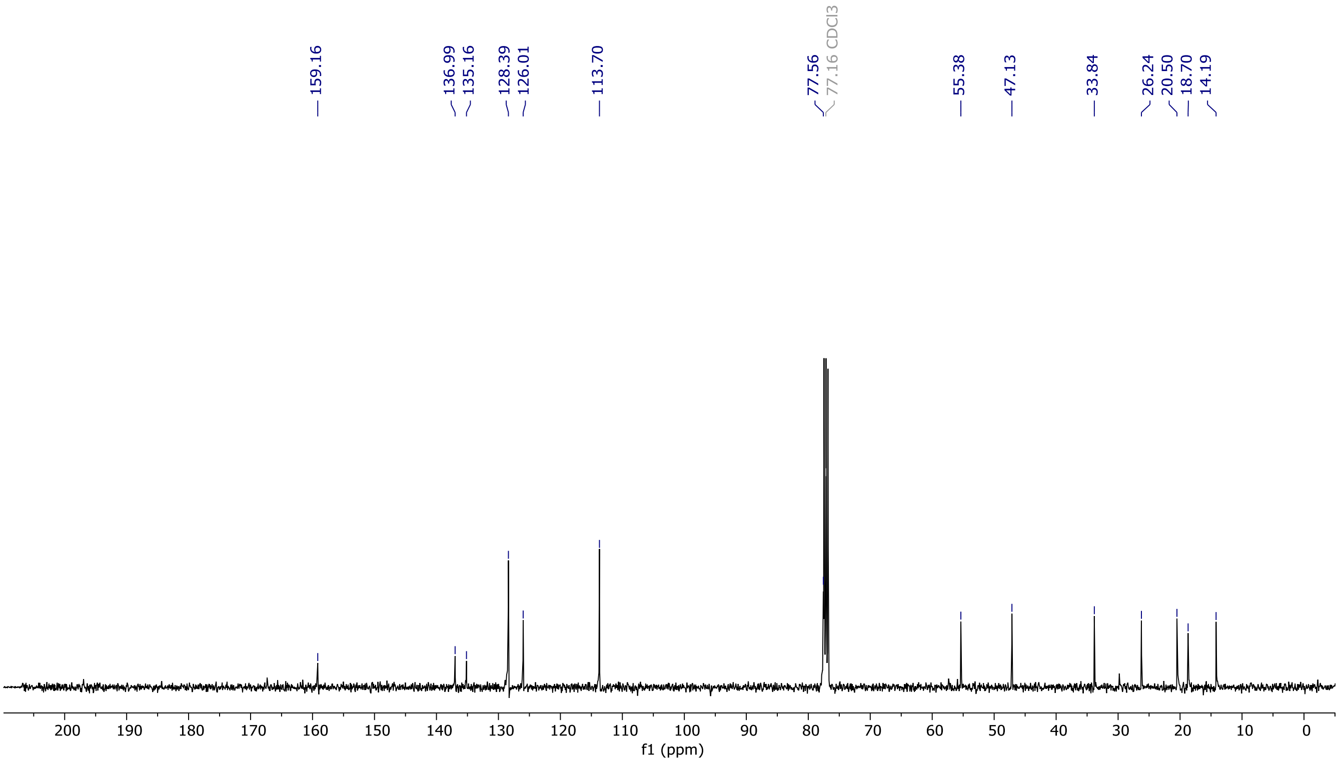

(*E*)-1-(4-Methoxyphenyl)-2,2-dimethylhept-3-en-1-ol 31

^{1}H NMR (400 MHz, $CDCl_3$)

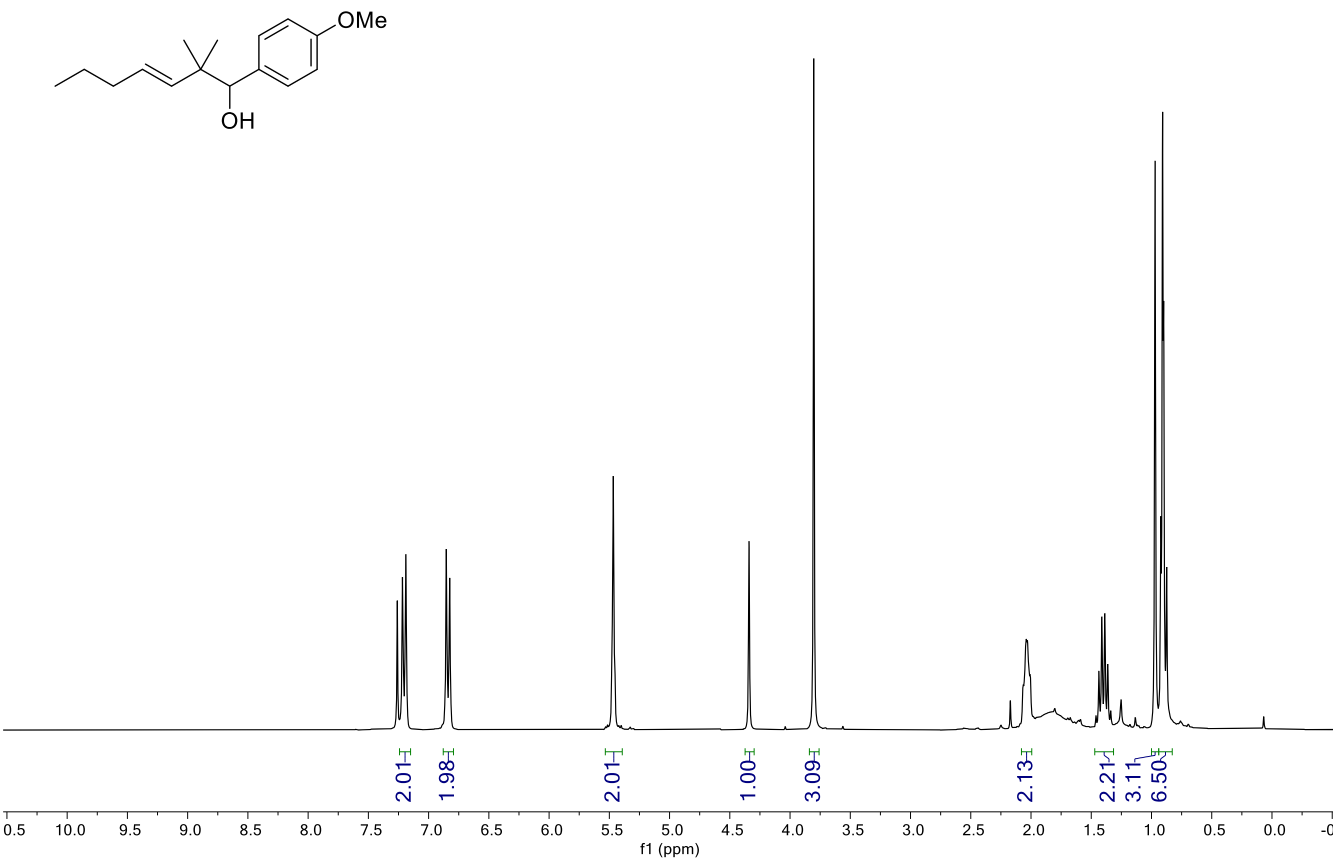

^{13}C NMR (100 MHz, $CDCl_3$)

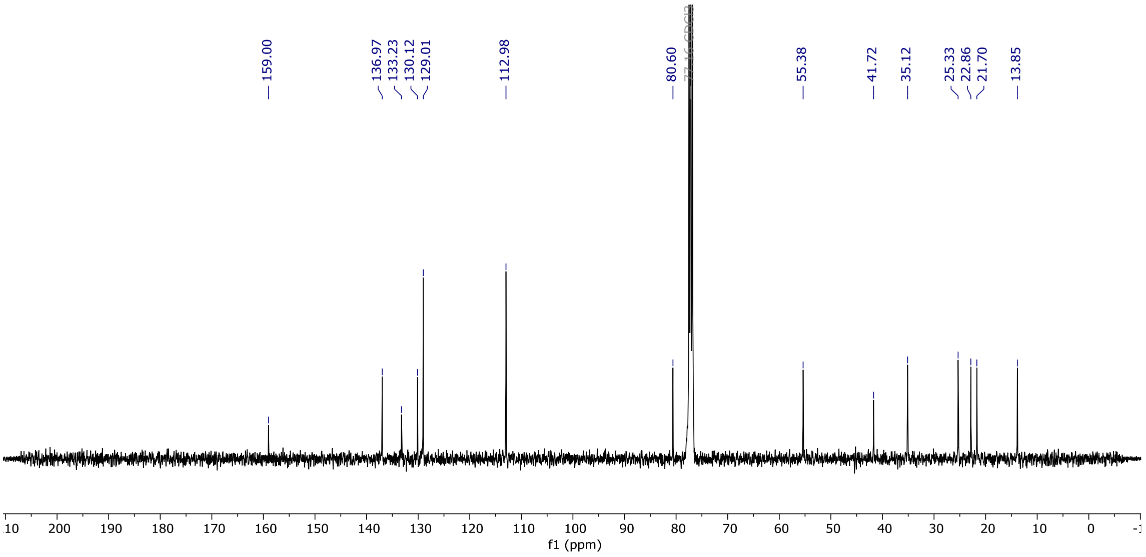

Supplement: SC-015-D4SC06537A-s001 [file SC-015-D4SC06537A-s001.pdf]
